# Supplementary figures and images for: Peptidoglycan precursor synthesis along the sidewall of pole-growing mycobacteria (part 2 of 3)
Source: eLife. 2018 Sep 10;7:e37243. doi: 10.7554/eLife.37243 (PMC6191288; doi:10.7554/eLife.37243)

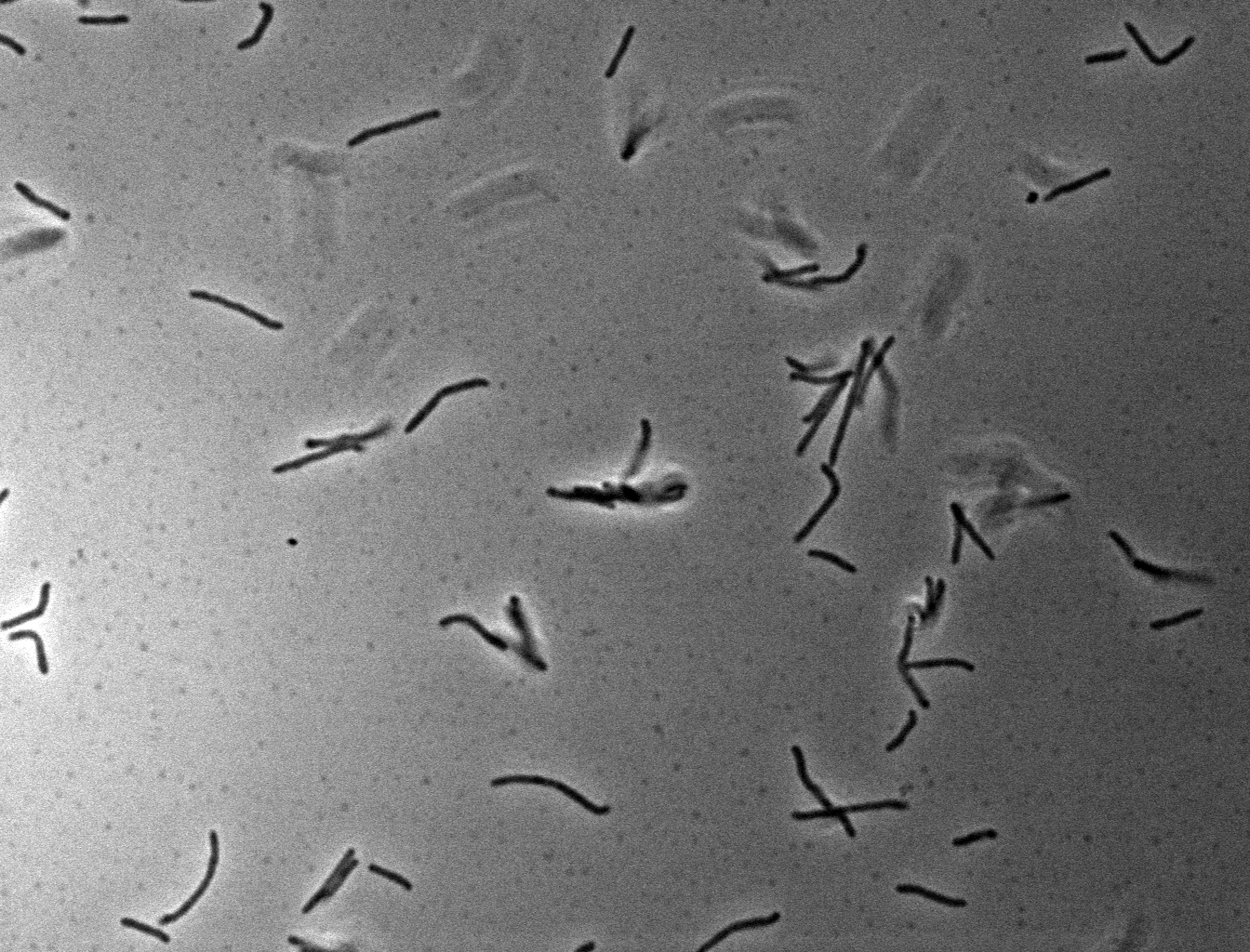

Supplement: Figure 2—source data 1. [file elife-37243-fig2-data1.zip › Figure 2 source data/Figure 2 source data-conventional microscopy (OalkTMM + RADA)/1. Phase/9.tif]

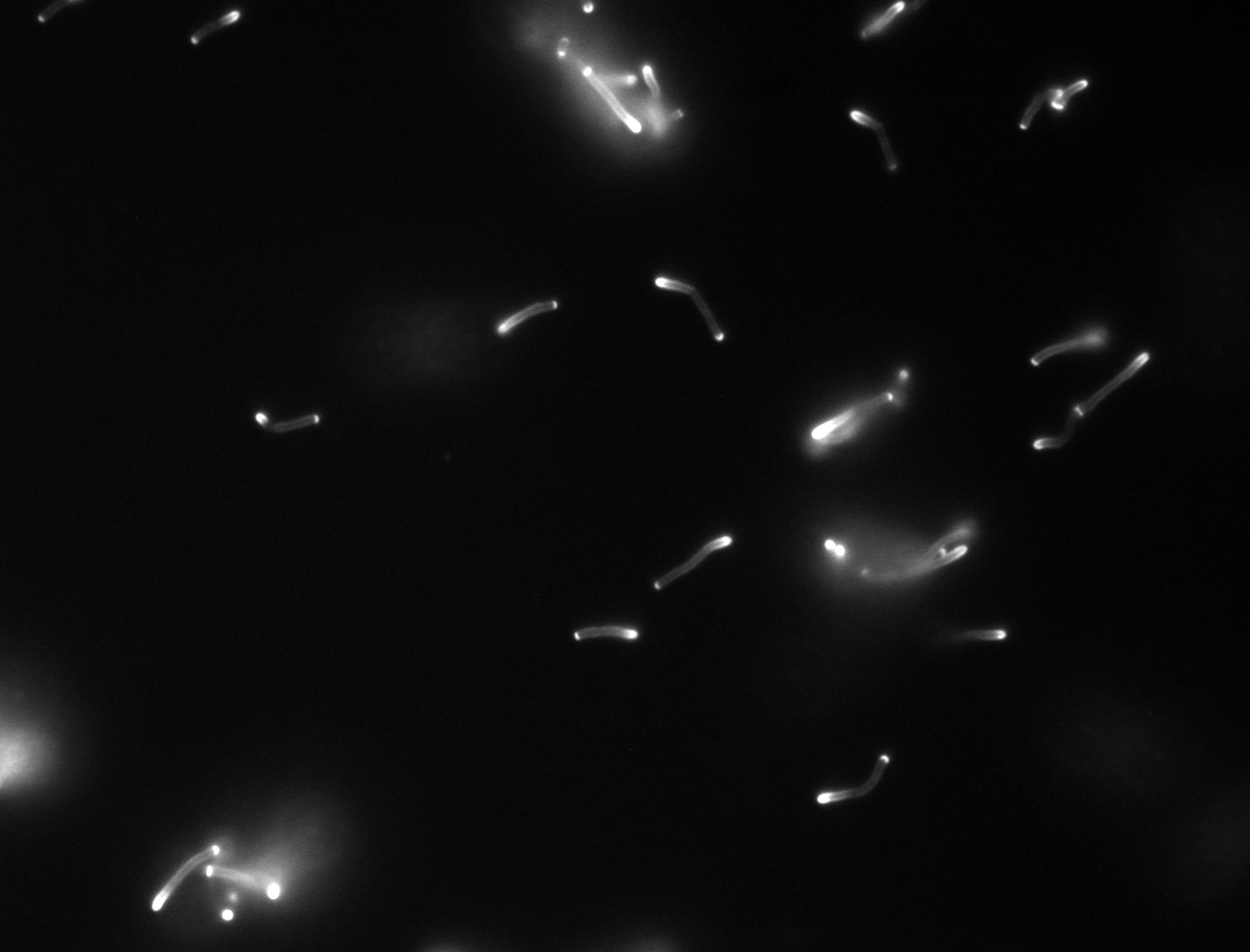

Supplement: Figure 2—source data 1. [file elife-37243-fig2-data1.zip › Figure 2 source data/Figure 2 source data-conventional microscopy (OalkTMM + RADA)/2. OAlkTMM/1.tif]

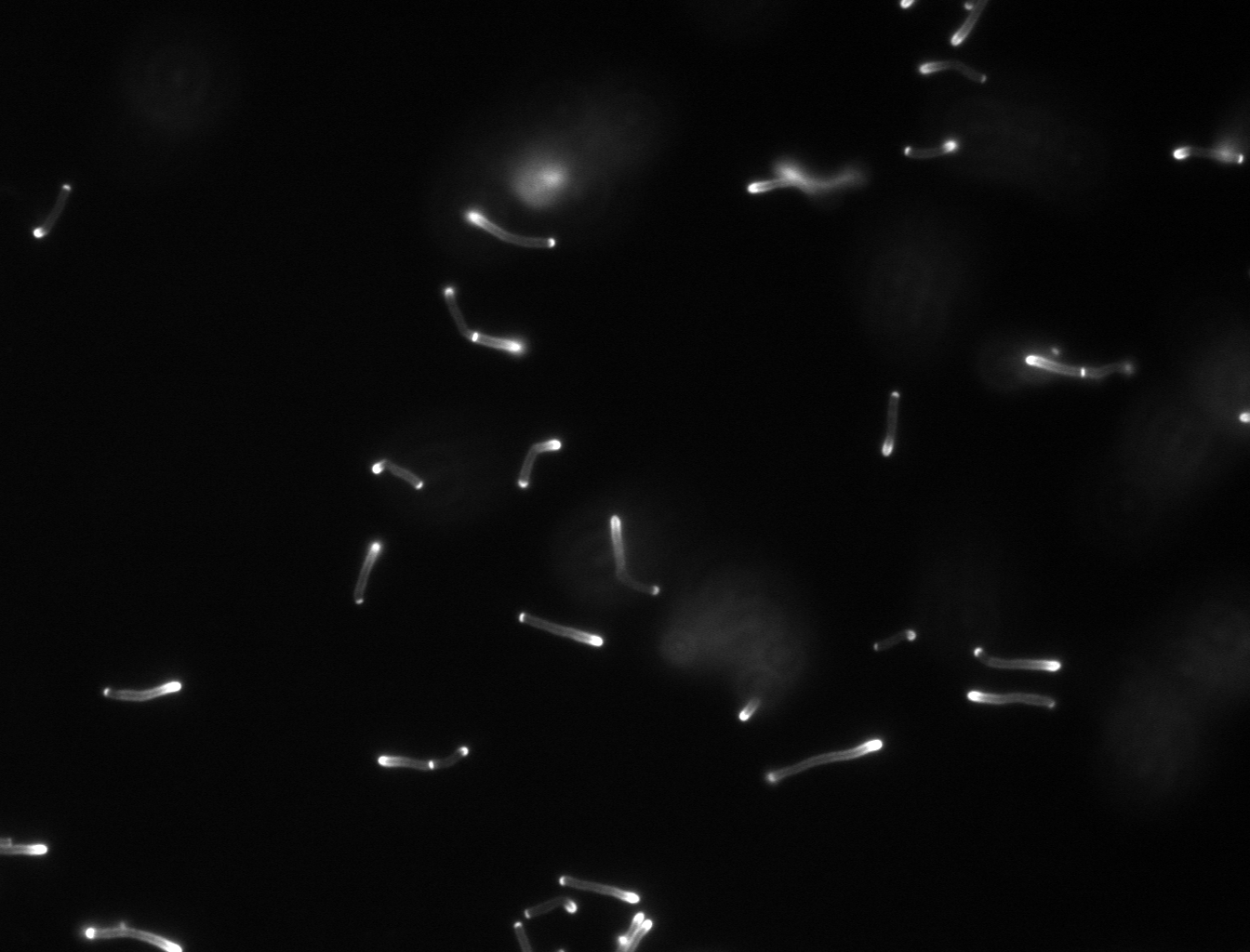

Supplement: Figure 2—source data 1. [file elife-37243-fig2-data1.zip › Figure 2 source data/Figure 2 source data-conventional microscopy (OalkTMM + RADA)/2. OAlkTMM/10.tif]

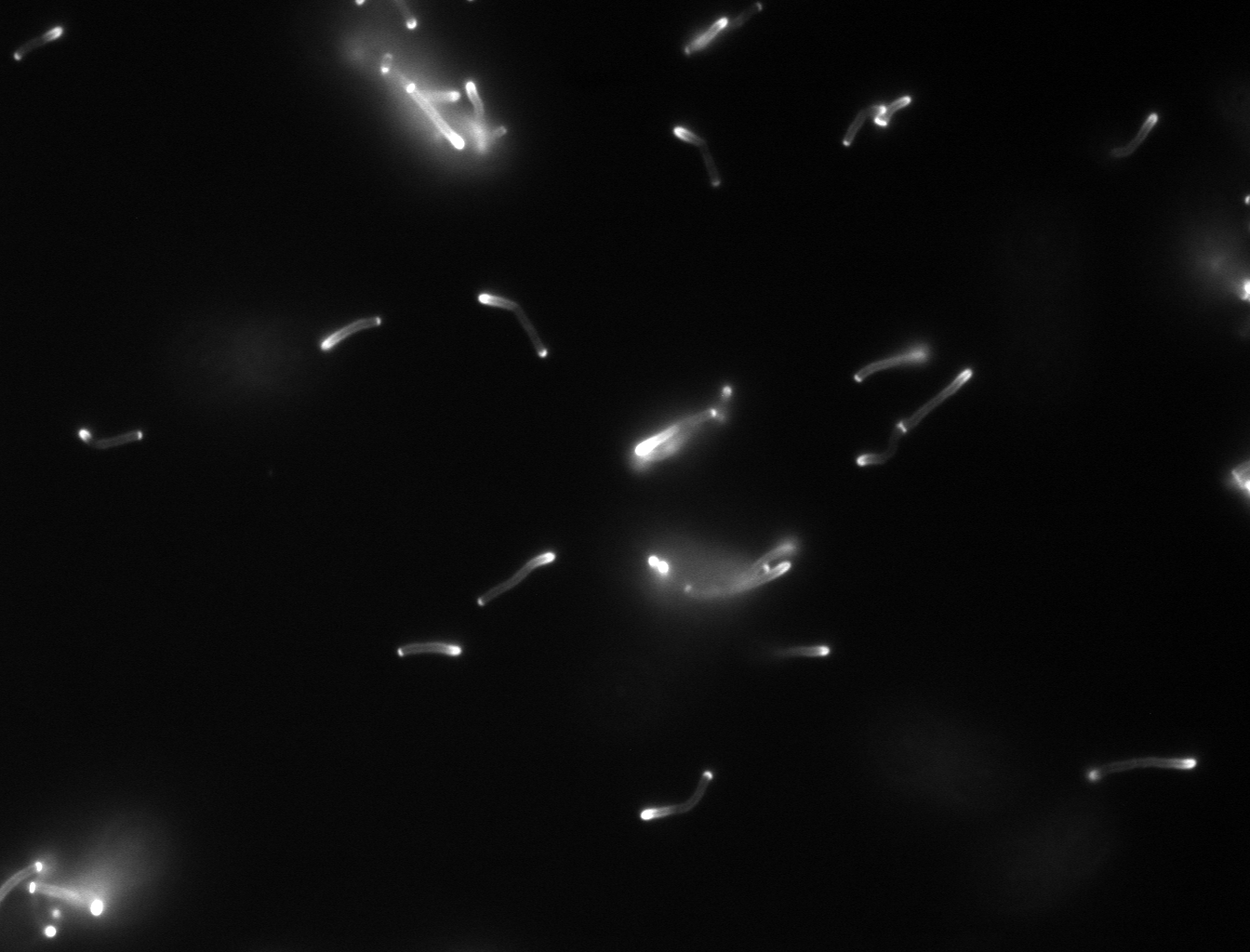

Supplement: Figure 2—source data 1. [file elife-37243-fig2-data1.zip › Figure 2 source data/Figure 2 source data-conventional microscopy (OalkTMM + RADA)/2. OAlkTMM/2.tif]

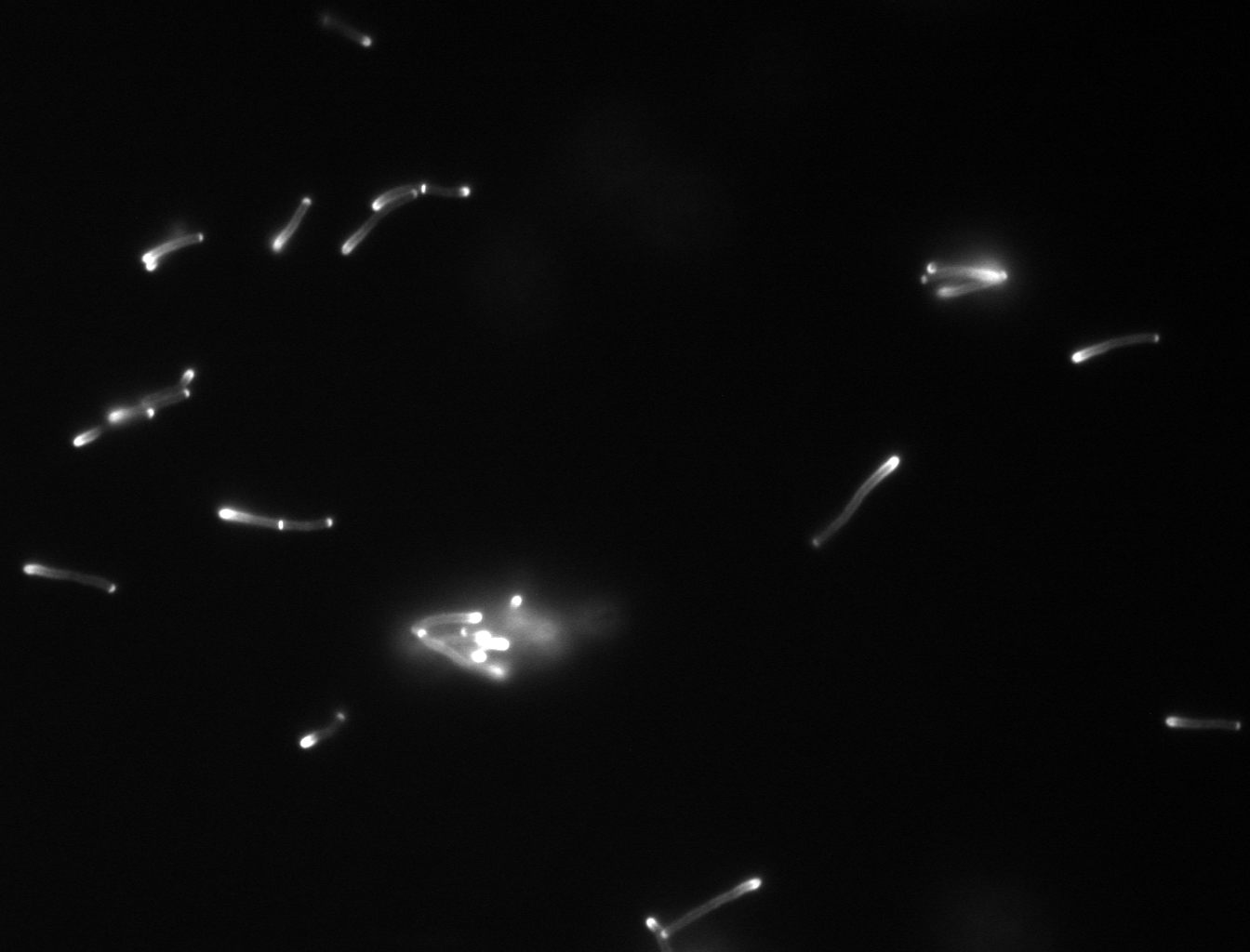

Supplement: Figure 2—source data 1. [file elife-37243-fig2-data1.zip › Figure 2 source data/Figure 2 source data-conventional microscopy (OalkTMM + RADA)/2. OAlkTMM/3.tif]

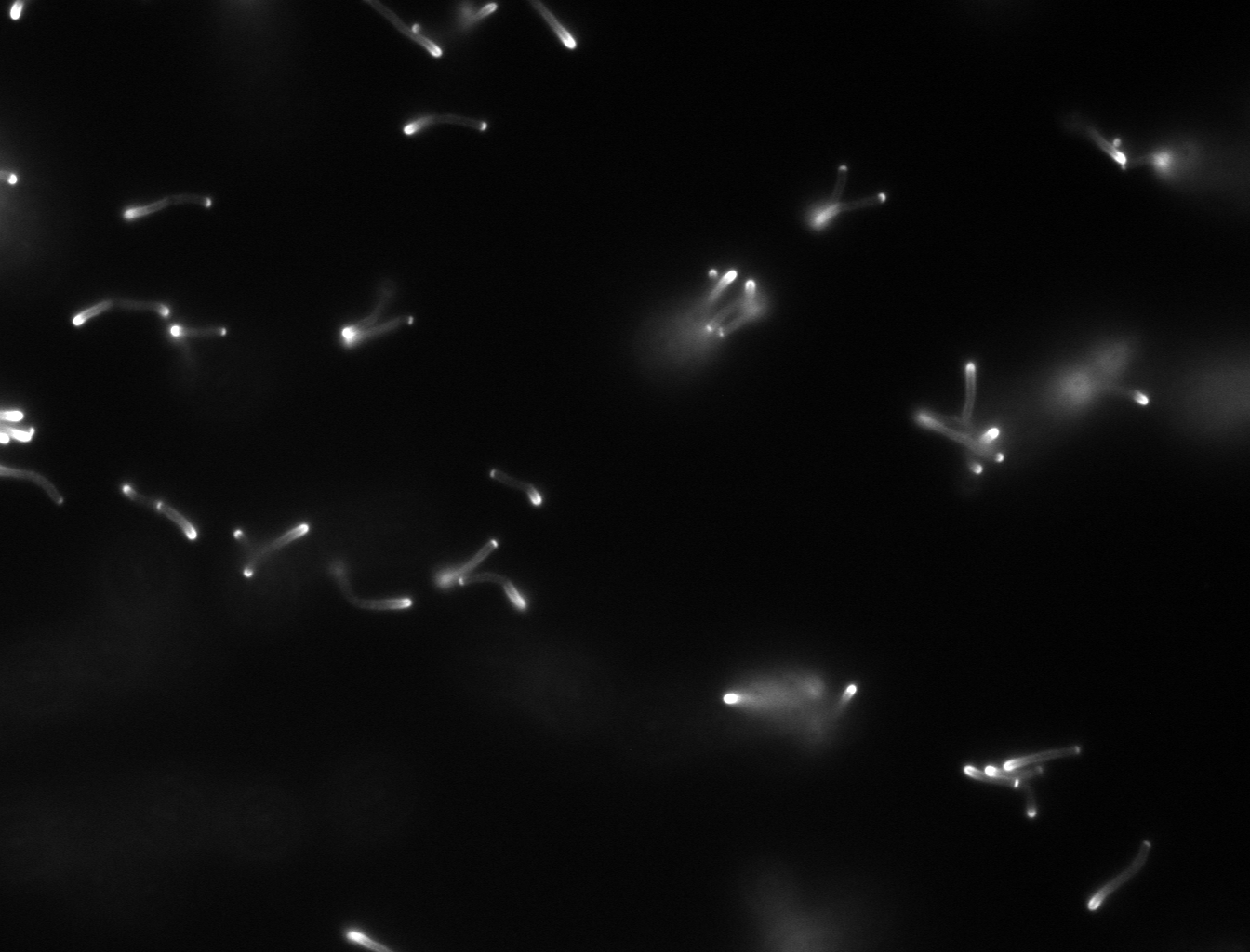

Supplement: Figure 2—source data 1. [file elife-37243-fig2-data1.zip › Figure 2 source data/Figure 2 source data-conventional microscopy (OalkTMM + RADA)/2. OAlkTMM/4.tif]

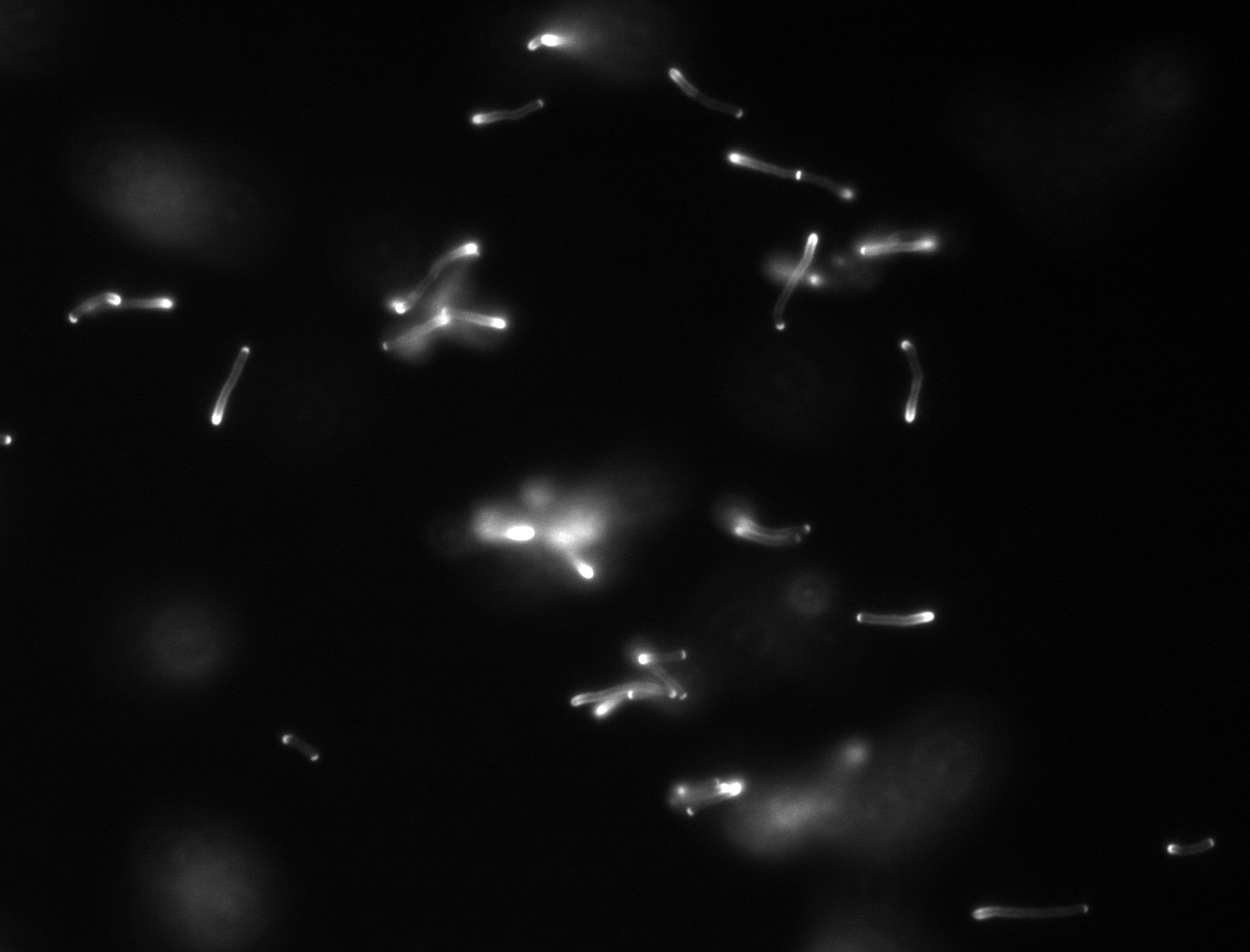

Supplement: Figure 2—source data 1. [file elife-37243-fig2-data1.zip › Figure 2 source data/Figure 2 source data-conventional microscopy (OalkTMM + RADA)/2. OAlkTMM/5.tif]

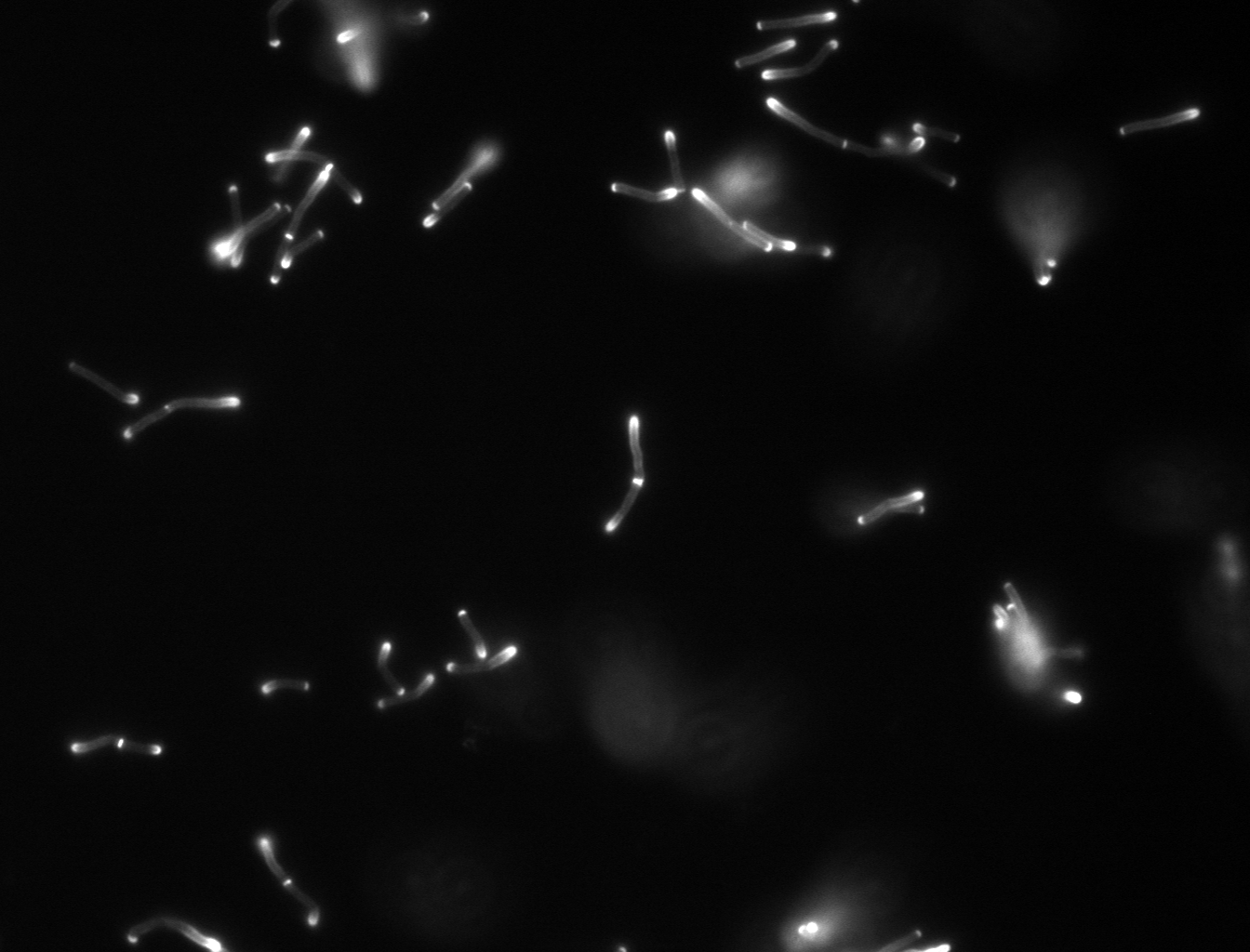

Supplement: Figure 2—source data 1. [file elife-37243-fig2-data1.zip › Figure 2 source data/Figure 2 source data-conventional microscopy (OalkTMM + RADA)/2. OAlkTMM/6.tif]

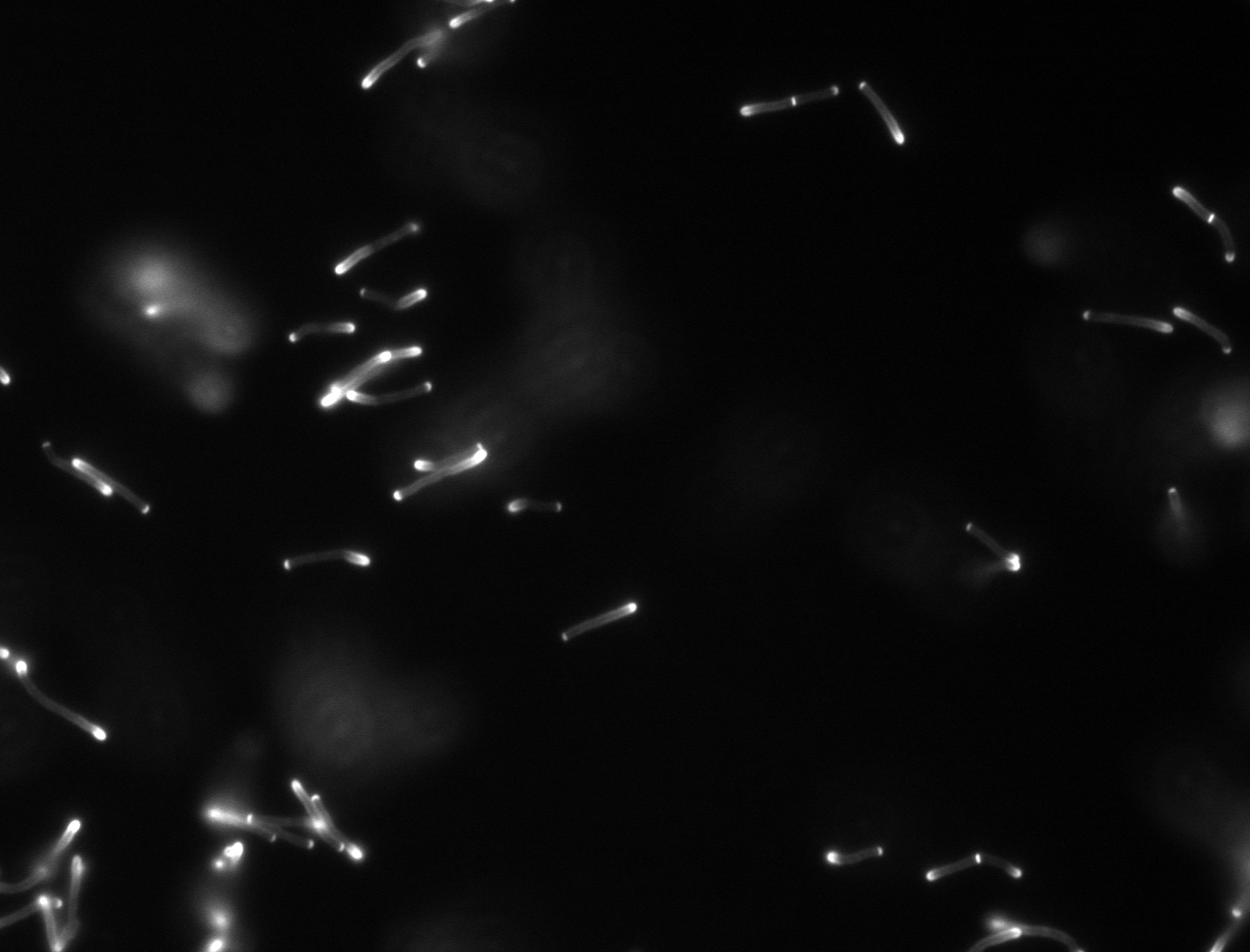

Supplement: Figure 2—source data 1. [file elife-37243-fig2-data1.zip › Figure 2 source data/Figure 2 source data-conventional microscopy (OalkTMM + RADA)/2. OAlkTMM/7.tif]

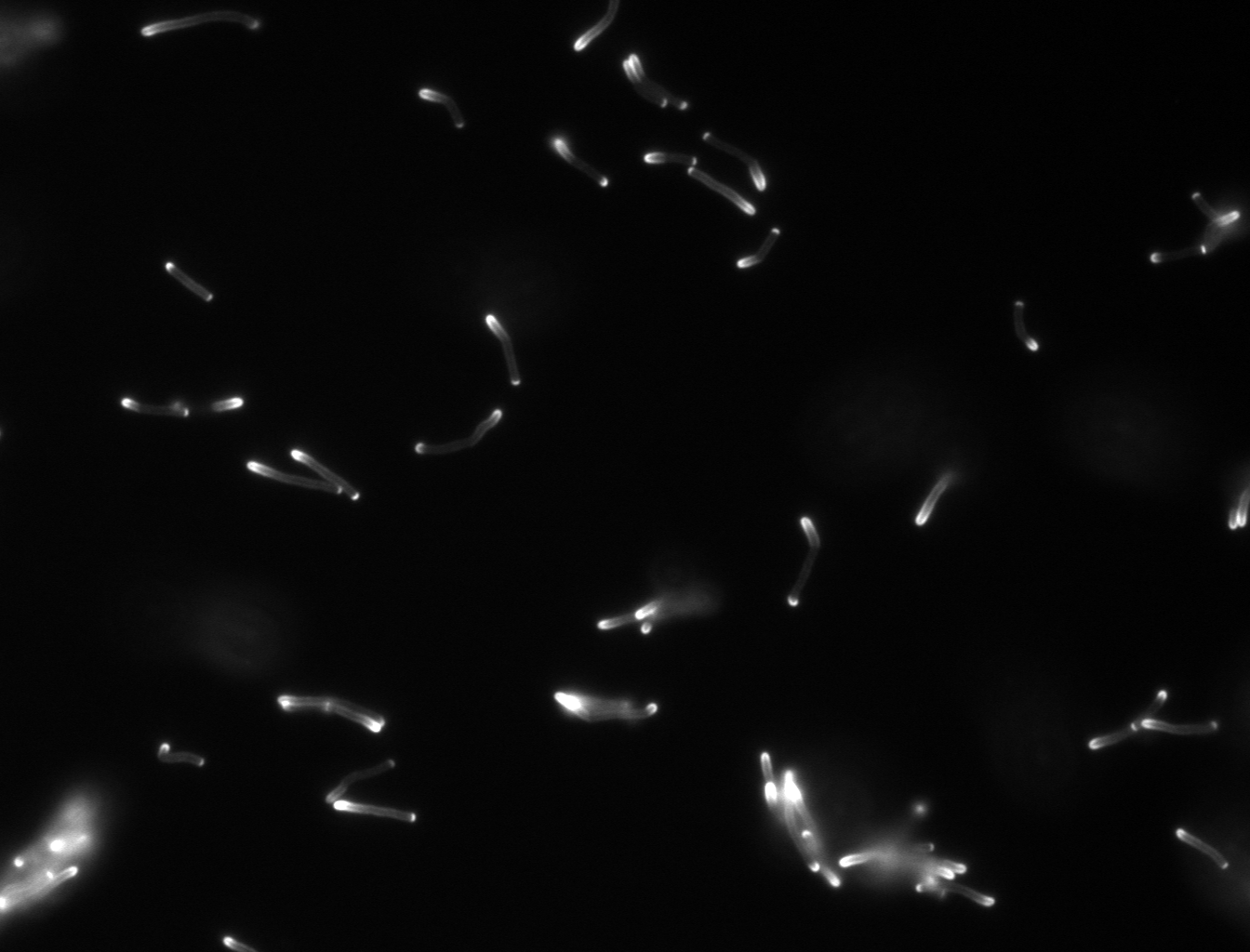

Supplement: Figure 2—source data 1. [file elife-37243-fig2-data1.zip › Figure 2 source data/Figure 2 source data-conventional microscopy (OalkTMM + RADA)/2. OAlkTMM/8.tif]

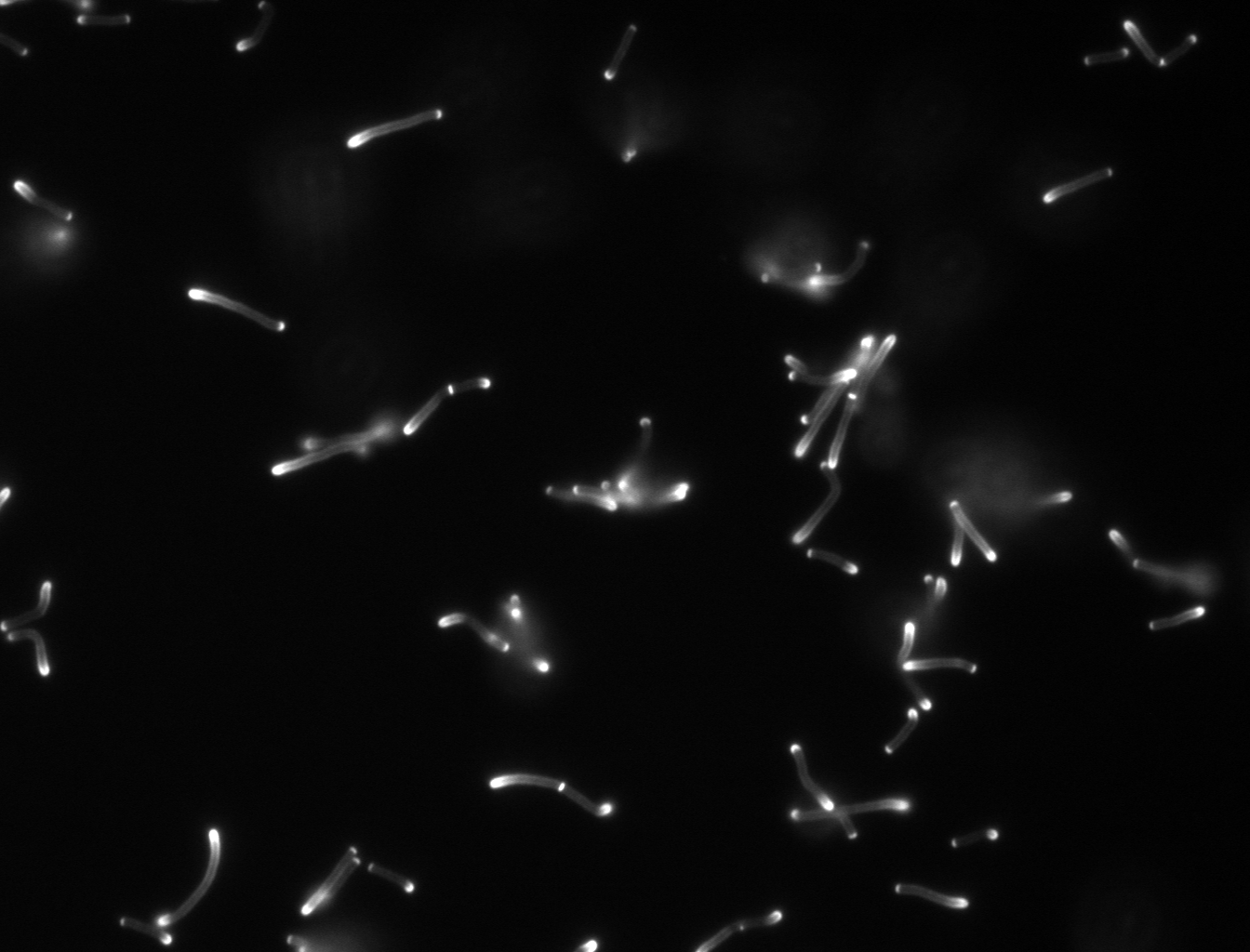

Supplement: Figure 2—source data 1. [file elife-37243-fig2-data1.zip › Figure 2 source data/Figure 2 source data-conventional microscopy (OalkTMM + RADA)/2. OAlkTMM/9.tif]

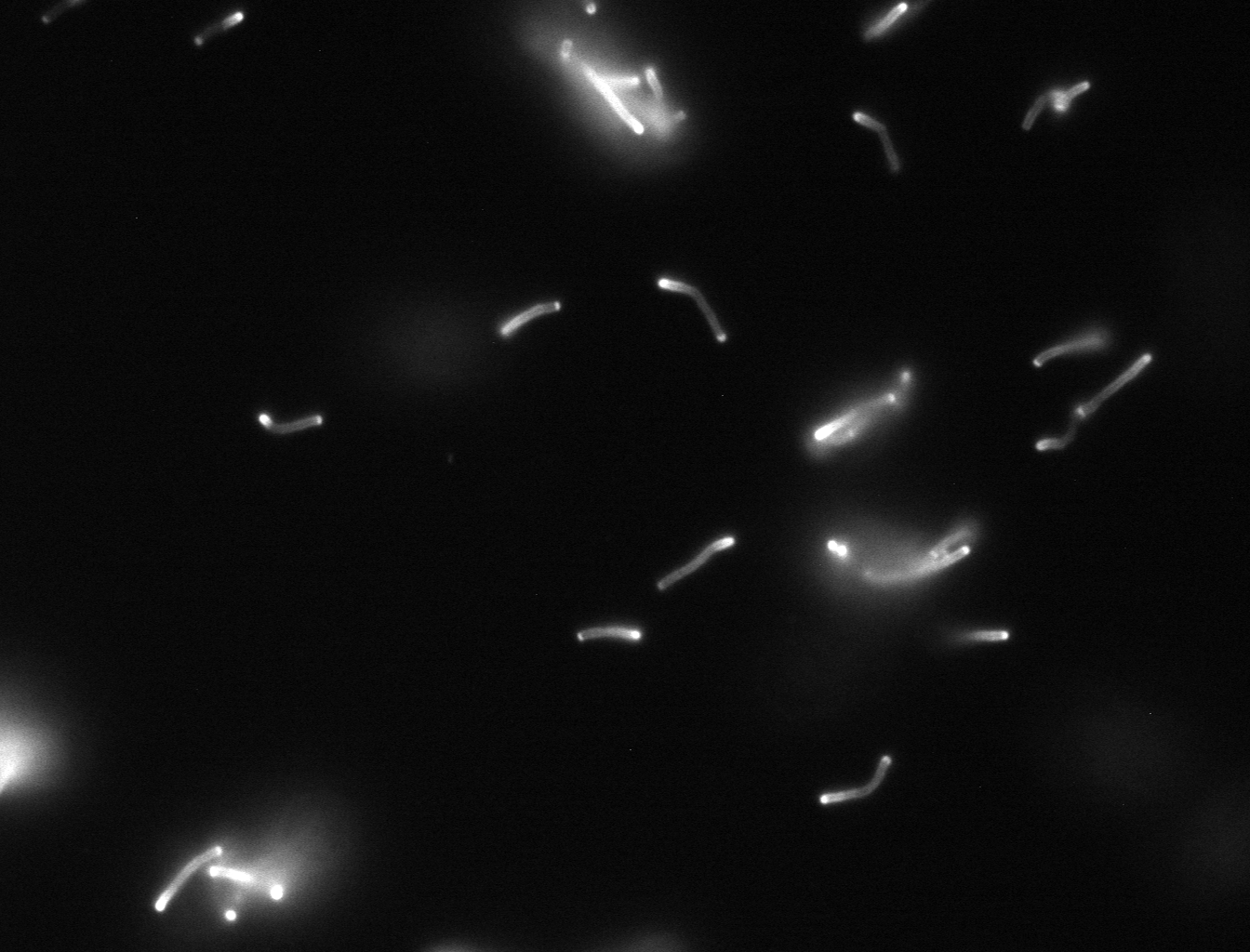

Supplement: Figure 2—source data 1. [file elife-37243-fig2-data1.zip › Figure 2 source data/Figure 2 source data-conventional microscopy (OalkTMM + RADA)/3. RADA/1.tif]

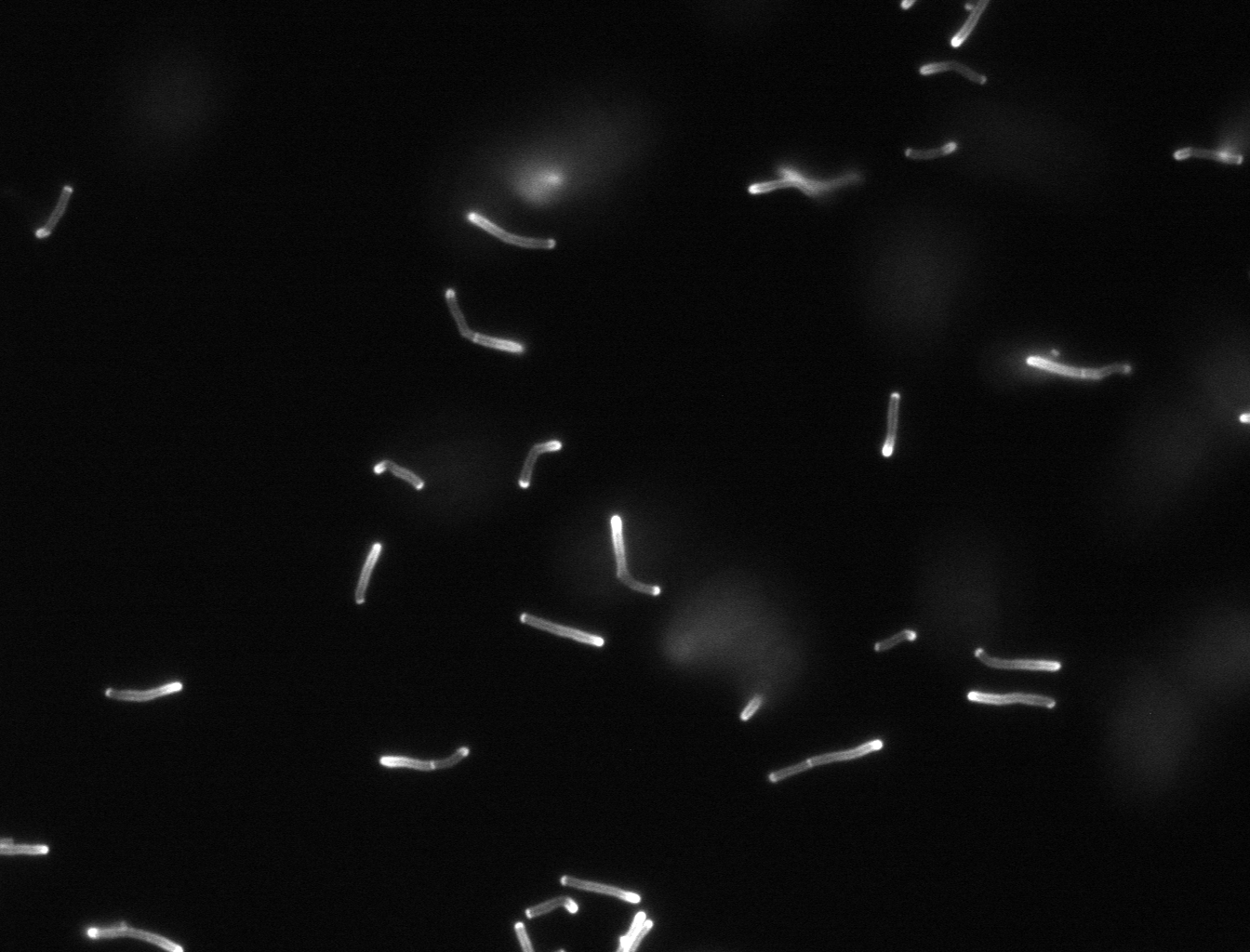

Supplement: Figure 2—source data 1. [file elife-37243-fig2-data1.zip › Figure 2 source data/Figure 2 source data-conventional microscopy (OalkTMM + RADA)/3. RADA/10.tif]

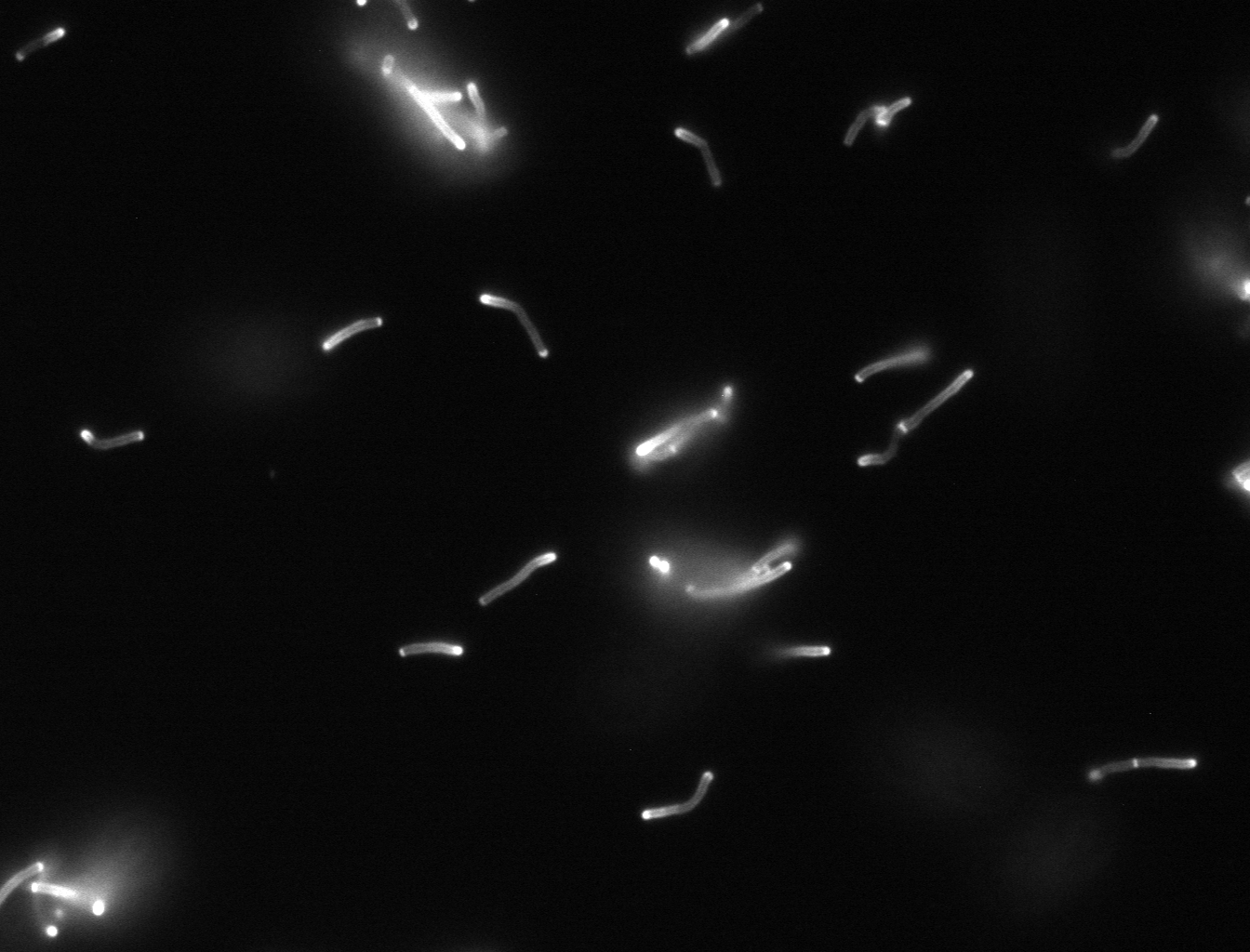

Supplement: Figure 2—source data 1. [file elife-37243-fig2-data1.zip › Figure 2 source data/Figure 2 source data-conventional microscopy (OalkTMM + RADA)/3. RADA/2.tif]

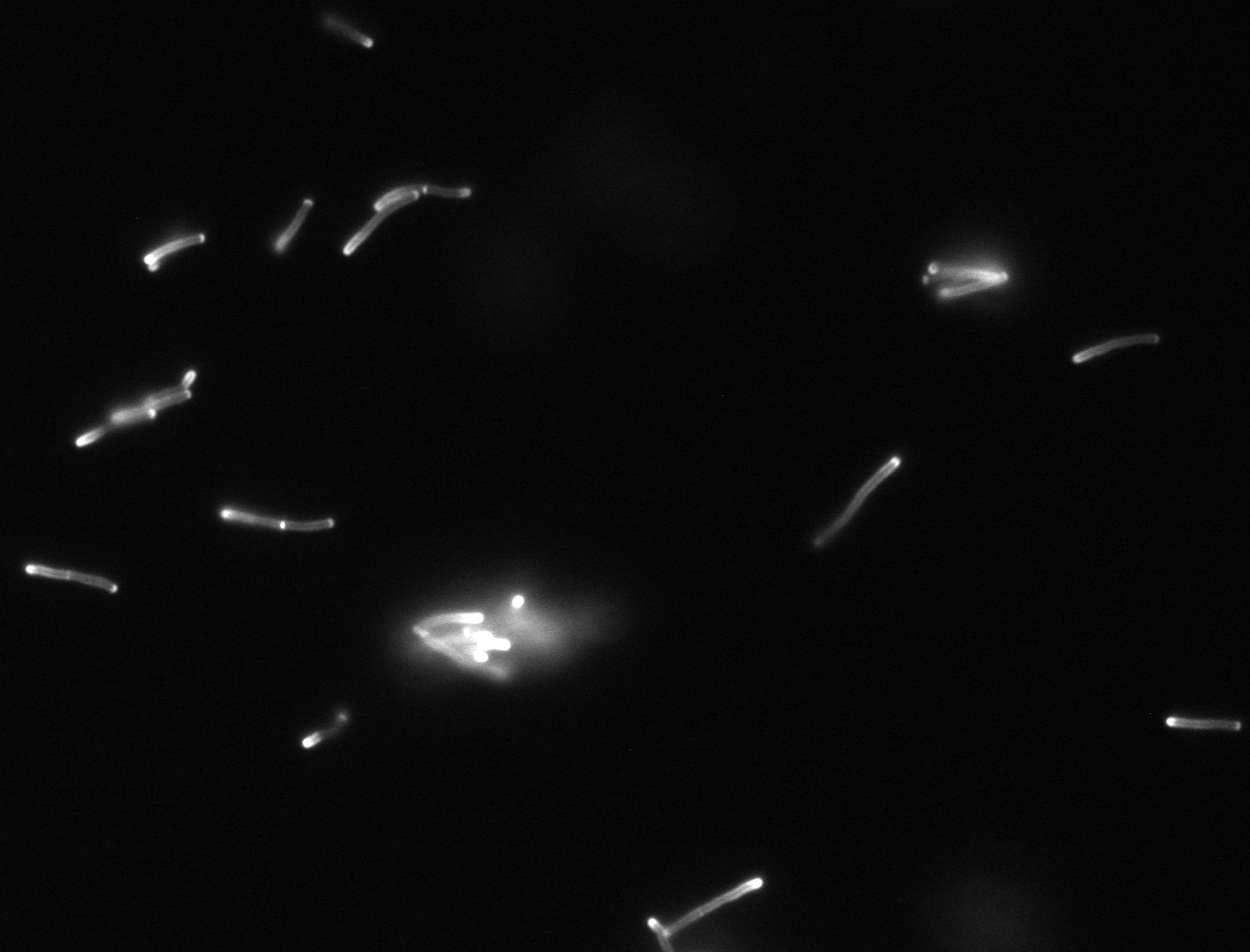

Supplement: Figure 2—source data 1. [file elife-37243-fig2-data1.zip › Figure 2 source data/Figure 2 source data-conventional microscopy (OalkTMM + RADA)/3. RADA/3.tif]

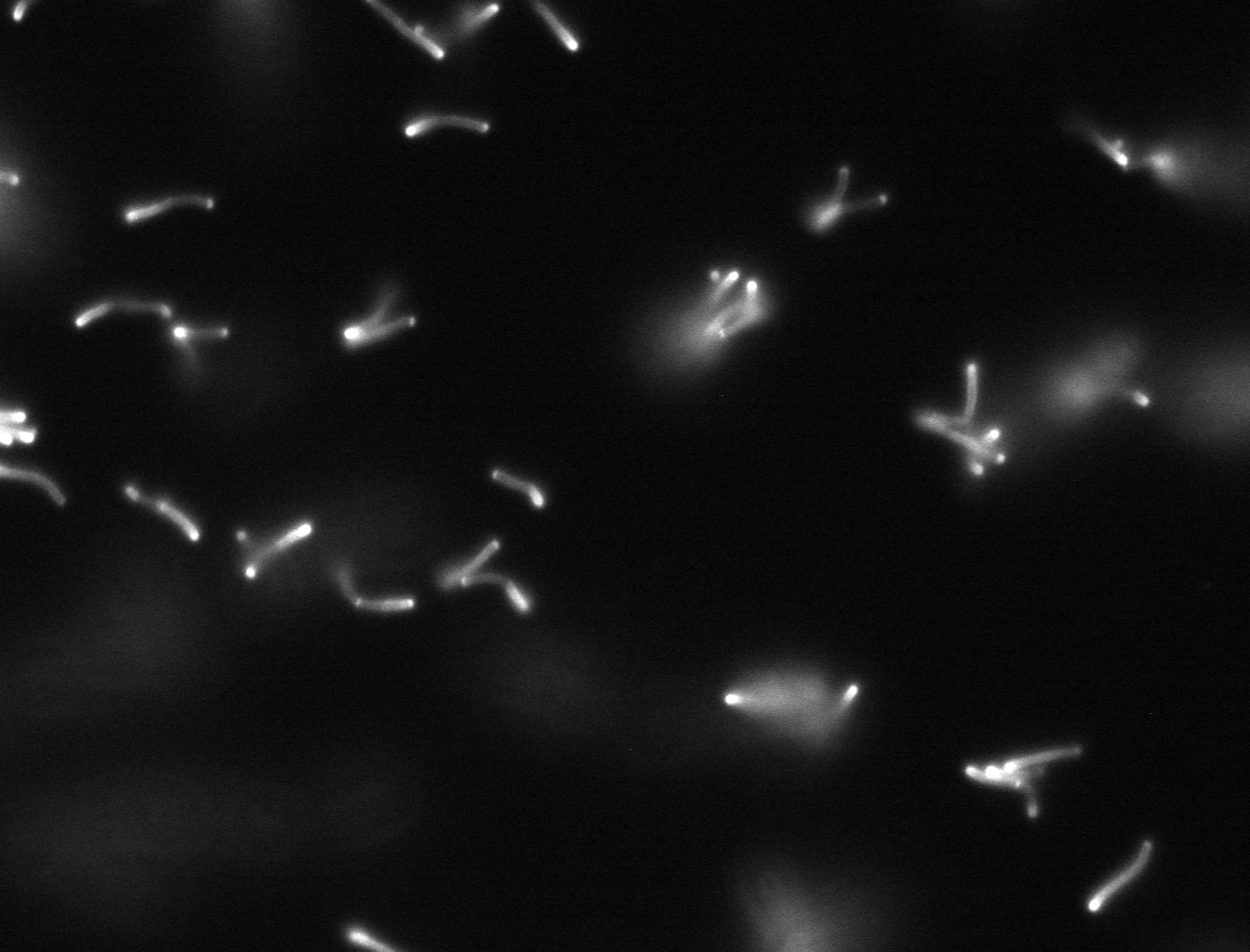

Supplement: Figure 2—source data 1. [file elife-37243-fig2-data1.zip › Figure 2 source data/Figure 2 source data-conventional microscopy (OalkTMM + RADA)/3. RADA/4.tif]

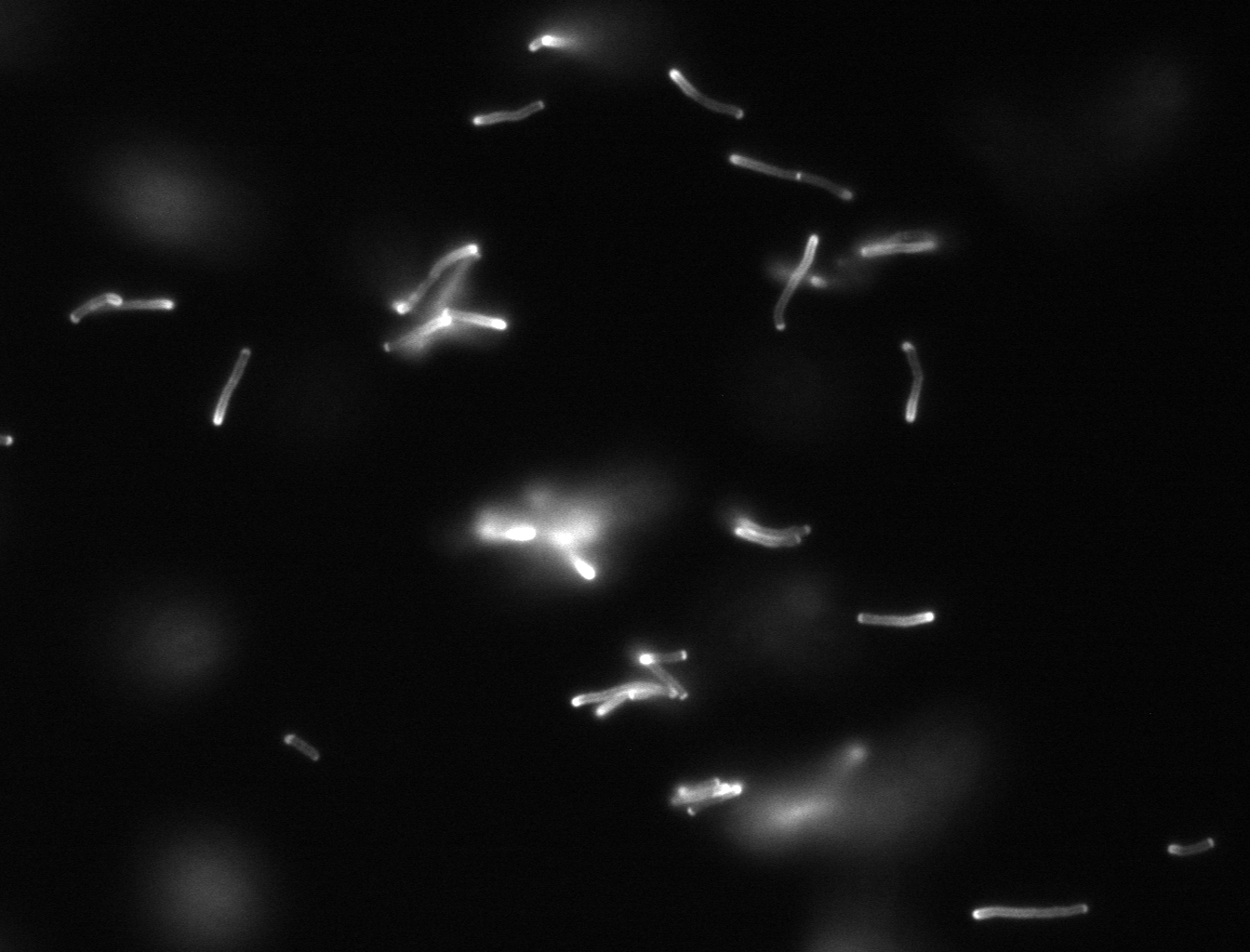

Supplement: Figure 2—source data 1. [file elife-37243-fig2-data1.zip › Figure 2 source data/Figure 2 source data-conventional microscopy (OalkTMM + RADA)/3. RADA/5.tif]

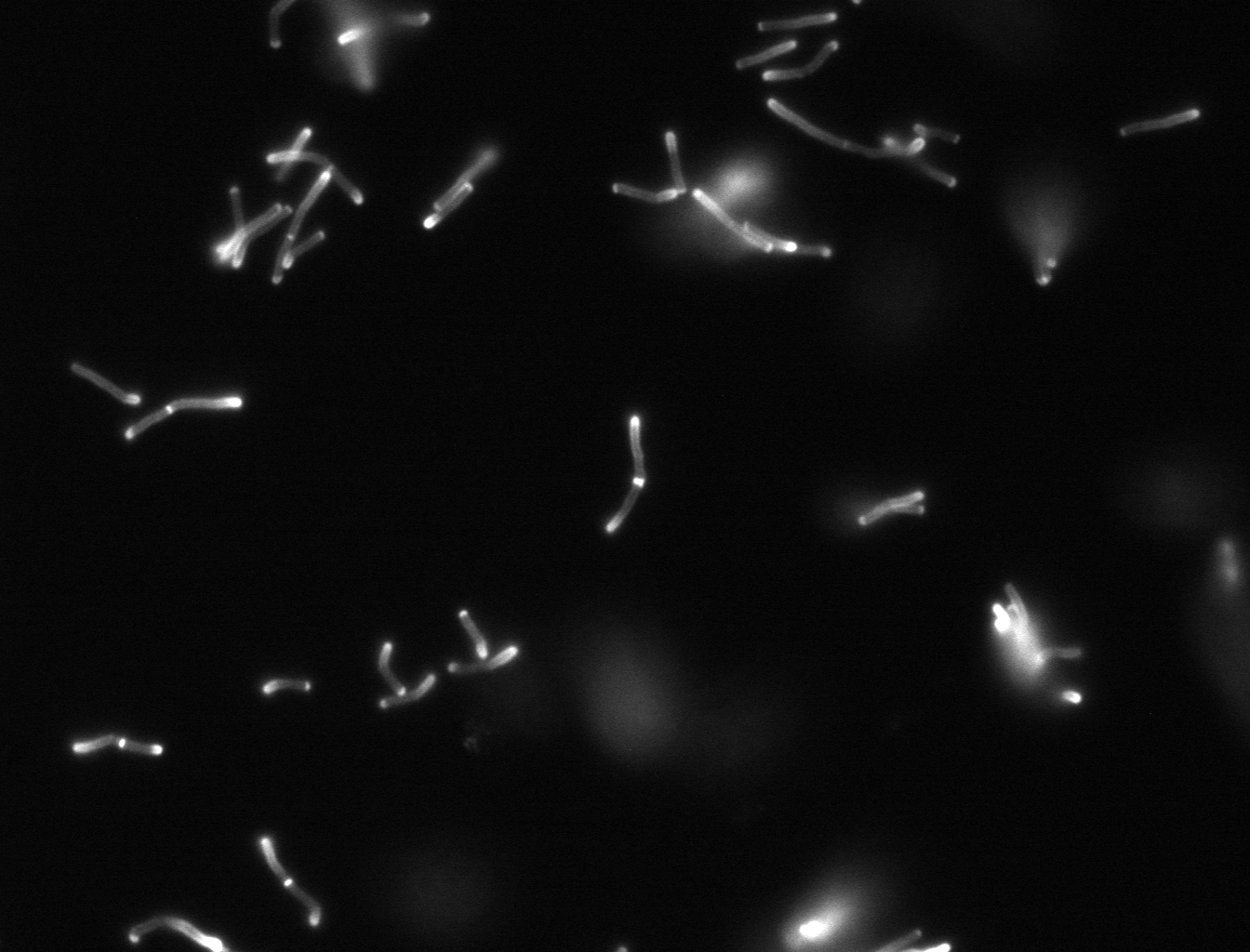

Supplement: Figure 2—source data 1. [file elife-37243-fig2-data1.zip › Figure 2 source data/Figure 2 source data-conventional microscopy (OalkTMM + RADA)/3. RADA/6.tif]

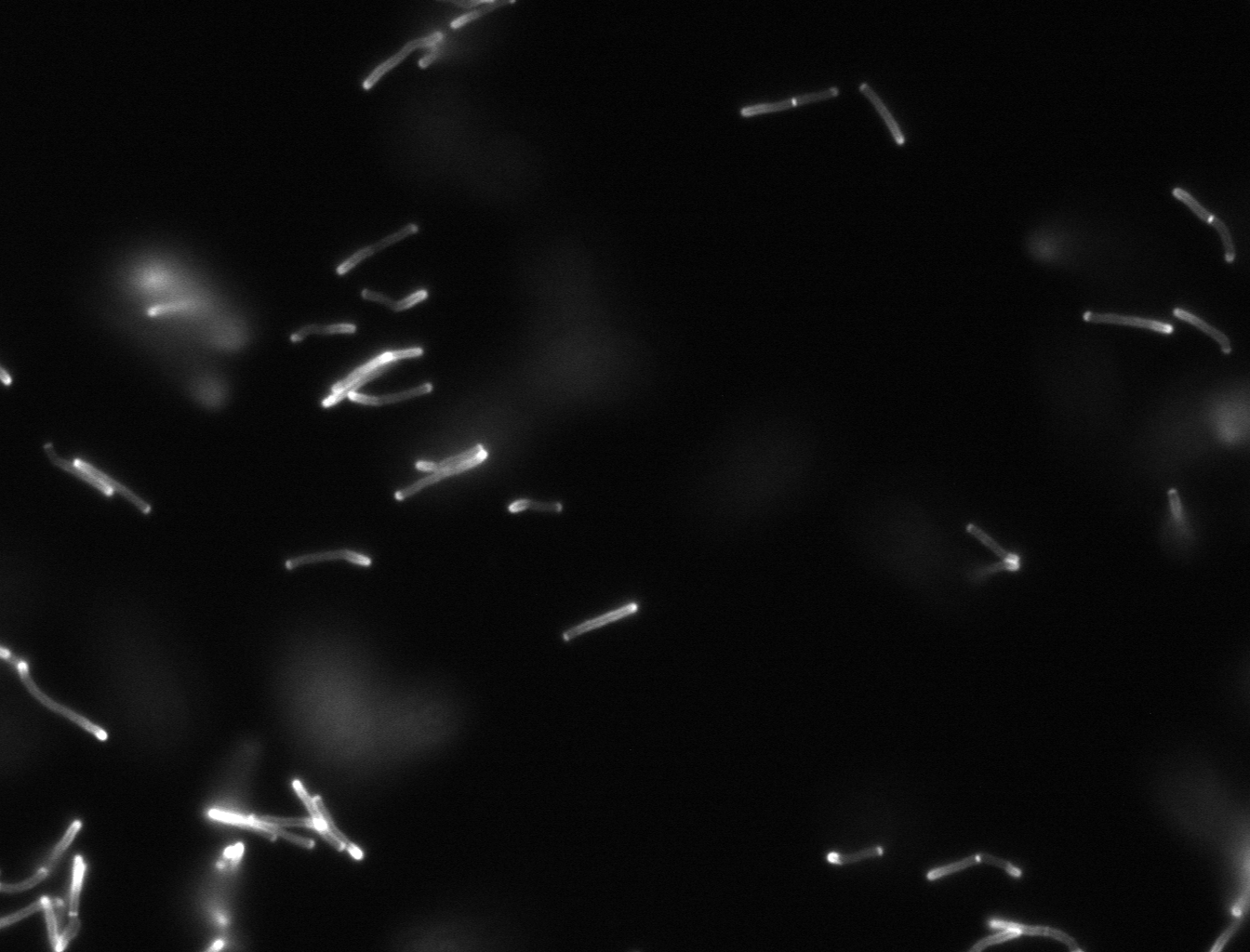

Supplement: Figure 2—source data 1. [file elife-37243-fig2-data1.zip › Figure 2 source data/Figure 2 source data-conventional microscopy (OalkTMM + RADA)/3. RADA/7.tif]

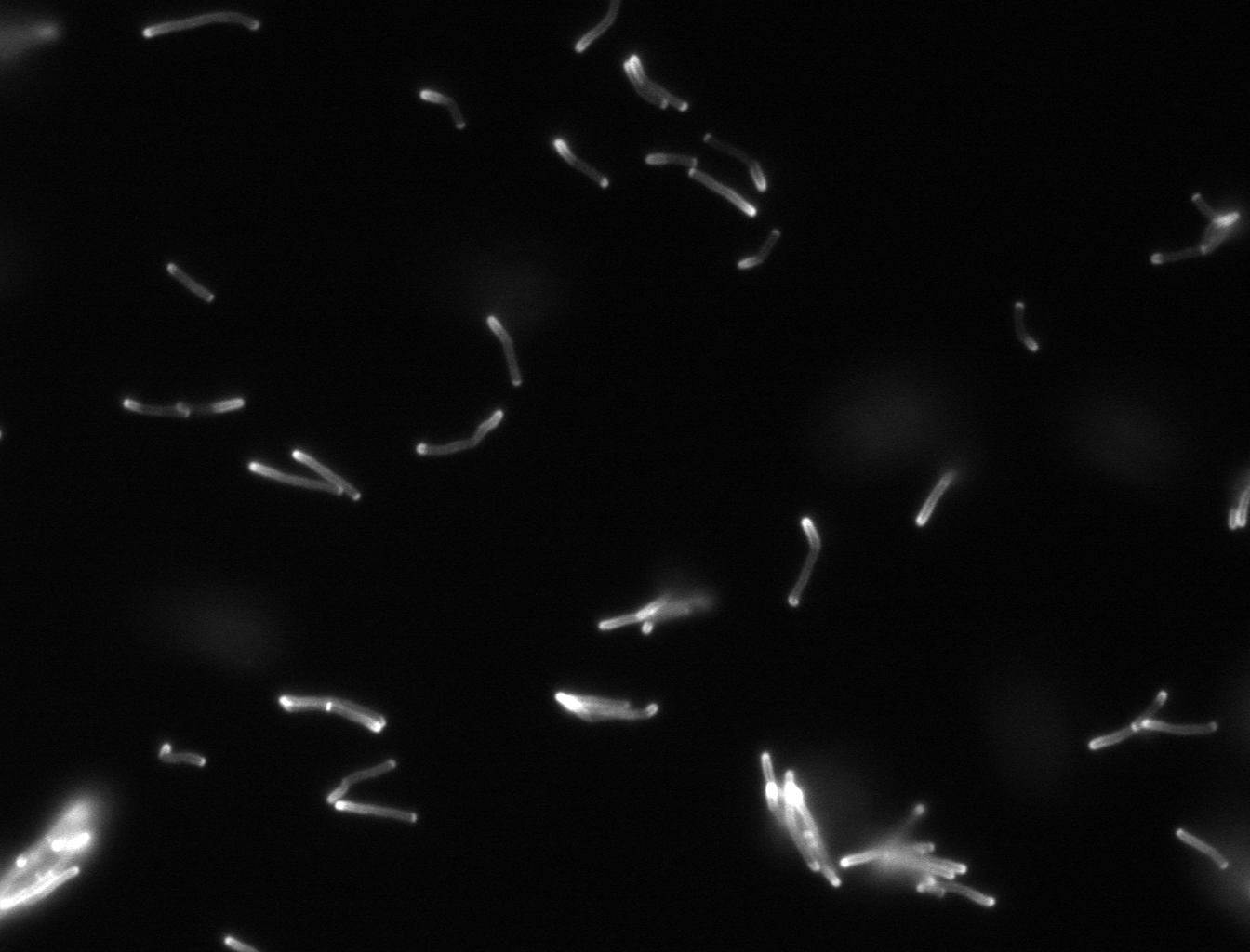

Supplement: Figure 2—source data 1. [file elife-37243-fig2-data1.zip › Figure 2 source data/Figure 2 source data-conventional microscopy (OalkTMM + RADA)/3. RADA/8.tif]

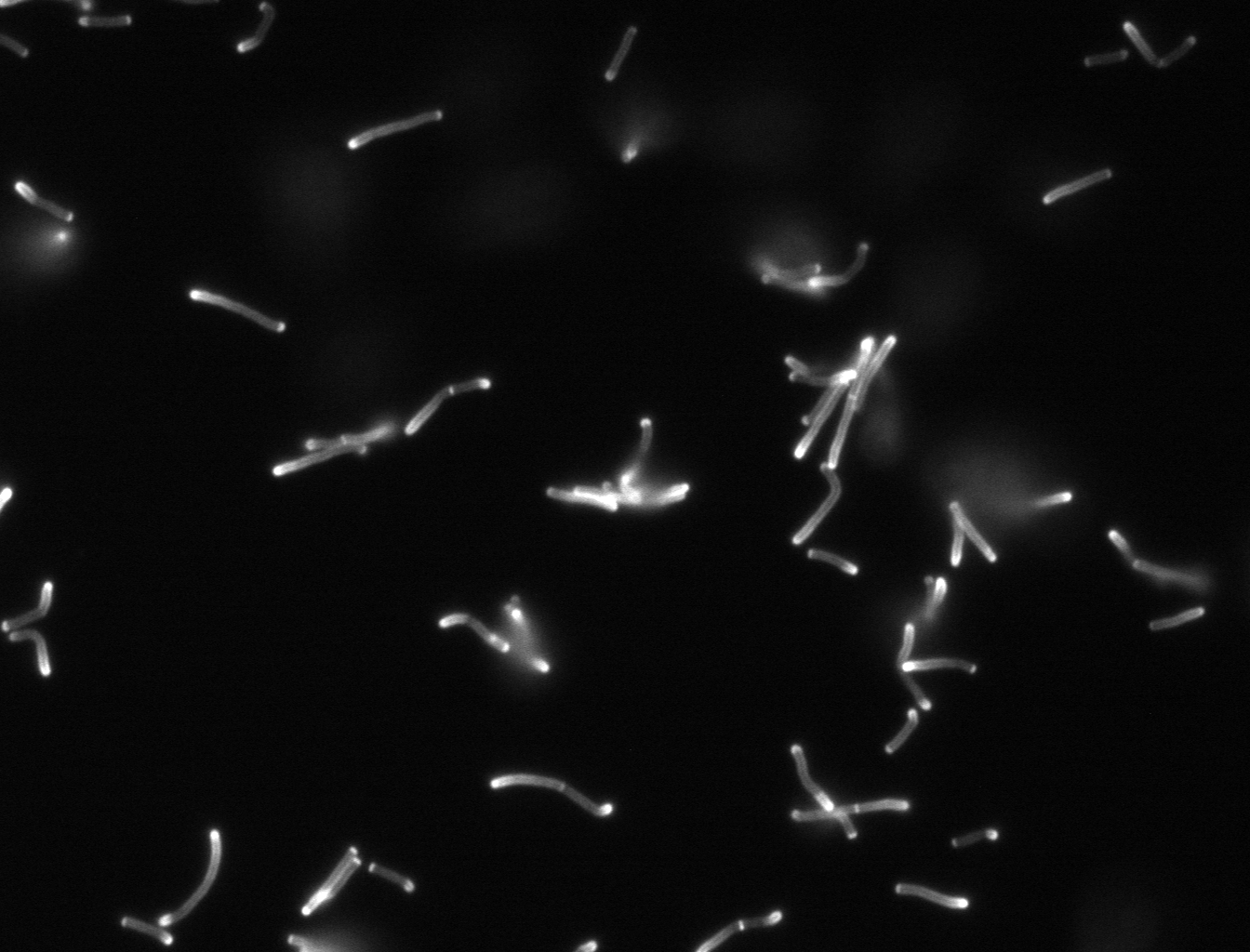

Supplement: Figure 2—source data 1. [file elife-37243-fig2-data1.zip › Figure 2 source data/Figure 2 source data-conventional microscopy (OalkTMM + RADA)/3. RADA/9.tif]

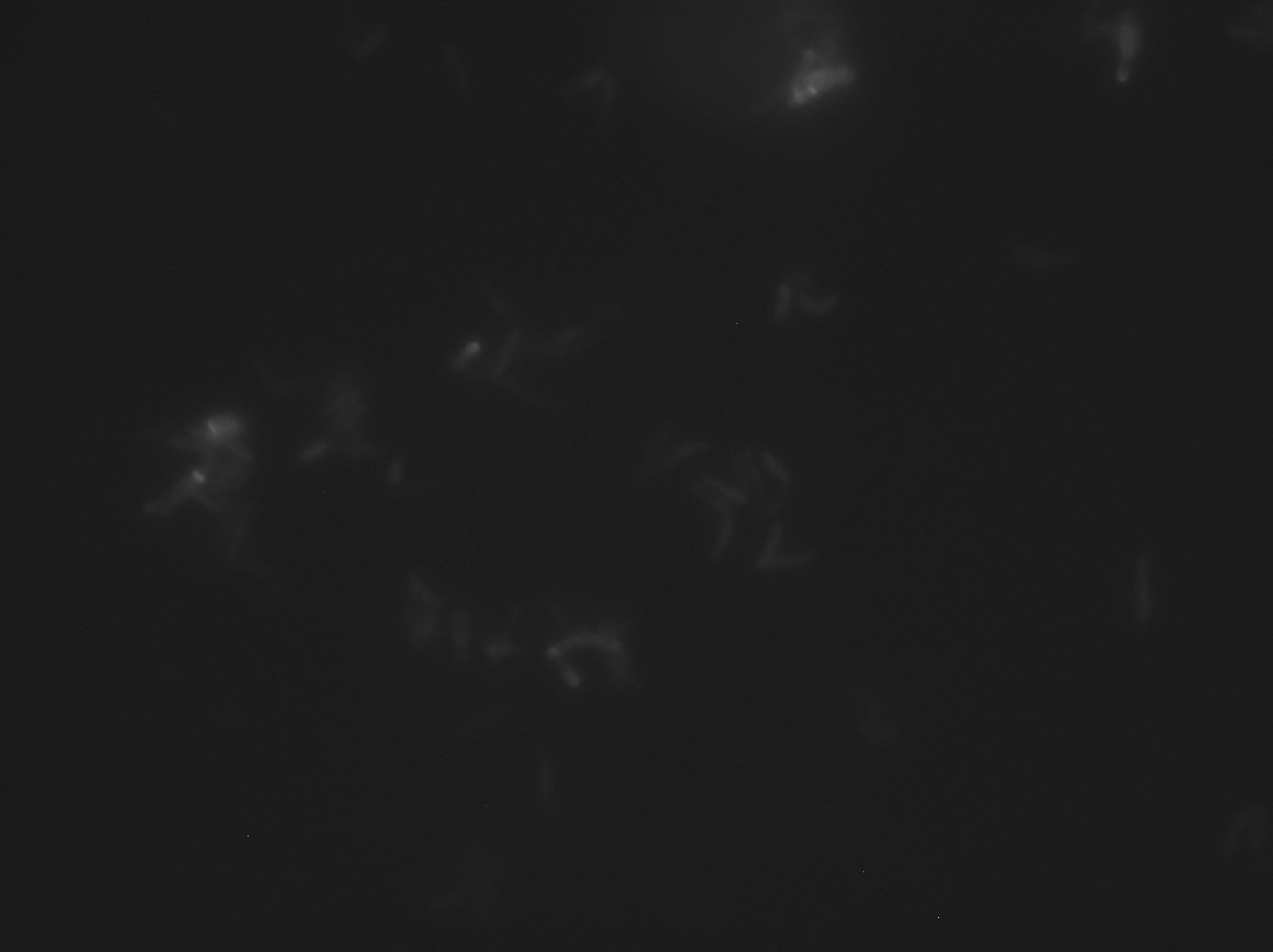

Supplement: Figure 2—figure supplement 2—source data 1. [file elife-37243-fig2-figsupp2-data1.zip › Figure 2--figure supplement 2/Figure 2--figure supplement 2C/alkDADA/CFP 4 sec 10.jpg]

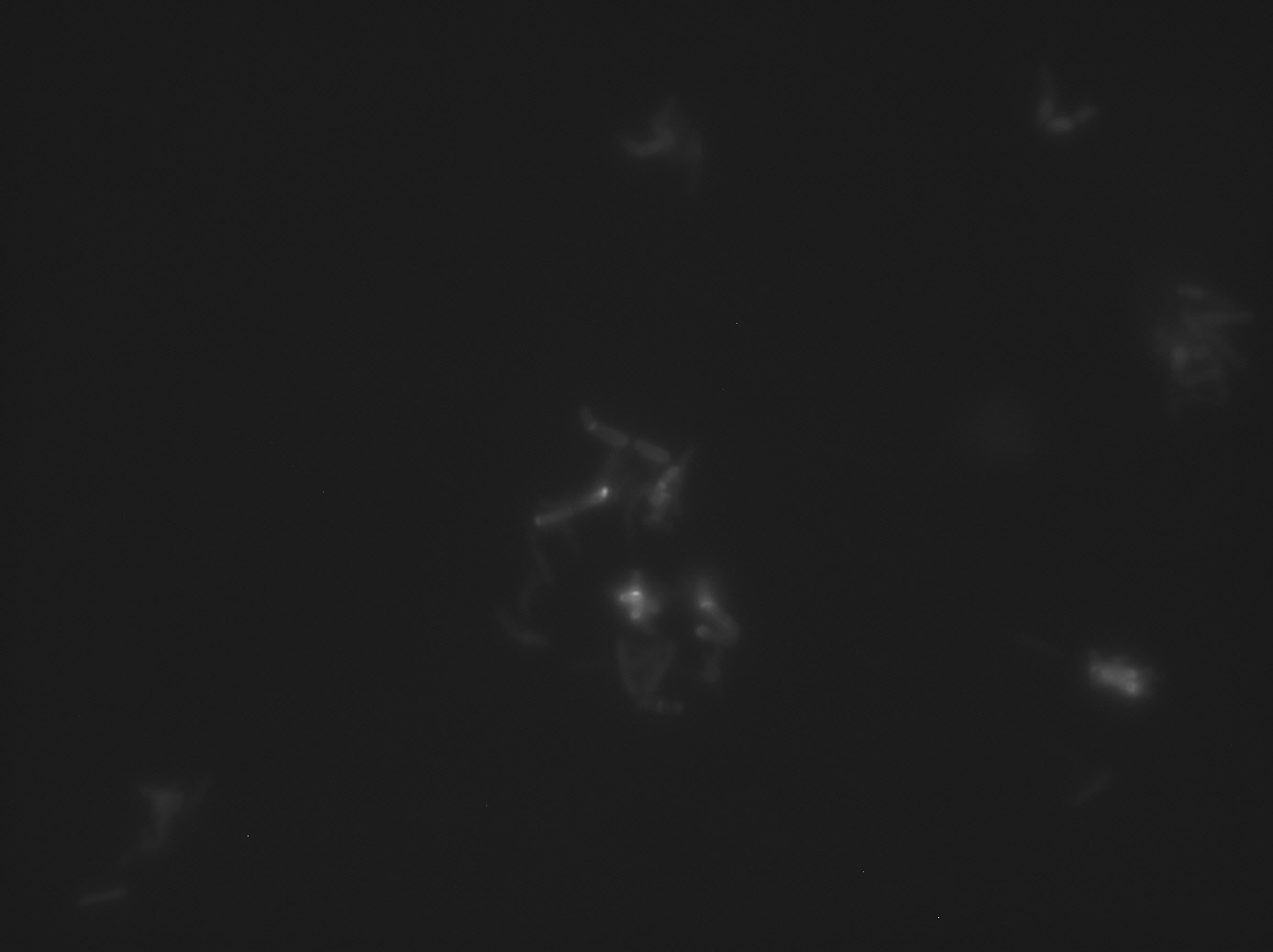

Supplement: Figure 2—figure supplement 2—source data 1. [file elife-37243-fig2-figsupp2-data1.zip › Figure 2--figure supplement 2/Figure 2--figure supplement 2C/alkDADA/CFP 4 sec 11.jpg]

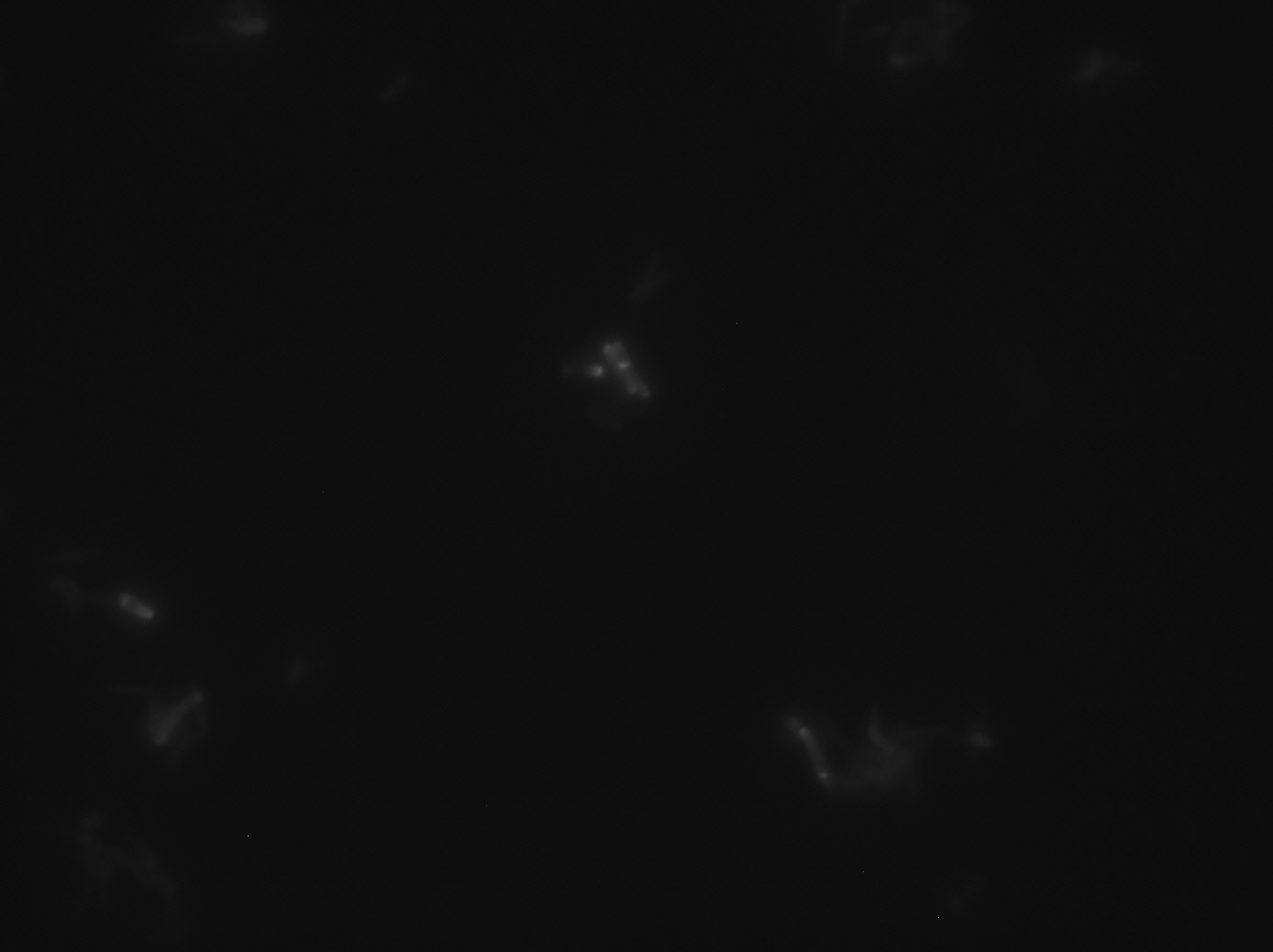

Supplement: Figure 2—figure supplement 2—source data 1. [file elife-37243-fig2-figsupp2-data1.zip › Figure 2--figure supplement 2/Figure 2--figure supplement 2C/alkDADA/CFP 4 sec 7.jpg]

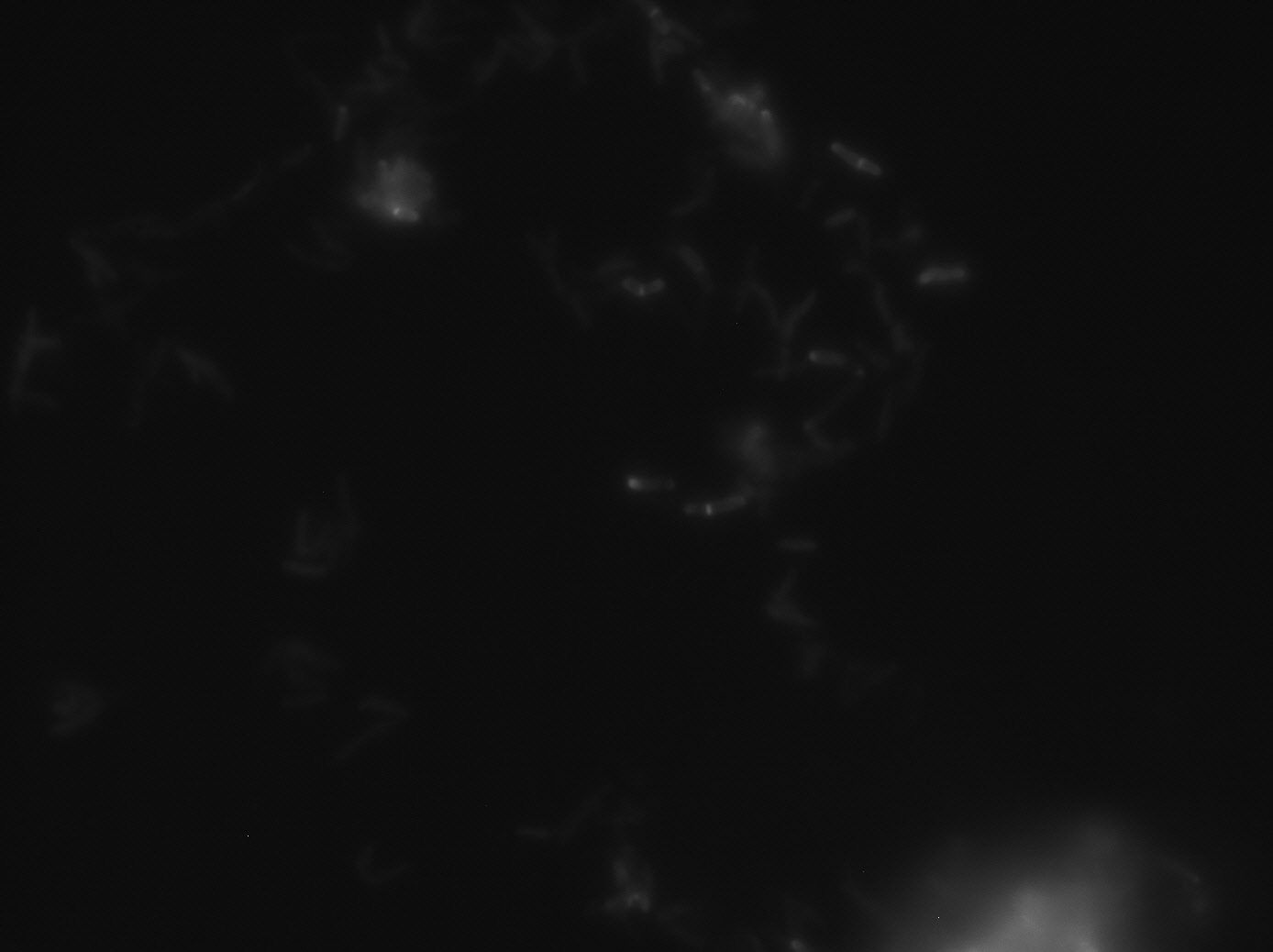

Supplement: Figure 2—figure supplement 2—source data 1. [file elife-37243-fig2-figsupp2-data1.zip › Figure 2--figure supplement 2/Figure 2--figure supplement 2C/alkDADA/CFP 4 sec 9.jpg]

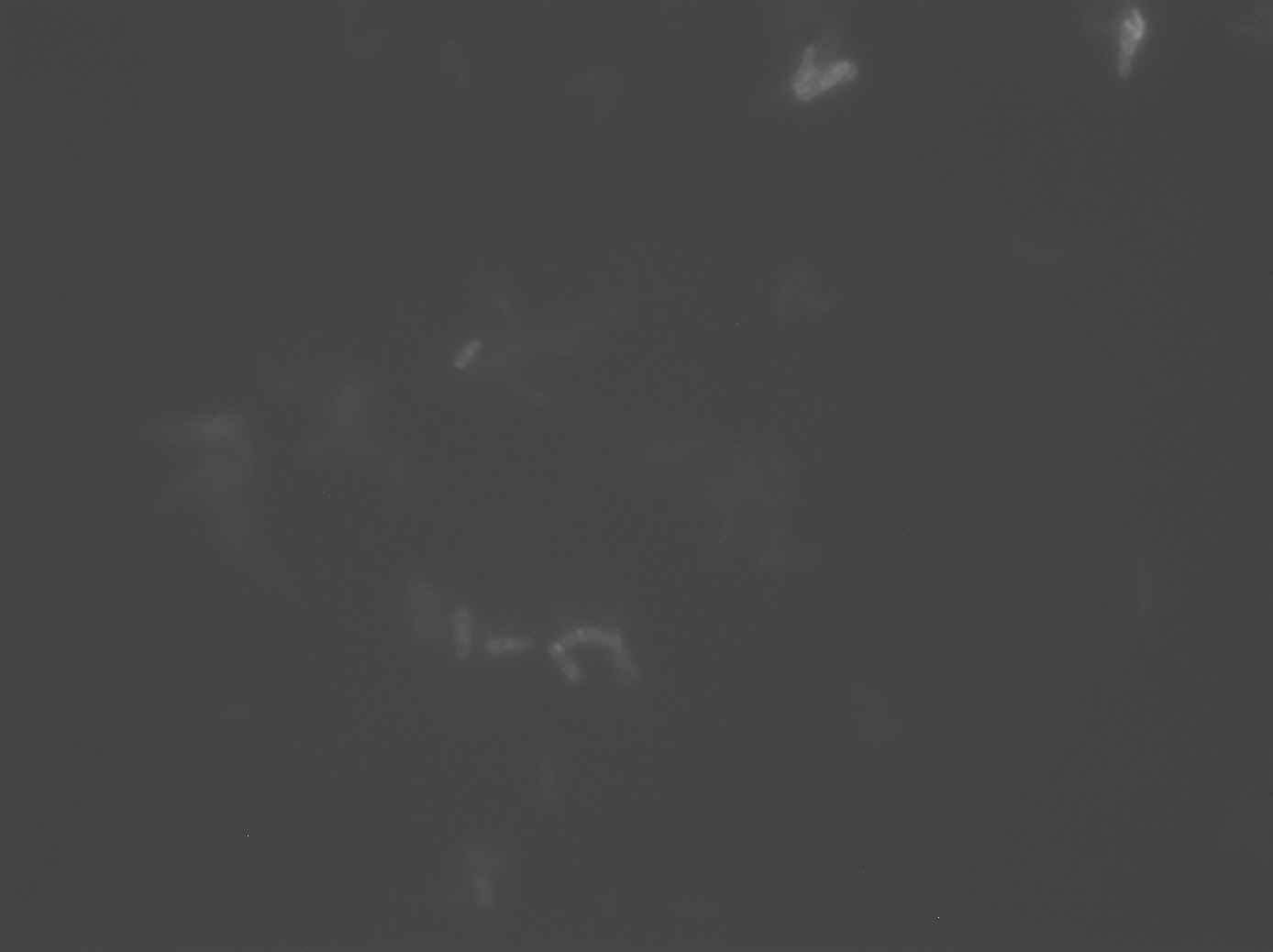

Supplement: Figure 2—figure supplement 2—source data 1. [file elife-37243-fig2-figsupp2-data1.zip › Figure 2--figure supplement 2/Figure 2--figure supplement 2C/alkDADA/GFP 2 sec 10.jpg]

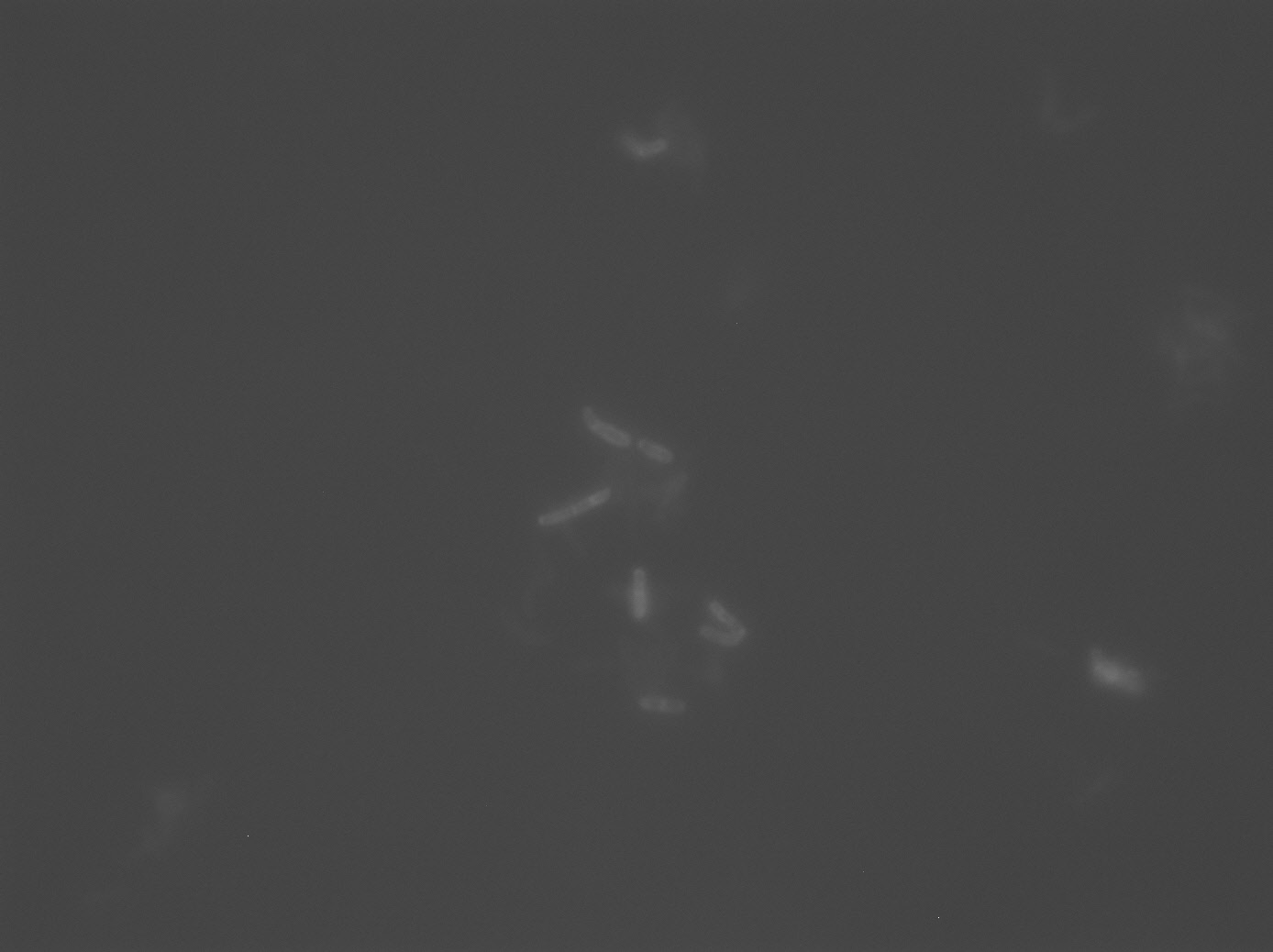

Supplement: Figure 2—figure supplement 2—source data 1. [file elife-37243-fig2-figsupp2-data1.zip › Figure 2--figure supplement 2/Figure 2--figure supplement 2C/alkDADA/GFP 2 sec 11.jpg]

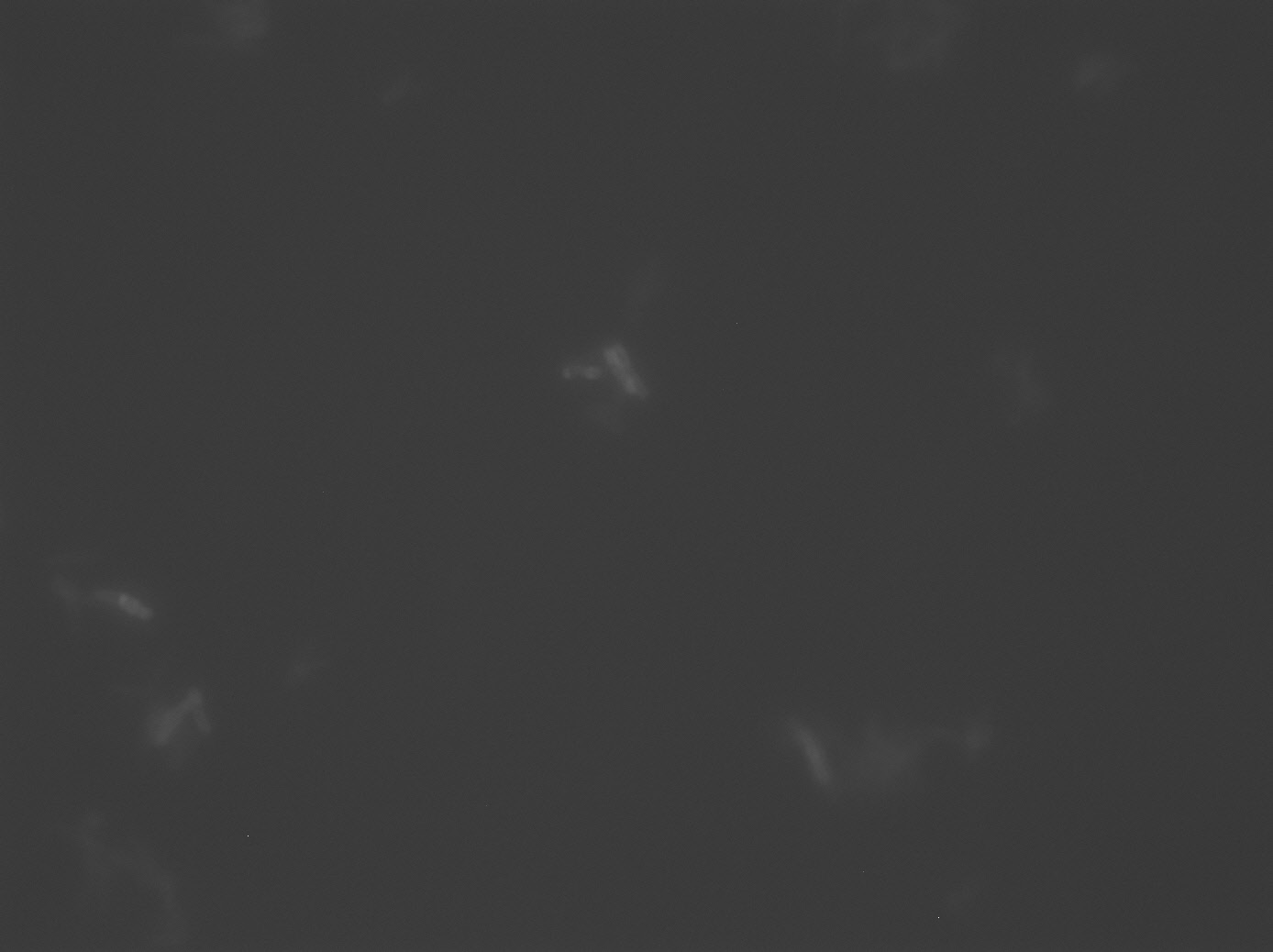

Supplement: Figure 2—figure supplement 2—source data 1. [file elife-37243-fig2-figsupp2-data1.zip › Figure 2--figure supplement 2/Figure 2--figure supplement 2C/alkDADA/GFP 2 sec 7.jpg]

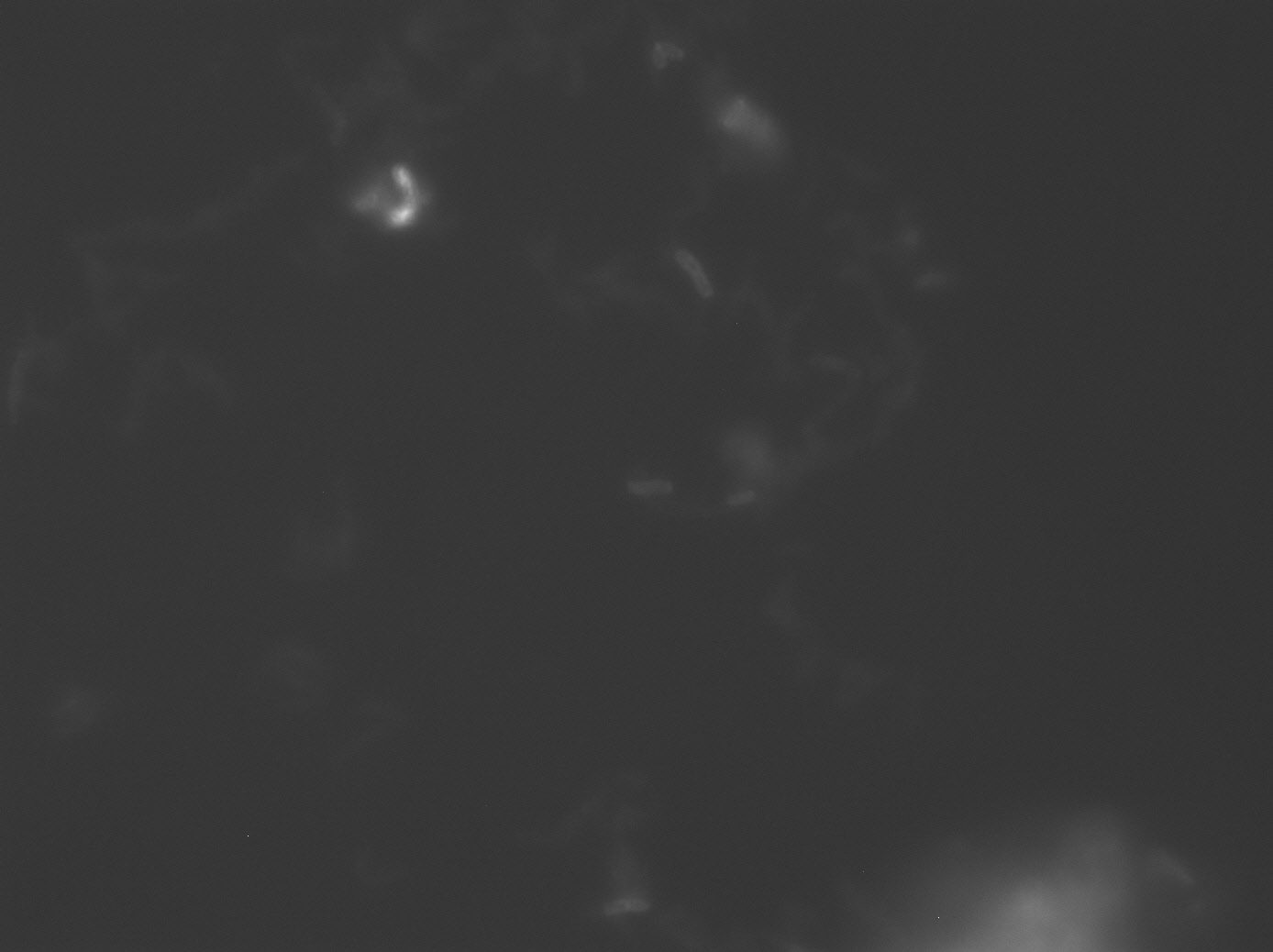

Supplement: Figure 2—figure supplement 2—source data 1. [file elife-37243-fig2-figsupp2-data1.zip › Figure 2--figure supplement 2/Figure 2--figure supplement 2C/alkDADA/GFP 2 sec 9.jpg]

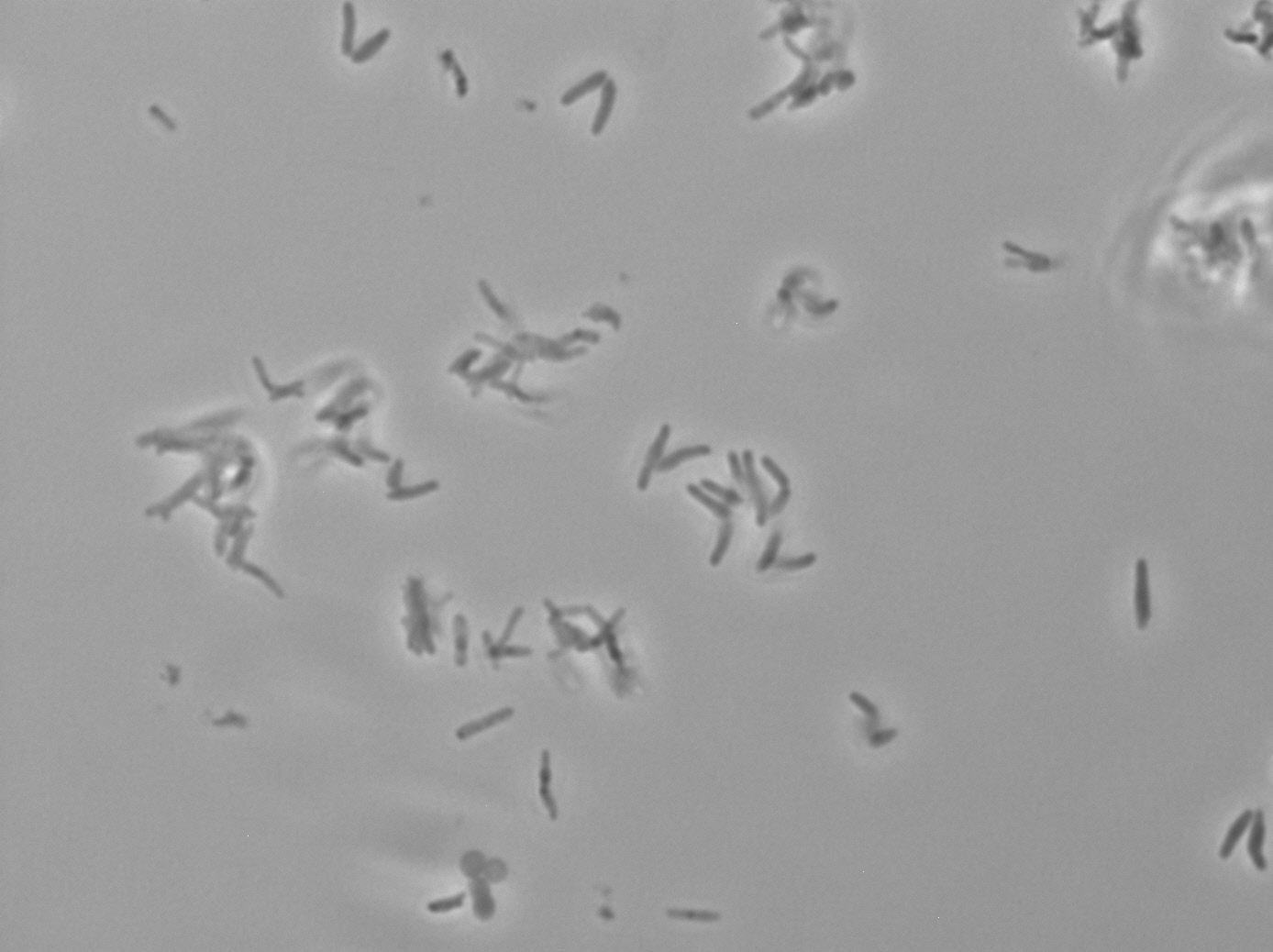

Supplement: Figure 2—figure supplement 2—source data 1. [file elife-37243-fig2-figsupp2-data1.zip › Figure 2--figure supplement 2/Figure 2--figure supplement 2C/alkDADA/PC 10.jpg]

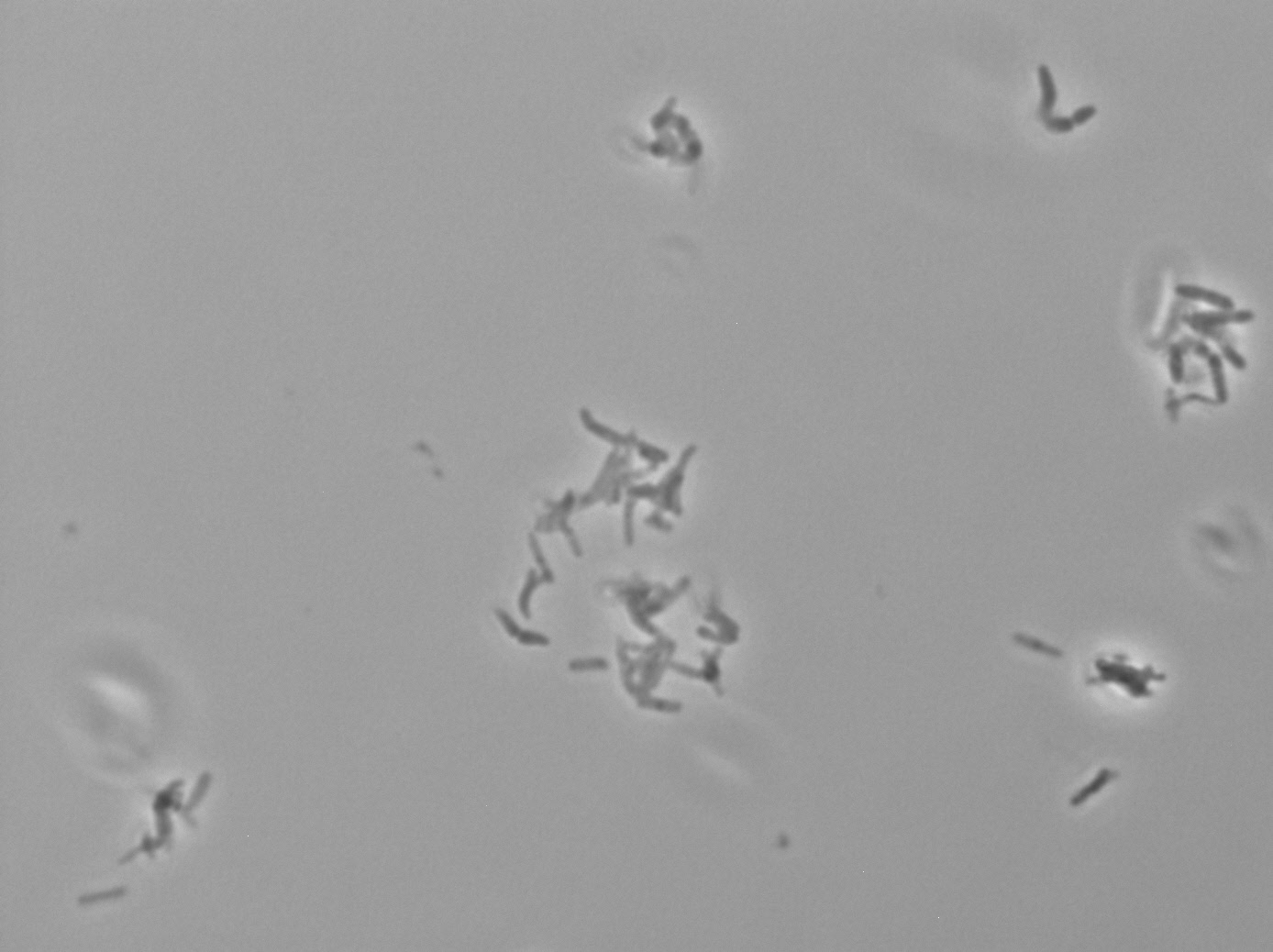

Supplement: Figure 2—figure supplement 2—source data 1. [file elife-37243-fig2-figsupp2-data1.zip › Figure 2--figure supplement 2/Figure 2--figure supplement 2C/alkDADA/PC 11.jpg]

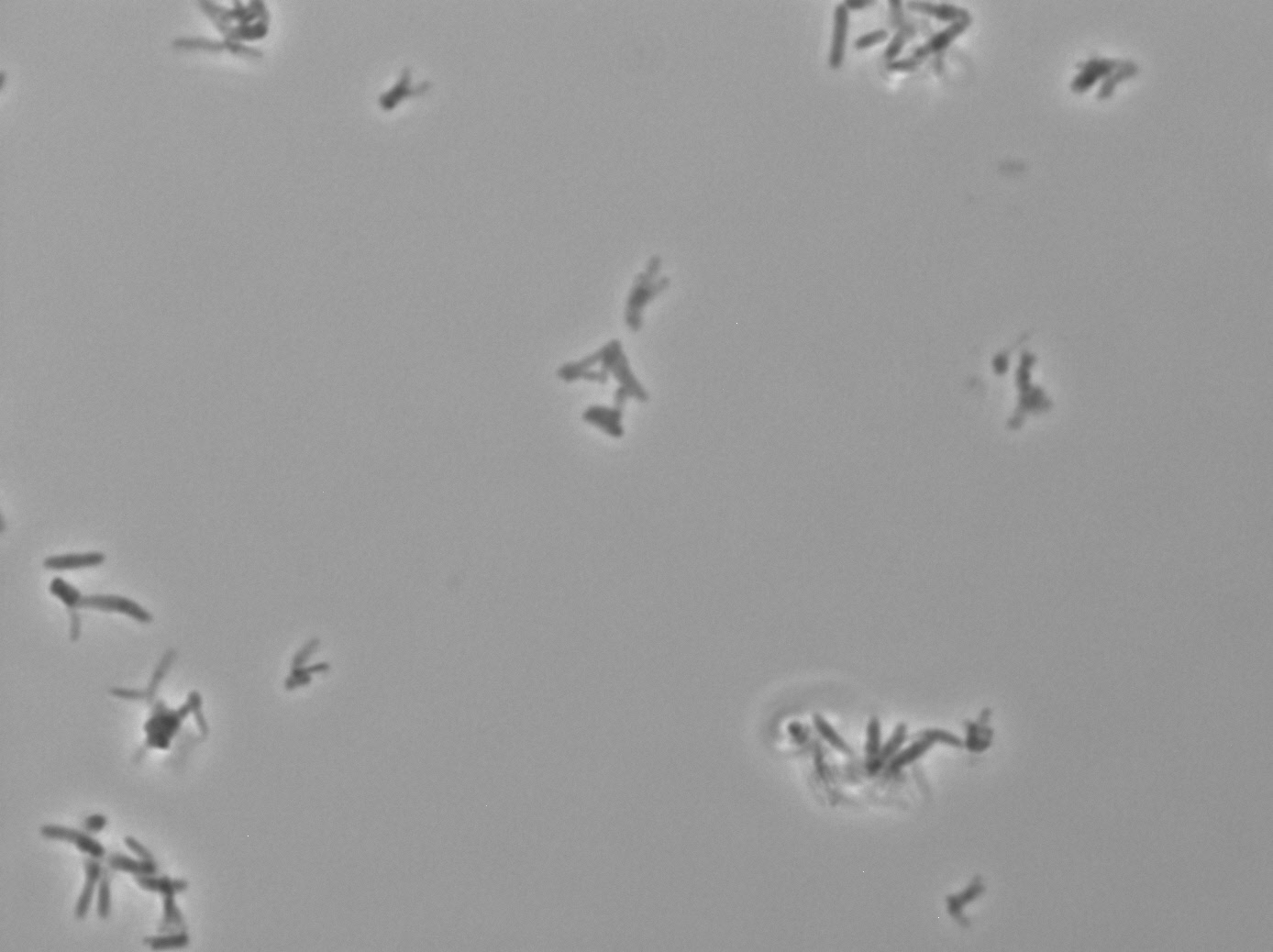

Supplement: Figure 2—figure supplement 2—source data 1. [file elife-37243-fig2-figsupp2-data1.zip › Figure 2--figure supplement 2/Figure 2--figure supplement 2C/alkDADA/PC 7.jpg]

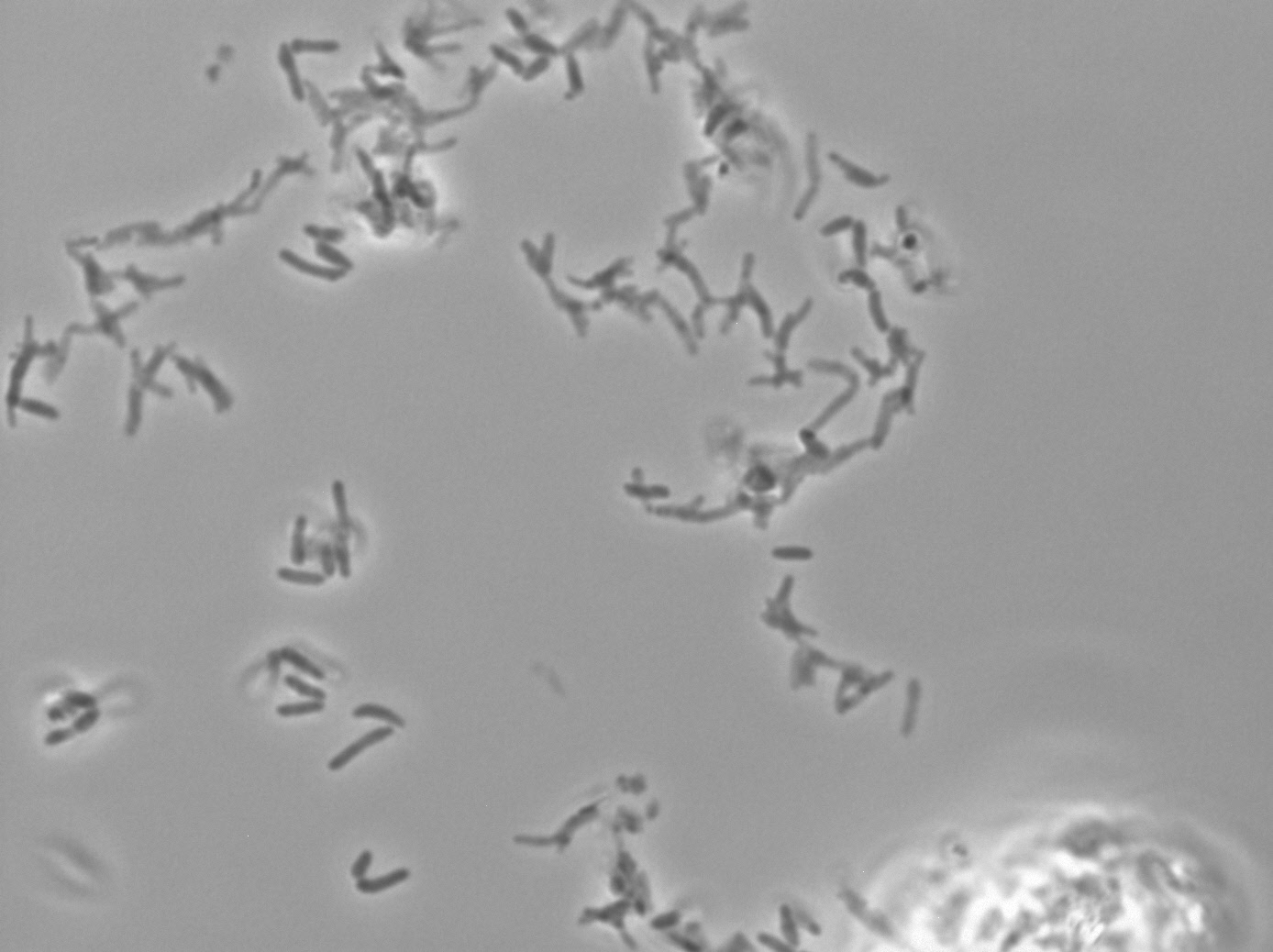

Supplement: Figure 2—figure supplement 2—source data 1. [file elife-37243-fig2-figsupp2-data1.zip › Figure 2--figure supplement 2/Figure 2--figure supplement 2C/alkDADA/PC 9.jpg]

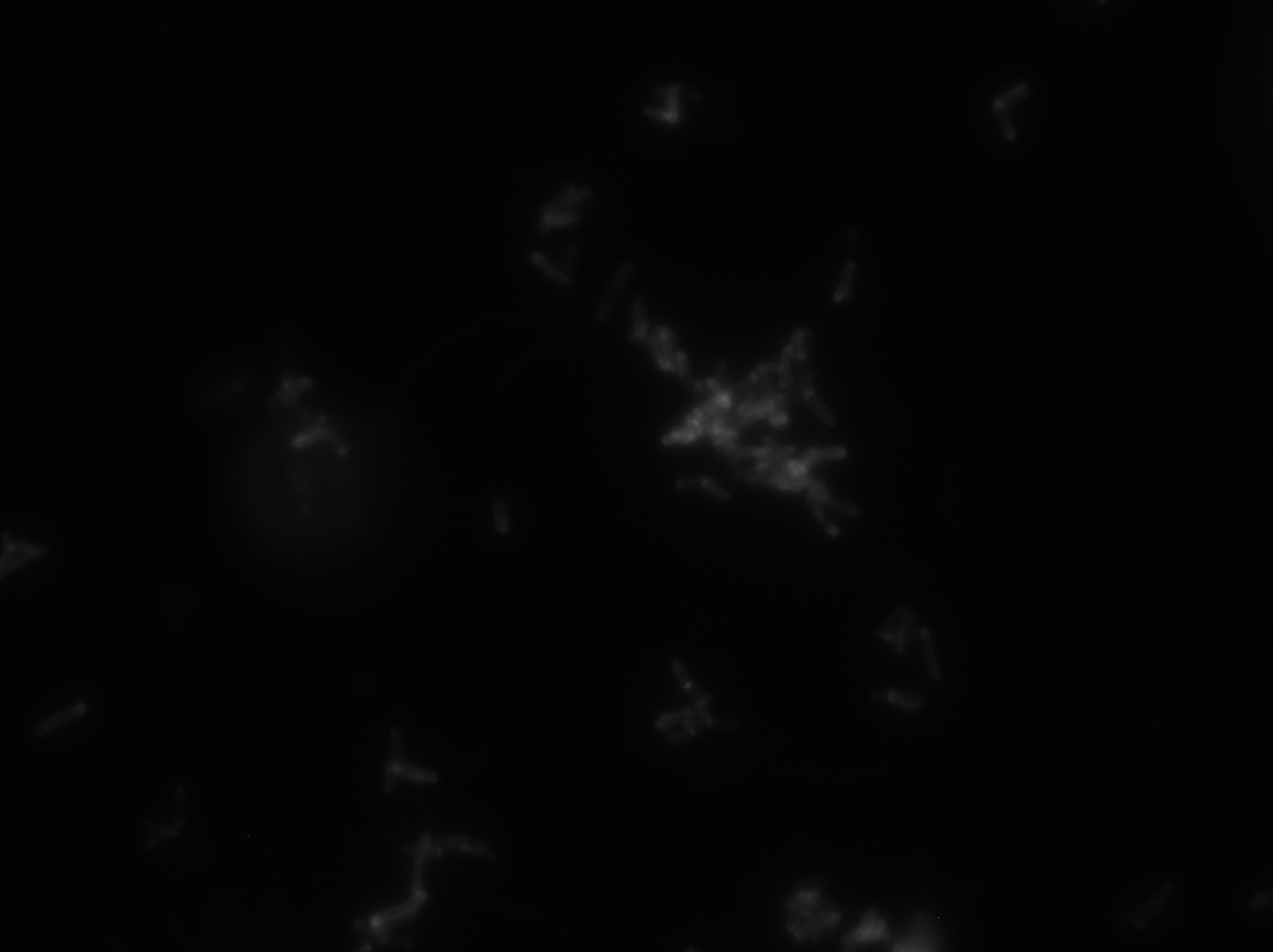

Supplement: Figure 2—figure supplement 2—source data 1. [file elife-37243-fig2-figsupp2-data1.zip › Figure 2--figure supplement 2/Figure 2--figure supplement 2C/HADA/CFP 1 500 ms.jpg]

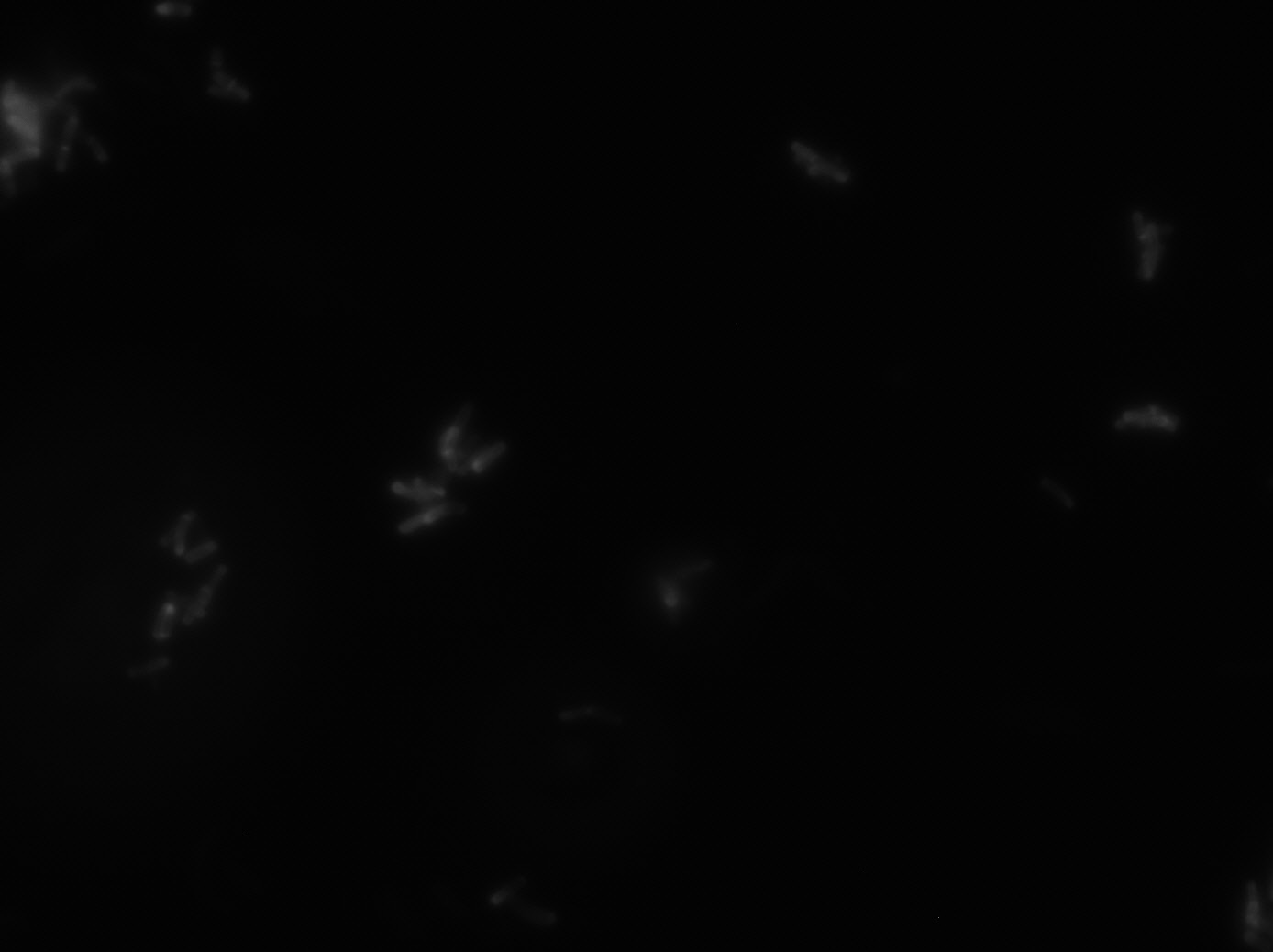

Supplement: Figure 2—figure supplement 2—source data 1. [file elife-37243-fig2-figsupp2-data1.zip › Figure 2--figure supplement 2/Figure 2--figure supplement 2C/HADA/CFP 2 500 ms.jpg]

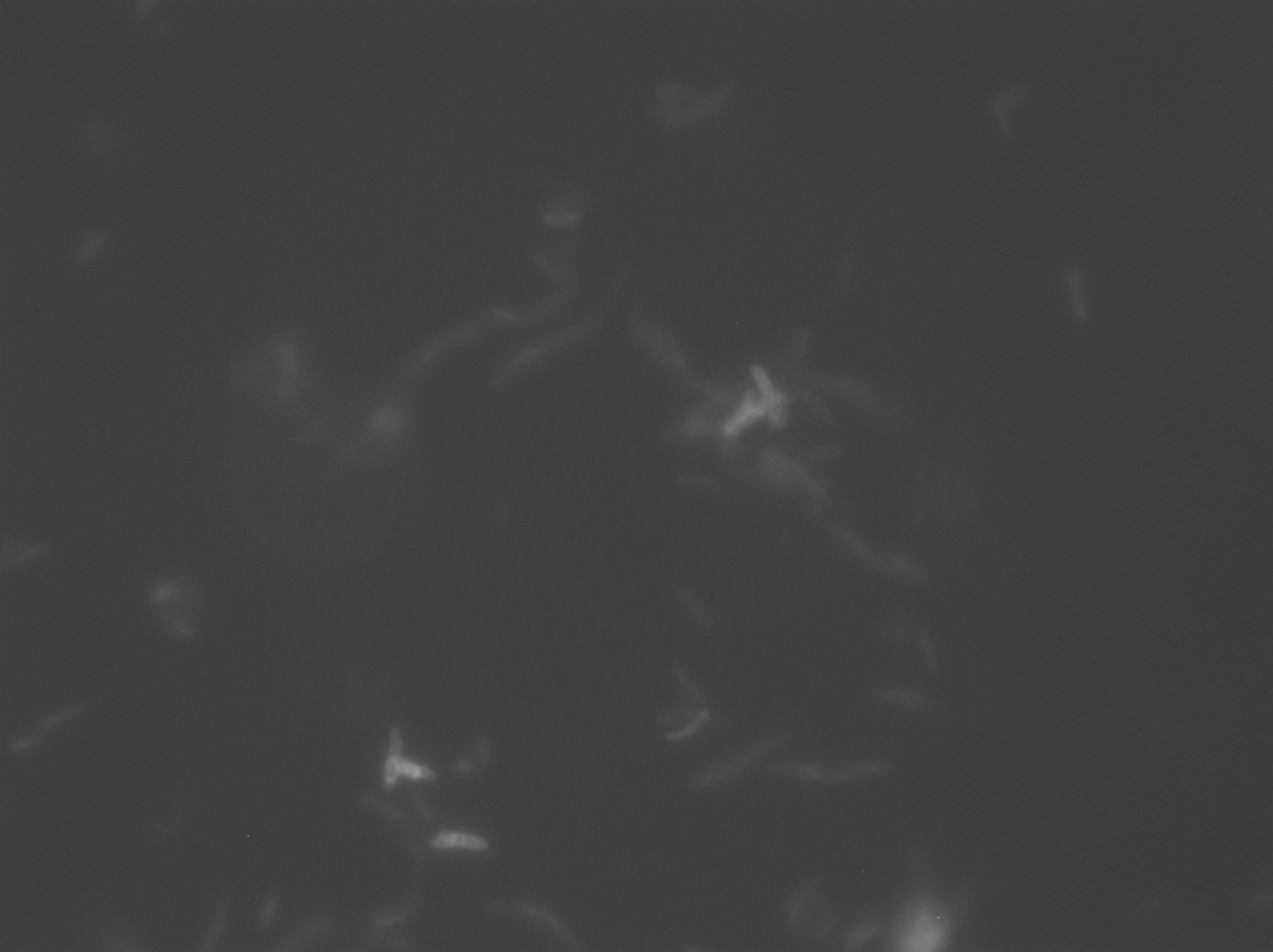

Supplement: Figure 2—figure supplement 2—source data 1. [file elife-37243-fig2-figsupp2-data1.zip › Figure 2--figure supplement 2/Figure 2--figure supplement 2C/HADA/GFP 1.jpg]

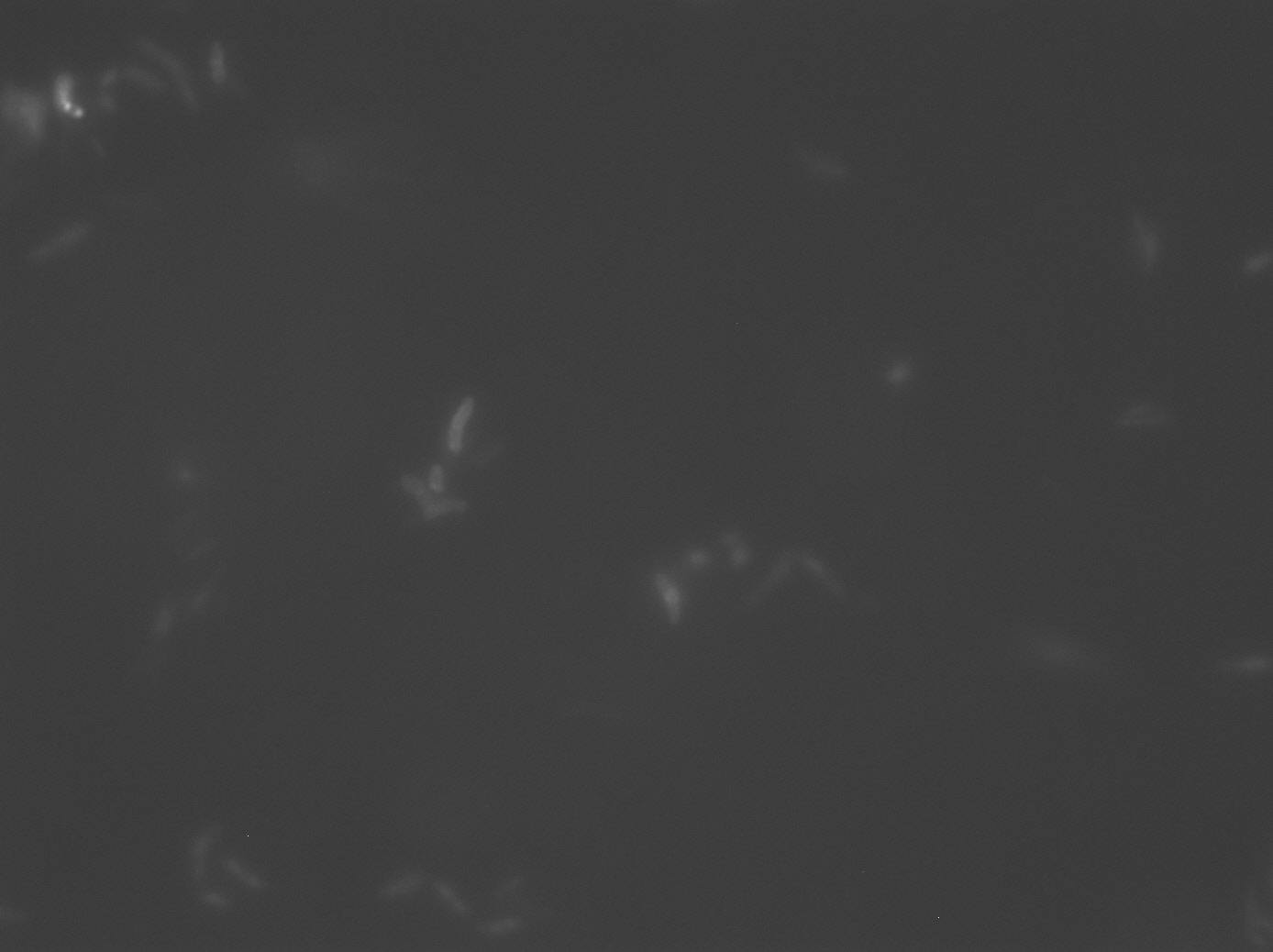

Supplement: Figure 2—figure supplement 2—source data 1. [file elife-37243-fig2-figsupp2-data1.zip › Figure 2--figure supplement 2/Figure 2--figure supplement 2C/HADA/GFP 2.jpg]

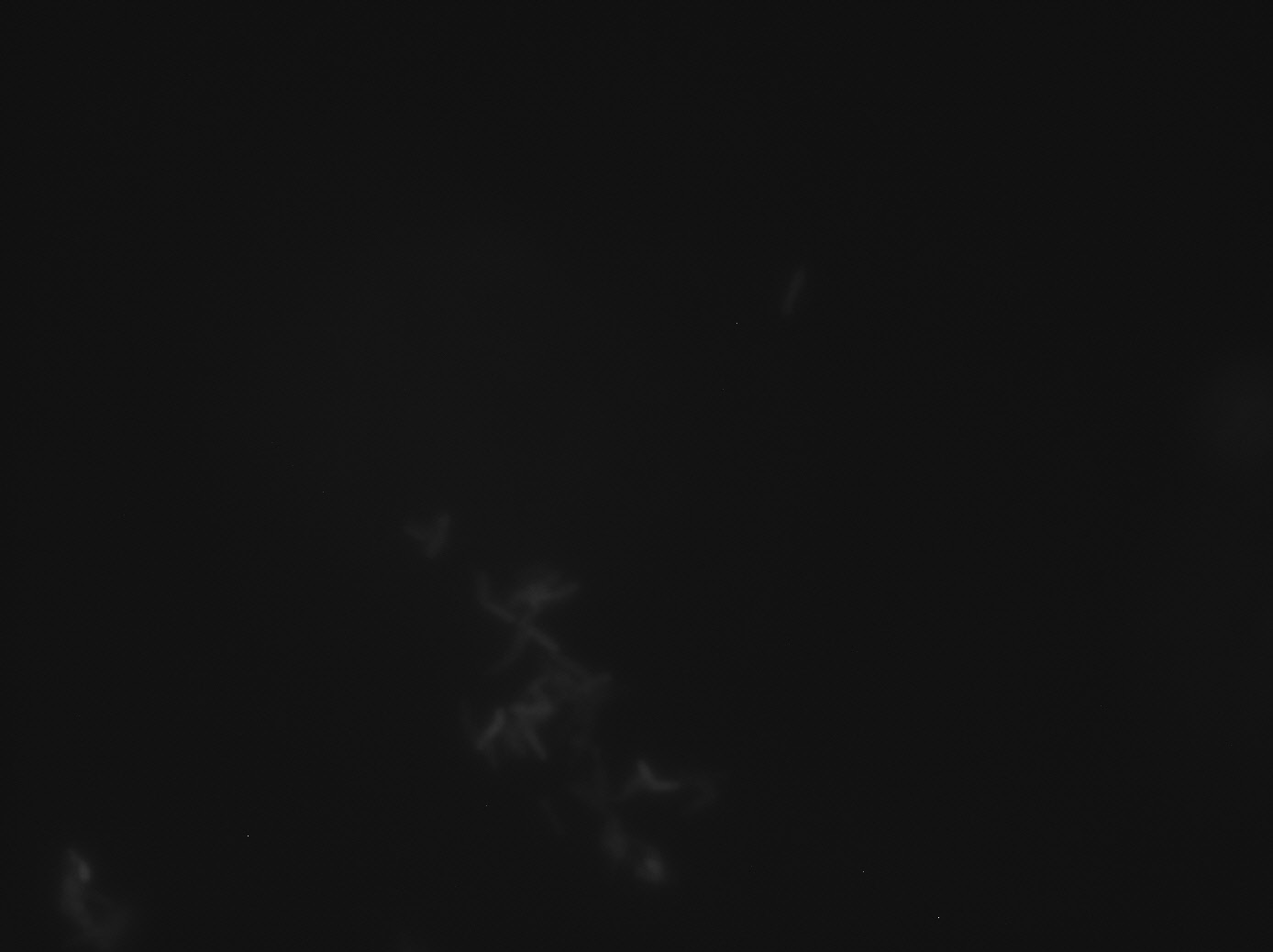

Supplement: Figure 2—figure supplement 2—source data 1. [file elife-37243-fig2-figsupp2-data1.zip › Figure 2--figure supplement 2/Figure 2--figure supplement 2C/no probe control for CuAAC/CFP 1 4 sec.jpg]

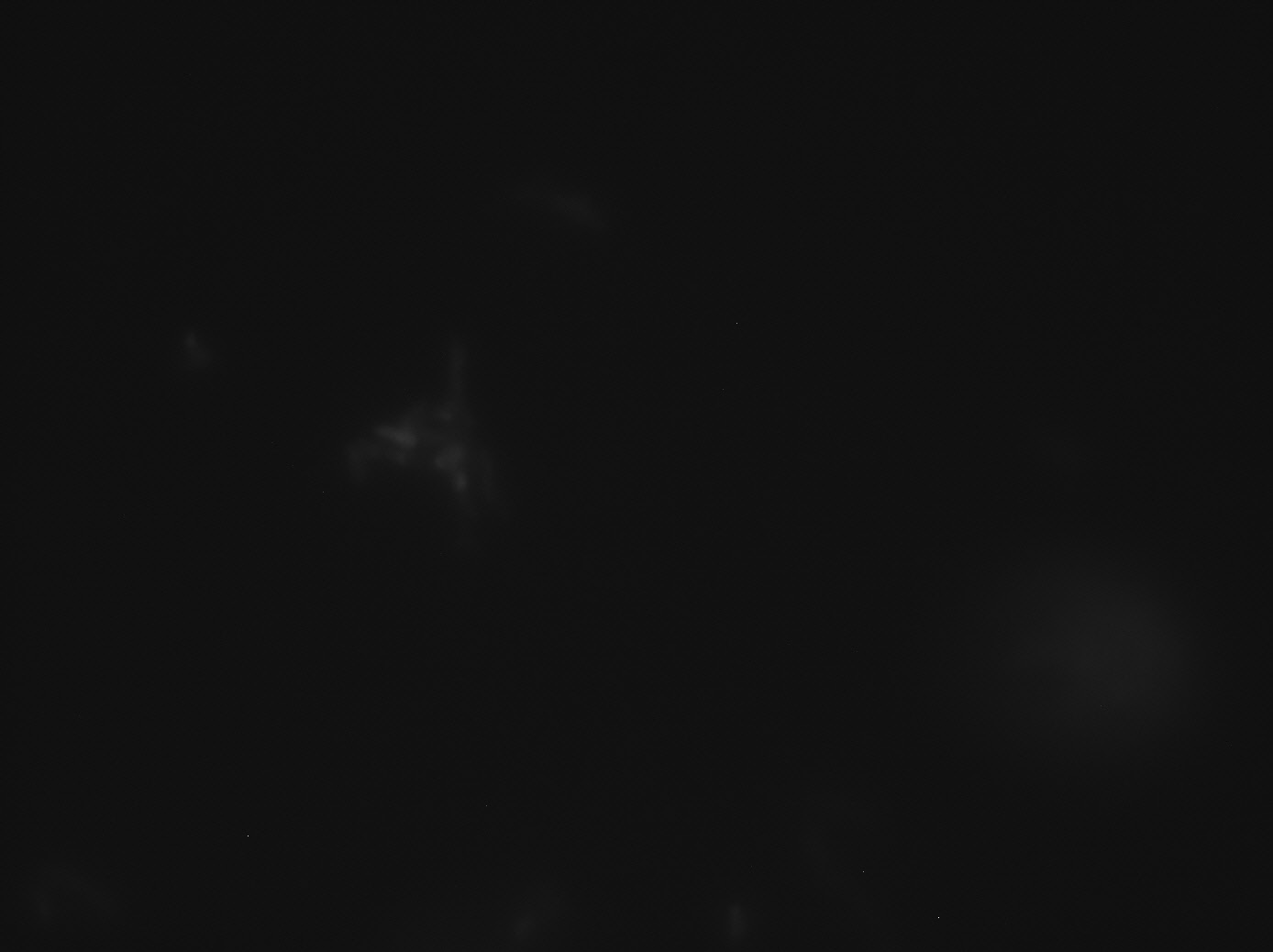

Supplement: Figure 2—figure supplement 2—source data 1. [file elife-37243-fig2-figsupp2-data1.zip › Figure 2--figure supplement 2/Figure 2--figure supplement 2C/no probe control for CuAAC/CFP 2 4 sec.jpg]

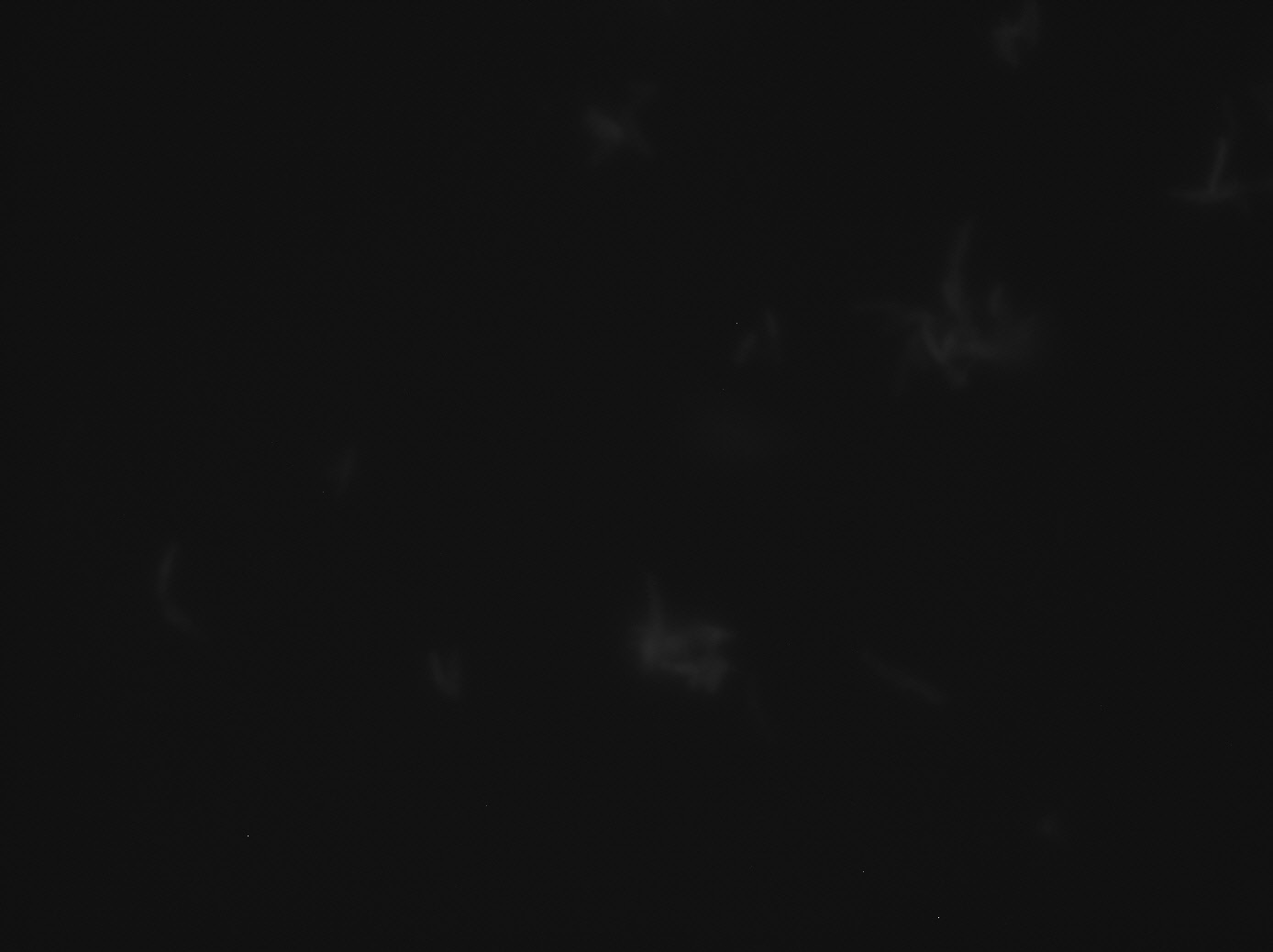

Supplement: Figure 2—figure supplement 2—source data 1. [file elife-37243-fig2-figsupp2-data1.zip › Figure 2--figure supplement 2/Figure 2--figure supplement 2C/no probe control for CuAAC/CFP 3 4 sec.jpg]

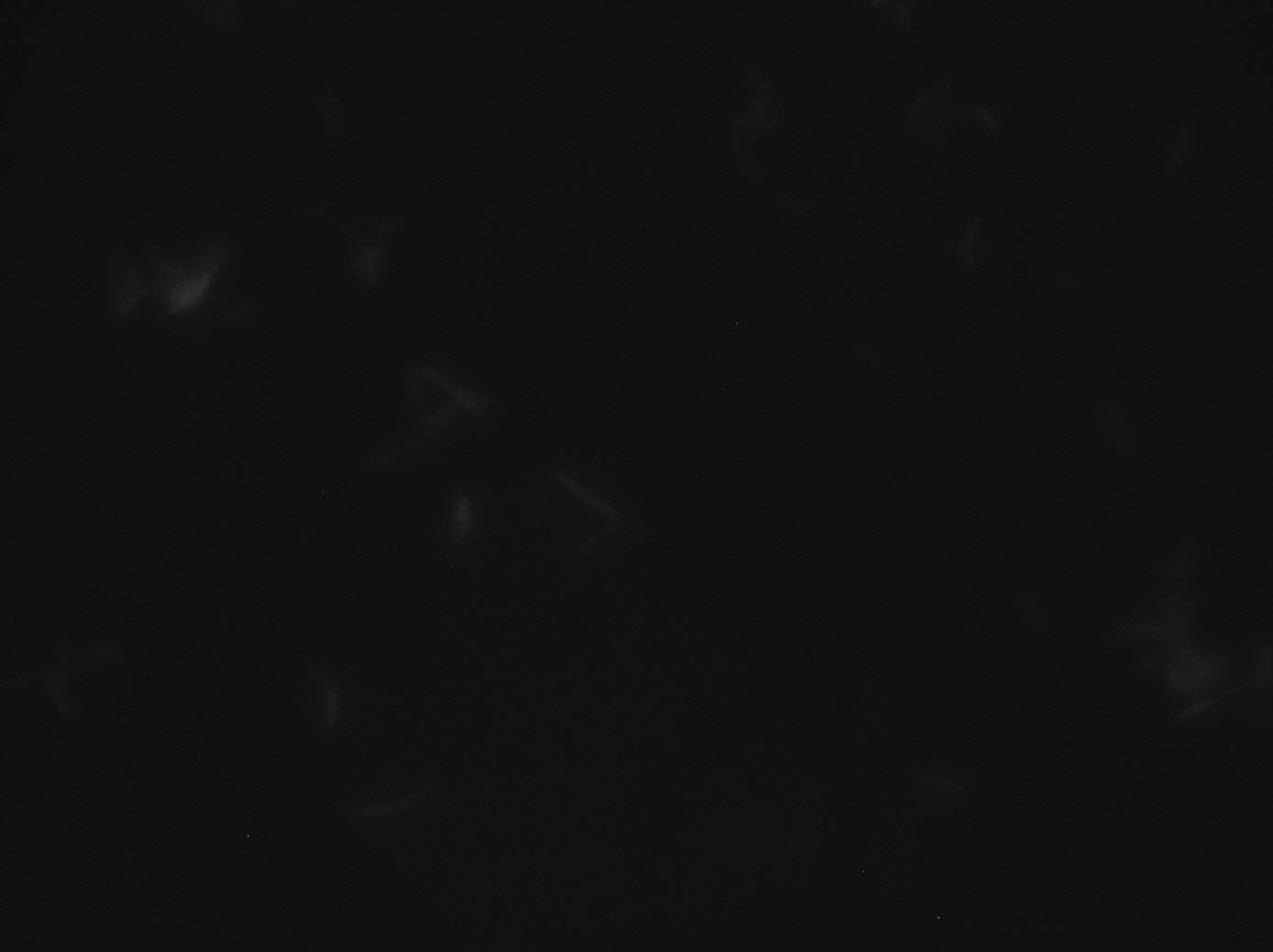

Supplement: Figure 2—figure supplement 2—source data 1. [file elife-37243-fig2-figsupp2-data1.zip › Figure 2--figure supplement 2/Figure 2--figure supplement 2C/no probe control for CuAAC/CFP 4 4 sec.jpg]

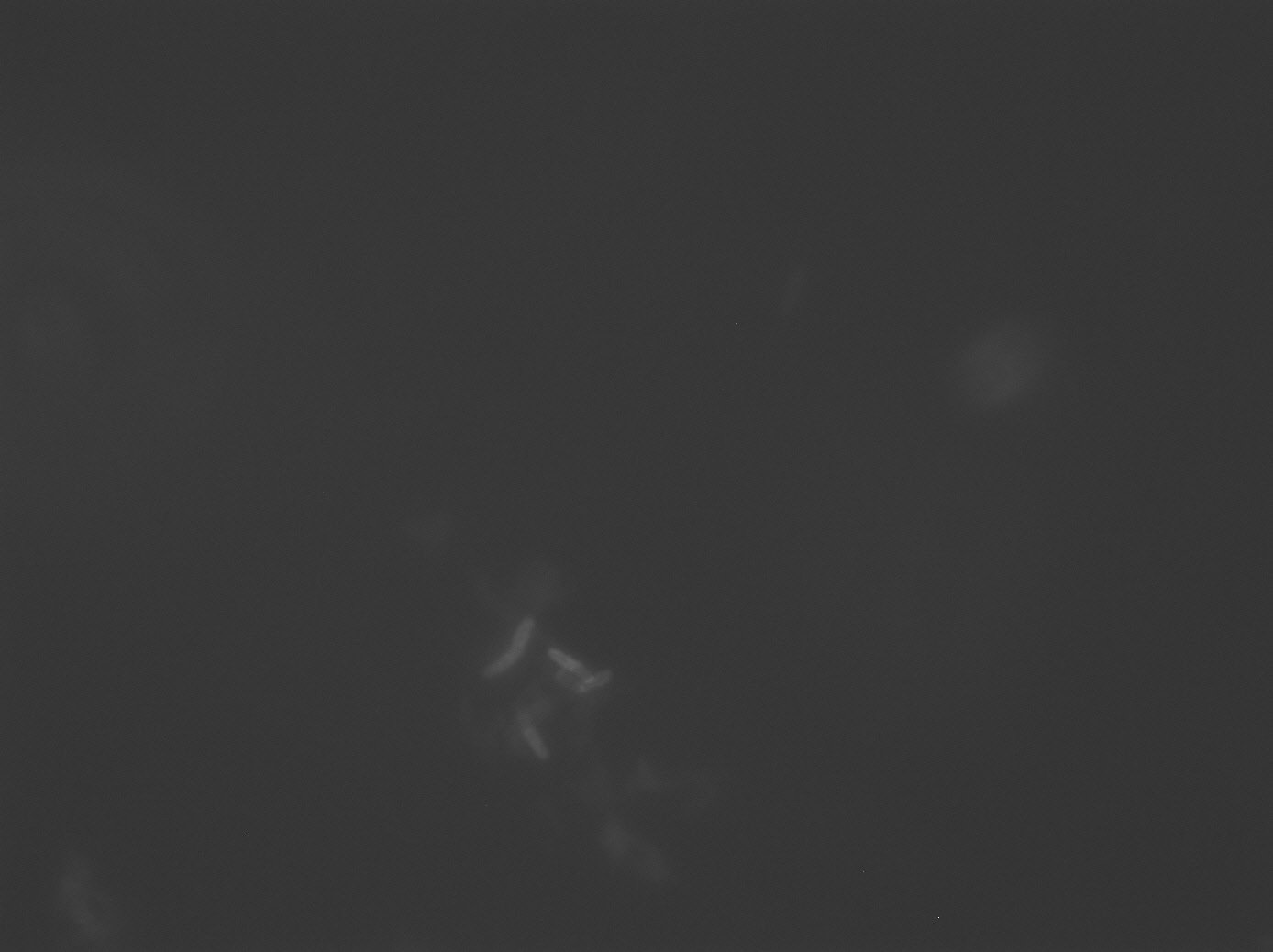

Supplement: Figure 2—figure supplement 2—source data 1. [file elife-37243-fig2-figsupp2-data1.zip › Figure 2--figure supplement 2/Figure 2--figure supplement 2C/no probe control for CuAAC/GFP 1.jpg]

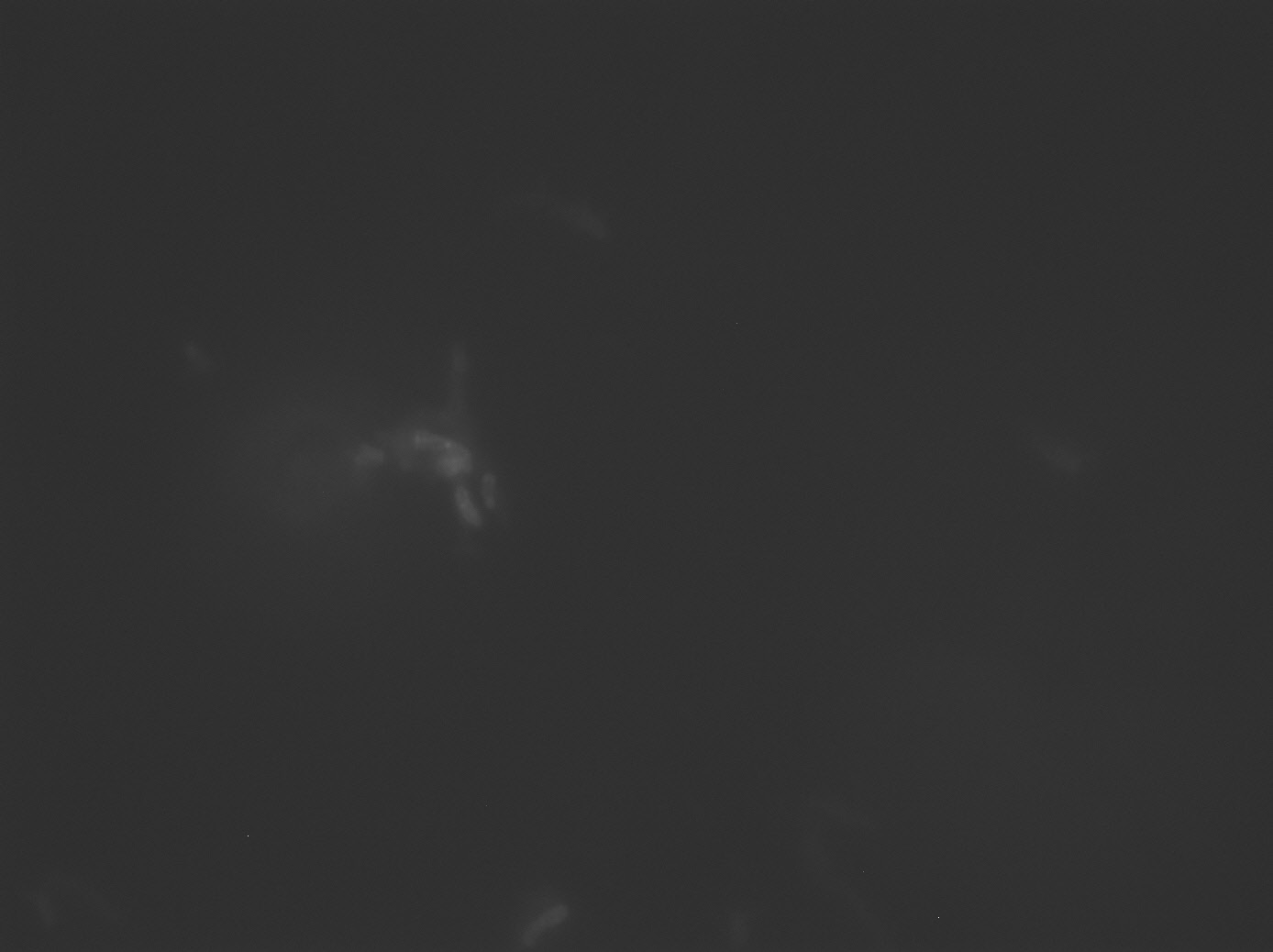

Supplement: Figure 2—figure supplement 2—source data 1. [file elife-37243-fig2-figsupp2-data1.zip › Figure 2--figure supplement 2/Figure 2--figure supplement 2C/no probe control for CuAAC/GFP 2.jpg]

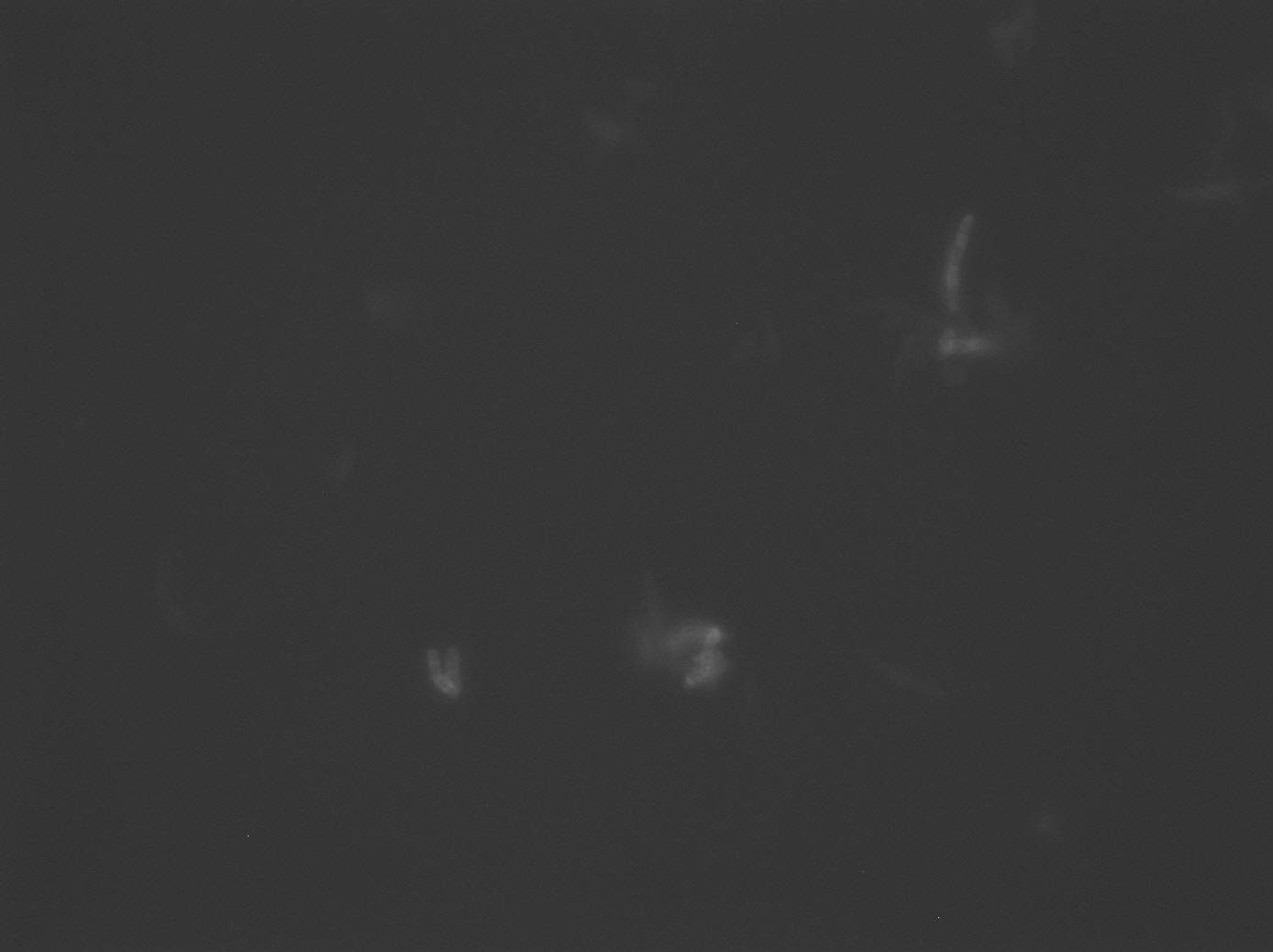

Supplement: Figure 2—figure supplement 2—source data 1. [file elife-37243-fig2-figsupp2-data1.zip › Figure 2--figure supplement 2/Figure 2--figure supplement 2C/no probe control for CuAAC/GFP 3.jpg]

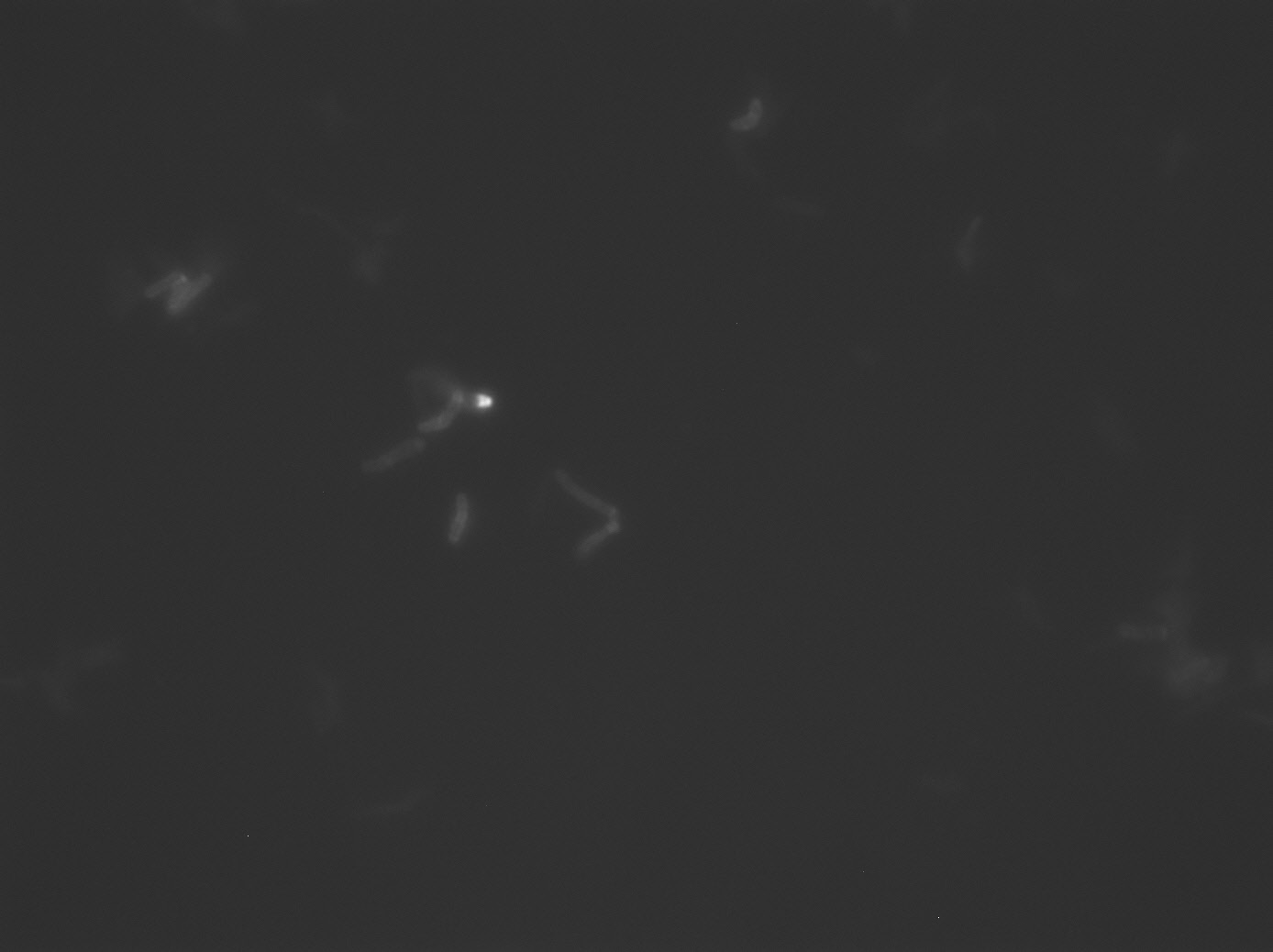

Supplement: Figure 2—figure supplement 2—source data 1. [file elife-37243-fig2-figsupp2-data1.zip › Figure 2--figure supplement 2/Figure 2--figure supplement 2C/no probe control for CuAAC/GFP 4.jpg]

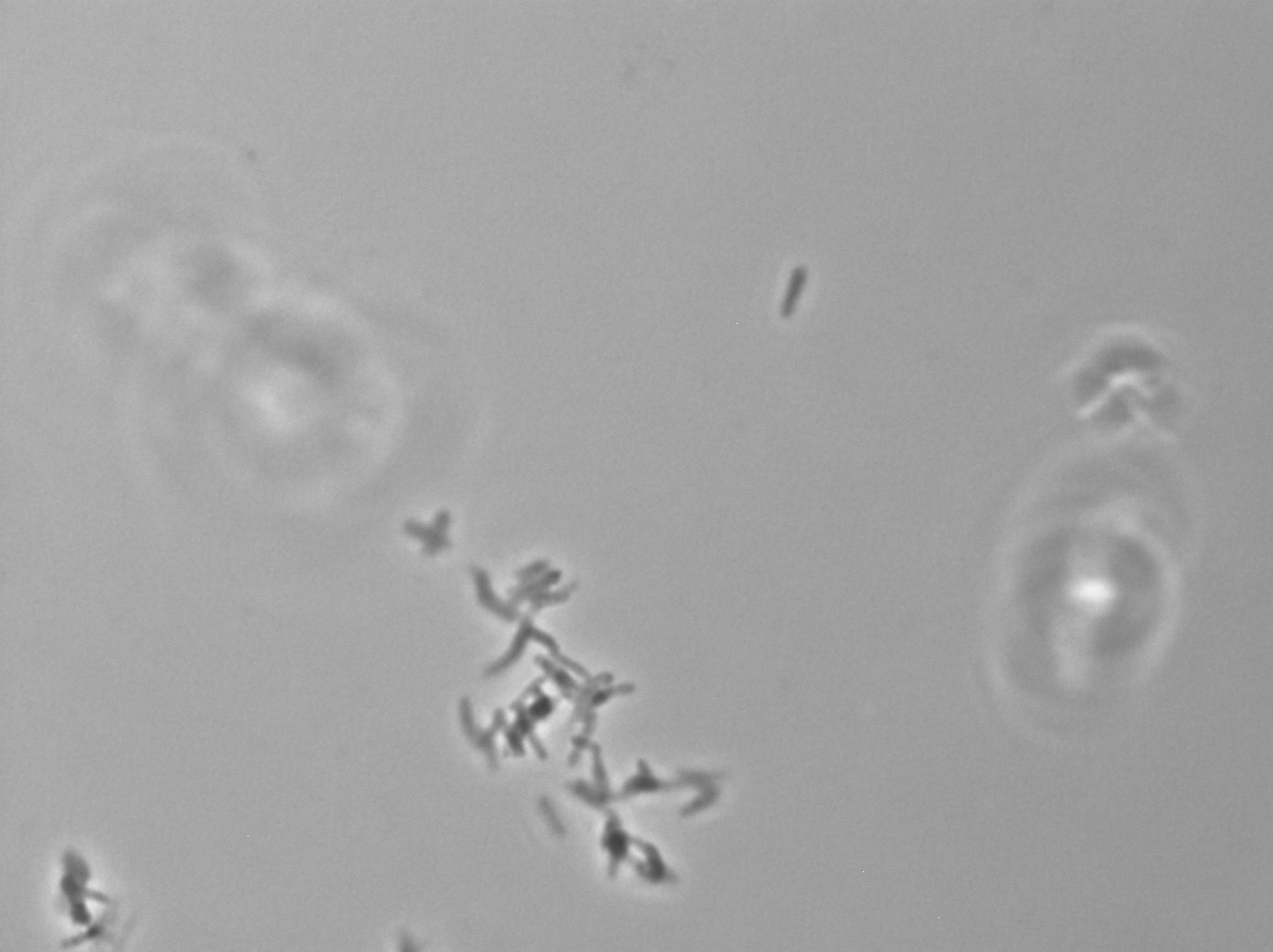

Supplement: Figure 2—figure supplement 2—source data 1. [file elife-37243-fig2-figsupp2-data1.zip › Figure 2--figure supplement 2/Figure 2--figure supplement 2C/no probe control for CuAAC/PC 1 .jpg]

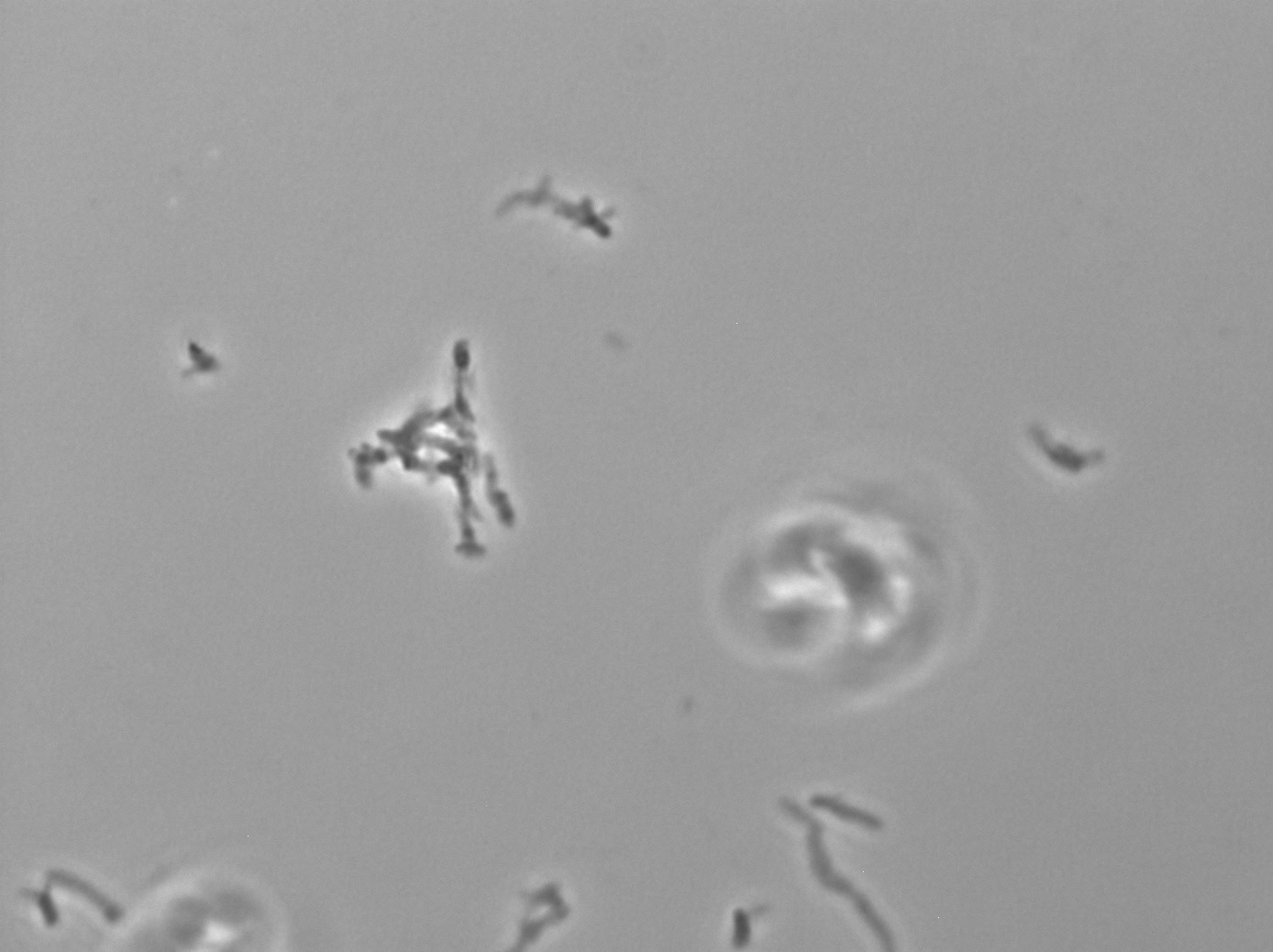

Supplement: Figure 2—figure supplement 2—source data 1. [file elife-37243-fig2-figsupp2-data1.zip › Figure 2--figure supplement 2/Figure 2--figure supplement 2C/no probe control for CuAAC/PC 2 2 sec.jpg]

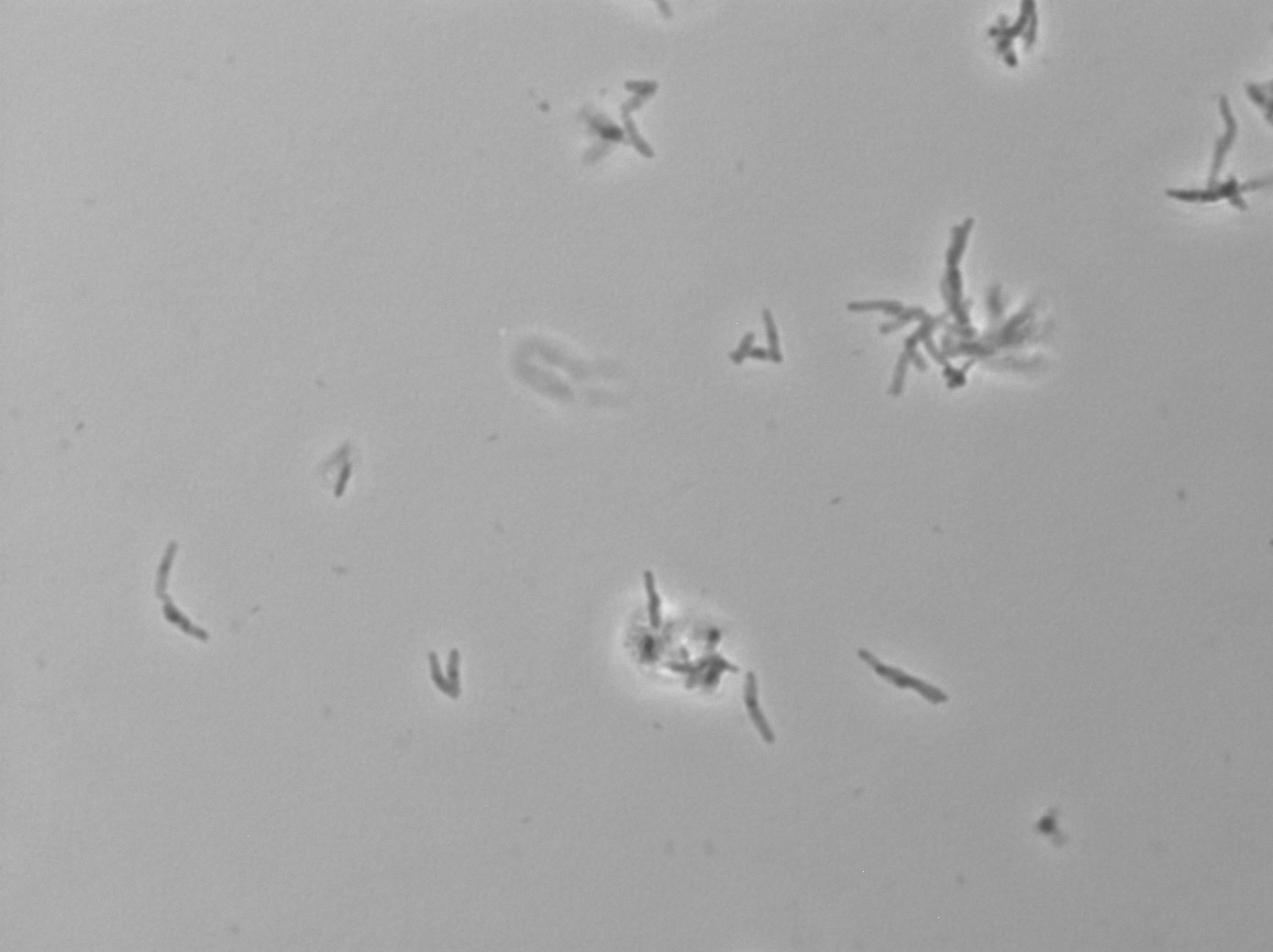

Supplement: Figure 2—figure supplement 2—source data 1. [file elife-37243-fig2-figsupp2-data1.zip › Figure 2--figure supplement 2/Figure 2--figure supplement 2C/no probe control for CuAAC/PC 3.jpg]

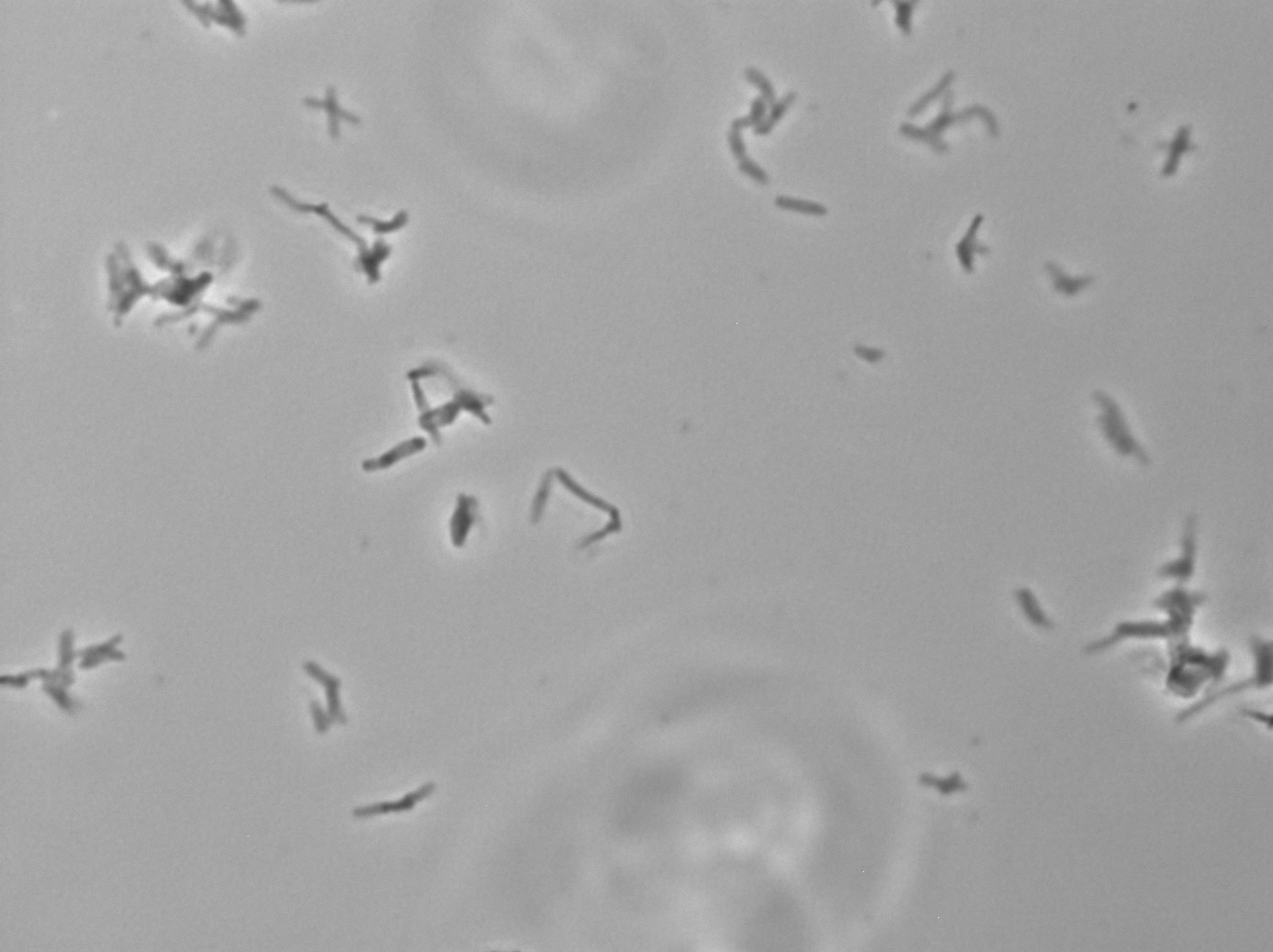

Supplement: Figure 2—figure supplement 2—source data 1. [file elife-37243-fig2-figsupp2-data1.zip › Figure 2--figure supplement 2/Figure 2--figure supplement 2C/no probe control for CuAAC/PC 4.jpg]

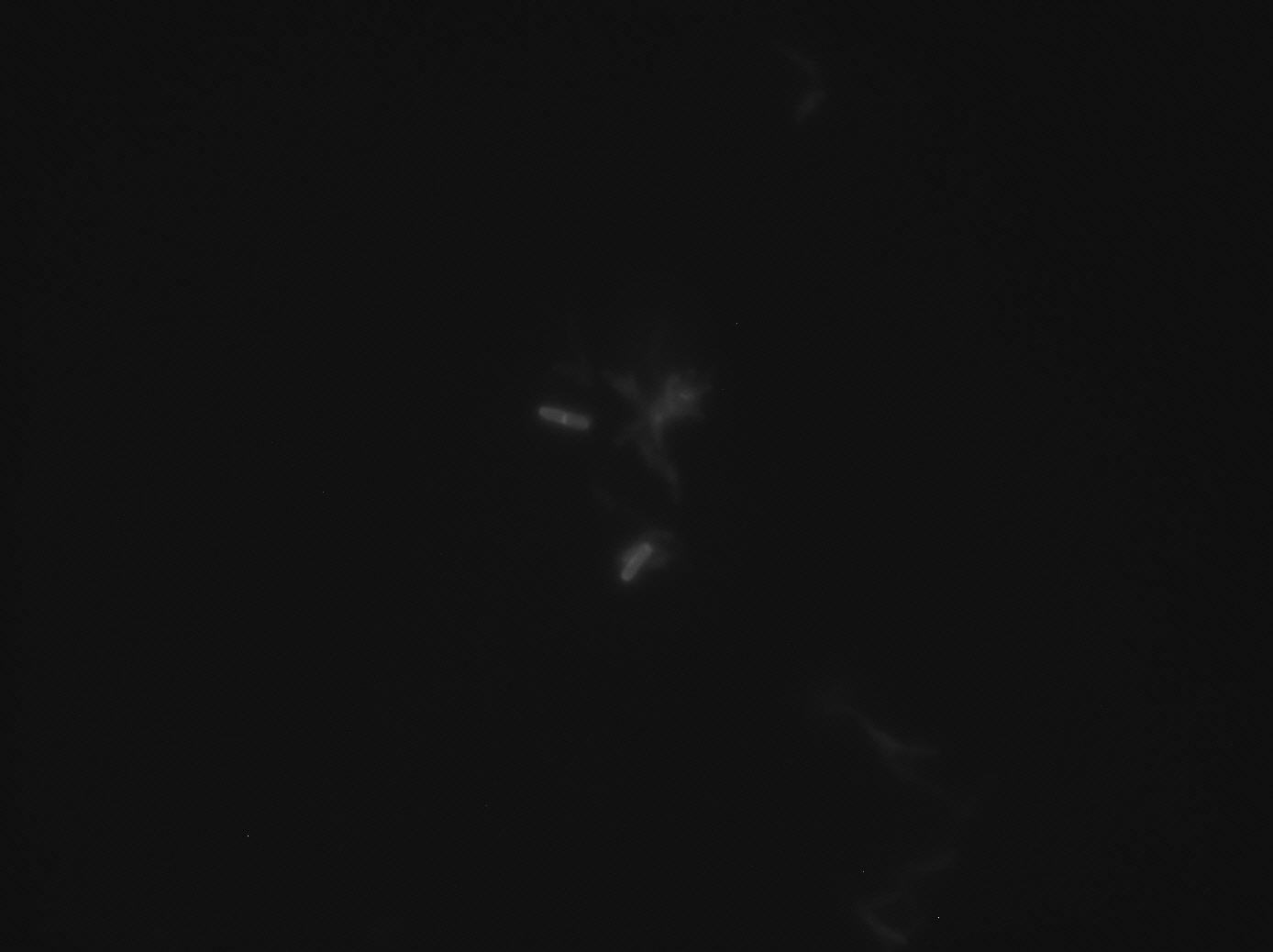

Supplement: Figure 2—figure supplement 2—source data 1. [file elife-37243-fig2-figsupp2-data1.zip › Figure 2--figure supplement 2/Figure 2--figure supplement 2C/OalkTMM/CFP 1 4 sec.jpg]

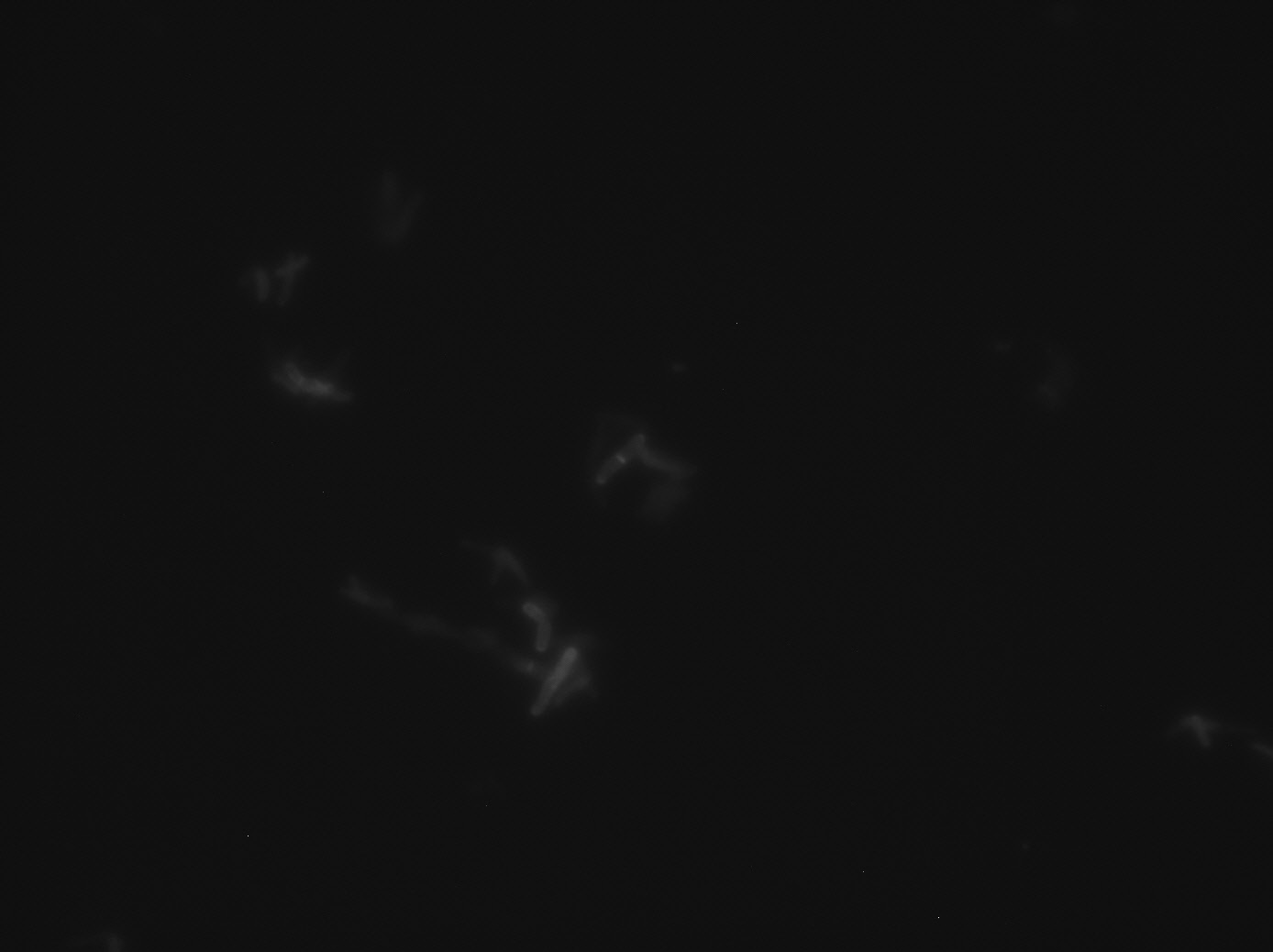

Supplement: Figure 2—figure supplement 2—source data 1. [file elife-37243-fig2-figsupp2-data1.zip › Figure 2--figure supplement 2/Figure 2--figure supplement 2C/OalkTMM/CFP 10 4 sec.jpg]

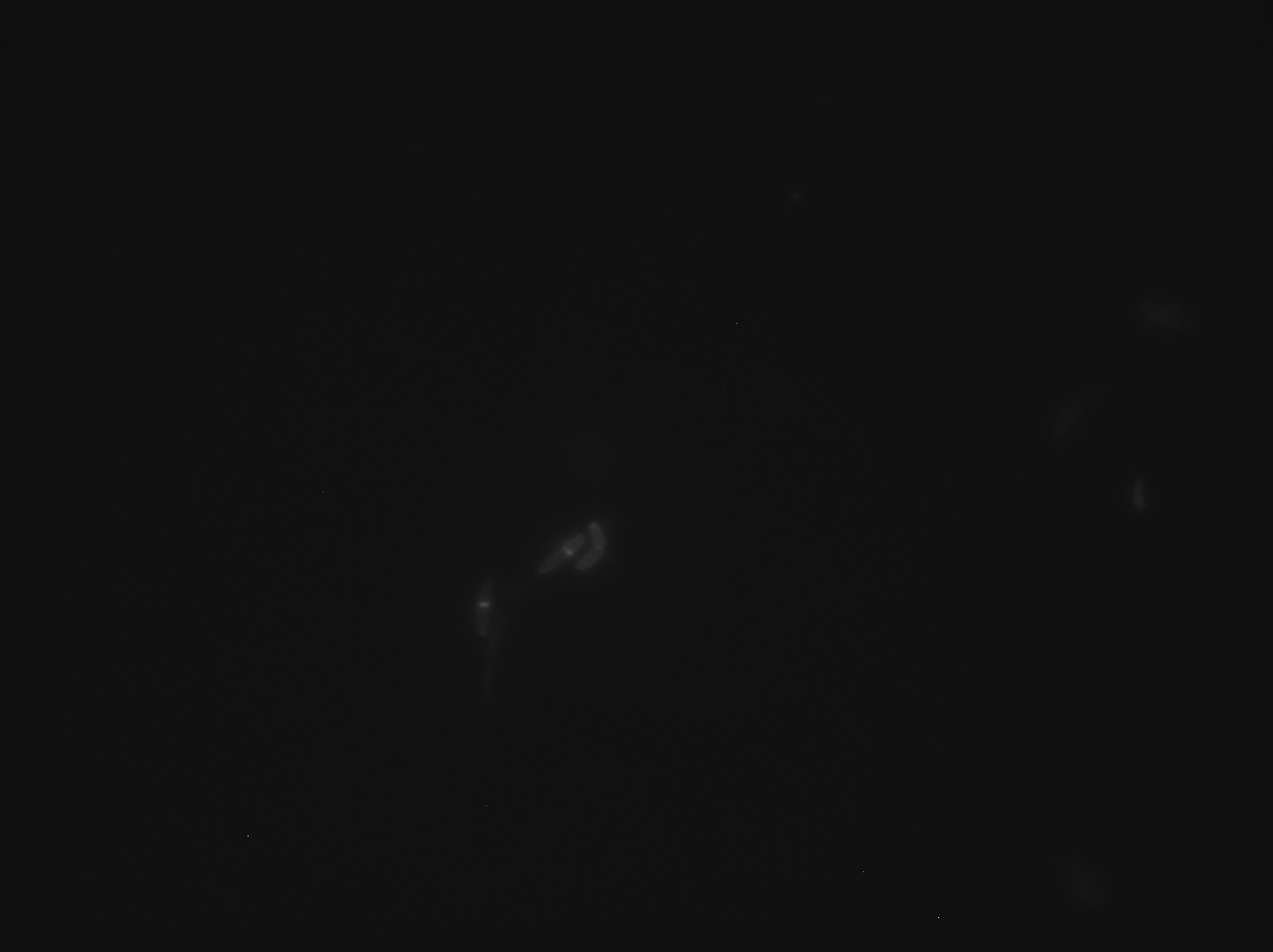

Supplement: Figure 2—figure supplement 2—source data 1. [file elife-37243-fig2-figsupp2-data1.zip › Figure 2--figure supplement 2/Figure 2--figure supplement 2C/OalkTMM/CFP 2 4 sec.jpg]

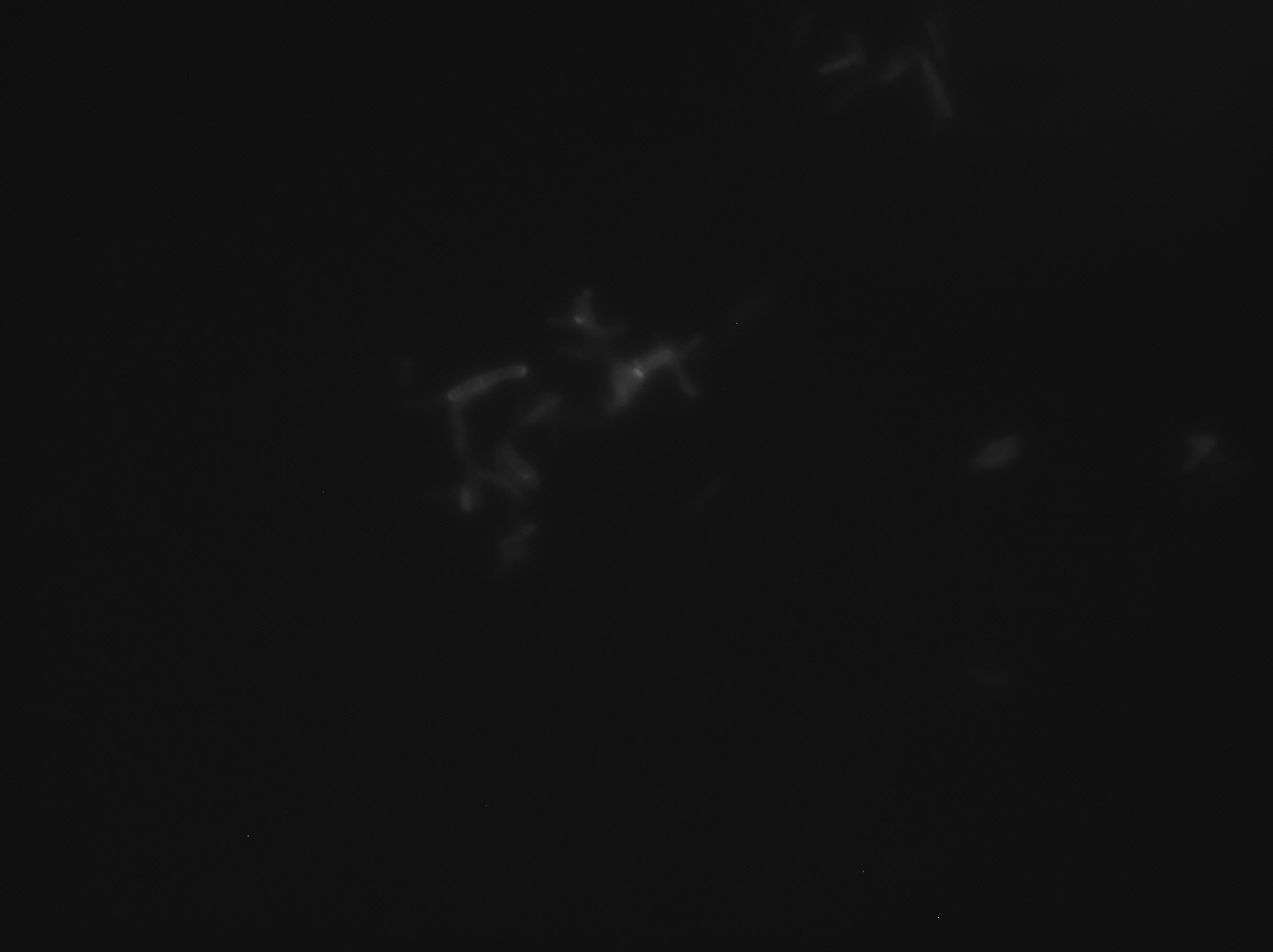

Supplement: Figure 2—figure supplement 2—source data 1. [file elife-37243-fig2-figsupp2-data1.zip › Figure 2--figure supplement 2/Figure 2--figure supplement 2C/OalkTMM/CFP 6 4 sec.jpg]

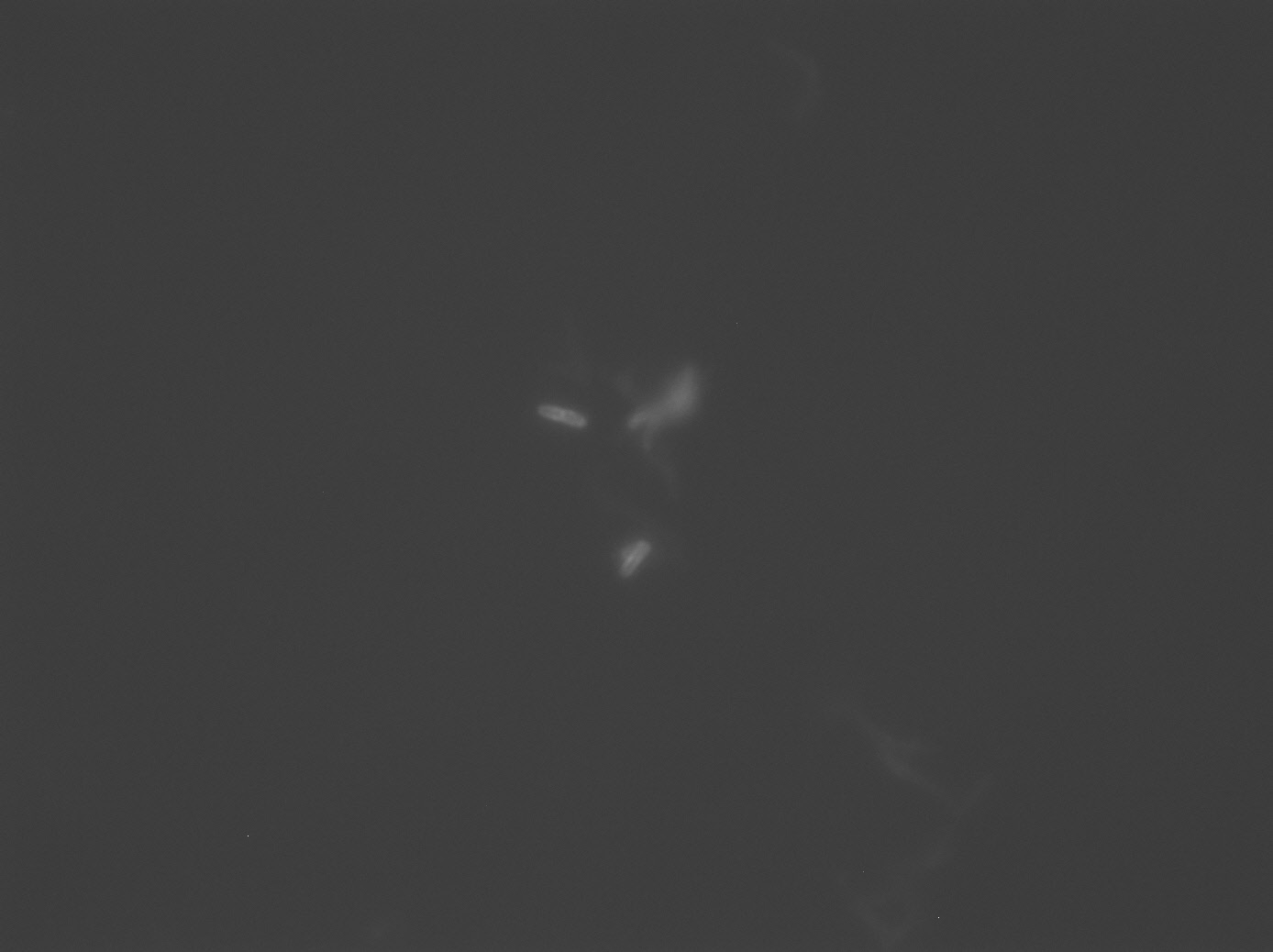

Supplement: Figure 2—figure supplement 2—source data 1. [file elife-37243-fig2-figsupp2-data1.zip › Figure 2--figure supplement 2/Figure 2--figure supplement 2C/OalkTMM/GFP 1.jpg]

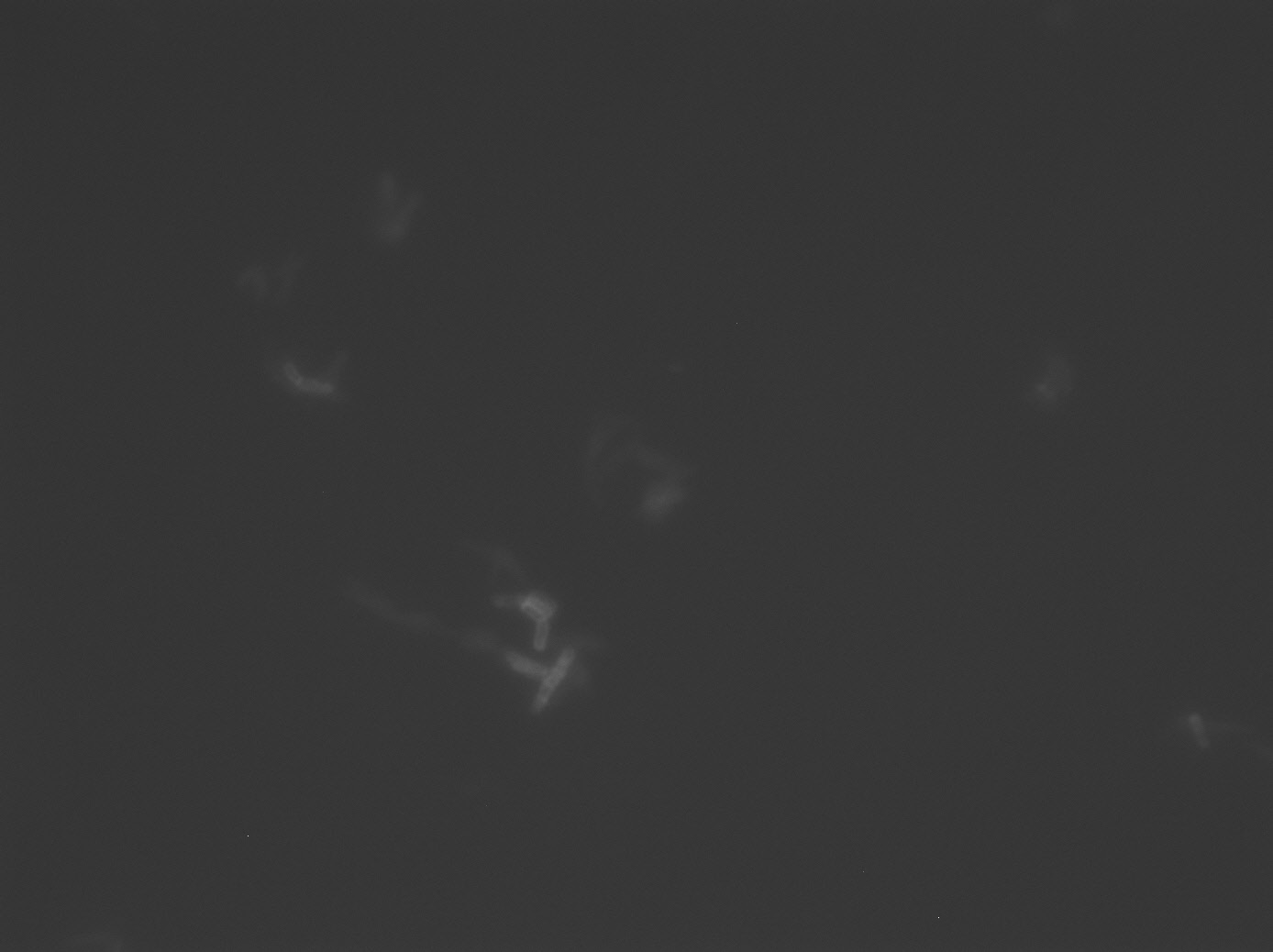

Supplement: Figure 2—figure supplement 2—source data 1. [file elife-37243-fig2-figsupp2-data1.zip › Figure 2--figure supplement 2/Figure 2--figure supplement 2C/OalkTMM/GFP 10.jpg]

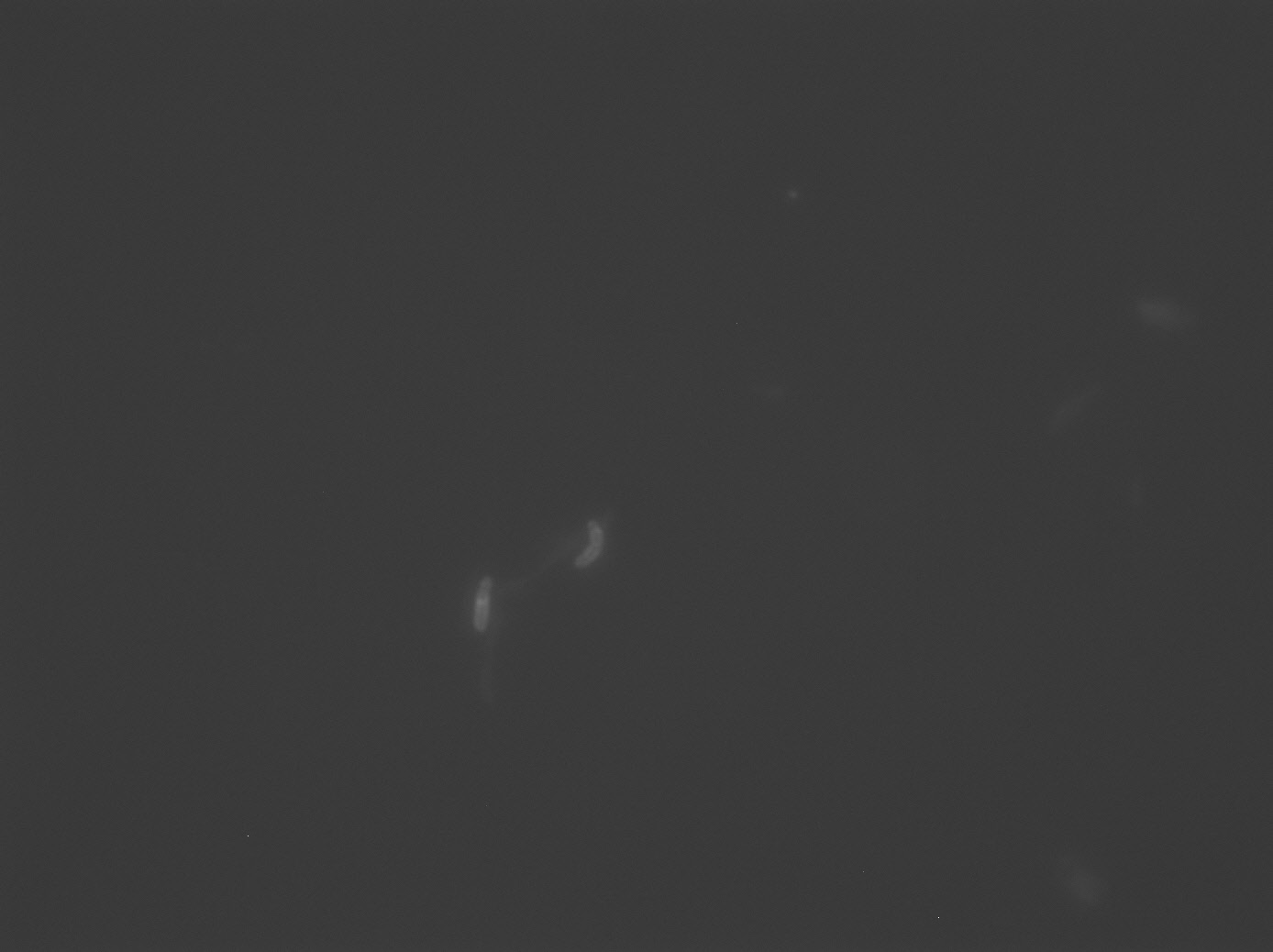

Supplement: Figure 2—figure supplement 2—source data 1. [file elife-37243-fig2-figsupp2-data1.zip › Figure 2--figure supplement 2/Figure 2--figure supplement 2C/OalkTMM/GFP 2.jpg]

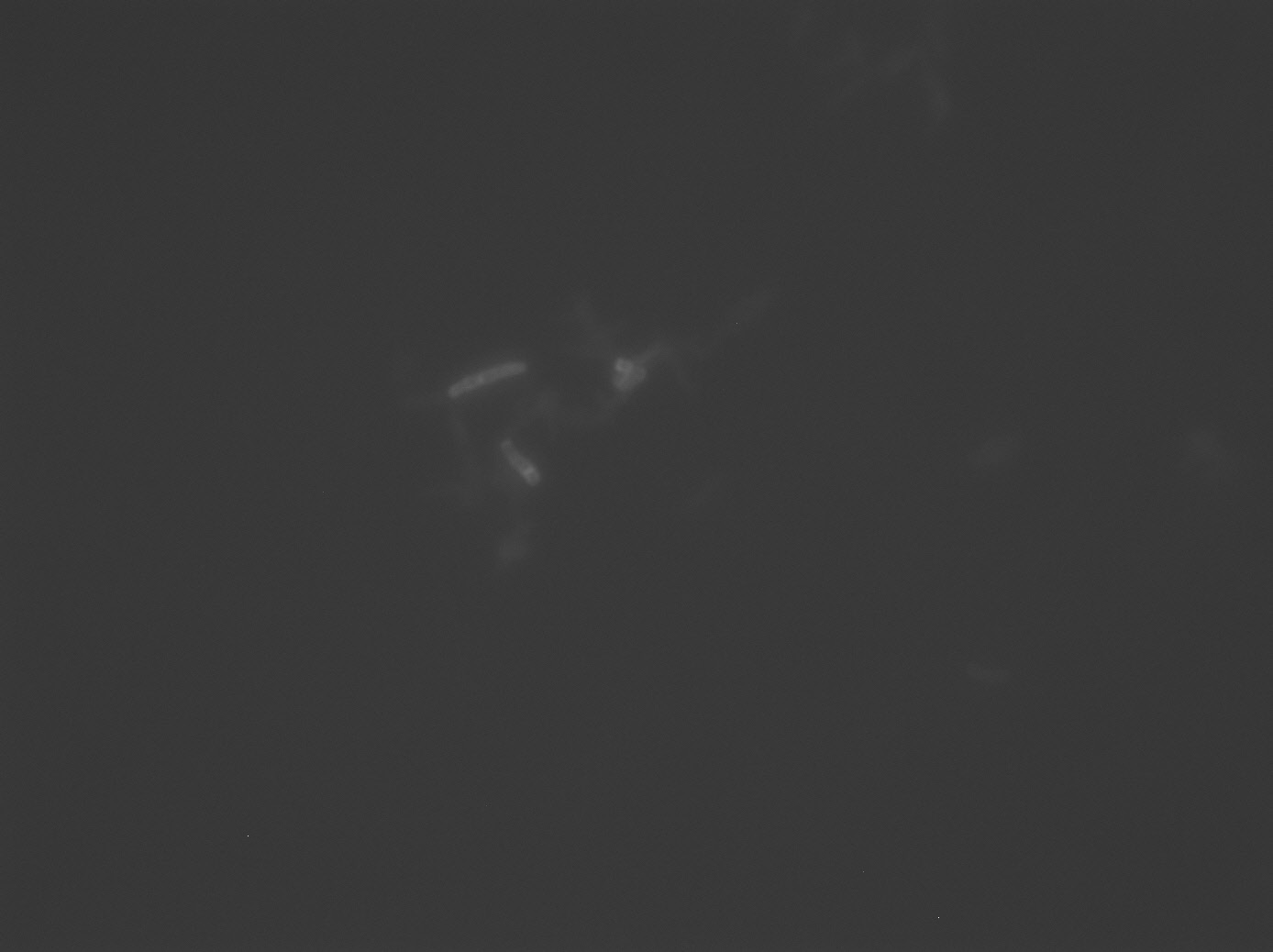

Supplement: Figure 2—figure supplement 2—source data 1. [file elife-37243-fig2-figsupp2-data1.zip › Figure 2--figure supplement 2/Figure 2--figure supplement 2C/OalkTMM/GFP 6.jpg]

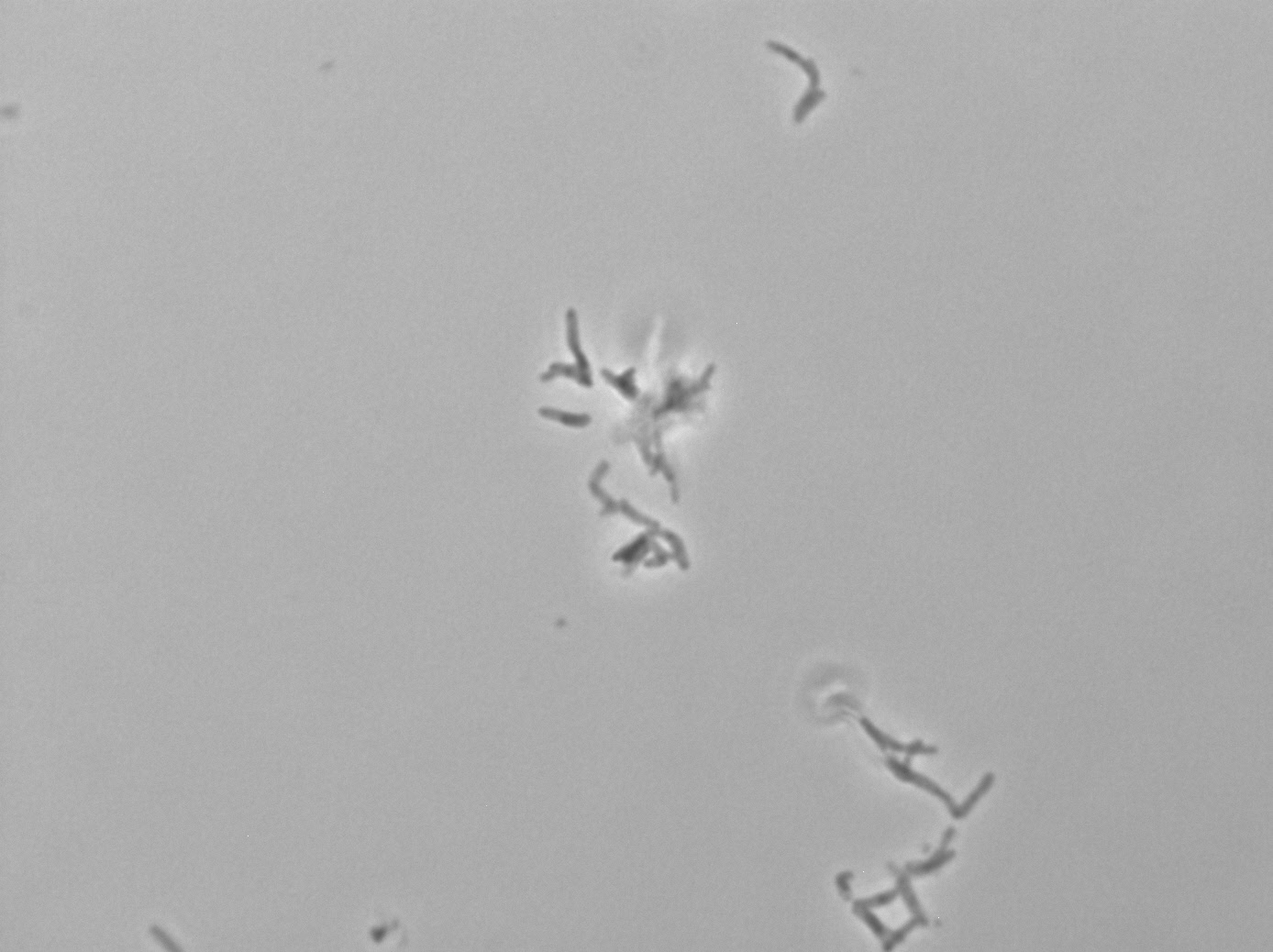

Supplement: Figure 2—figure supplement 2—source data 1. [file elife-37243-fig2-figsupp2-data1.zip › Figure 2--figure supplement 2/Figure 2--figure supplement 2C/OalkTMM/PC 1.jpg]

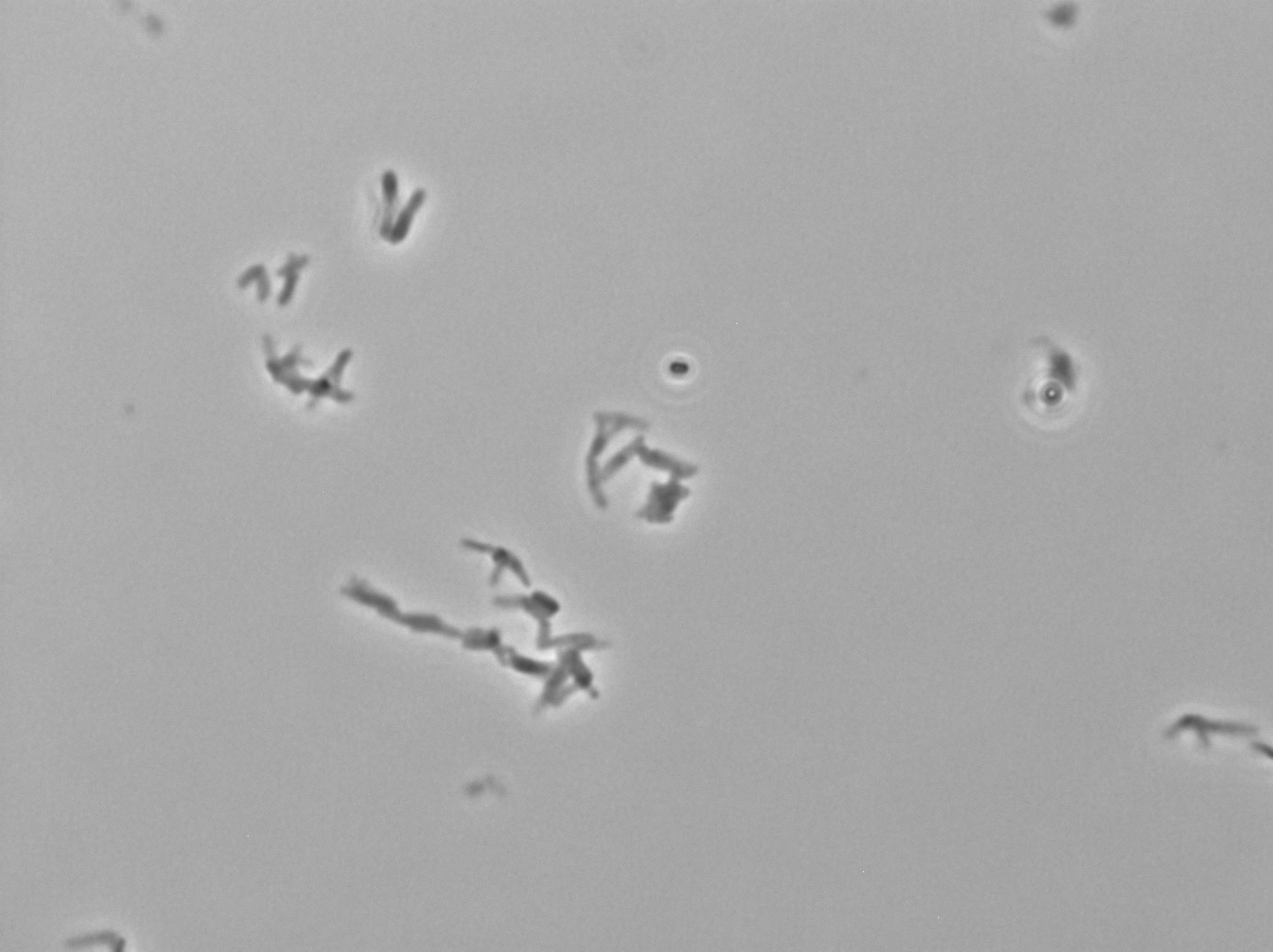

Supplement: Figure 2—figure supplement 2—source data 1. [file elife-37243-fig2-figsupp2-data1.zip › Figure 2--figure supplement 2/Figure 2--figure supplement 2C/OalkTMM/PC 10.jpg]

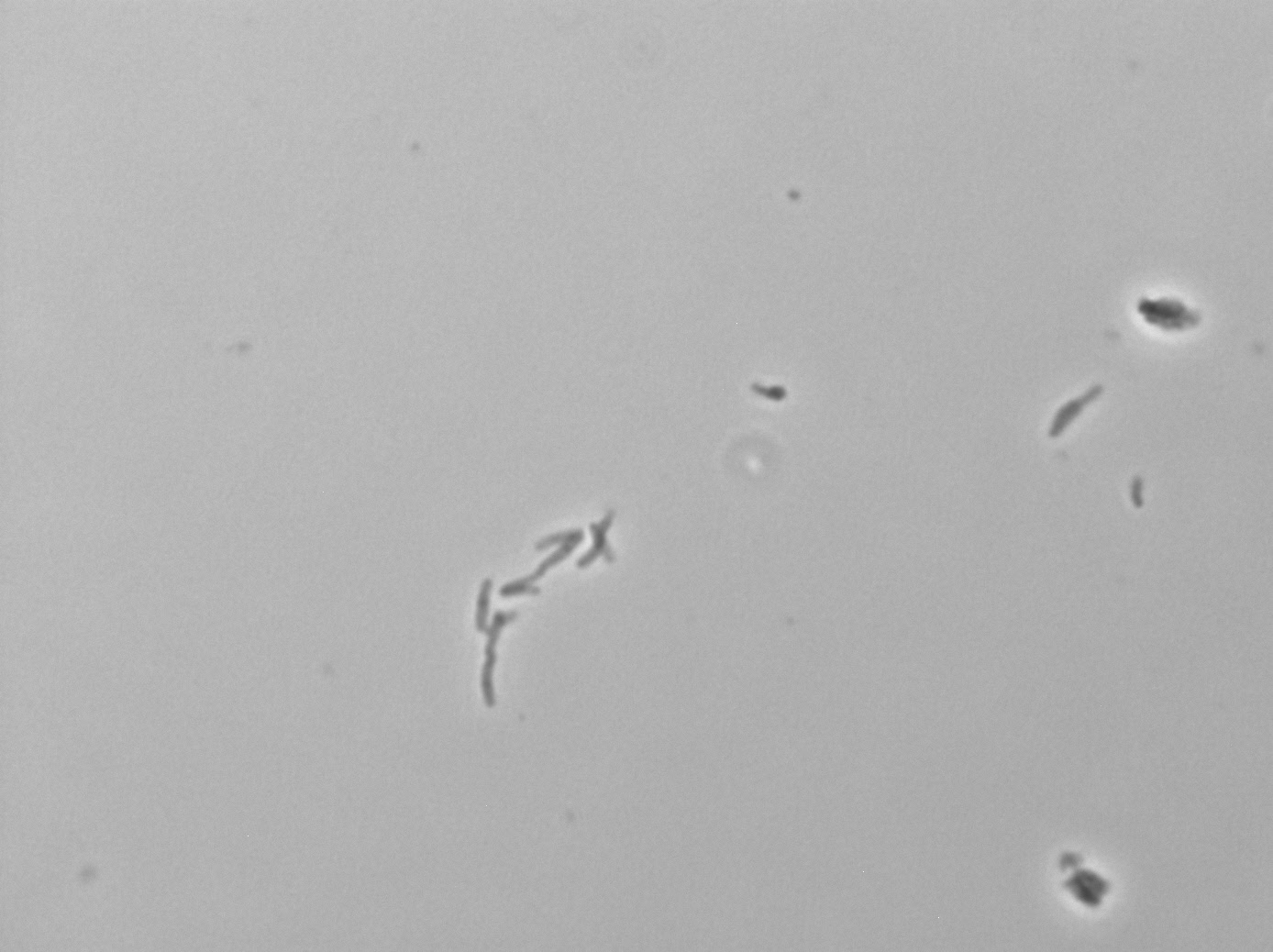

Supplement: Figure 2—figure supplement 2—source data 1. [file elife-37243-fig2-figsupp2-data1.zip › Figure 2--figure supplement 2/Figure 2--figure supplement 2C/OalkTMM/PC 2.jpg]

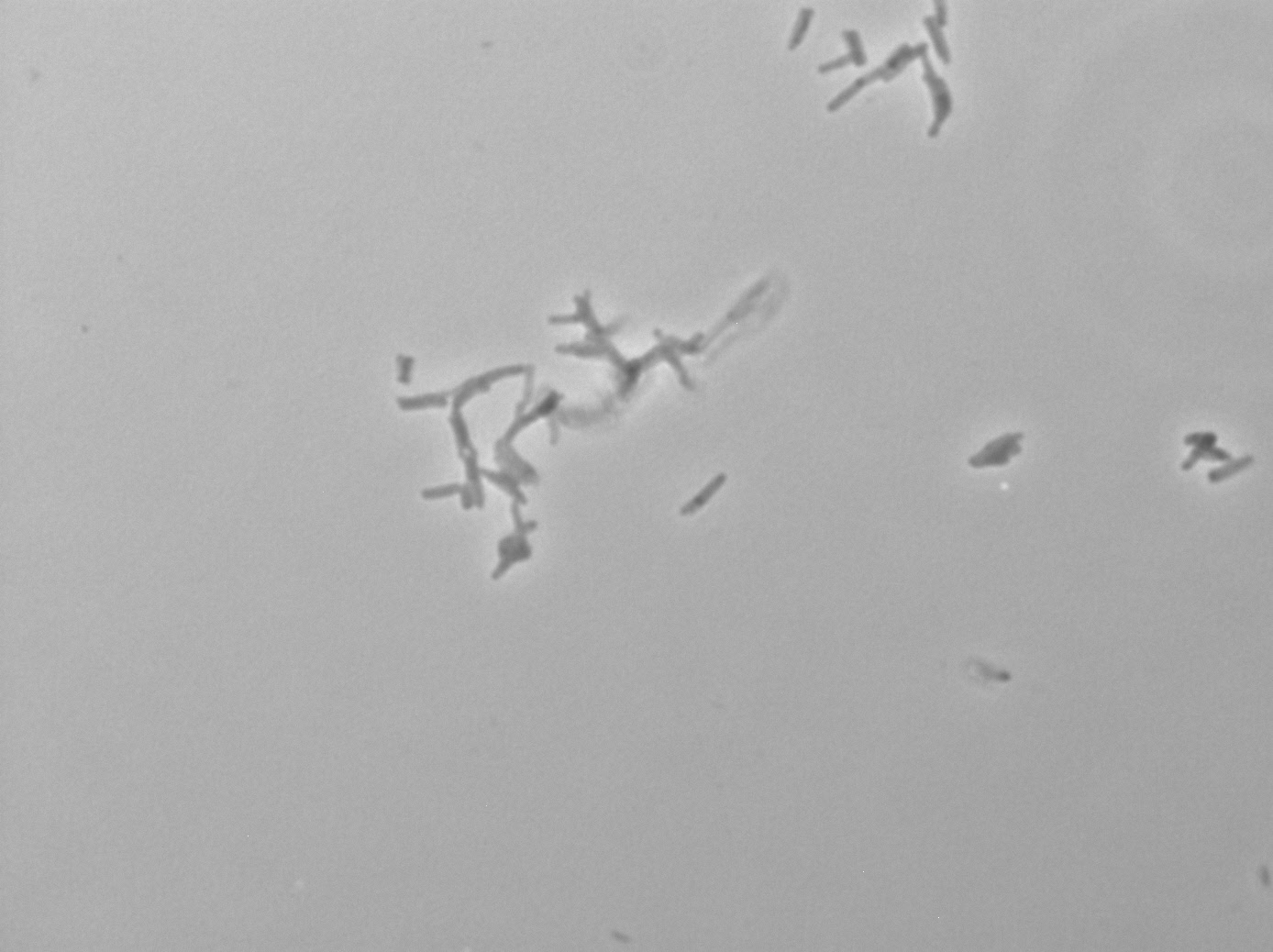

Supplement: Figure 2—figure supplement 2—source data 1. [file elife-37243-fig2-figsupp2-data1.zip › Figure 2--figure supplement 2/Figure 2--figure supplement 2C/OalkTMM/PC 6.jpg]

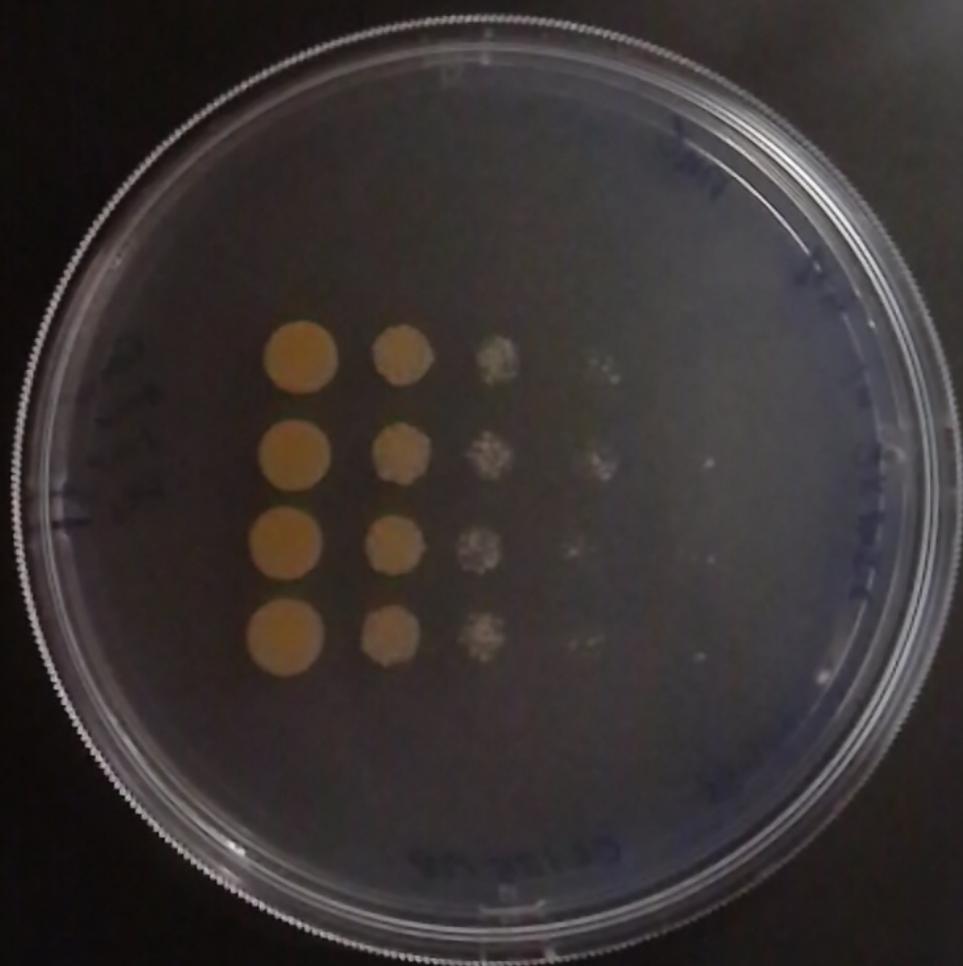

Supplement: Figure 3—figure supplement 2—source data 1. [file elife-37243-fig3-figsupp2-data1.pdf]

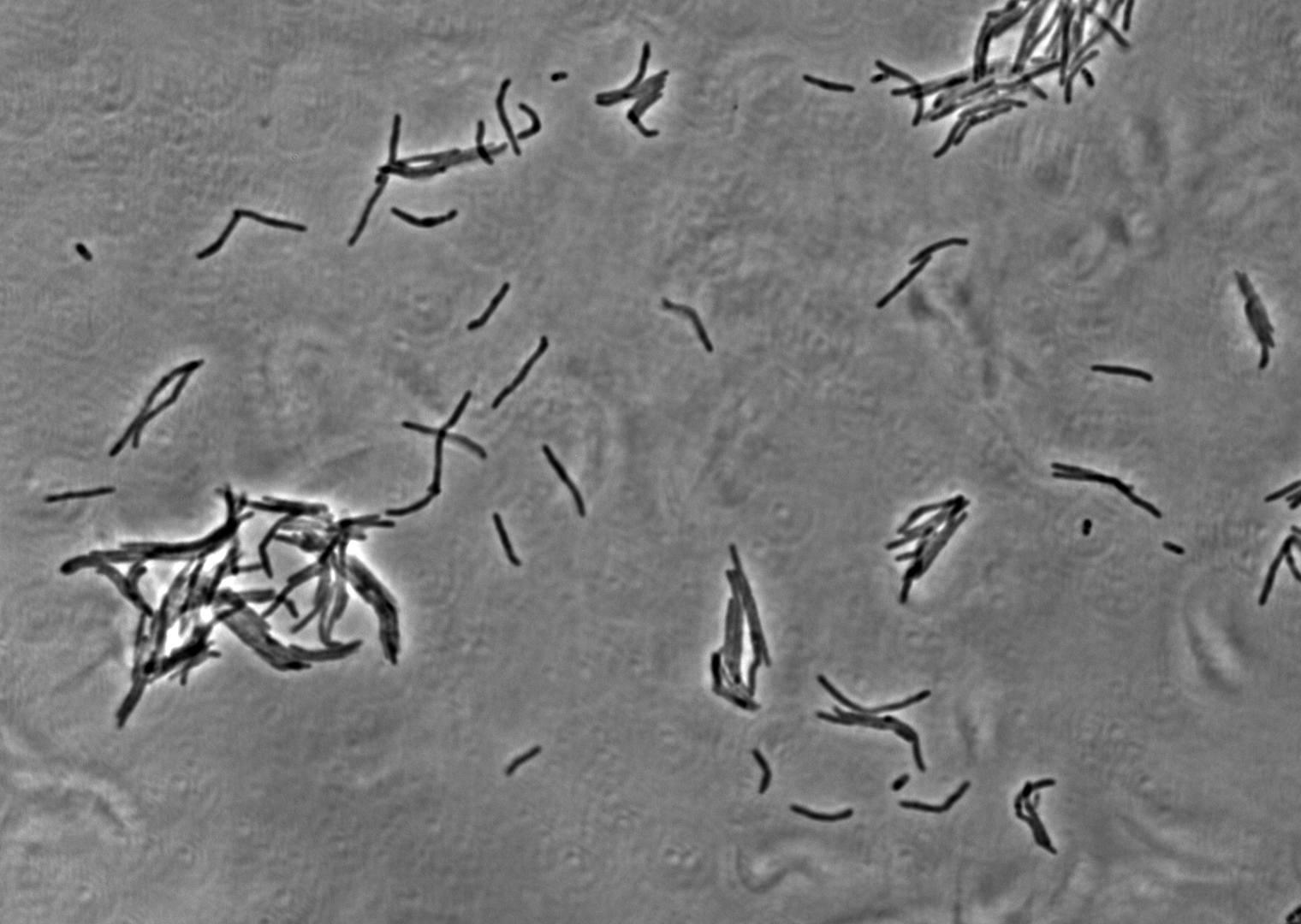

Supplement: Figure 4—figure supplement 2—source data 1. [file elife-37243-fig4-figsupp2-data1.zip › Figure 4-supplement 2 source data/Figure 4ΓÇöfigure supplement 2 (B)/1. wt no abx edada/1. PC/1.tif]

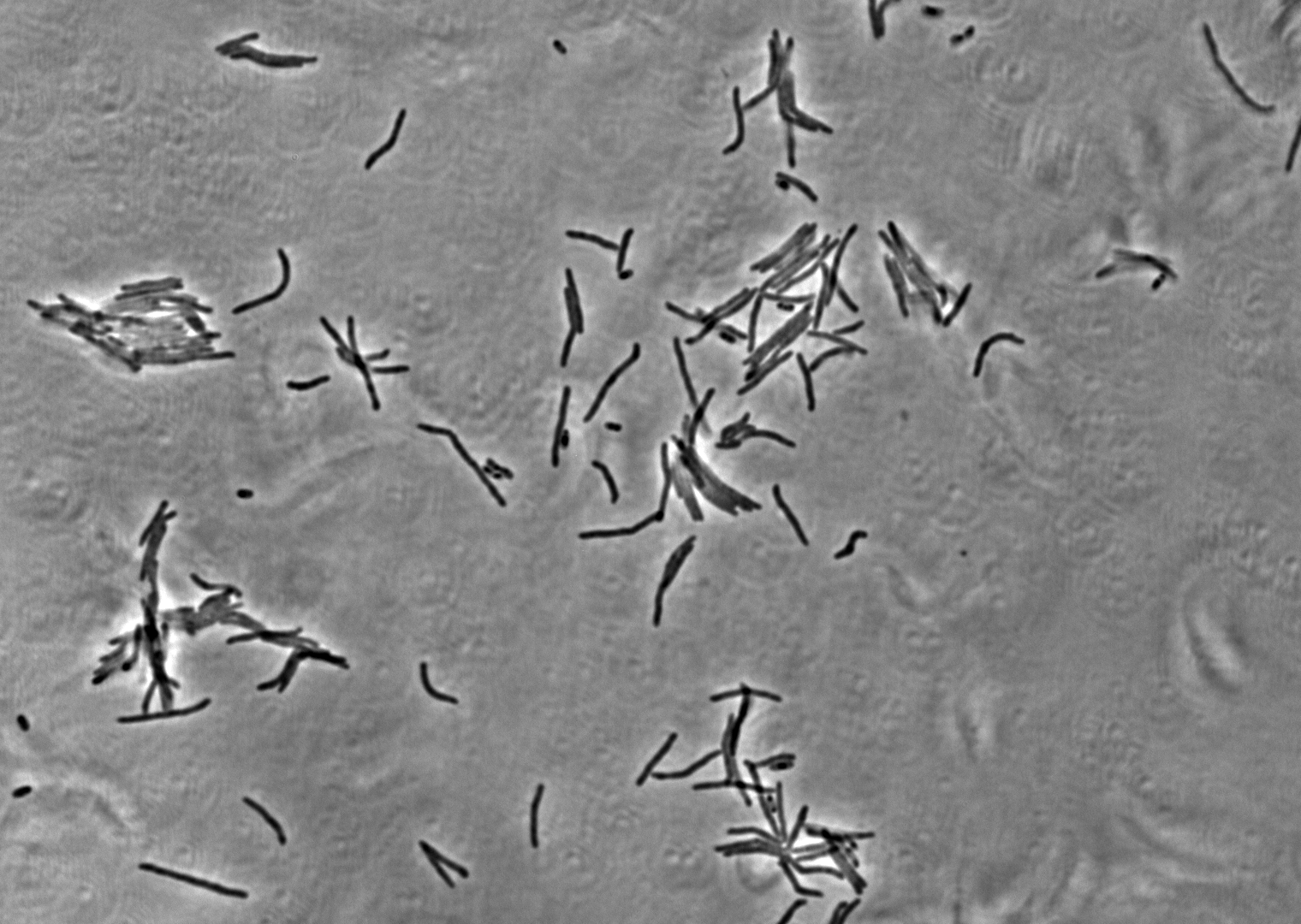

Supplement: Figure 4—figure supplement 2—source data 1. [file elife-37243-fig4-figsupp2-data1.zip › Figure 4-supplement 2 source data/Figure 4ΓÇöfigure supplement 2 (B)/1. wt no abx edada/1. PC/11.tif]

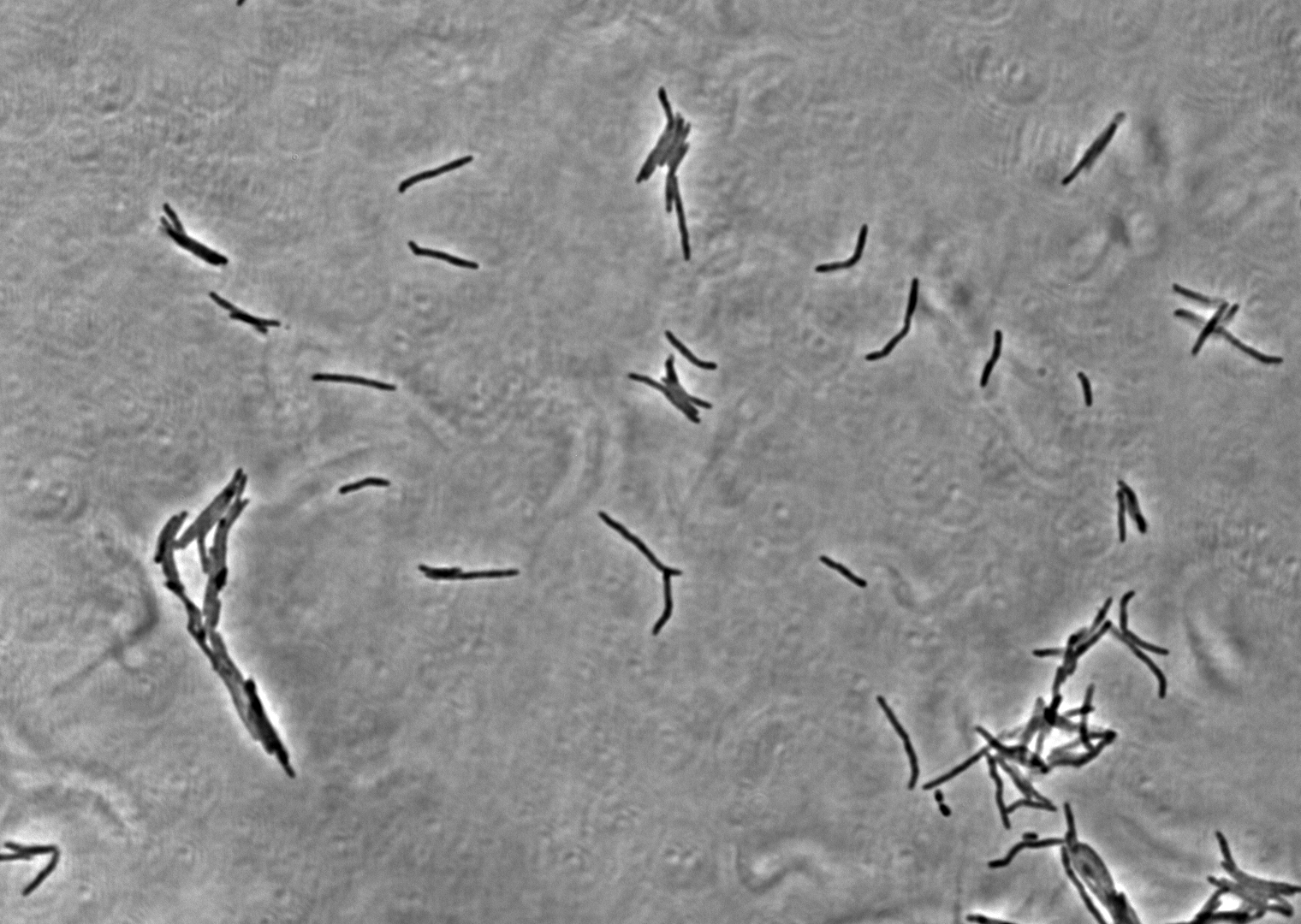

Supplement: Figure 4—figure supplement 2—source data 1. [file elife-37243-fig4-figsupp2-data1.zip › Figure 4-supplement 2 source data/Figure 4ΓÇöfigure supplement 2 (B)/1. wt no abx edada/1. PC/4.tif]

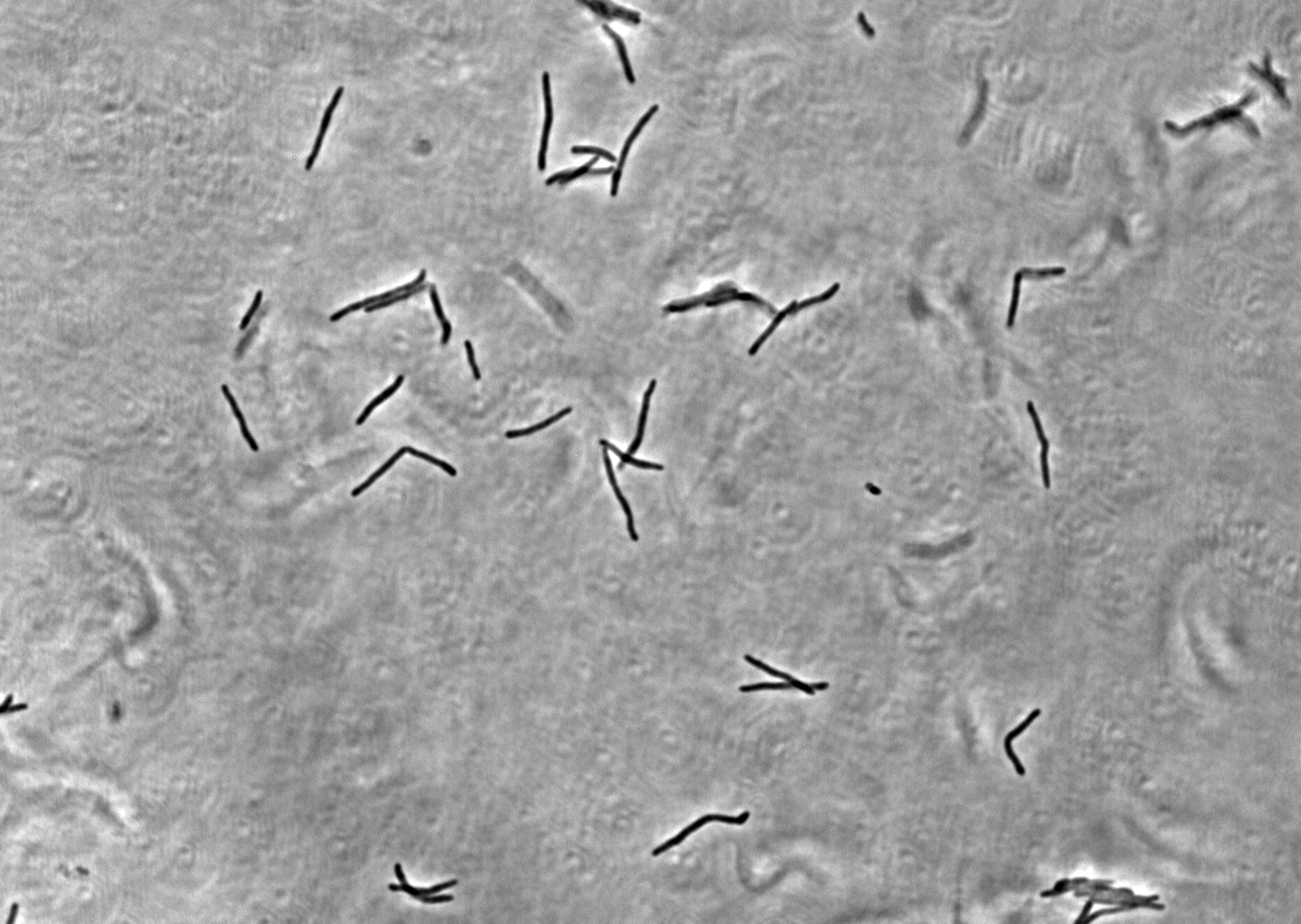

Supplement: Figure 4—figure supplement 2—source data 1. [file elife-37243-fig4-figsupp2-data1.zip › Figure 4-supplement 2 source data/Figure 4ΓÇöfigure supplement 2 (B)/1. wt no abx edada/1. PC/5.tif]

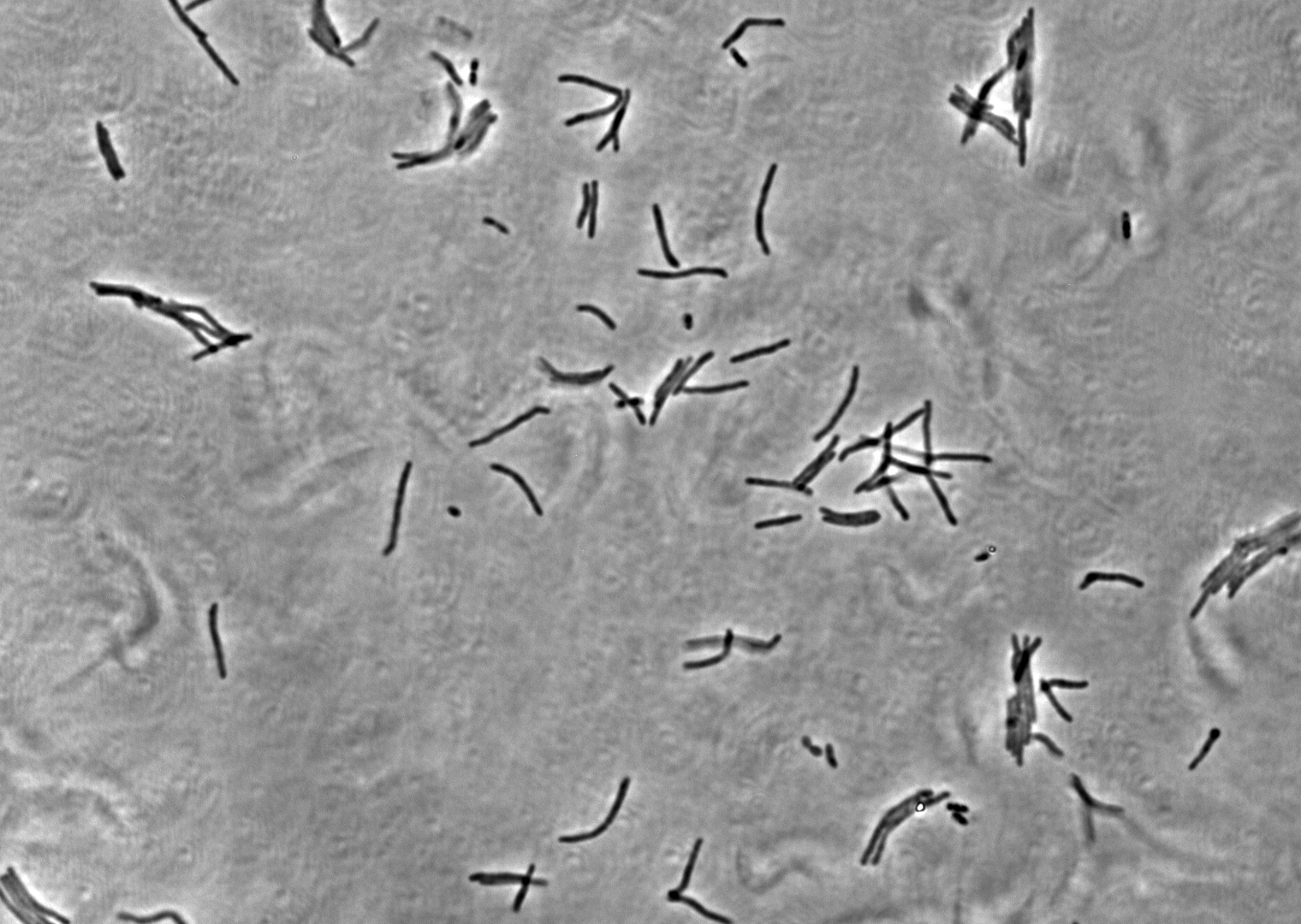

Supplement: Figure 4—figure supplement 2—source data 1. [file elife-37243-fig4-figsupp2-data1.zip › Figure 4-supplement 2 source data/Figure 4ΓÇöfigure supplement 2 (B)/1. wt no abx edada/1. PC/6.tif]

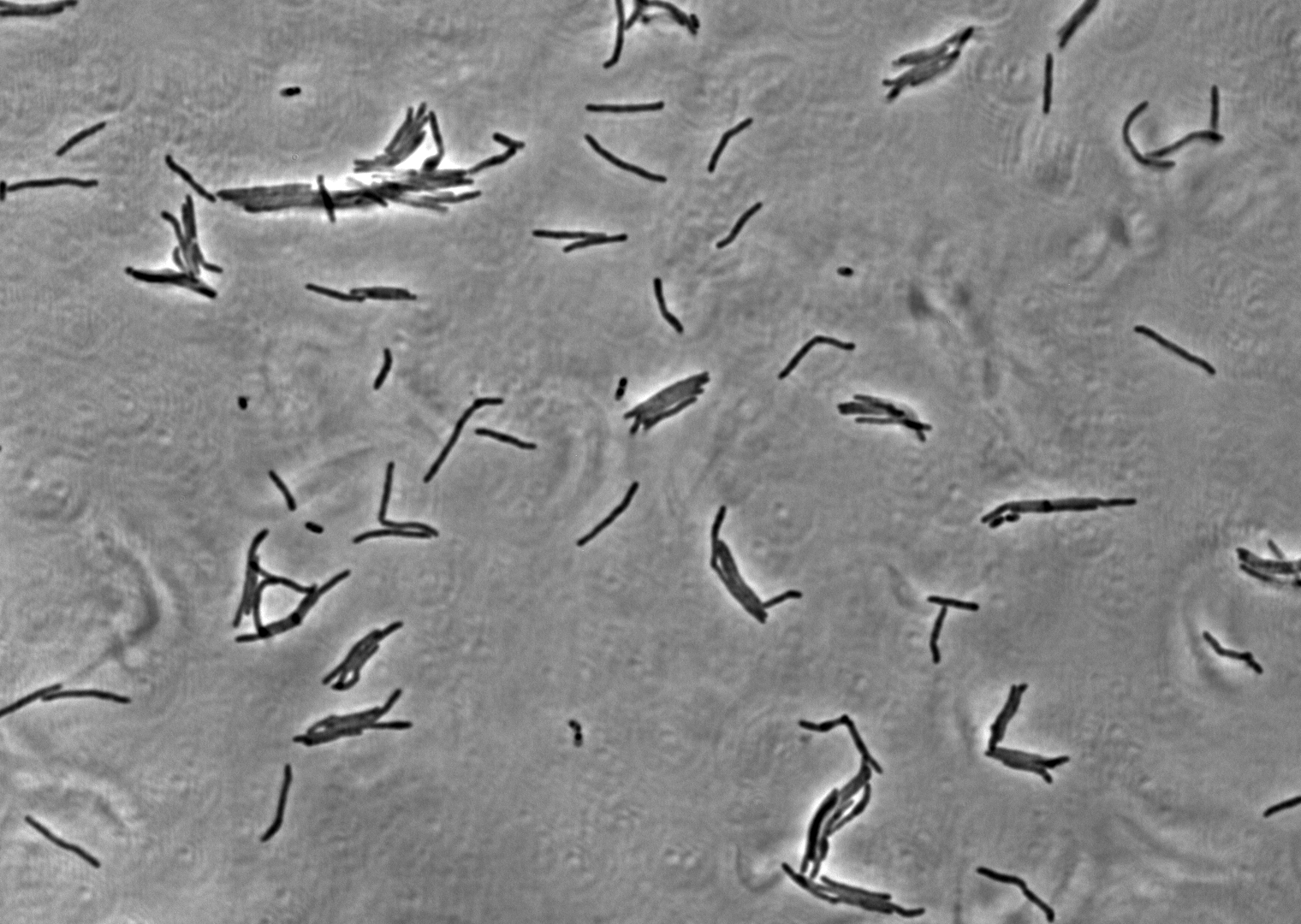

Supplement: Figure 4—figure supplement 2—source data 1. [file elife-37243-fig4-figsupp2-data1.zip › Figure 4-supplement 2 source data/Figure 4ΓÇöfigure supplement 2 (B)/1. wt no abx edada/1. PC/7.tif]

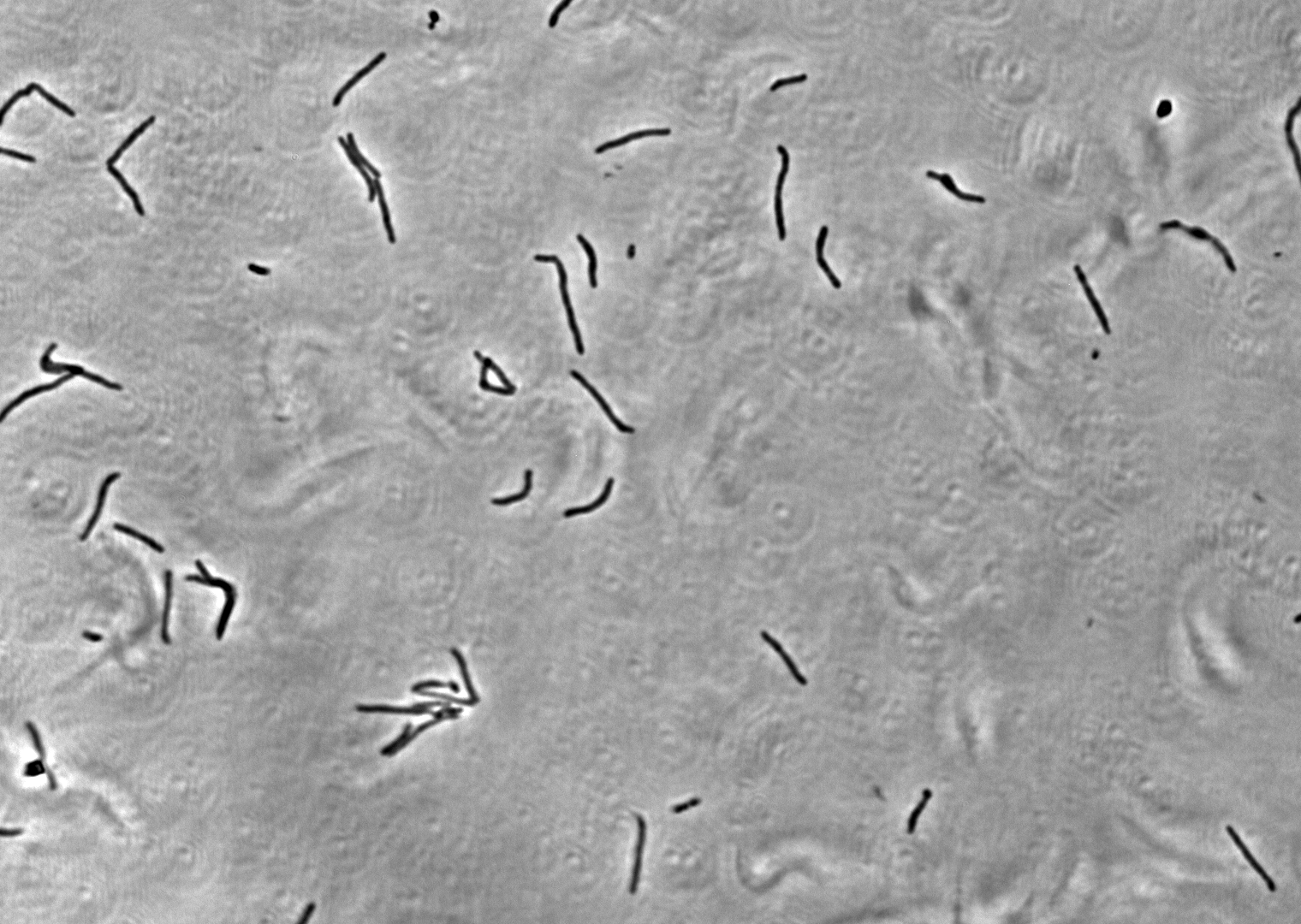

Supplement: Figure 4—figure supplement 2—source data 1. [file elife-37243-fig4-figsupp2-data1.zip › Figure 4-supplement 2 source data/Figure 4ΓÇöfigure supplement 2 (B)/1. wt no abx edada/1. PC/8.tif]

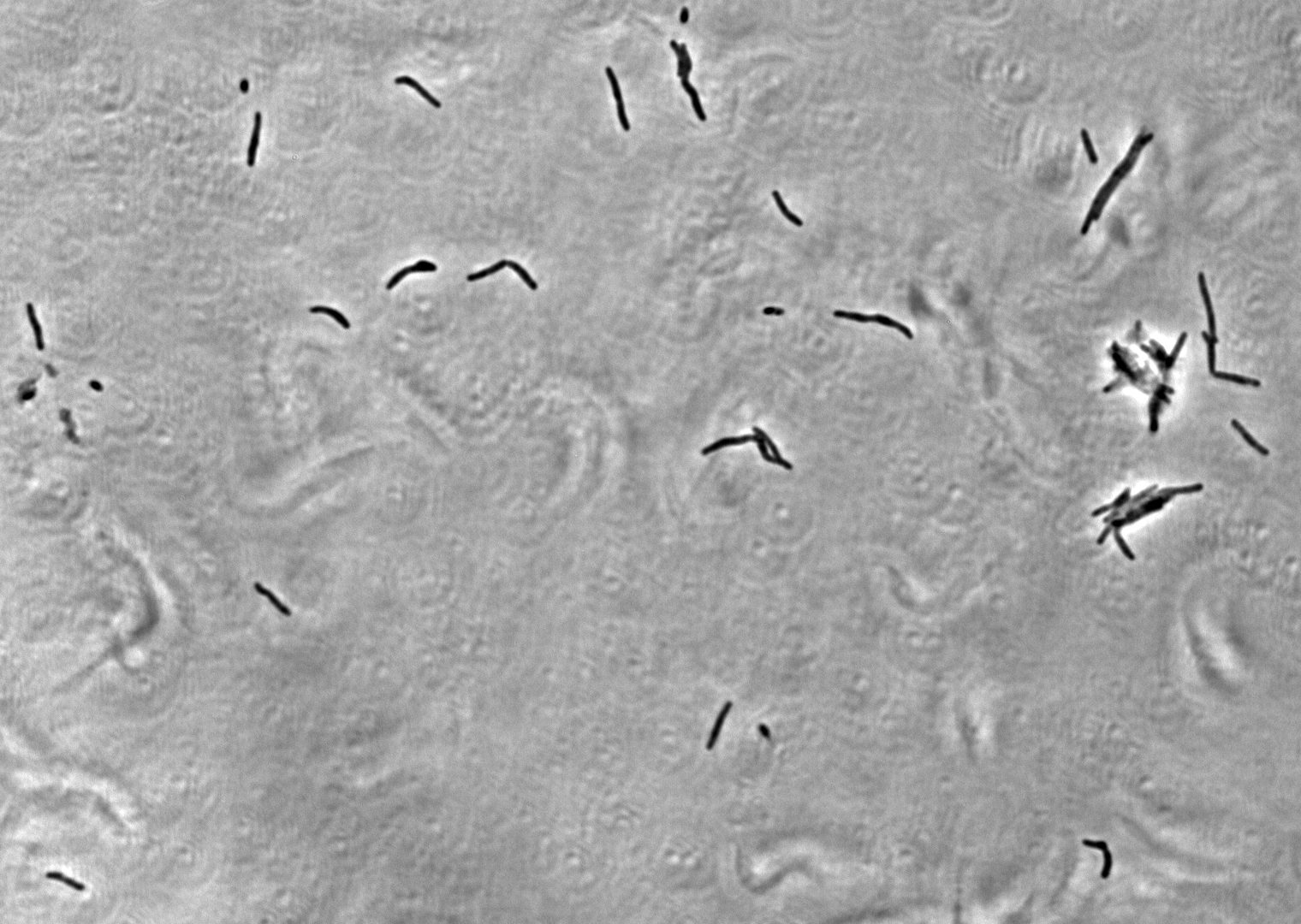

Supplement: Figure 4—figure supplement 2—source data 1. [file elife-37243-fig4-figsupp2-data1.zip › Figure 4-supplement 2 source data/Figure 4ΓÇöfigure supplement 2 (B)/2. ldts no abx/1. PC/1.tif]

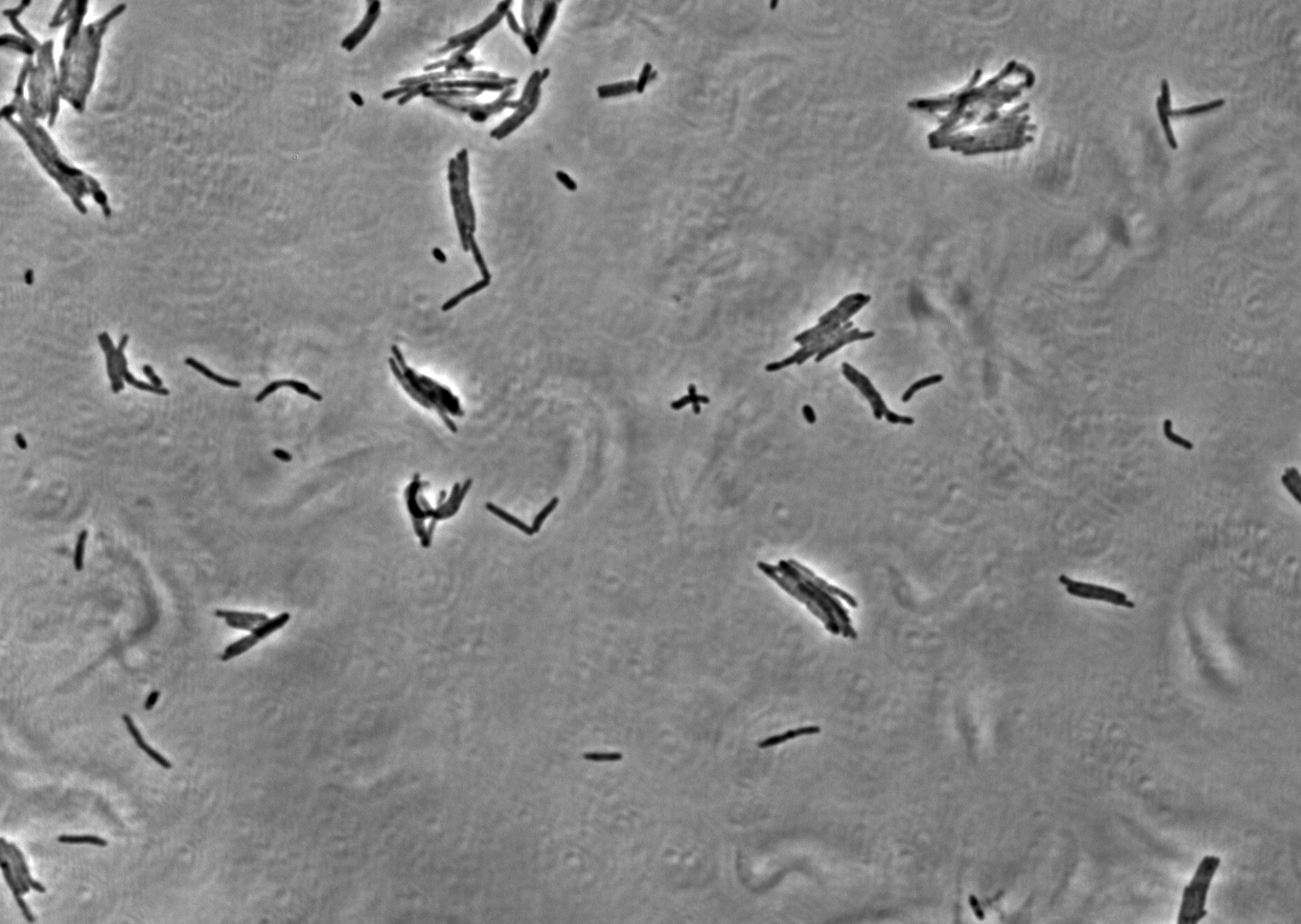

Supplement: Figure 4—figure supplement 2—source data 1. [file elife-37243-fig4-figsupp2-data1.zip › Figure 4-supplement 2 source data/Figure 4ΓÇöfigure supplement 2 (B)/2. ldts no abx/1. PC/2.tif]

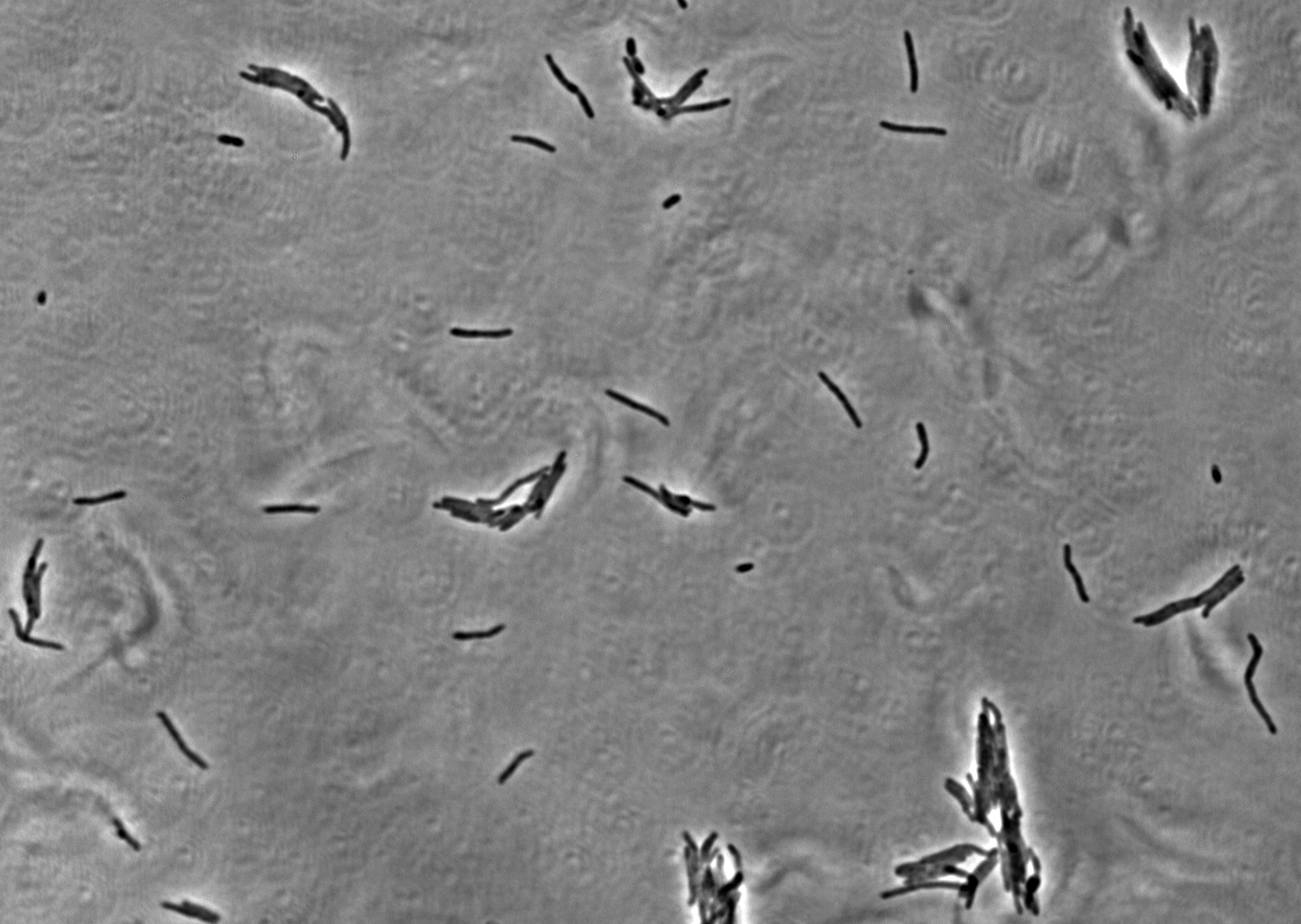

Supplement: Figure 4—figure supplement 2—source data 1. [file elife-37243-fig4-figsupp2-data1.zip › Figure 4-supplement 2 source data/Figure 4ΓÇöfigure supplement 2 (B)/2. ldts no abx/1. PC/3.tif]

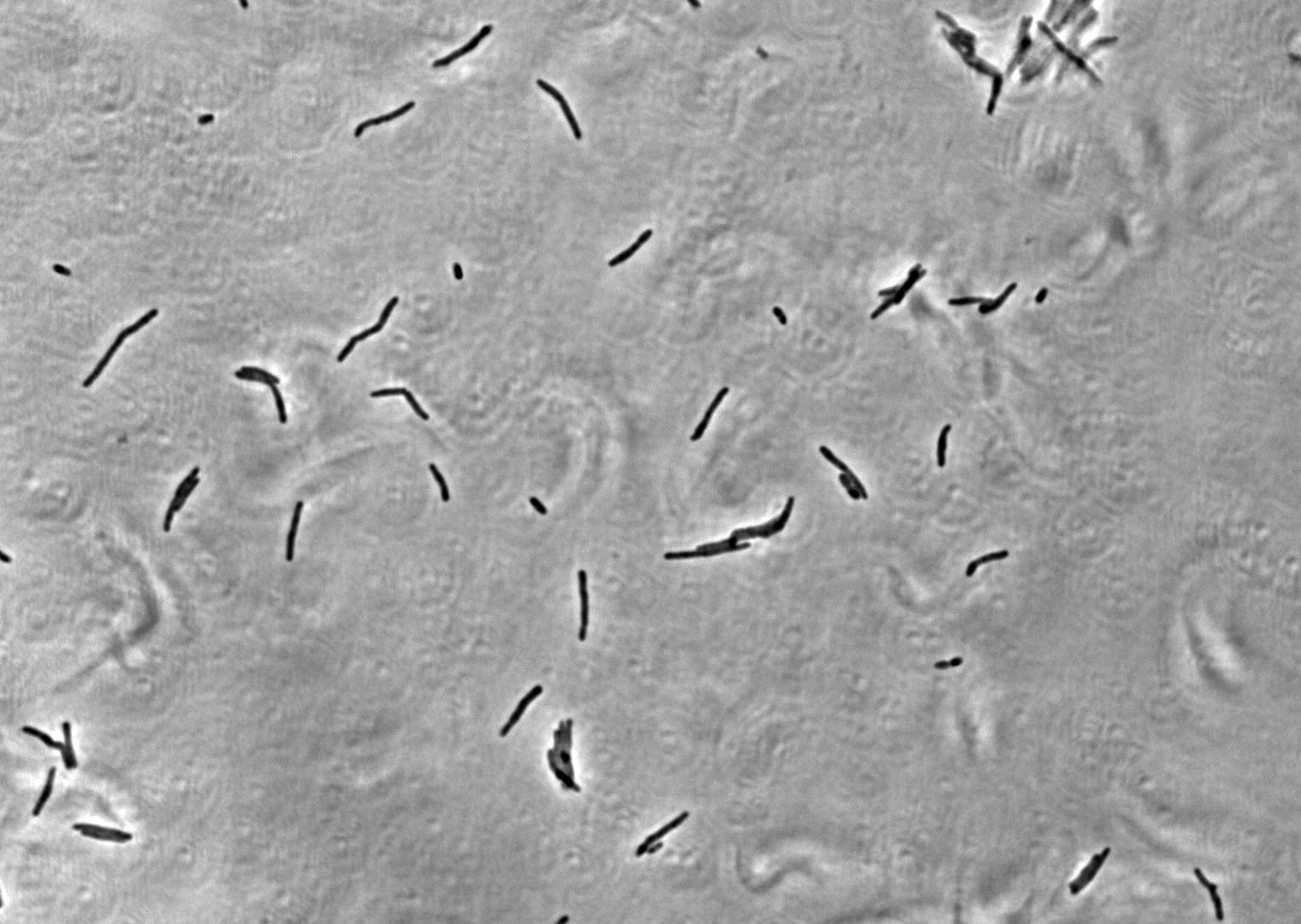

Supplement: Figure 4—figure supplement 2—source data 1. [file elife-37243-fig4-figsupp2-data1.zip › Figure 4-supplement 2 source data/Figure 4ΓÇöfigure supplement 2 (B)/2. ldts no abx/1. PC/4.tif]

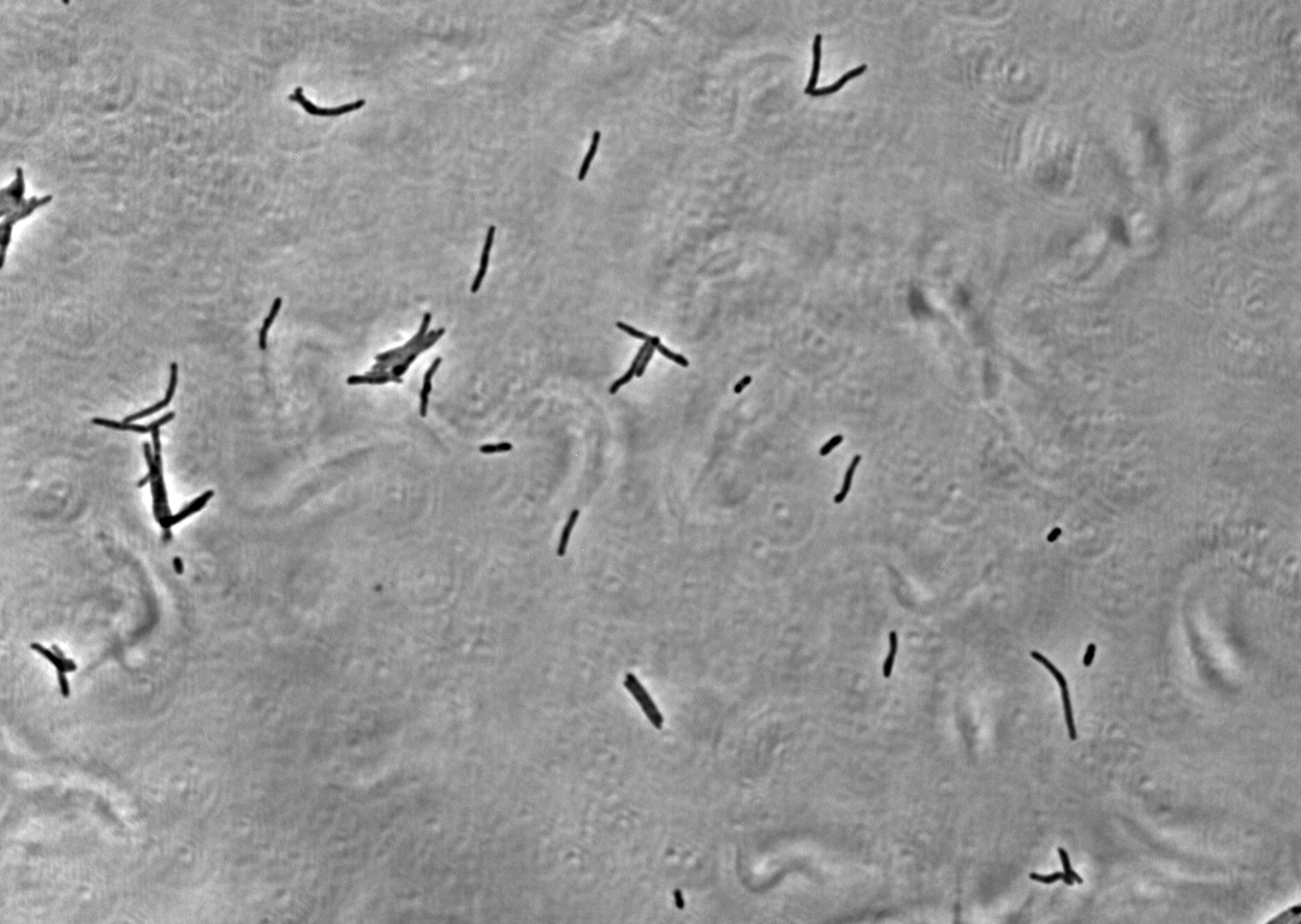

Supplement: Figure 4—figure supplement 2—source data 1. [file elife-37243-fig4-figsupp2-data1.zip › Figure 4-supplement 2 source data/Figure 4ΓÇöfigure supplement 2 (B)/2. ldts no abx/1. PC/5.tif]

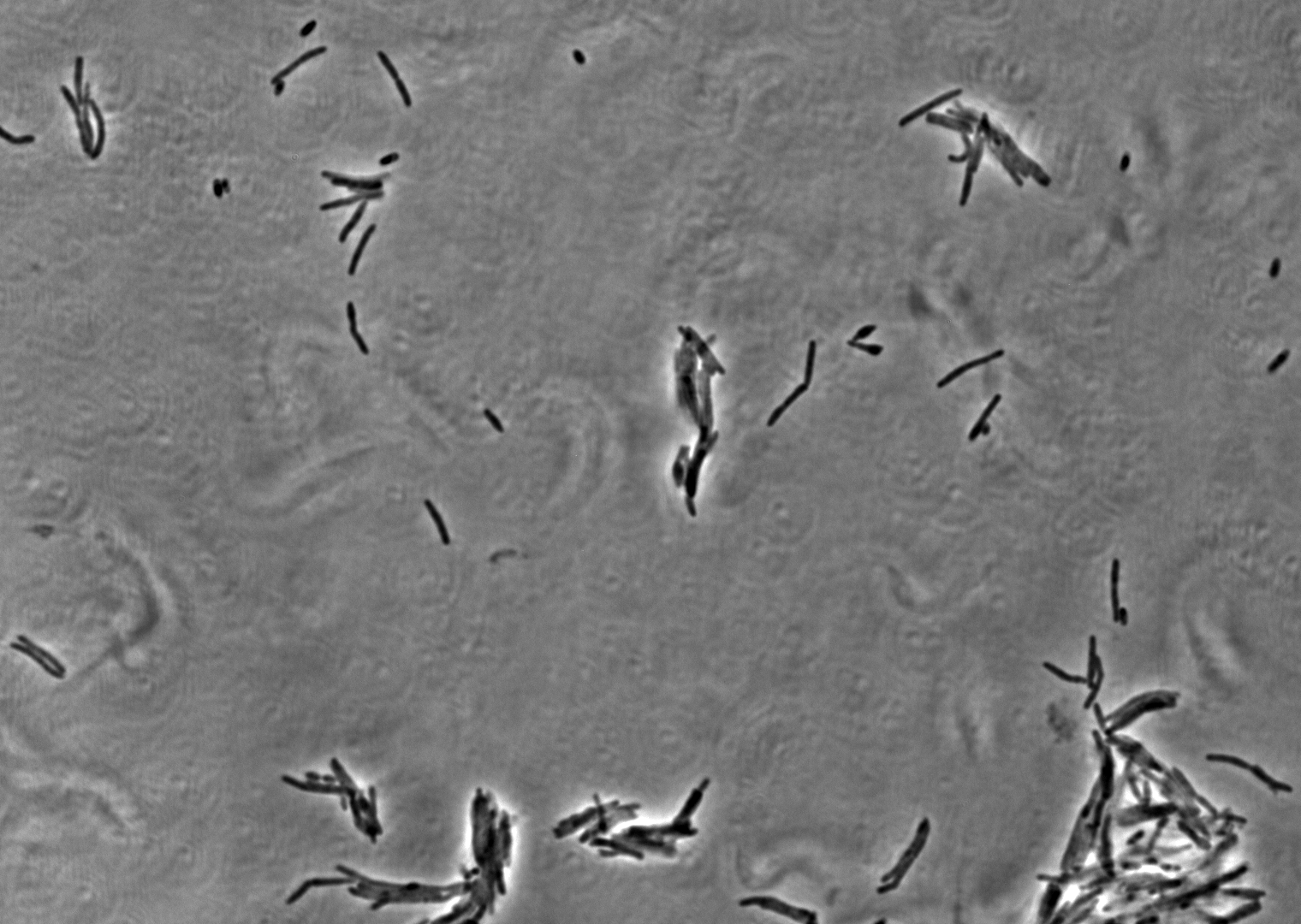

Supplement: Figure 4—figure supplement 2—source data 1. [file elife-37243-fig4-figsupp2-data1.zip › Figure 4-supplement 2 source data/Figure 4ΓÇöfigure supplement 2 (B)/2. ldts no abx/1. PC/6.tif]

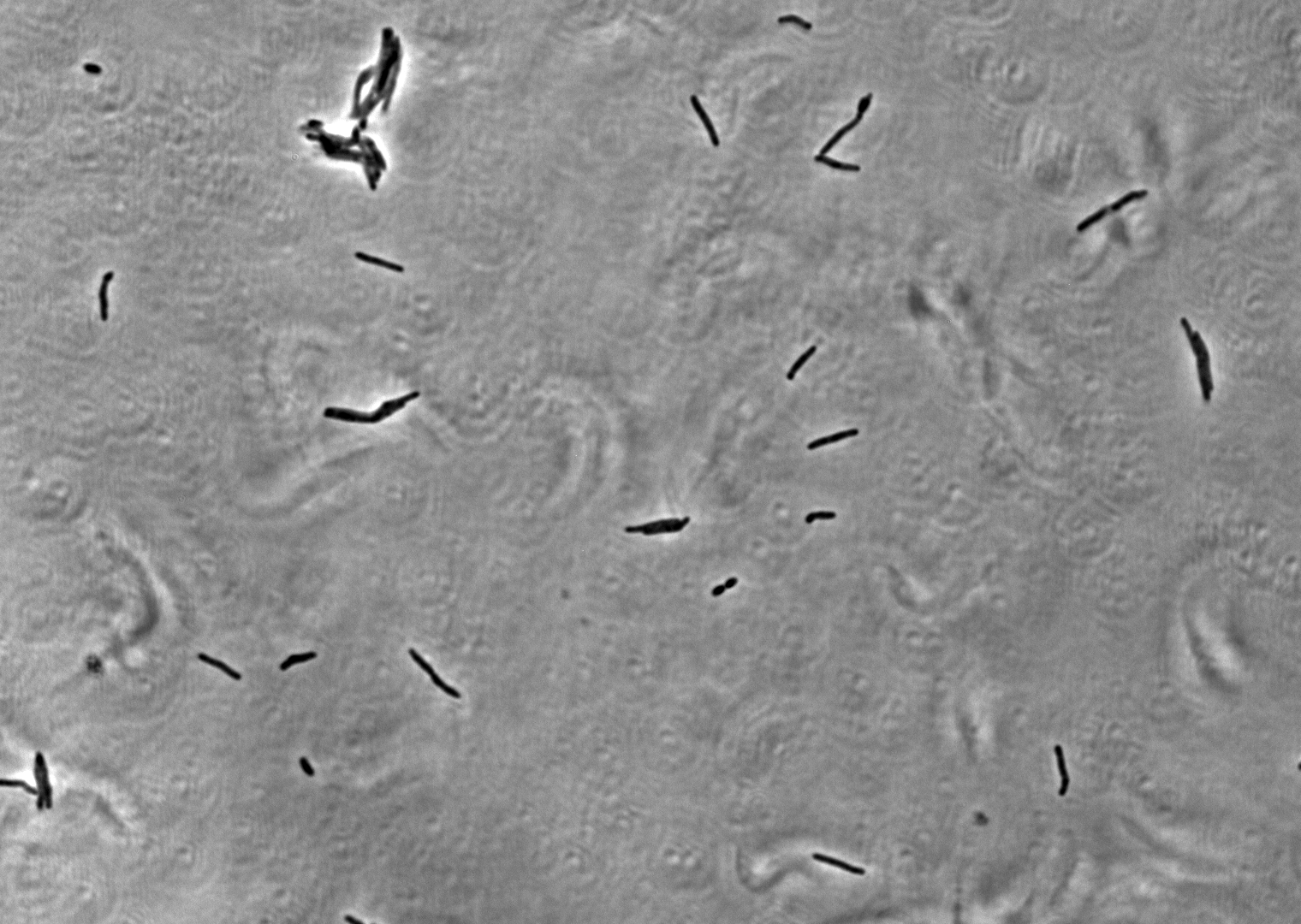

Supplement: Figure 4—figure supplement 2—source data 1. [file elife-37243-fig4-figsupp2-data1.zip › Figure 4-supplement 2 source data/Figure 4ΓÇöfigure supplement 2 (B)/2. ldts no abx/1. PC/7.tif]

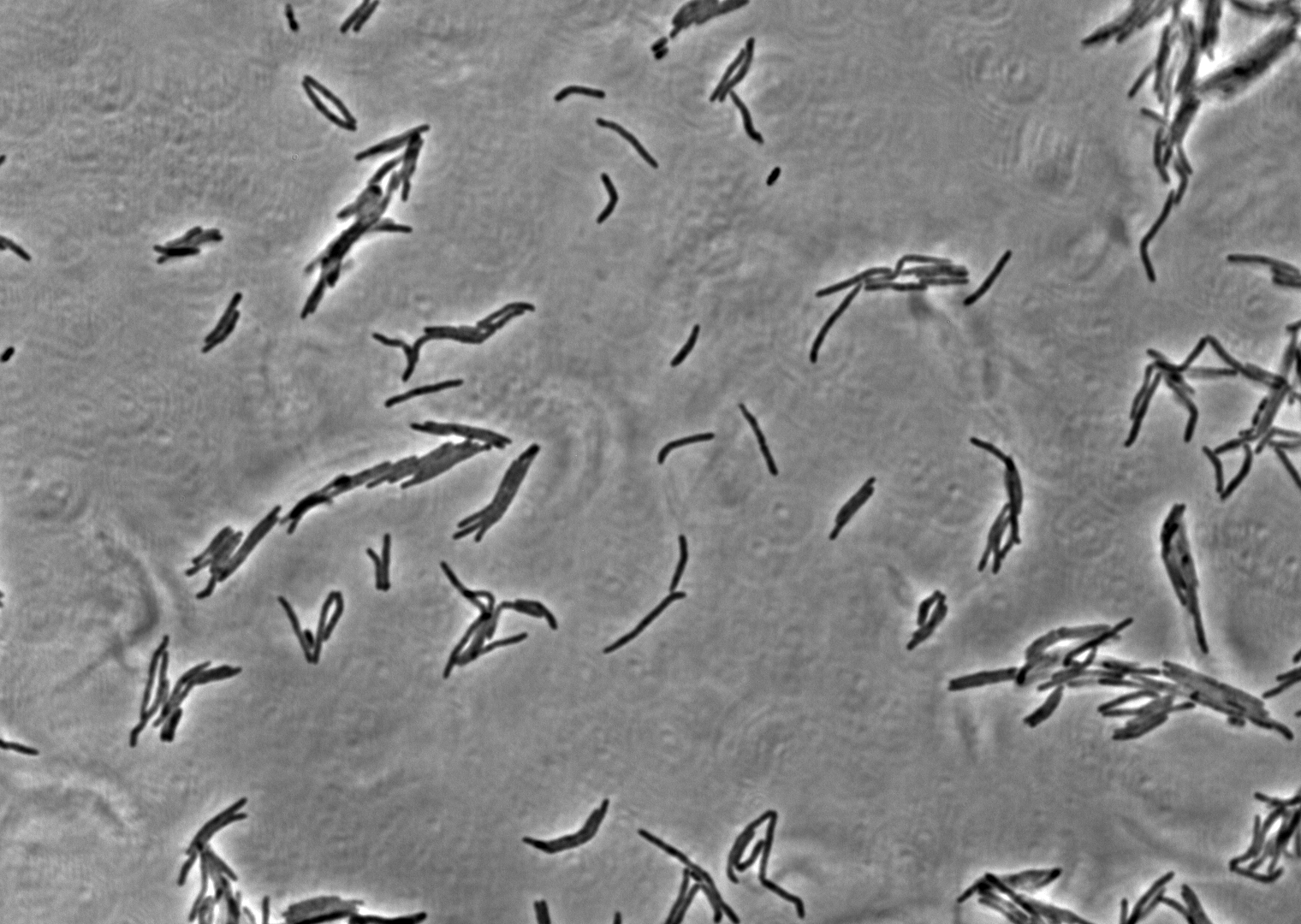

Supplement: Figure 4—figure supplement 2—source data 1. [file elife-37243-fig4-figsupp2-data1.zip › Figure 4-supplement 2 source data/Figure 4ΓÇöfigure supplement 2 (B)/3. wt no probe/1. PC/Image1.tif]

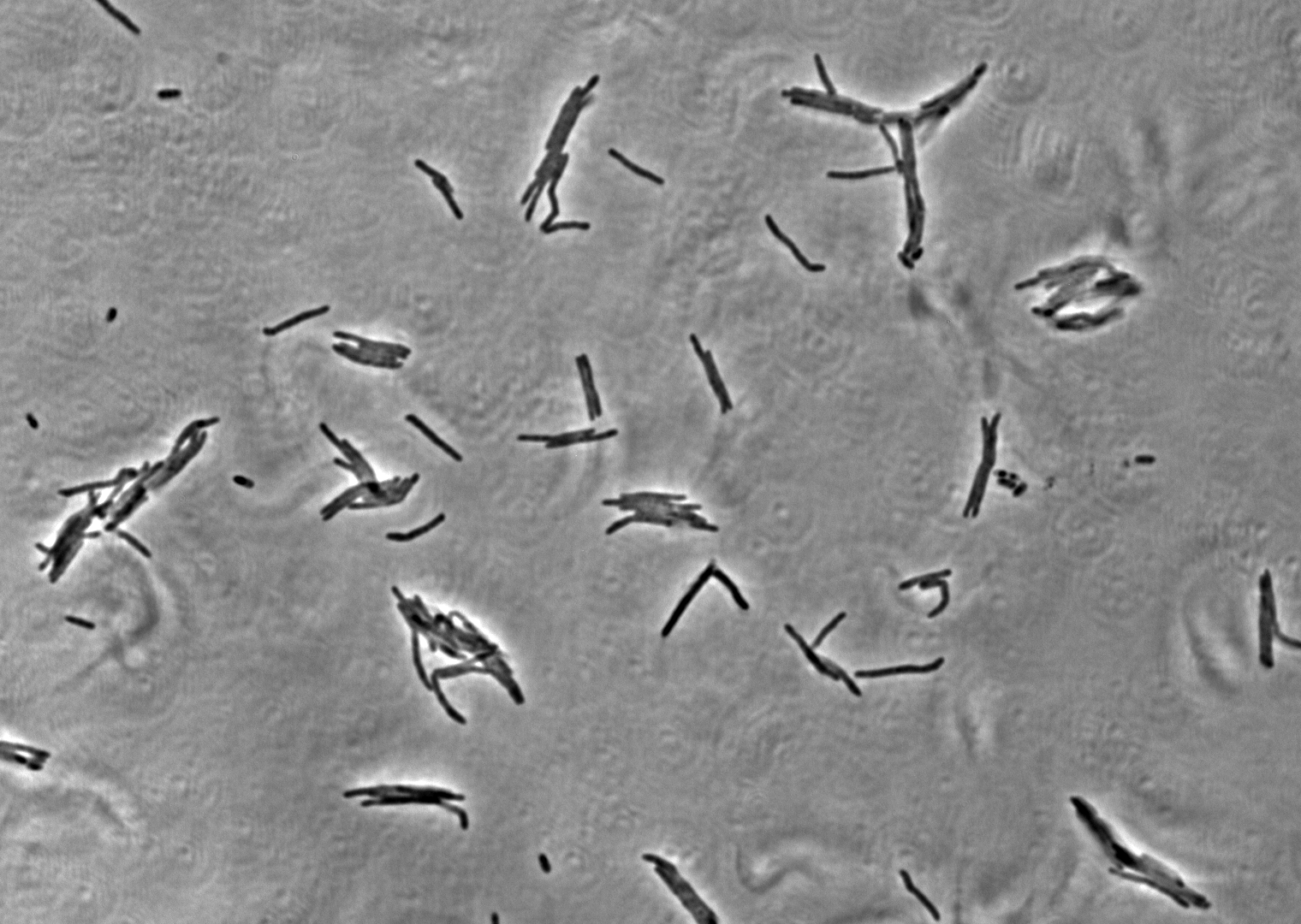

Supplement: Figure 4—figure supplement 2—source data 1. [file elife-37243-fig4-figsupp2-data1.zip › Figure 4-supplement 2 source data/Figure 4ΓÇöfigure supplement 2 (B)/3. wt no probe/1. PC/Image3.tif]

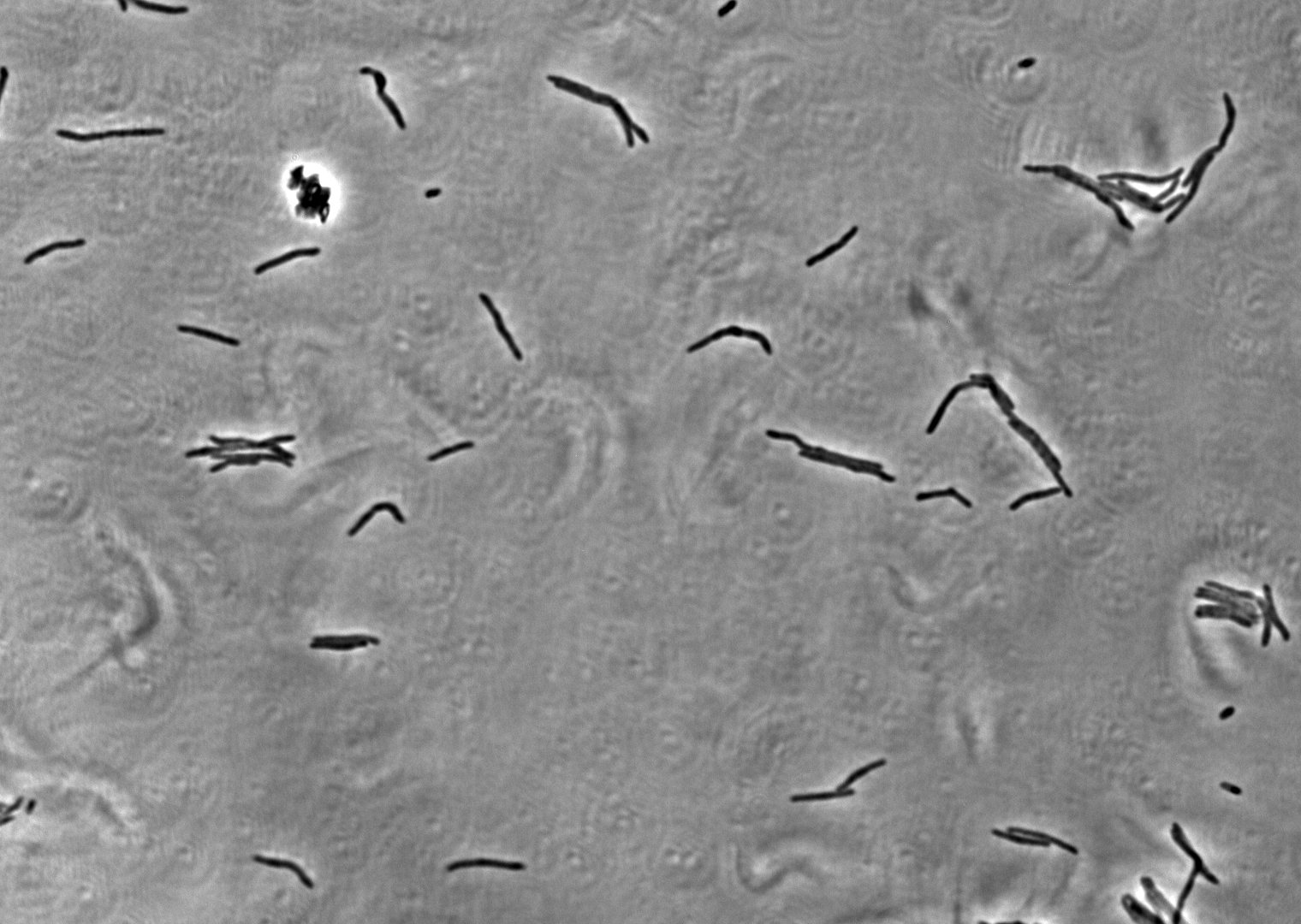

Supplement: Figure 4—figure supplement 2—source data 1. [file elife-37243-fig4-figsupp2-data1.zip › Figure 4-supplement 2 source data/Figure 4ΓÇöfigure supplement 2 (B)/3. wt no probe/1. PC/Image5.tif]

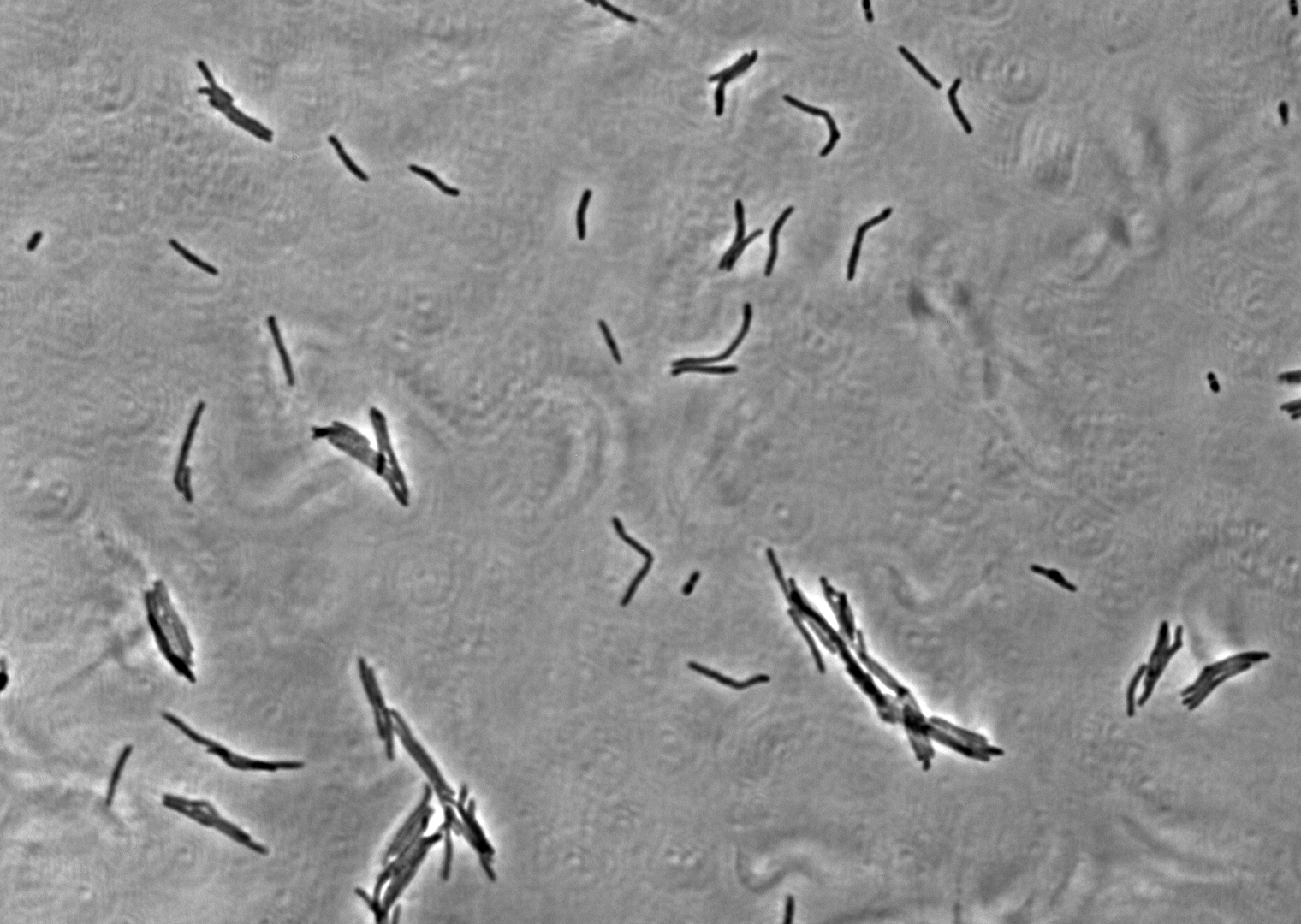

Supplement: Figure 4—figure supplement 2—source data 1. [file elife-37243-fig4-figsupp2-data1.zip › Figure 4-supplement 2 source data/Figure 4ΓÇöfigure supplement 2 (B)/3. wt no probe/1. PC/Image7.tif]

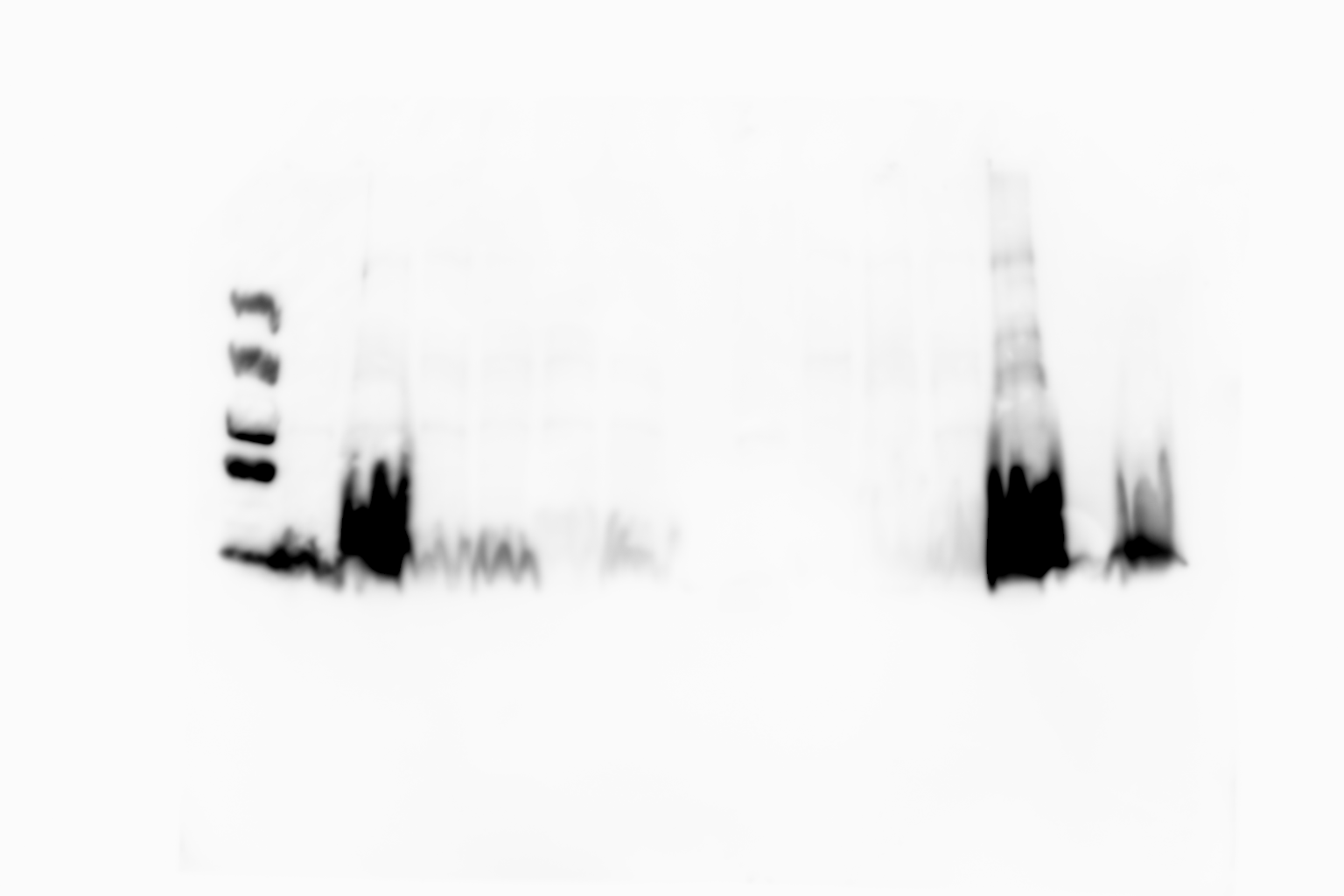

Supplement: Figure 5—source data 1. [file elife-37243-fig5-data1.zip › Figure 5 source data/1. Lipid II control blot.tif]

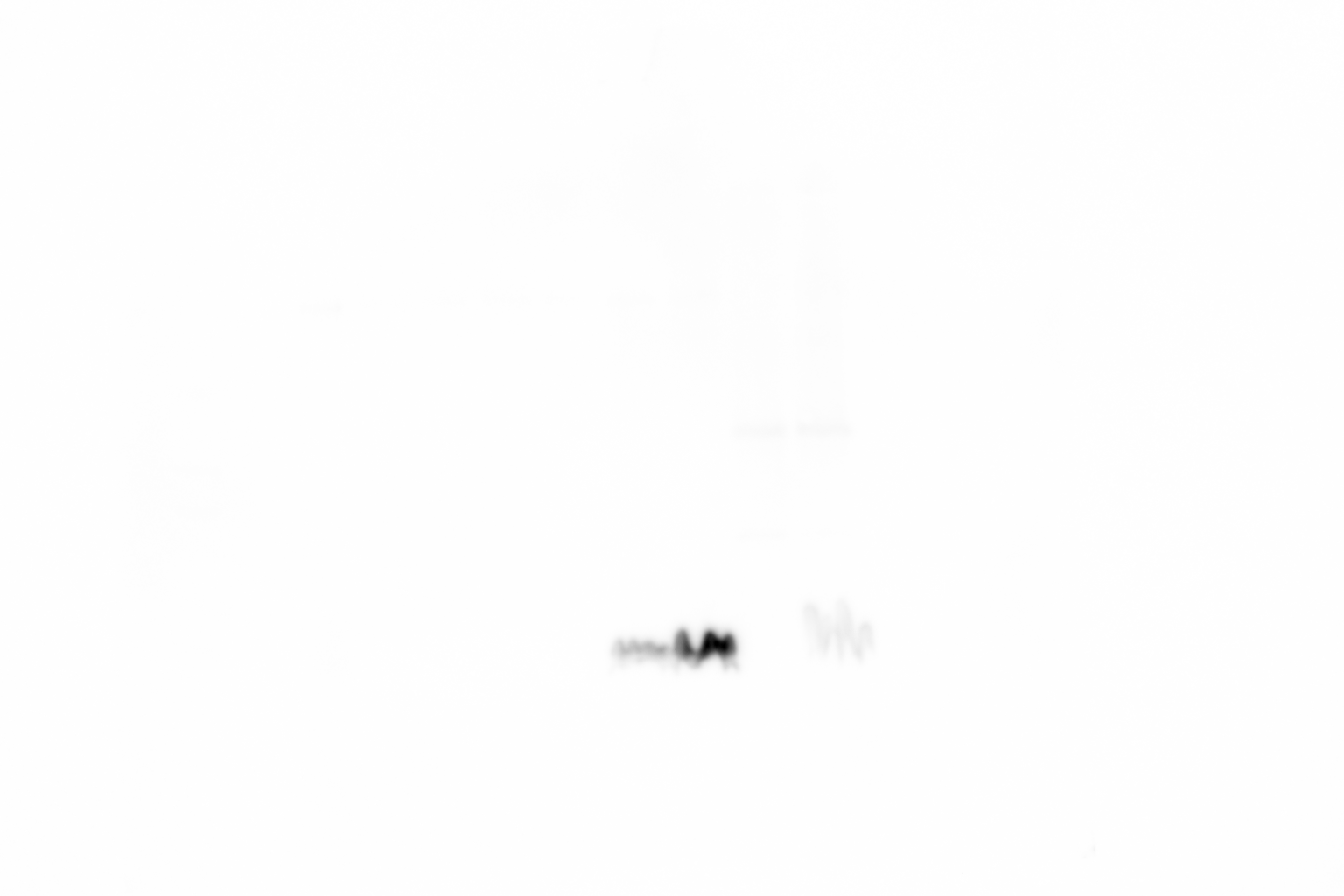

Supplement: Figure 5—source data 1. [file elife-37243-fig5-data1.zip › Figure 5 source data/2. EDADA in lipid II blot.tif]

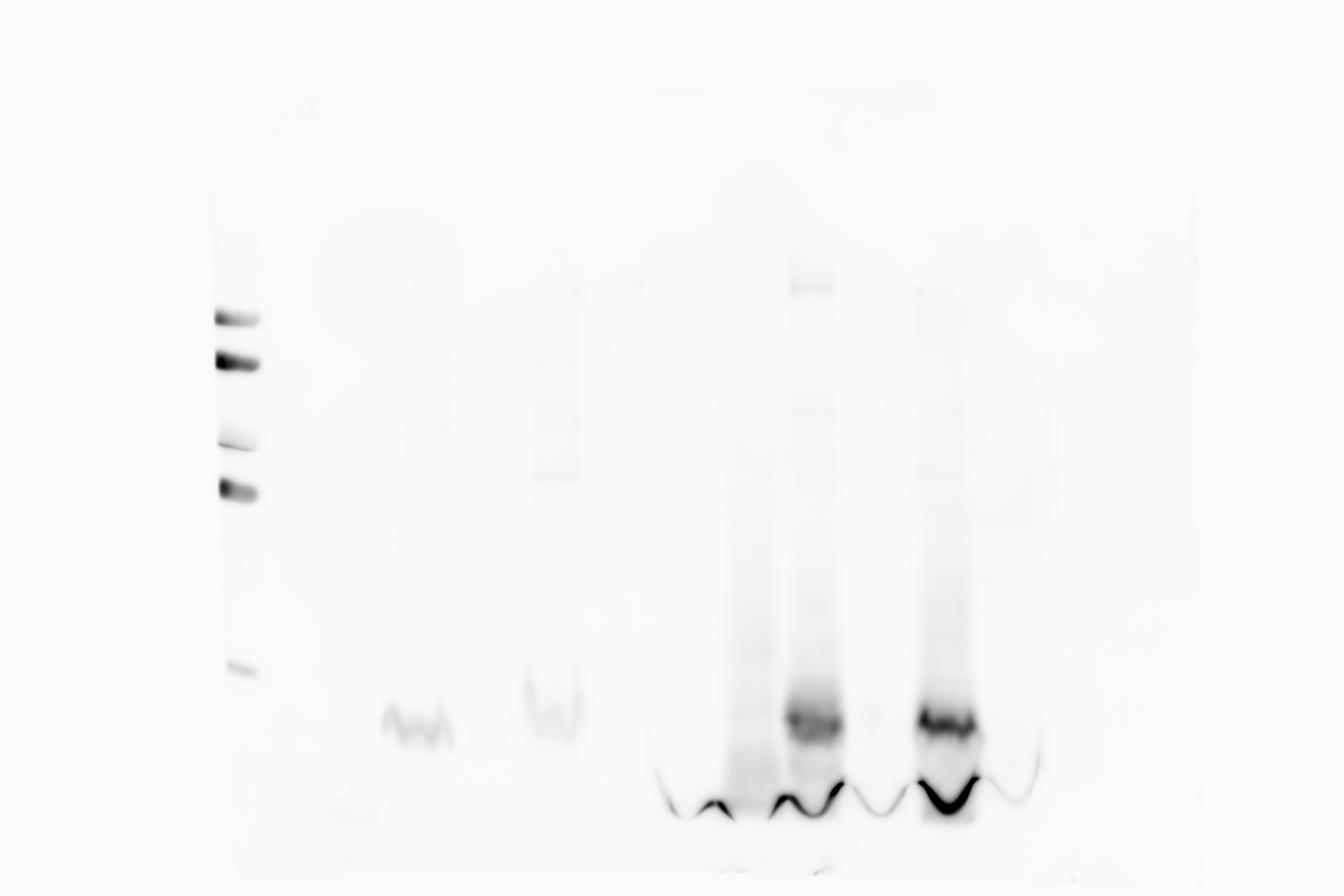

Supplement: Figure 5—source data 1. [file elife-37243-fig5-data1.zip › Figure 5 source data/3. ADADA in lipid II blot.tif]

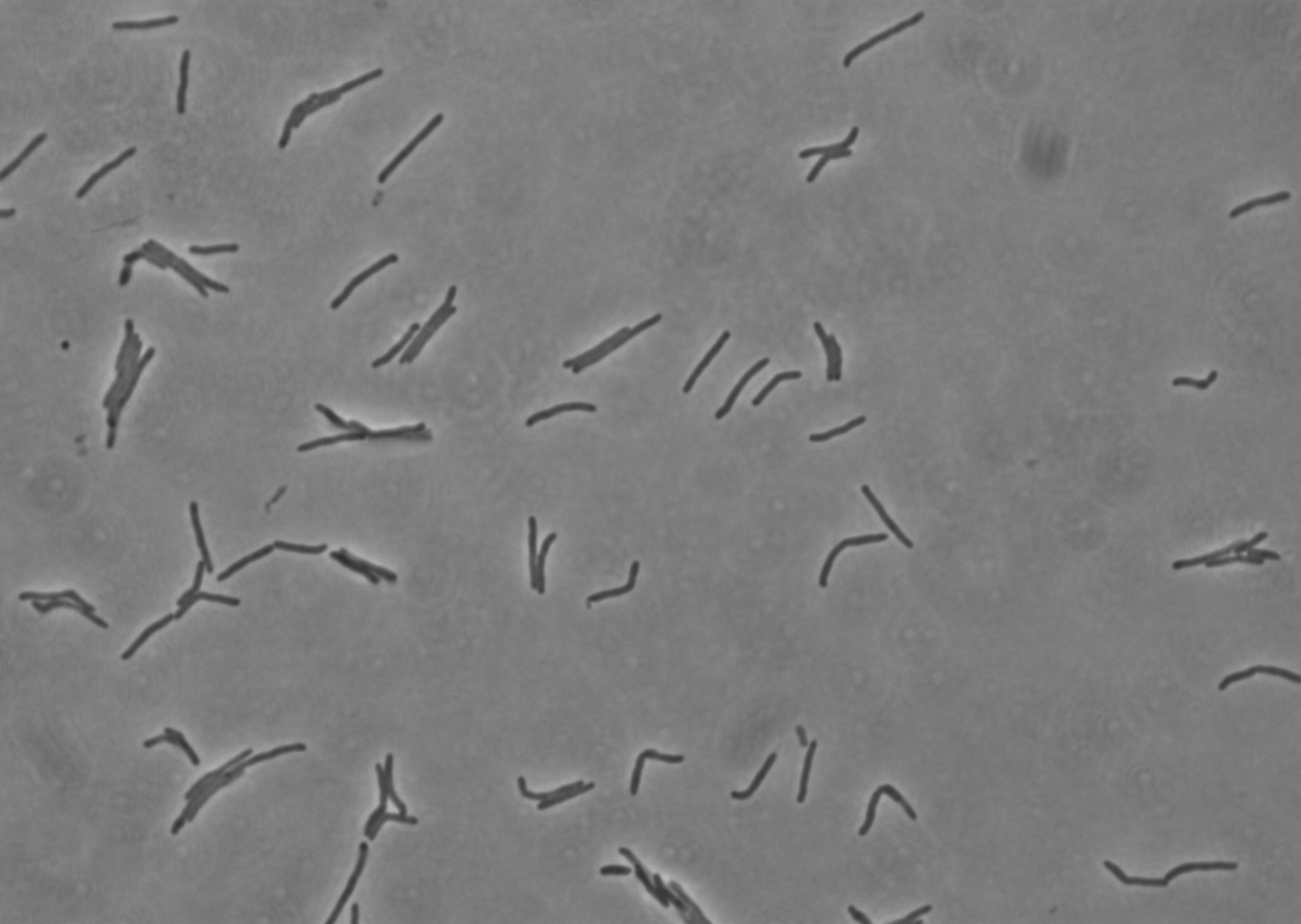

Supplement: Figure 5—figure supplement 3—source data 1. [file elife-37243-fig5-figsupp3-data1.zip › Figure 5-figure supplement 3 source data/2. Bocillin labeling- conventional microscopy /1. No bocillin/1. PC/1.tif]

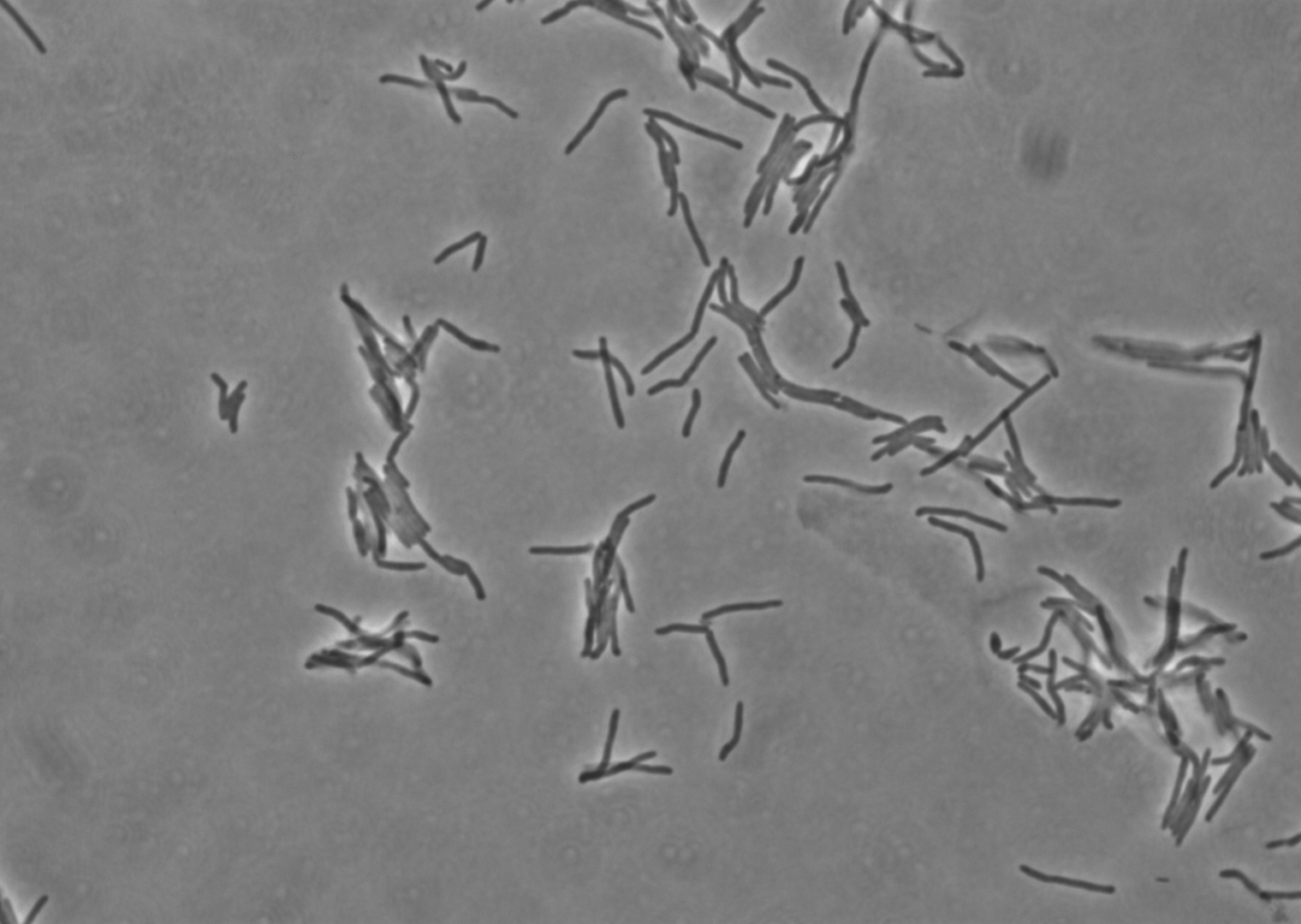

Supplement: Figure 5—figure supplement 3—source data 1. [file elife-37243-fig5-figsupp3-data1.zip › Figure 5-figure supplement 3 source data/2. Bocillin labeling- conventional microscopy /1. No bocillin/1. PC/2.tif]

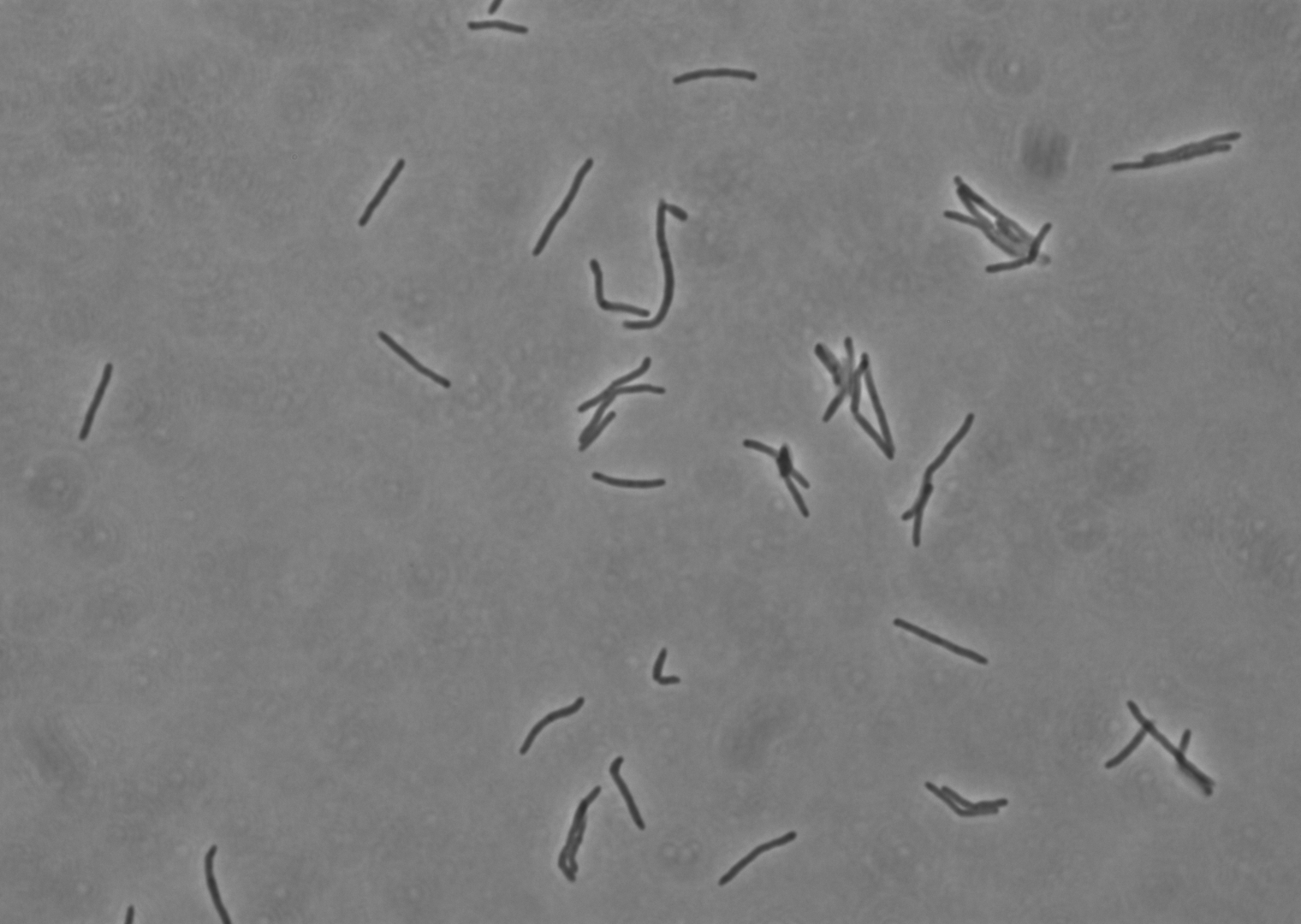

Supplement: Figure 5—figure supplement 3—source data 1. [file elife-37243-fig5-figsupp3-data1.zip › Figure 5-figure supplement 3 source data/2. Bocillin labeling- conventional microscopy /1. No bocillin/1. PC/3.tif]

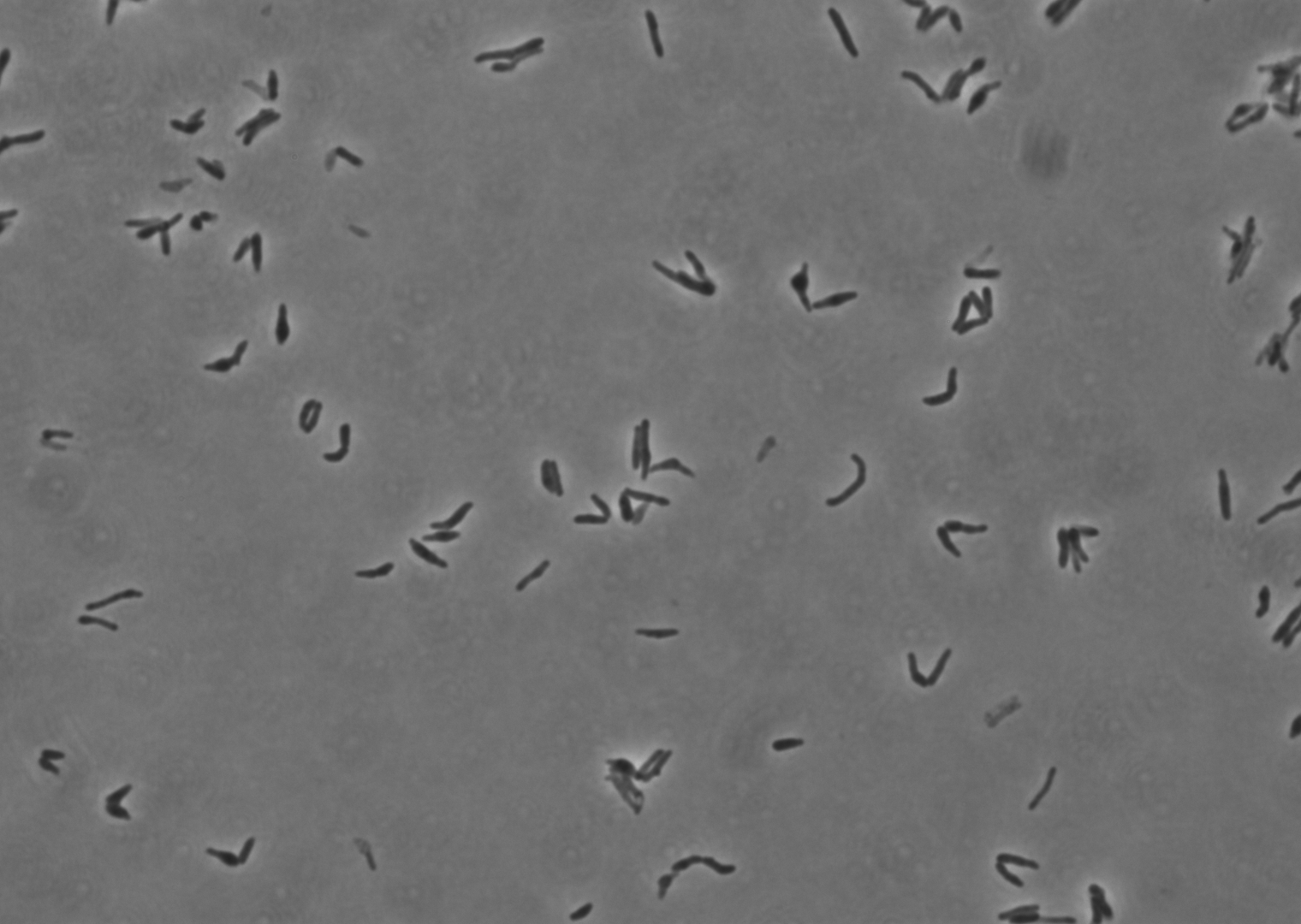

Supplement: Figure 5—figure supplement 3—source data 1. [file elife-37243-fig5-figsupp3-data1.zip › Figure 5-figure supplement 3 source data/2. Bocillin labeling- conventional microscopy /2. No antibiotics/1. PC/1.tif]

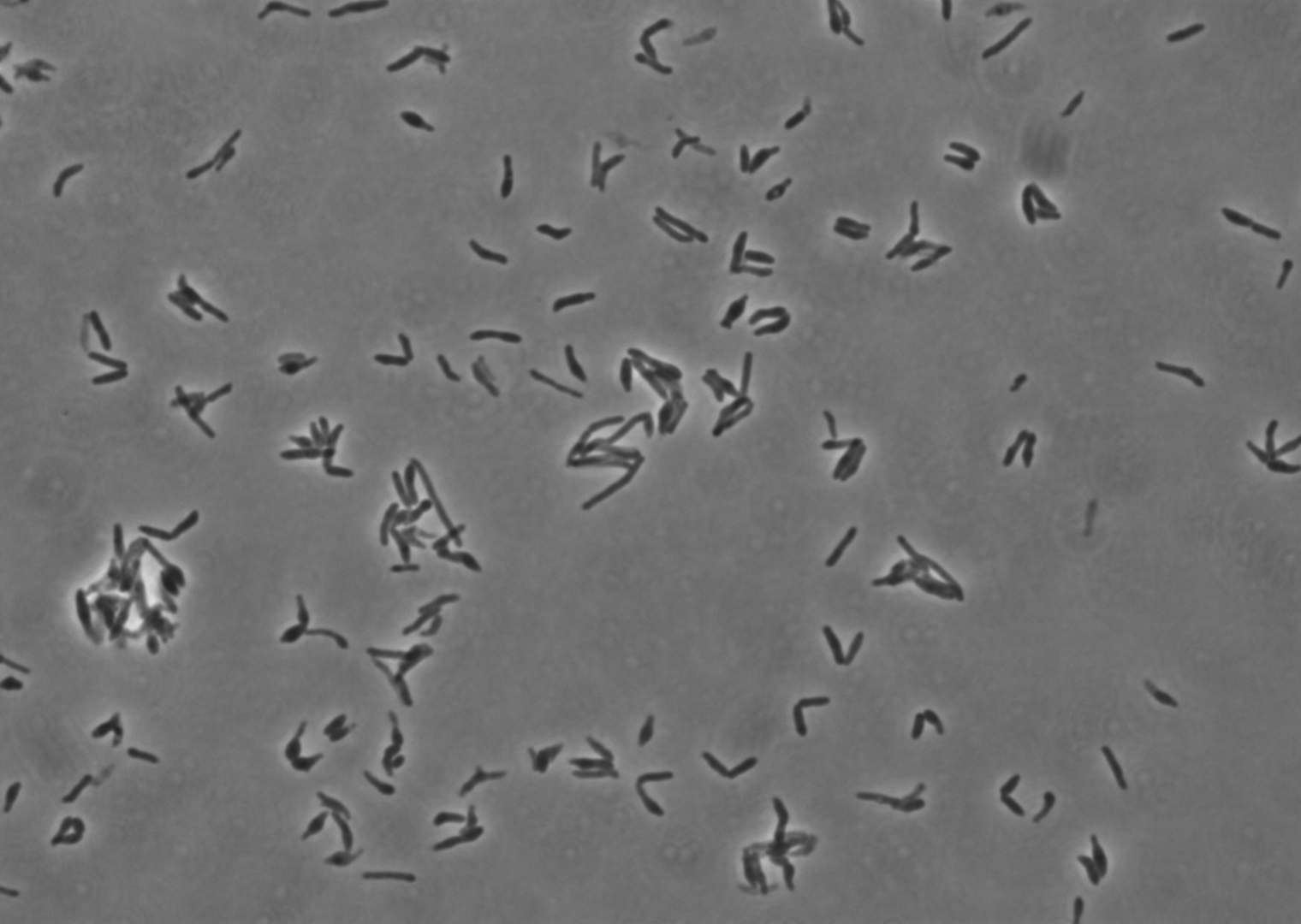

Supplement: Figure 5—figure supplement 3—source data 1. [file elife-37243-fig5-figsupp3-data1.zip › Figure 5-figure supplement 3 source data/2. Bocillin labeling- conventional microscopy /2. No antibiotics/1. PC/2.tif]

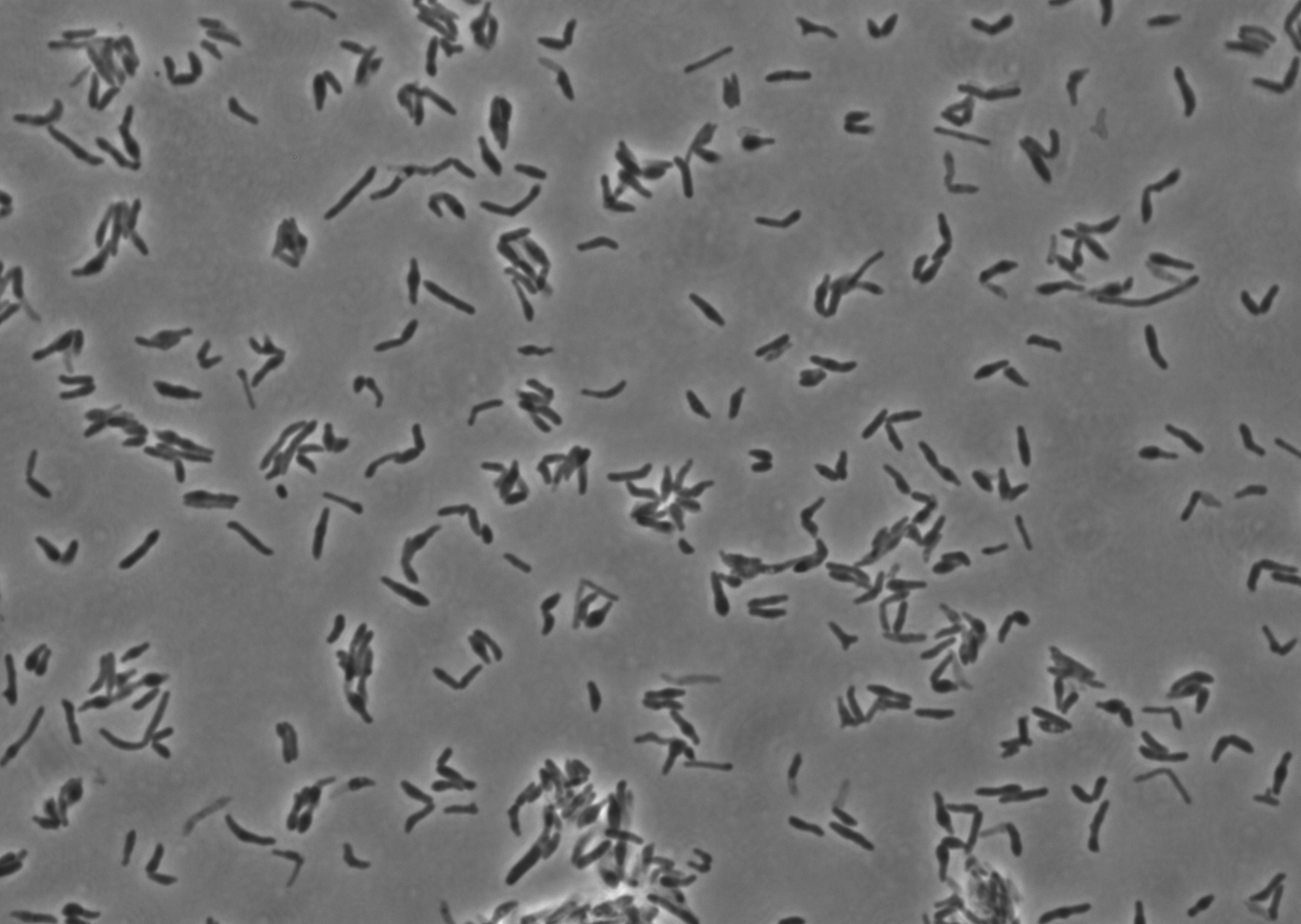

Supplement: Figure 5—figure supplement 3—source data 1. [file elife-37243-fig5-figsupp3-data1.zip › Figure 5-figure supplement 3 source data/2. Bocillin labeling- conventional microscopy /2. No antibiotics/1. PC/3.tif]

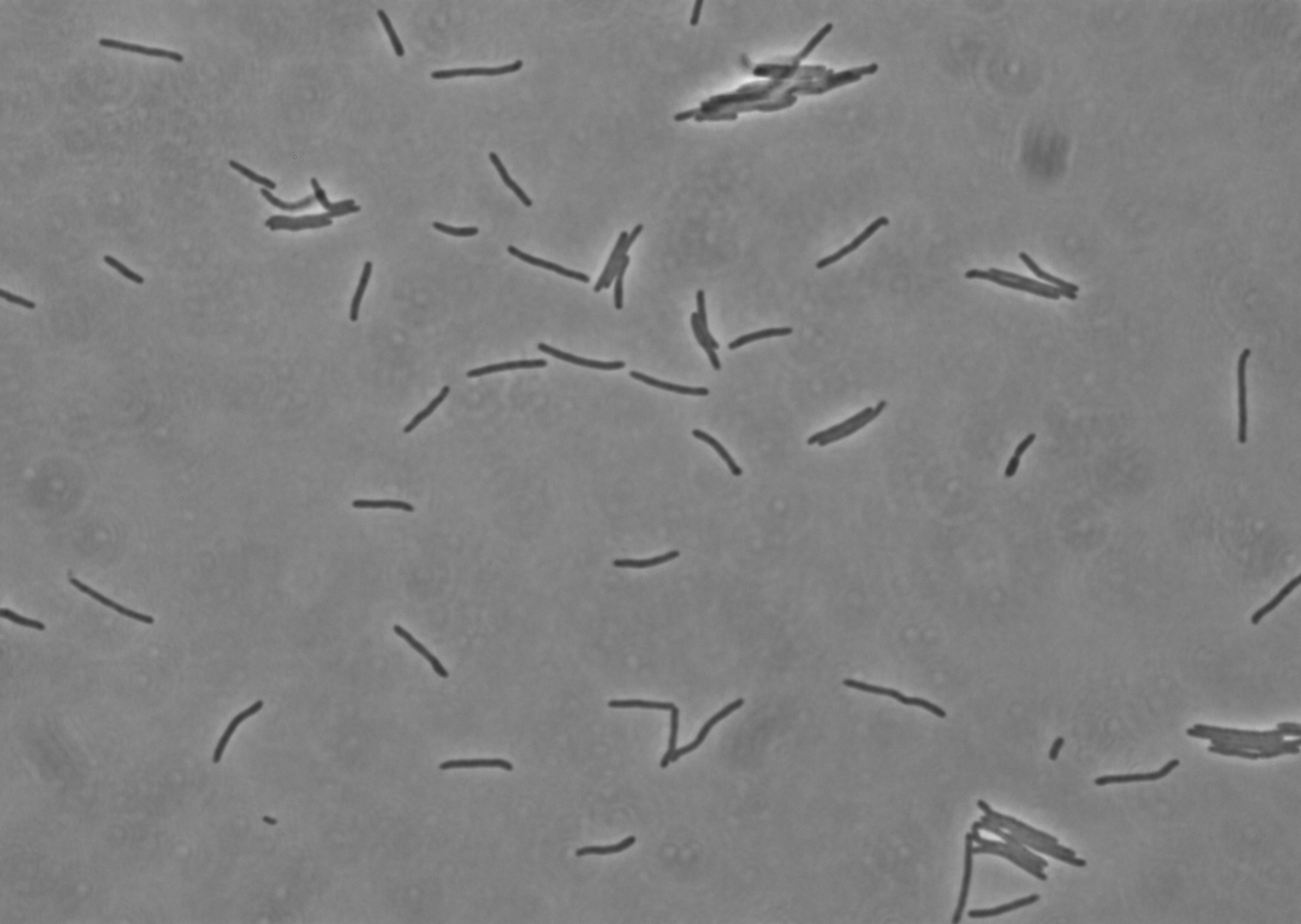

Supplement: Figure 5—figure supplement 3—source data 1. [file elife-37243-fig5-figsupp3-data1.zip › Figure 5-figure supplement 3 source data/2. Bocillin labeling- conventional microscopy /3. Ampicillin treated/1. PC/1.tif]

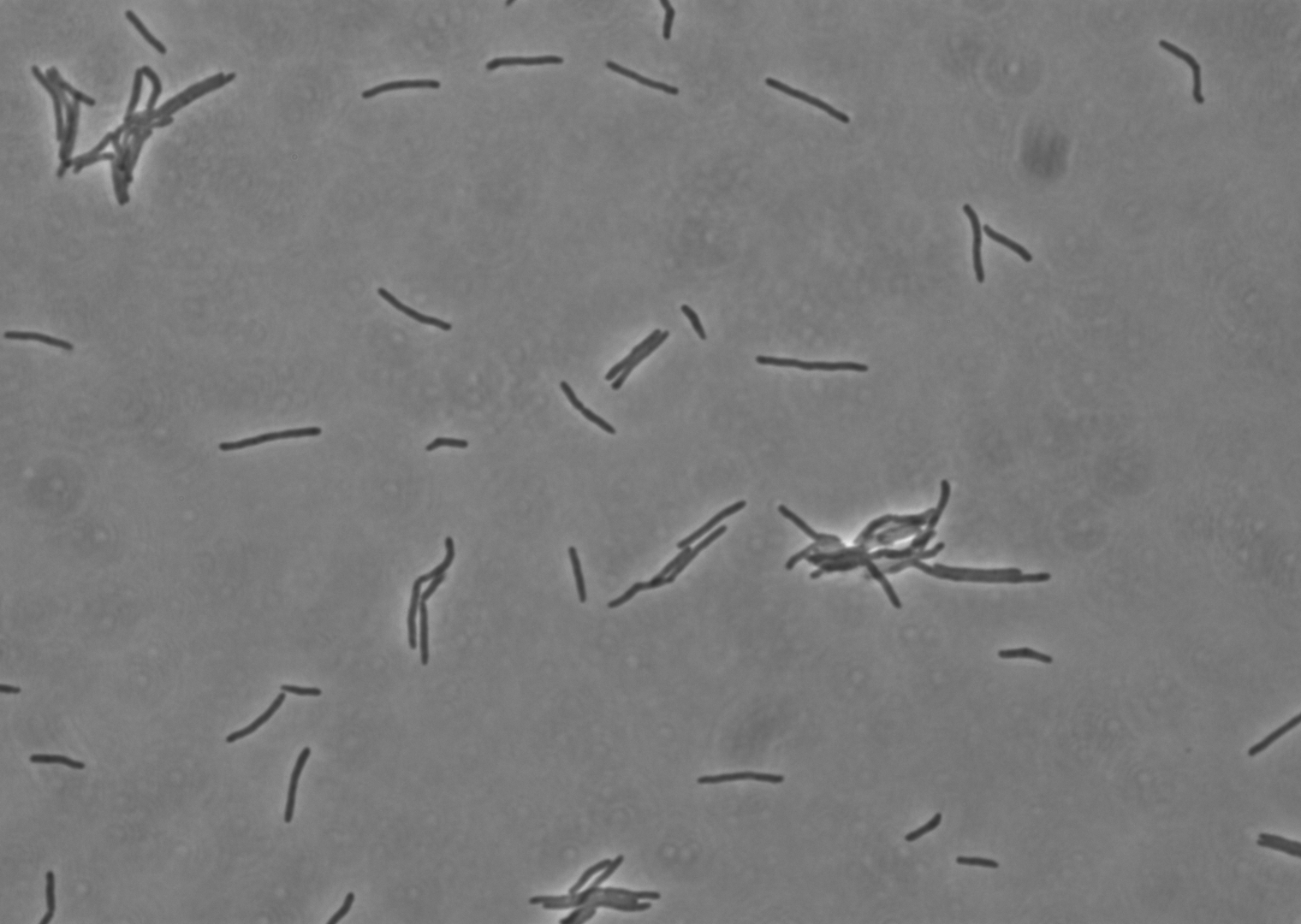

Supplement: Figure 5—figure supplement 3—source data 1. [file elife-37243-fig5-figsupp3-data1.zip › Figure 5-figure supplement 3 source data/2. Bocillin labeling- conventional microscopy /3. Ampicillin treated/1. PC/10.tif]

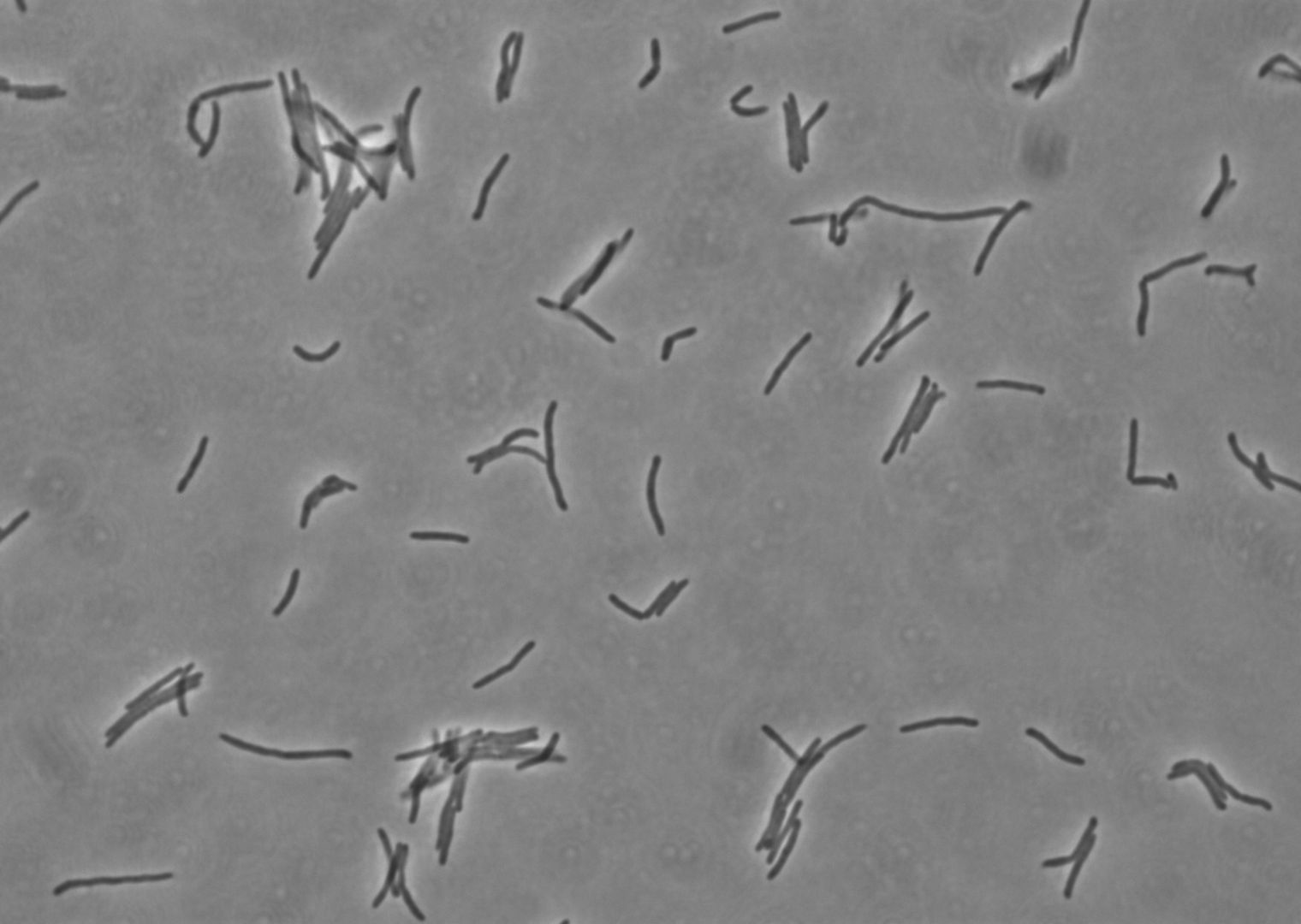

Supplement: Figure 5—figure supplement 3—source data 1. [file elife-37243-fig5-figsupp3-data1.zip › Figure 5-figure supplement 3 source data/2. Bocillin labeling- conventional microscopy /3. Ampicillin treated/1. PC/2.tif]

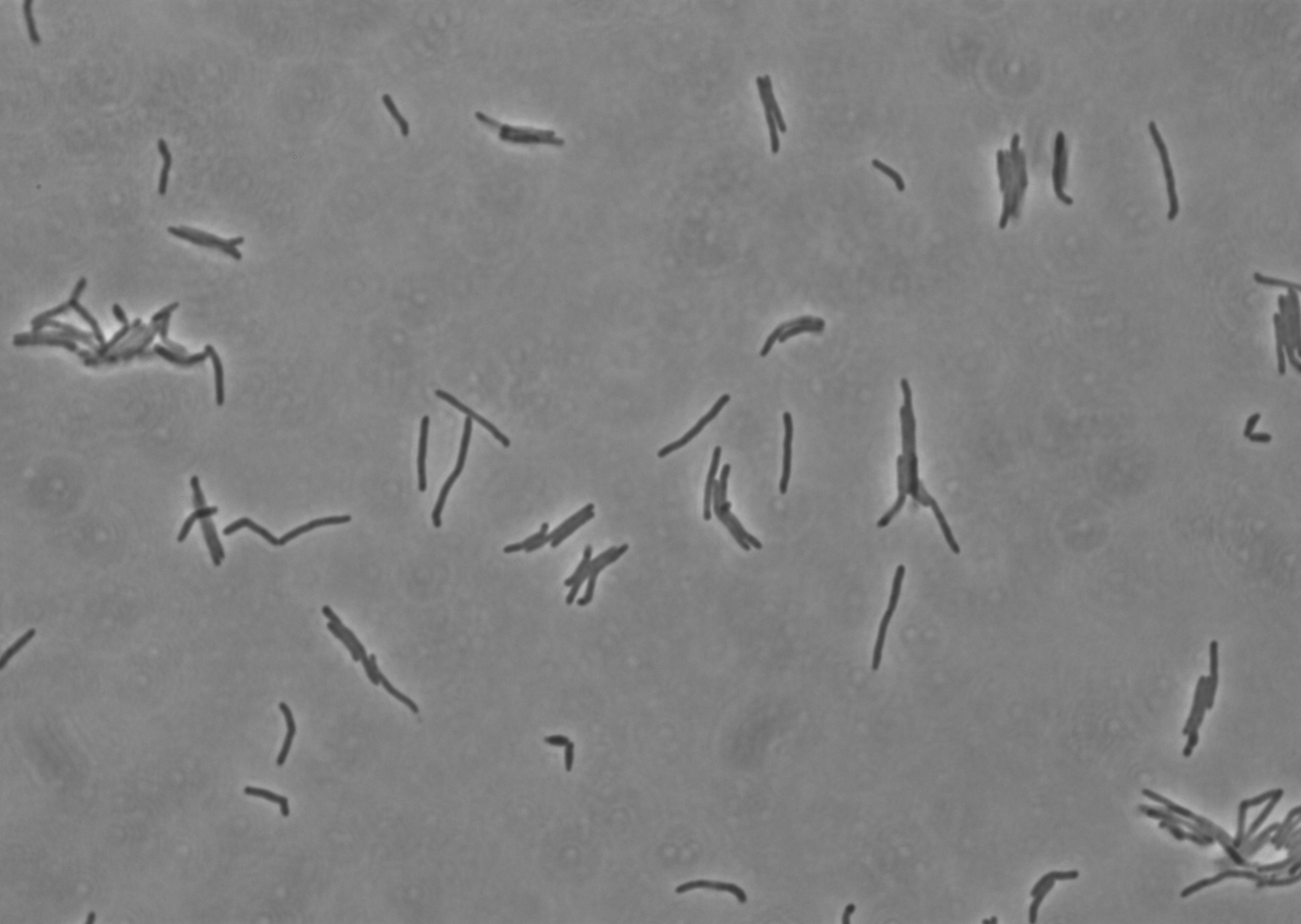

Supplement: Figure 5—figure supplement 3—source data 1. [file elife-37243-fig5-figsupp3-data1.zip › Figure 5-figure supplement 3 source data/2. Bocillin labeling- conventional microscopy /3. Ampicillin treated/1. PC/3.tif]

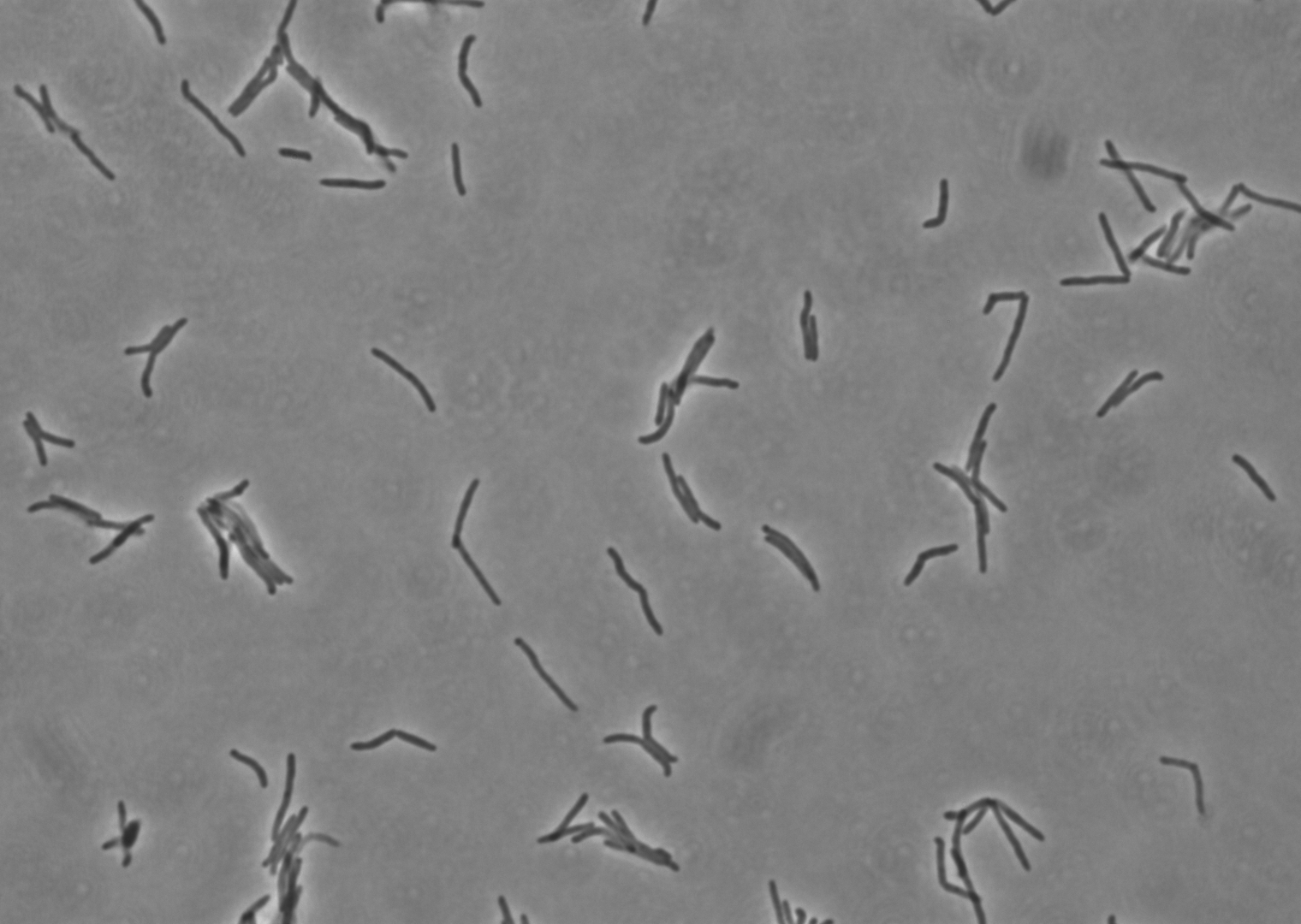

Supplement: Figure 5—figure supplement 3—source data 1. [file elife-37243-fig5-figsupp3-data1.zip › Figure 5-figure supplement 3 source data/2. Bocillin labeling- conventional microscopy /3. Ampicillin treated/1. PC/4.tif]

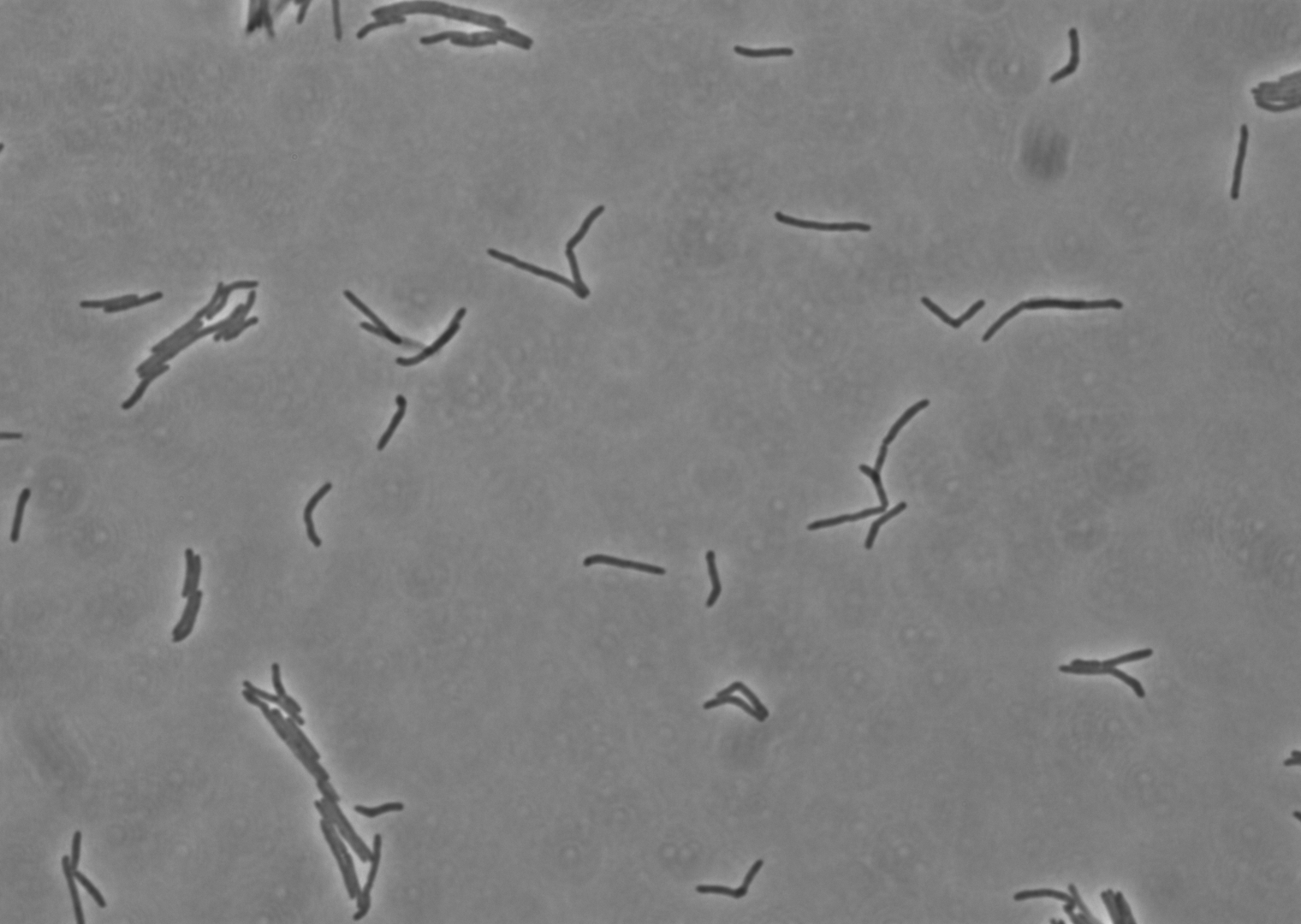

Supplement: Figure 5—figure supplement 3—source data 1. [file elife-37243-fig5-figsupp3-data1.zip › Figure 5-figure supplement 3 source data/2. Bocillin labeling- conventional microscopy /3. Ampicillin treated/1. PC/5.tif]

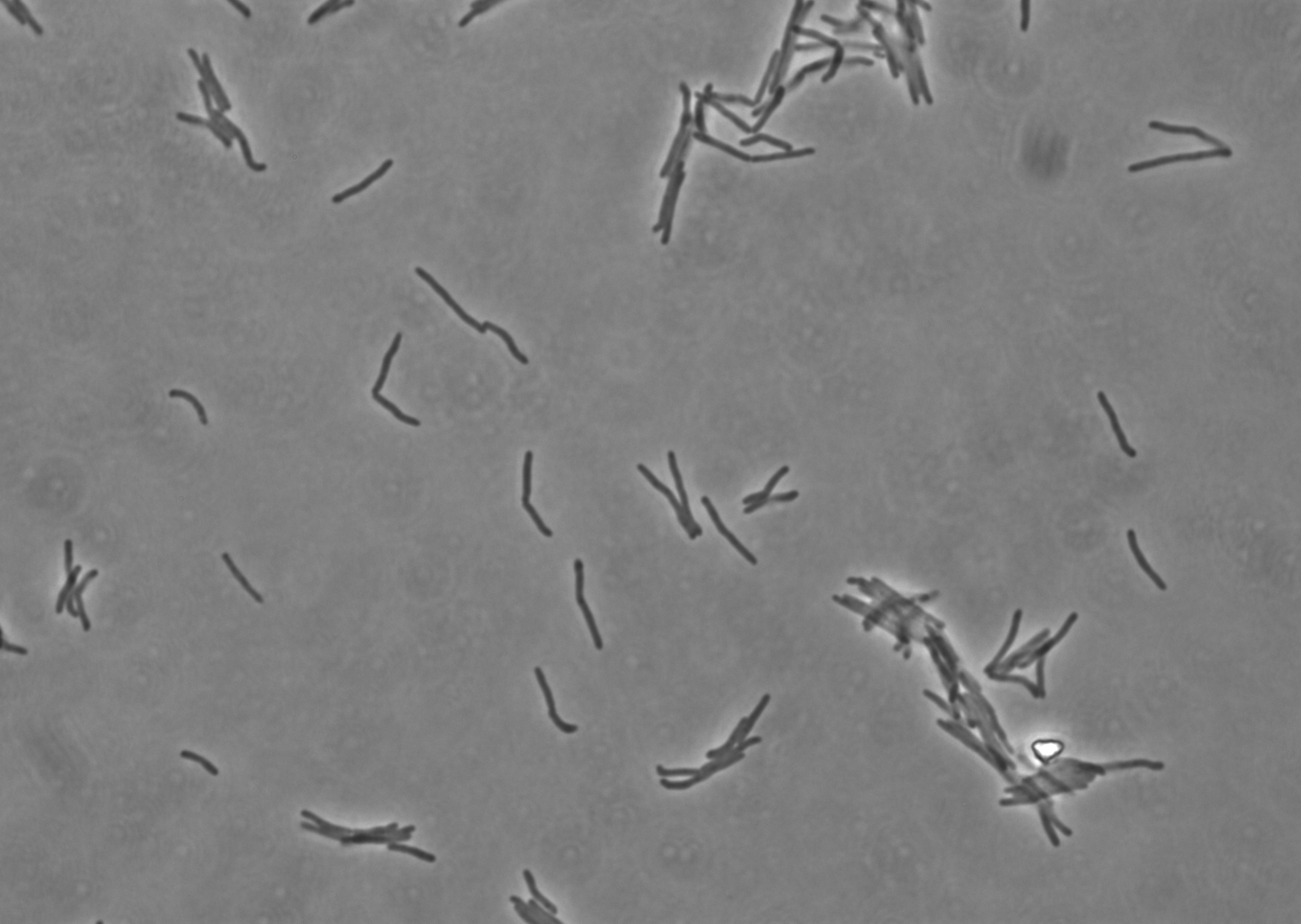

Supplement: Figure 5—figure supplement 3—source data 1. [file elife-37243-fig5-figsupp3-data1.zip › Figure 5-figure supplement 3 source data/2. Bocillin labeling- conventional microscopy /3. Ampicillin treated/1. PC/6.tif]

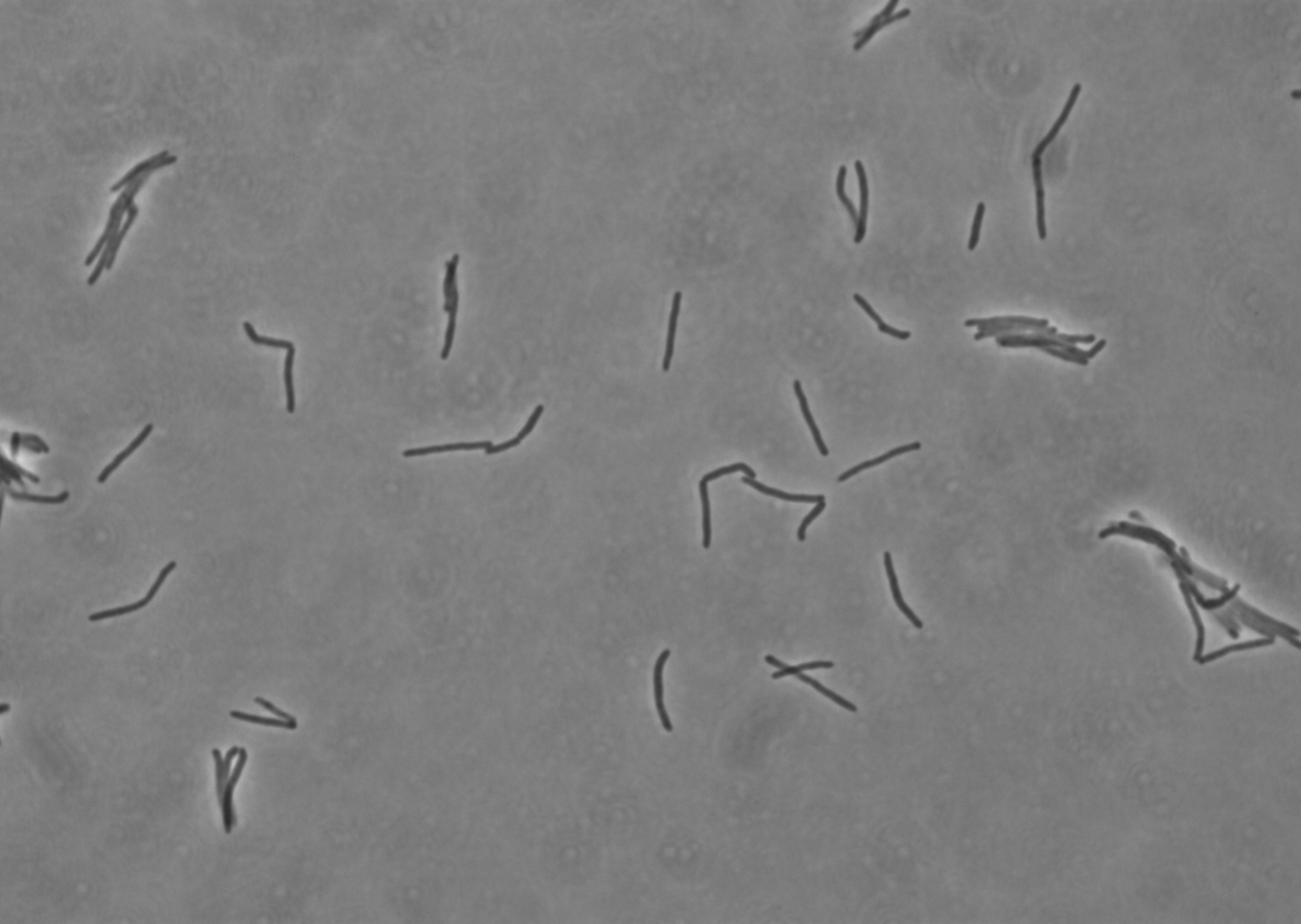

Supplement: Figure 5—figure supplement 3—source data 1. [file elife-37243-fig5-figsupp3-data1.zip › Figure 5-figure supplement 3 source data/2. Bocillin labeling- conventional microscopy /3. Ampicillin treated/1. PC/7.tif]

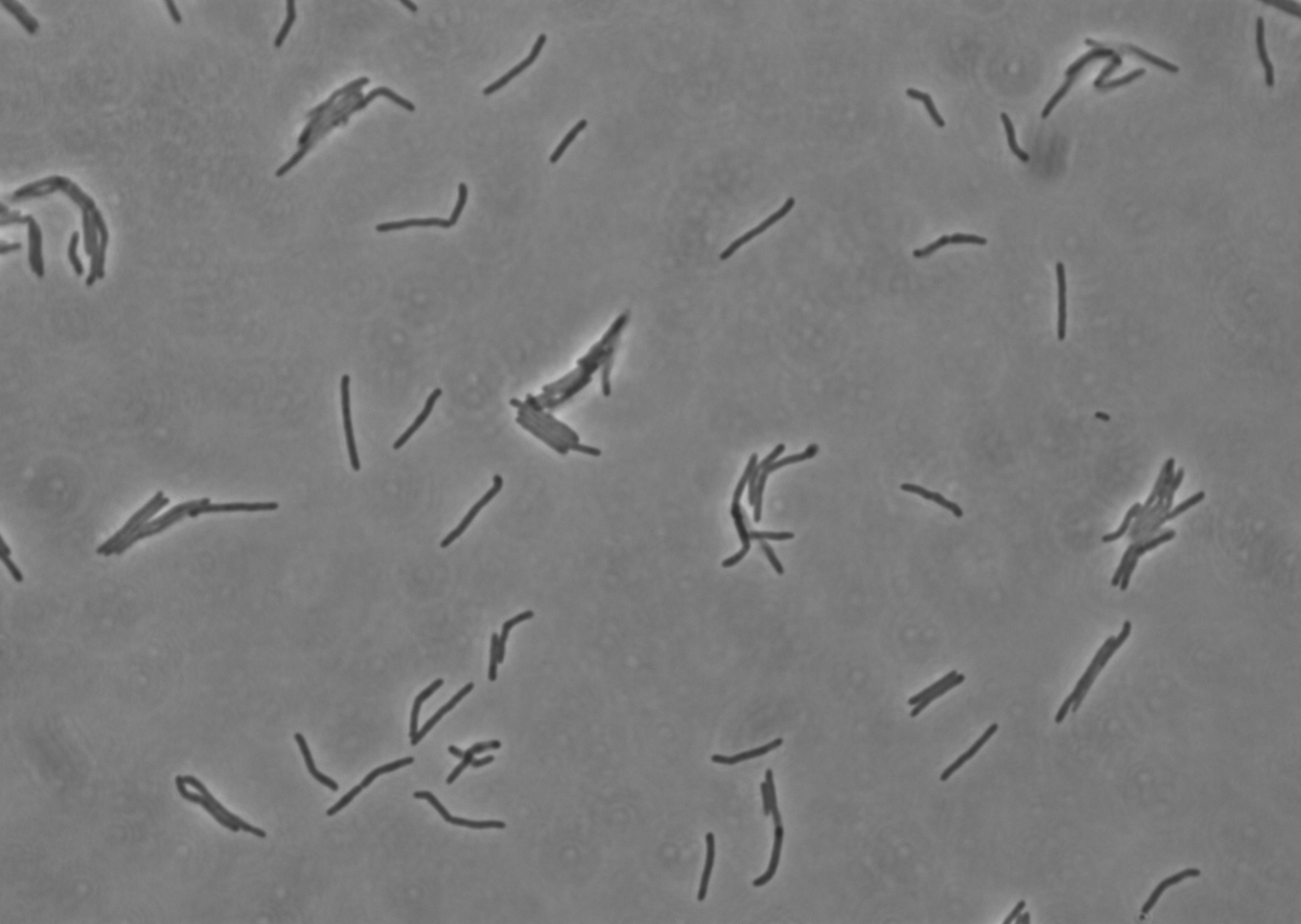

Supplement: Figure 5—figure supplement 3—source data 1. [file elife-37243-fig5-figsupp3-data1.zip › Figure 5-figure supplement 3 source data/2. Bocillin labeling- conventional microscopy /3. Ampicillin treated/1. PC/8.tif]

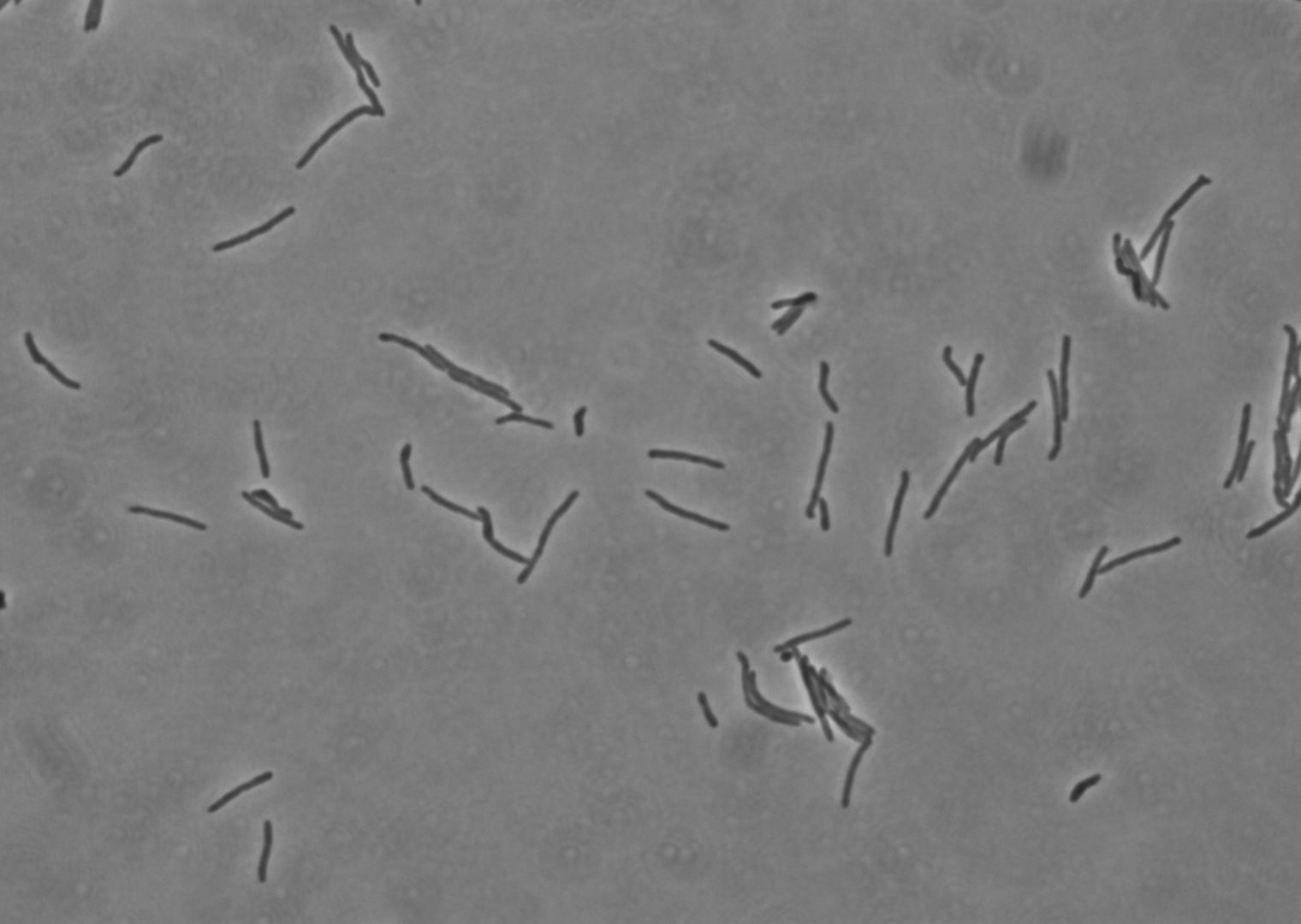

Supplement: Figure 5—figure supplement 3—source data 1. [file elife-37243-fig5-figsupp3-data1.zip › Figure 5-figure supplement 3 source data/2. Bocillin labeling- conventional microscopy /3. Ampicillin treated/1. PC/9.tif]

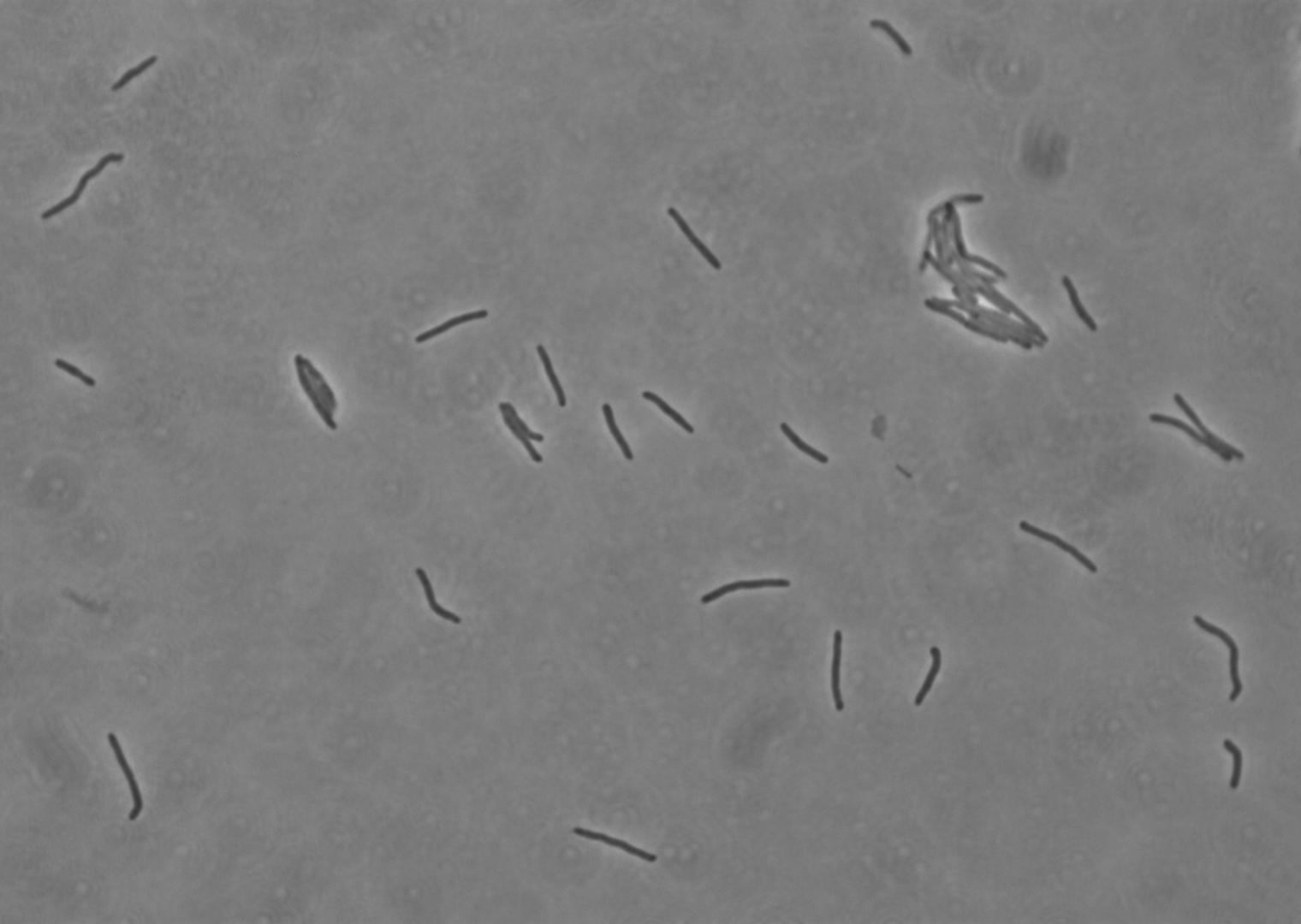

Supplement: Figure 5—figure supplement 3—source data 1. [file elife-37243-fig5-figsupp3-data1.zip › Figure 5-figure supplement 3 source data/2. Bocillin labeling- conventional microscopy /4. DCS treated/1. PC/1.tif]
