# Supplementary figures and images for: Peptidoglycan precursor synthesis along the sidewall of pole-growing mycobacteria (part 1 of 3)
Source: eLife. 2018 Sep 10;7:e37243. doi: 10.7554/eLife.37243 (PMC6191288; doi:10.7554/eLife.37243)

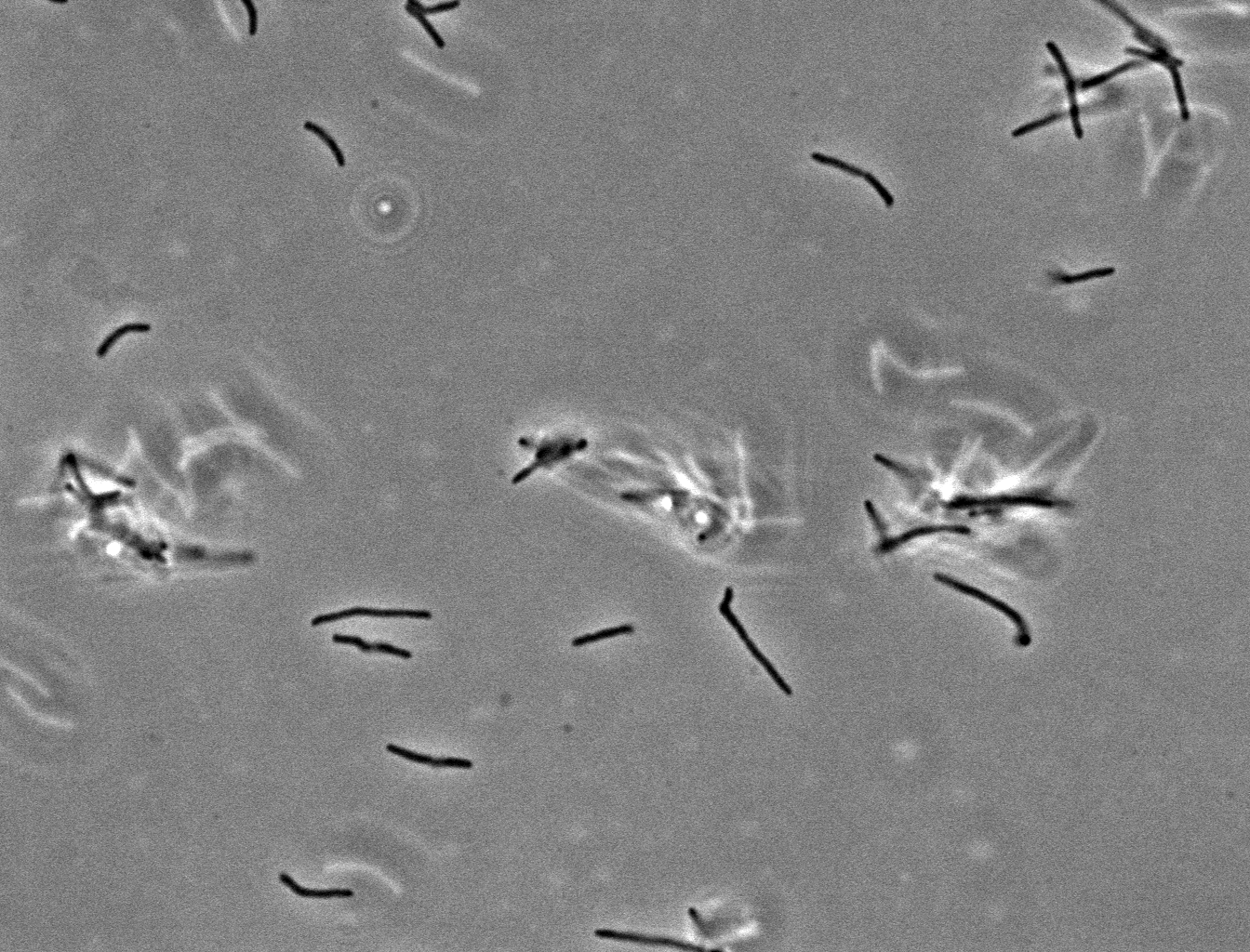

Supplement: Figure 2—source data 1. [file elife-37243-fig2-data1.zip › Figure 2 source data/Figure 2 source data-conventional microscopy (AlkDa + RADA)/1. Phase/1.tif]

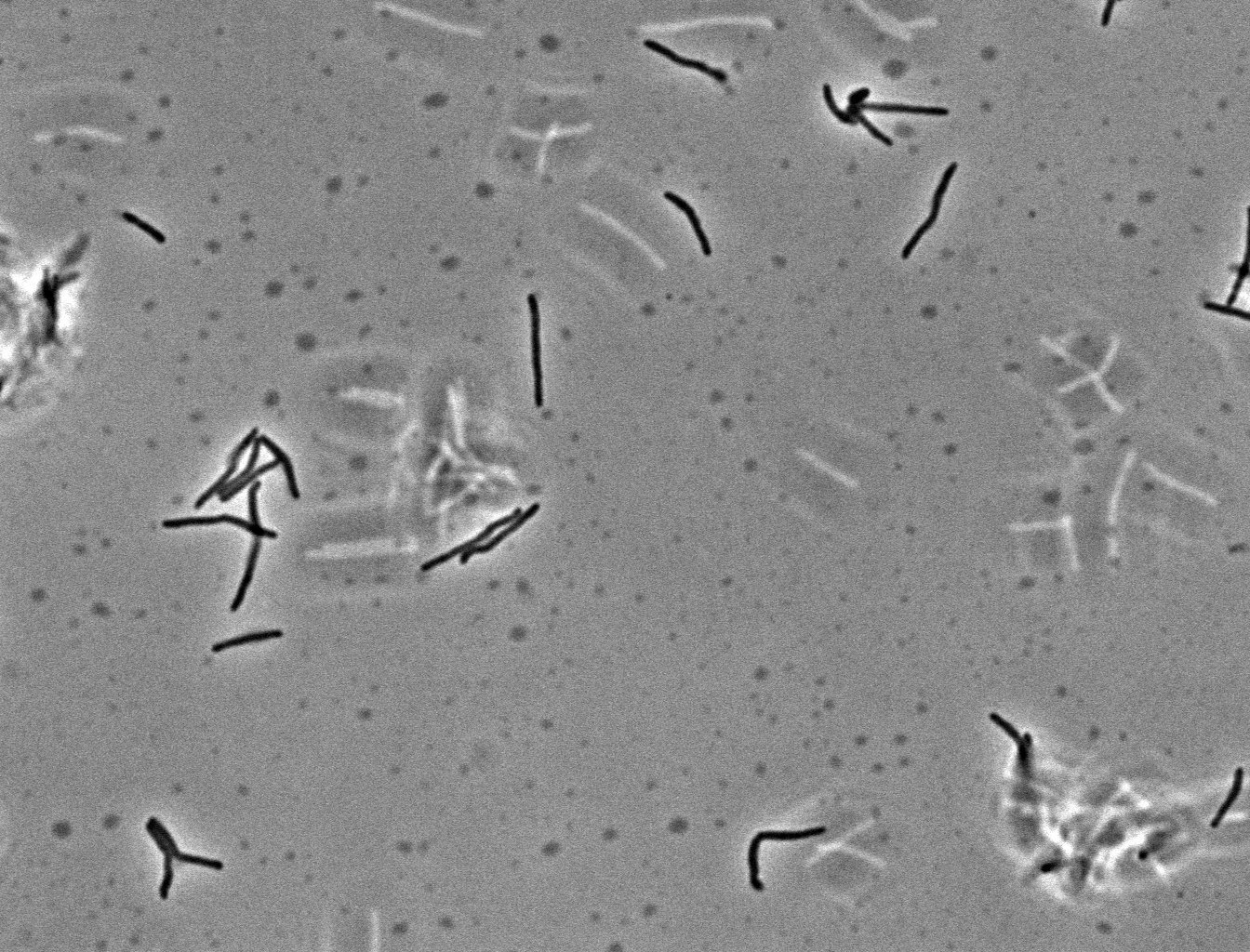

Supplement: Figure 2—source data 1. [file elife-37243-fig2-data1.zip › Figure 2 source data/Figure 2 source data-conventional microscopy (AlkDa + RADA)/1. Phase/2.tif]

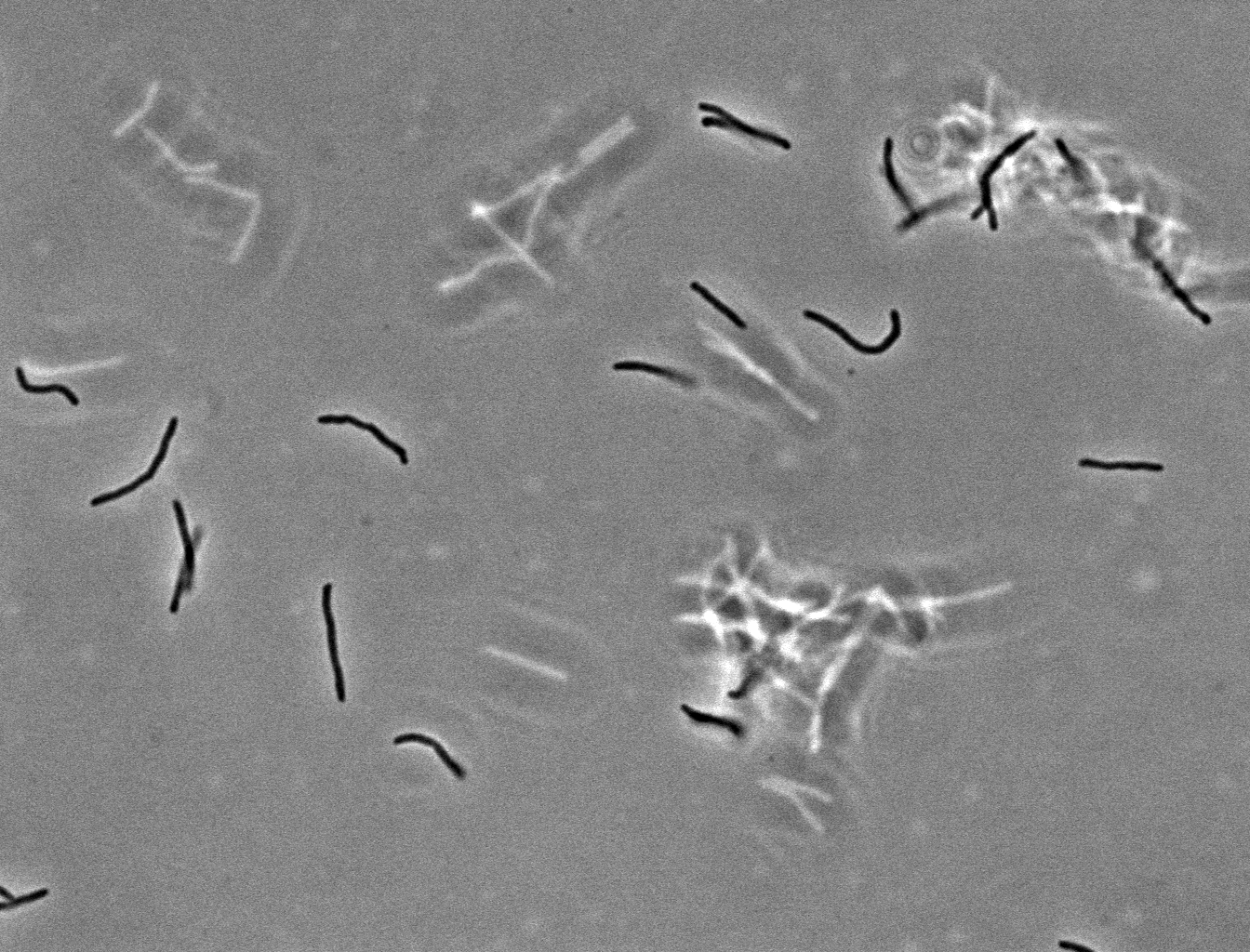

Supplement: Figure 2—source data 1. [file elife-37243-fig2-data1.zip › Figure 2 source data/Figure 2 source data-conventional microscopy (AlkDa + RADA)/1. Phase/3.tif]

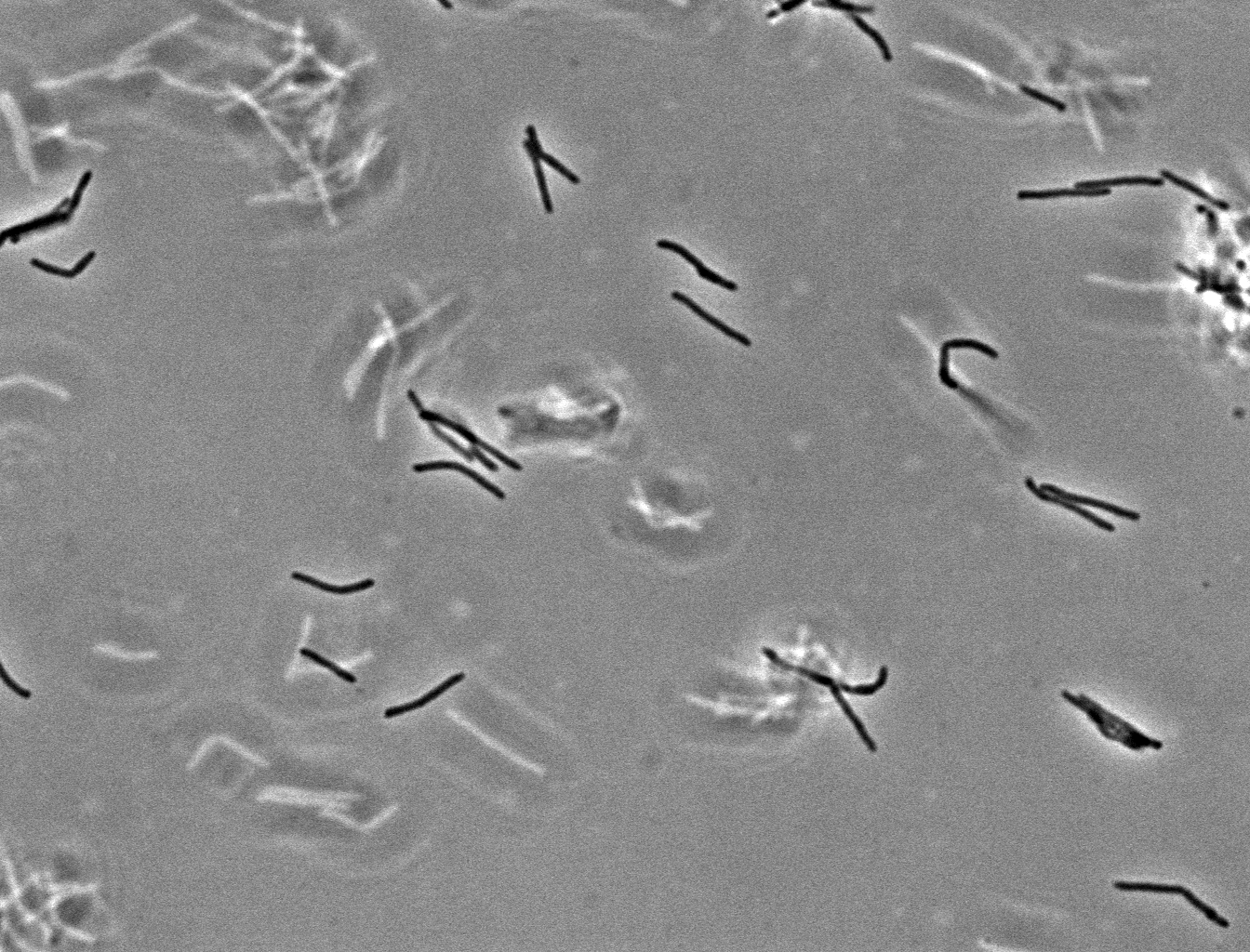

Supplement: Figure 2—source data 1. [file elife-37243-fig2-data1.zip › Figure 2 source data/Figure 2 source data-conventional microscopy (AlkDa + RADA)/1. Phase/4.tif]

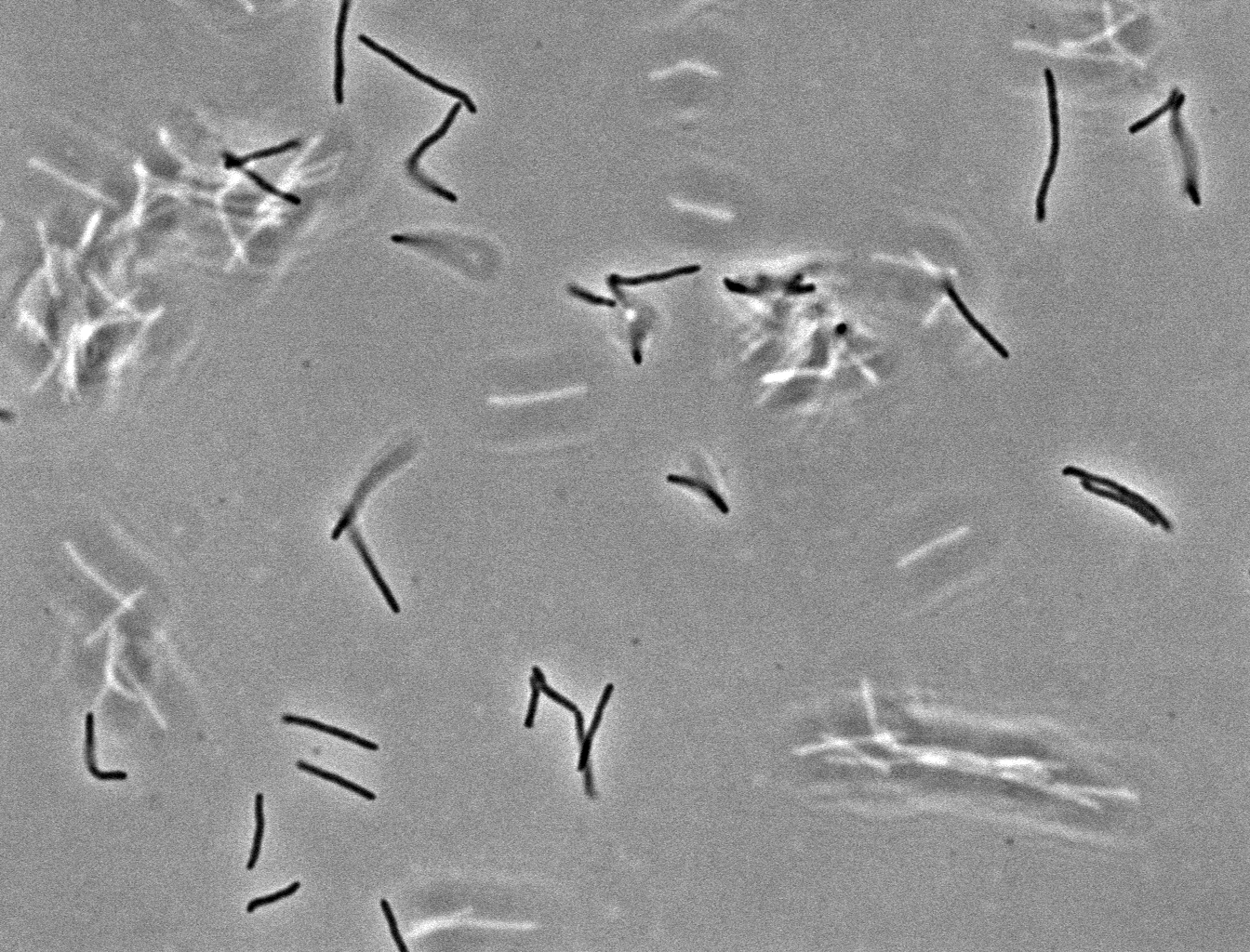

Supplement: Figure 2—source data 1. [file elife-37243-fig2-data1.zip › Figure 2 source data/Figure 2 source data-conventional microscopy (AlkDa + RADA)/1. Phase/5.tif]

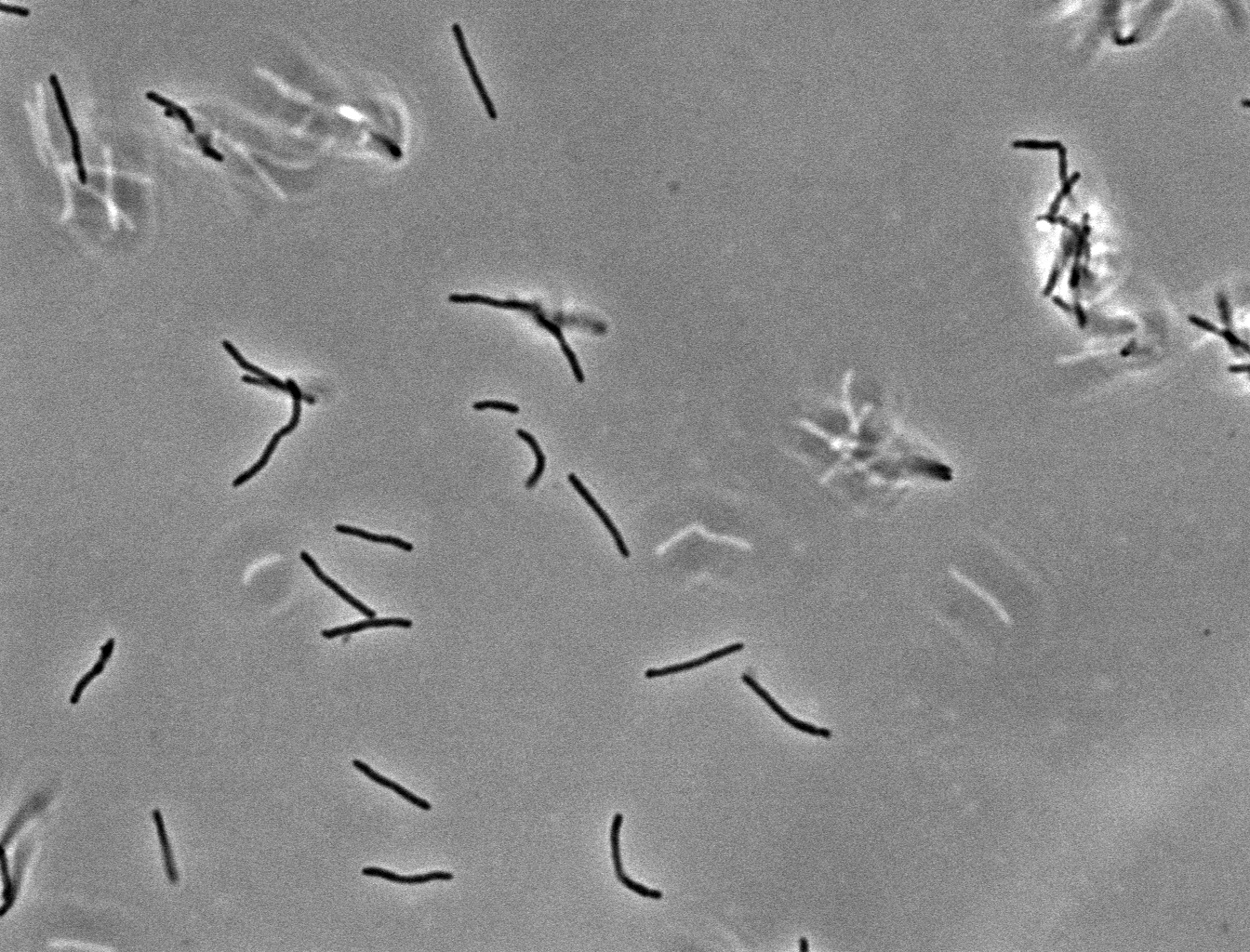

Supplement: Figure 2—source data 1. [file elife-37243-fig2-data1.zip › Figure 2 source data/Figure 2 source data-conventional microscopy (AlkDa + RADA)/1. Phase/6.tif]

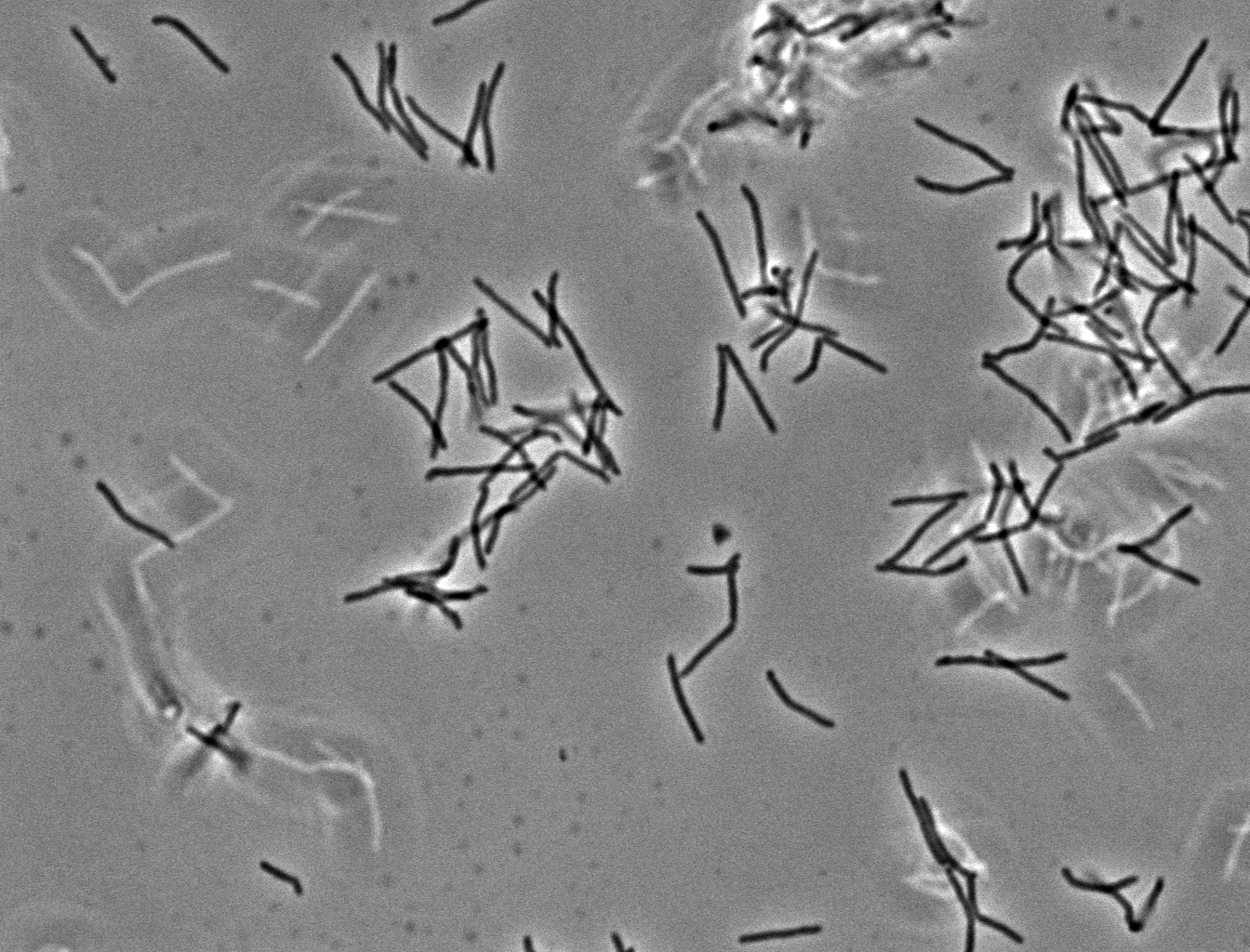

Supplement: Figure 2—source data 1. [file elife-37243-fig2-data1.zip › Figure 2 source data/Figure 2 source data-conventional microscopy (AlkDa + RADA)/1. Phase/7.tif]

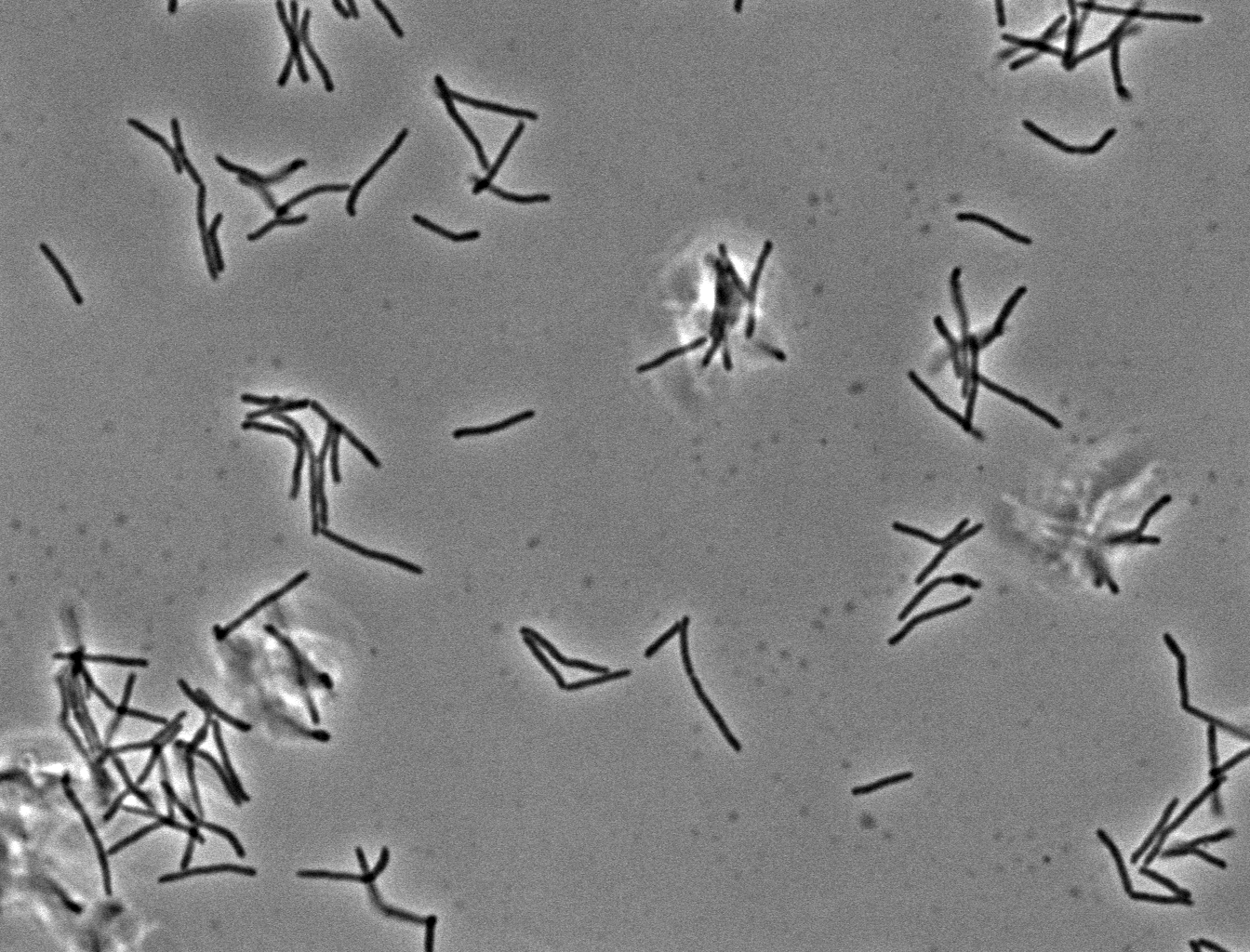

Supplement: Figure 2—source data 1. [file elife-37243-fig2-data1.zip › Figure 2 source data/Figure 2 source data-conventional microscopy (AlkDa + RADA)/1. Phase/8.tif]

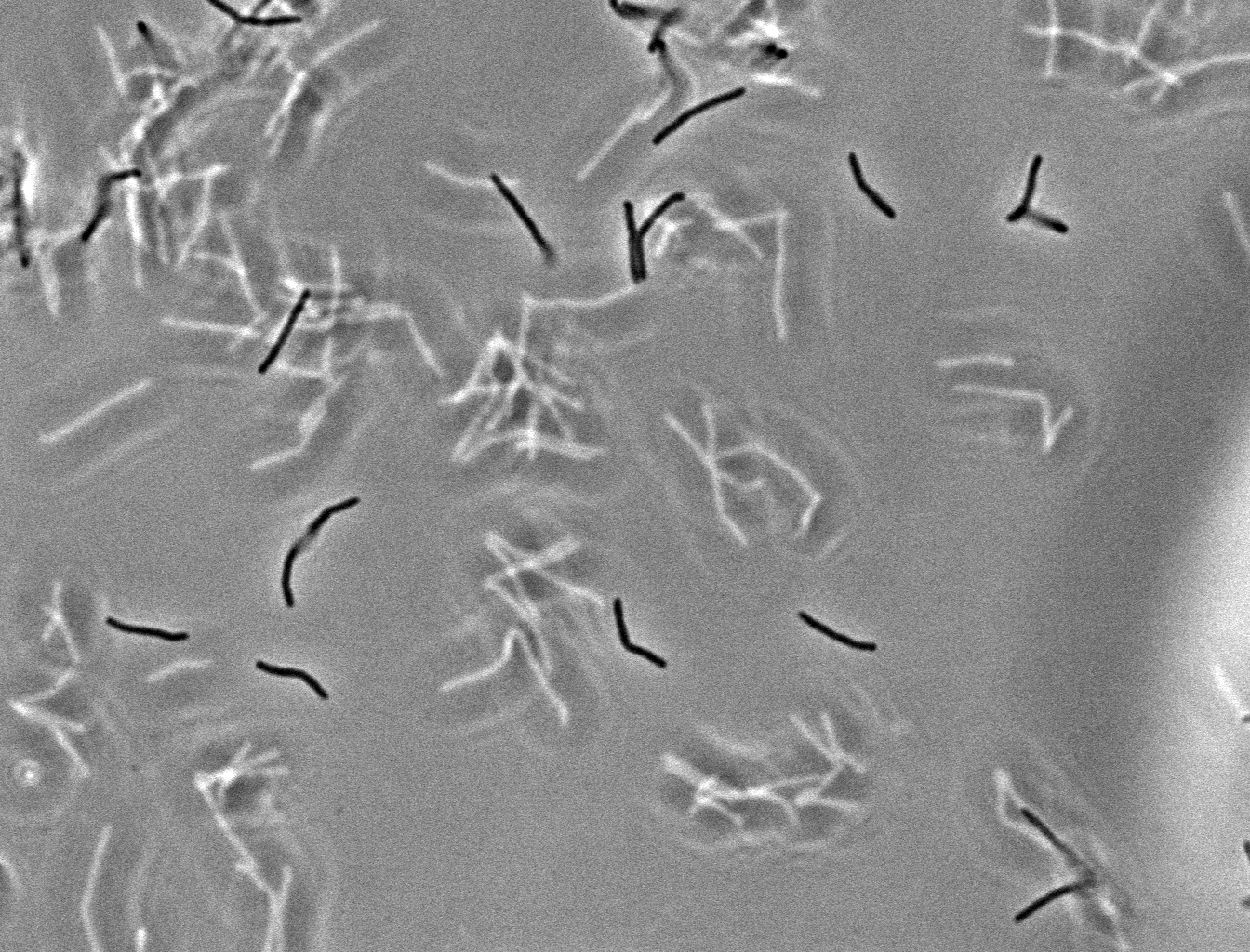

Supplement: Figure 2—source data 1. [file elife-37243-fig2-data1.zip › Figure 2 source data/Figure 2 source data-conventional microscopy (AlkDa + RADA)/1. Phase/9.tif]

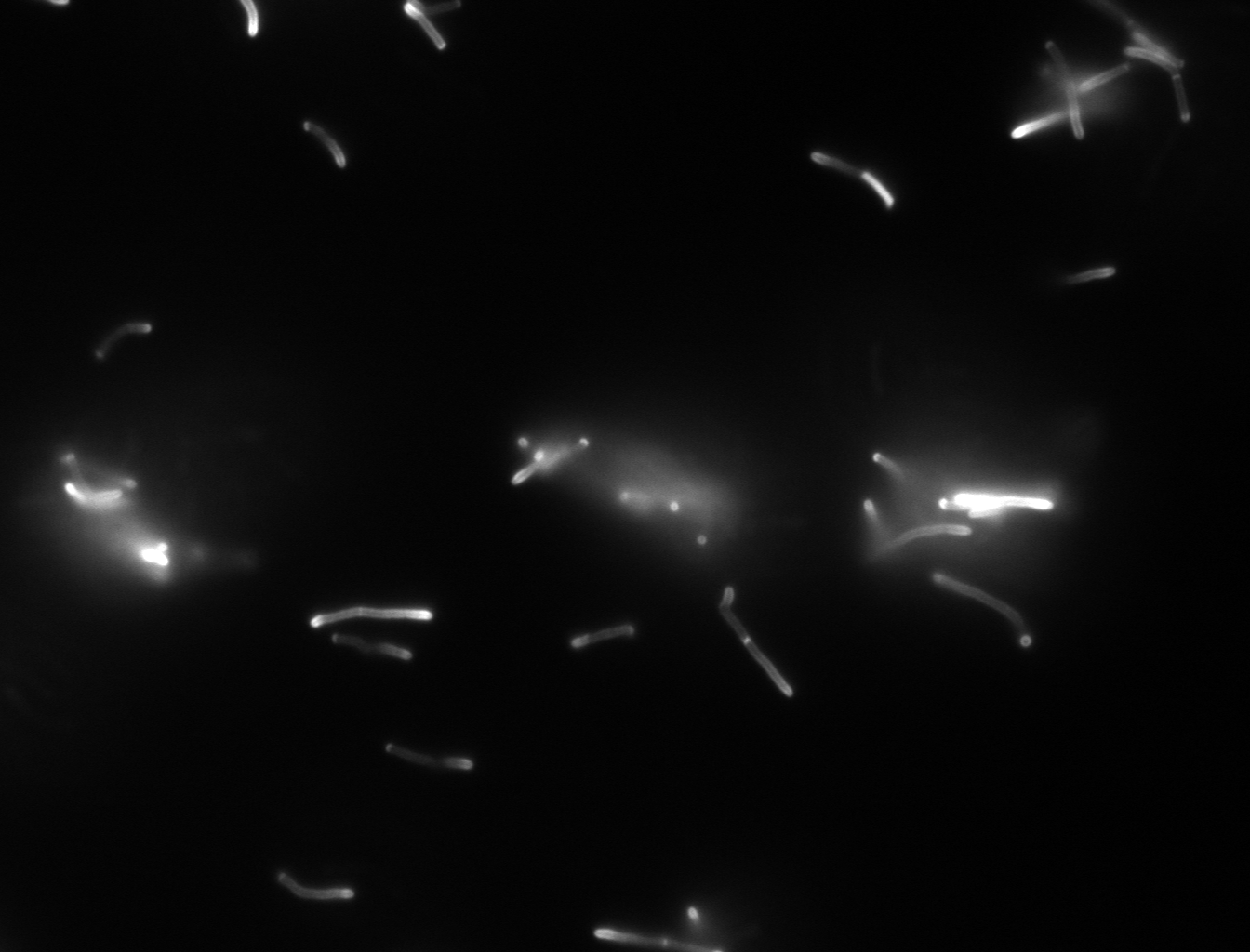

Supplement: Figure 2—source data 1. [file elife-37243-fig2-data1.zip › Figure 2 source data/Figure 2 source data-conventional microscopy (AlkDa + RADA)/2. AlkDa/1.tif]

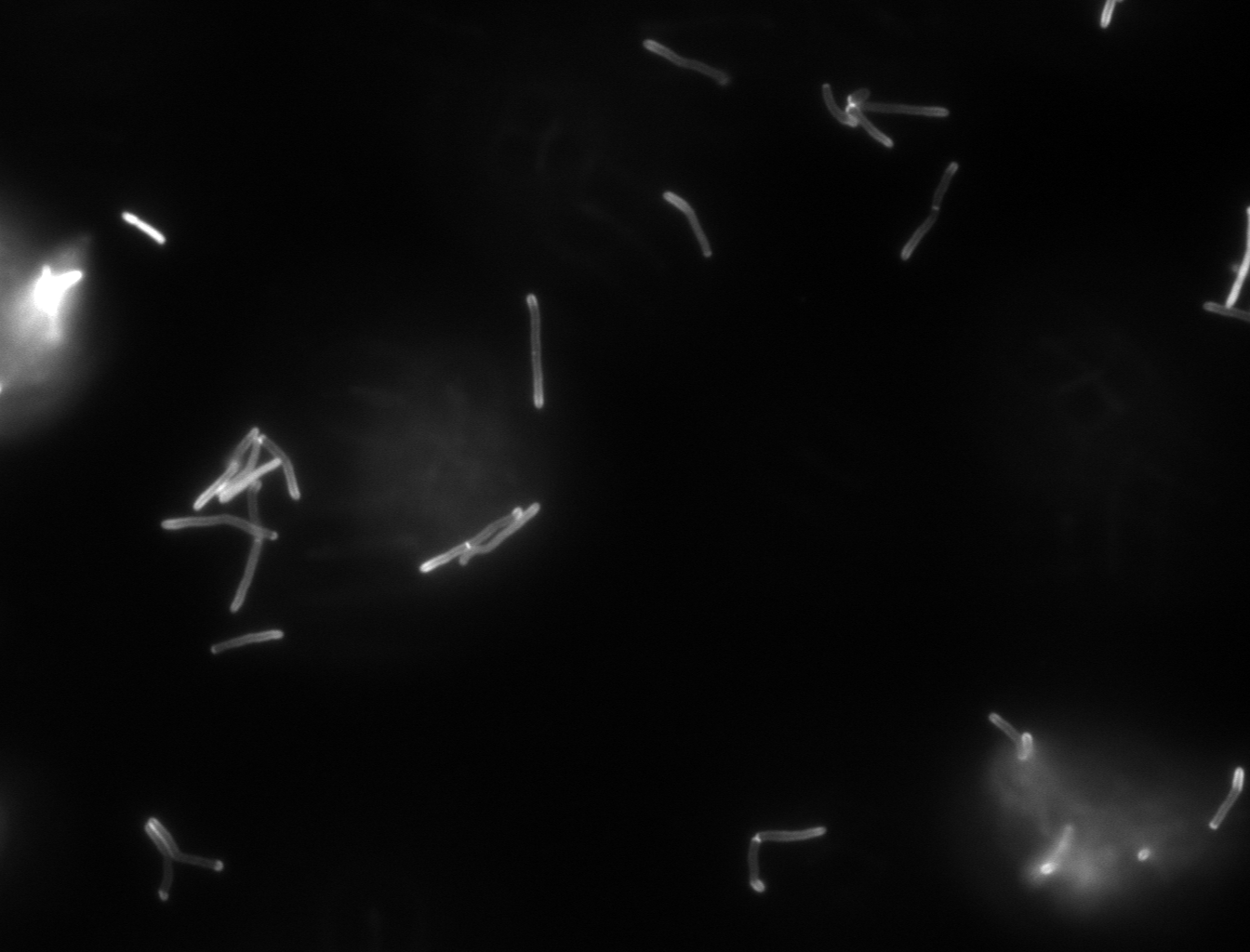

Supplement: Figure 2—source data 1. [file elife-37243-fig2-data1.zip › Figure 2 source data/Figure 2 source data-conventional microscopy (AlkDa + RADA)/2. AlkDa/2.tif]

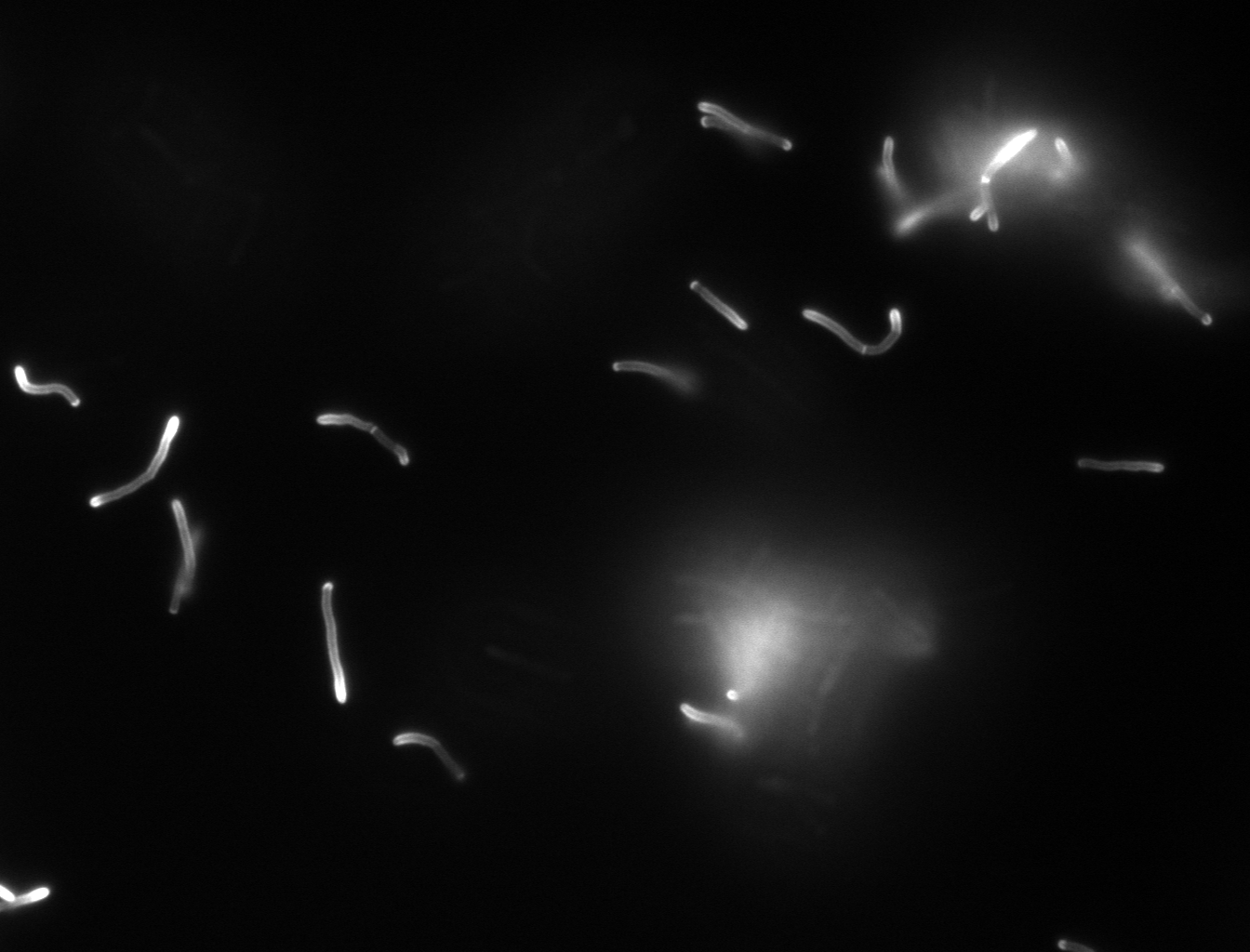

Supplement: Figure 2—source data 1. [file elife-37243-fig2-data1.zip › Figure 2 source data/Figure 2 source data-conventional microscopy (AlkDa + RADA)/2. AlkDa/3.tif]

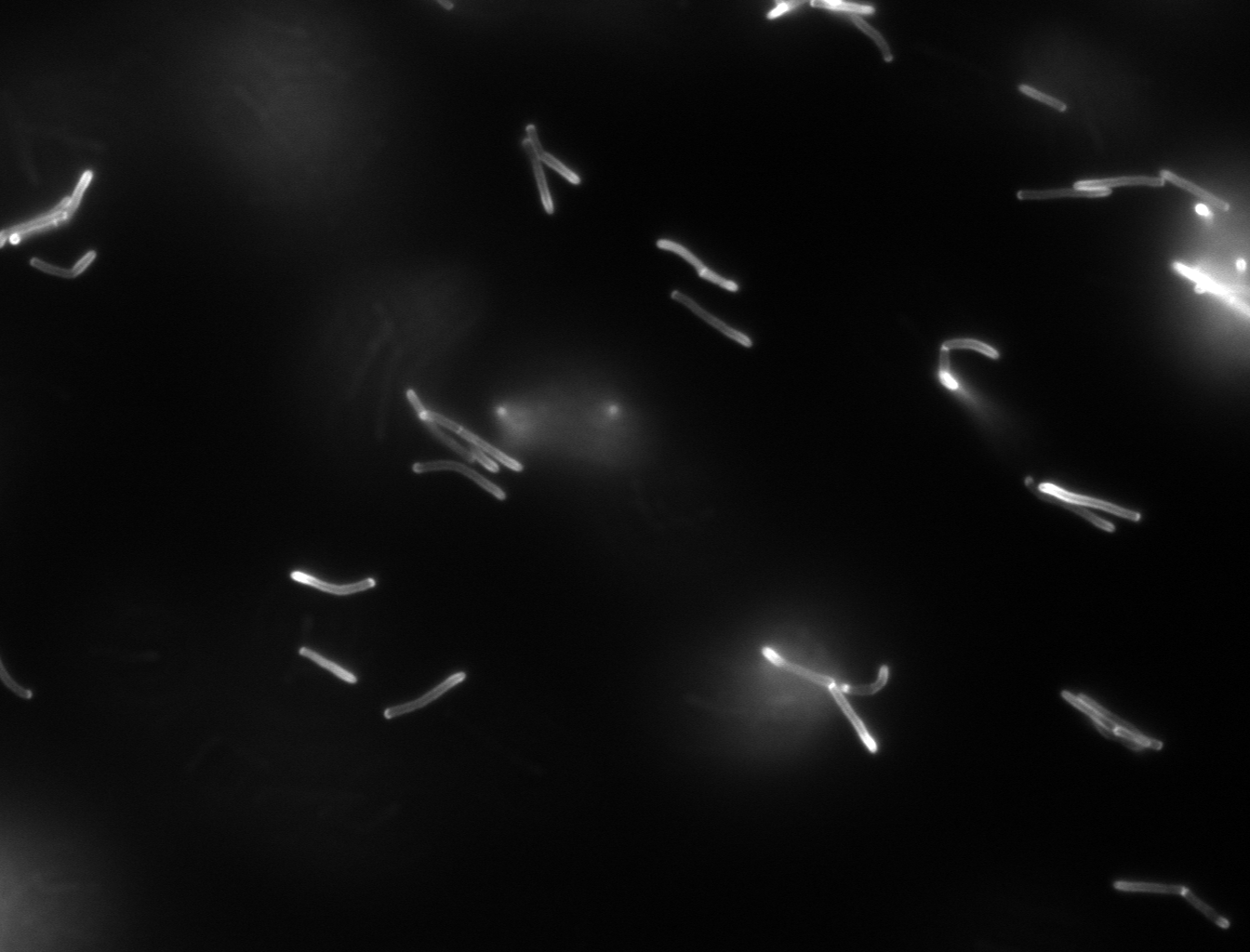

Supplement: Figure 2—source data 1. [file elife-37243-fig2-data1.zip › Figure 2 source data/Figure 2 source data-conventional microscopy (AlkDa + RADA)/2. AlkDa/4.tif]

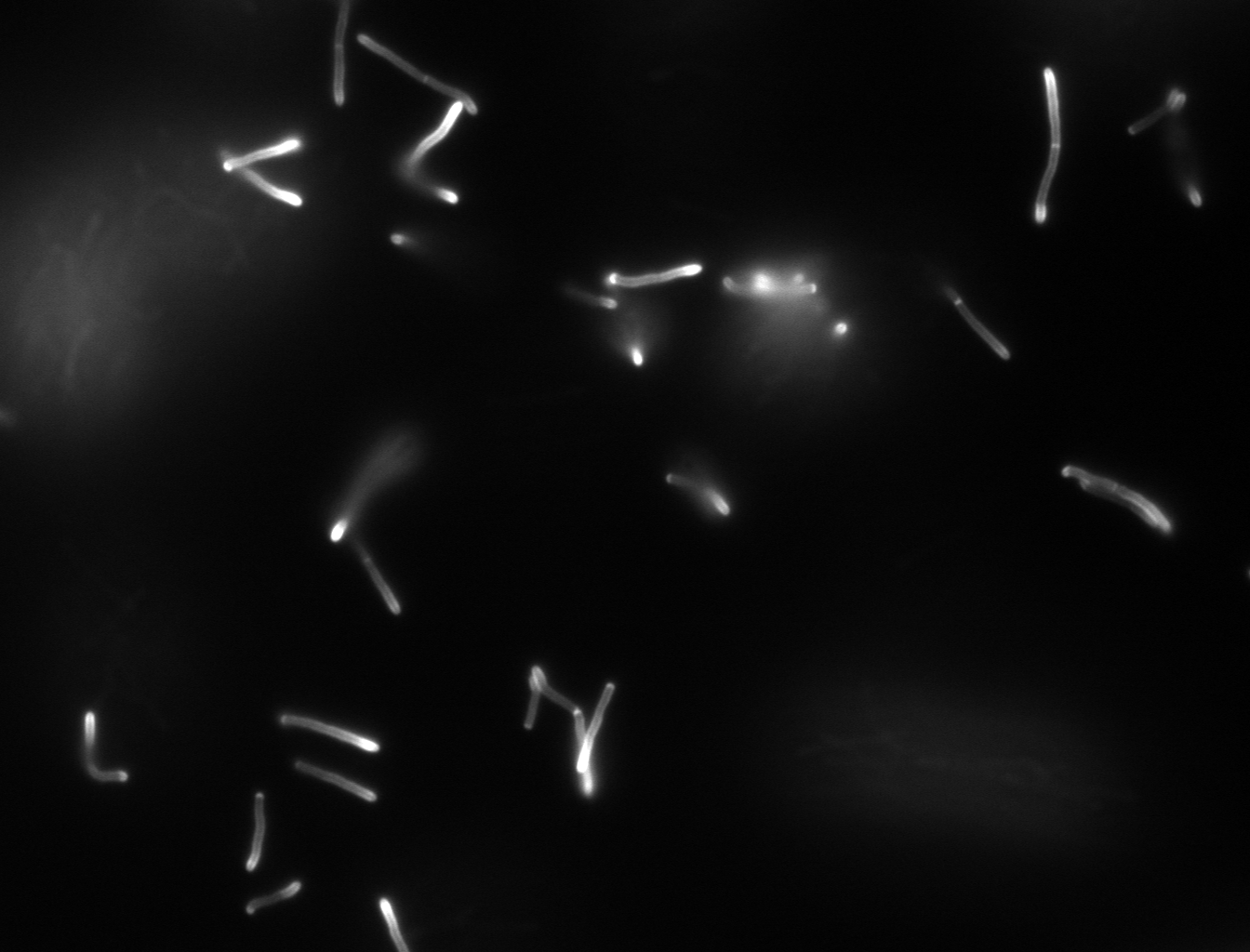

Supplement: Figure 2—source data 1. [file elife-37243-fig2-data1.zip › Figure 2 source data/Figure 2 source data-conventional microscopy (AlkDa + RADA)/2. AlkDa/5.tif]

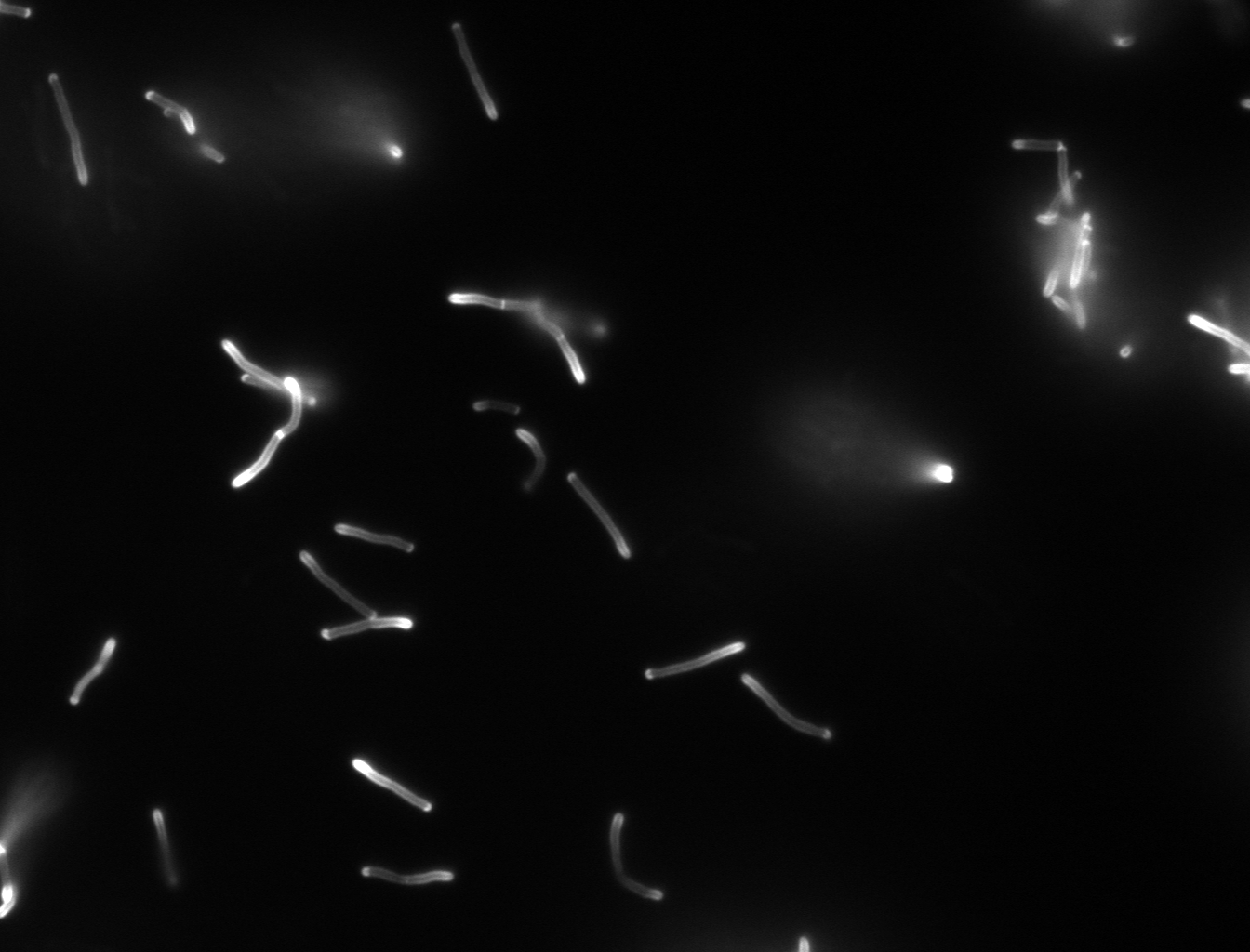

Supplement: Figure 2—source data 1. [file elife-37243-fig2-data1.zip › Figure 2 source data/Figure 2 source data-conventional microscopy (AlkDa + RADA)/2. AlkDa/6.tif]

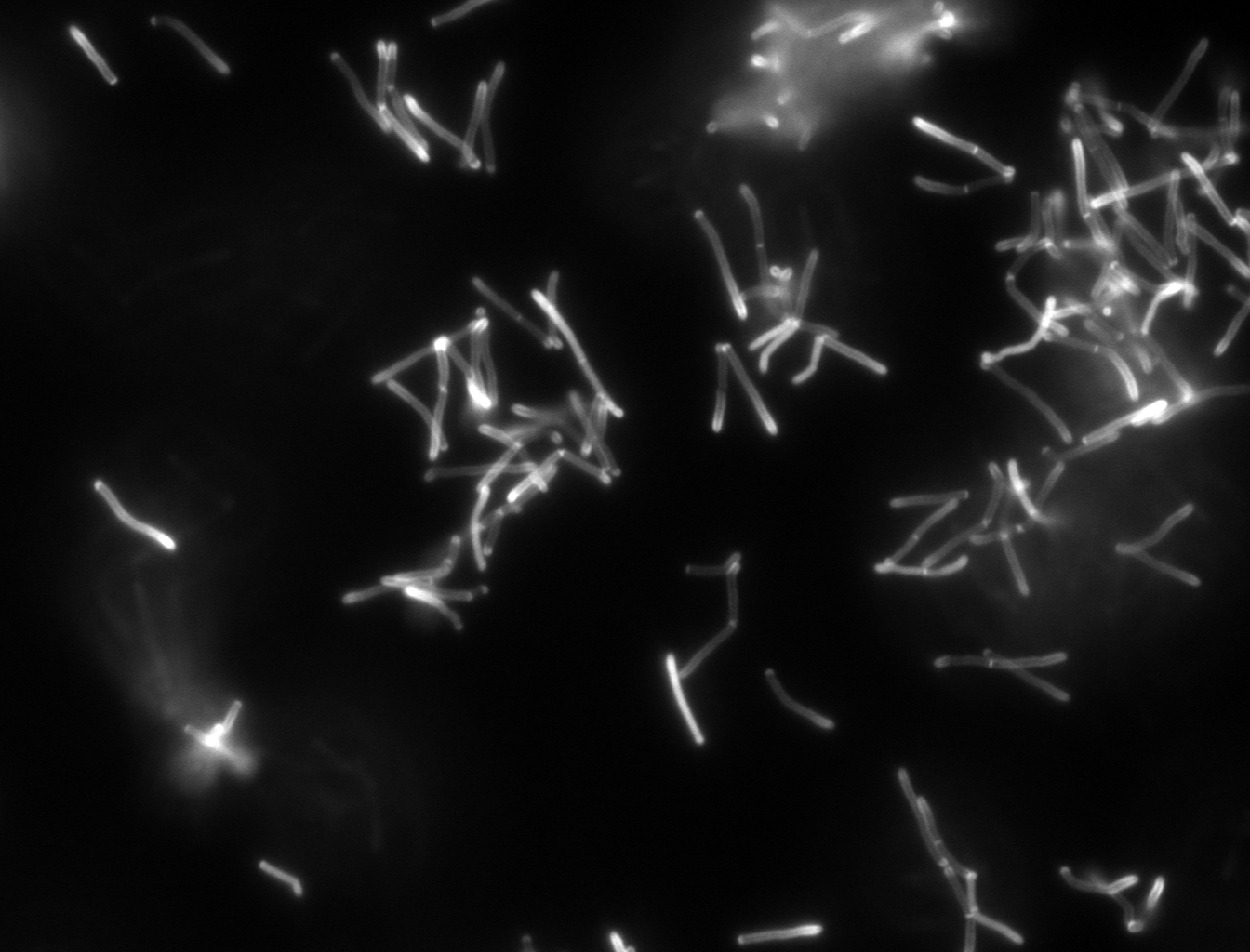

Supplement: Figure 2—source data 1. [file elife-37243-fig2-data1.zip › Figure 2 source data/Figure 2 source data-conventional microscopy (AlkDa + RADA)/2. AlkDa/7.tif]

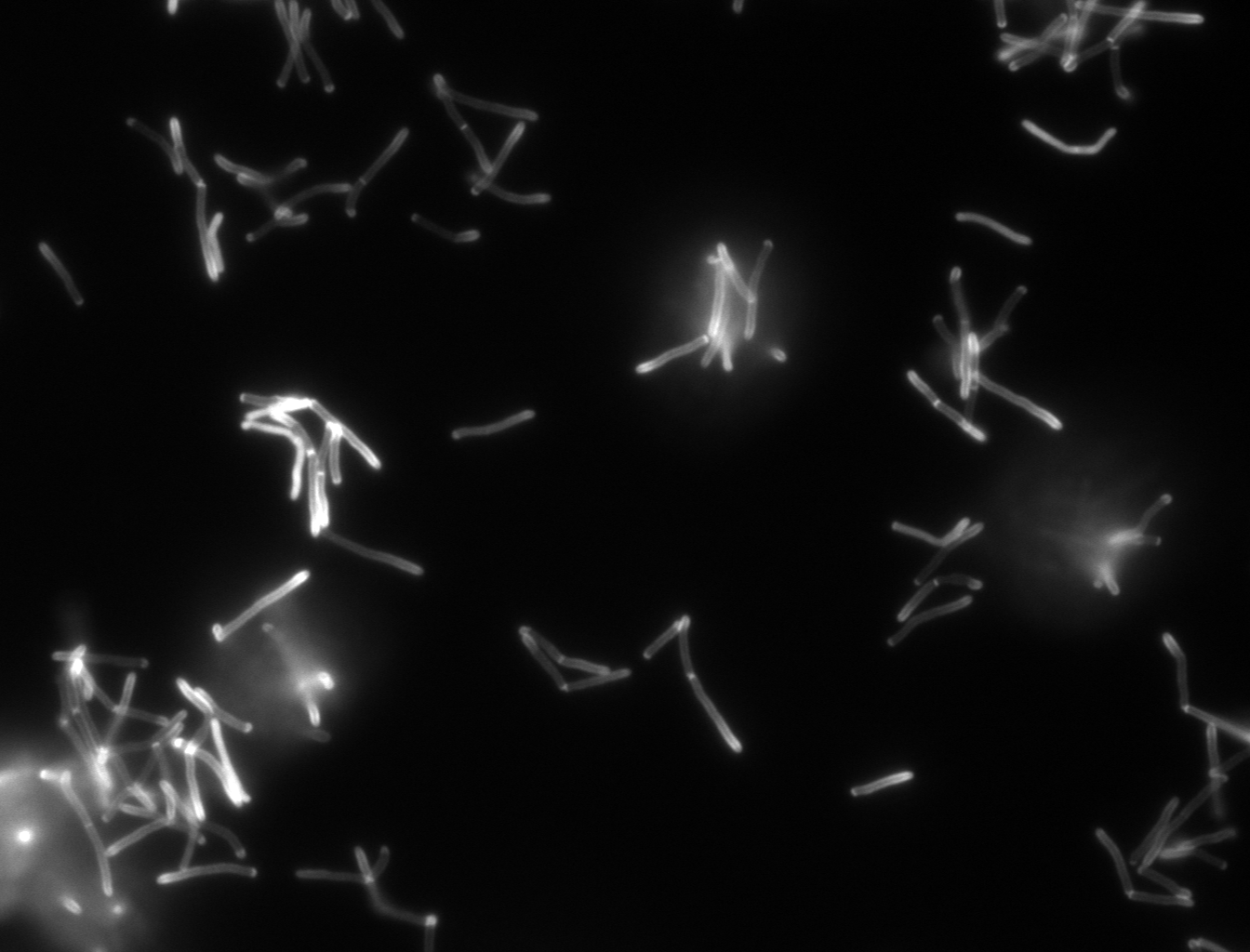

Supplement: Figure 2—source data 1. [file elife-37243-fig2-data1.zip › Figure 2 source data/Figure 2 source data-conventional microscopy (AlkDa + RADA)/2. AlkDa/8.tif]

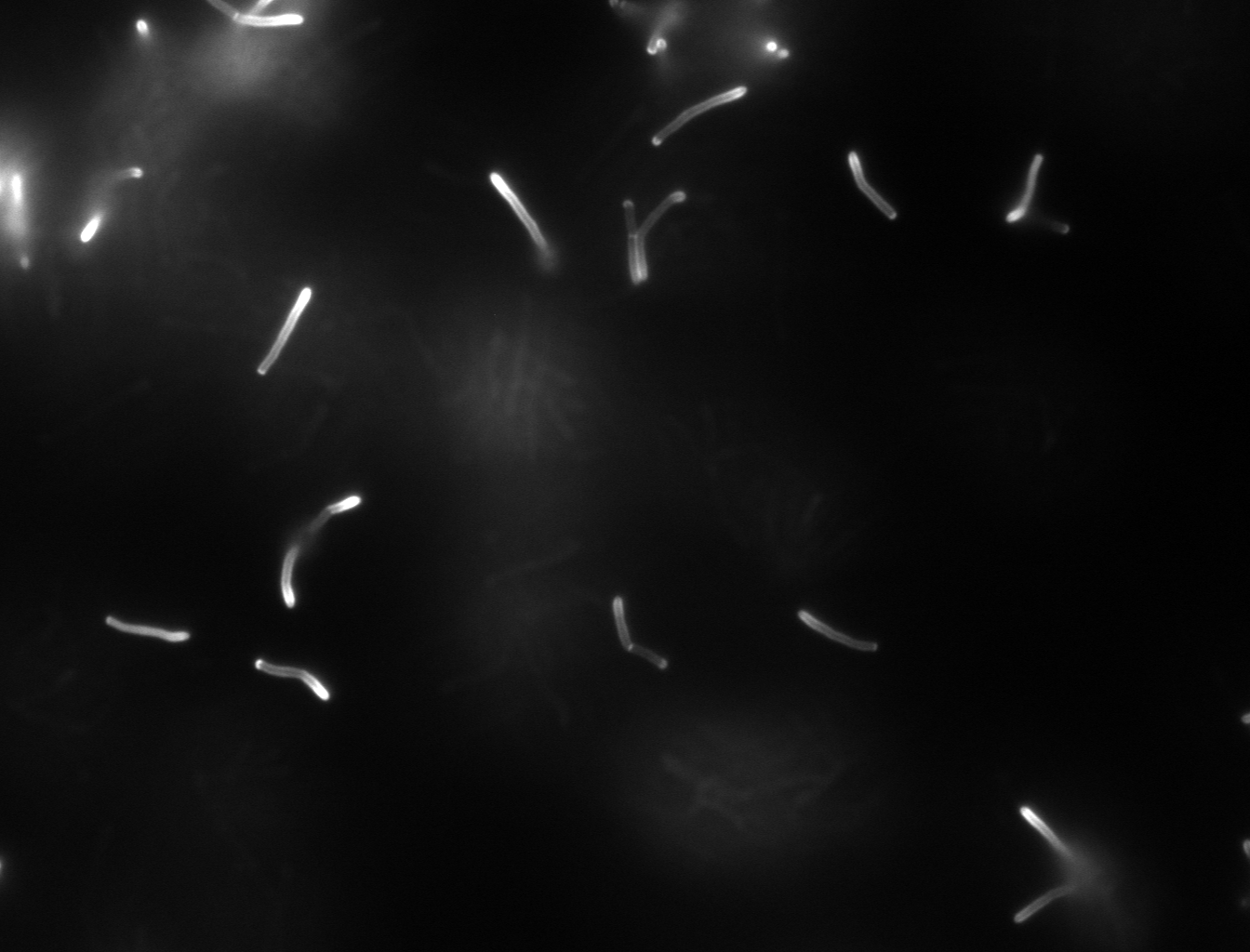

Supplement: Figure 2—source data 1. [file elife-37243-fig2-data1.zip › Figure 2 source data/Figure 2 source data-conventional microscopy (AlkDa + RADA)/2. AlkDa/9.tif]

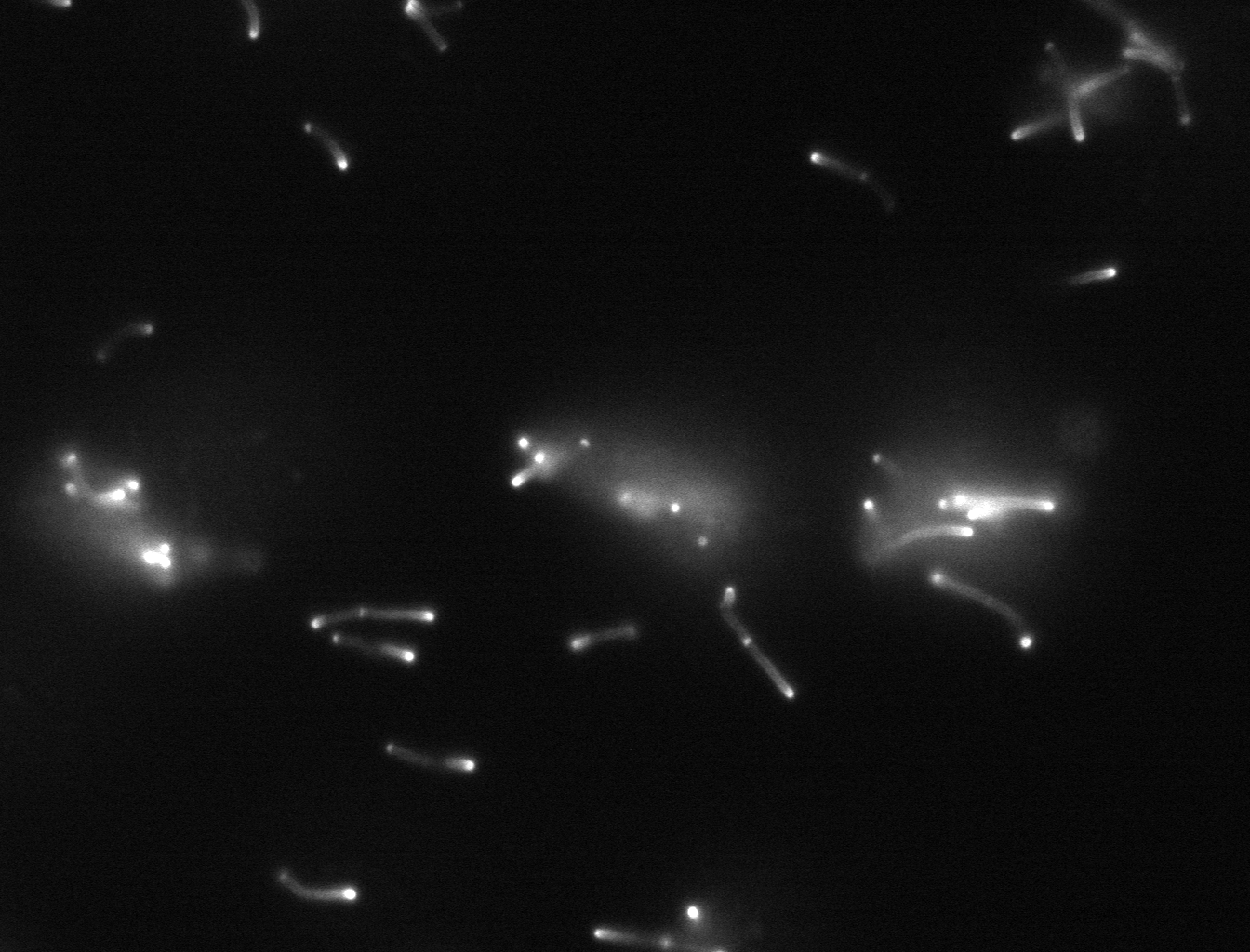

Supplement: Figure 2—source data 1. [file elife-37243-fig2-data1.zip › Figure 2 source data/Figure 2 source data-conventional microscopy (AlkDa + RADA)/3. RADA/1.tif]

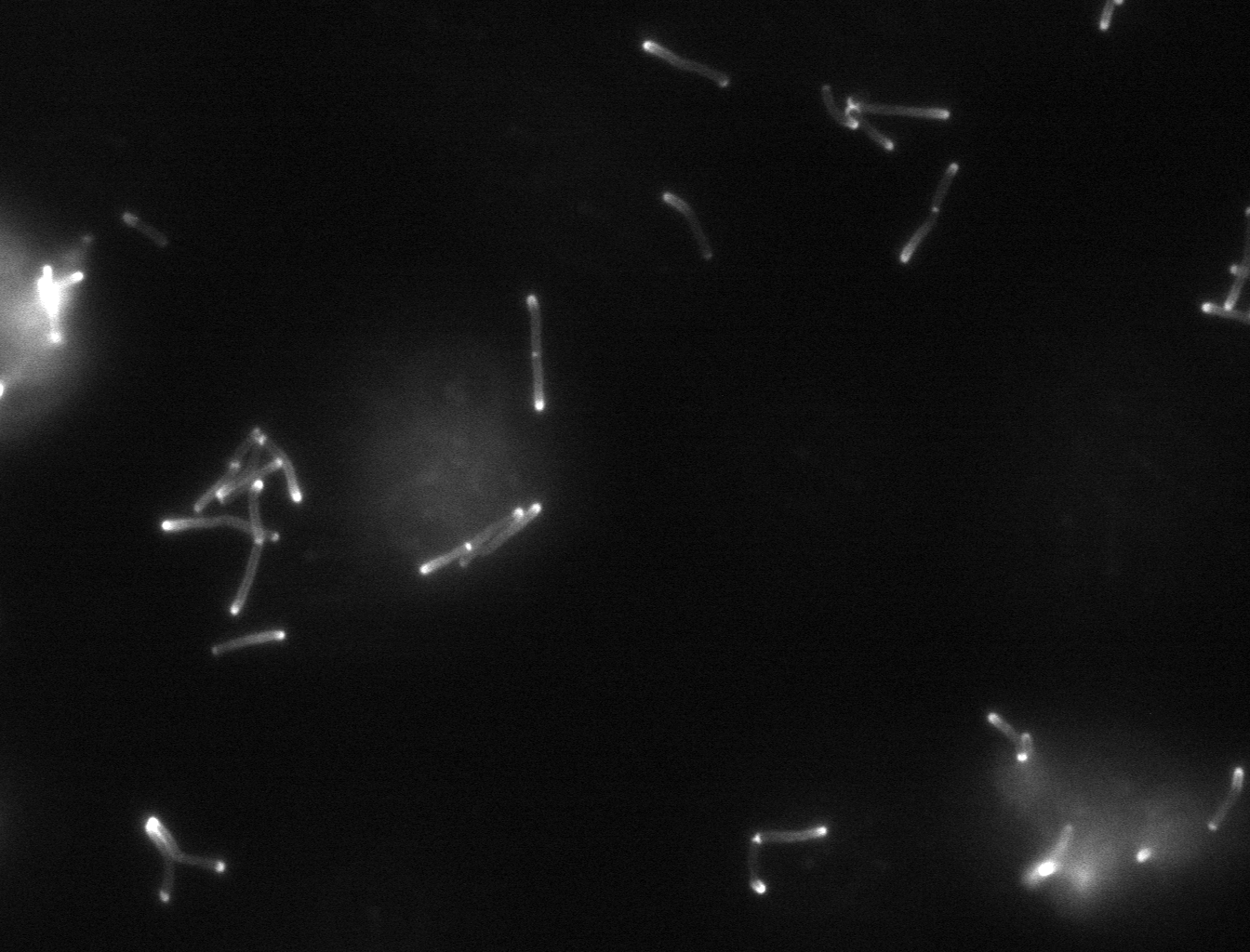

Supplement: Figure 2—source data 1. [file elife-37243-fig2-data1.zip › Figure 2 source data/Figure 2 source data-conventional microscopy (AlkDa + RADA)/3. RADA/2.tif]

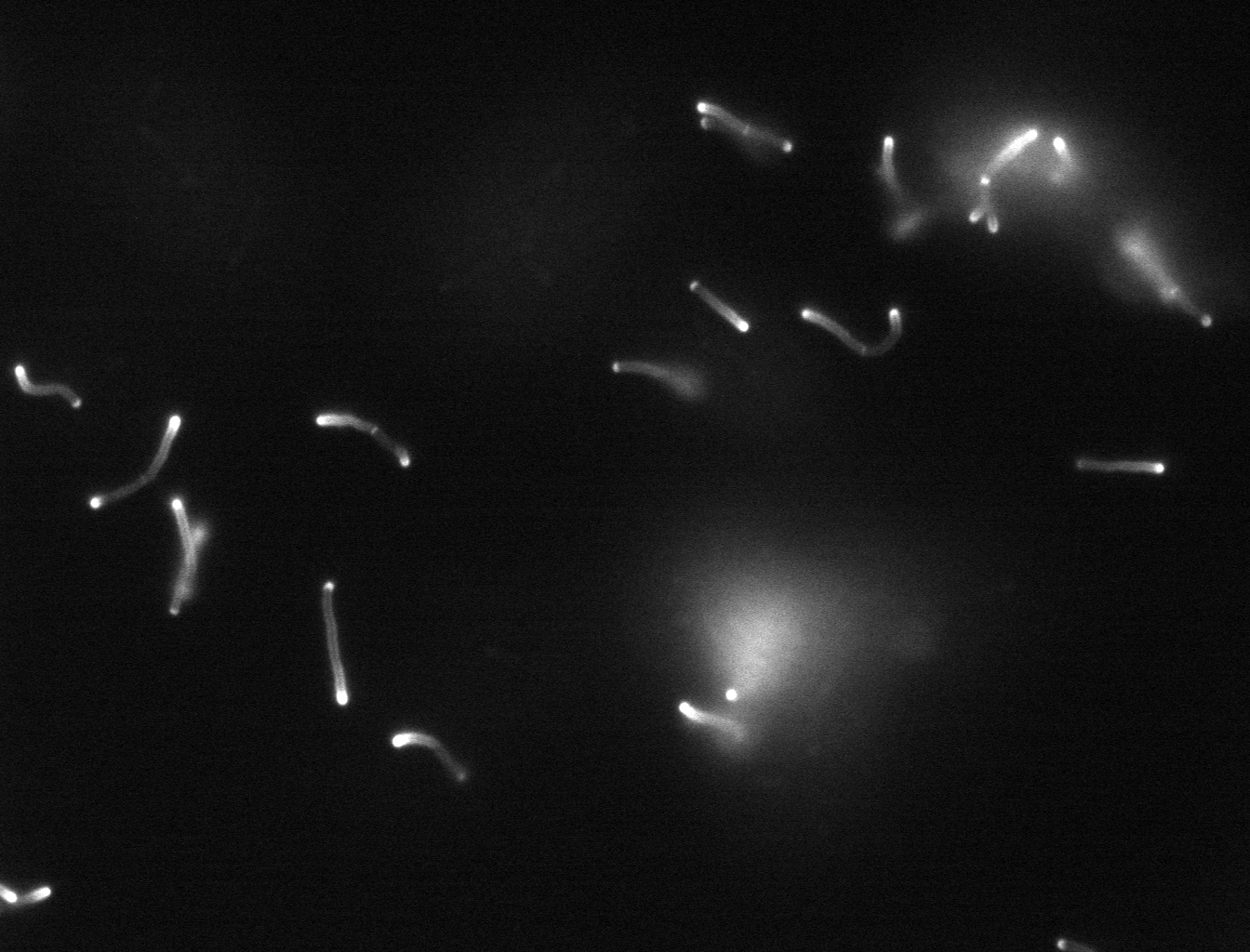

Supplement: Figure 2—source data 1. [file elife-37243-fig2-data1.zip › Figure 2 source data/Figure 2 source data-conventional microscopy (AlkDa + RADA)/3. RADA/3.tif]

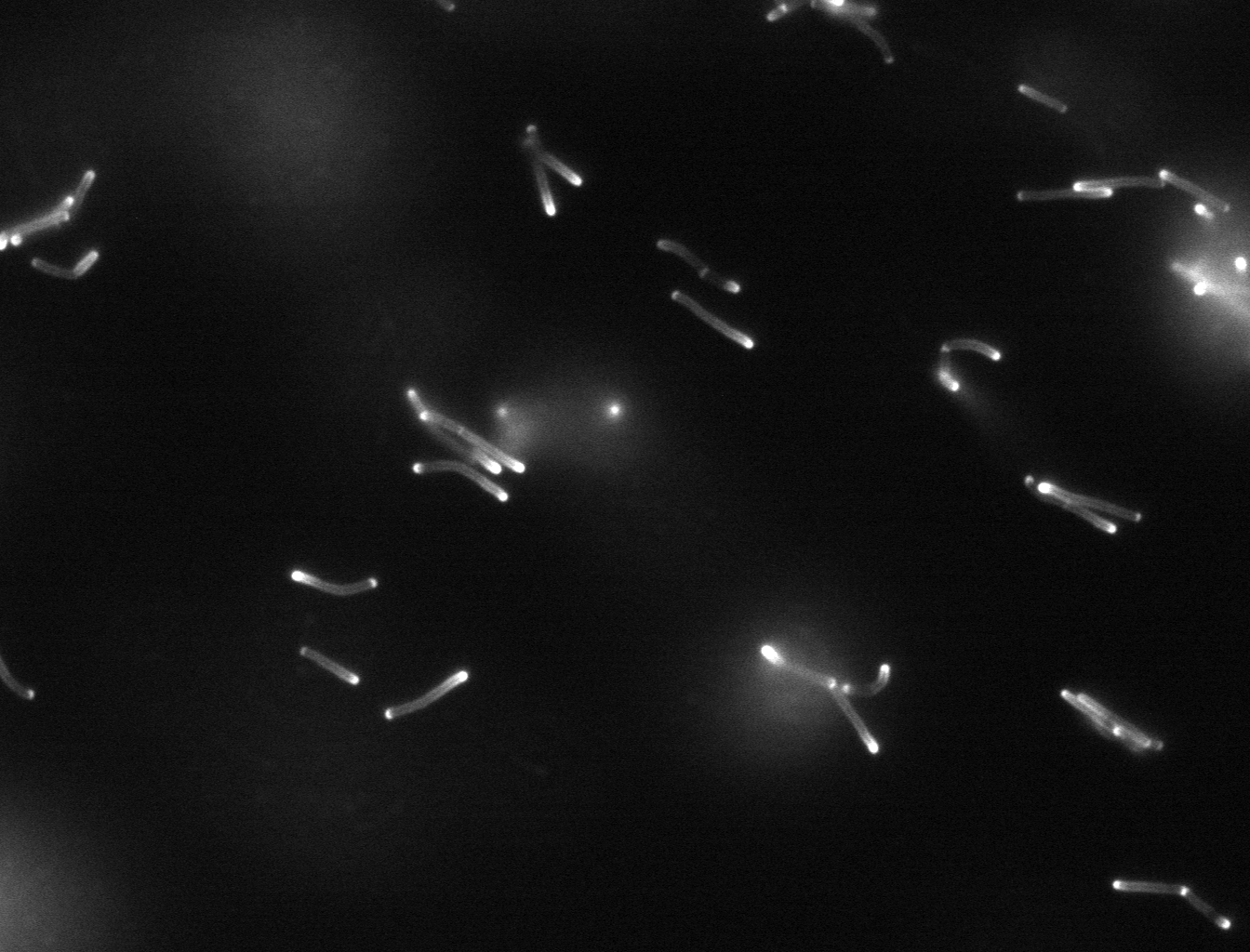

Supplement: Figure 2—source data 1. [file elife-37243-fig2-data1.zip › Figure 2 source data/Figure 2 source data-conventional microscopy (AlkDa + RADA)/3. RADA/4.tif]

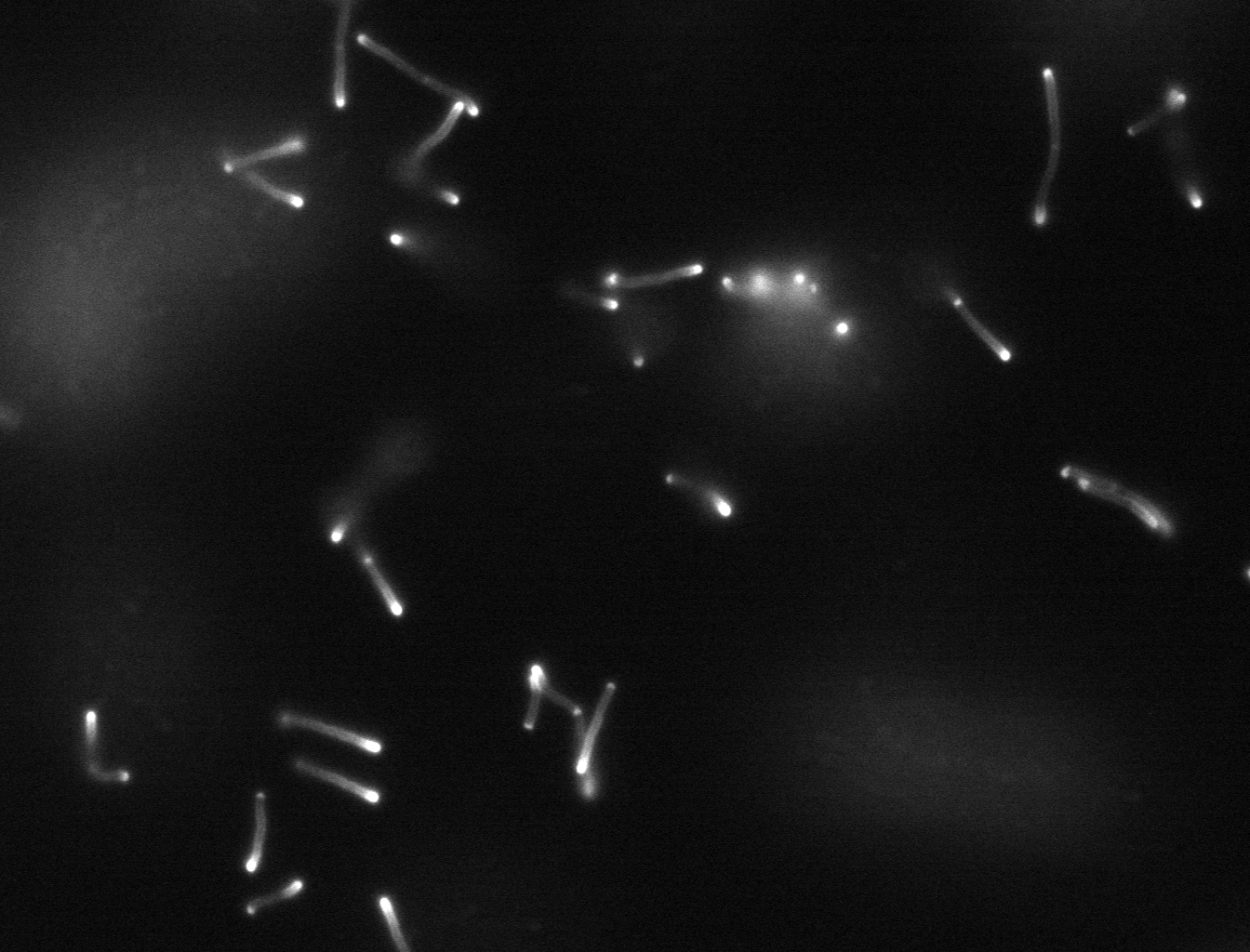

Supplement: Figure 2—source data 1. [file elife-37243-fig2-data1.zip › Figure 2 source data/Figure 2 source data-conventional microscopy (AlkDa + RADA)/3. RADA/5.tif]

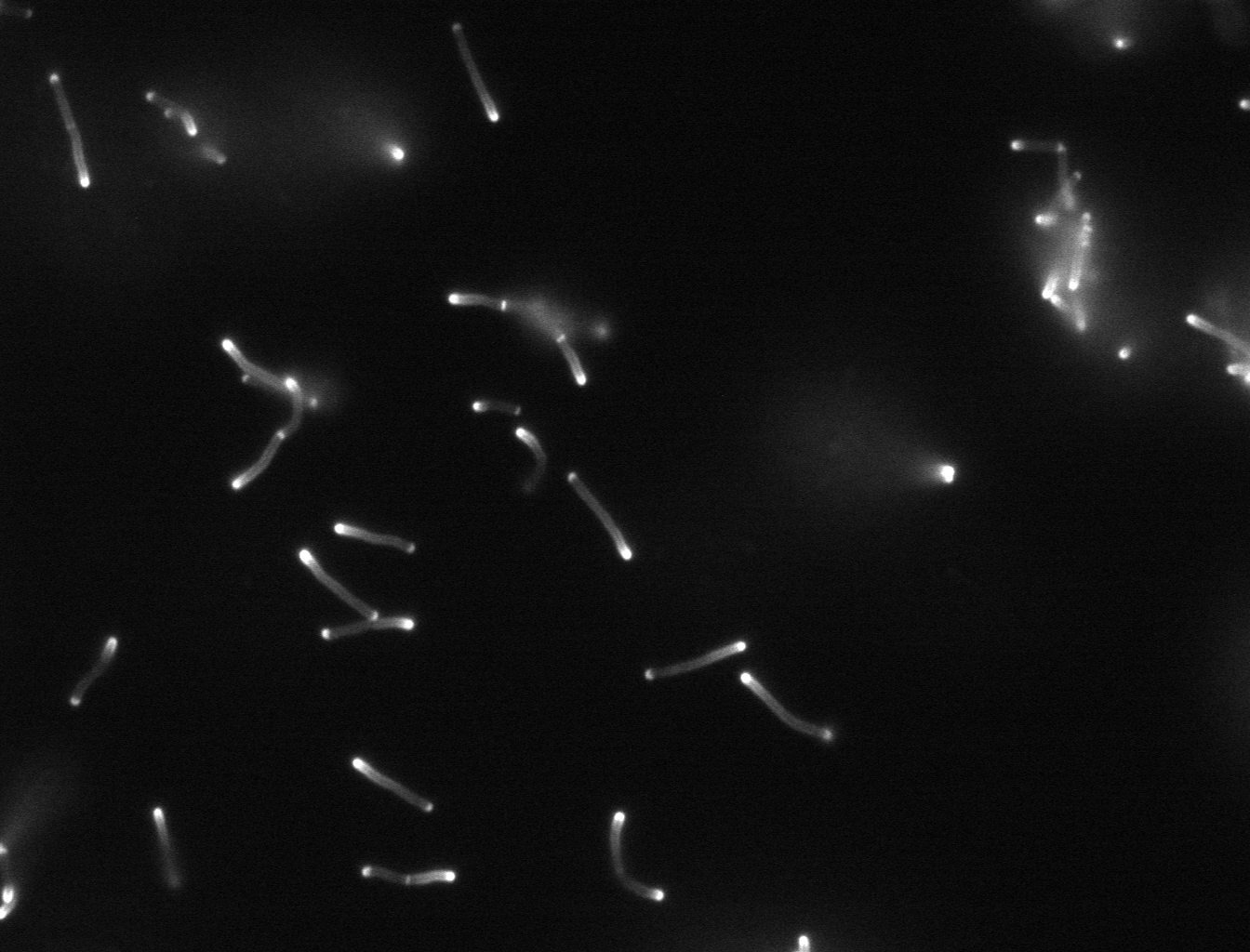

Supplement: Figure 2—source data 1. [file elife-37243-fig2-data1.zip › Figure 2 source data/Figure 2 source data-conventional microscopy (AlkDa + RADA)/3. RADA/6.tif]

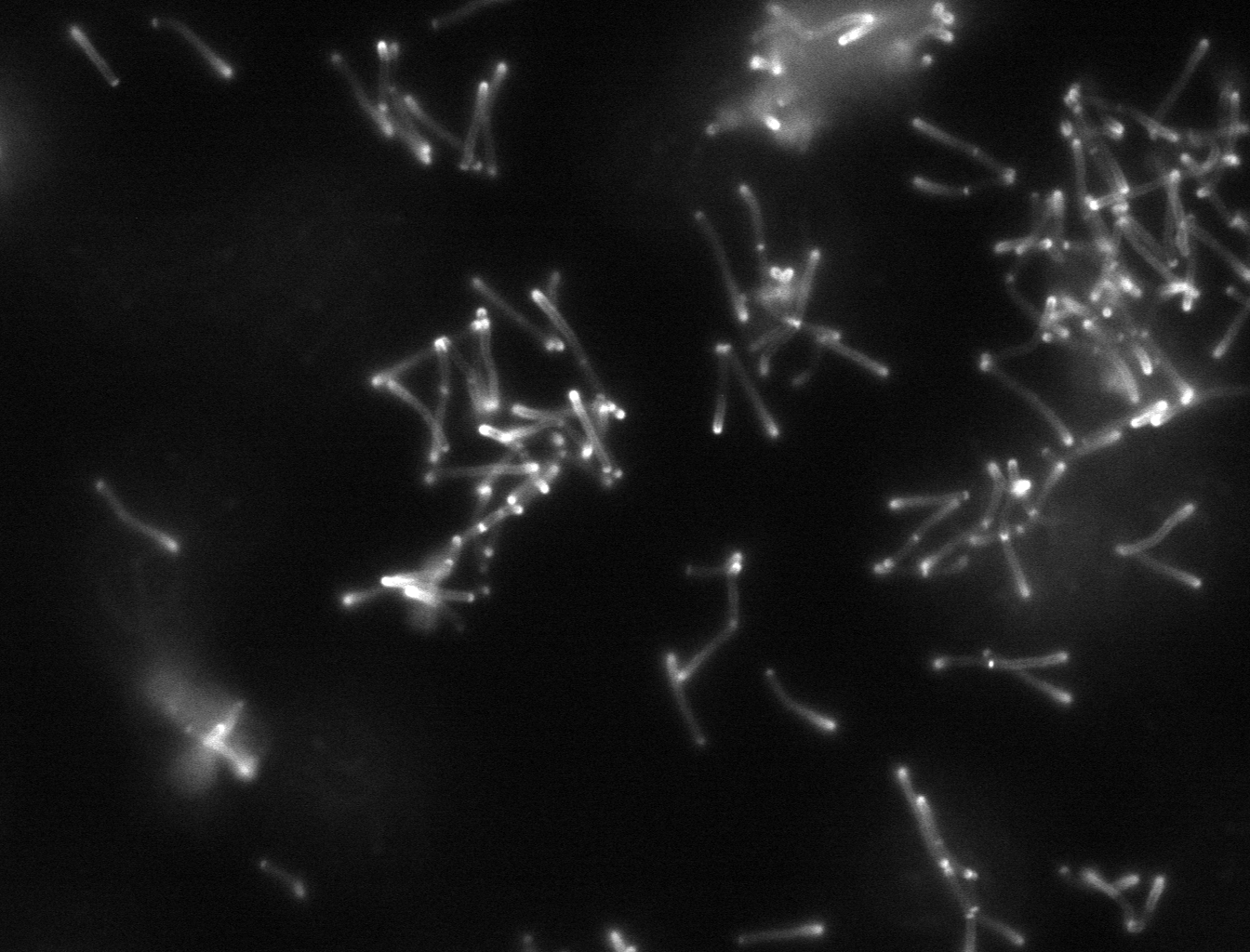

Supplement: Figure 2—source data 1. [file elife-37243-fig2-data1.zip › Figure 2 source data/Figure 2 source data-conventional microscopy (AlkDa + RADA)/3. RADA/7.tif]

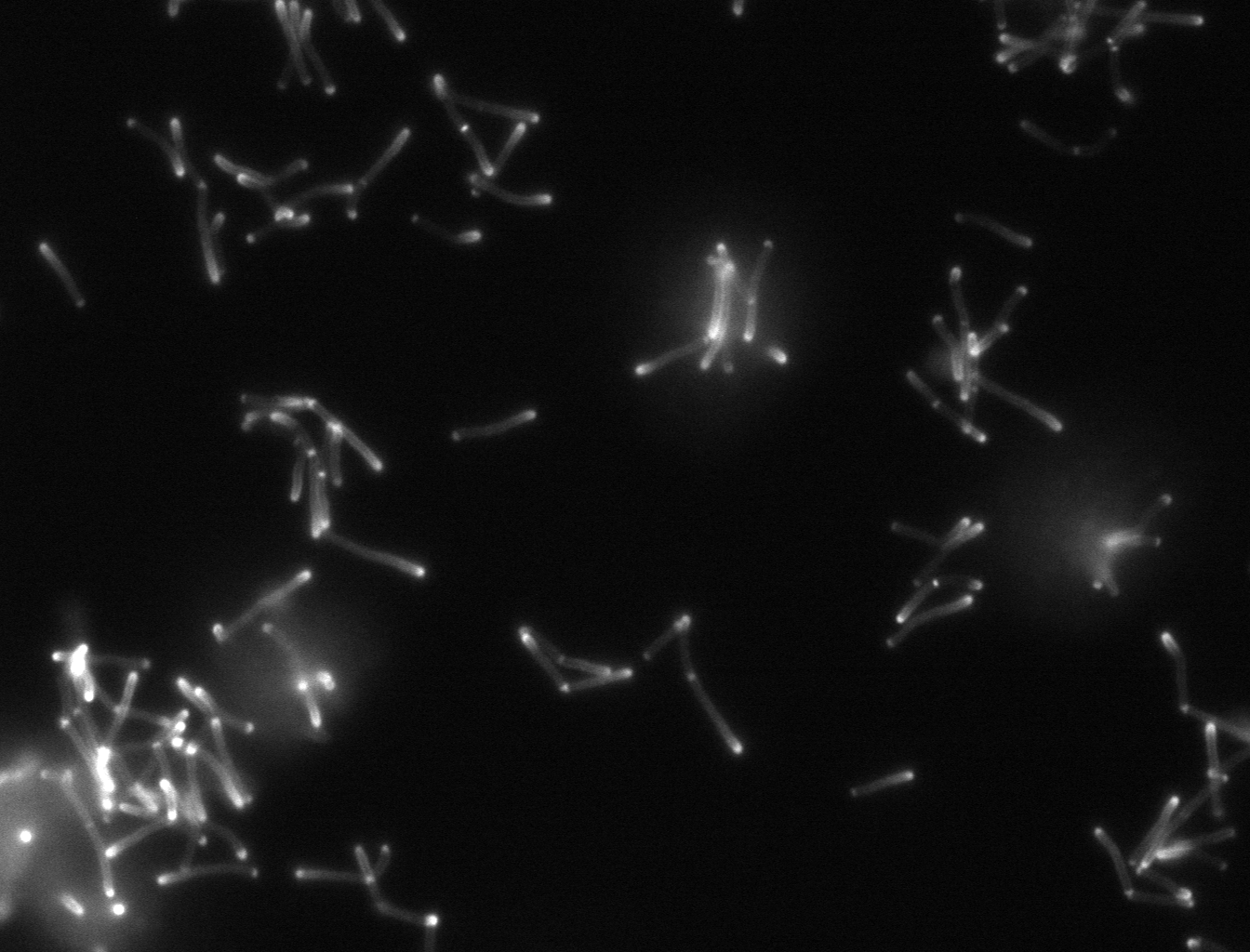

Supplement: Figure 2—source data 1. [file elife-37243-fig2-data1.zip › Figure 2 source data/Figure 2 source data-conventional microscopy (AlkDa + RADA)/3. RADA/8.tif]

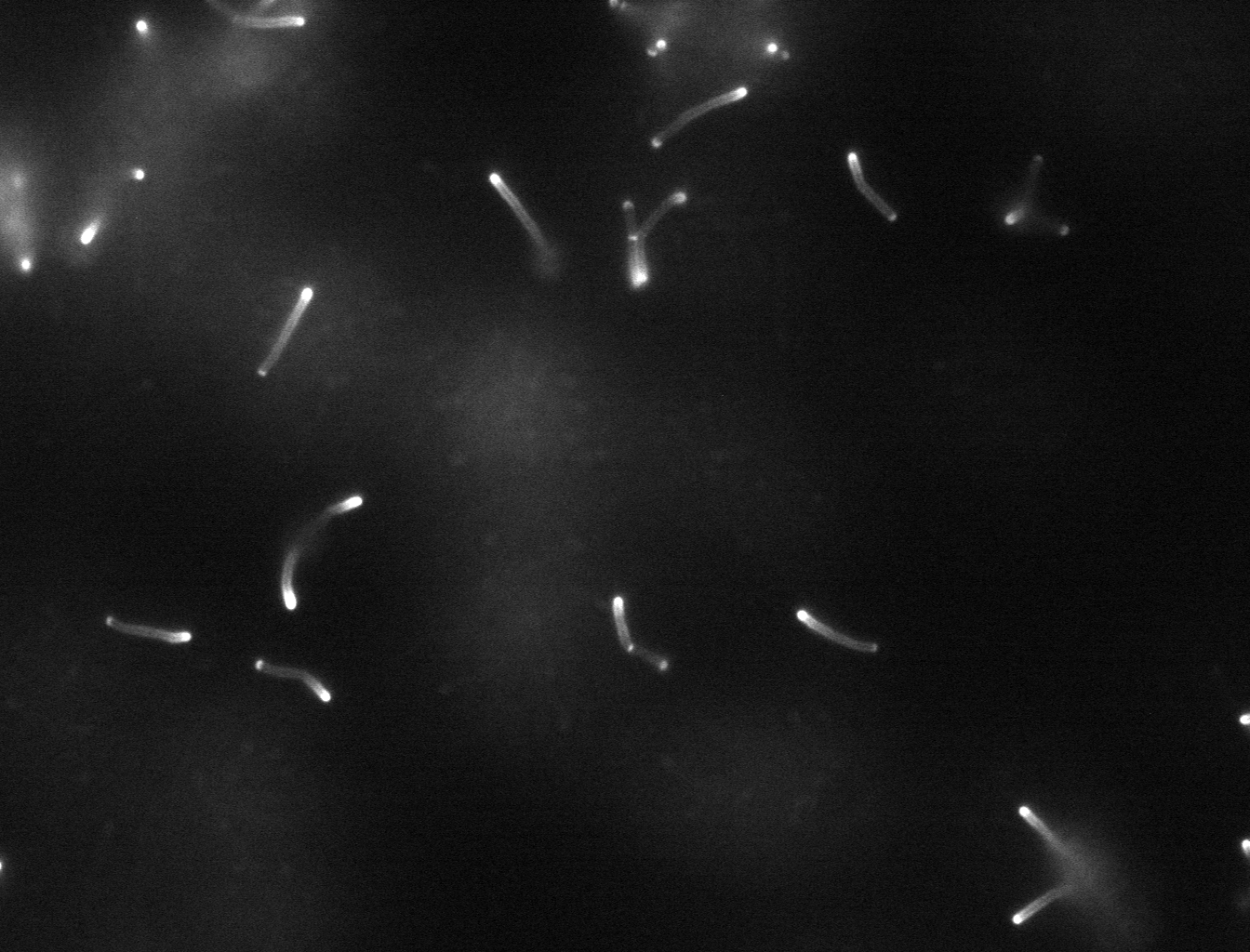

Supplement: Figure 2—source data 1. [file elife-37243-fig2-data1.zip › Figure 2 source data/Figure 2 source data-conventional microscopy (AlkDa + RADA)/3. RADA/9.tif]

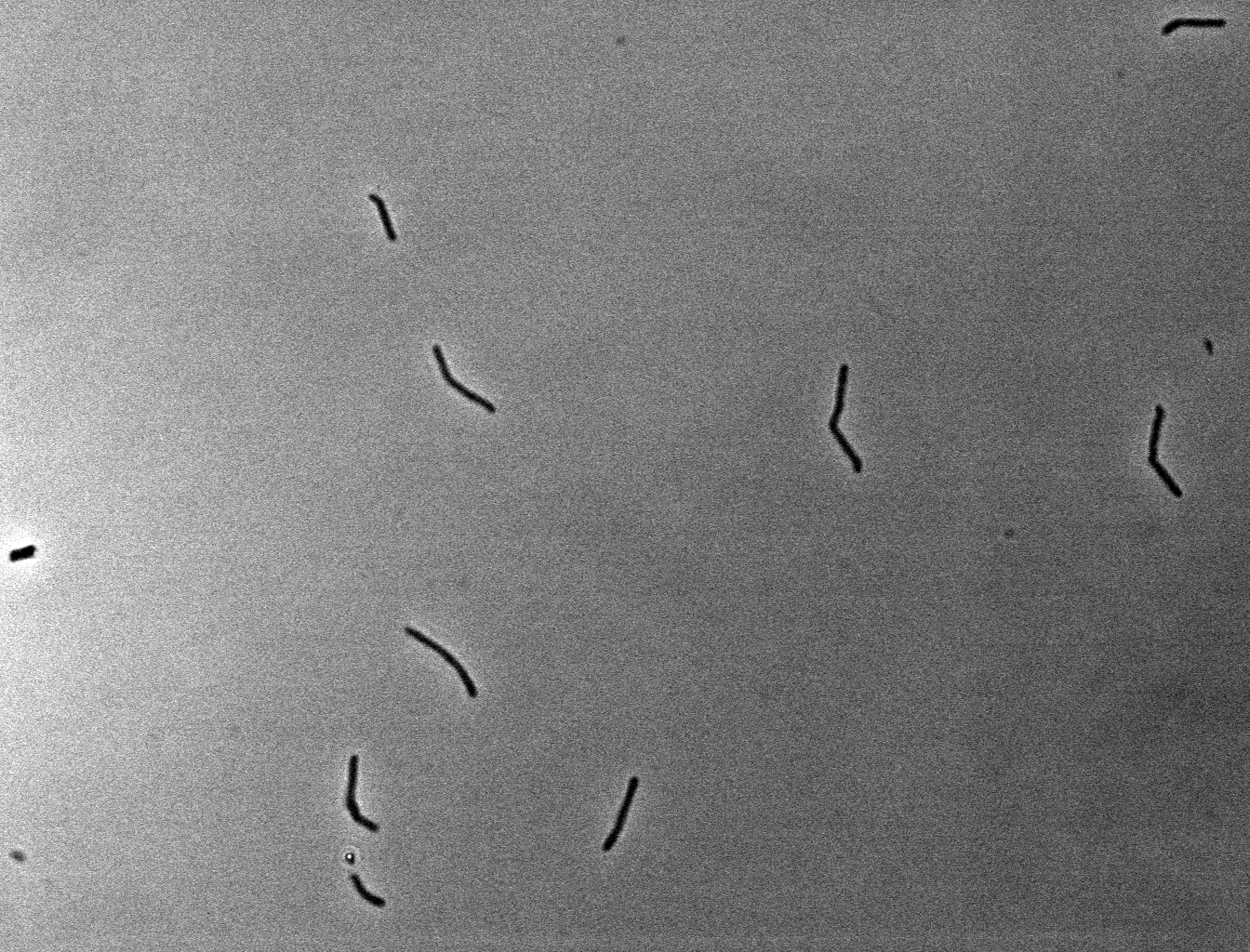

Supplement: Figure 2—source data 1. [file elife-37243-fig2-data1.zip › Figure 2 source data/Figure 2 source data-conventional microscopy (alkDD + RADA)/1. Phase/1.tif]

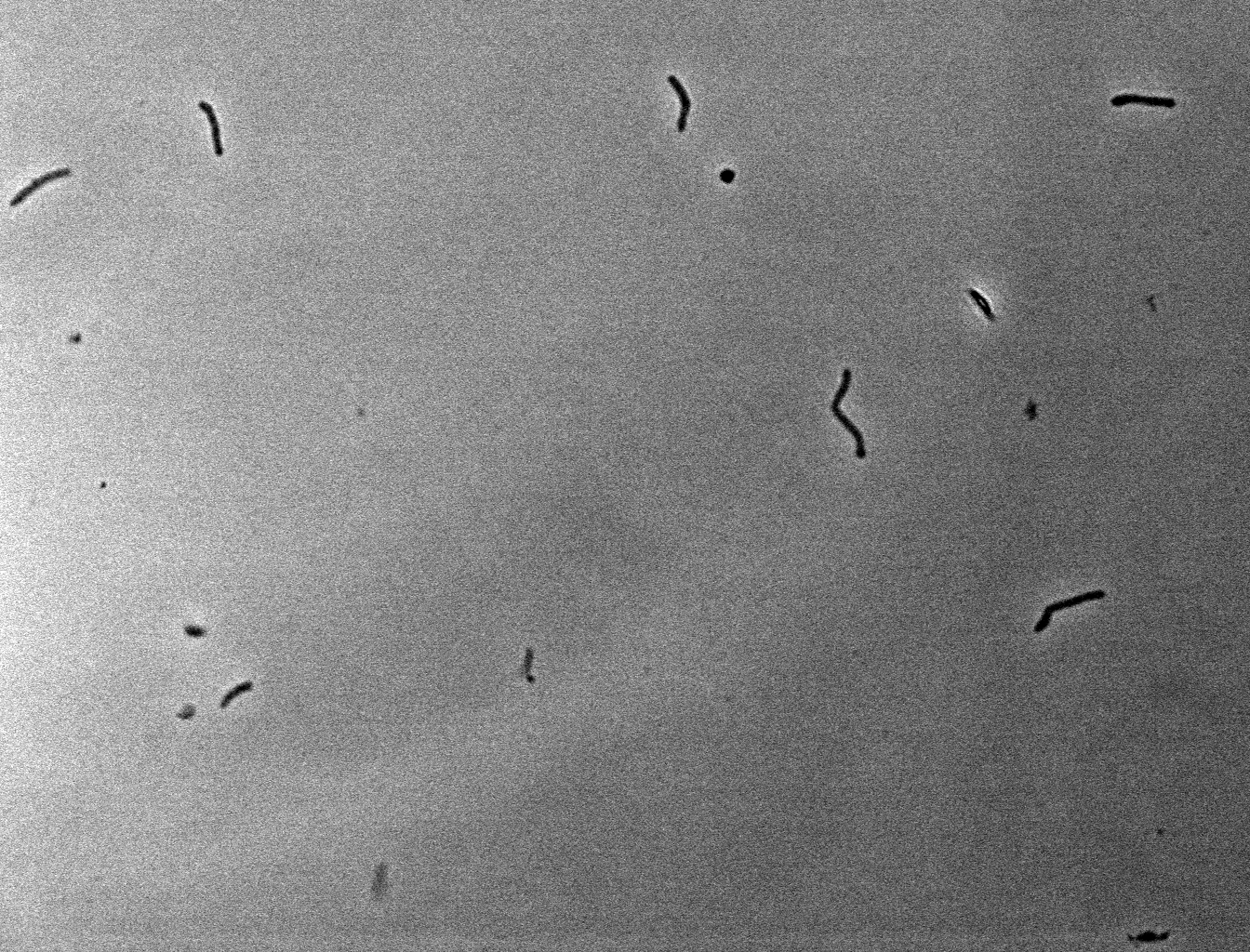

Supplement: Figure 2—source data 1. [file elife-37243-fig2-data1.zip › Figure 2 source data/Figure 2 source data-conventional microscopy (alkDD + RADA)/1. Phase/10.tif]

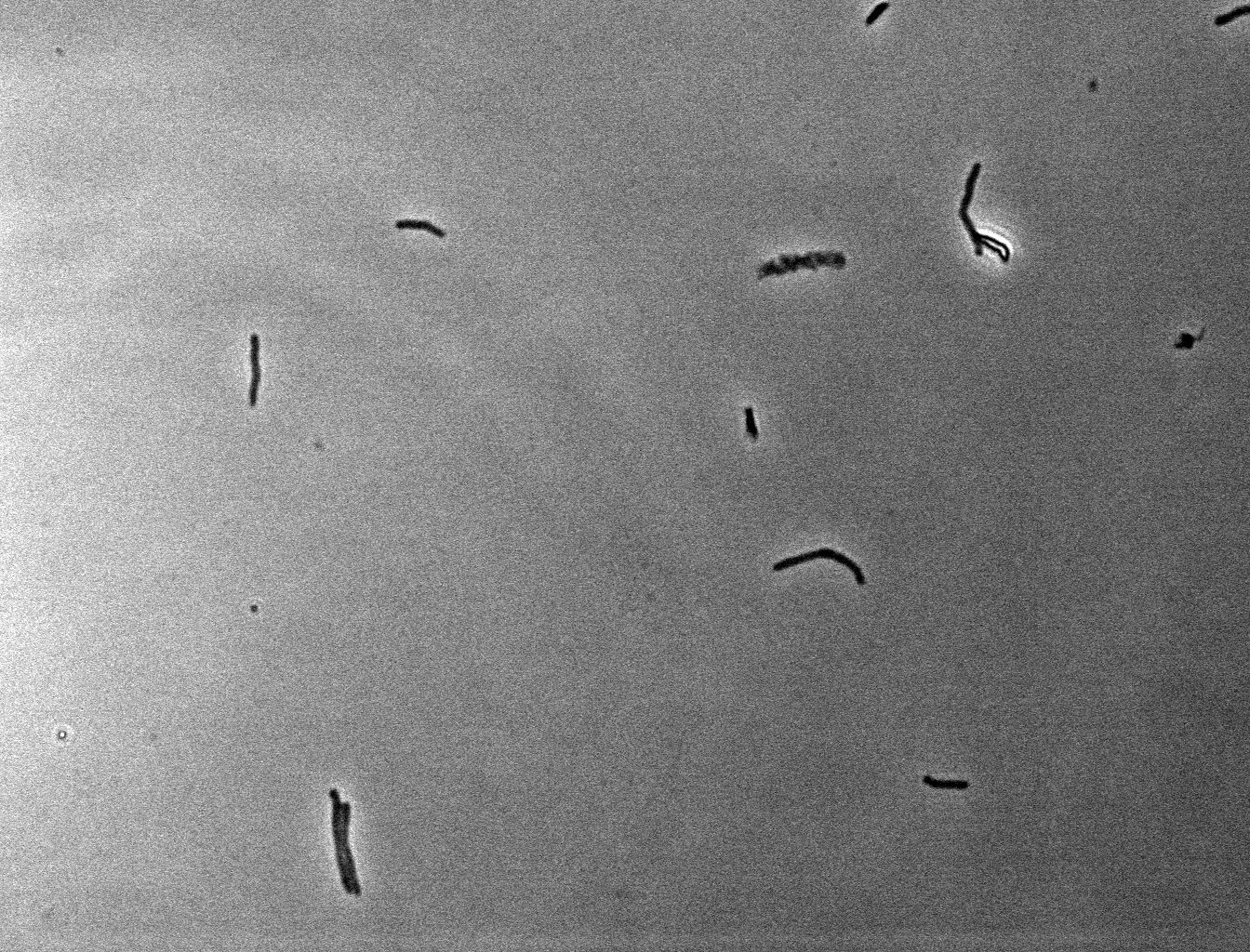

Supplement: Figure 2—source data 1. [file elife-37243-fig2-data1.zip › Figure 2 source data/Figure 2 source data-conventional microscopy (alkDD + RADA)/1. Phase/2.tif]

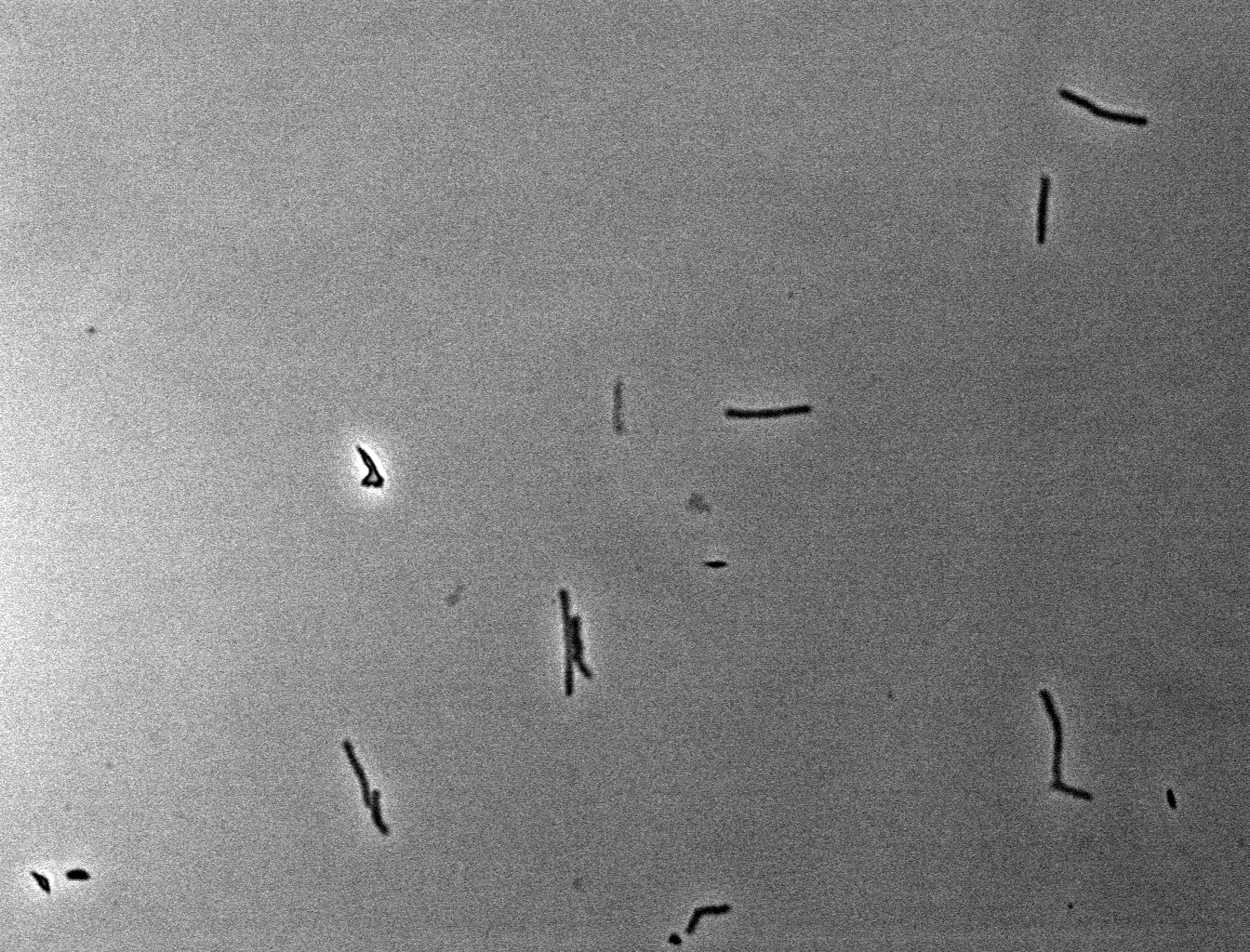

Supplement: Figure 2—source data 1. [file elife-37243-fig2-data1.zip › Figure 2 source data/Figure 2 source data-conventional microscopy (alkDD + RADA)/1. Phase/3.tif]

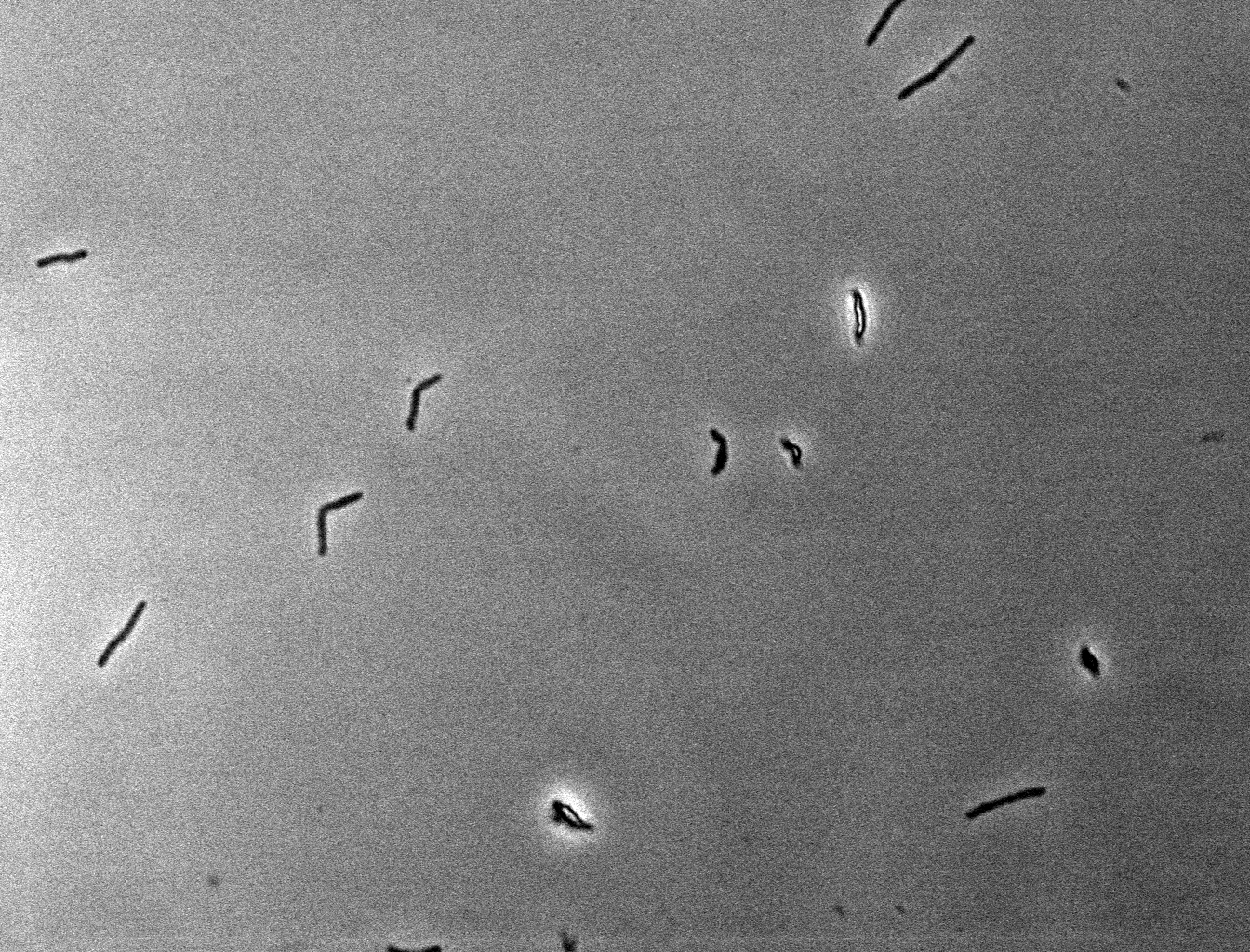

Supplement: Figure 2—source data 1. [file elife-37243-fig2-data1.zip › Figure 2 source data/Figure 2 source data-conventional microscopy (alkDD + RADA)/1. Phase/4.tif]

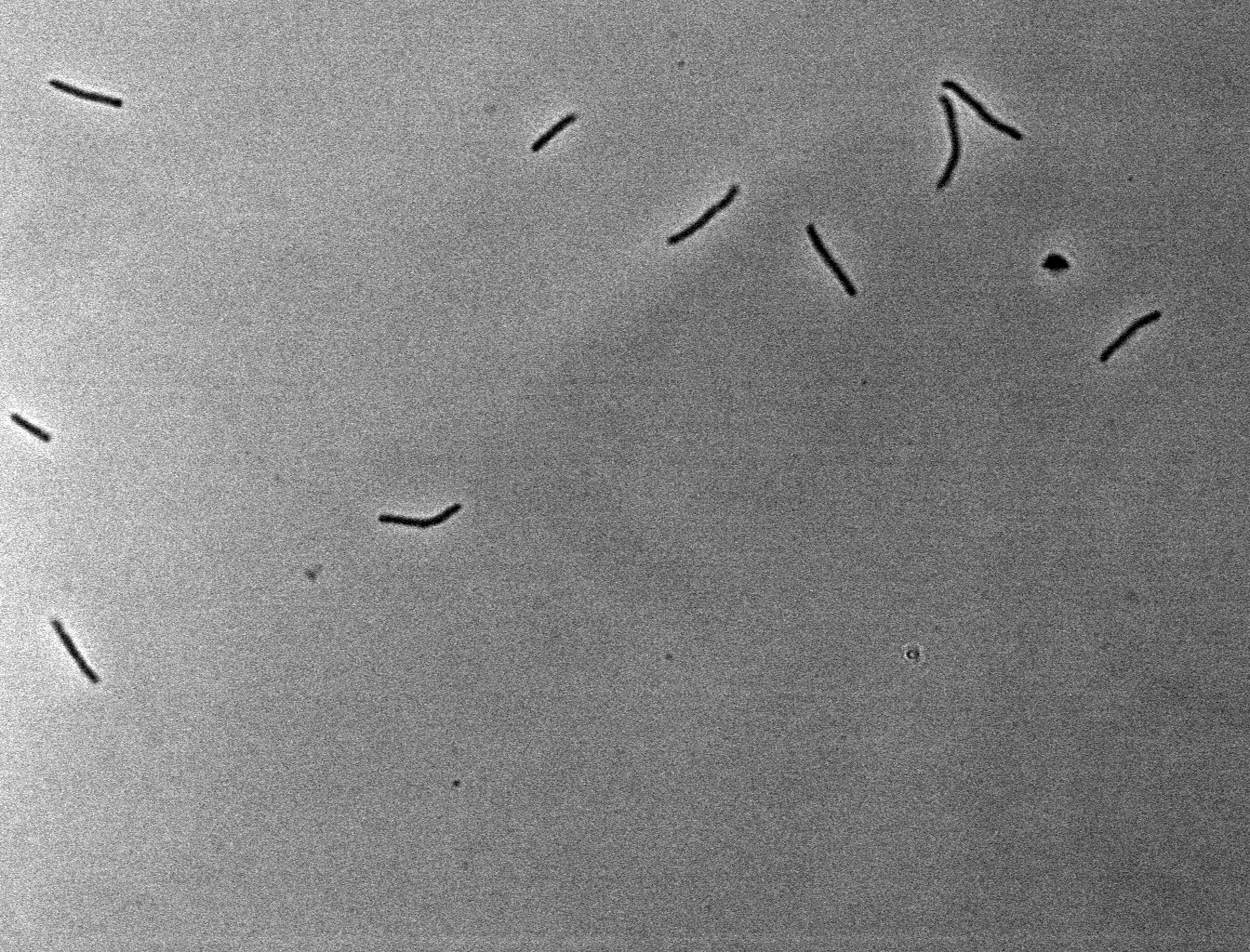

Supplement: Figure 2—source data 1. [file elife-37243-fig2-data1.zip › Figure 2 source data/Figure 2 source data-conventional microscopy (alkDD + RADA)/1. Phase/5.tif]

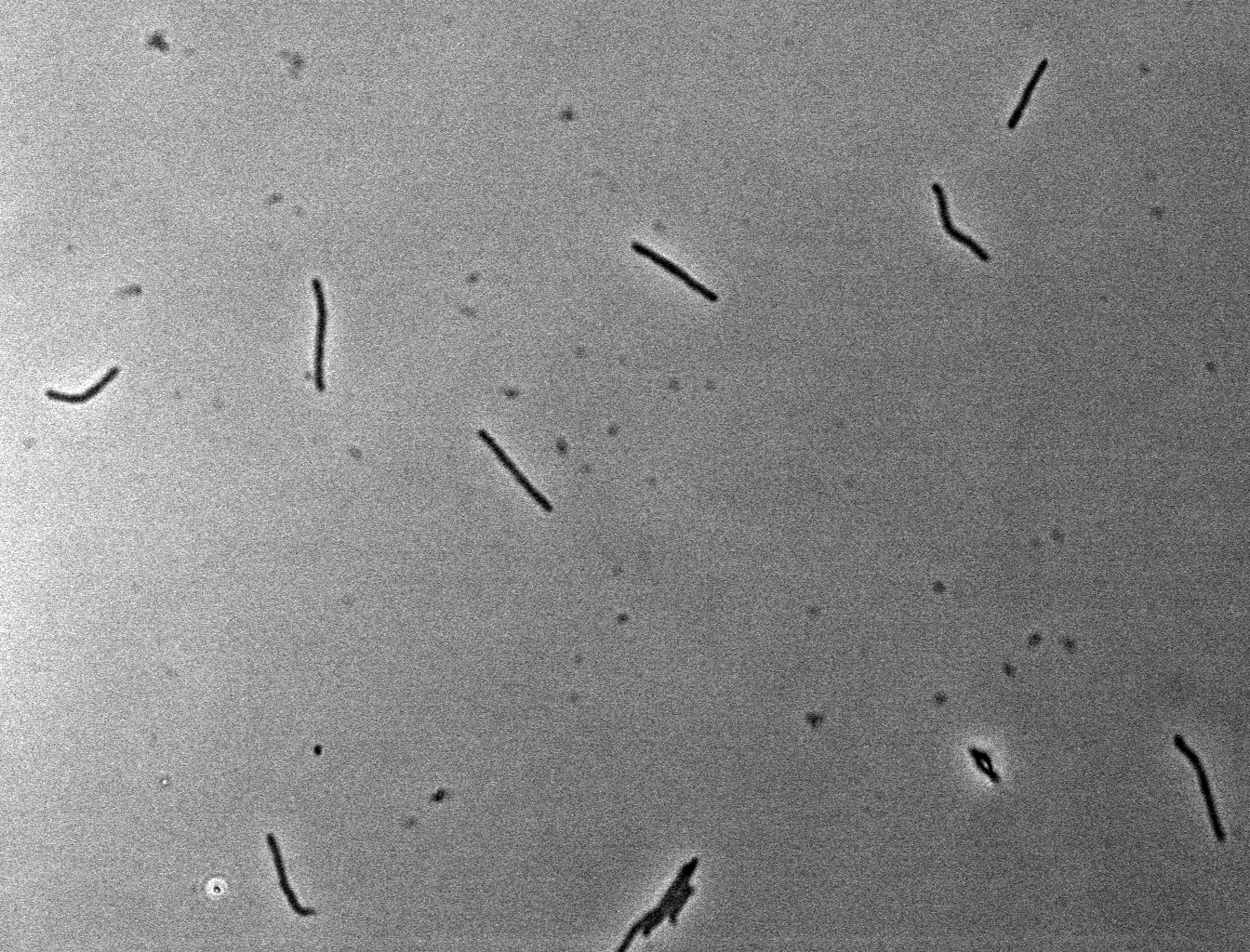

Supplement: Figure 2—source data 1. [file elife-37243-fig2-data1.zip › Figure 2 source data/Figure 2 source data-conventional microscopy (alkDD + RADA)/1. Phase/6.tif]

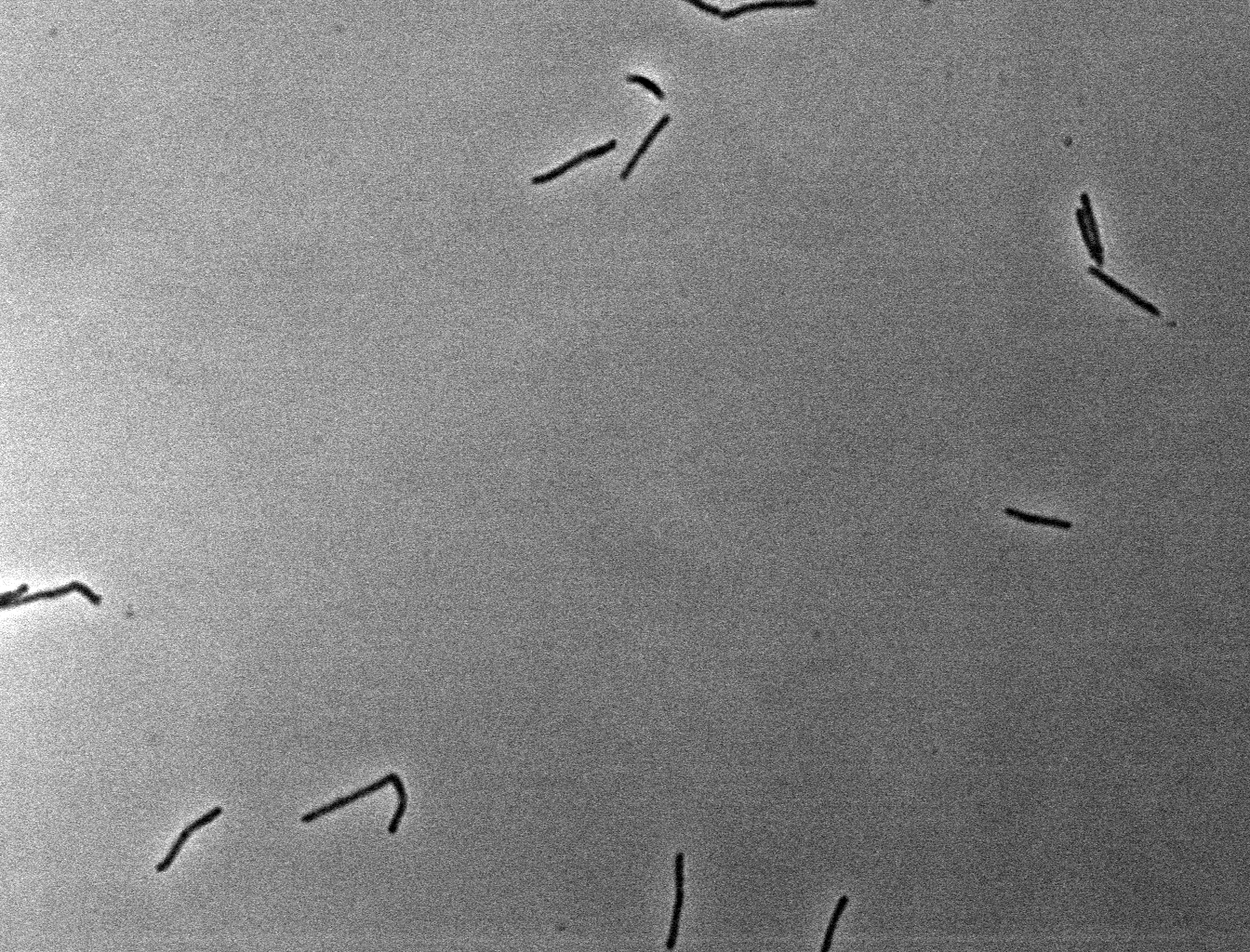

Supplement: Figure 2—source data 1. [file elife-37243-fig2-data1.zip › Figure 2 source data/Figure 2 source data-conventional microscopy (alkDD + RADA)/1. Phase/7.tif]

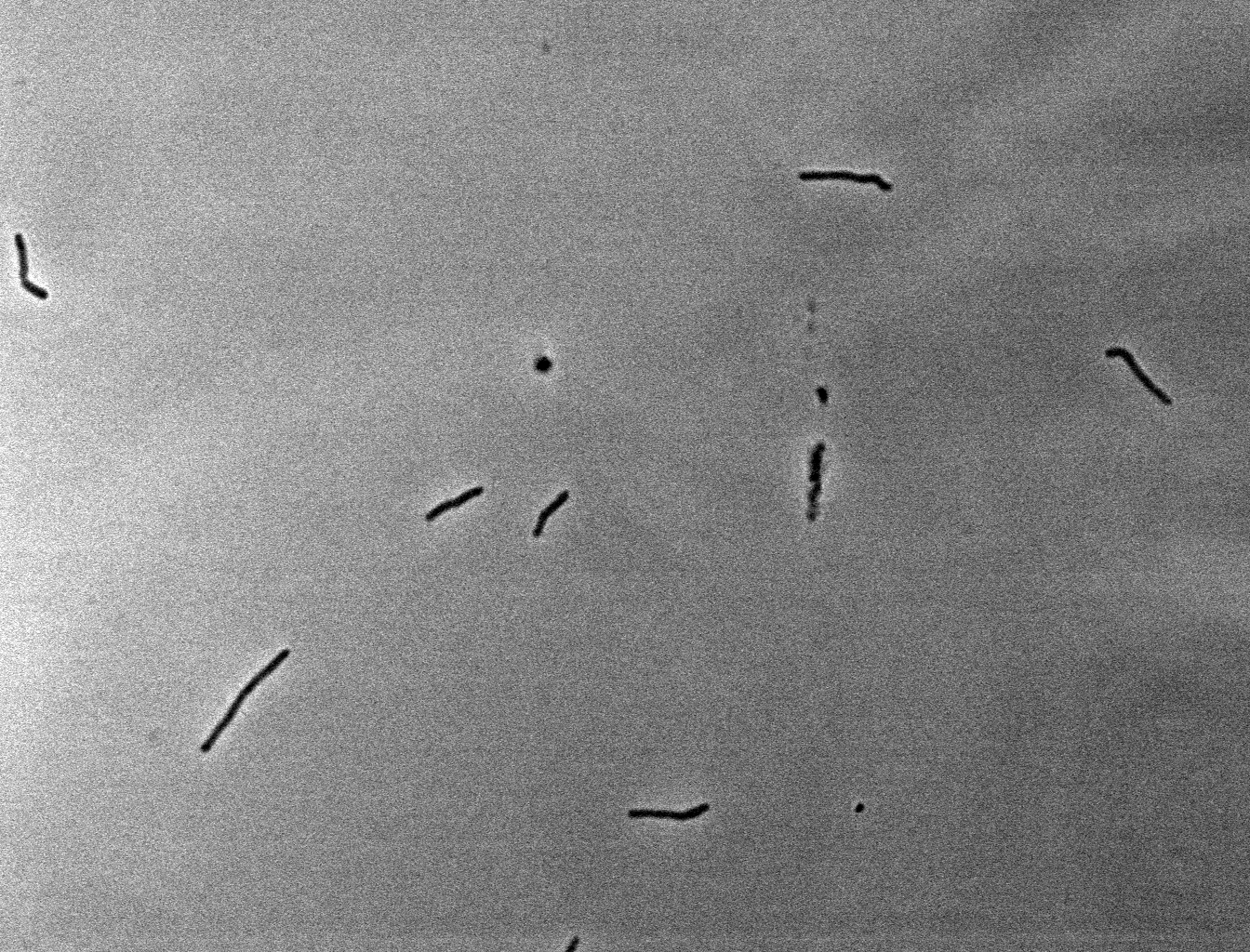

Supplement: Figure 2—source data 1. [file elife-37243-fig2-data1.zip › Figure 2 source data/Figure 2 source data-conventional microscopy (alkDD + RADA)/1. Phase/8.tif]

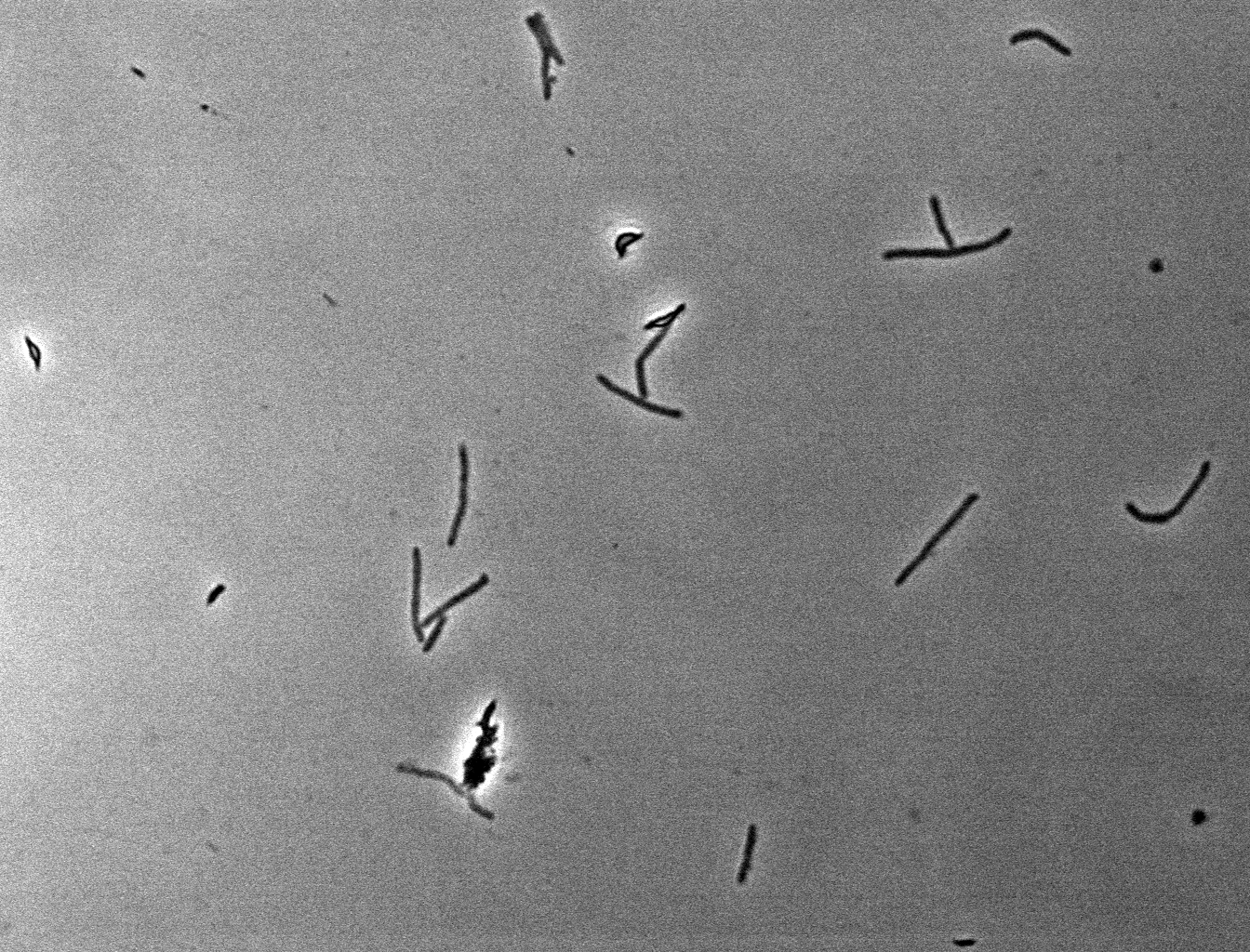

Supplement: Figure 2—source data 1. [file elife-37243-fig2-data1.zip › Figure 2 source data/Figure 2 source data-conventional microscopy (alkDD + RADA)/1. Phase/9.tif]

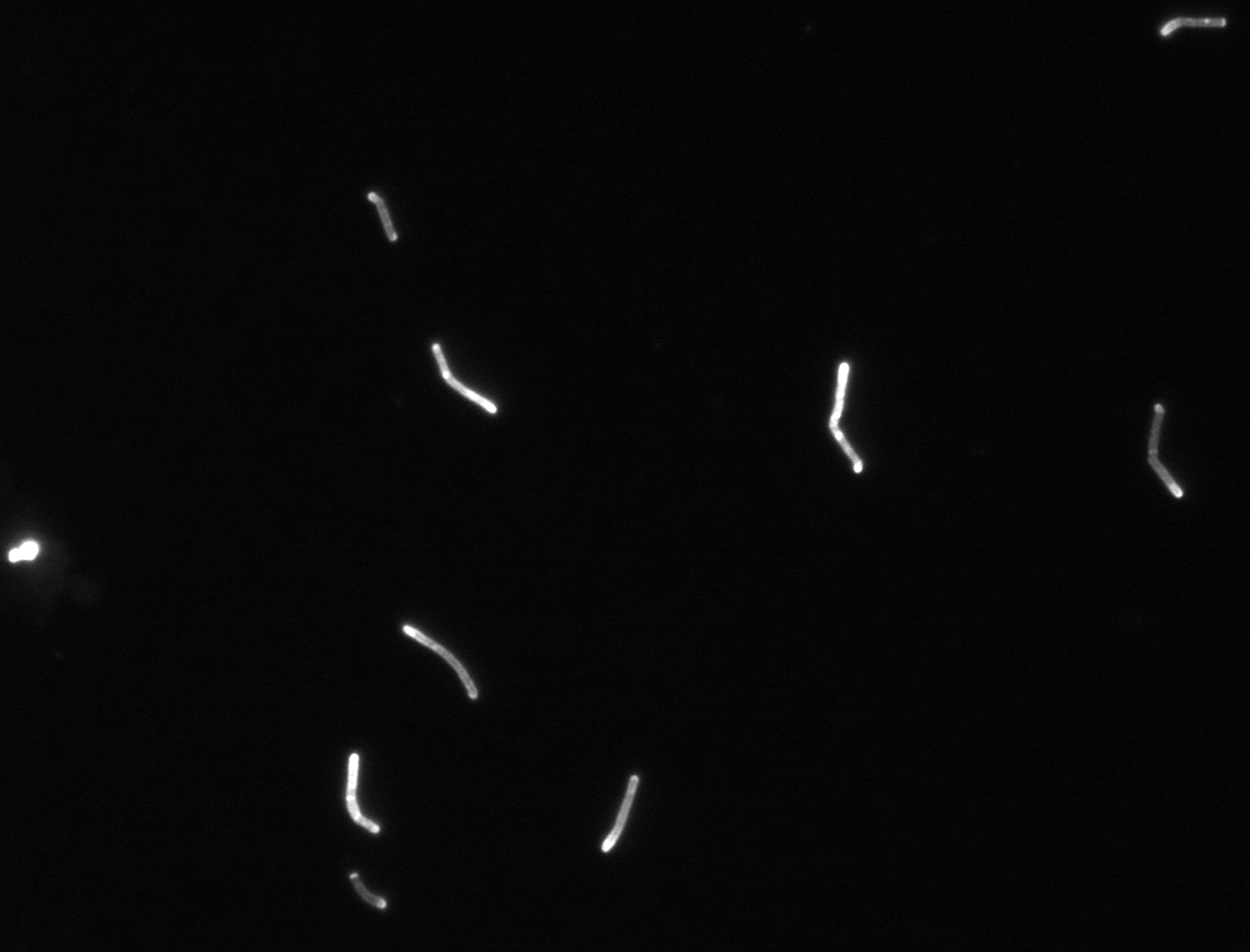

Supplement: Figure 2—source data 1. [file elife-37243-fig2-data1.zip › Figure 2 source data/Figure 2 source data-conventional microscopy (alkDD + RADA)/2. AlkDD/1.tif]

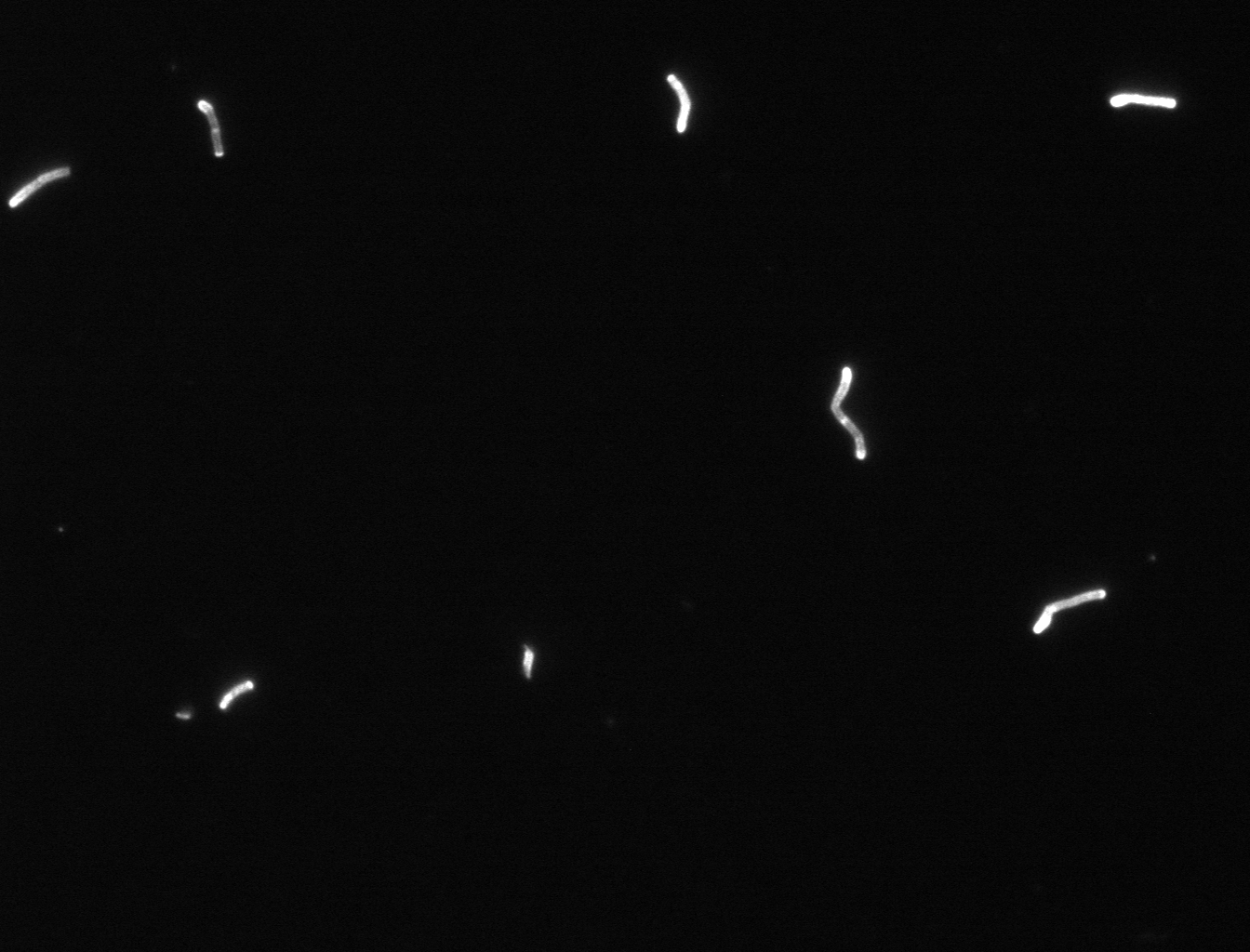

Supplement: Figure 2—source data 1. [file elife-37243-fig2-data1.zip › Figure 2 source data/Figure 2 source data-conventional microscopy (alkDD + RADA)/2. AlkDD/10.tif]

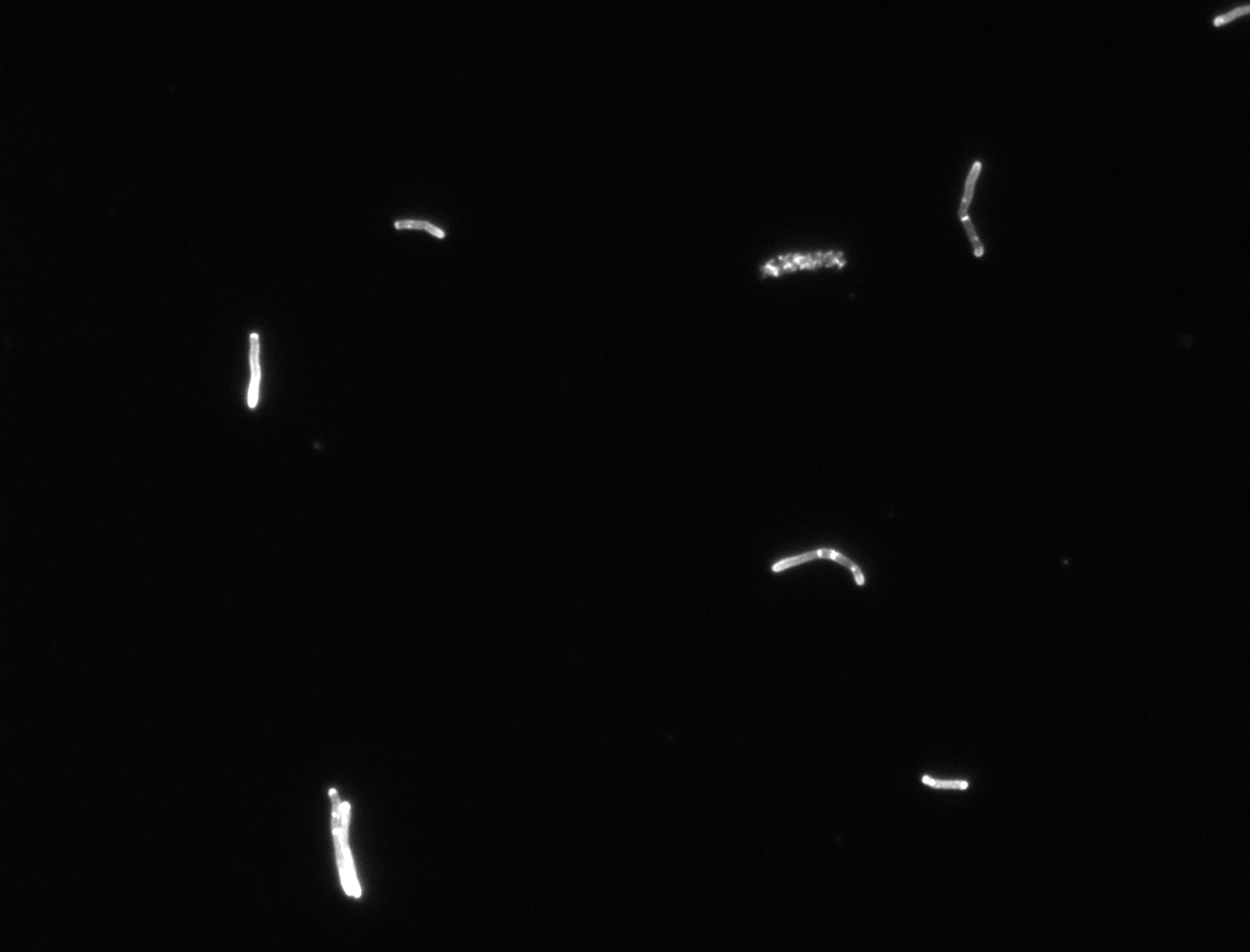

Supplement: Figure 2—source data 1. [file elife-37243-fig2-data1.zip › Figure 2 source data/Figure 2 source data-conventional microscopy (alkDD + RADA)/2. AlkDD/2.tif]

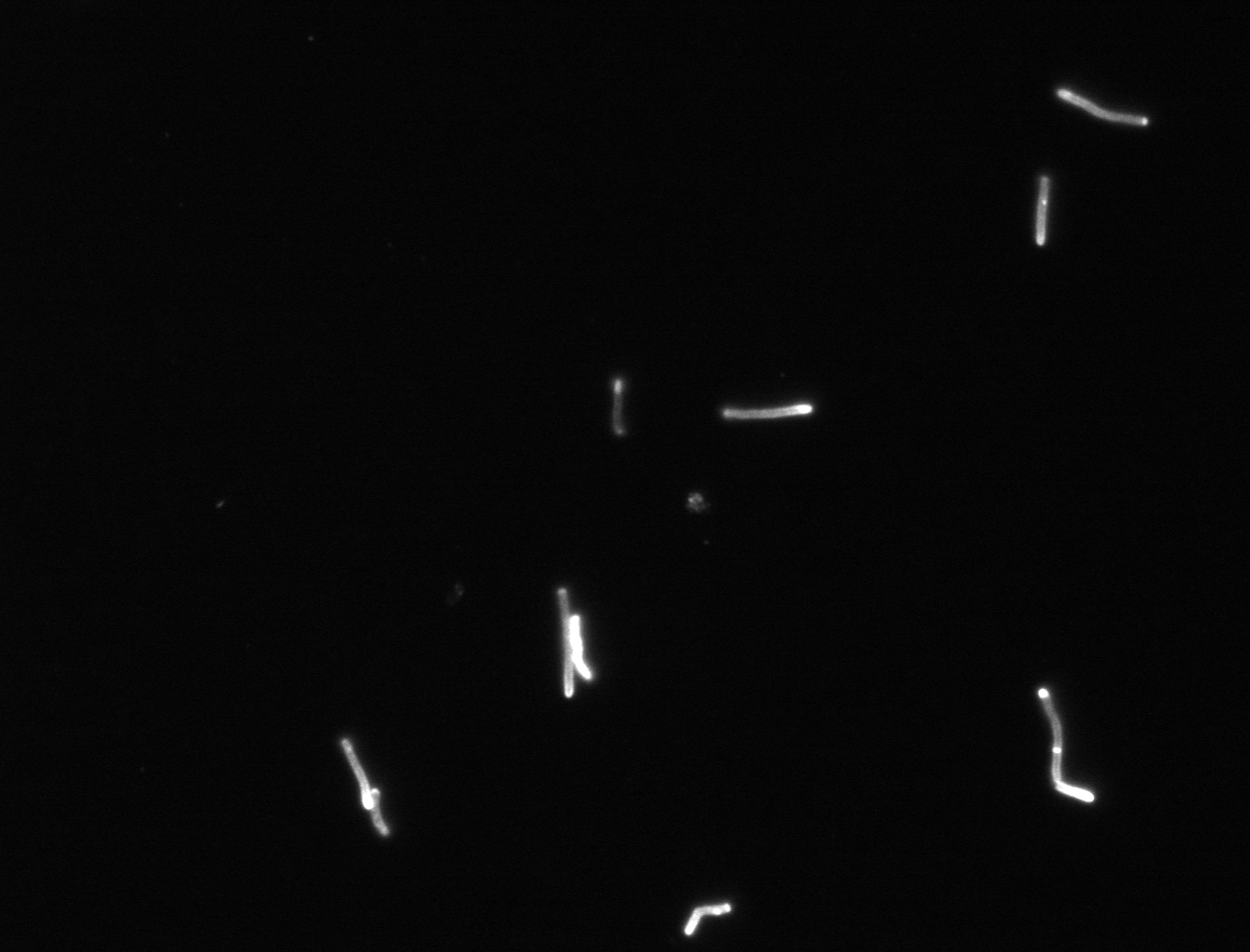

Supplement: Figure 2—source data 1. [file elife-37243-fig2-data1.zip › Figure 2 source data/Figure 2 source data-conventional microscopy (alkDD + RADA)/2. AlkDD/3.tif]

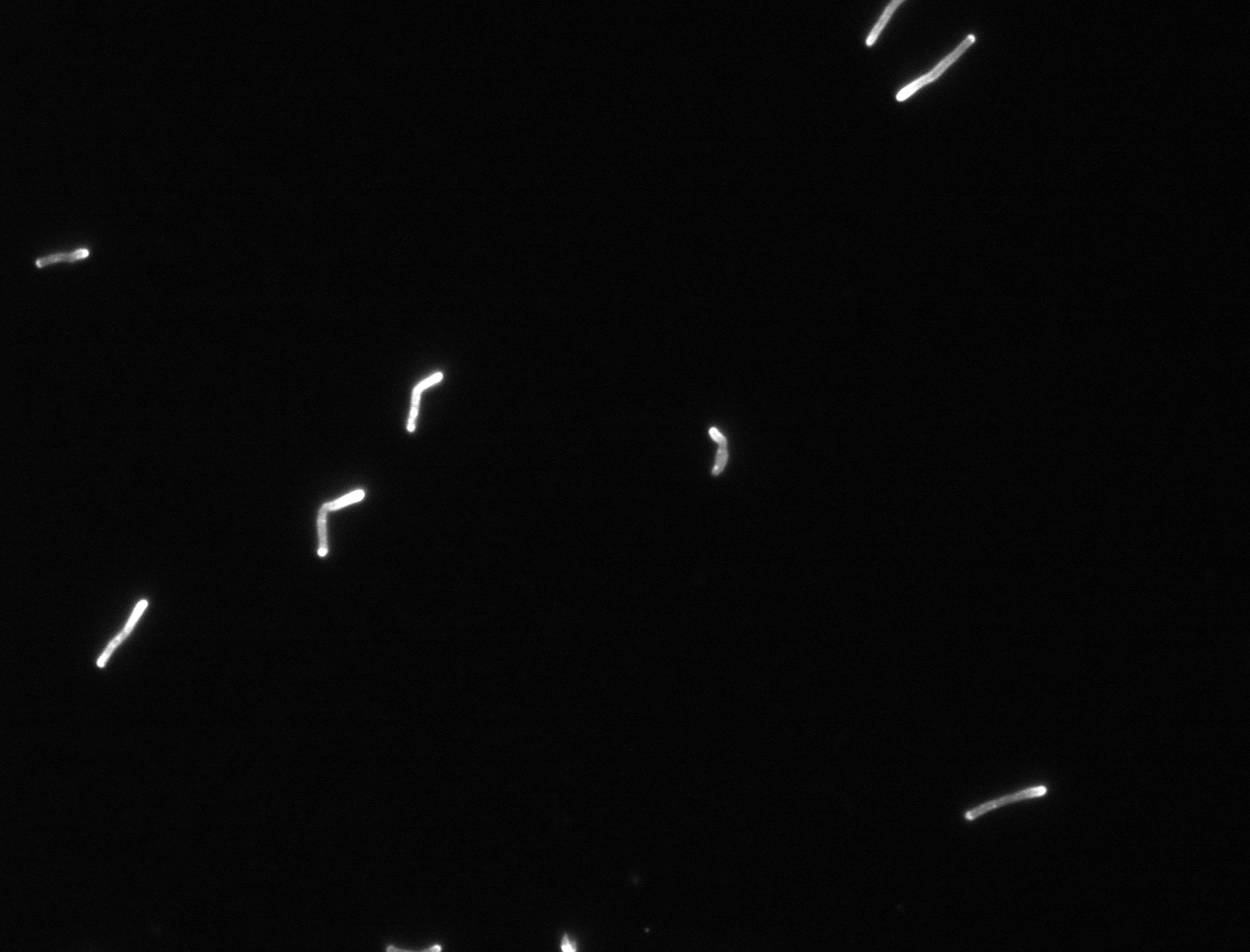

Supplement: Figure 2—source data 1. [file elife-37243-fig2-data1.zip › Figure 2 source data/Figure 2 source data-conventional microscopy (alkDD + RADA)/2. AlkDD/4.tif]

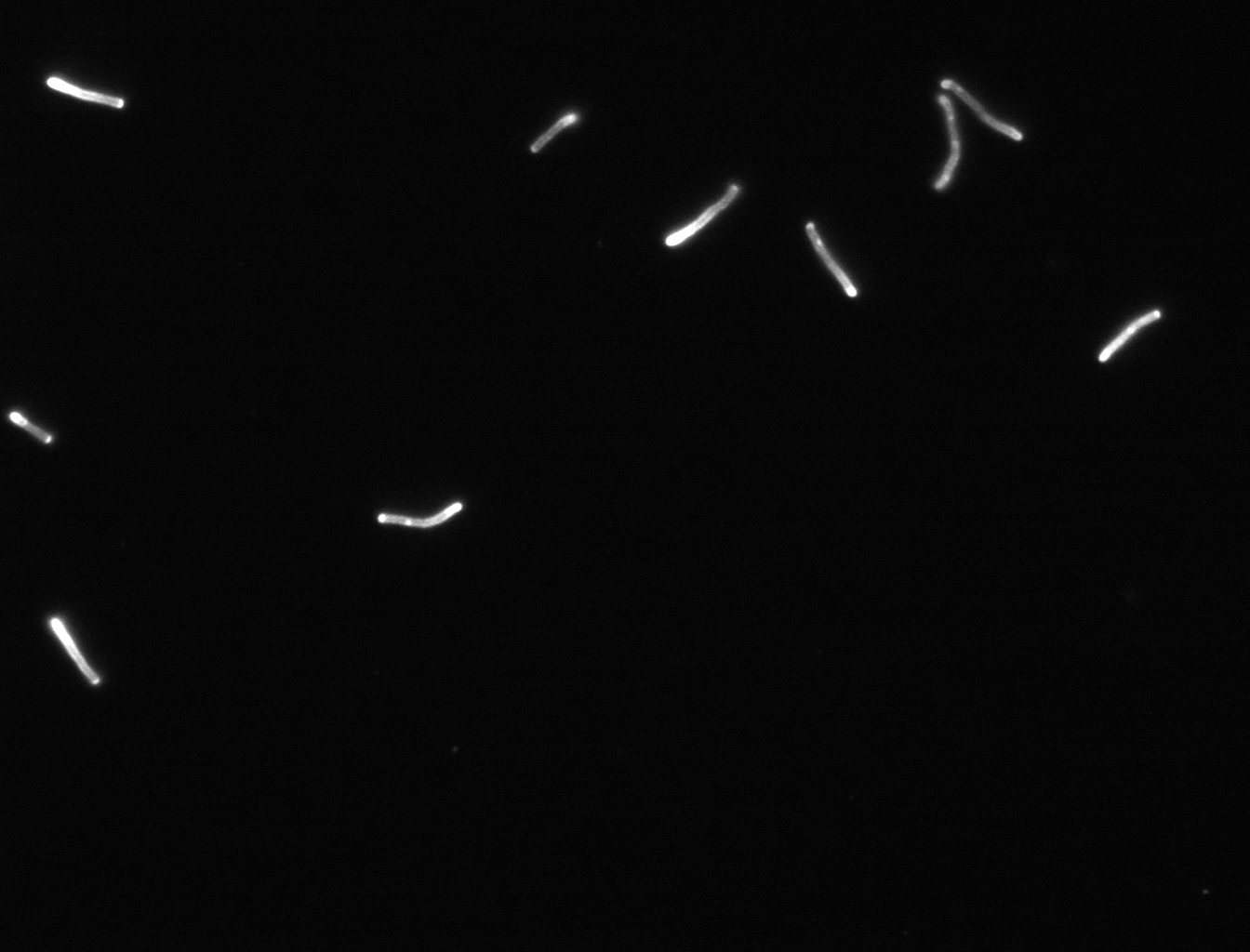

Supplement: Figure 2—source data 1. [file elife-37243-fig2-data1.zip › Figure 2 source data/Figure 2 source data-conventional microscopy (alkDD + RADA)/2. AlkDD/5.tif]

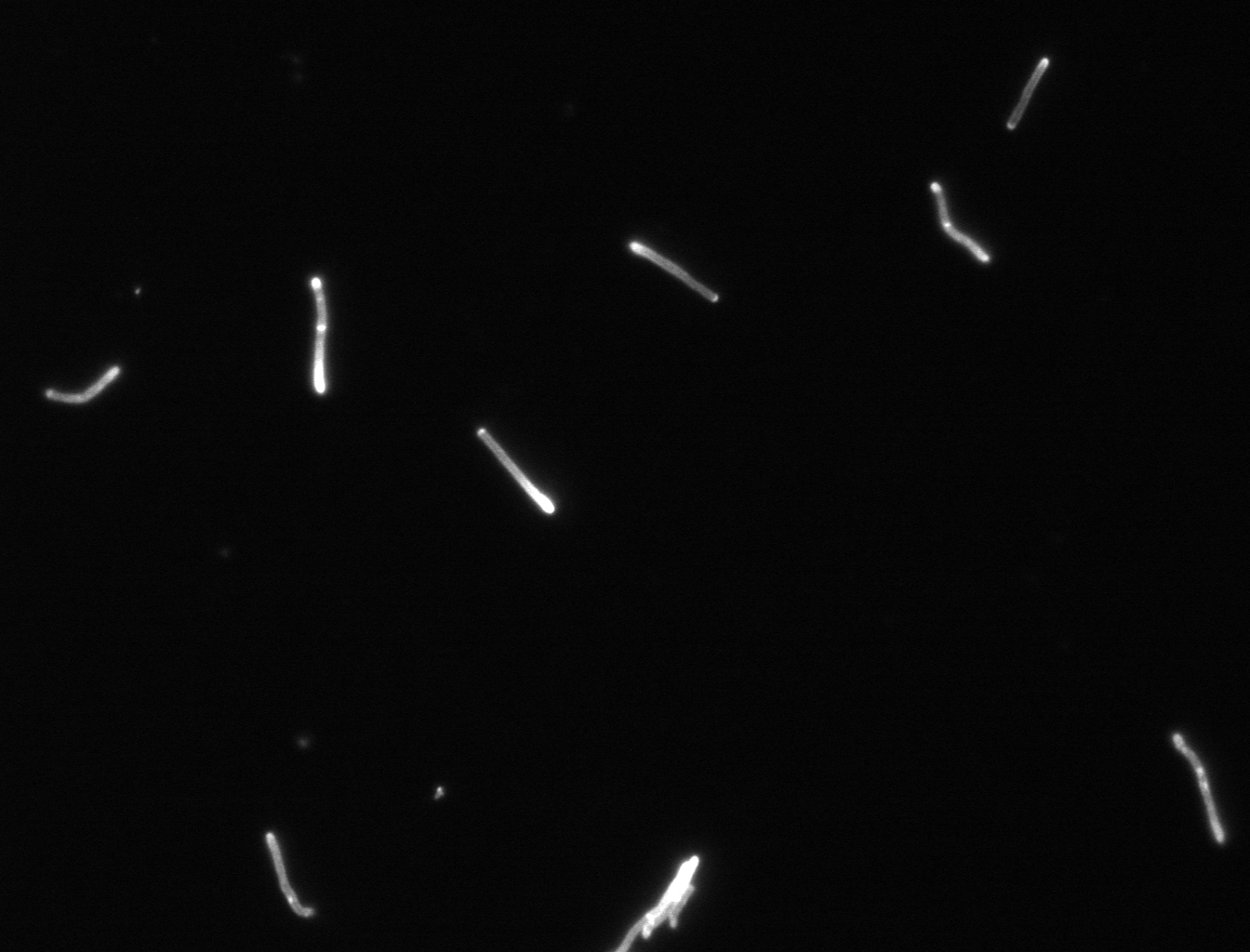

Supplement: Figure 2—source data 1. [file elife-37243-fig2-data1.zip › Figure 2 source data/Figure 2 source data-conventional microscopy (alkDD + RADA)/2. AlkDD/6.tif]

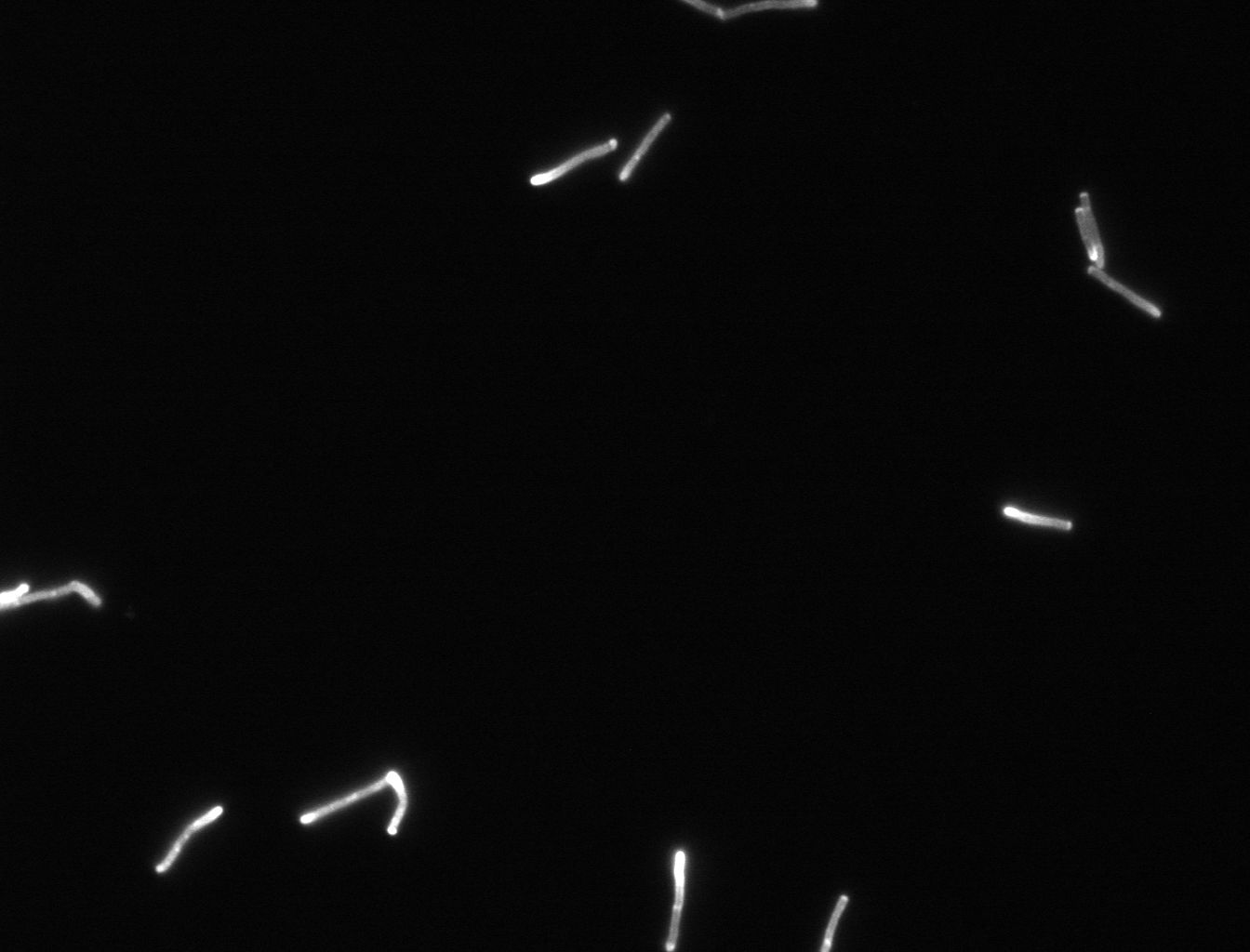

Supplement: Figure 2—source data 1. [file elife-37243-fig2-data1.zip › Figure 2 source data/Figure 2 source data-conventional microscopy (alkDD + RADA)/2. AlkDD/7.tif]

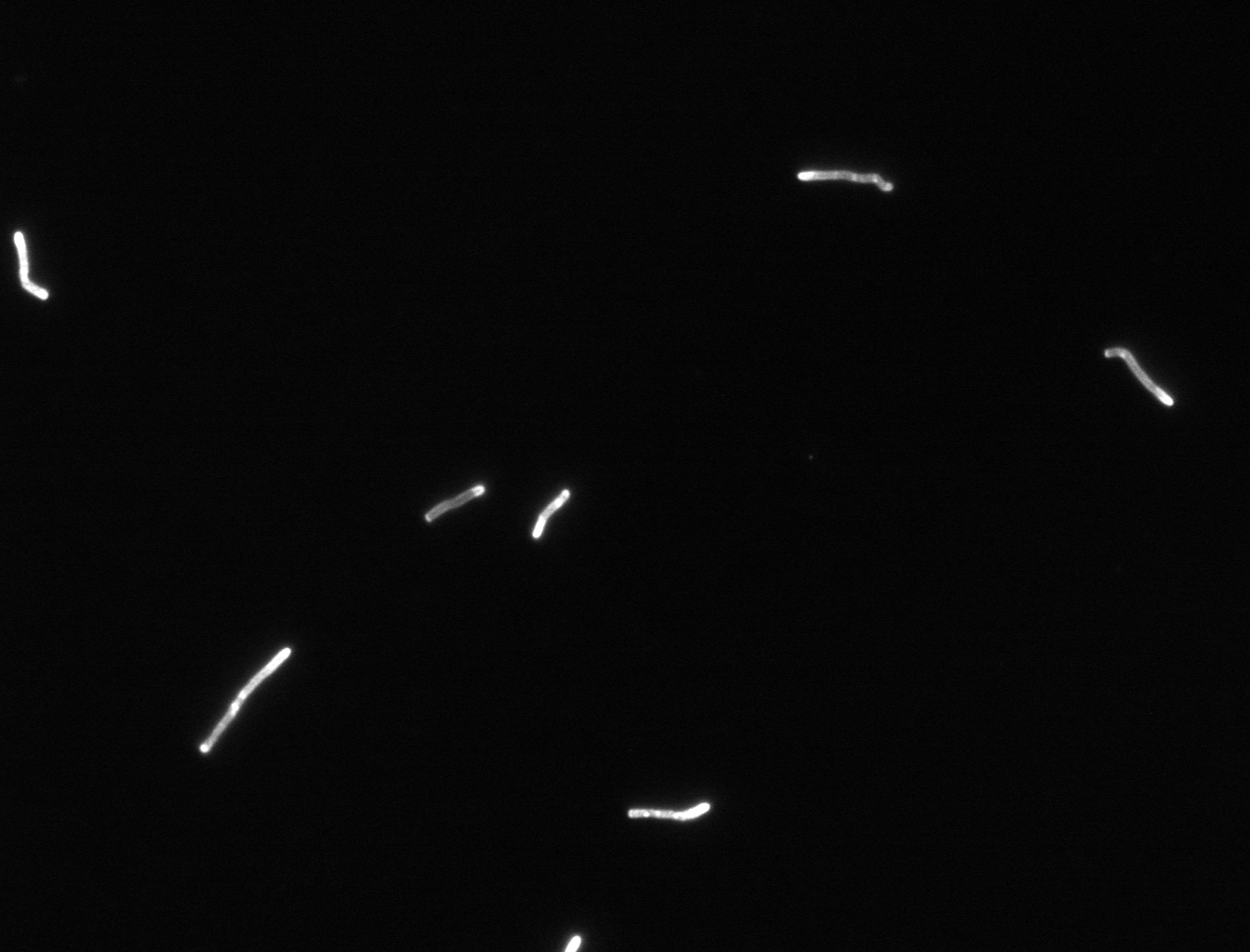

Supplement: Figure 2—source data 1. [file elife-37243-fig2-data1.zip › Figure 2 source data/Figure 2 source data-conventional microscopy (alkDD + RADA)/2. AlkDD/8.tif]

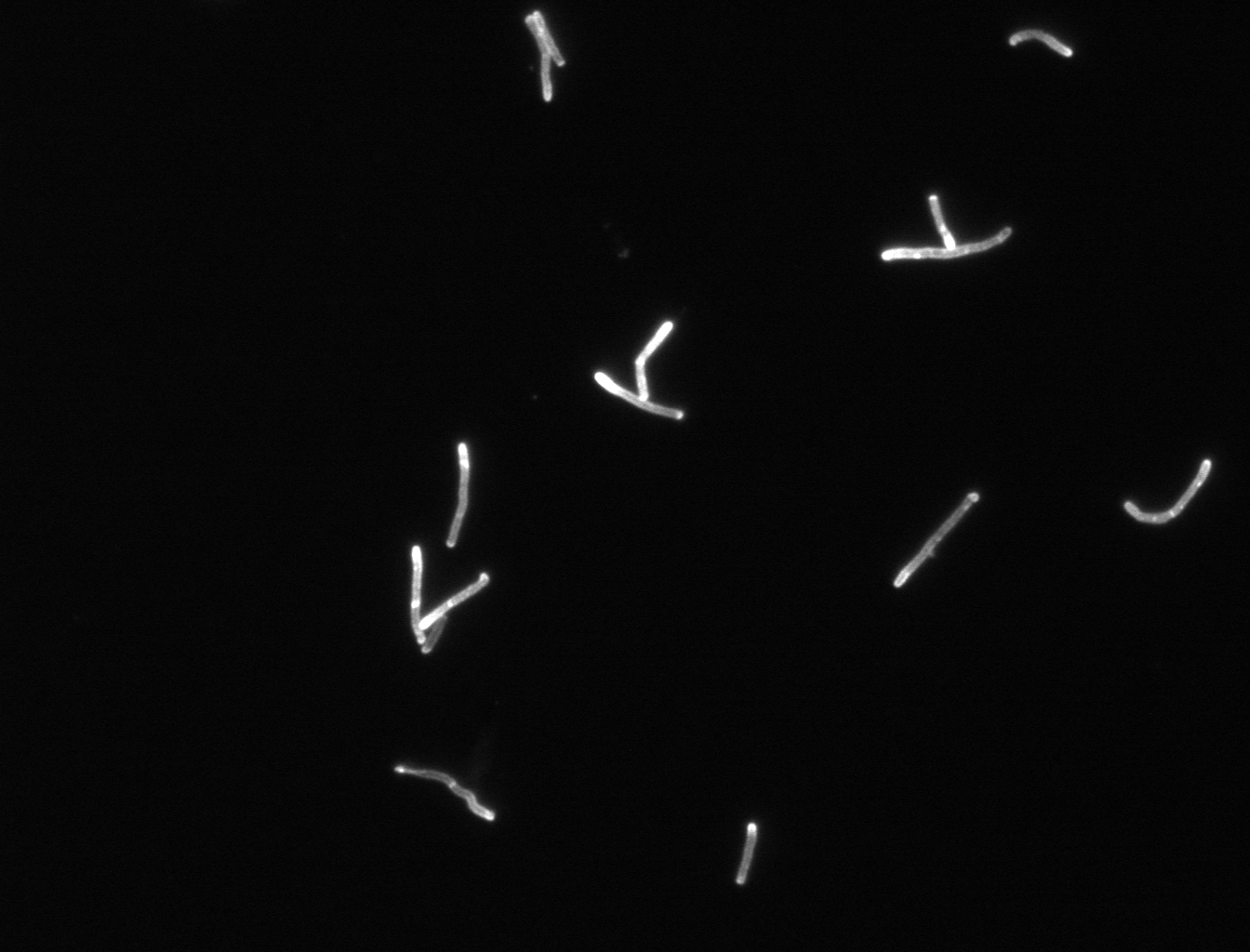

Supplement: Figure 2—source data 1. [file elife-37243-fig2-data1.zip › Figure 2 source data/Figure 2 source data-conventional microscopy (alkDD + RADA)/2. AlkDD/9.tif]

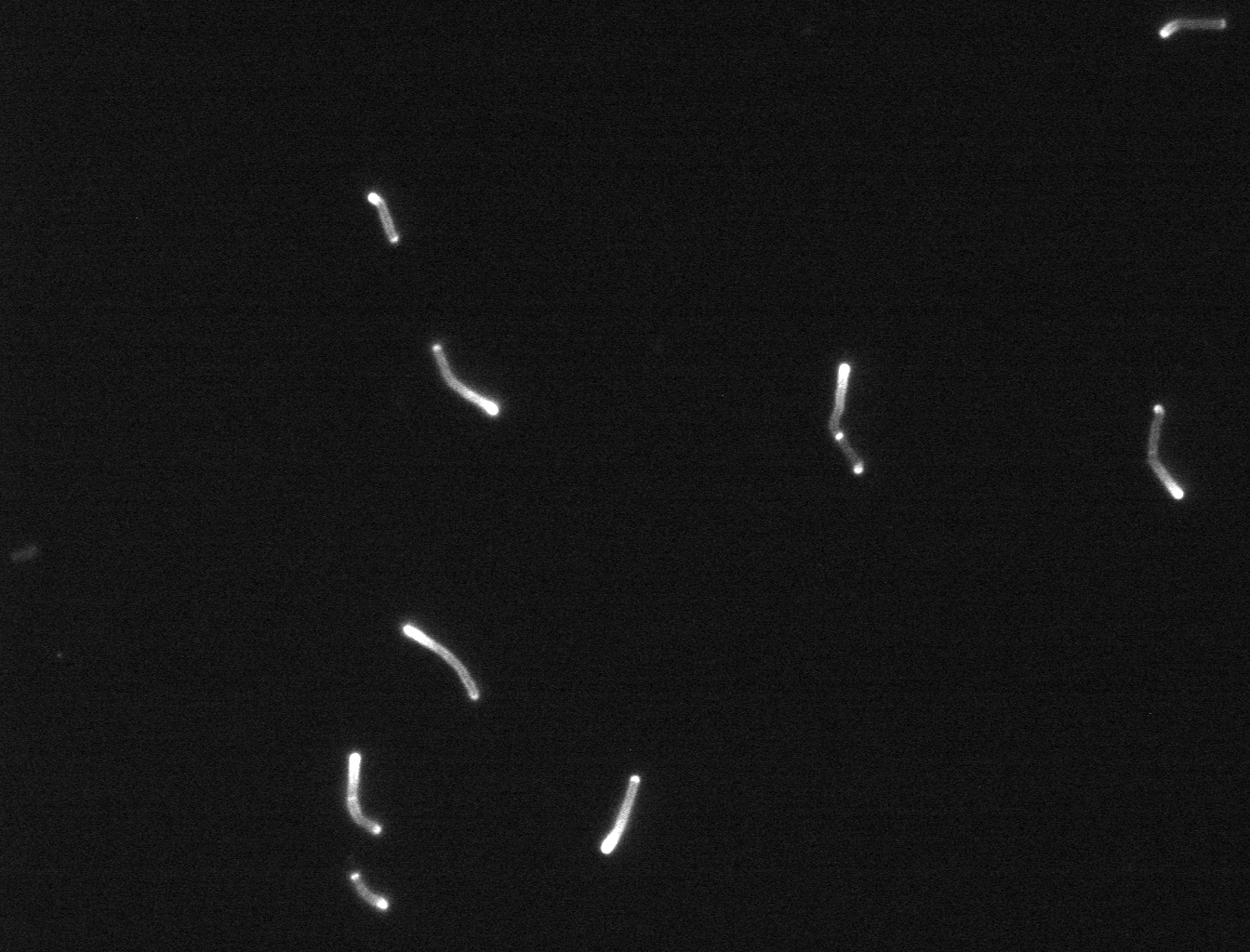

Supplement: Figure 2—source data 1. [file elife-37243-fig2-data1.zip › Figure 2 source data/Figure 2 source data-conventional microscopy (alkDD + RADA)/3. RADA/1.tif]

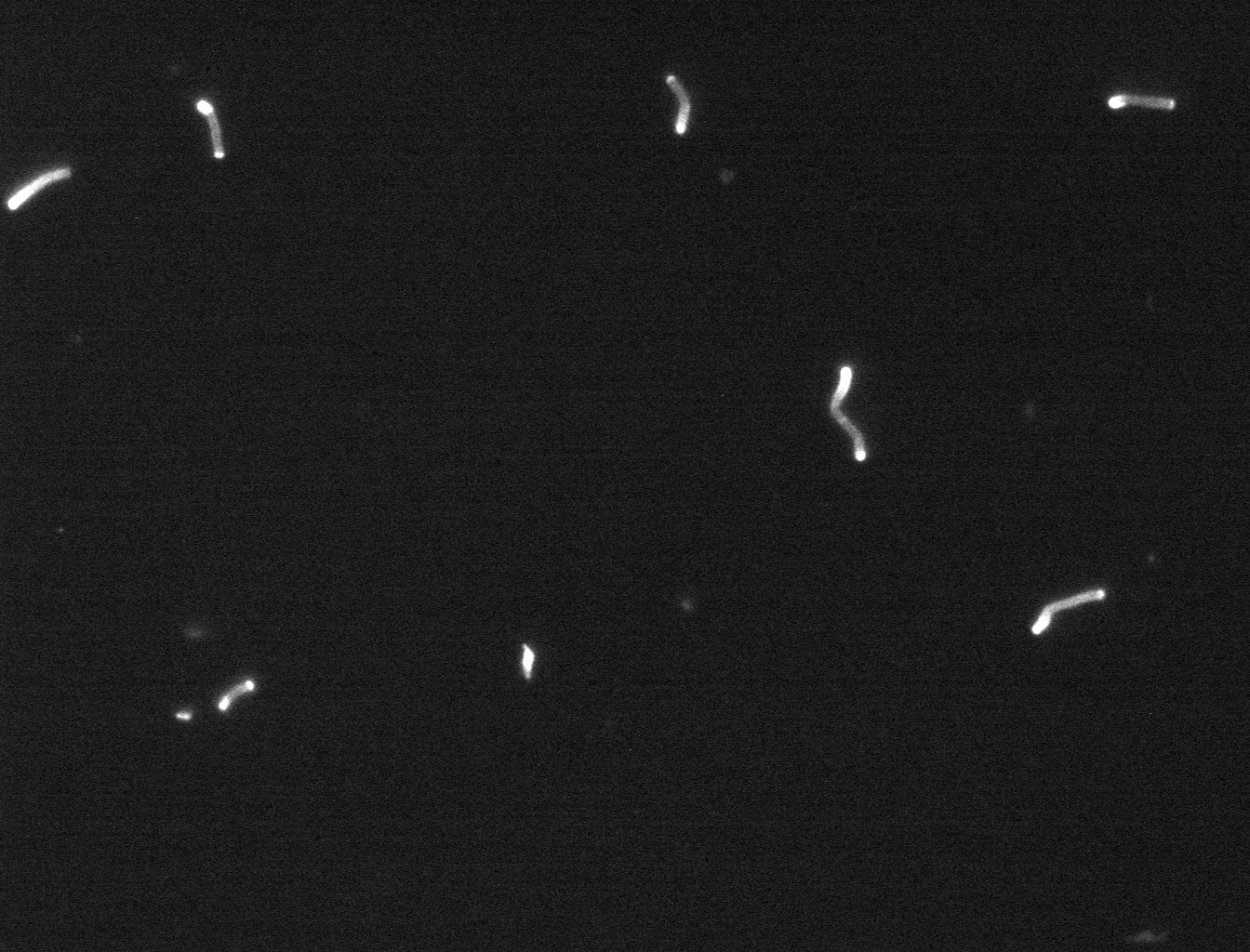

Supplement: Figure 2—source data 1. [file elife-37243-fig2-data1.zip › Figure 2 source data/Figure 2 source data-conventional microscopy (alkDD + RADA)/3. RADA/10.tif]

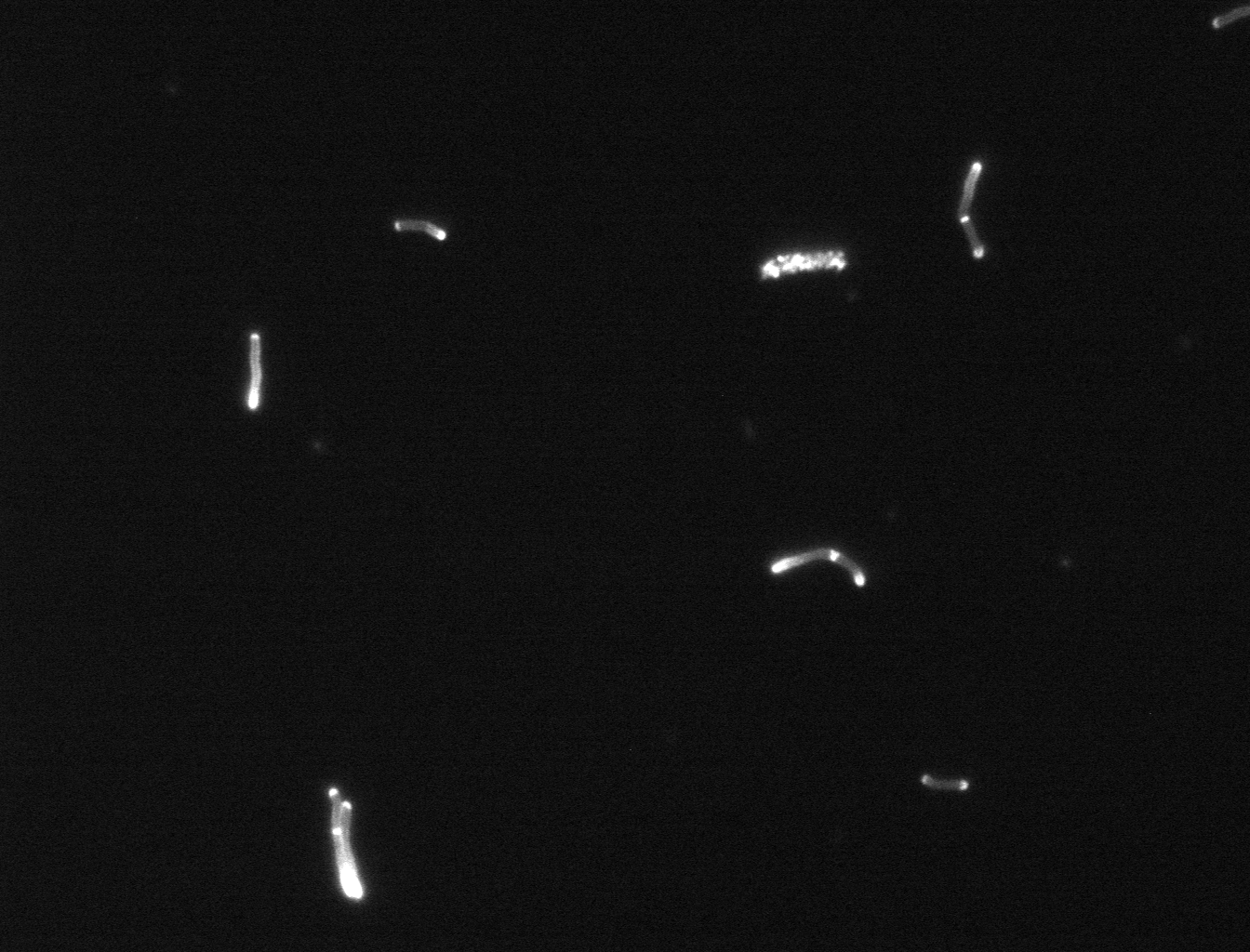

Supplement: Figure 2—source data 1. [file elife-37243-fig2-data1.zip › Figure 2 source data/Figure 2 source data-conventional microscopy (alkDD + RADA)/3. RADA/2.tif]

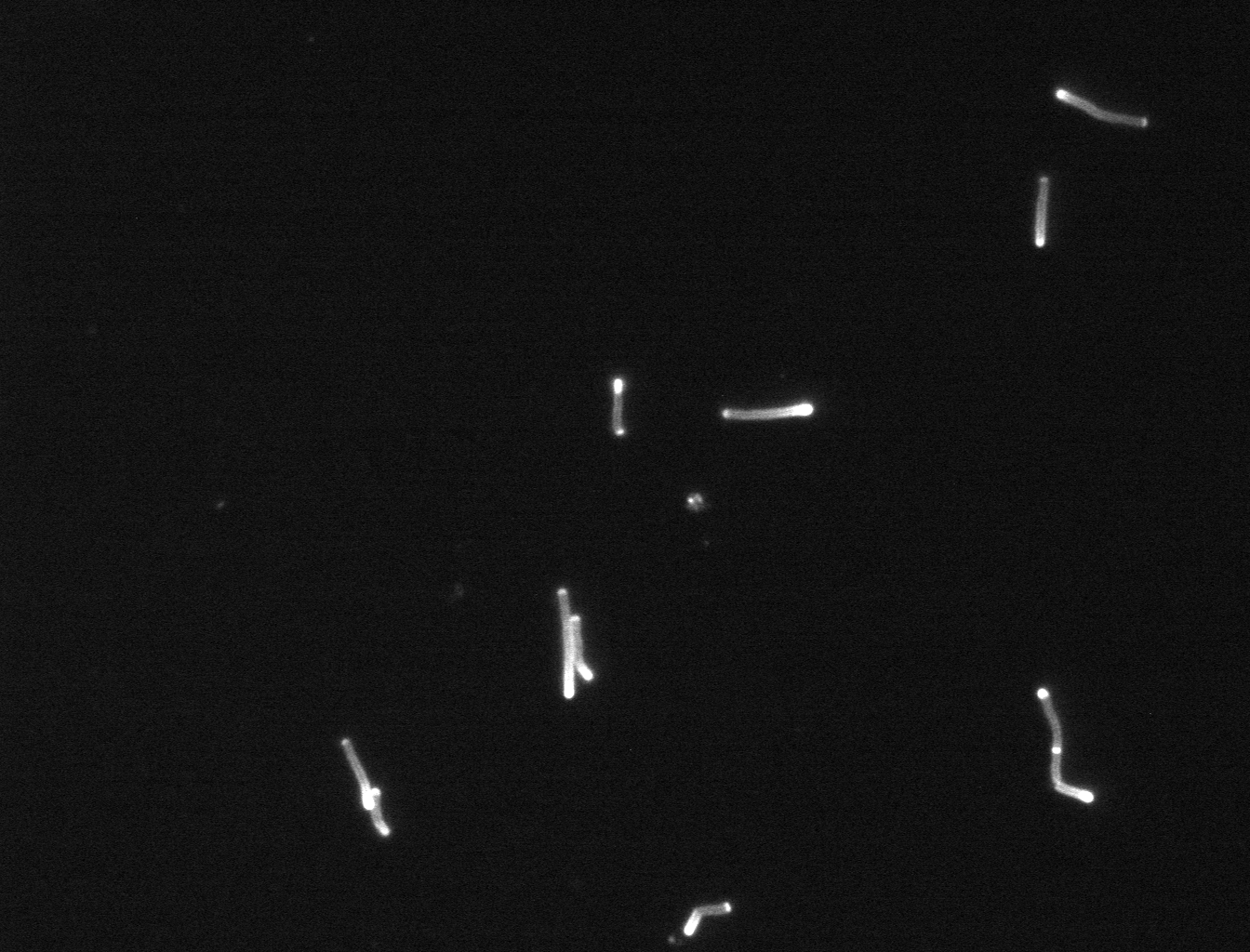

Supplement: Figure 2—source data 1. [file elife-37243-fig2-data1.zip › Figure 2 source data/Figure 2 source data-conventional microscopy (alkDD + RADA)/3. RADA/3.tif]

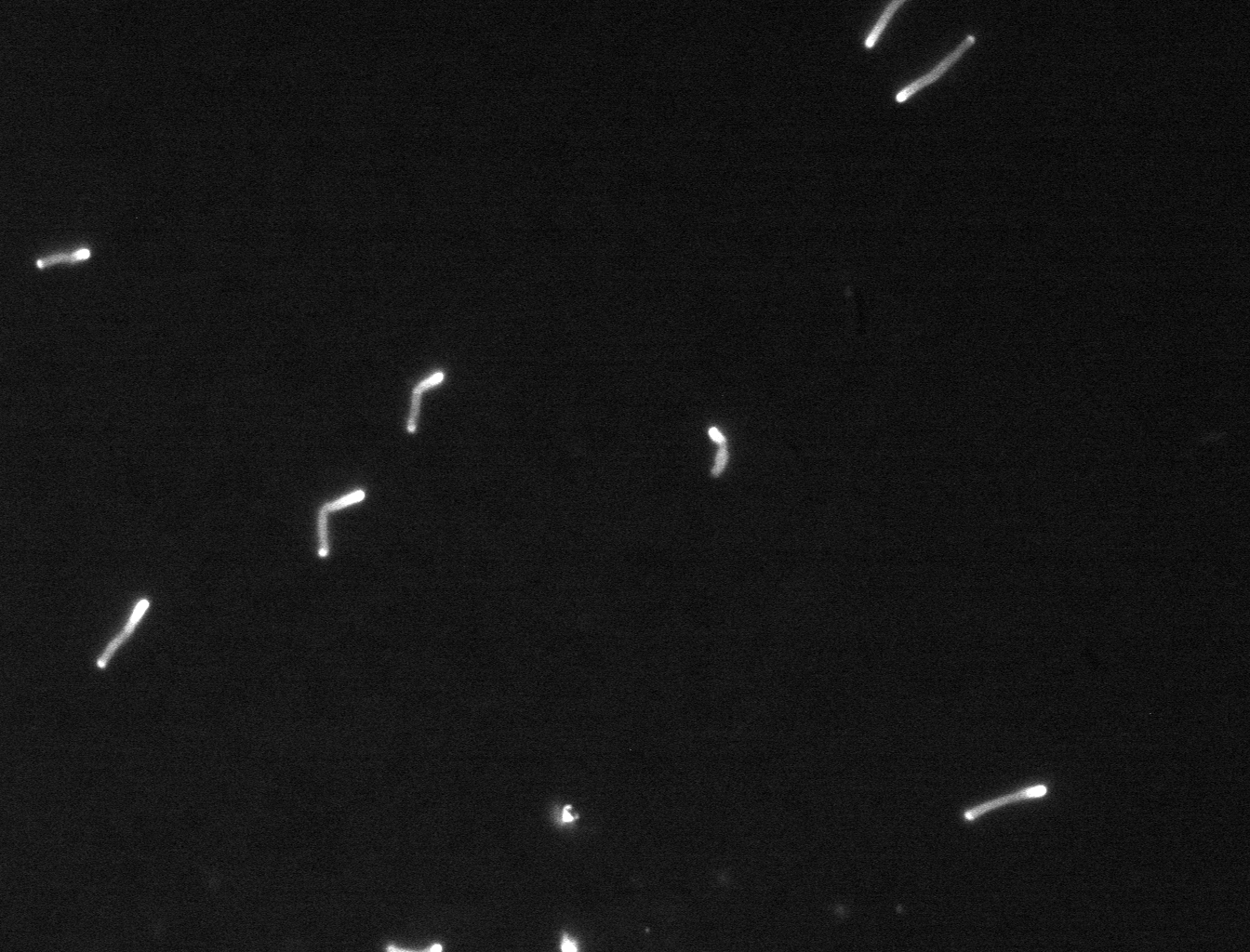

Supplement: Figure 2—source data 1. [file elife-37243-fig2-data1.zip › Figure 2 source data/Figure 2 source data-conventional microscopy (alkDD + RADA)/3. RADA/4.tif]

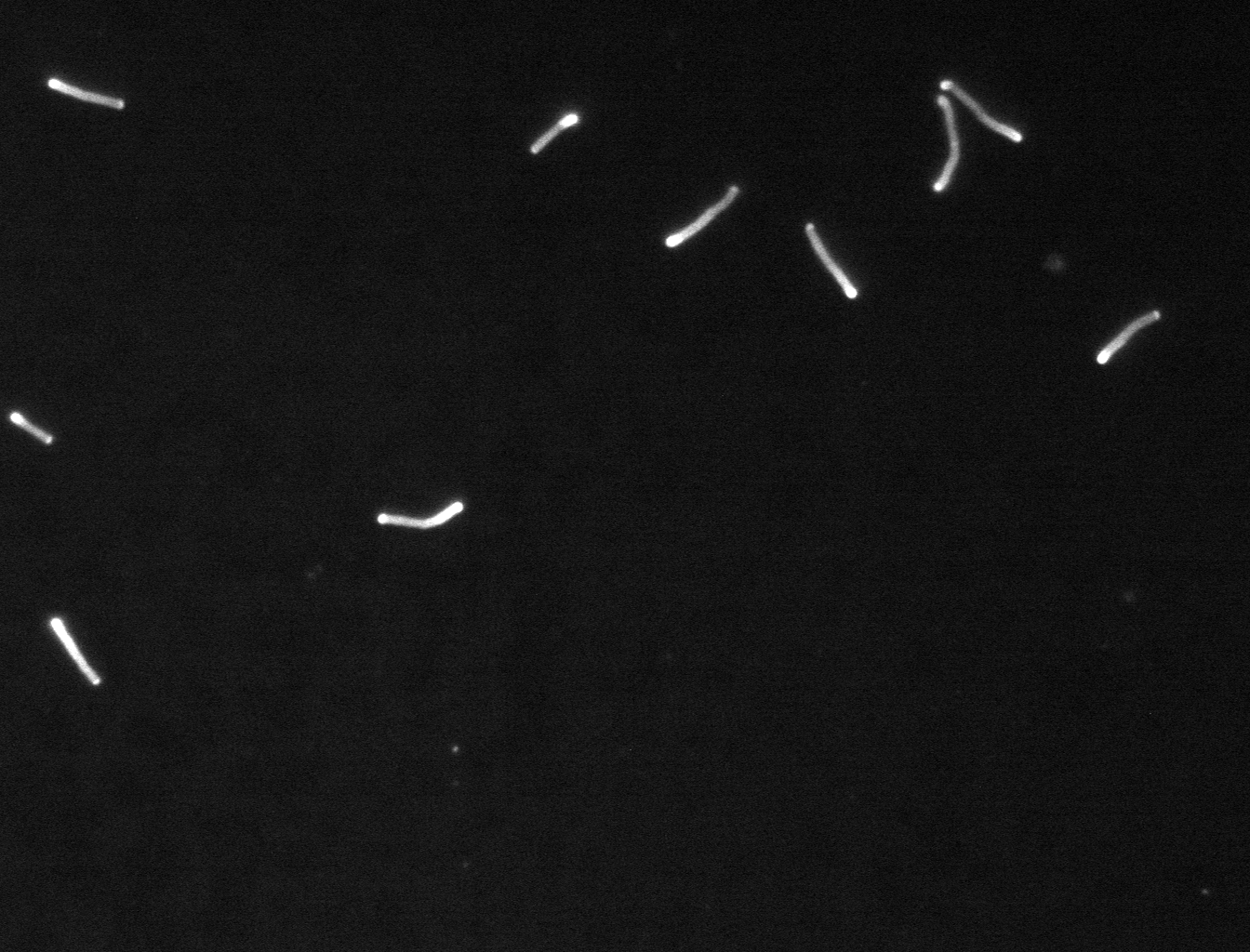

Supplement: Figure 2—source data 1. [file elife-37243-fig2-data1.zip › Figure 2 source data/Figure 2 source data-conventional microscopy (alkDD + RADA)/3. RADA/5.tif]

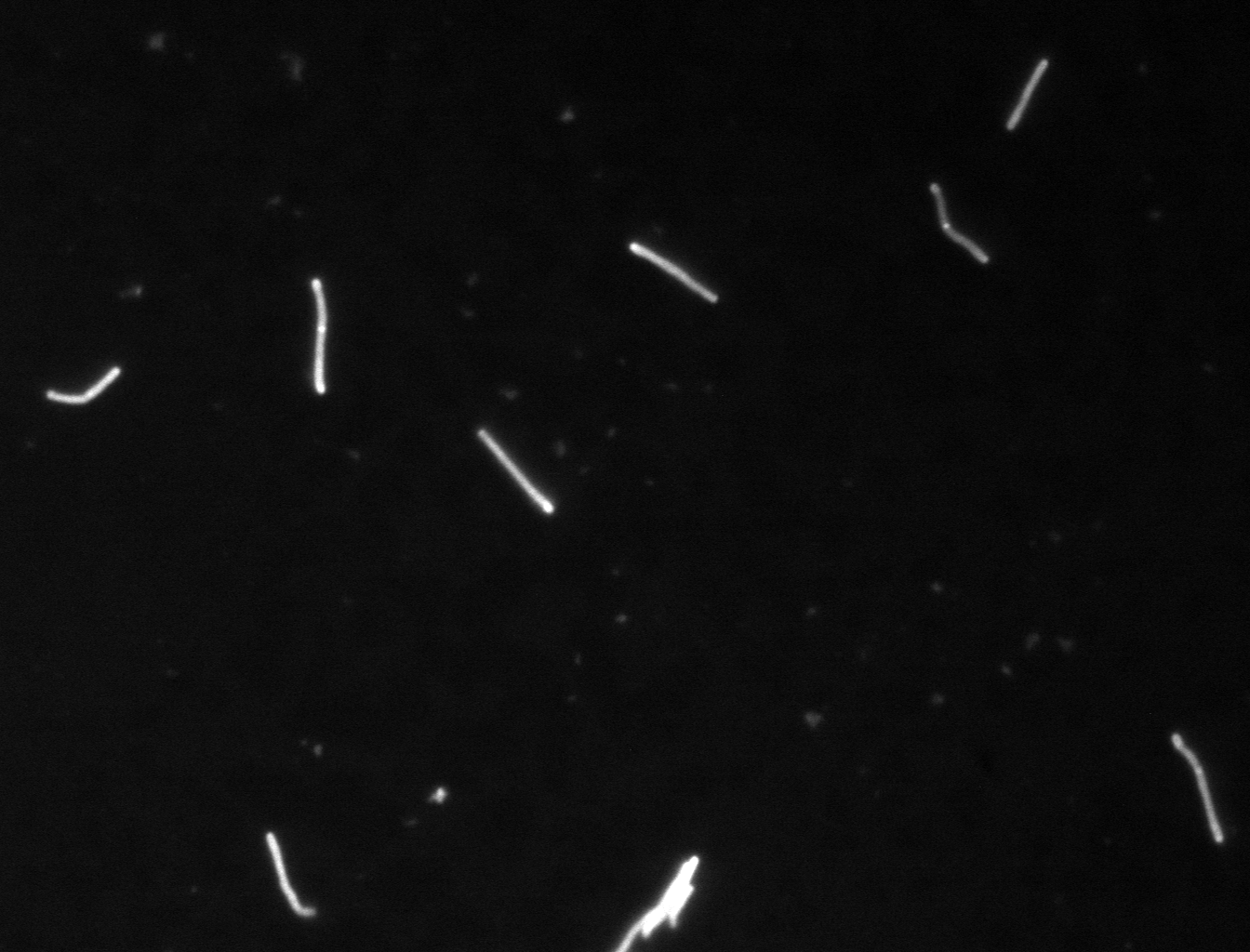

Supplement: Figure 2—source data 1. [file elife-37243-fig2-data1.zip › Figure 2 source data/Figure 2 source data-conventional microscopy (alkDD + RADA)/3. RADA/6.tif]

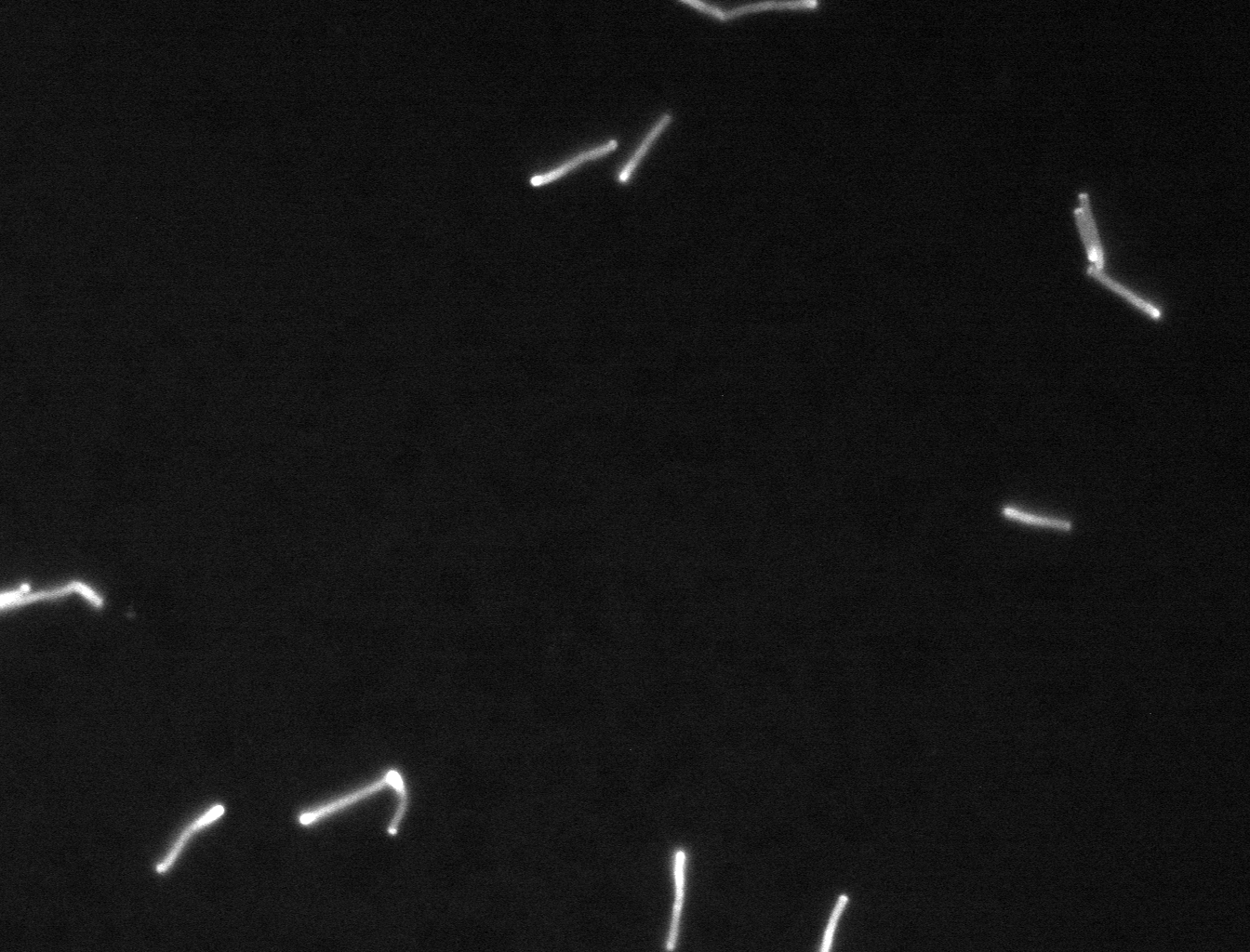

Supplement: Figure 2—source data 1. [file elife-37243-fig2-data1.zip › Figure 2 source data/Figure 2 source data-conventional microscopy (alkDD + RADA)/3. RADA/7.tif]

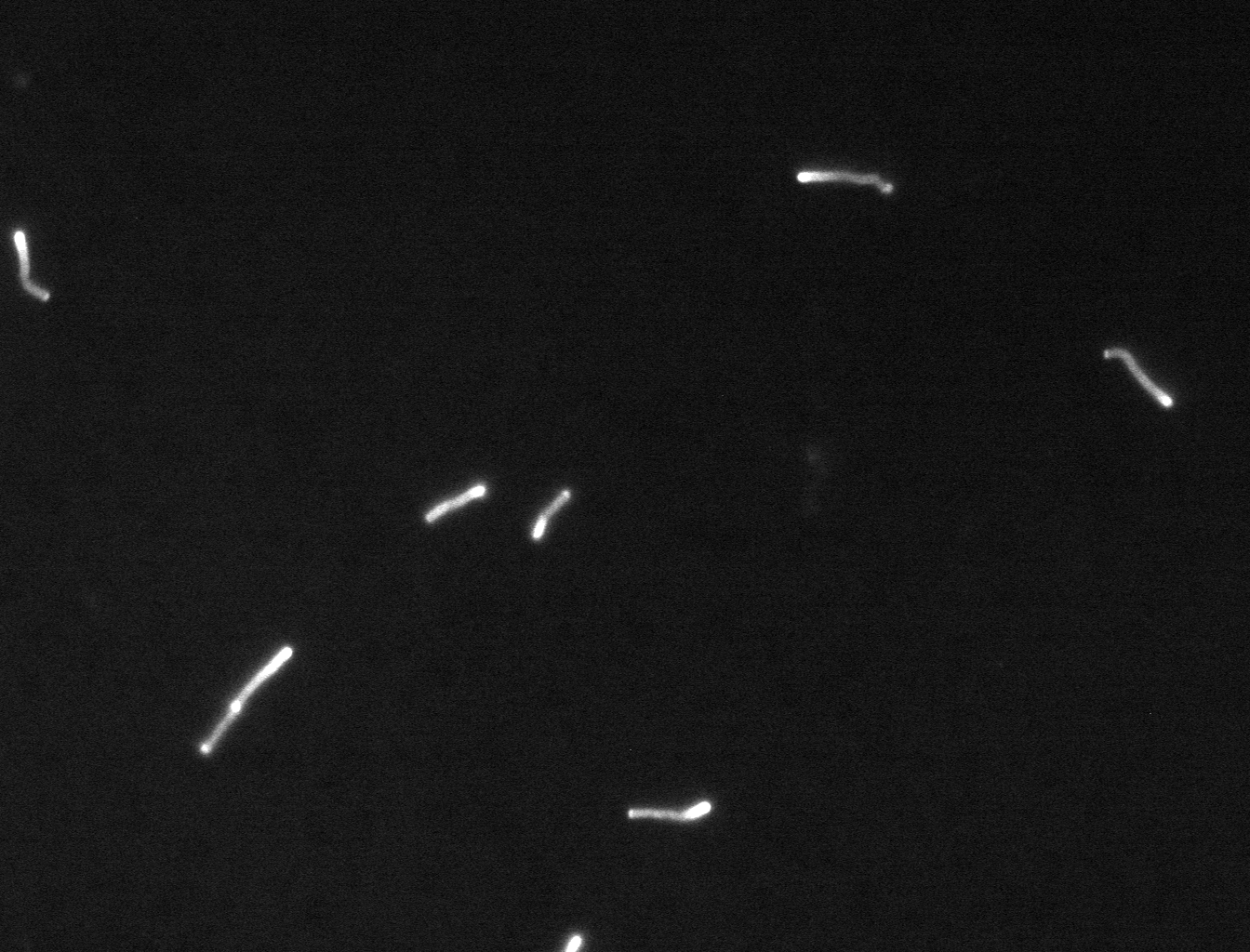

Supplement: Figure 2—source data 1. [file elife-37243-fig2-data1.zip › Figure 2 source data/Figure 2 source data-conventional microscopy (alkDD + RADA)/3. RADA/8.tif]

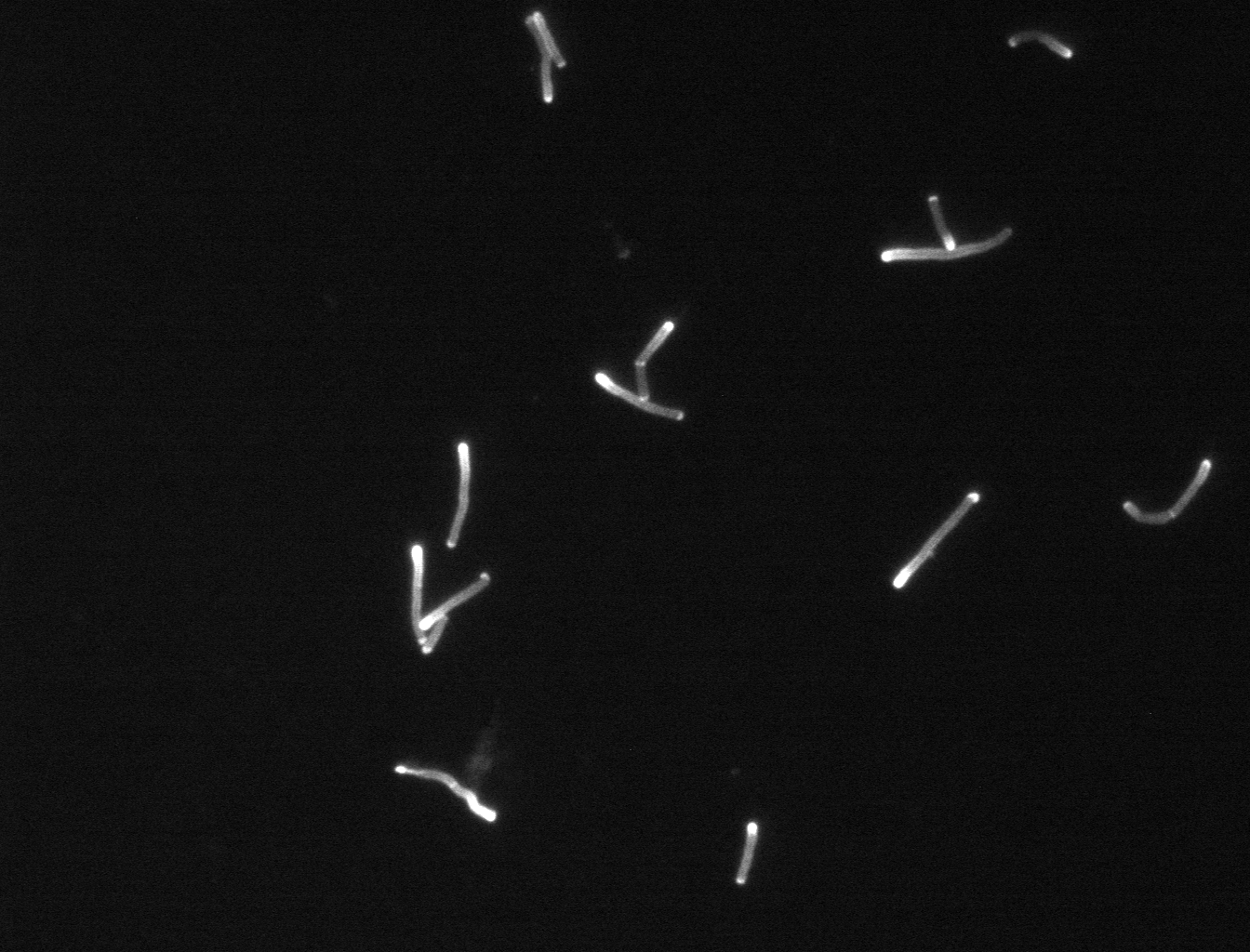

Supplement: Figure 2—source data 1. [file elife-37243-fig2-data1.zip › Figure 2 source data/Figure 2 source data-conventional microscopy (alkDD + RADA)/3. RADA/9.tif]

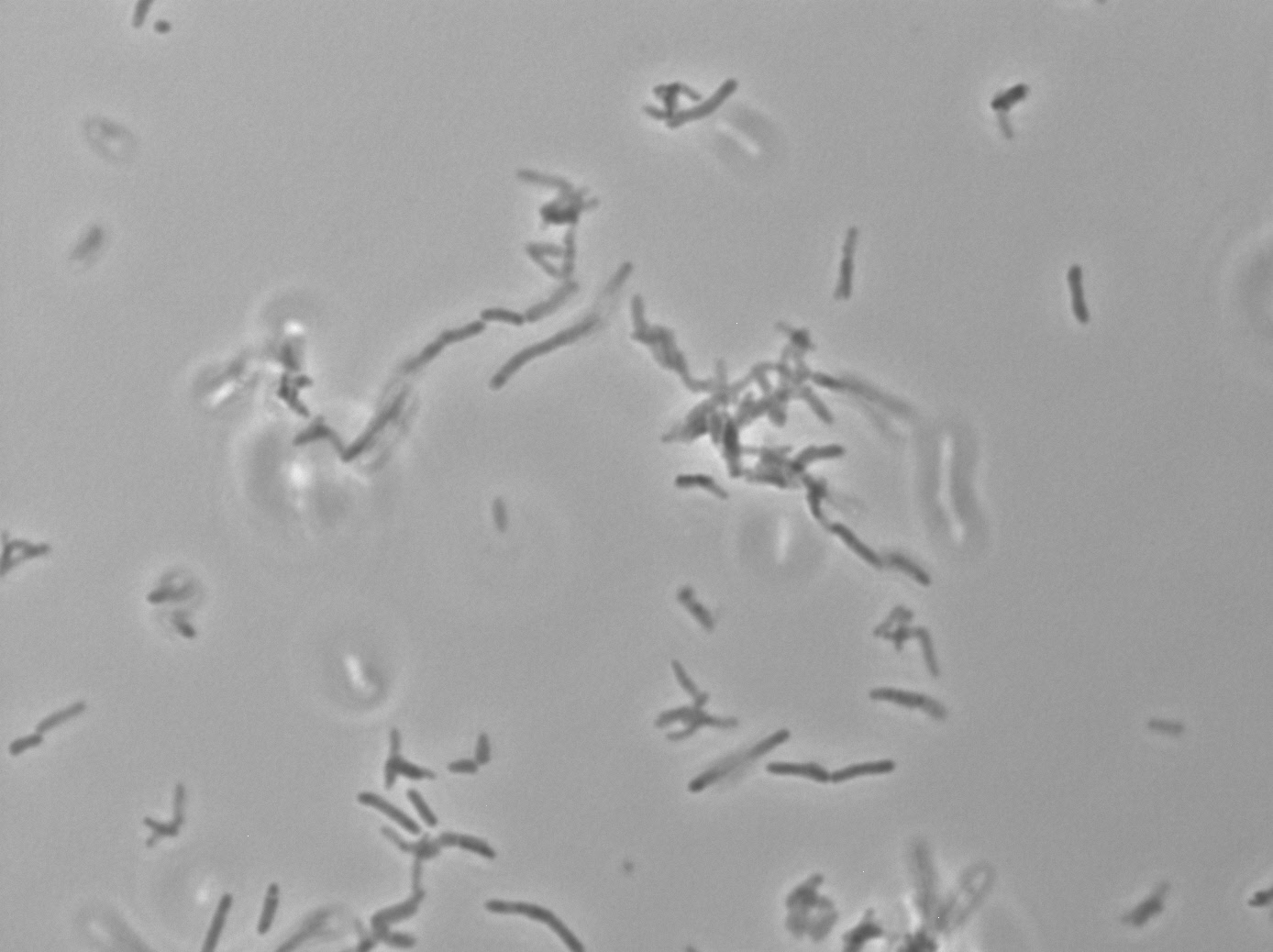

Supplement: Figure 2—source data 1. [file elife-37243-fig2-data1.zip › Figure 2 source data/Figure 2 source data-conventional microscopy (Mtb + HADA)/1. Phase/PC 1.jpg]

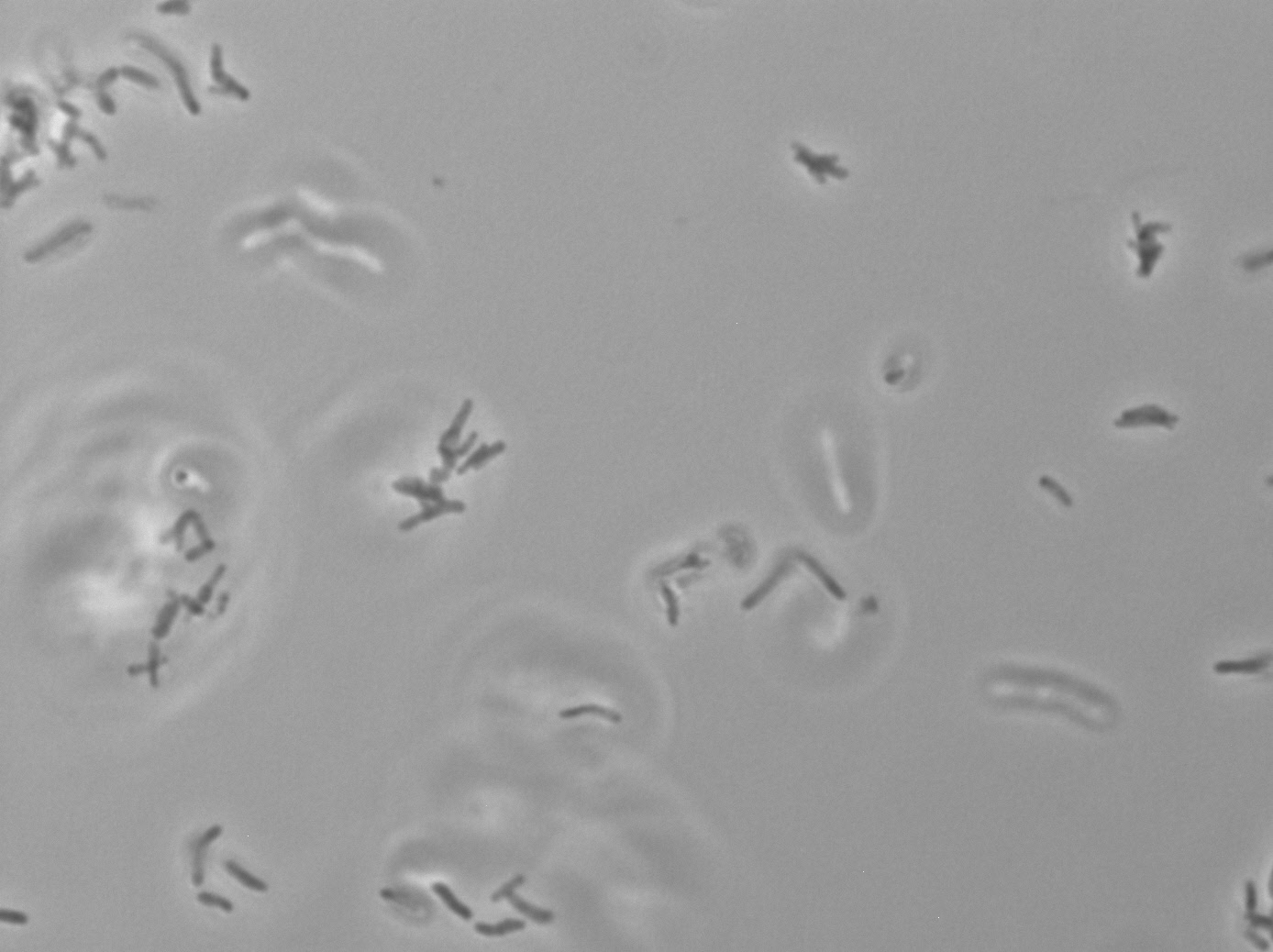

Supplement: Figure 2—source data 1. [file elife-37243-fig2-data1.zip › Figure 2 source data/Figure 2 source data-conventional microscopy (Mtb + HADA)/1. Phase/PC 2.jpg]

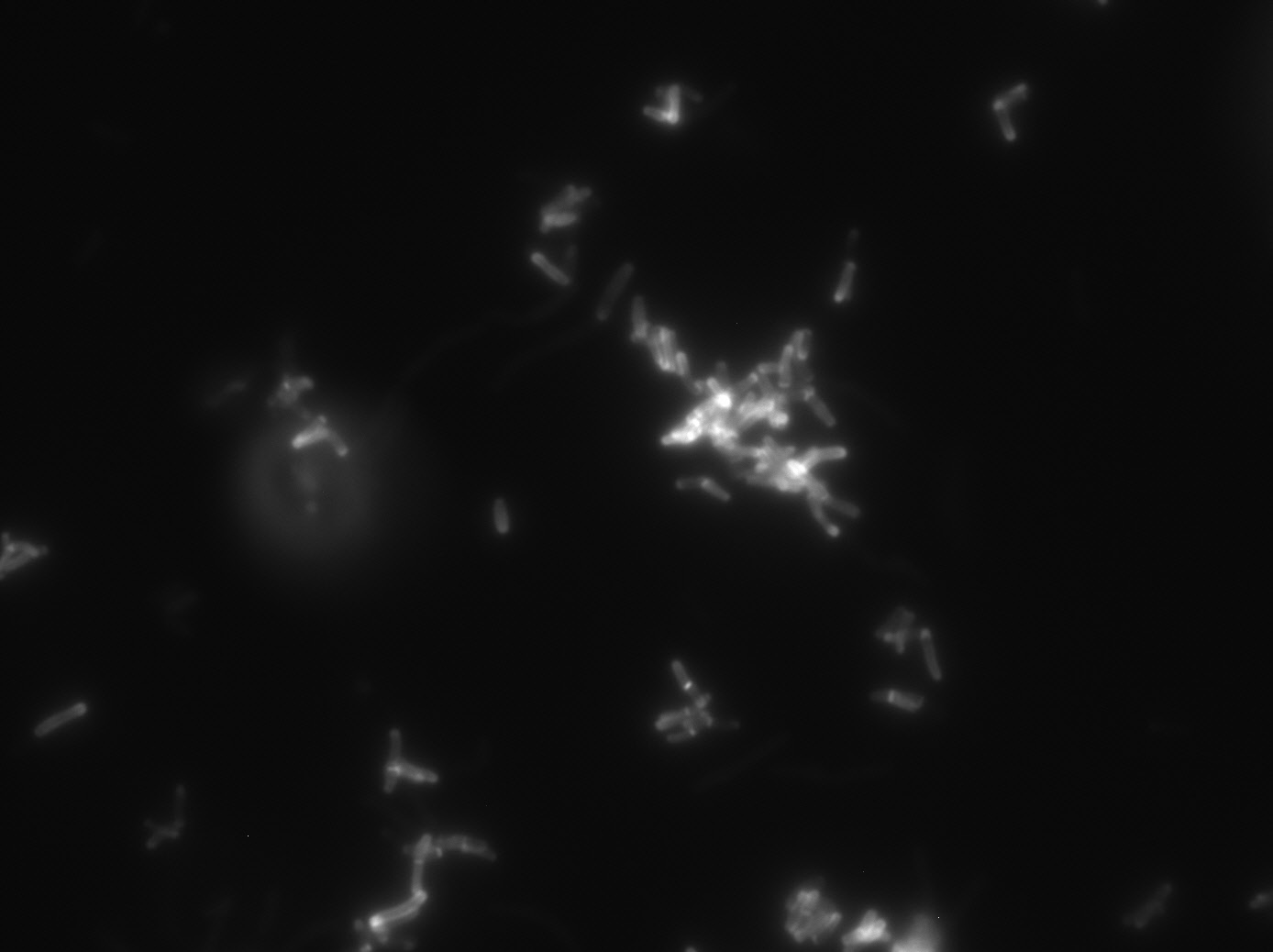

Supplement: Figure 2—source data 1. [file elife-37243-fig2-data1.zip › Figure 2 source data/Figure 2 source data-conventional microscopy (Mtb + HADA)/2. HADA/CFP 1.jpg]

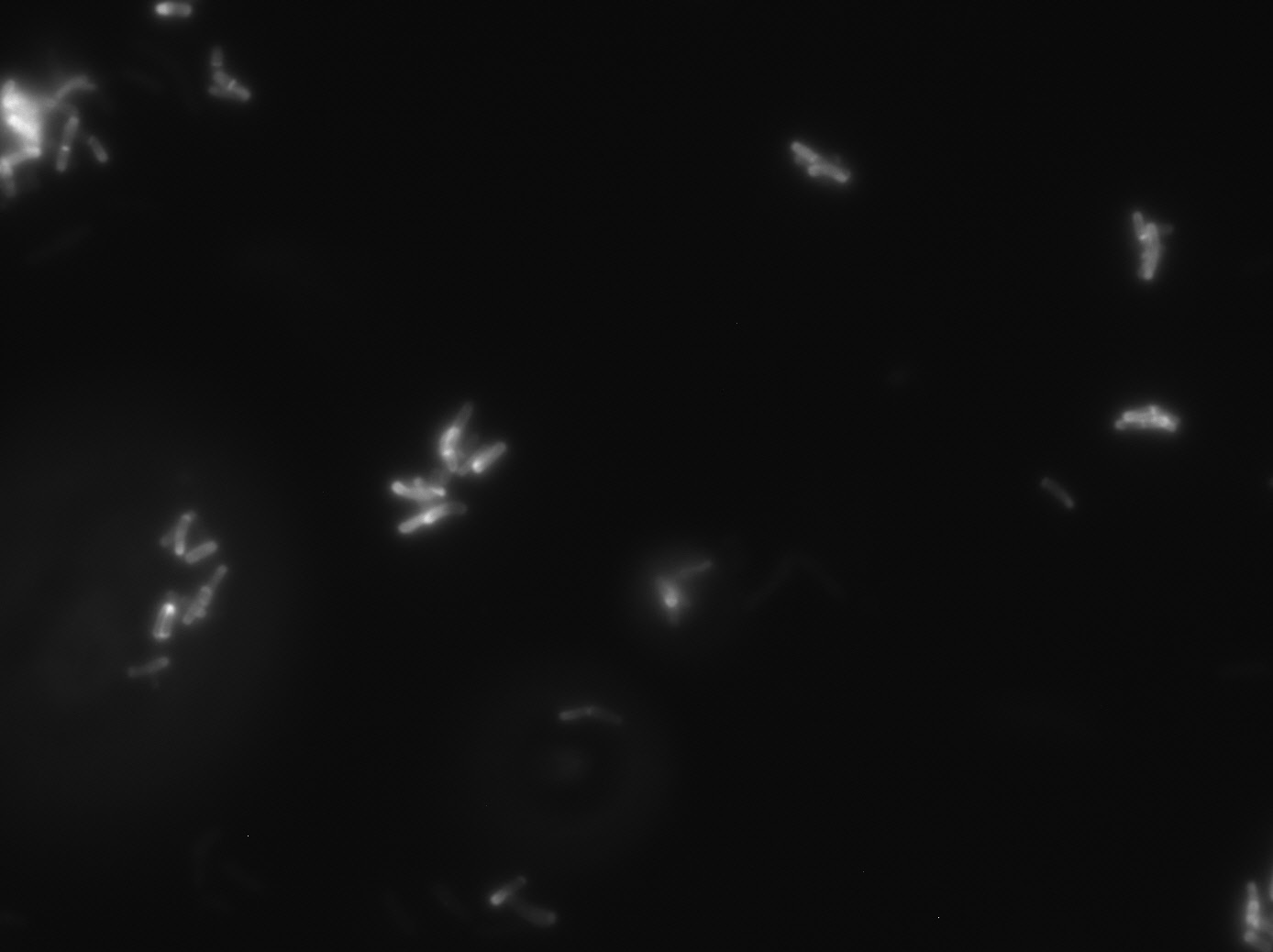

Supplement: Figure 2—source data 1. [file elife-37243-fig2-data1.zip › Figure 2 source data/Figure 2 source data-conventional microscopy (Mtb + HADA)/2. HADA/CFP 2.jpg]

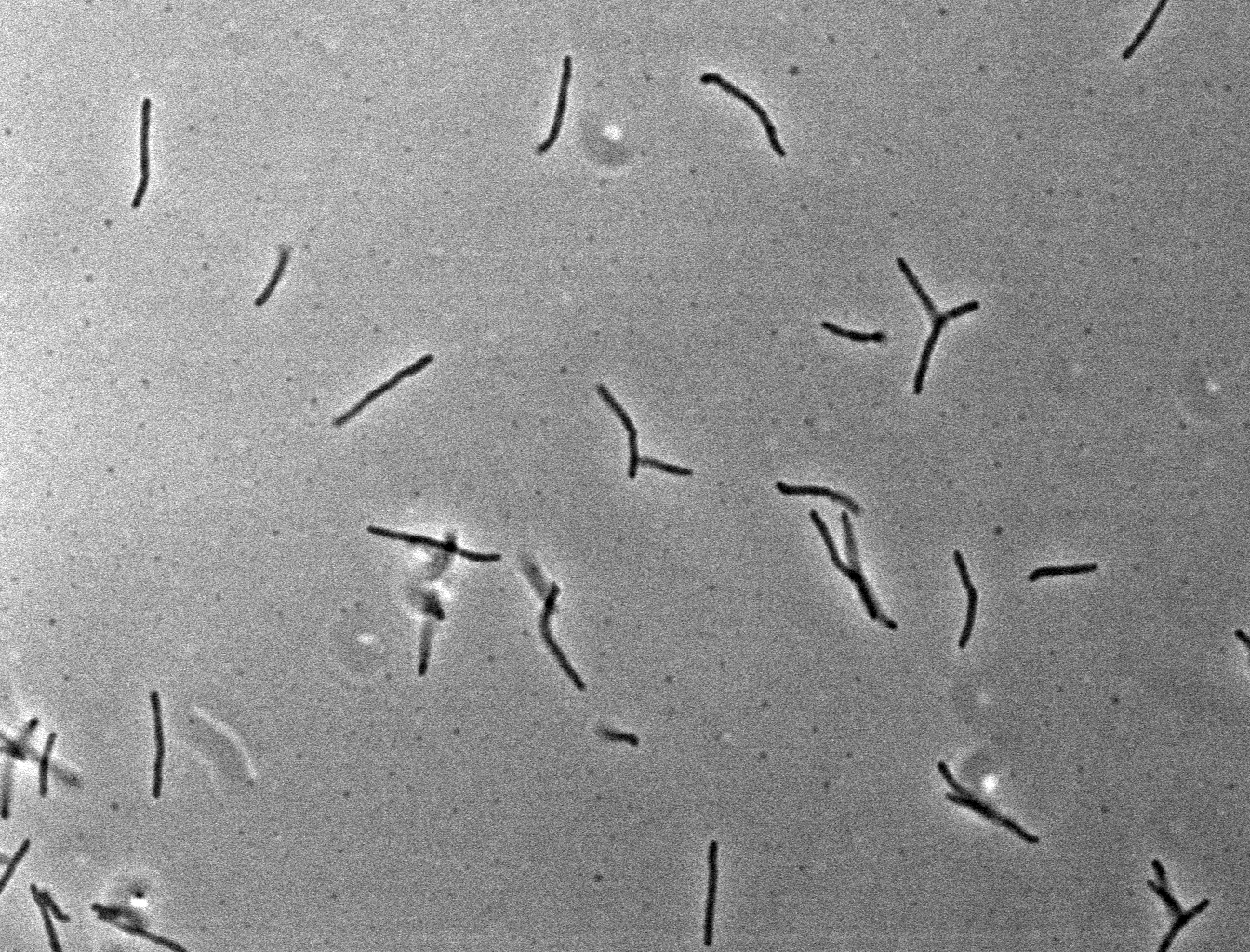

Supplement: Figure 2—source data 1. [file elife-37243-fig2-data1.zip › Figure 2 source data/Figure 2 source data-conventional microscopy (N alk TMM + RADA)/1. Phase/1.tif]

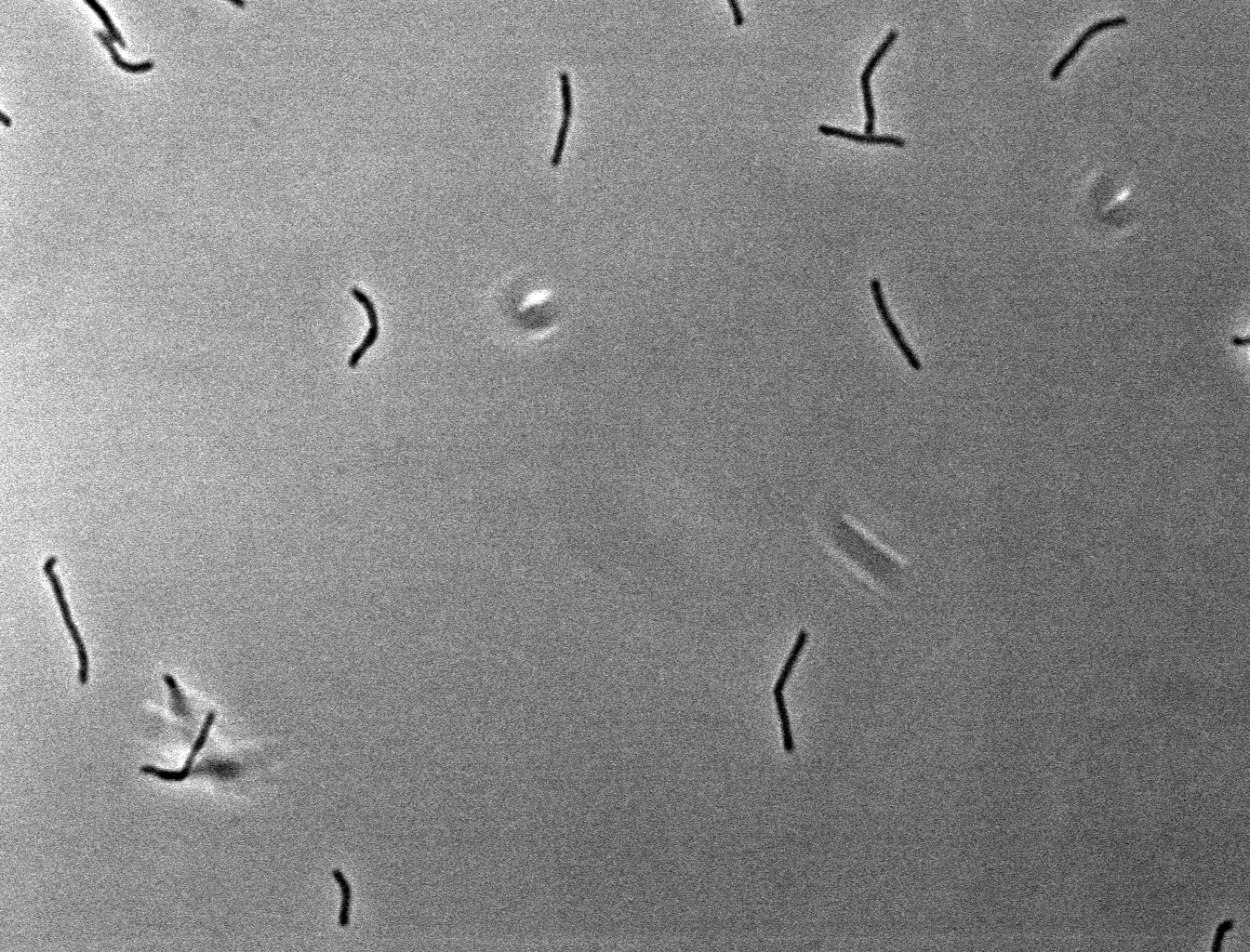

Supplement: Figure 2—source data 1. [file elife-37243-fig2-data1.zip › Figure 2 source data/Figure 2 source data-conventional microscopy (N alk TMM + RADA)/1. Phase/10.tif]

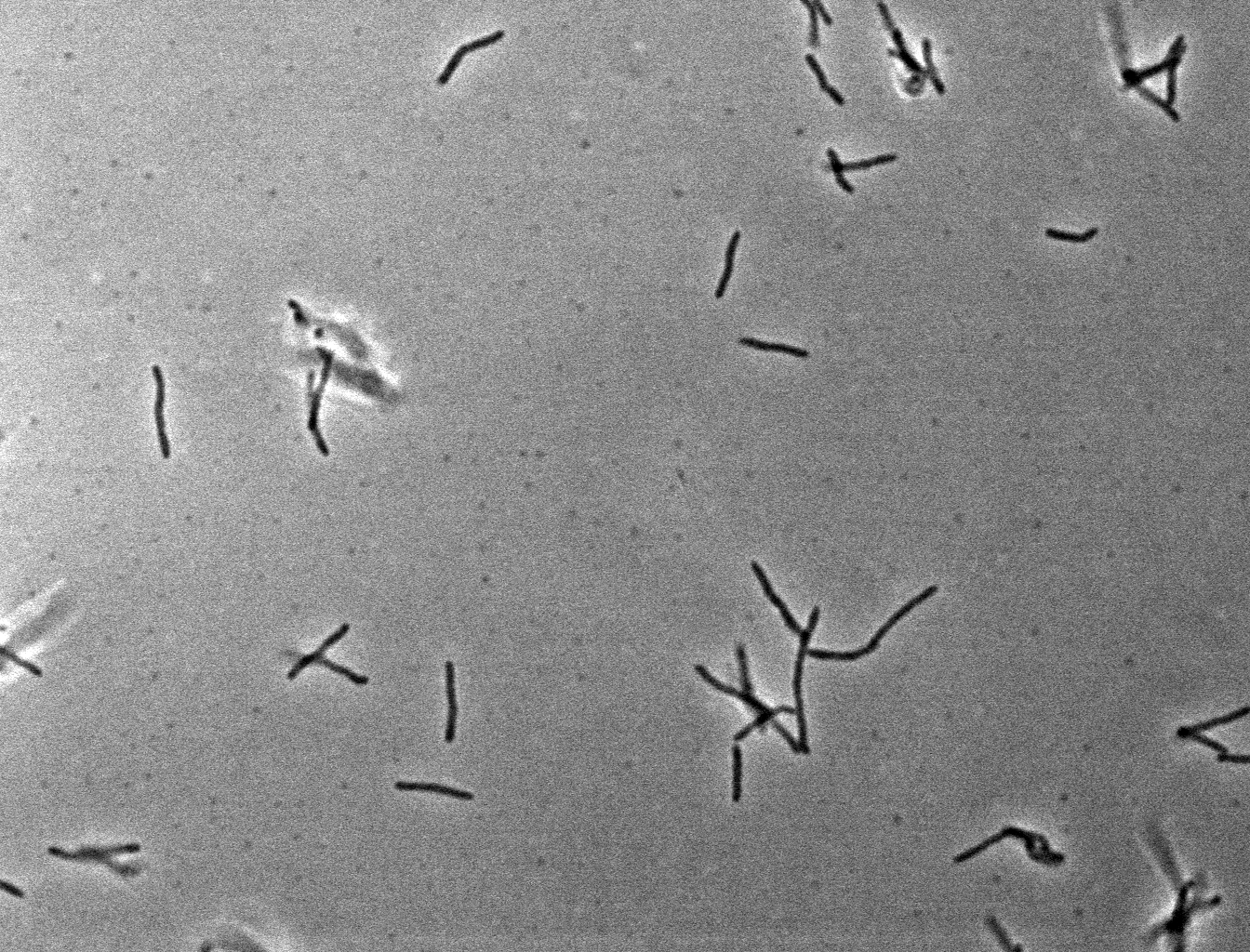

Supplement: Figure 2—source data 1. [file elife-37243-fig2-data1.zip › Figure 2 source data/Figure 2 source data-conventional microscopy (N alk TMM + RADA)/1. Phase/2.tif]

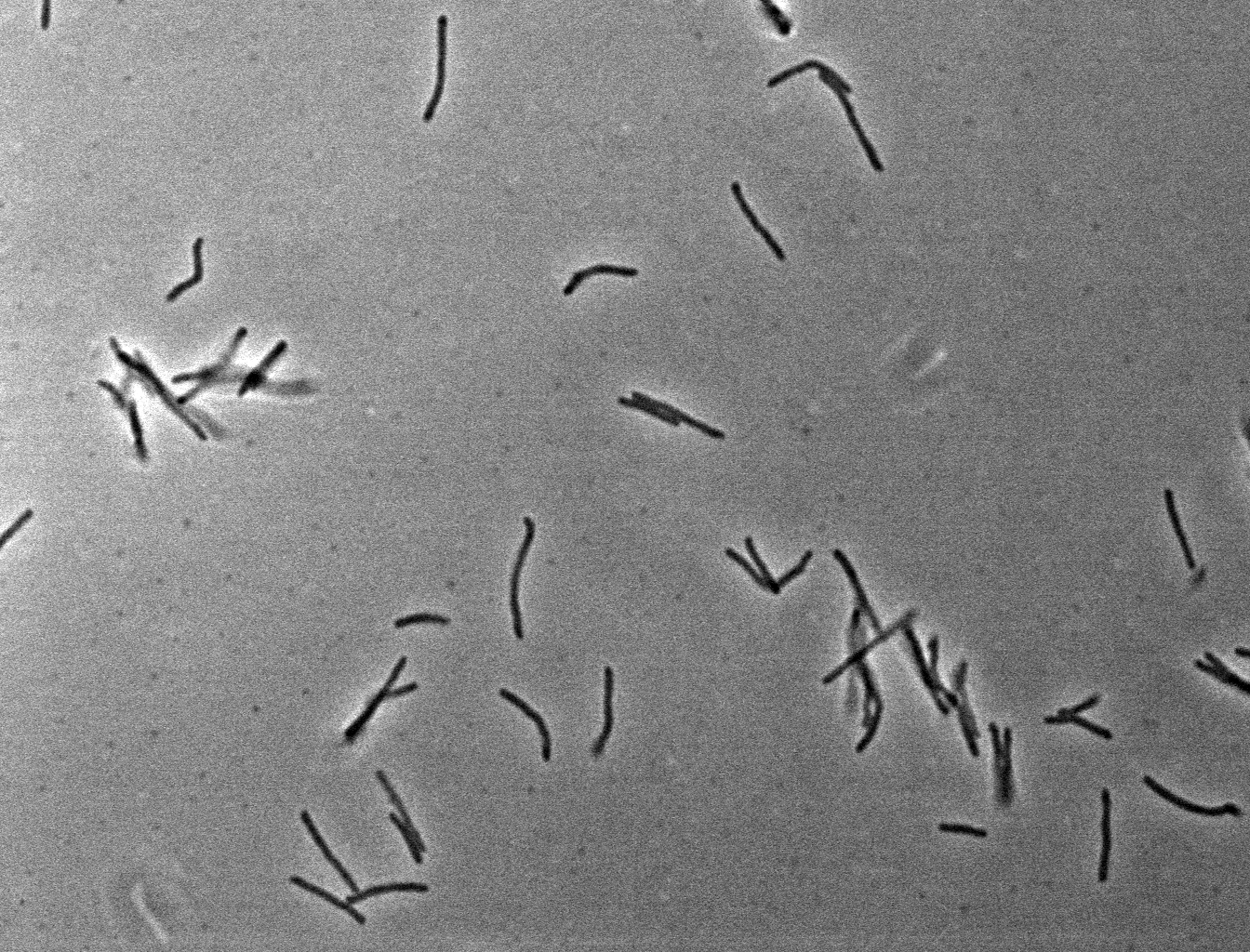

Supplement: Figure 2—source data 1. [file elife-37243-fig2-data1.zip › Figure 2 source data/Figure 2 source data-conventional microscopy (N alk TMM + RADA)/1. Phase/3.tif]

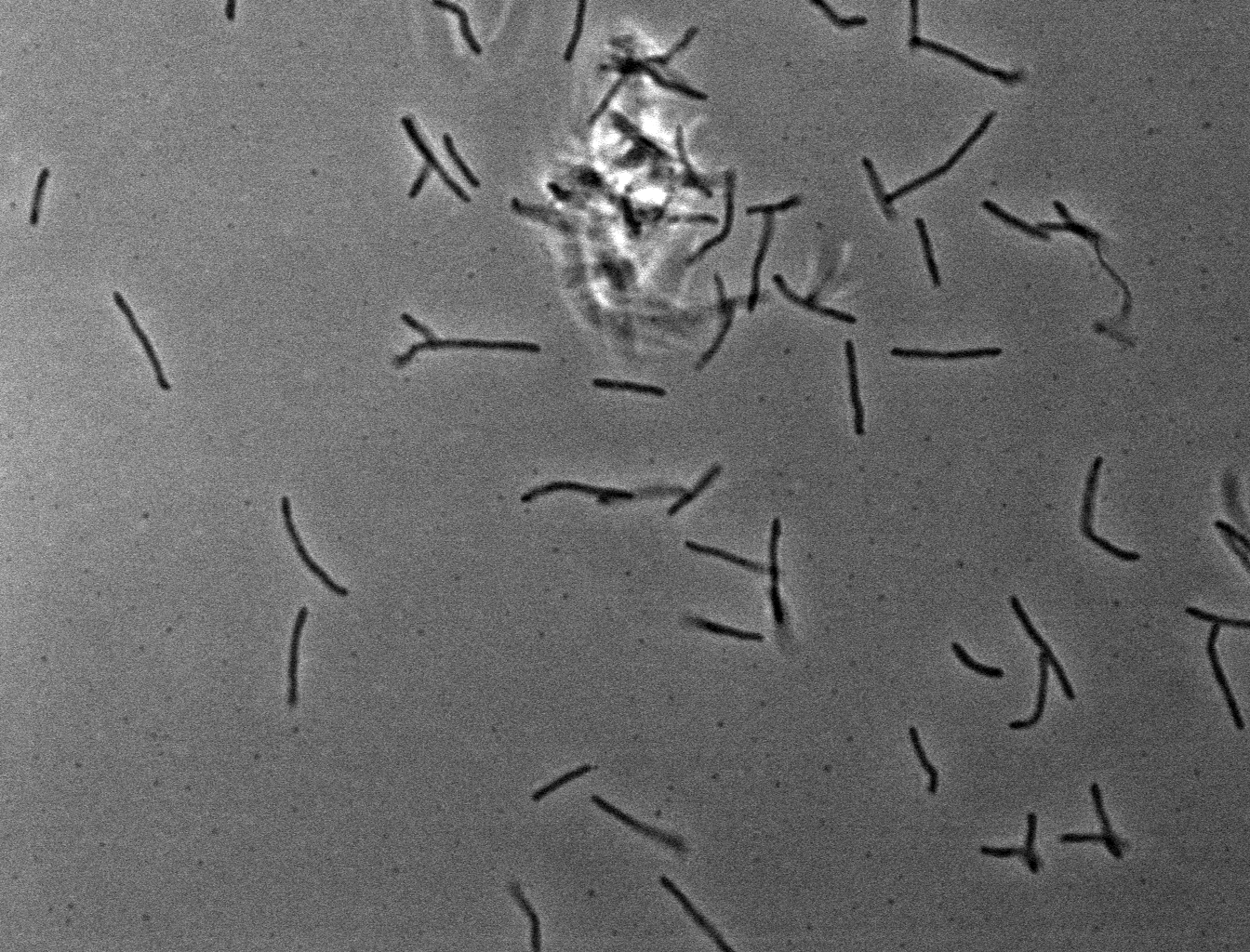

Supplement: Figure 2—source data 1. [file elife-37243-fig2-data1.zip › Figure 2 source data/Figure 2 source data-conventional microscopy (N alk TMM + RADA)/1. Phase/4.tif]

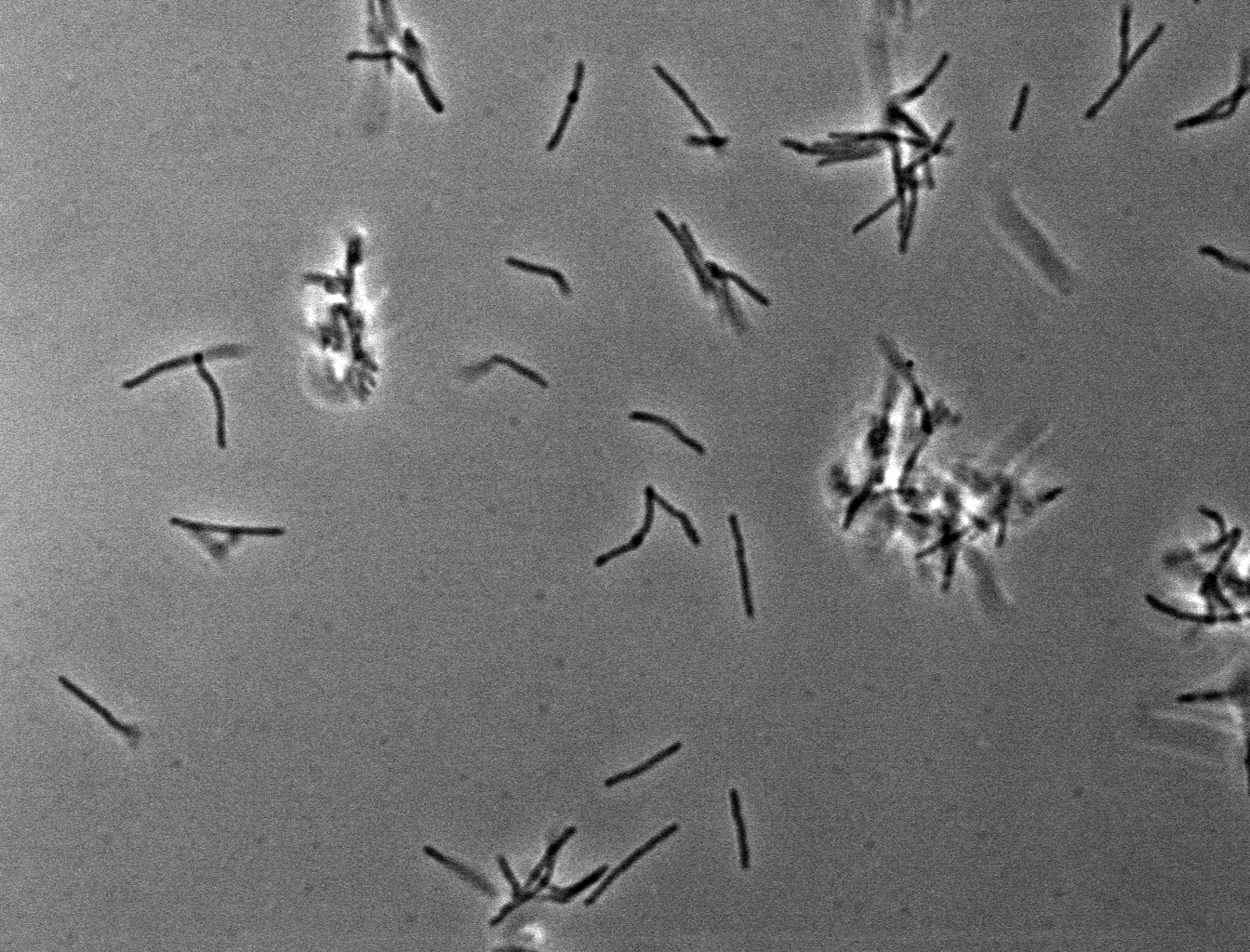

Supplement: Figure 2—source data 1. [file elife-37243-fig2-data1.zip › Figure 2 source data/Figure 2 source data-conventional microscopy (N alk TMM + RADA)/1. Phase/5.tif]

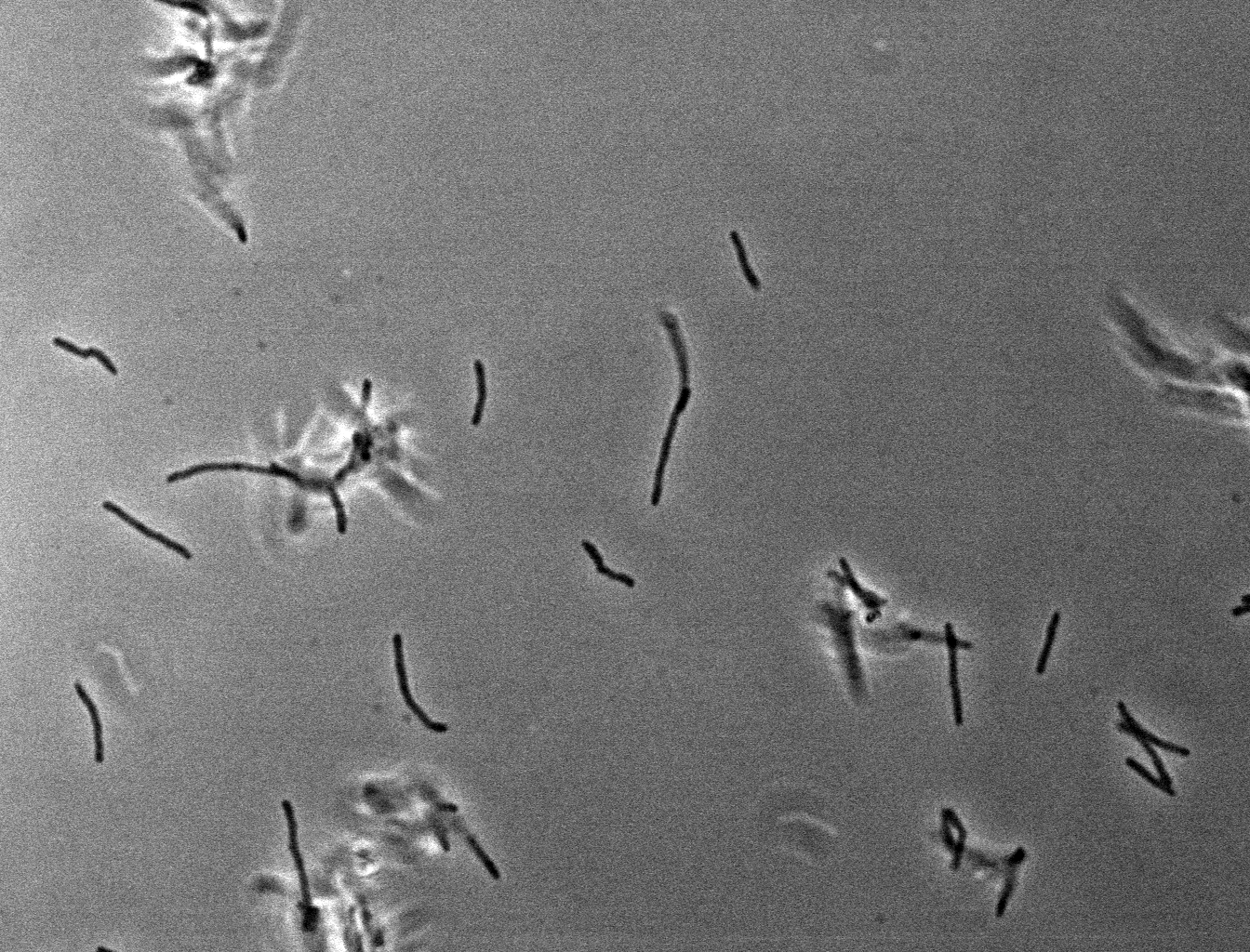

Supplement: Figure 2—source data 1. [file elife-37243-fig2-data1.zip › Figure 2 source data/Figure 2 source data-conventional microscopy (N alk TMM + RADA)/1. Phase/6.tif]

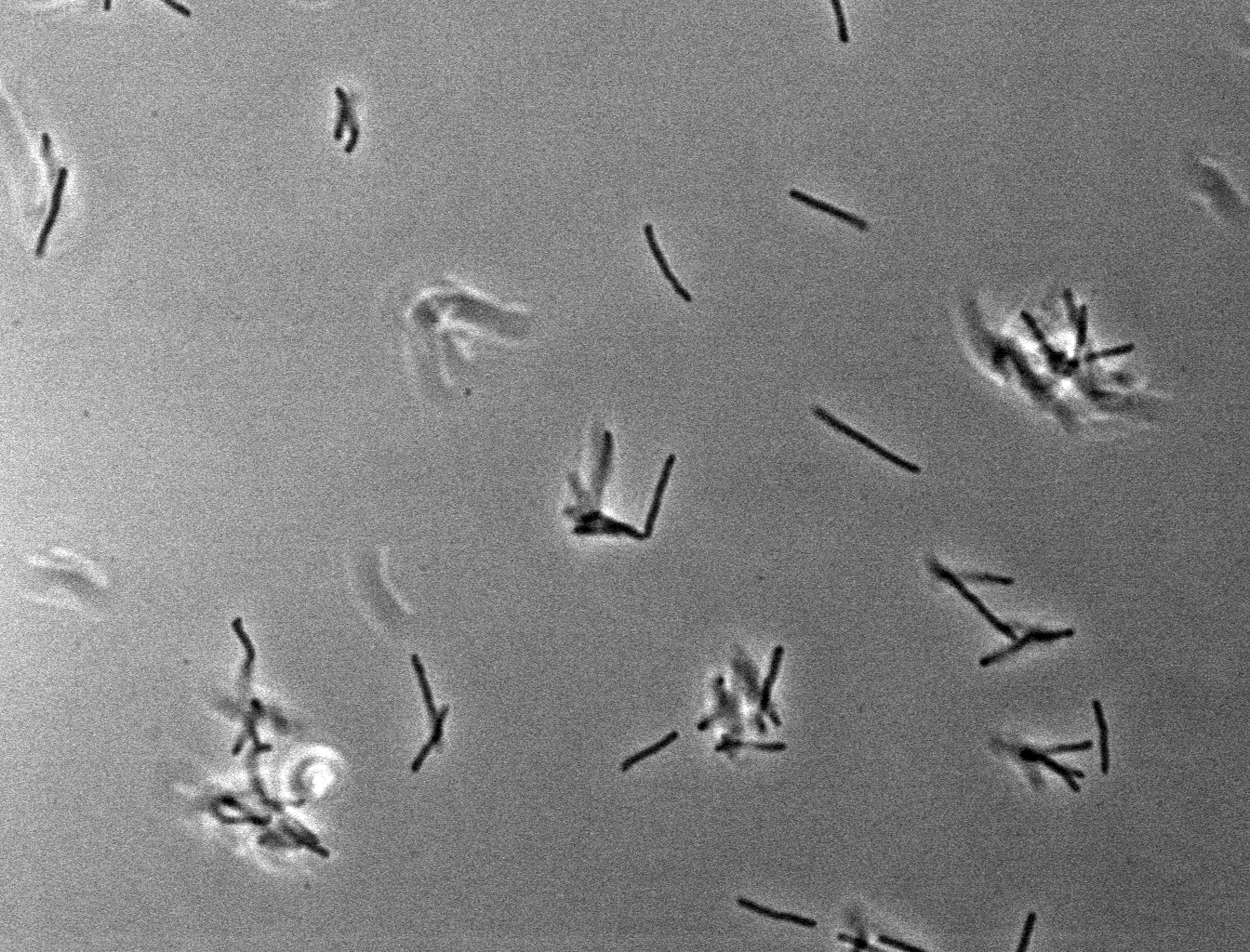

Supplement: Figure 2—source data 1. [file elife-37243-fig2-data1.zip › Figure 2 source data/Figure 2 source data-conventional microscopy (N alk TMM + RADA)/1. Phase/7.tif]

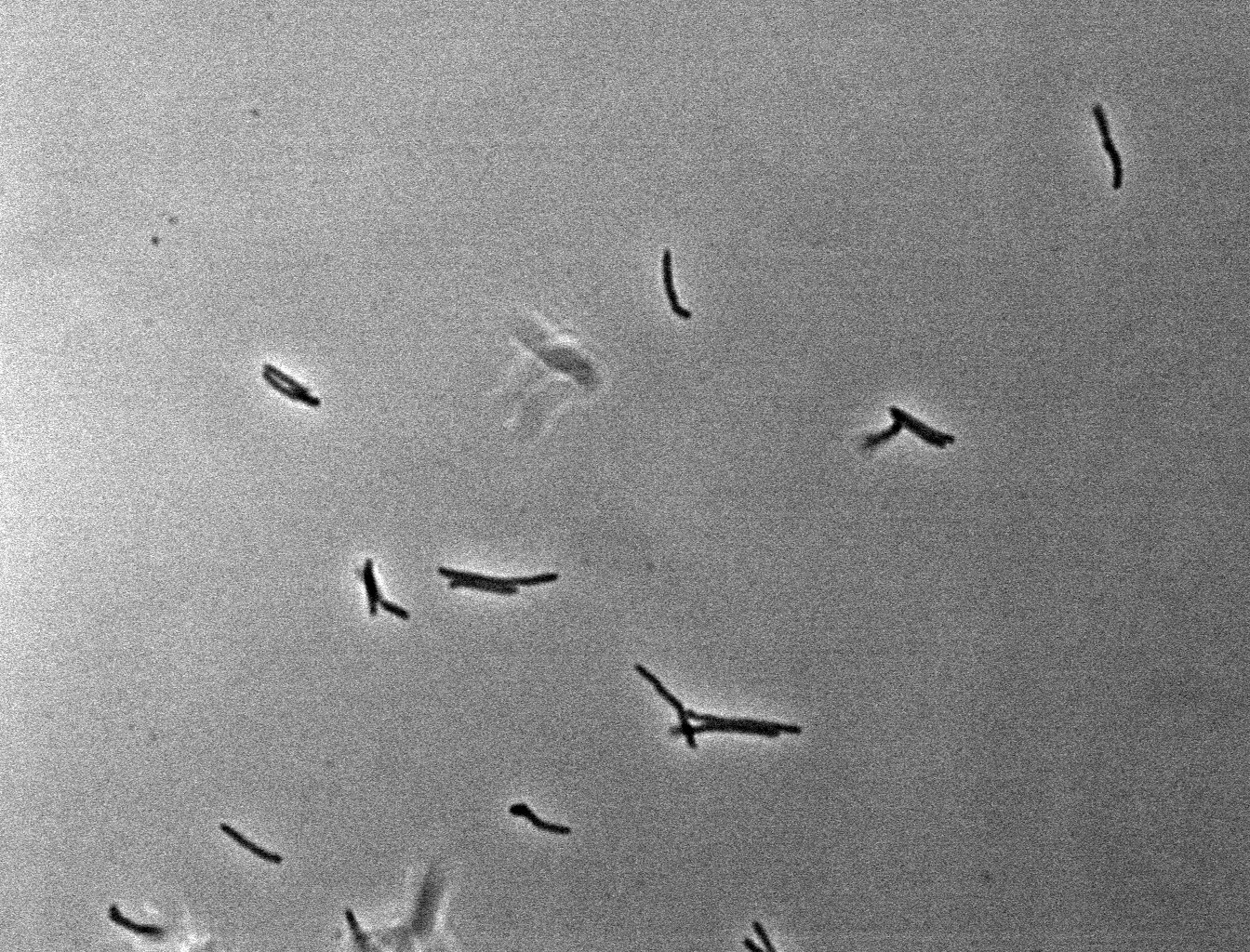

Supplement: Figure 2—source data 1. [file elife-37243-fig2-data1.zip › Figure 2 source data/Figure 2 source data-conventional microscopy (N alk TMM + RADA)/1. Phase/8.tif]

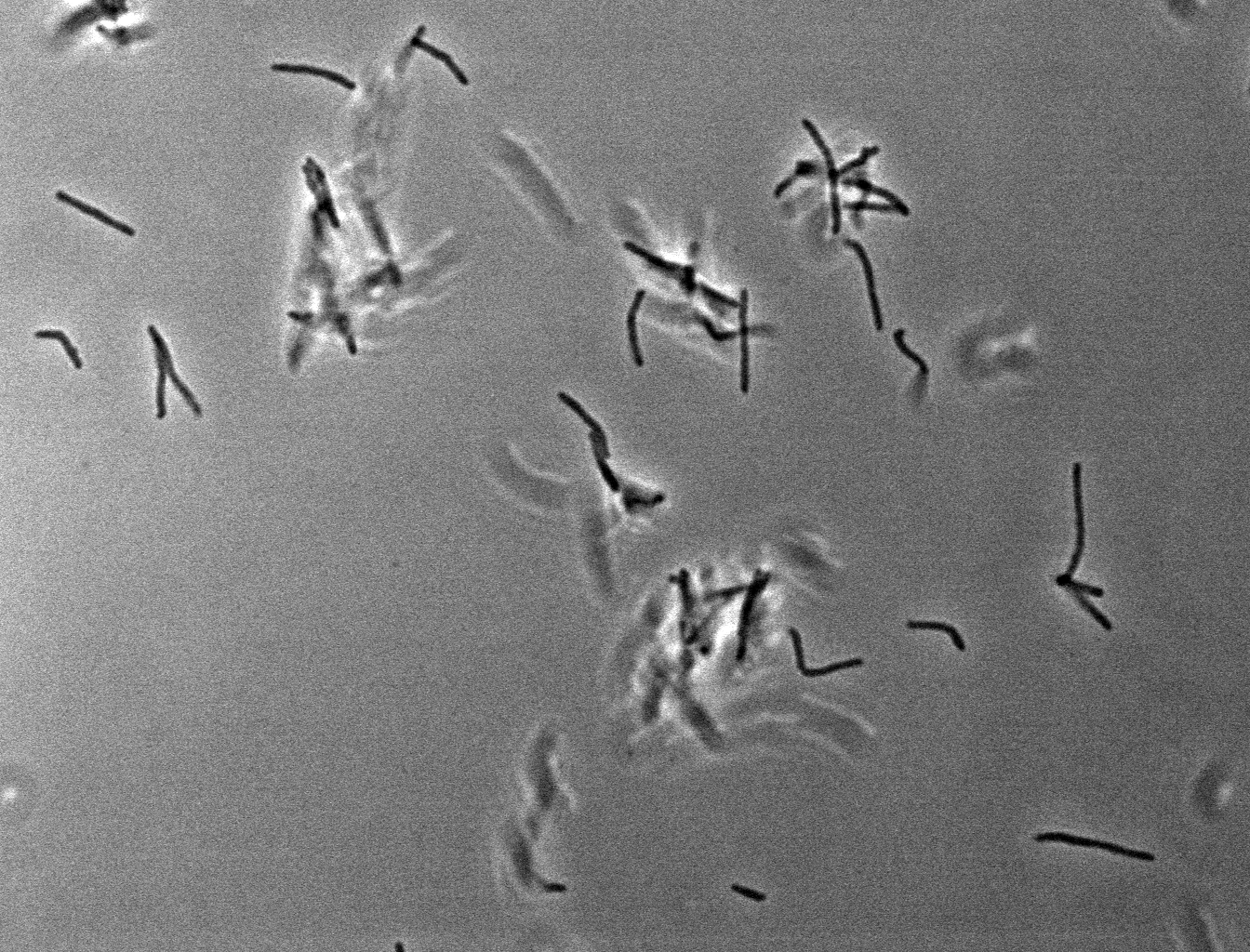

Supplement: Figure 2—source data 1. [file elife-37243-fig2-data1.zip › Figure 2 source data/Figure 2 source data-conventional microscopy (N alk TMM + RADA)/1. Phase/9.tif]

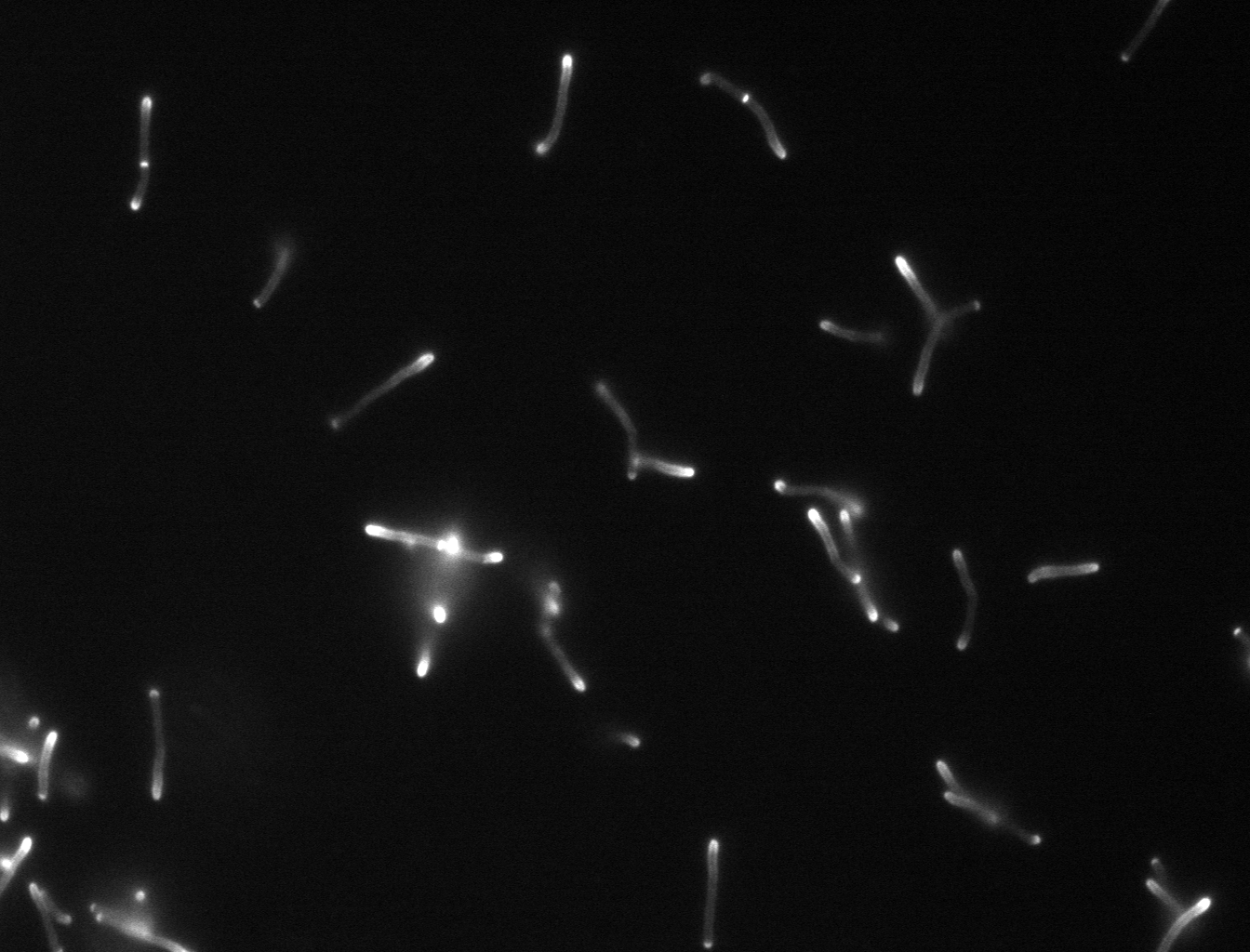

Supplement: Figure 2—source data 1. [file elife-37243-fig2-data1.zip › Figure 2 source data/Figure 2 source data-conventional microscopy (N alk TMM + RADA)/2. NAlkTMM/1.tif]

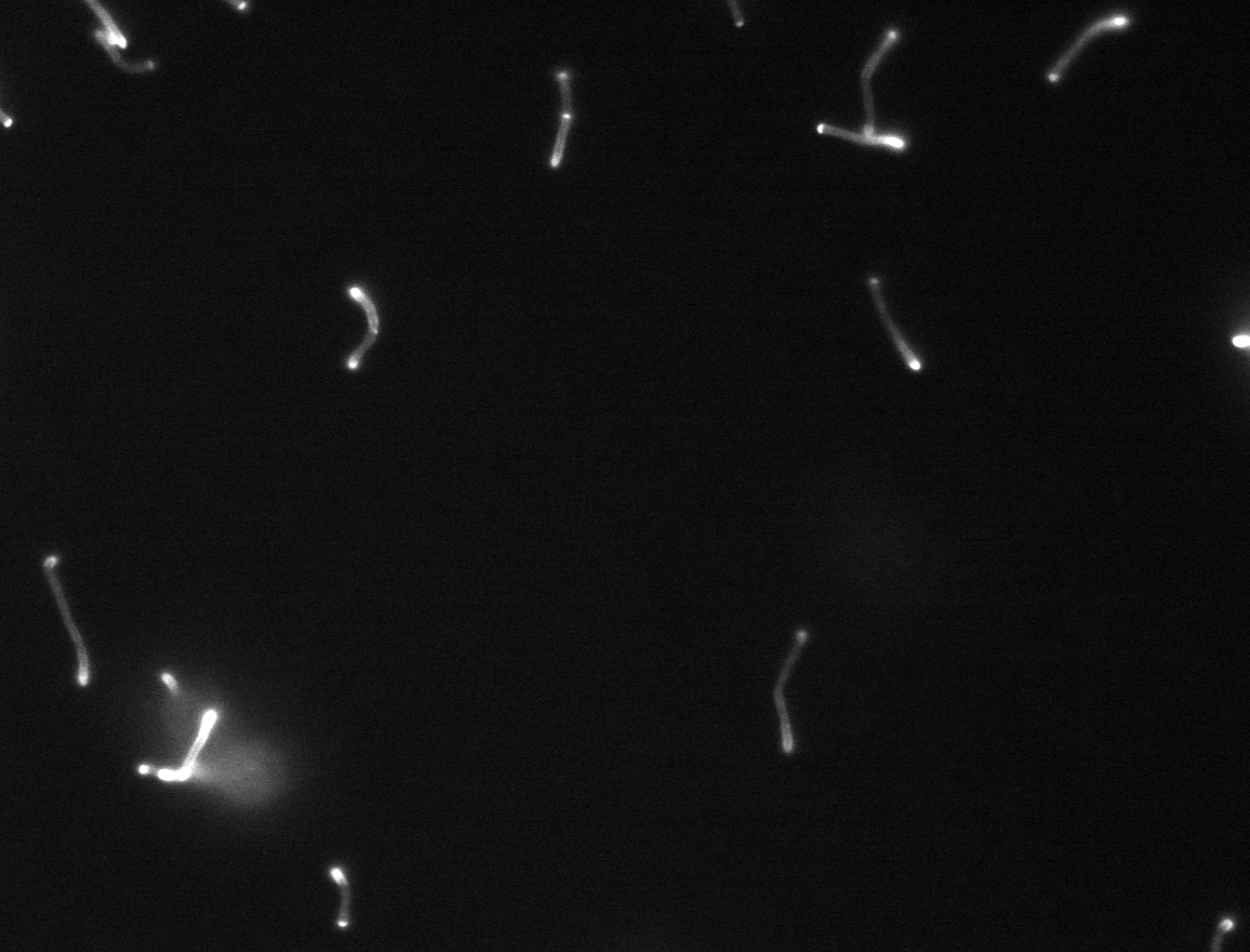

Supplement: Figure 2—source data 1. [file elife-37243-fig2-data1.zip › Figure 2 source data/Figure 2 source data-conventional microscopy (N alk TMM + RADA)/2. NAlkTMM/10.tif]

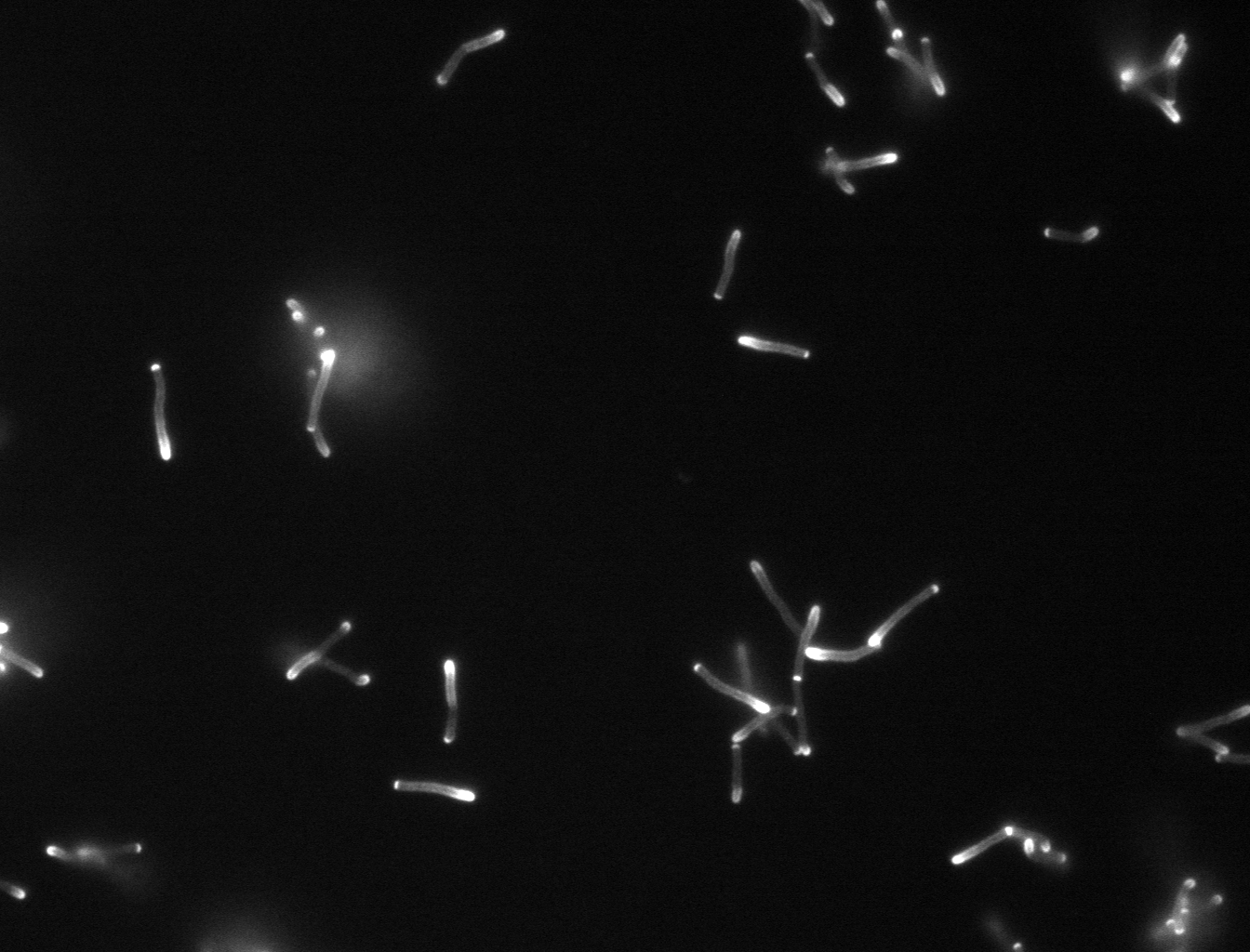

Supplement: Figure 2—source data 1. [file elife-37243-fig2-data1.zip › Figure 2 source data/Figure 2 source data-conventional microscopy (N alk TMM + RADA)/2. NAlkTMM/2.tif]

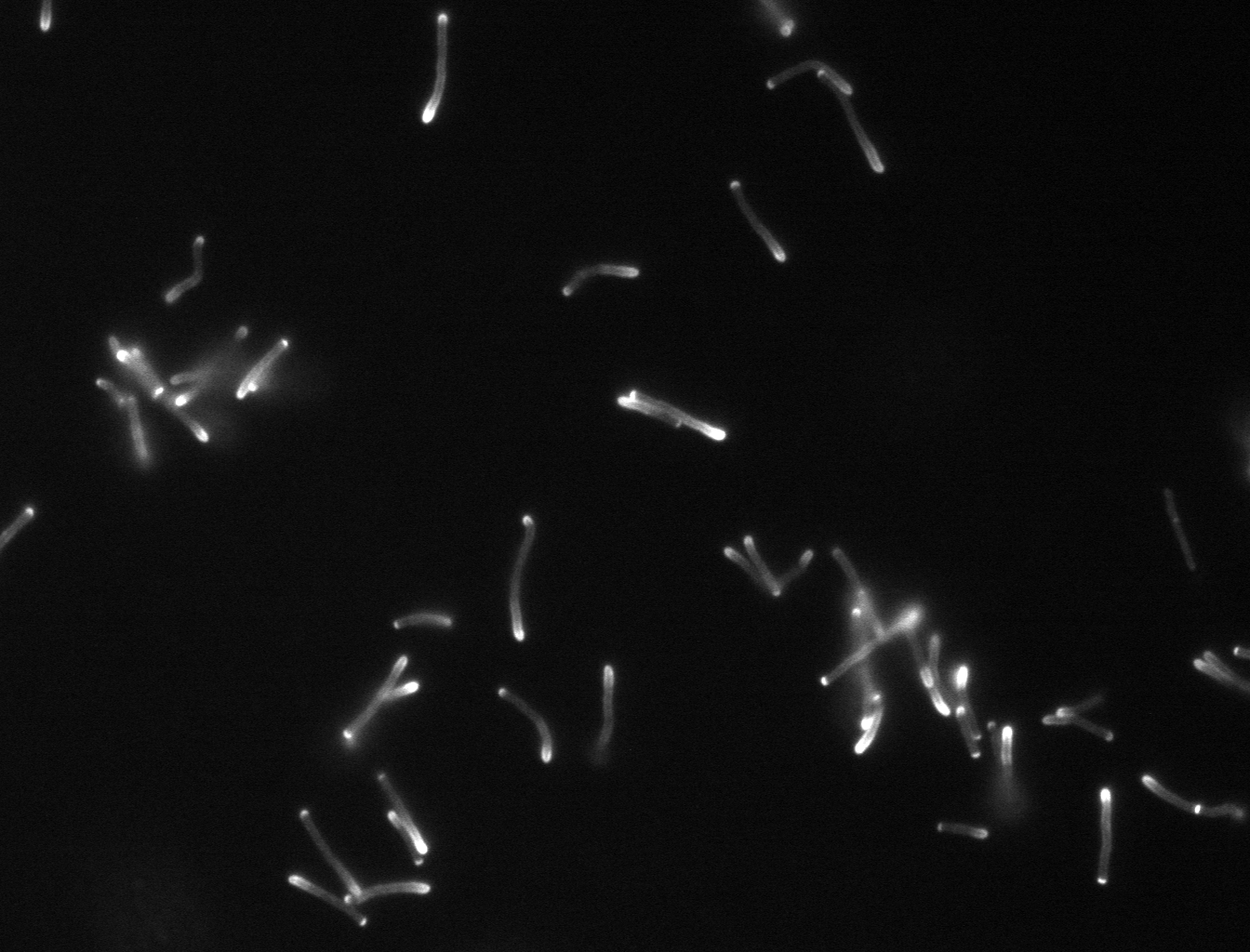

Supplement: Figure 2—source data 1. [file elife-37243-fig2-data1.zip › Figure 2 source data/Figure 2 source data-conventional microscopy (N alk TMM + RADA)/2. NAlkTMM/3.tif]

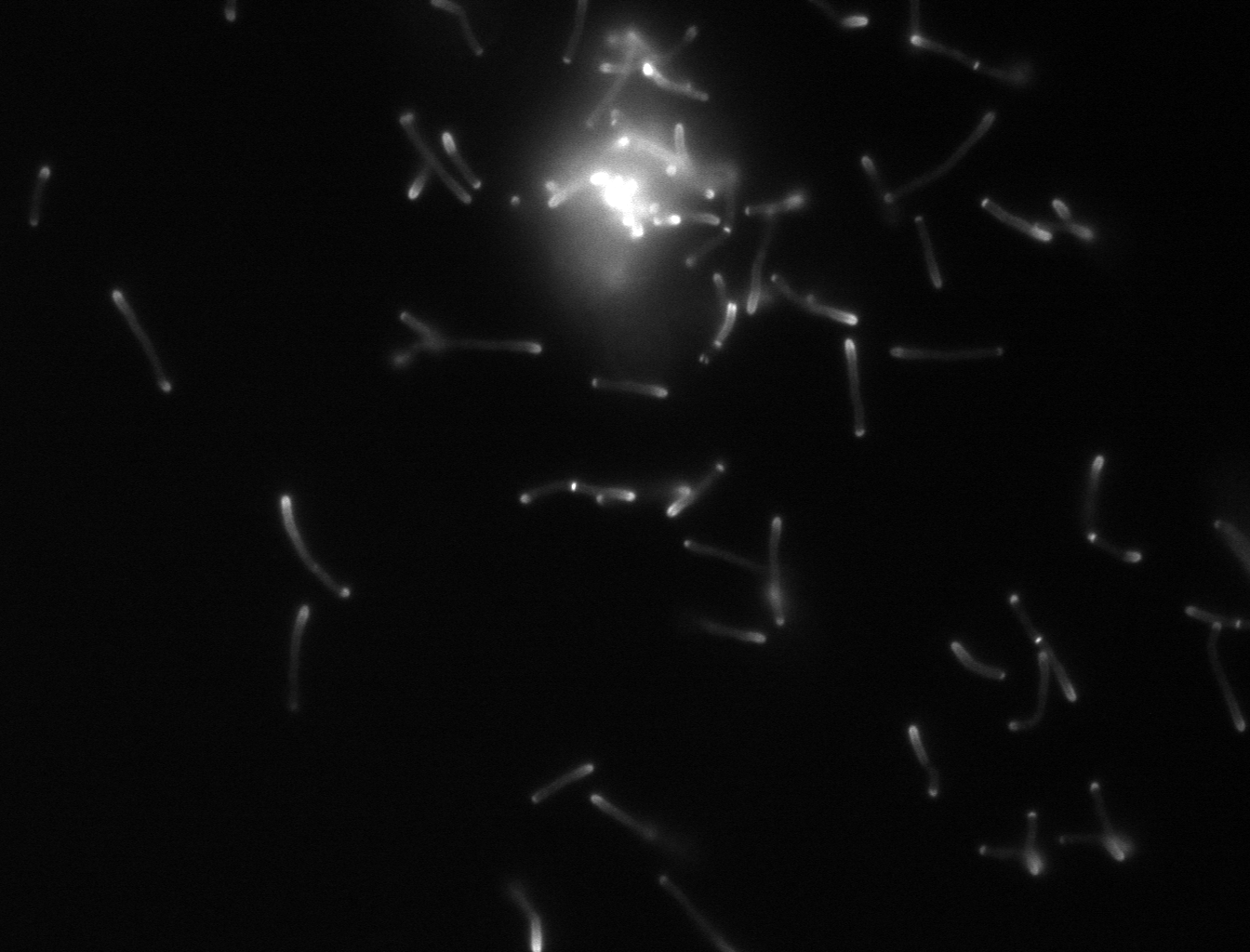

Supplement: Figure 2—source data 1. [file elife-37243-fig2-data1.zip › Figure 2 source data/Figure 2 source data-conventional microscopy (N alk TMM + RADA)/2. NAlkTMM/4.tif]

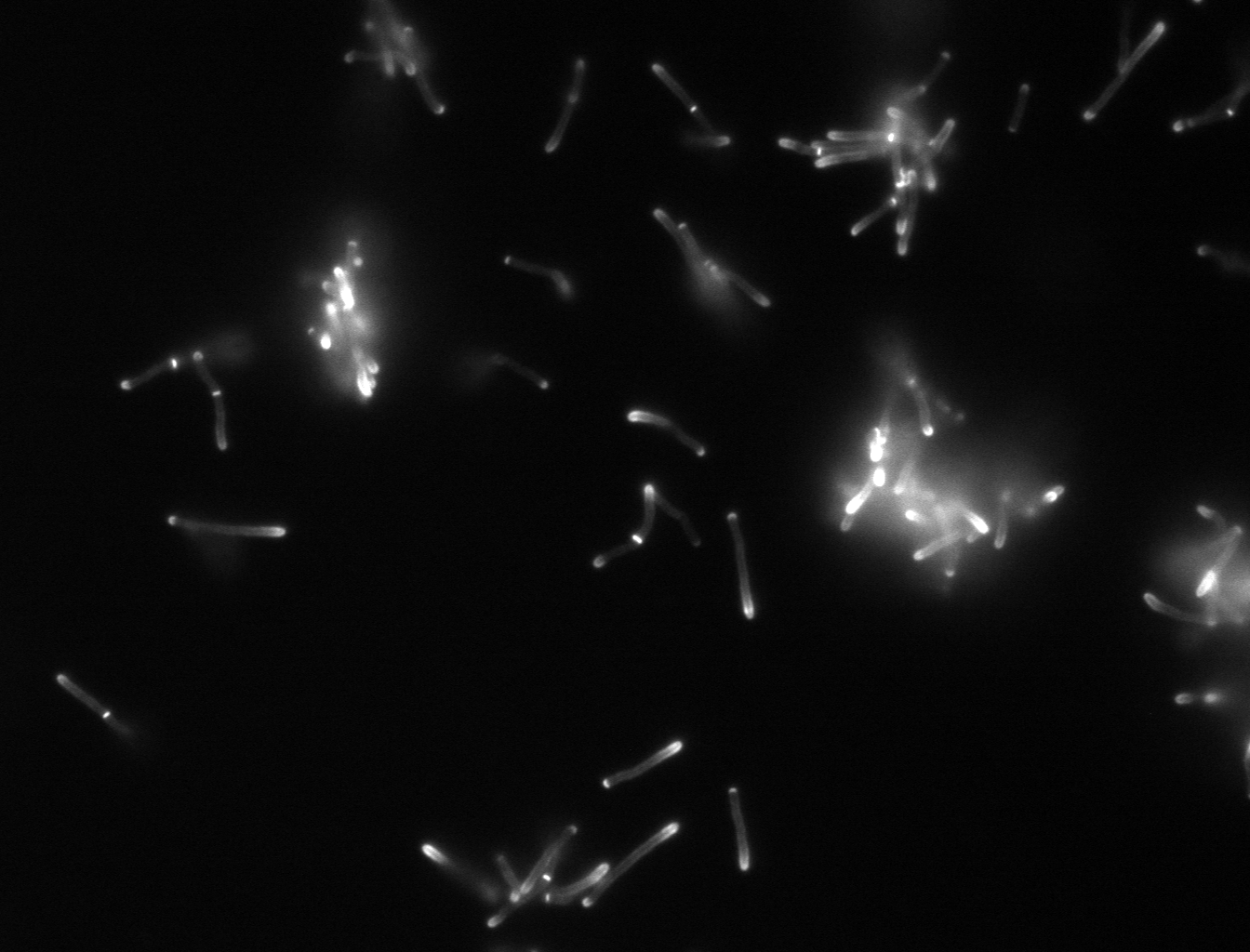

Supplement: Figure 2—source data 1. [file elife-37243-fig2-data1.zip › Figure 2 source data/Figure 2 source data-conventional microscopy (N alk TMM + RADA)/2. NAlkTMM/5.tif]

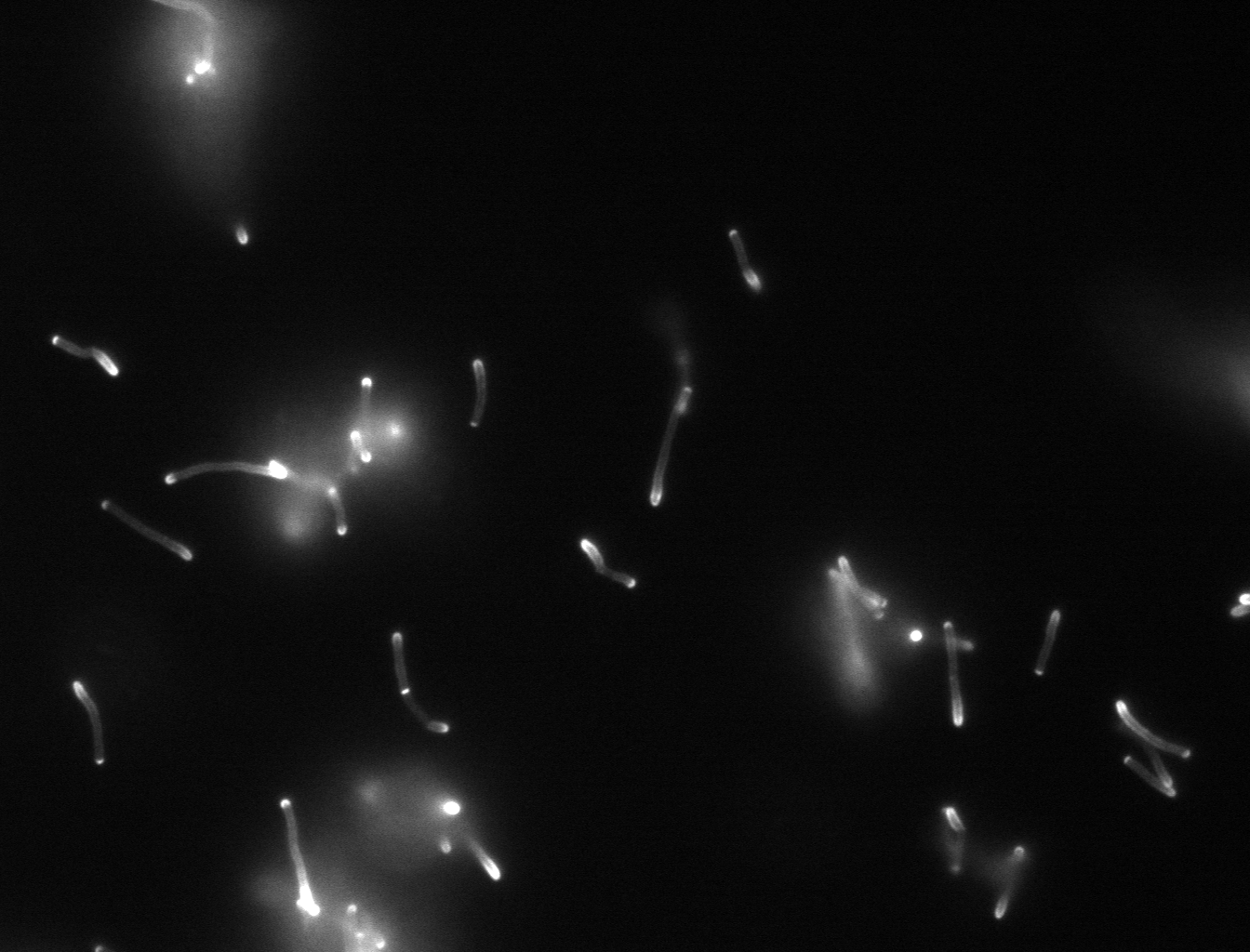

Supplement: Figure 2—source data 1. [file elife-37243-fig2-data1.zip › Figure 2 source data/Figure 2 source data-conventional microscopy (N alk TMM + RADA)/2. NAlkTMM/6.tif]

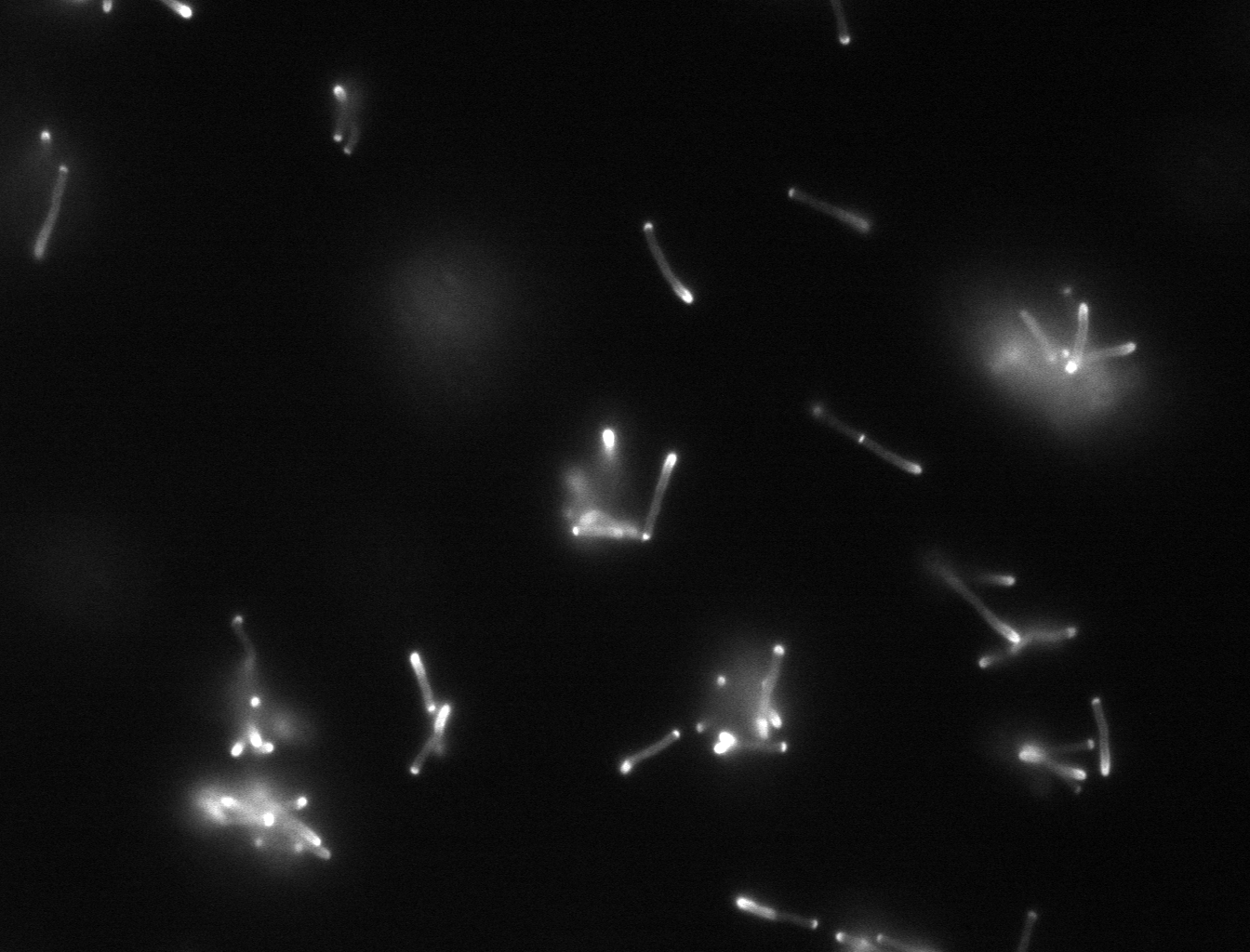

Supplement: Figure 2—source data 1. [file elife-37243-fig2-data1.zip › Figure 2 source data/Figure 2 source data-conventional microscopy (N alk TMM + RADA)/2. NAlkTMM/7.tif]

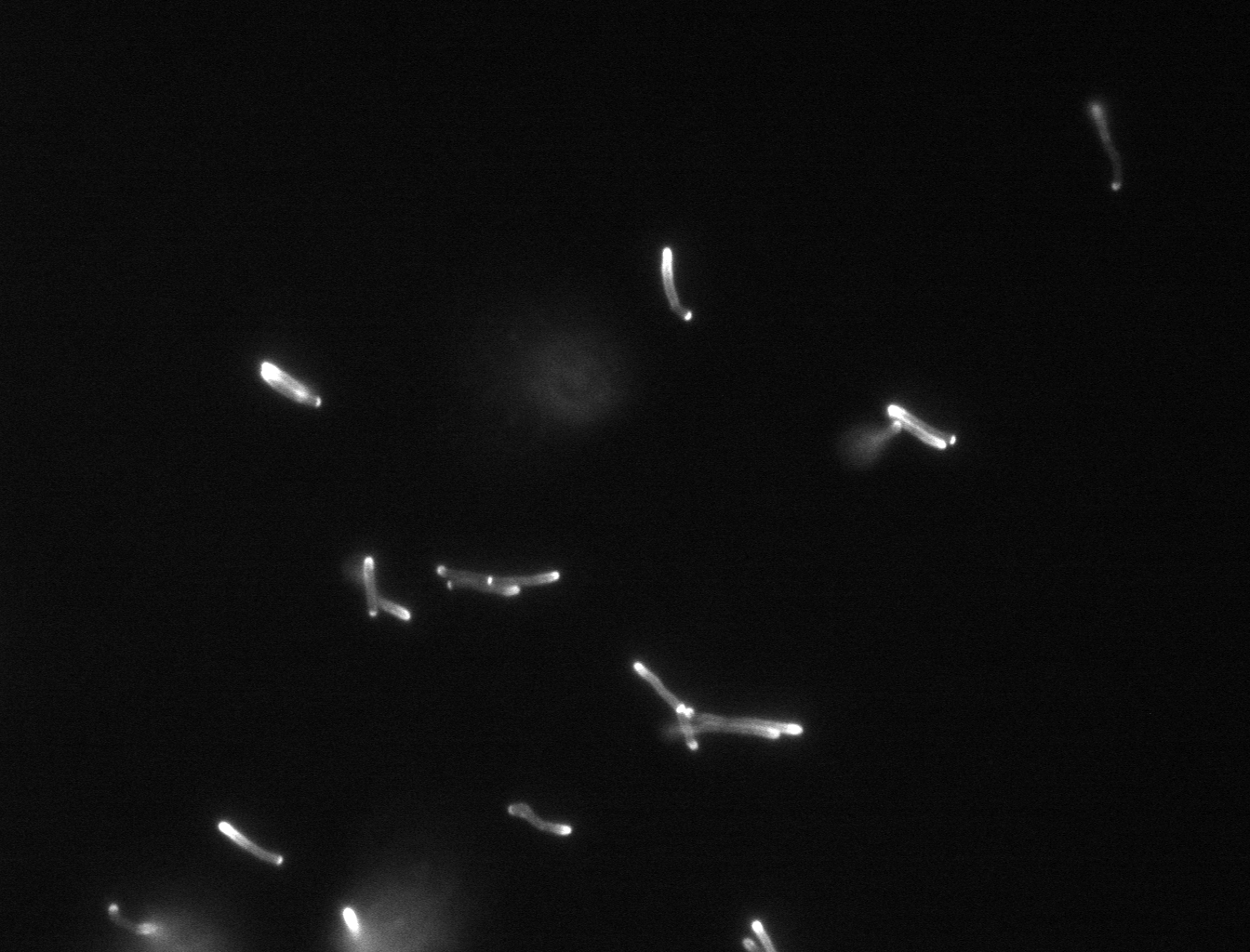

Supplement: Figure 2—source data 1. [file elife-37243-fig2-data1.zip › Figure 2 source data/Figure 2 source data-conventional microscopy (N alk TMM + RADA)/2. NAlkTMM/8.tif]

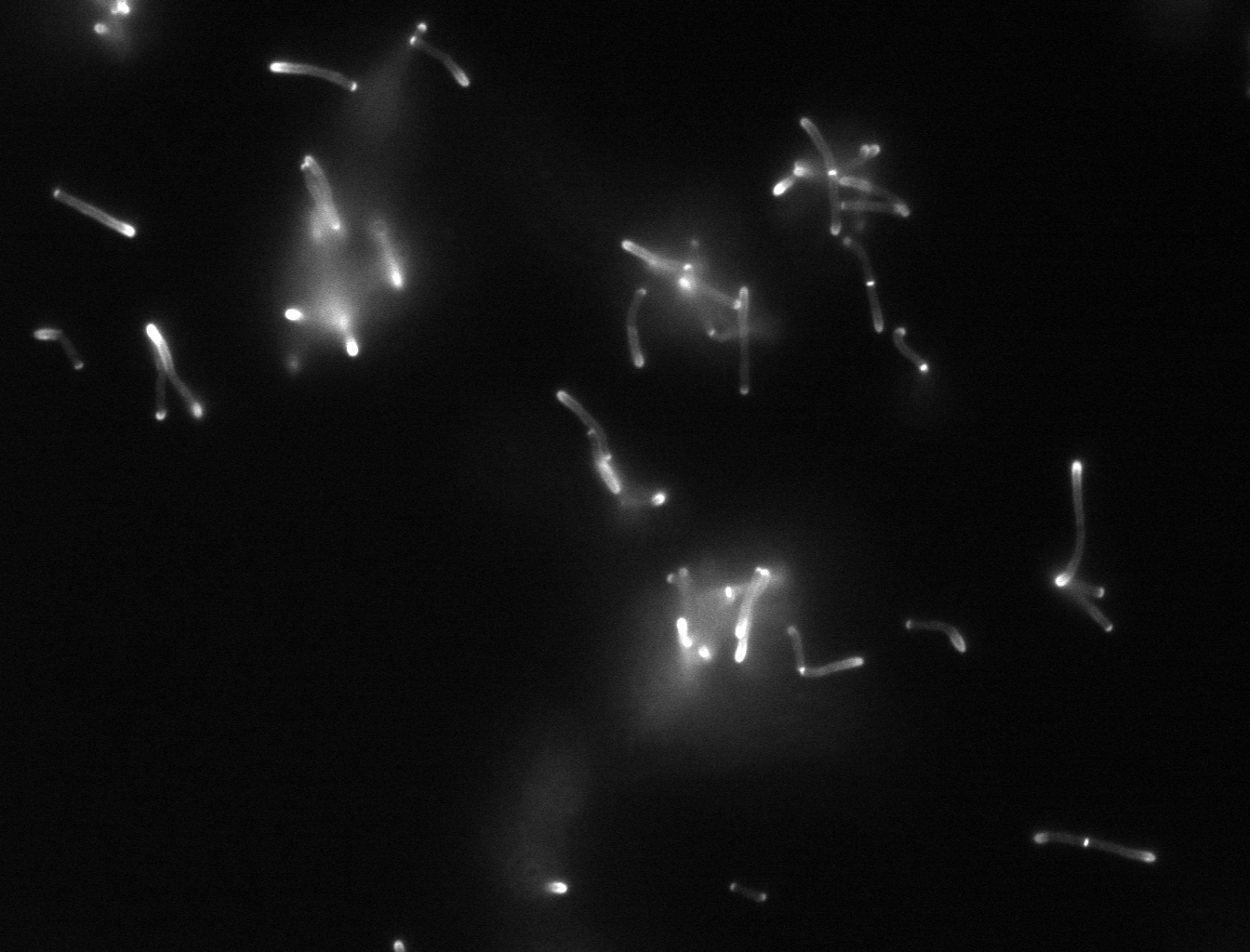

Supplement: Figure 2—source data 1. [file elife-37243-fig2-data1.zip › Figure 2 source data/Figure 2 source data-conventional microscopy (N alk TMM + RADA)/2. NAlkTMM/9.tif]

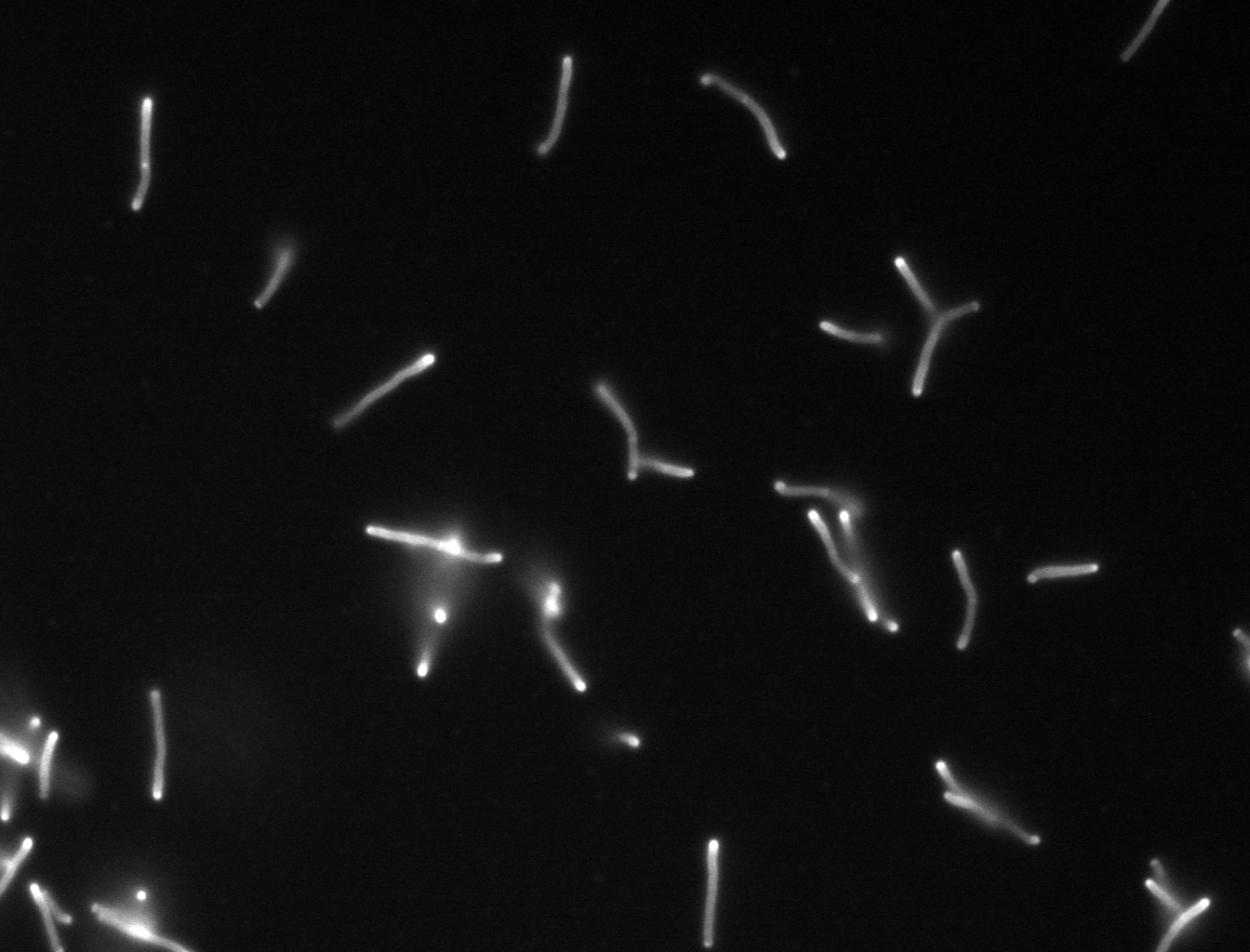

Supplement: Figure 2—source data 1. [file elife-37243-fig2-data1.zip › Figure 2 source data/Figure 2 source data-conventional microscopy (N alk TMM + RADA)/3. RADA/1.tif]

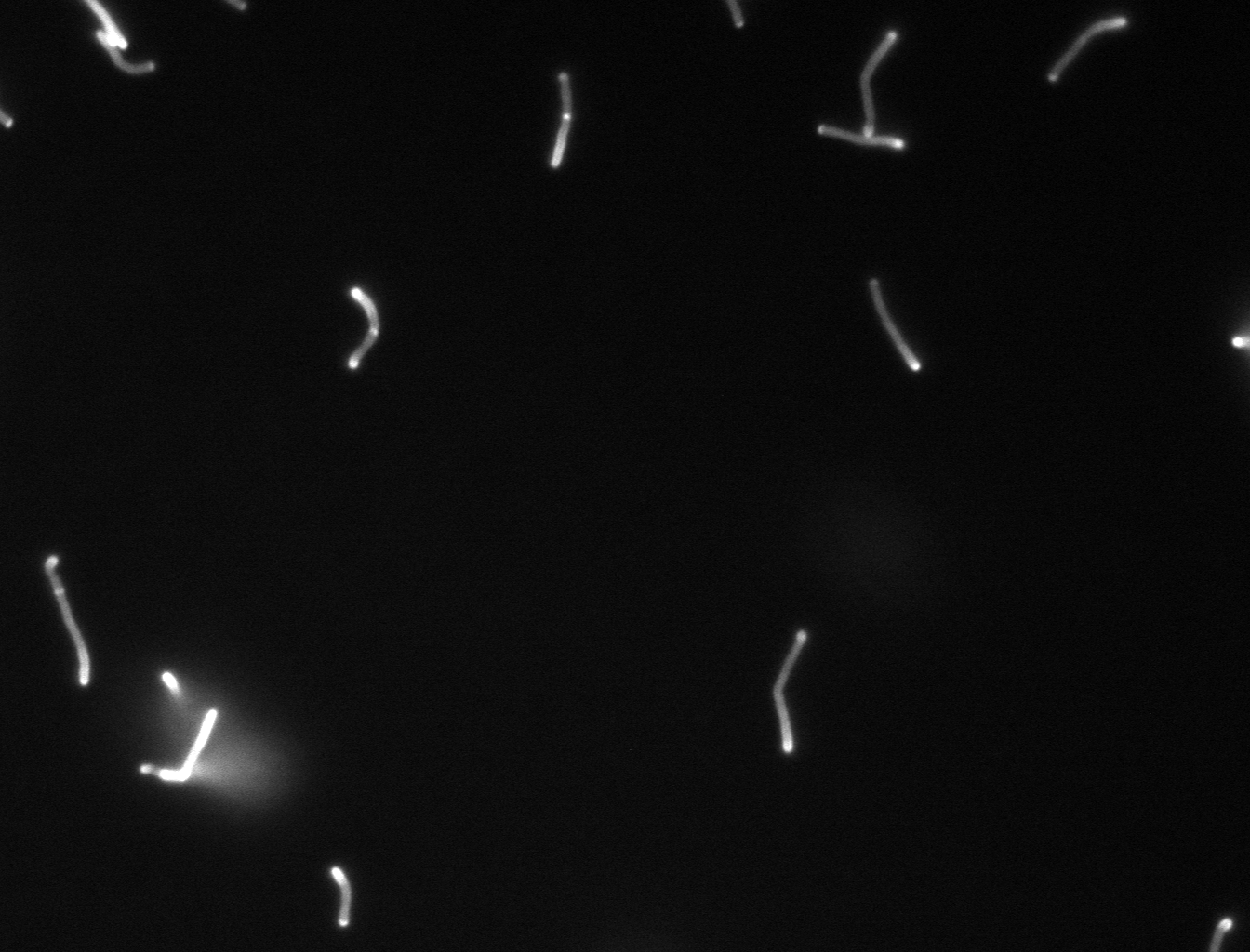

Supplement: Figure 2—source data 1. [file elife-37243-fig2-data1.zip › Figure 2 source data/Figure 2 source data-conventional microscopy (N alk TMM + RADA)/3. RADA/10.tif]

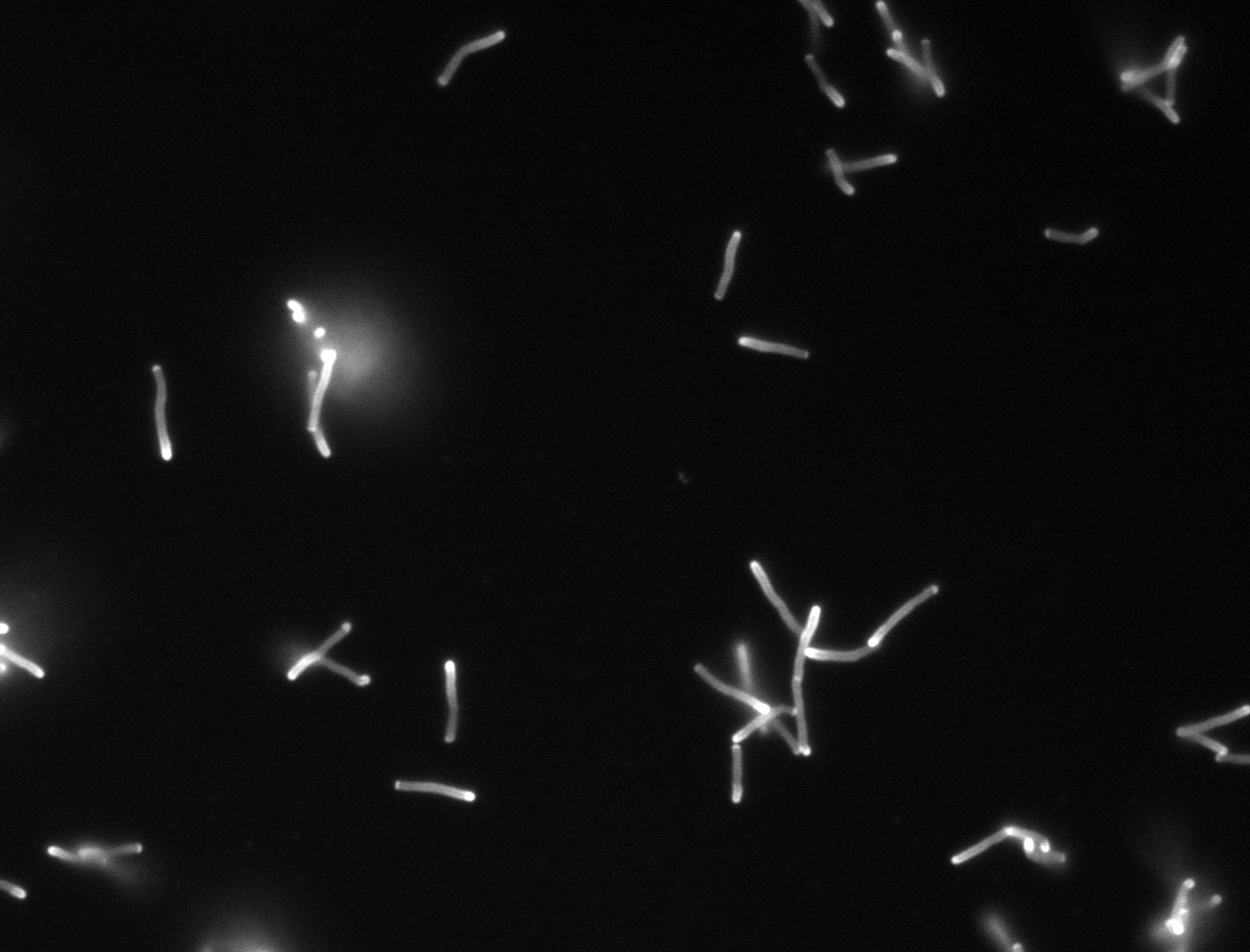

Supplement: Figure 2—source data 1. [file elife-37243-fig2-data1.zip › Figure 2 source data/Figure 2 source data-conventional microscopy (N alk TMM + RADA)/3. RADA/2.tif]

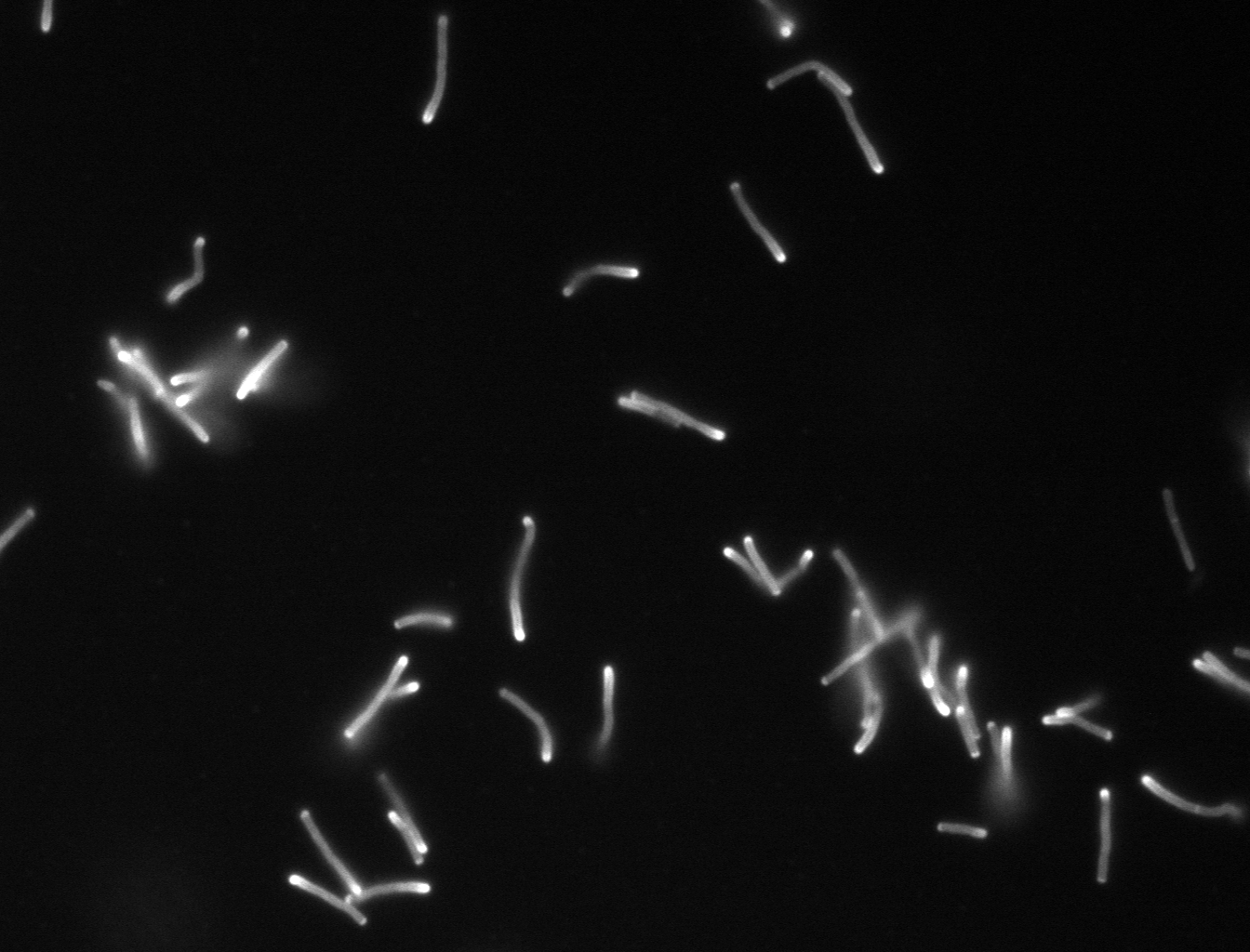

Supplement: Figure 2—source data 1. [file elife-37243-fig2-data1.zip › Figure 2 source data/Figure 2 source data-conventional microscopy (N alk TMM + RADA)/3. RADA/3.tif]

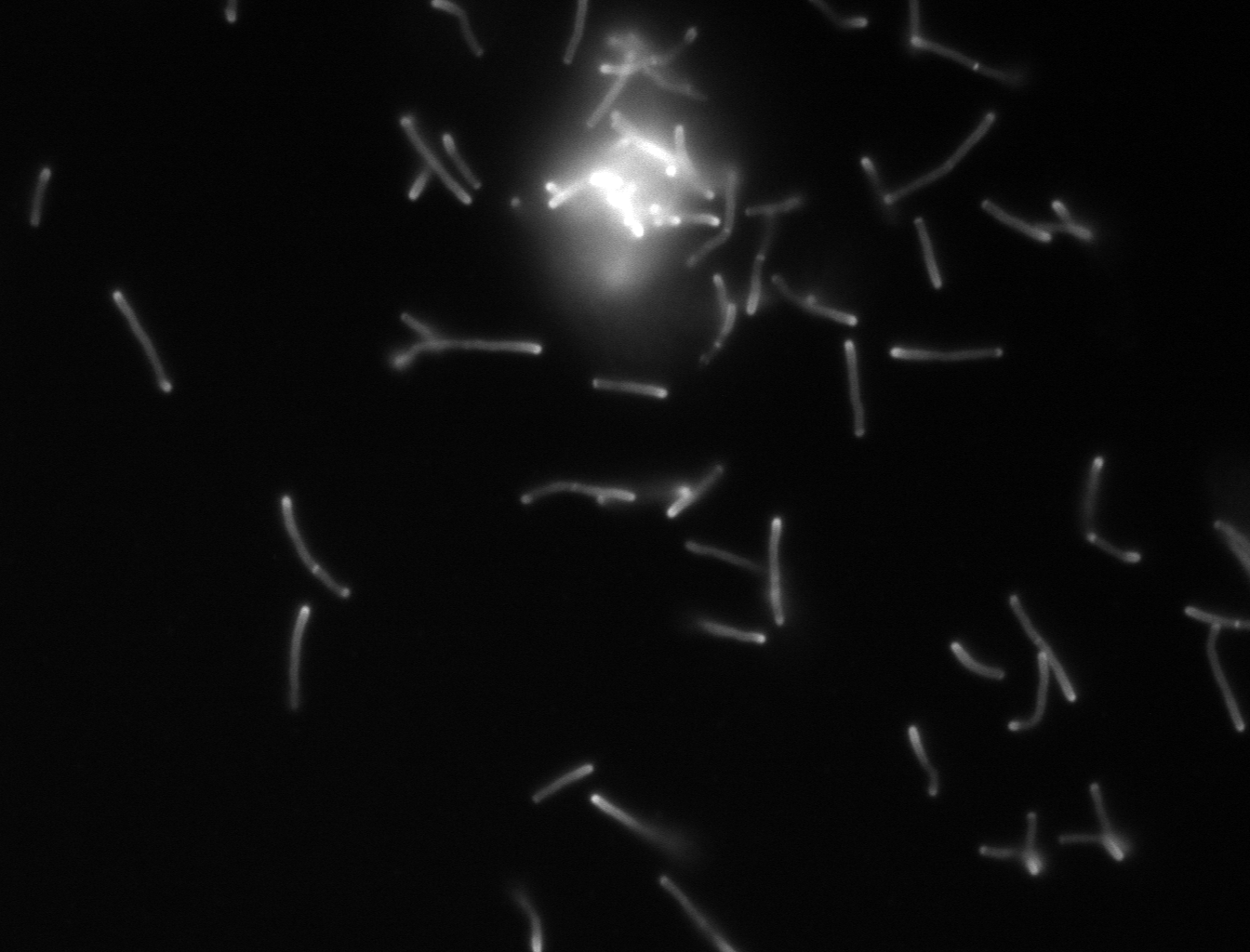

Supplement: Figure 2—source data 1. [file elife-37243-fig2-data1.zip › Figure 2 source data/Figure 2 source data-conventional microscopy (N alk TMM + RADA)/3. RADA/4.tif]

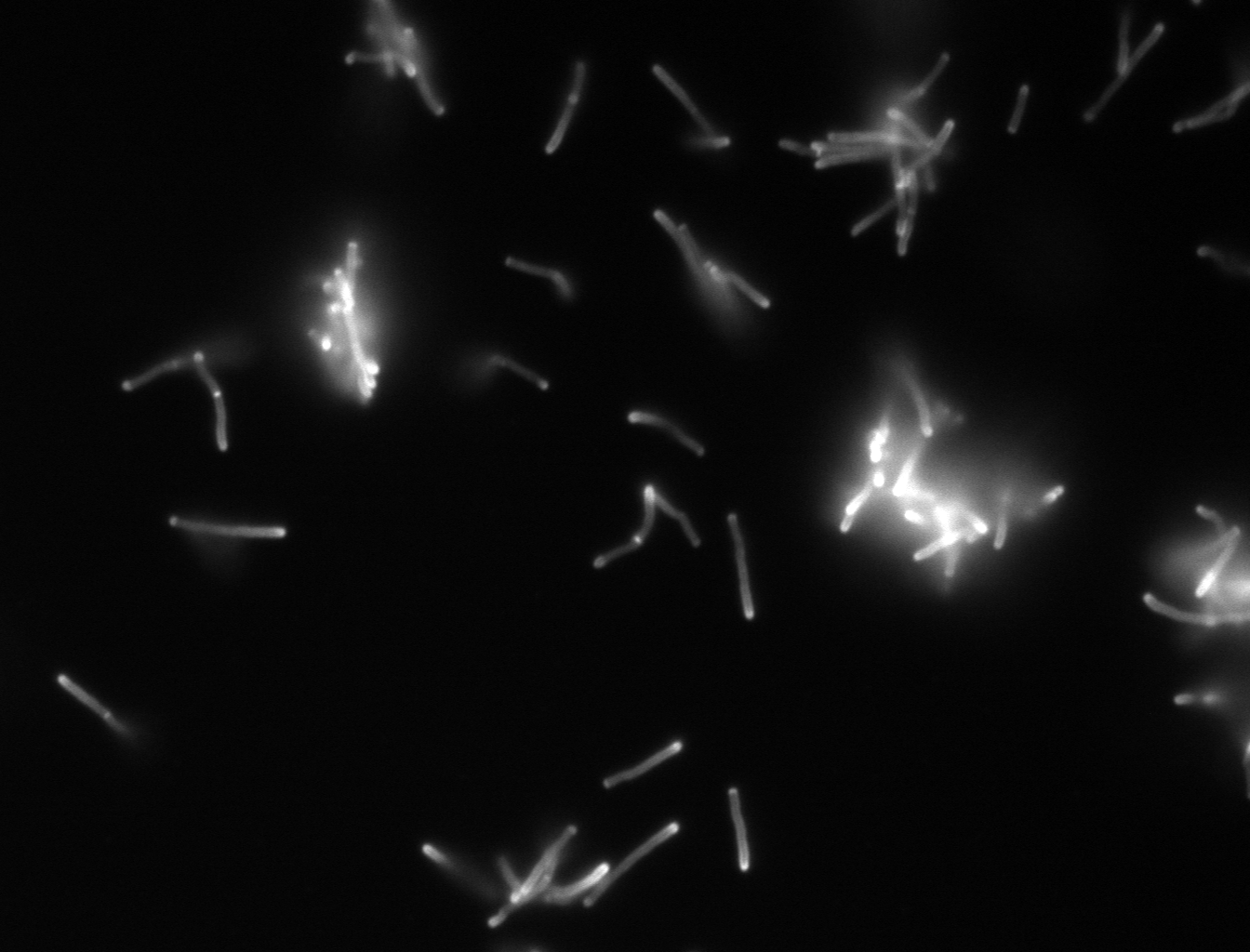

Supplement: Figure 2—source data 1. [file elife-37243-fig2-data1.zip › Figure 2 source data/Figure 2 source data-conventional microscopy (N alk TMM + RADA)/3. RADA/5.tif]

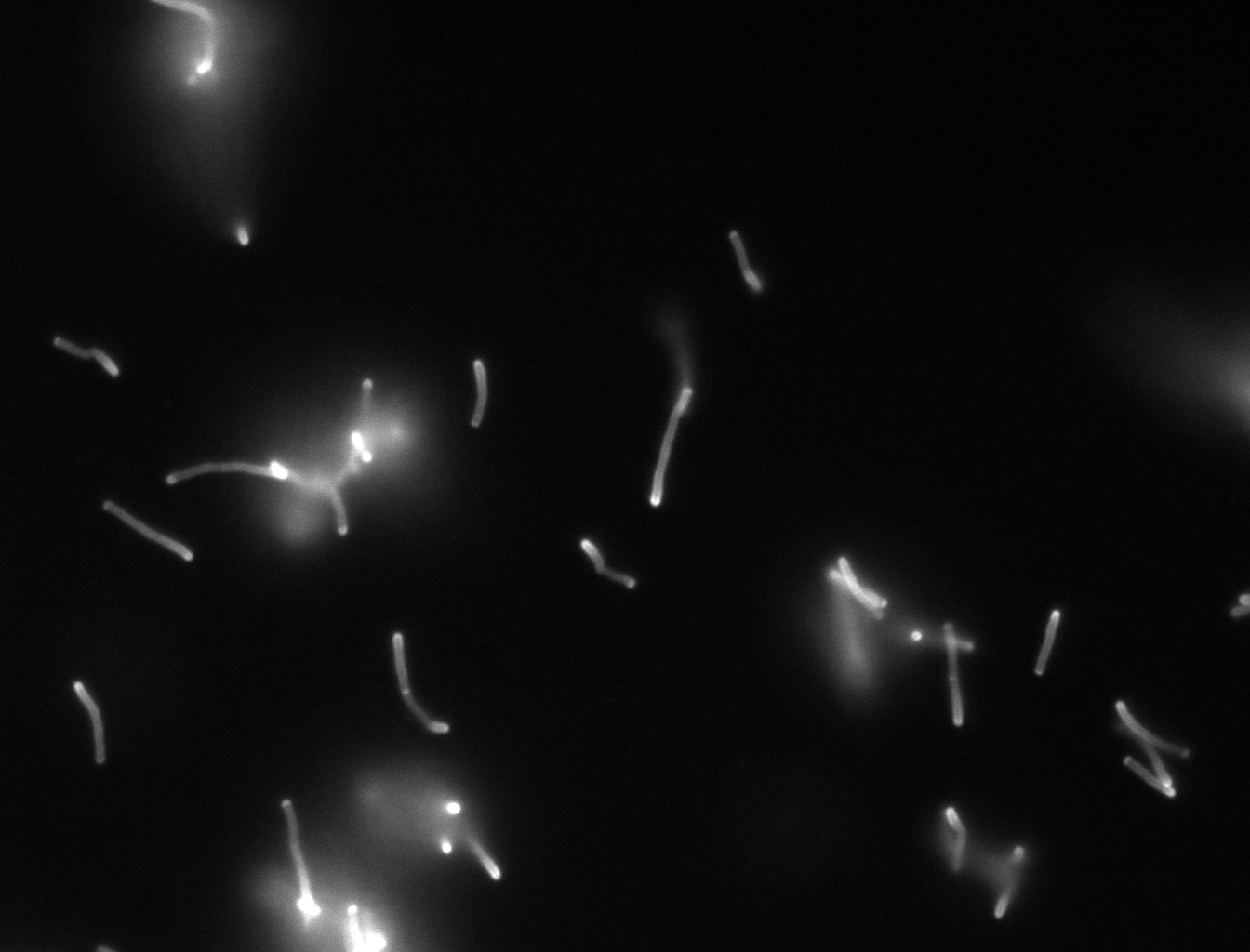

Supplement: Figure 2—source data 1. [file elife-37243-fig2-data1.zip › Figure 2 source data/Figure 2 source data-conventional microscopy (N alk TMM + RADA)/3. RADA/6.tif]

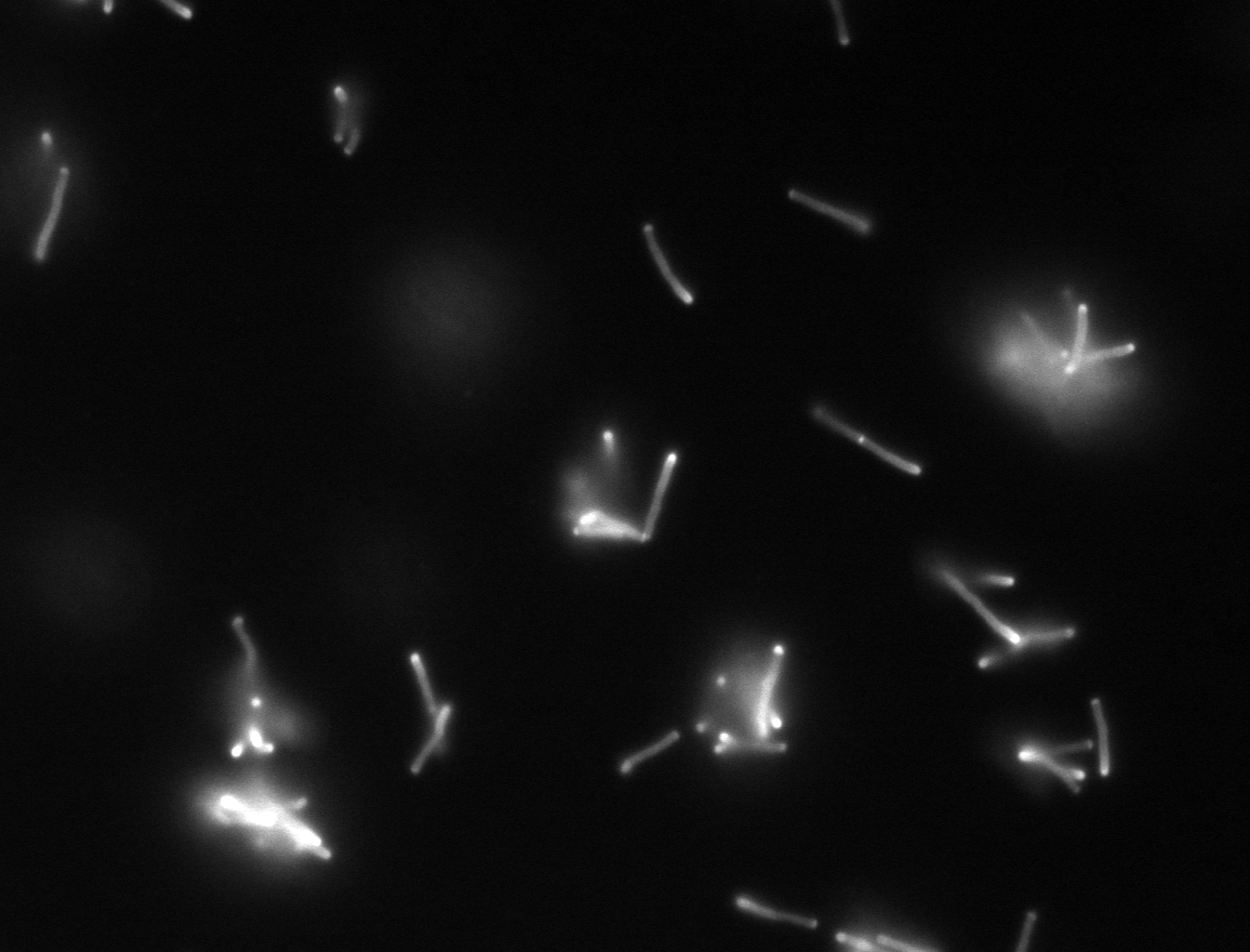

Supplement: Figure 2—source data 1. [file elife-37243-fig2-data1.zip › Figure 2 source data/Figure 2 source data-conventional microscopy (N alk TMM + RADA)/3. RADA/7.tif]

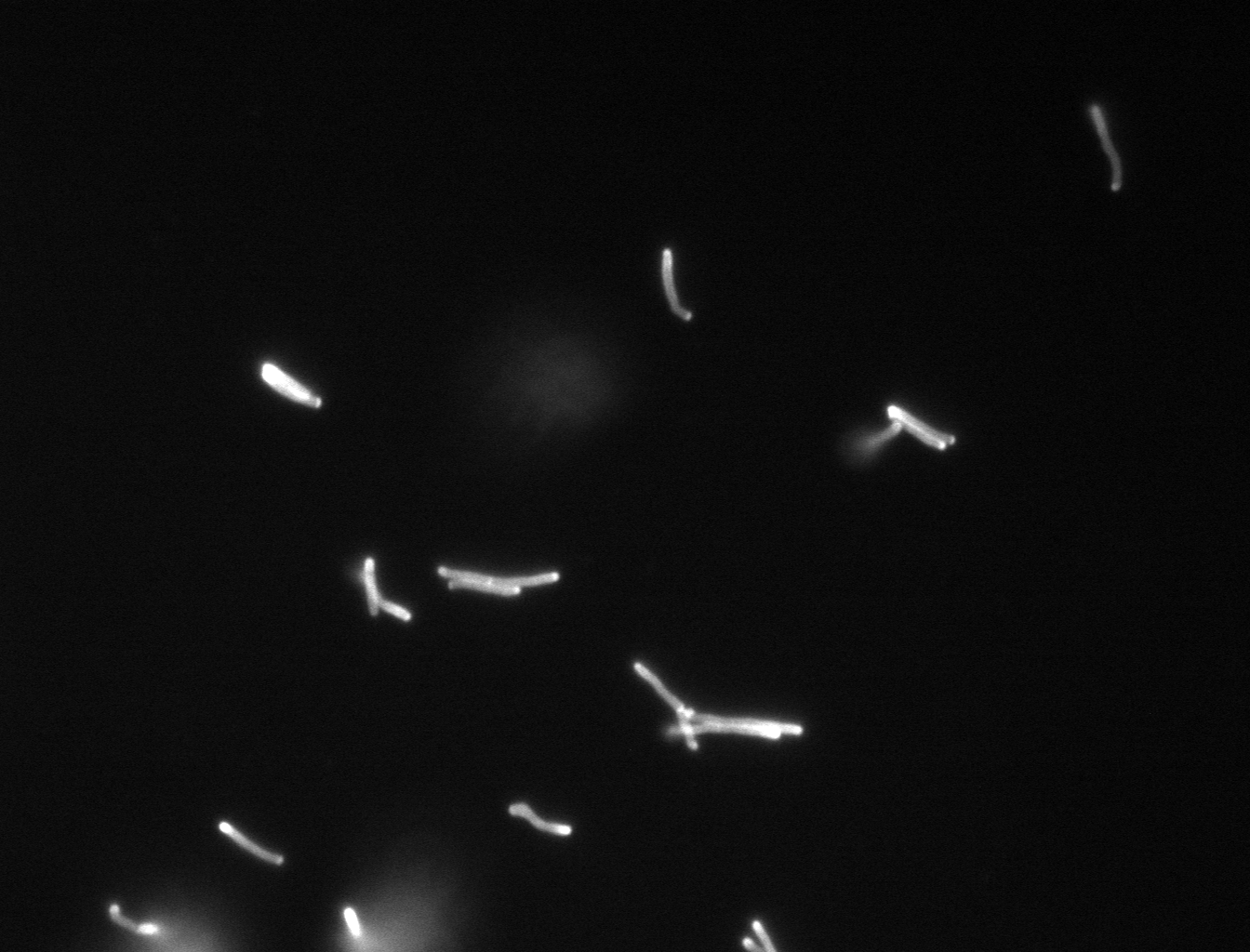

Supplement: Figure 2—source data 1. [file elife-37243-fig2-data1.zip › Figure 2 source data/Figure 2 source data-conventional microscopy (N alk TMM + RADA)/3. RADA/8.tif]

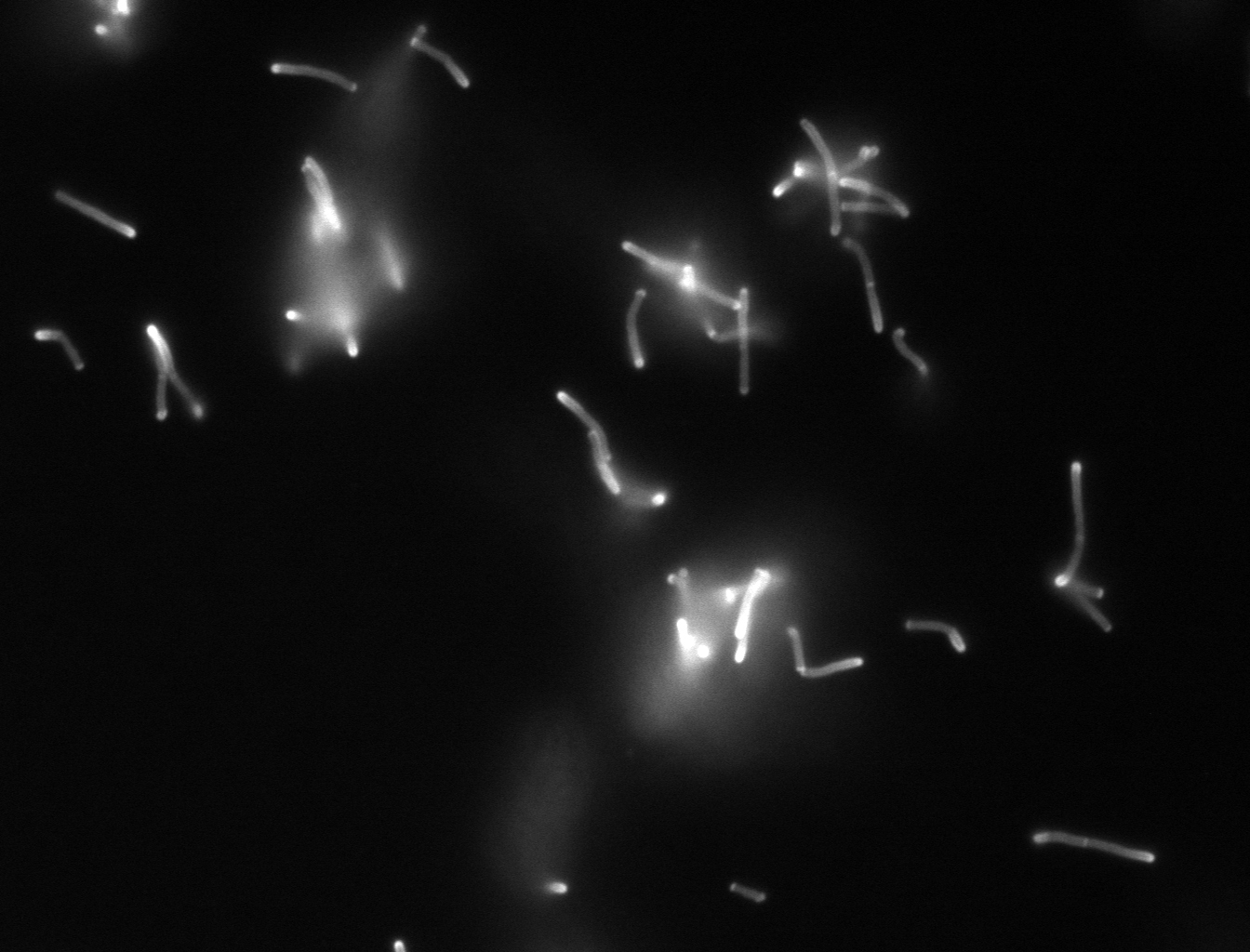

Supplement: Figure 2—source data 1. [file elife-37243-fig2-data1.zip › Figure 2 source data/Figure 2 source data-conventional microscopy (N alk TMM + RADA)/3. RADA/9.tif]

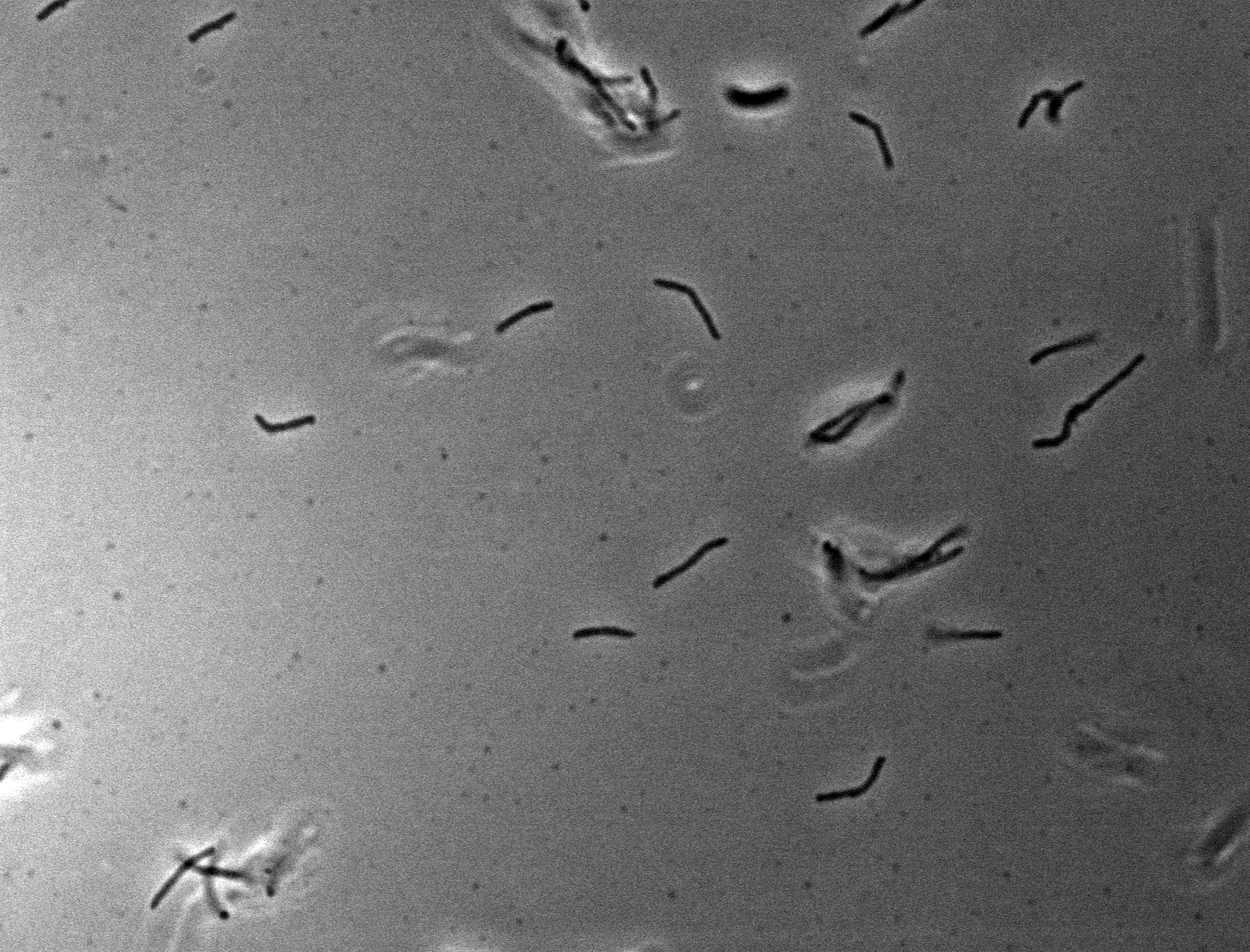

Supplement: Figure 2—source data 1. [file elife-37243-fig2-data1.zip › Figure 2 source data/Figure 2 source data-conventional microscopy (OalkTMM + RADA)/1. Phase/1.tif]

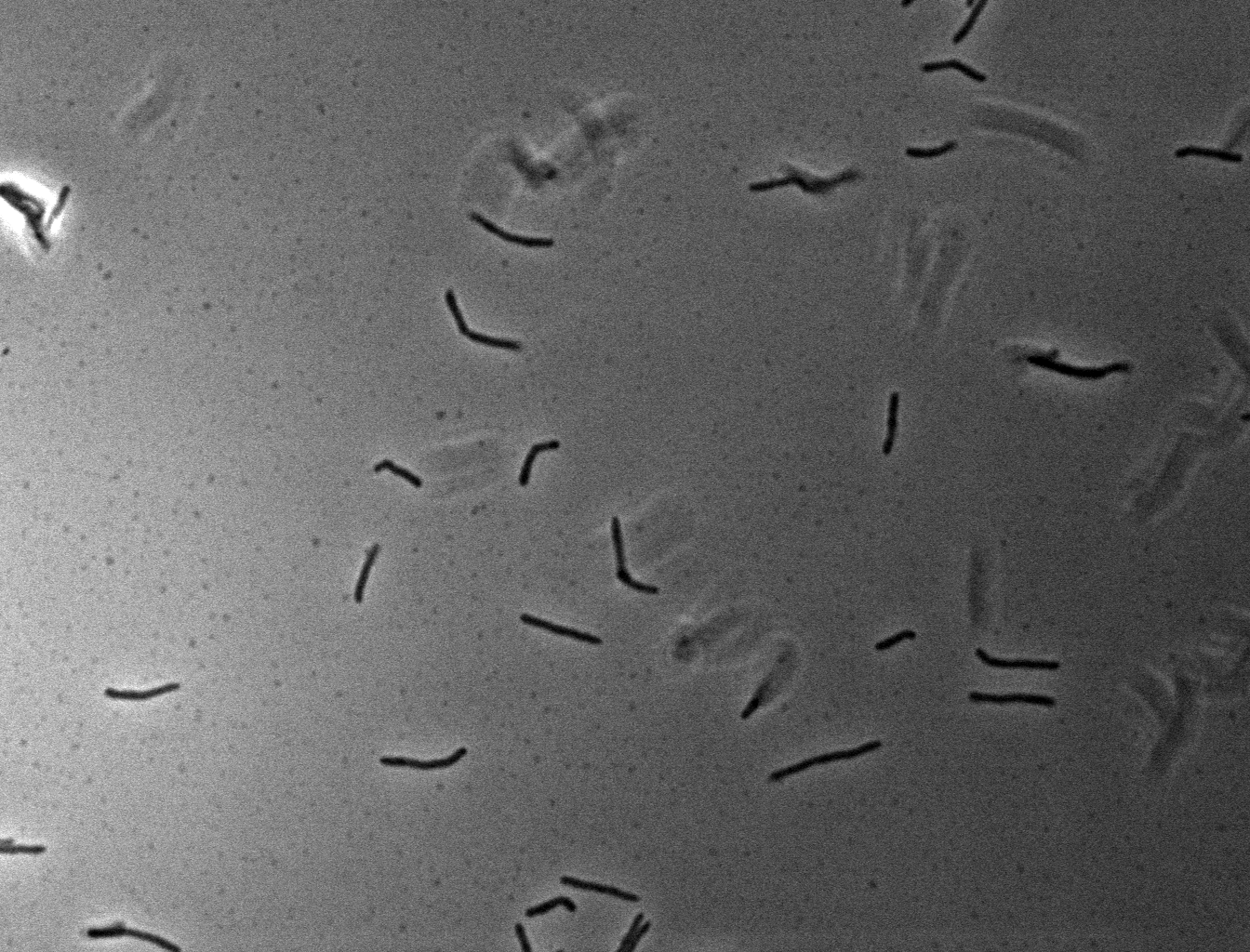

Supplement: Figure 2—source data 1. [file elife-37243-fig2-data1.zip › Figure 2 source data/Figure 2 source data-conventional microscopy (OalkTMM + RADA)/1. Phase/10.tif]

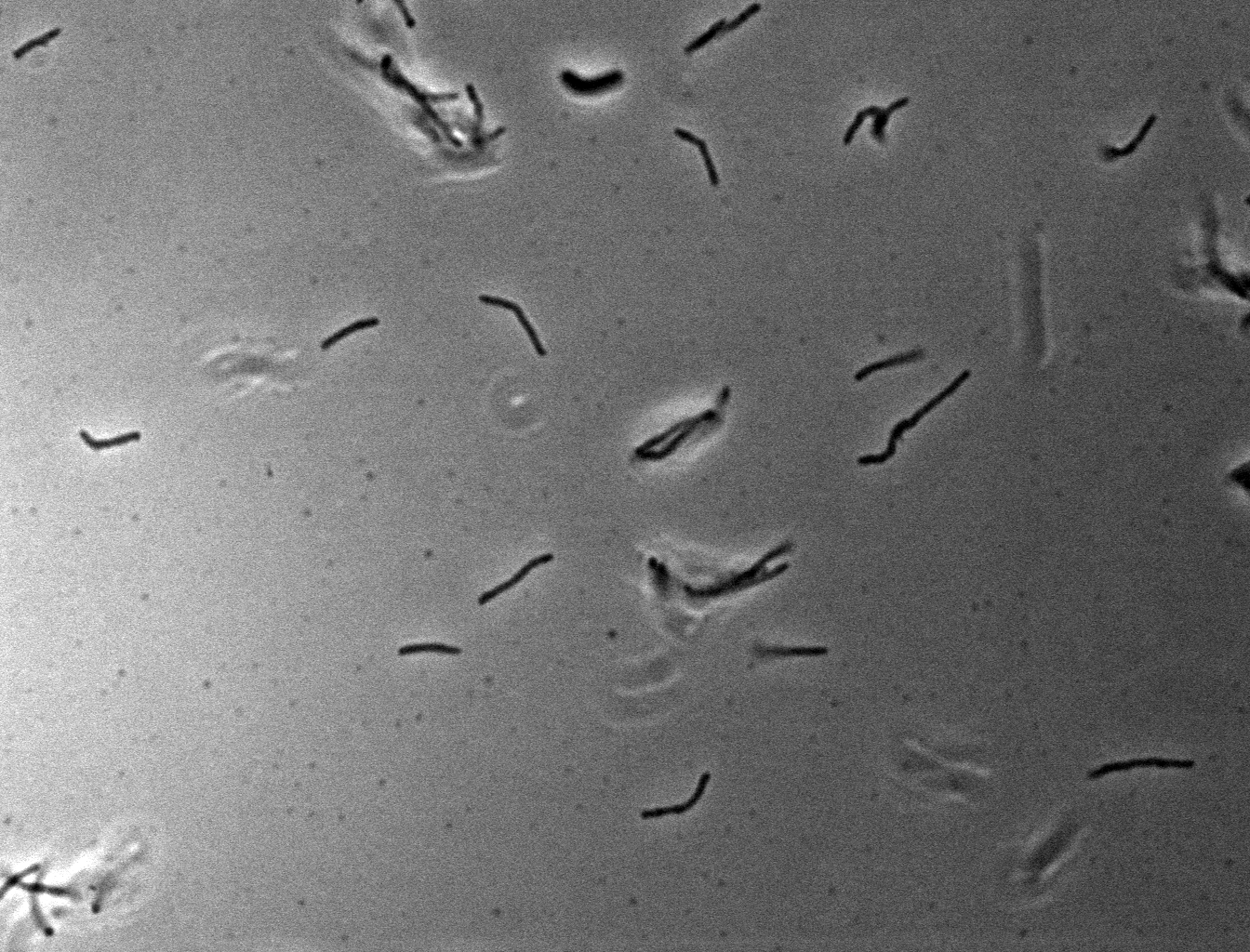

Supplement: Figure 2—source data 1. [file elife-37243-fig2-data1.zip › Figure 2 source data/Figure 2 source data-conventional microscopy (OalkTMM + RADA)/1. Phase/2.tif]

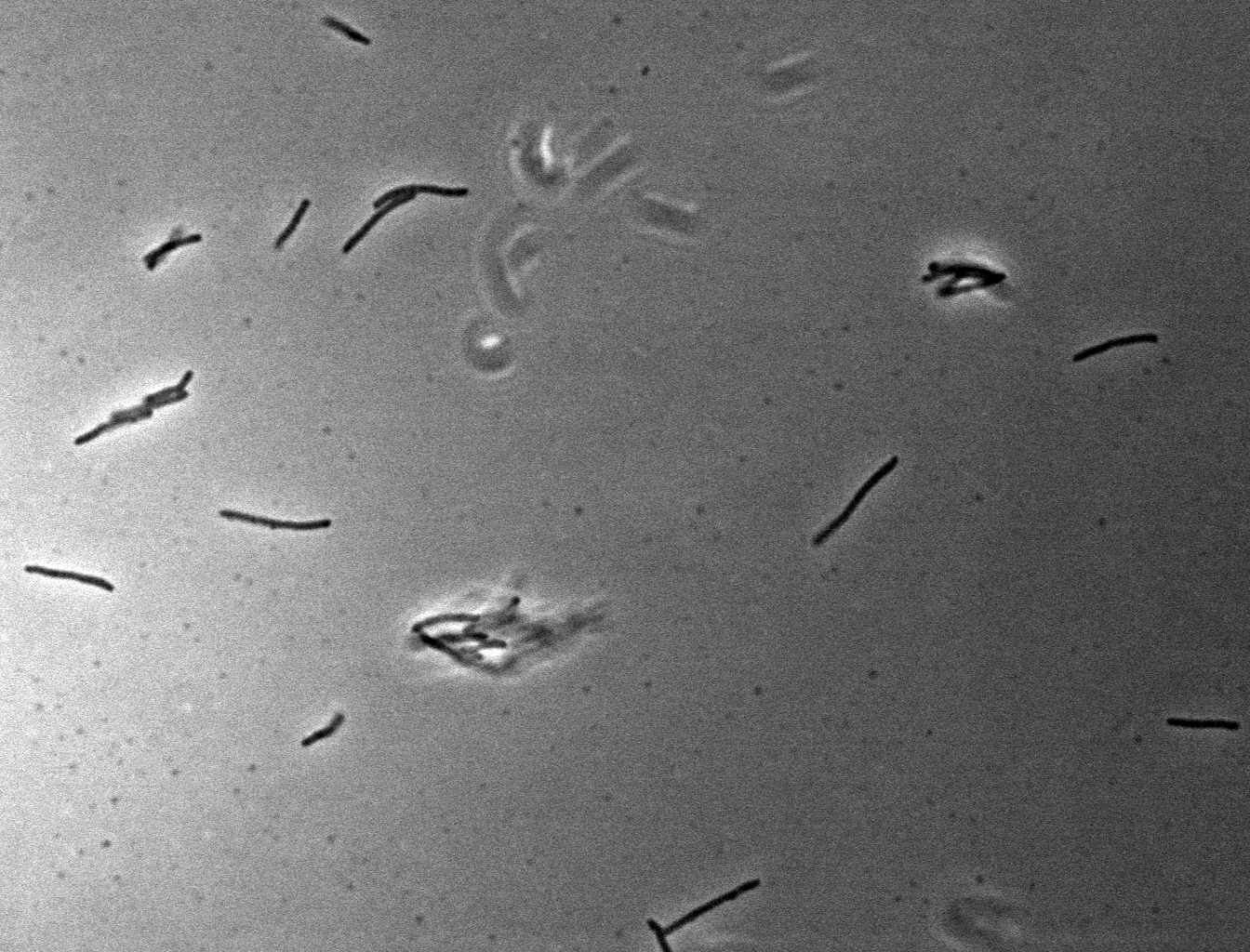

Supplement: Figure 2—source data 1. [file elife-37243-fig2-data1.zip › Figure 2 source data/Figure 2 source data-conventional microscopy (OalkTMM + RADA)/1. Phase/3.tif]

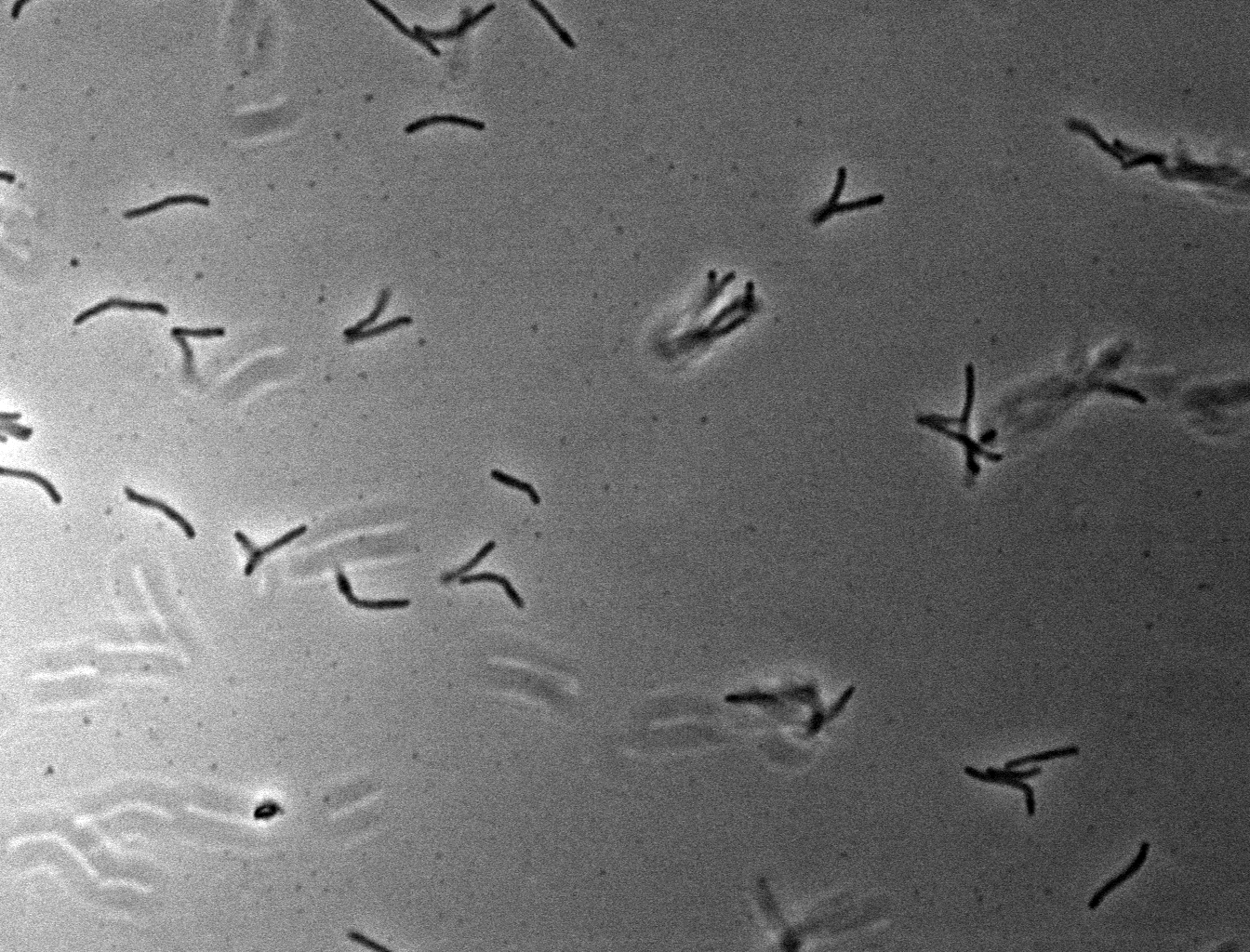

Supplement: Figure 2—source data 1. [file elife-37243-fig2-data1.zip › Figure 2 source data/Figure 2 source data-conventional microscopy (OalkTMM + RADA)/1. Phase/4.tif]

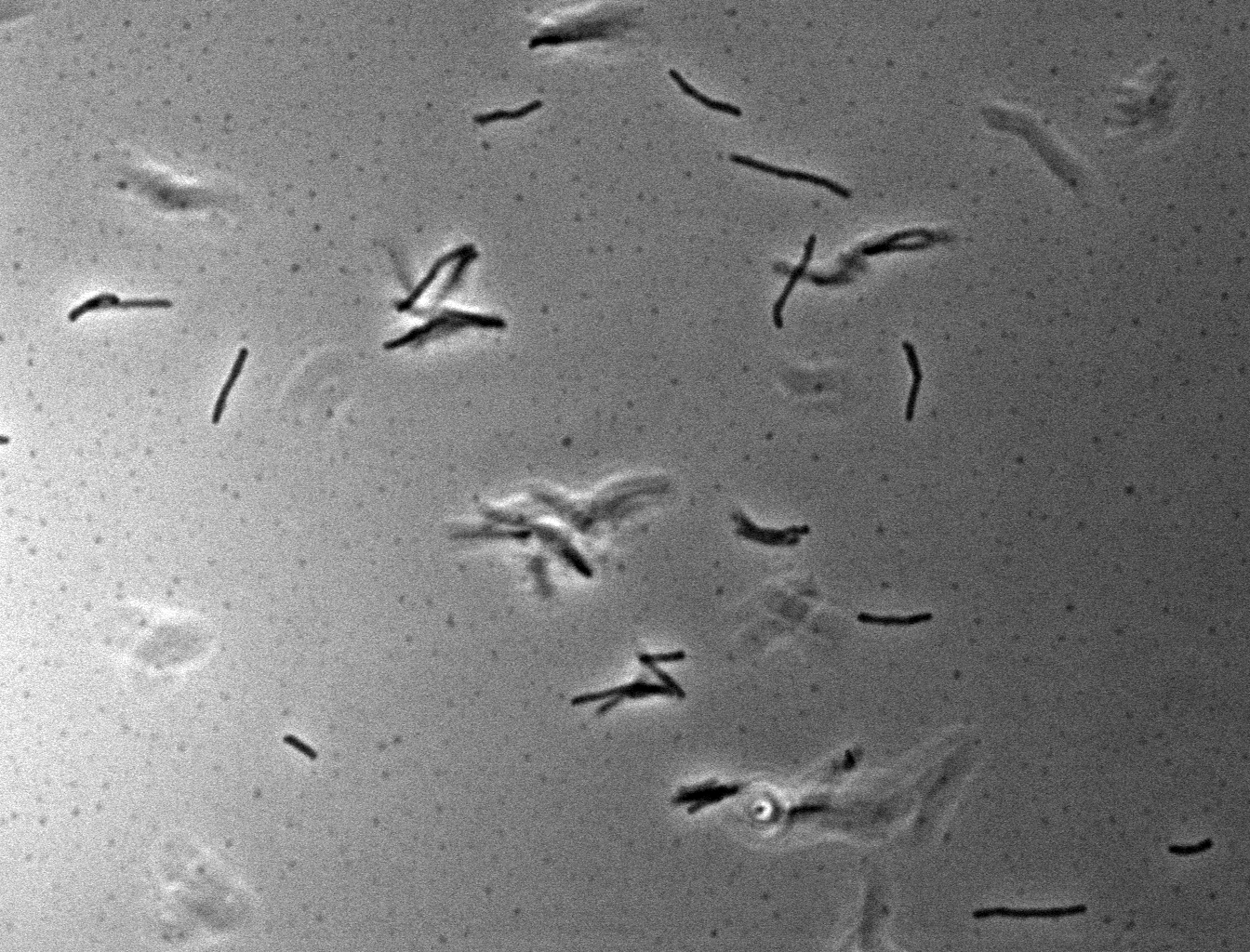

Supplement: Figure 2—source data 1. [file elife-37243-fig2-data1.zip › Figure 2 source data/Figure 2 source data-conventional microscopy (OalkTMM + RADA)/1. Phase/5.tif]

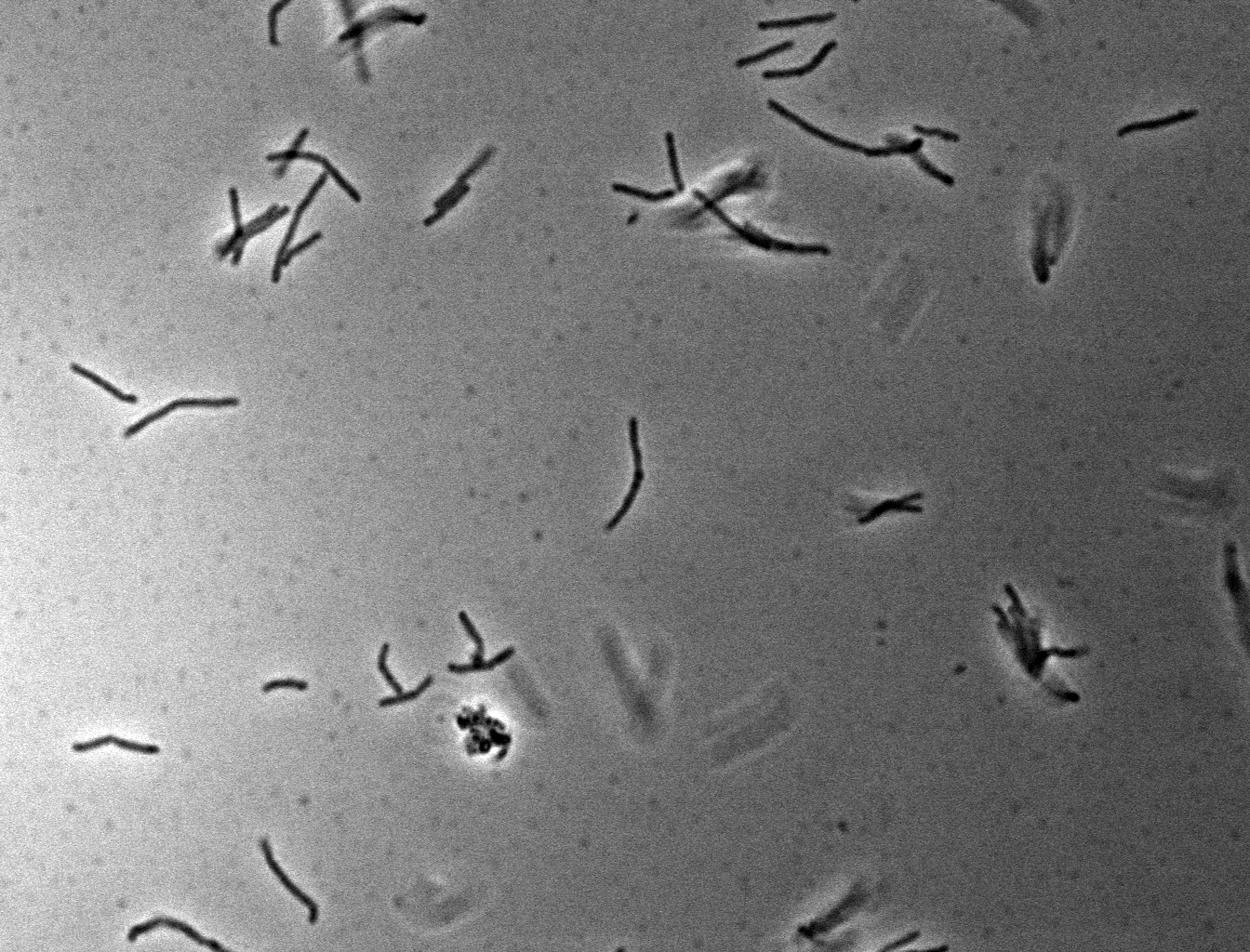

Supplement: Figure 2—source data 1. [file elife-37243-fig2-data1.zip › Figure 2 source data/Figure 2 source data-conventional microscopy (OalkTMM + RADA)/1. Phase/6.tif]

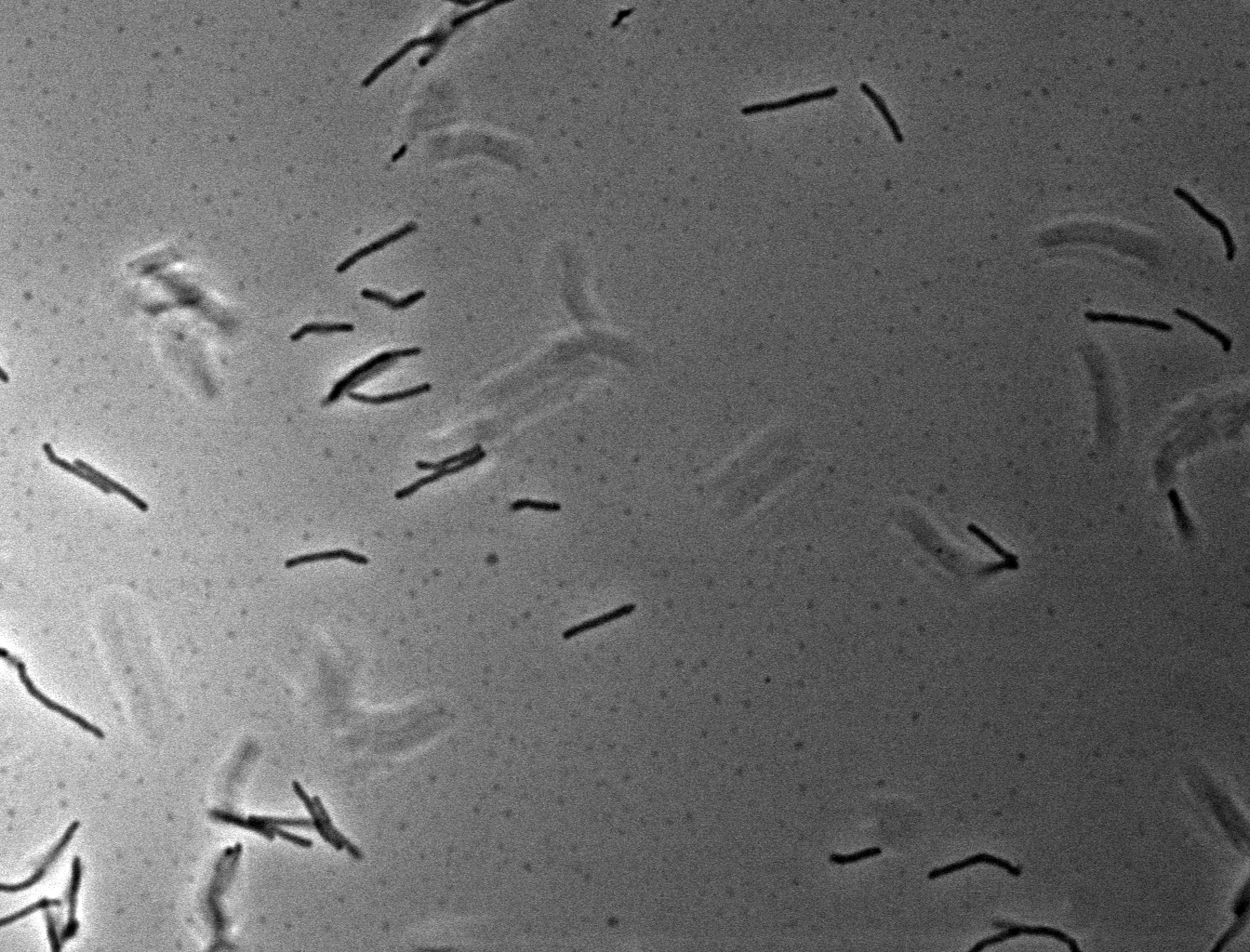

Supplement: Figure 2—source data 1. [file elife-37243-fig2-data1.zip › Figure 2 source data/Figure 2 source data-conventional microscopy (OalkTMM + RADA)/1. Phase/7.tif]

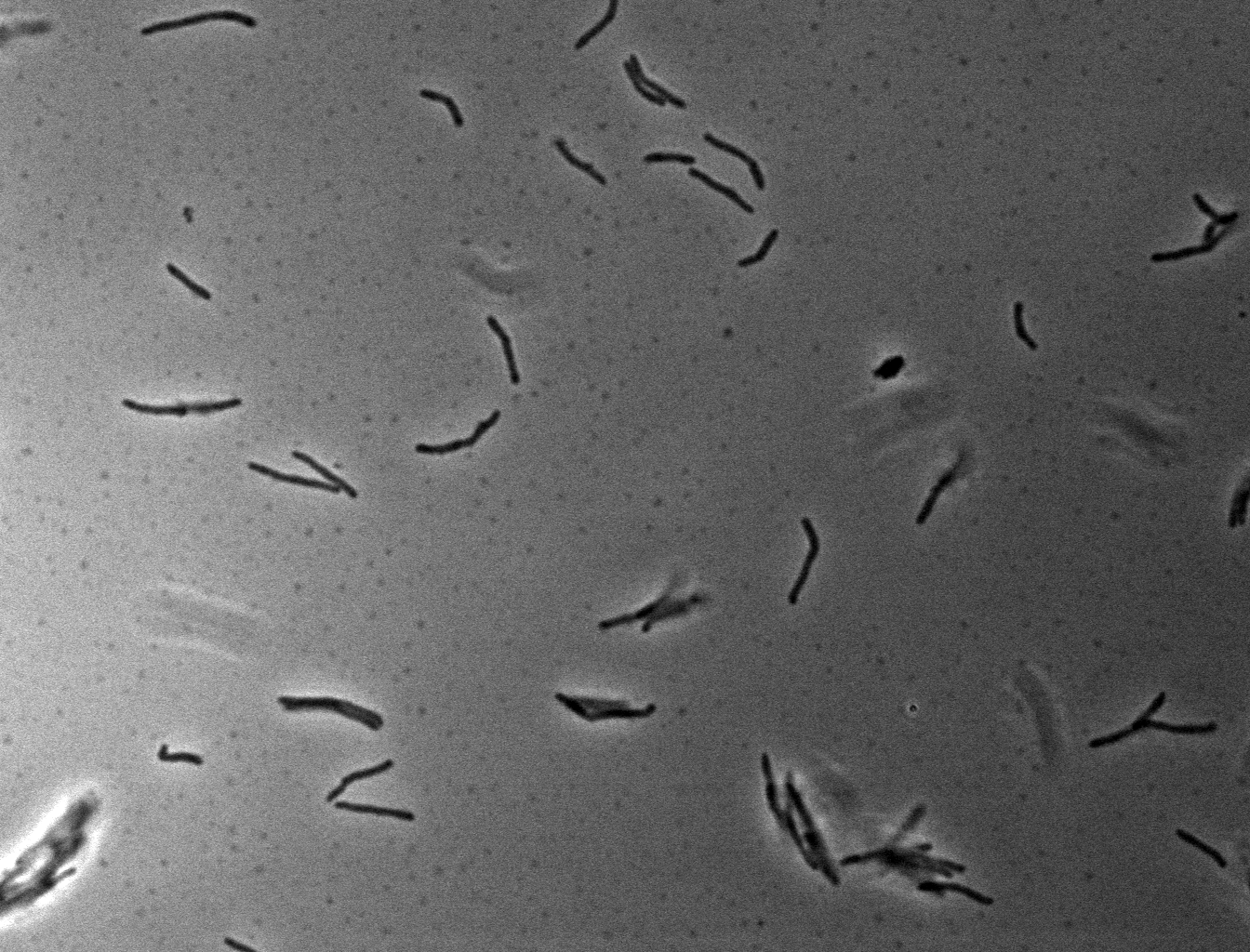

Supplement: Figure 2—source data 1. [file elife-37243-fig2-data1.zip › Figure 2 source data/Figure 2 source data-conventional microscopy (OalkTMM + RADA)/1. Phase/8.tif]
