# Supplementary material for: Peptidoglycan precursor synthesis along the sidewall of pole-growing mycobacteria
Source: eLife. 2018 Sep 10;7:e37243. doi: 10.7554/eLife.37243 (PMC6191288; doi:10.7554/eLife.37243)
Supplement: Figure 2—figure supplement 2—source data 1. [file elife-37243-fig2-figsupp2-data1.zip › Figure 2--figure supplement 2/Figure 2--figure supplement 2A.pdf]

BD FACSDiva 8.0

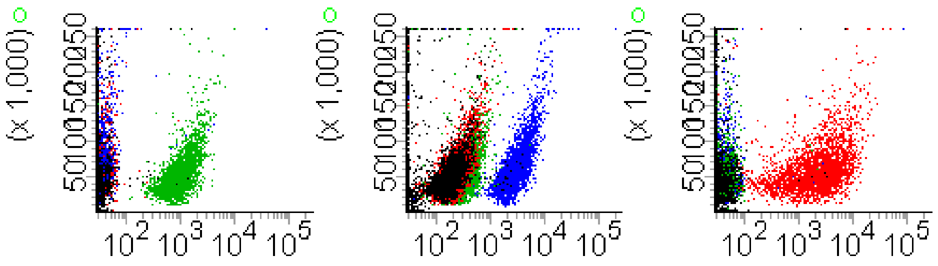

Tube: 7H9 -/-

Population	#Events	%Parent	%Total
All Events	10,996	####	100.0
P1	5,967	54.3	54.3
Specimen_001/7H9 R/R/All Events	10,000	####	100.0
Specimen_001/7H9 R/R/P1	9,377	93.8	93.8
Specimen_001/7H9 H/H/All Events	10,000	####	100.0
Specimen_001/7H9 H/H/P1	9,824	98.2	98.2
Specimen_001/7H9 N/N/All Events	10,000	####	100.0
Specimen_001/7H9 N/N/P1	9,471	94.7	94.7
