# Supplementary material for: Peptidoglycan precursor synthesis along the sidewall of pole-growing mycobacteria
Source: eLife. 2018 Sep 10;7:e37243. doi: 10.7554/eLife.37243 (PMC6191288; doi:10.7554/eLife.37243)
Supplement: Figure 2—figure supplement 2—source data 1. [file elife-37243-fig2-figsupp2-data1.zip › Figure 2--figure supplement 2/Figure 2--figure supplement 2B.pdf]

BD FACSDiva 8.0

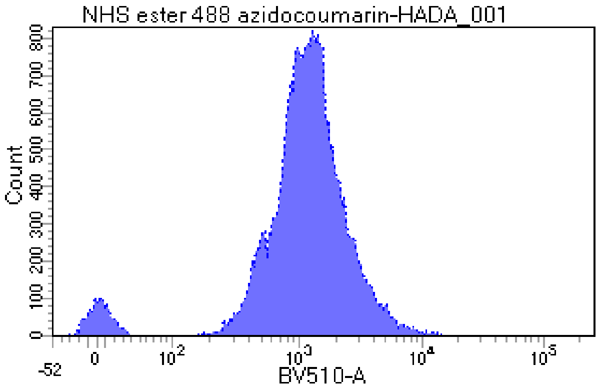

Tube: HADA_001

Population	#Events	%Parent	%Total
All Events	30,000	####	100.0
P1	29,141	97.1	97.1

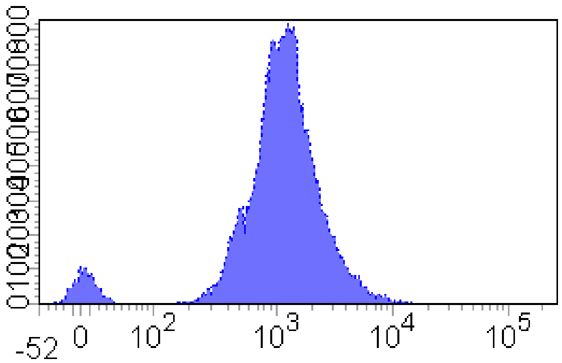

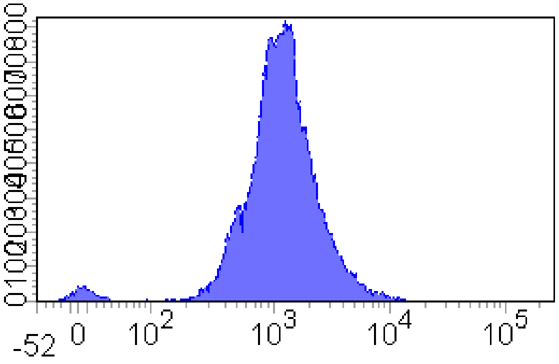

Sheet1

Printed on: Wed Dec 21, 2016 03:11:05 EST

BD FACSDiva 8.0

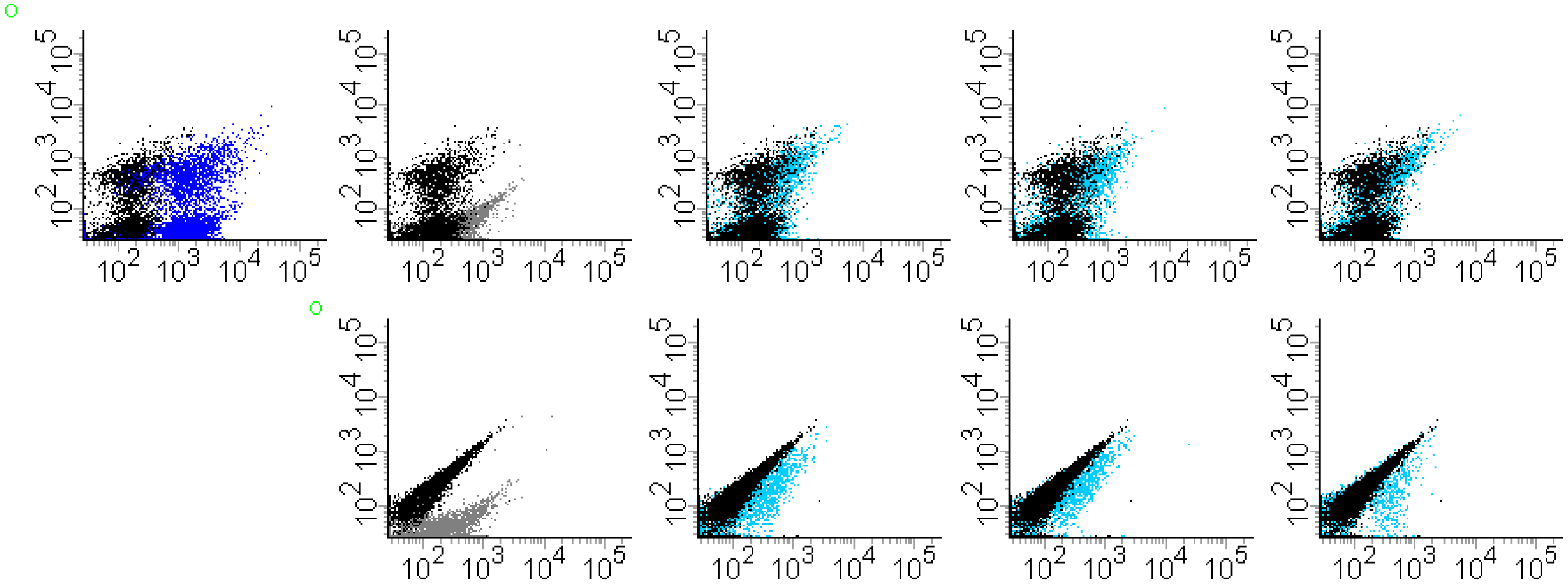

Tube: OalkTMM

Population	#Events	%Parent	%Total
NHS488/OalkTMM	30,000	####	100.0
P1	28,882	96.3	96.3
NHS ester 488 azidocoumarin/no p	30,000	####	100.0
NHS ester 488 azidocoumarin/r	27,947	93.2	93.2
NHS ester 488 azidocoumarin/HAD	30,000	####	100.0
NHS ester 488 azidocoumarin/H	29,141	97.1	97.1
NHS ester 488 azidocoumarin/alkD	30,000	####	100.0
NHS ester 488 azidocoumarin/a	28,220	94.1	94.1
NHS ester 488 azidocoumarin/alkD	30,000	####	100.0
NHS ester 488 azidocoumarin/a	28,476	94.9	94.9
no NADA no probe	30,000	####	100.0
FDAA/CuAAC/no FDAA no probe	28,566	95.2	95.2
NHS ester 488 azidocoumarin/Nalk	30,000	####	100.0
NHS ester 488 azidocoumarin/N	29,247	97.5	97.5

Tube: NADA no probe azidocoumarin

Population	#Events	%Parent	%Total
NADA no probe	30,000	####	100.0
P1	28,996	96.7	96.7
NADA alkD	30,000	####	100.0
FDAA/CuAAC/NADA alkDala azic	28,951	96.5	96.5
no NADA no probe	30,000	####	100.0
FDAA/CuAAC/no FDAA no probe	28,566	95.2	95.2
NADA alkDD	30,000	####	100.0
NADA OalkTMM	30,000	####	100.0
