# Supplementary figures and images for: Peptidoglycan precursor synthesis along the sidewall of pole-growing mycobacteria (part 3 of 3)
Source: eLife. 2018 Sep 10;7:e37243. doi: 10.7554/eLife.37243 (PMC6191288; doi:10.7554/eLife.37243)

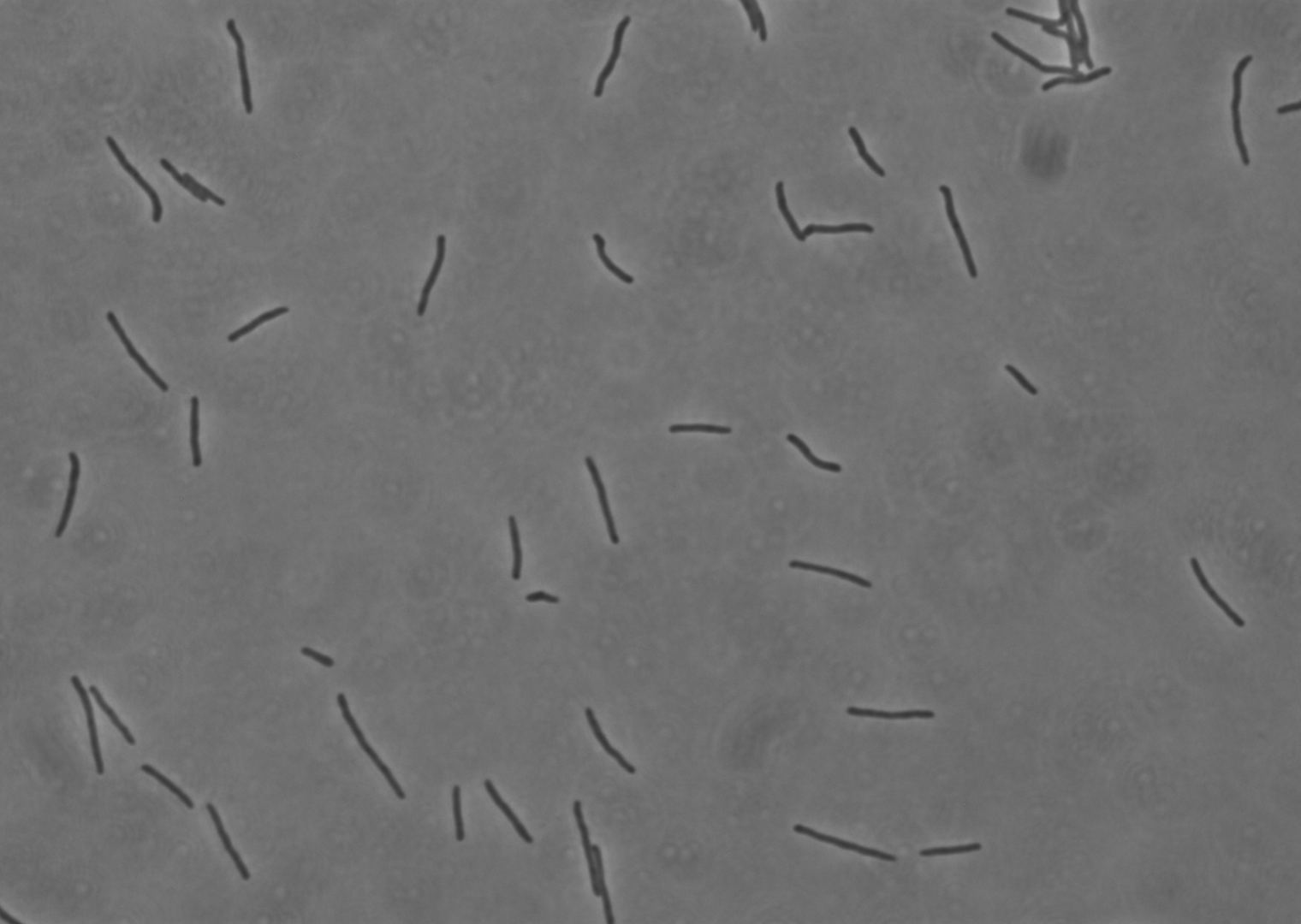

Supplement: Figure 5—figure supplement 3—source data 1. [file elife-37243-fig5-figsupp3-data1.zip › Figure 5-figure supplement 3 source data/2. Bocillin labeling- conventional microscopy /4. DCS treated/1. PC/10.tif]

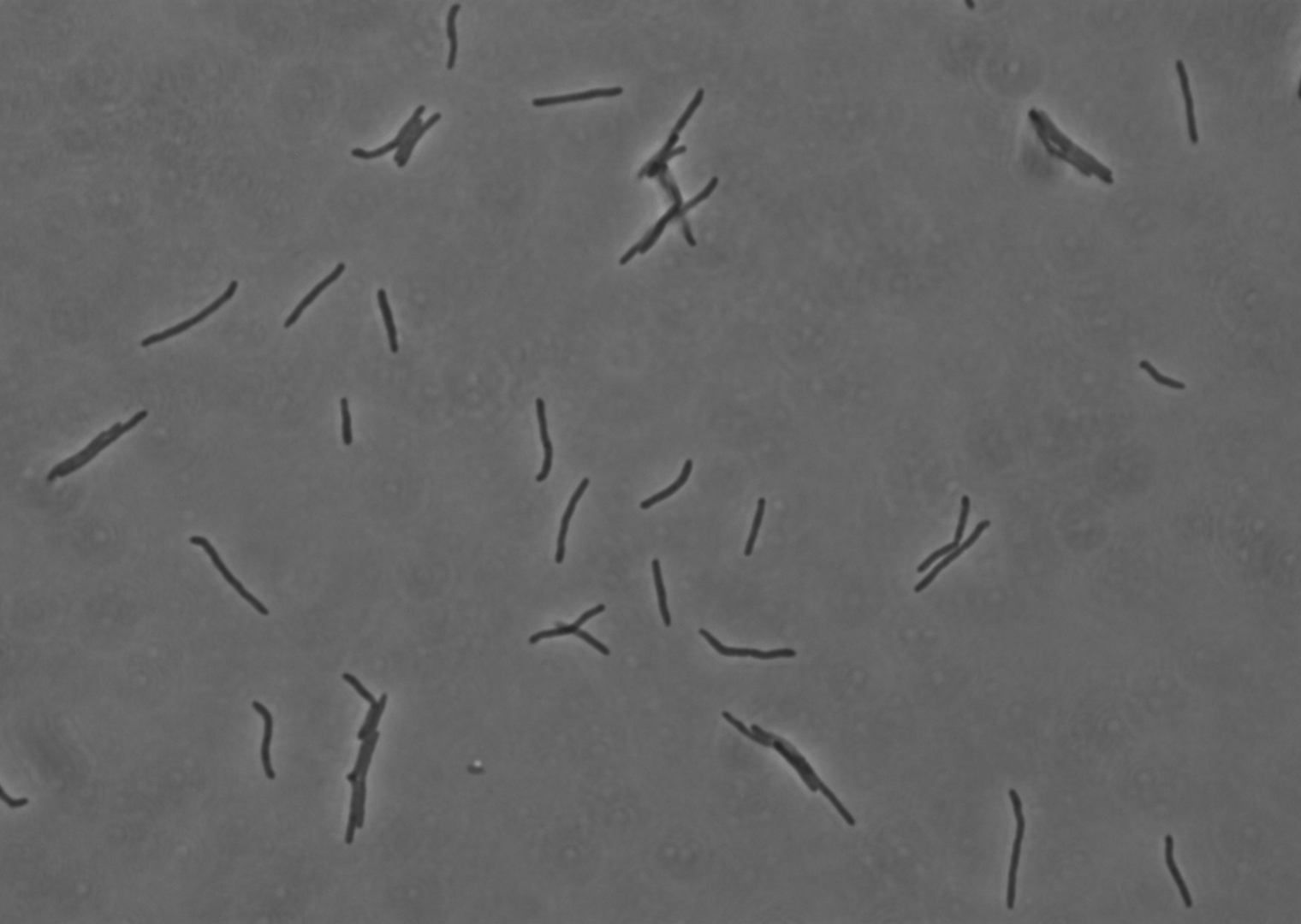

Supplement: Figure 5—figure supplement 3—source data 1. [file elife-37243-fig5-figsupp3-data1.zip › Figure 5-figure supplement 3 source data/2. Bocillin labeling- conventional microscopy /4. DCS treated/1. PC/11.tif]

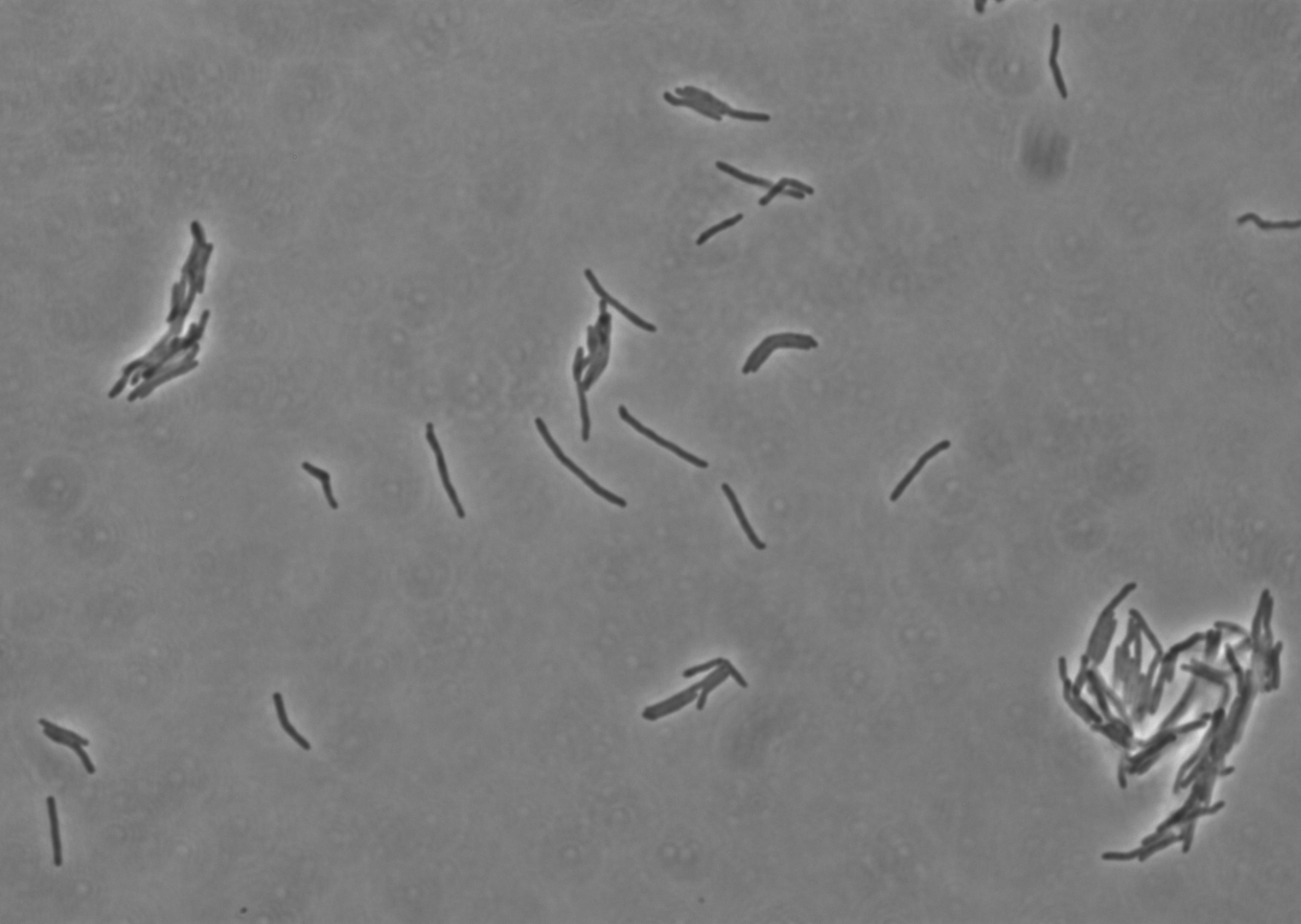

Supplement: Figure 5—figure supplement 3—source data 1. [file elife-37243-fig5-figsupp3-data1.zip › Figure 5-figure supplement 3 source data/2. Bocillin labeling- conventional microscopy /4. DCS treated/1. PC/2.tif]

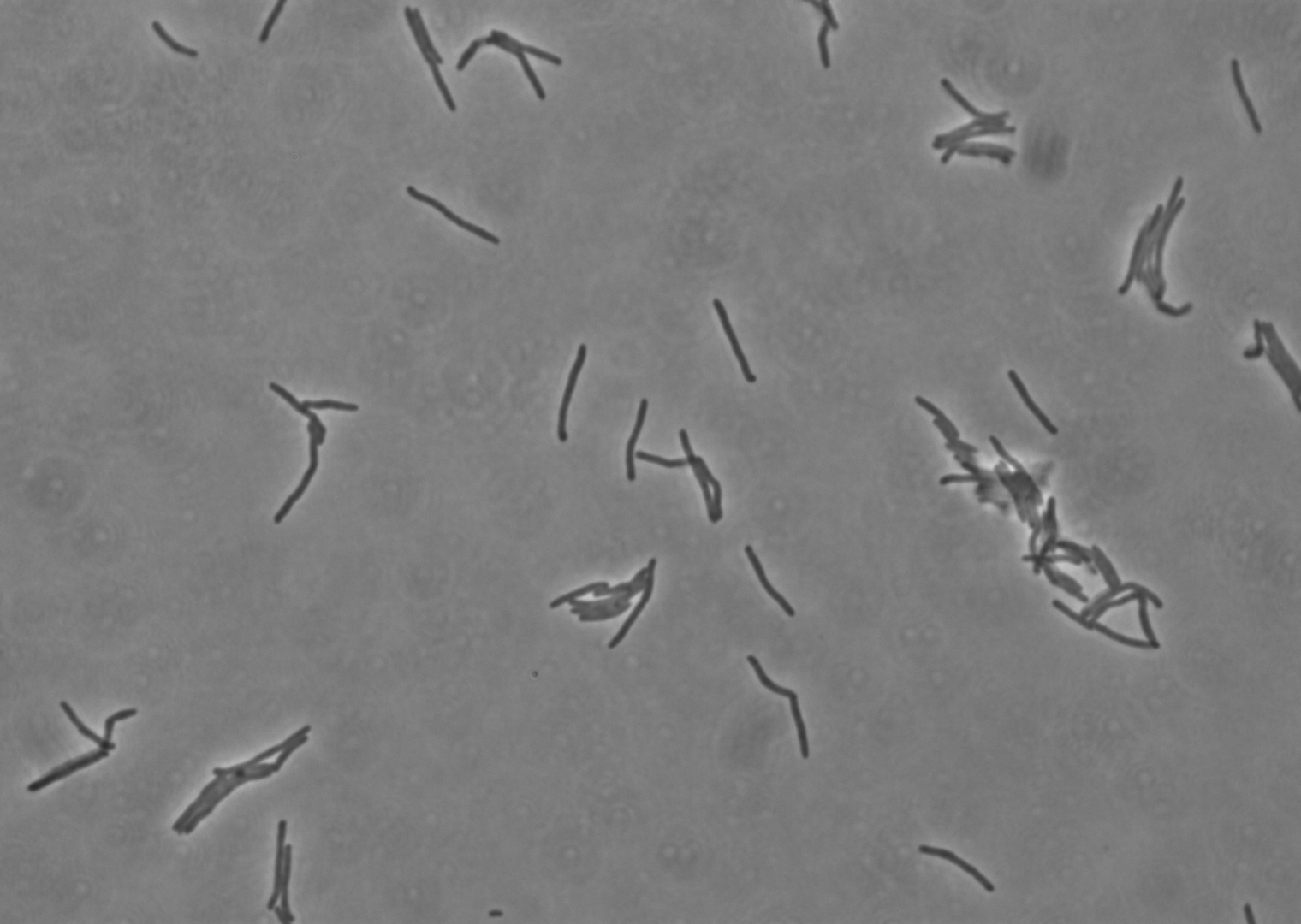

Supplement: Figure 5—figure supplement 3—source data 1. [file elife-37243-fig5-figsupp3-data1.zip › Figure 5-figure supplement 3 source data/2. Bocillin labeling- conventional microscopy /4. DCS treated/1. PC/3.tif]

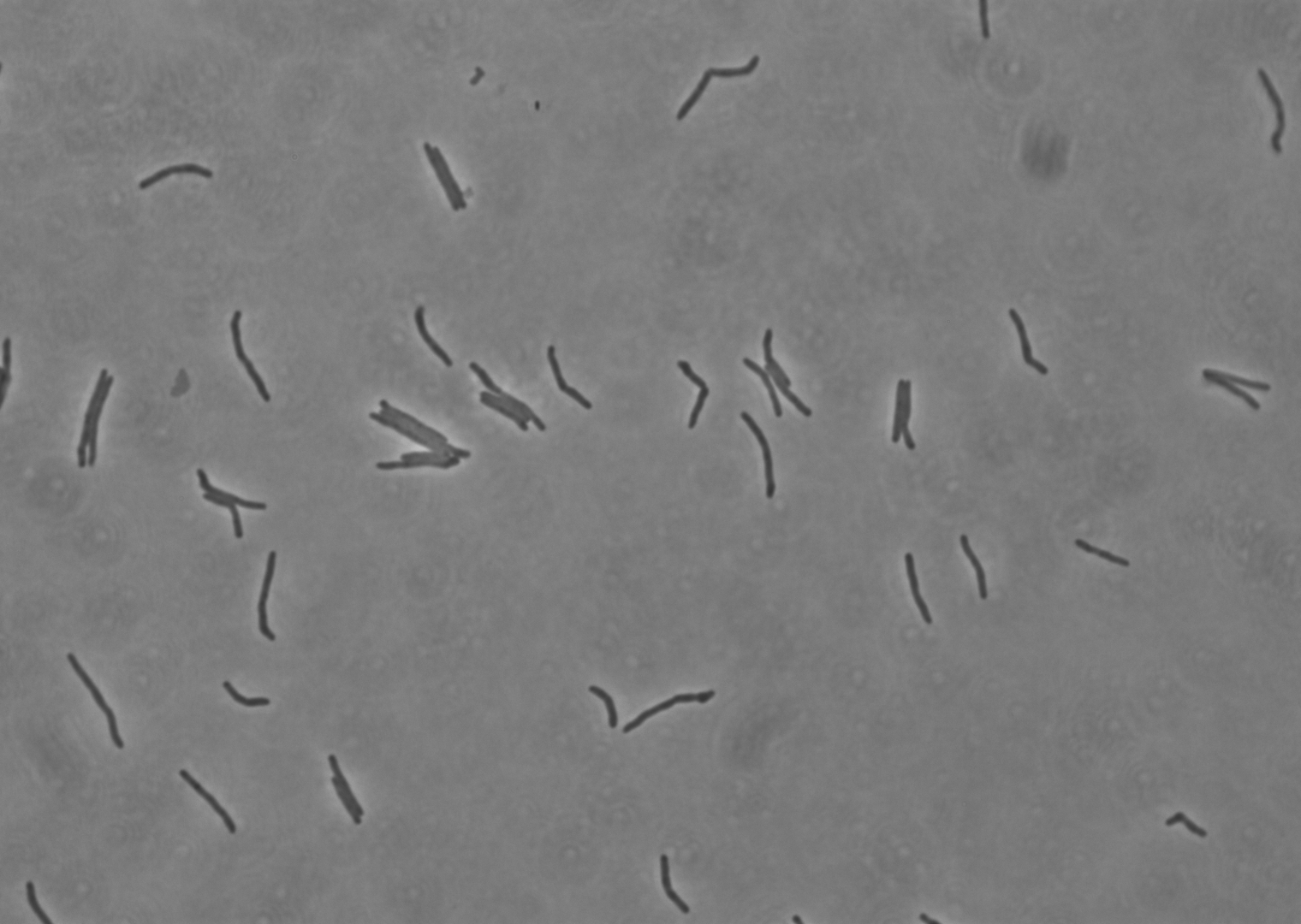

Supplement: Figure 5—figure supplement 3—source data 1. [file elife-37243-fig5-figsupp3-data1.zip › Figure 5-figure supplement 3 source data/2. Bocillin labeling- conventional microscopy /4. DCS treated/1. PC/5.tif]

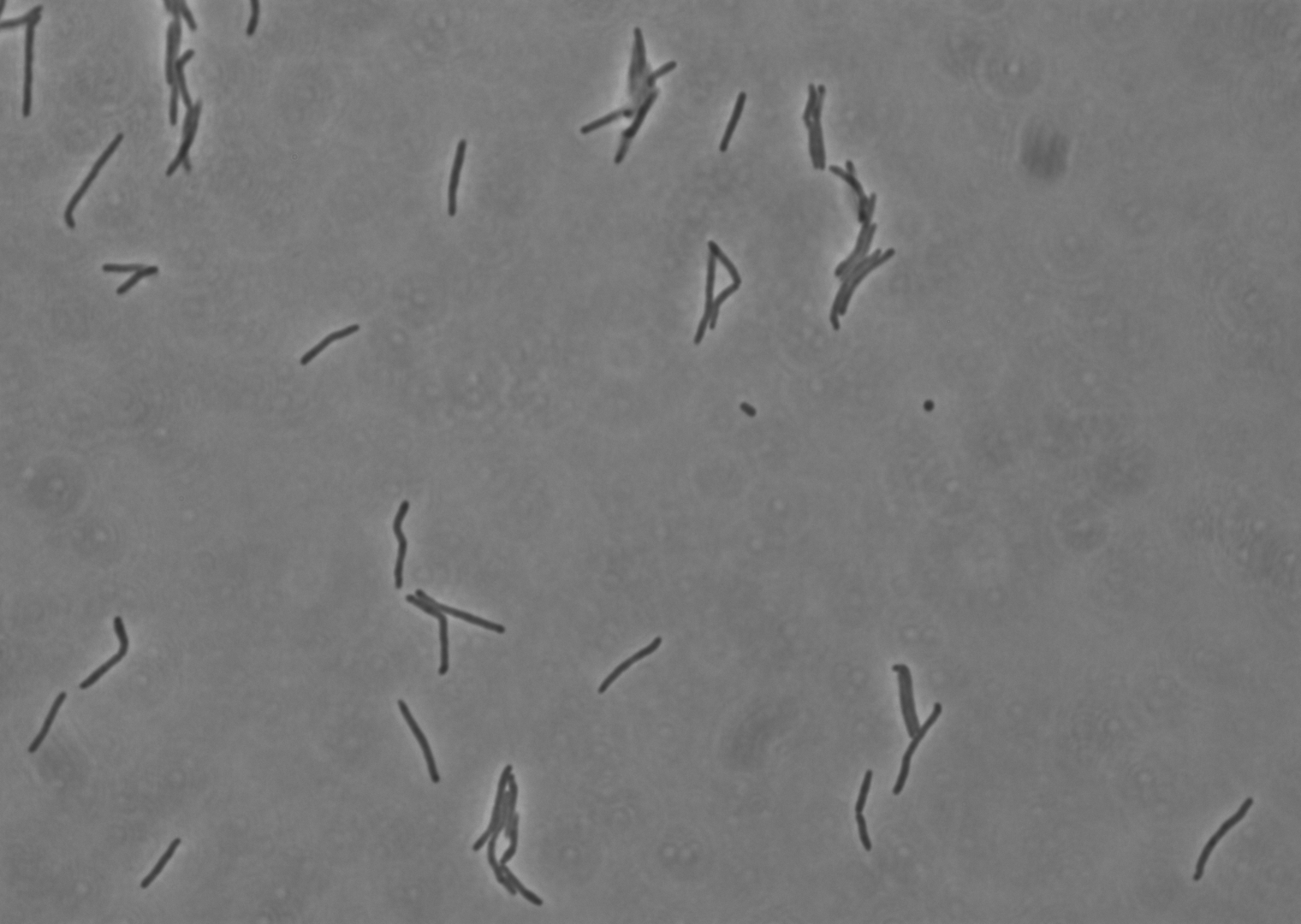

Supplement: Figure 5—figure supplement 3—source data 1. [file elife-37243-fig5-figsupp3-data1.zip › Figure 5-figure supplement 3 source data/2. Bocillin labeling- conventional microscopy /4. DCS treated/1. PC/6.tif]

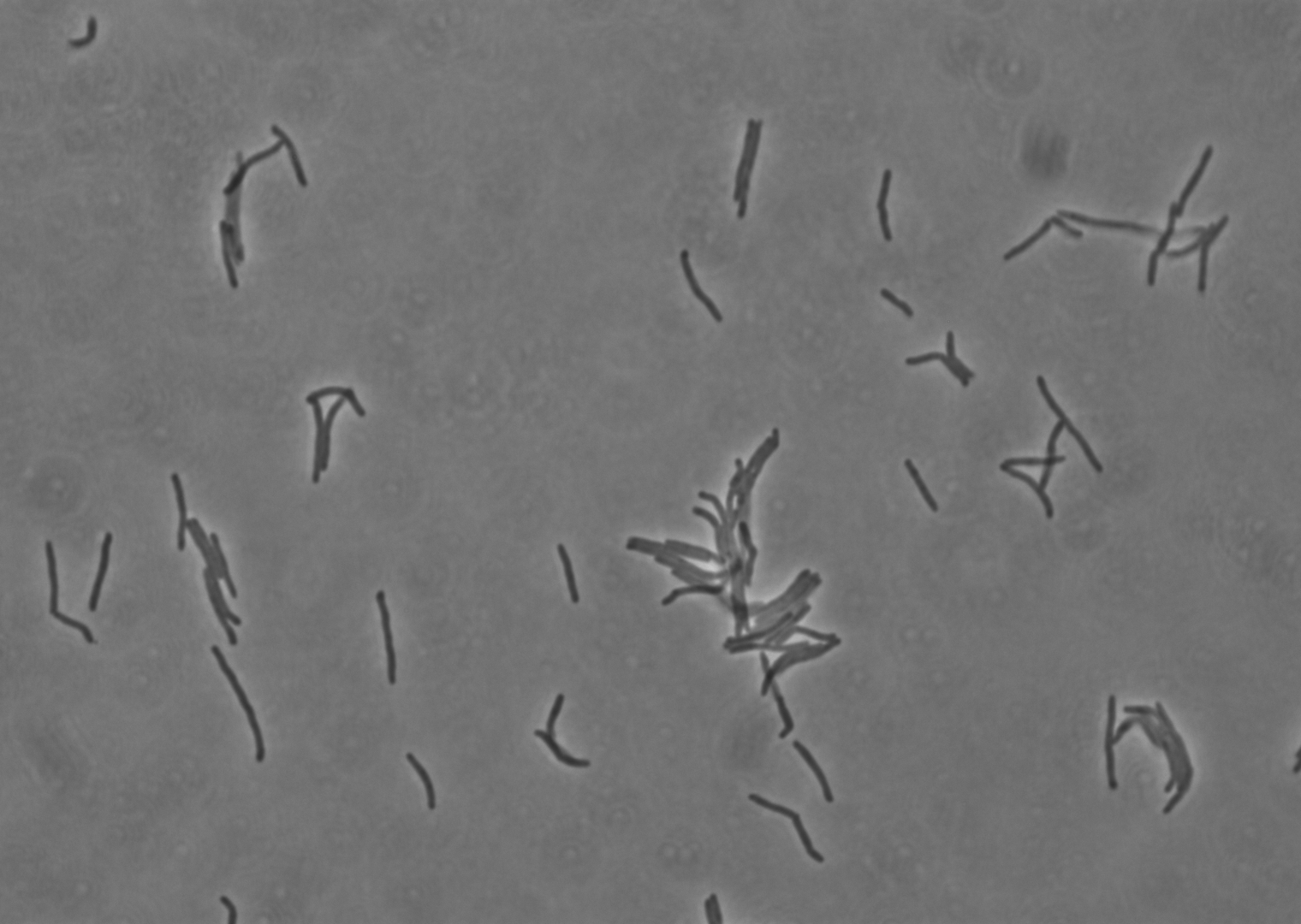

Supplement: Figure 5—figure supplement 3—source data 1. [file elife-37243-fig5-figsupp3-data1.zip › Figure 5-figure supplement 3 source data/2. Bocillin labeling- conventional microscopy /4. DCS treated/1. PC/7.tif]

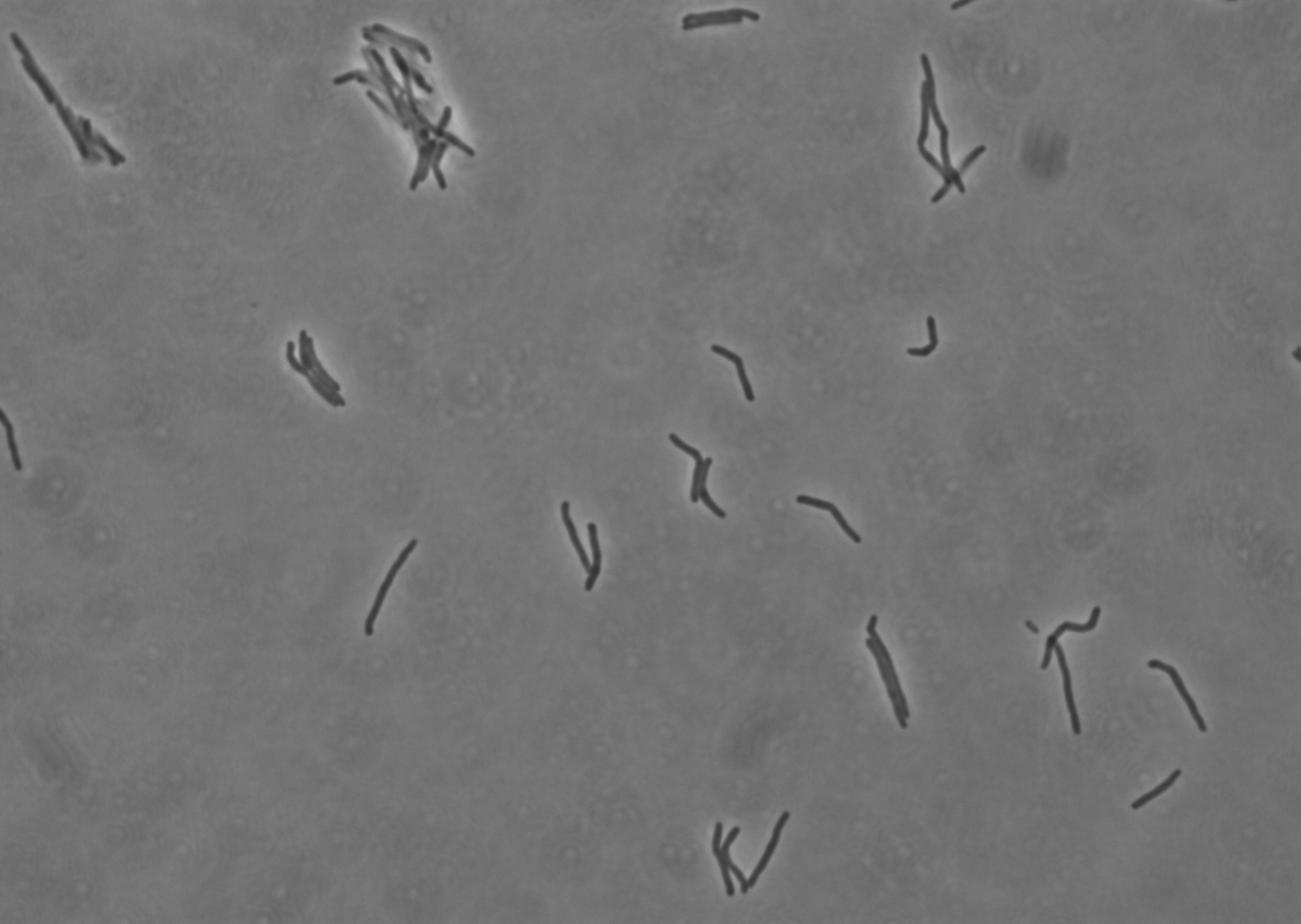

Supplement: Figure 5—figure supplement 3—source data 1. [file elife-37243-fig5-figsupp3-data1.zip › Figure 5-figure supplement 3 source data/2. Bocillin labeling- conventional microscopy /4. DCS treated/1. PC/8.tif]

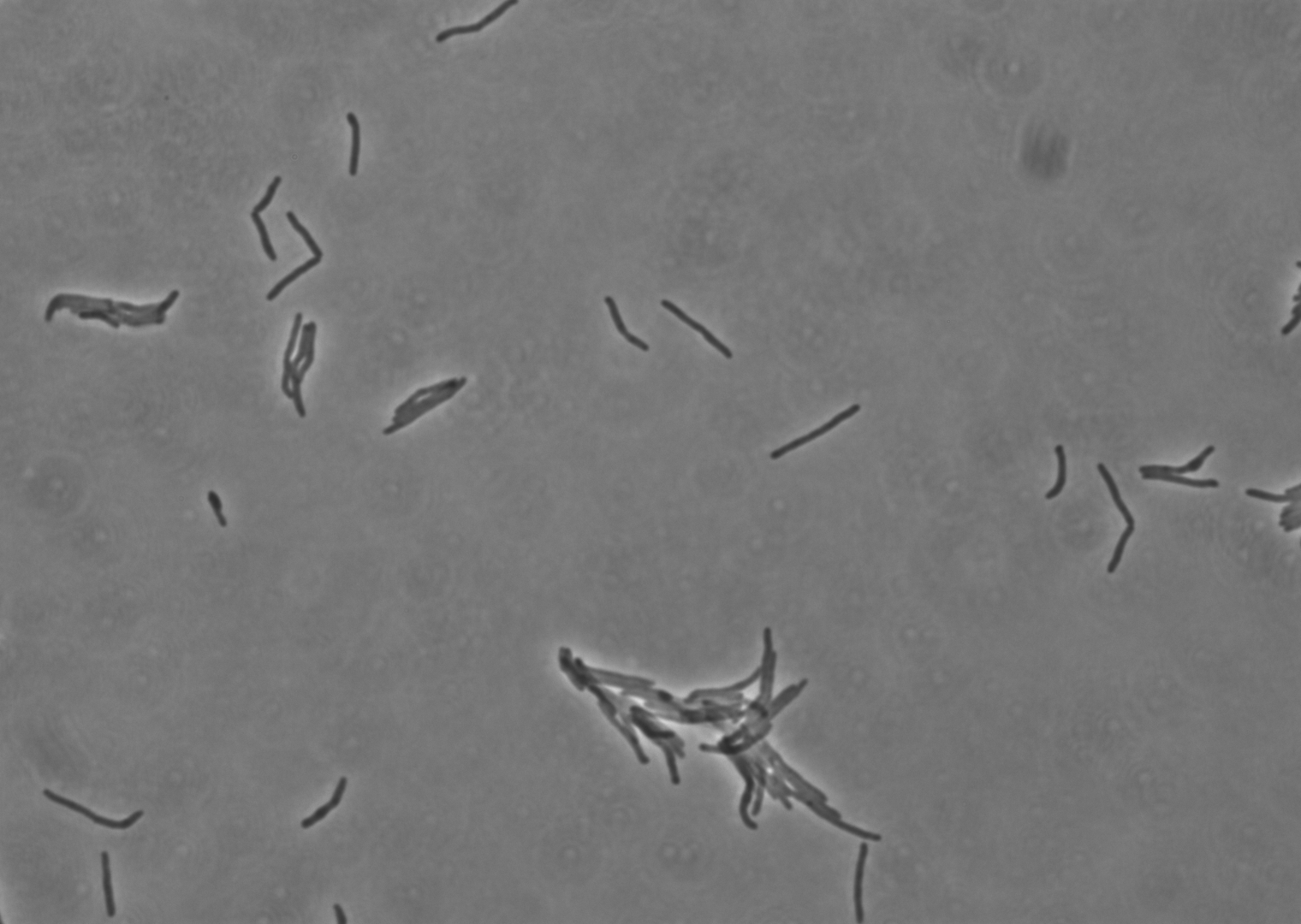

Supplement: Figure 5—figure supplement 3—source data 1. [file elife-37243-fig5-figsupp3-data1.zip › Figure 5-figure supplement 3 source data/2. Bocillin labeling- conventional microscopy /4. DCS treated/1. PC/9.tif]

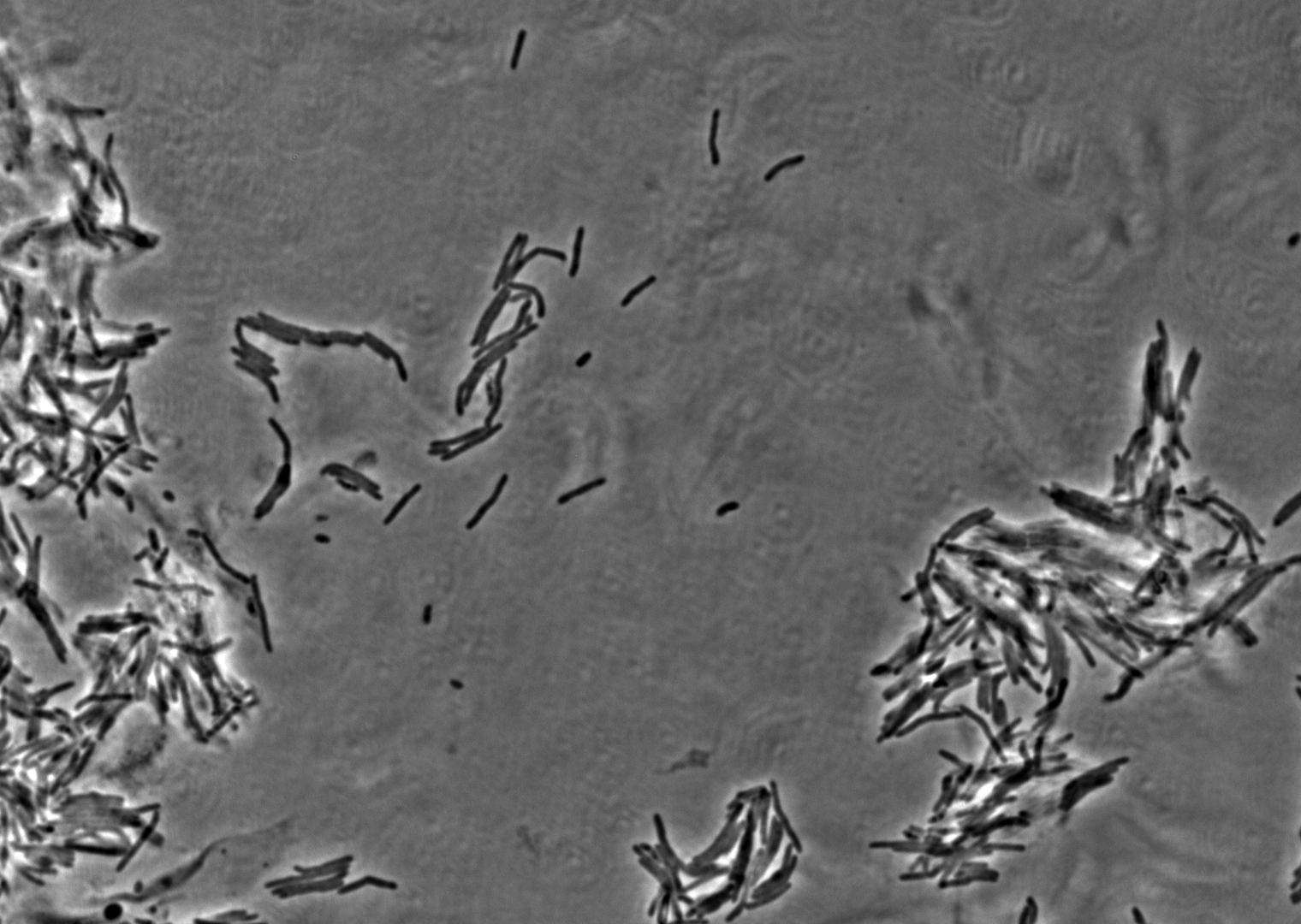

Supplement: Figure 5—figure supplement 4—source data 1. [file elife-37243-fig5-figsupp4-data1.zip › Figure 5-figure supplement 4 source data/1. T0/1. PC/1.tif]

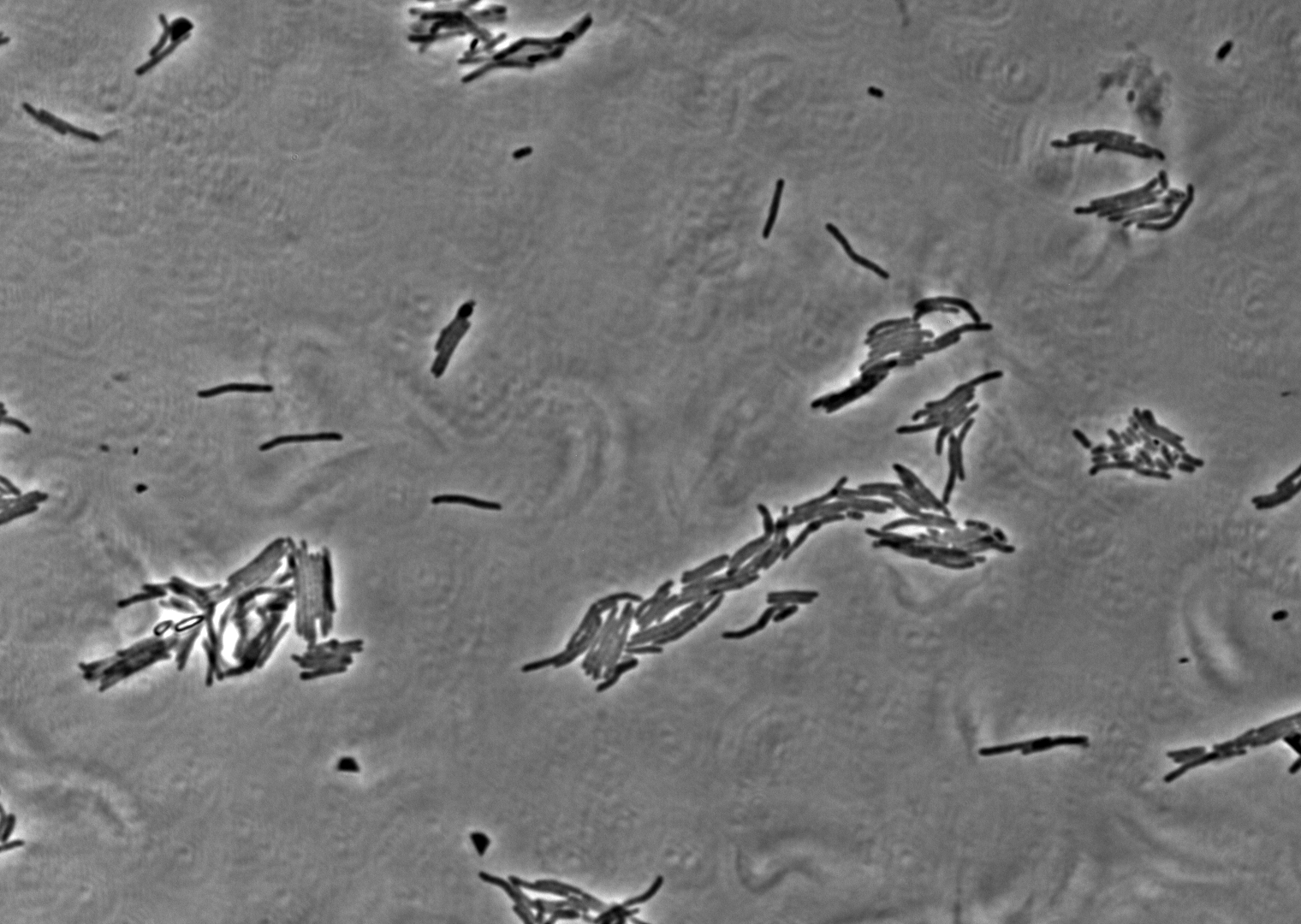

Supplement: Figure 5—figure supplement 4—source data 1. [file elife-37243-fig5-figsupp4-data1.zip › Figure 5-figure supplement 4 source data/1. T0/1. PC/2.tif]

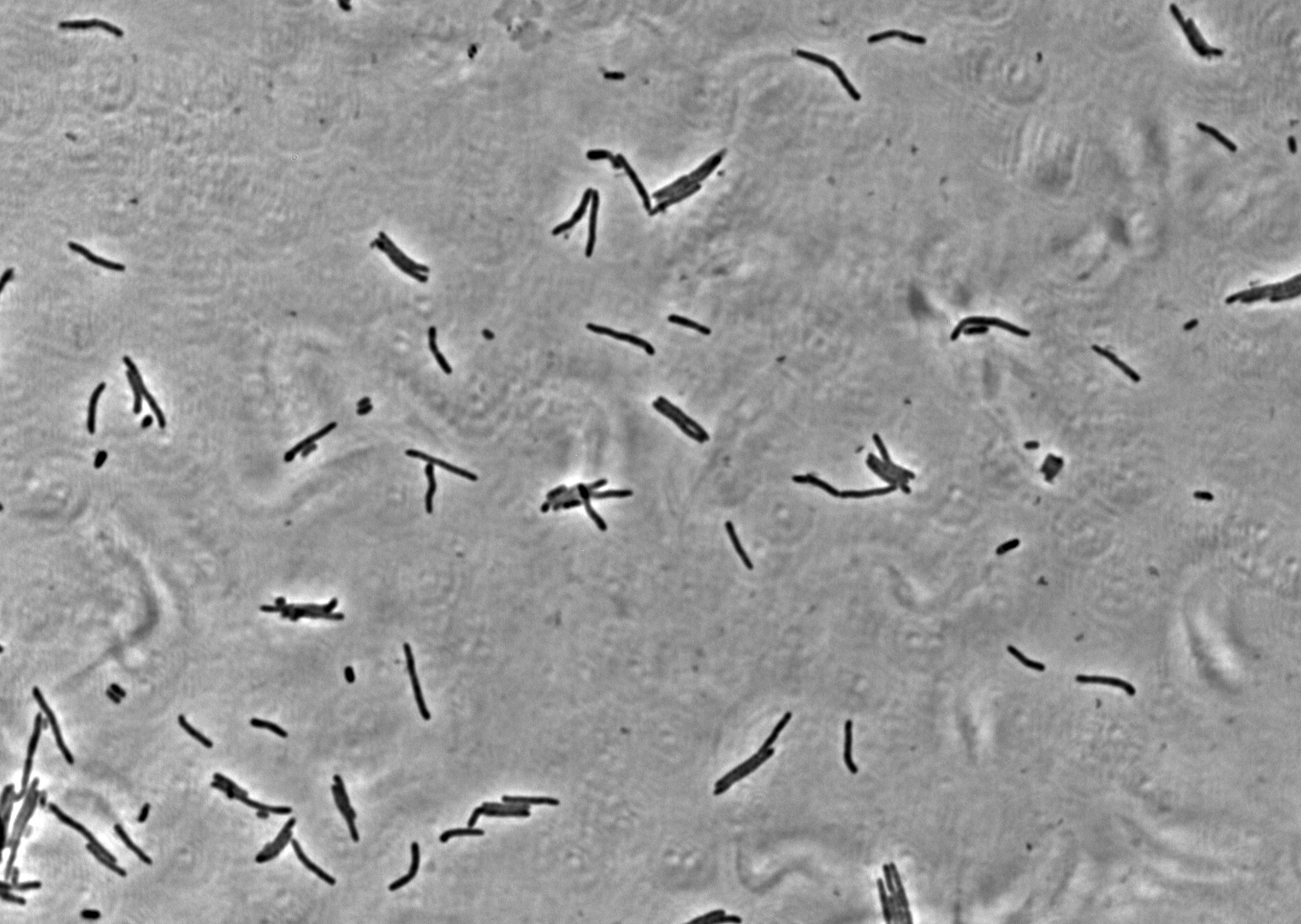

Supplement: Figure 5—figure supplement 4—source data 1. [file elife-37243-fig5-figsupp4-data1.zip › Figure 5-figure supplement 4 source data/1. T0/1. PC/3.tif]

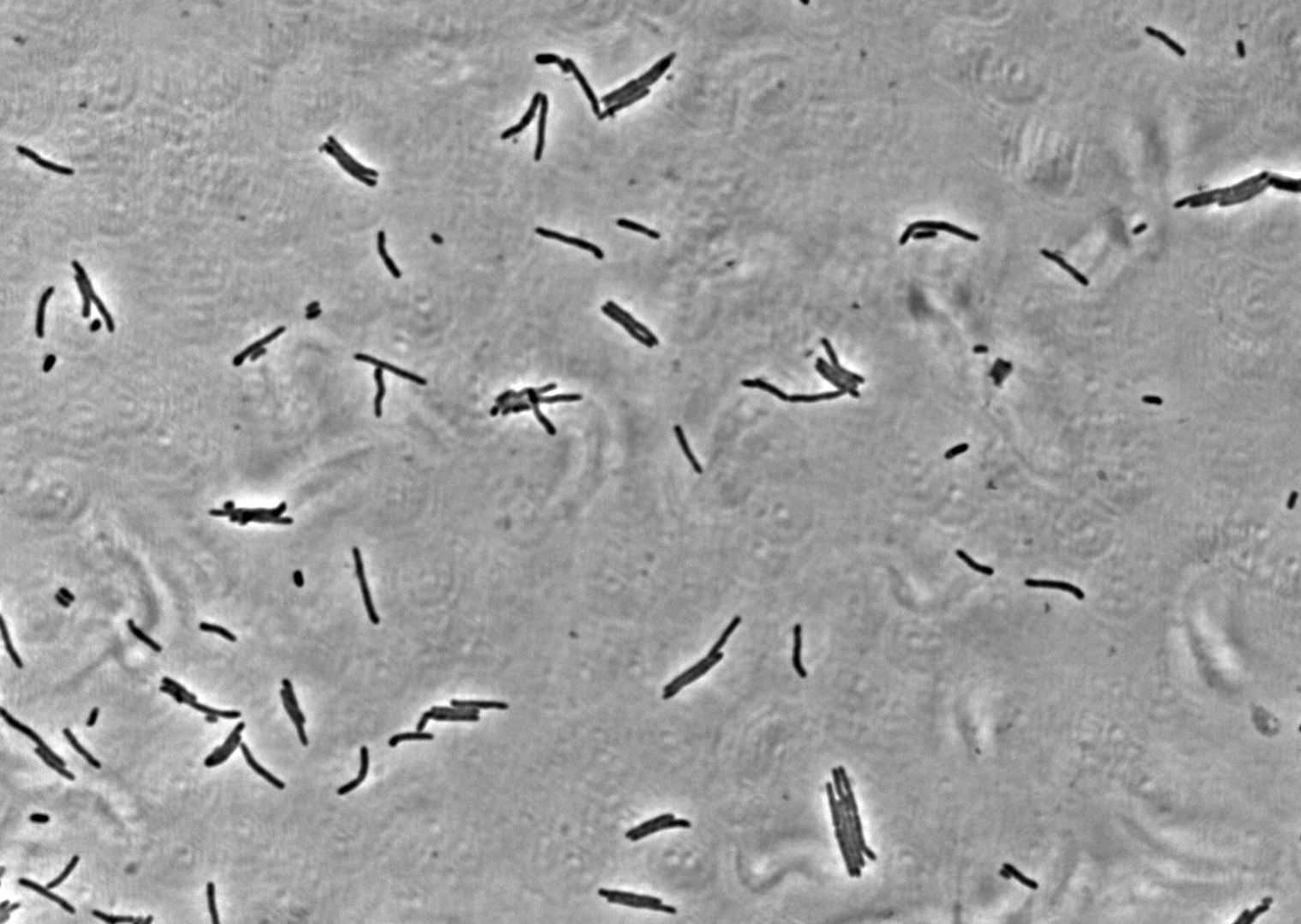

Supplement: Figure 5—figure supplement 4—source data 1. [file elife-37243-fig5-figsupp4-data1.zip › Figure 5-figure supplement 4 source data/1. T0/1. PC/4.tif]

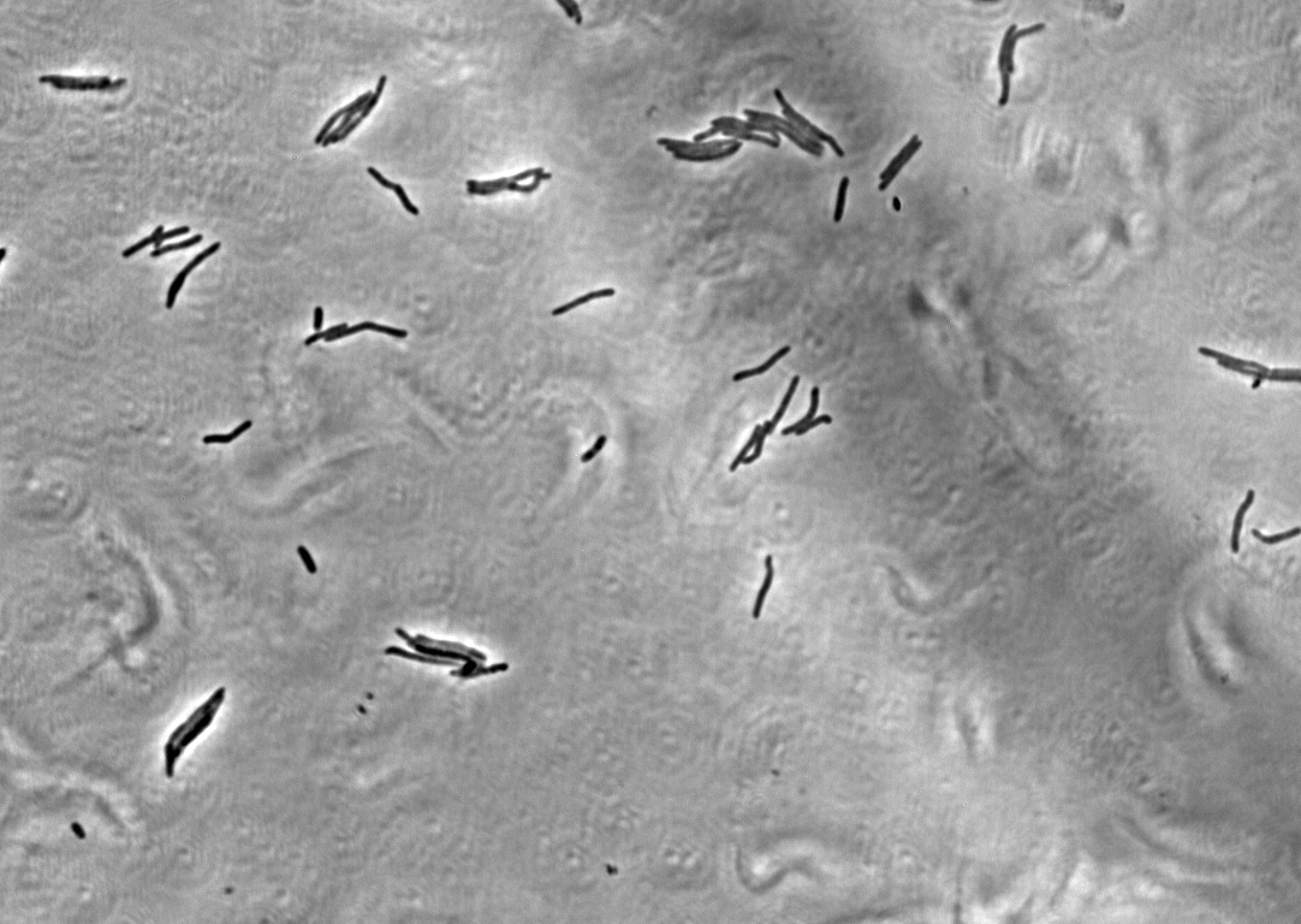

Supplement: Figure 5—figure supplement 4—source data 1. [file elife-37243-fig5-figsupp4-data1.zip › Figure 5-figure supplement 4 source data/1. T0/1. PC/5.tif]

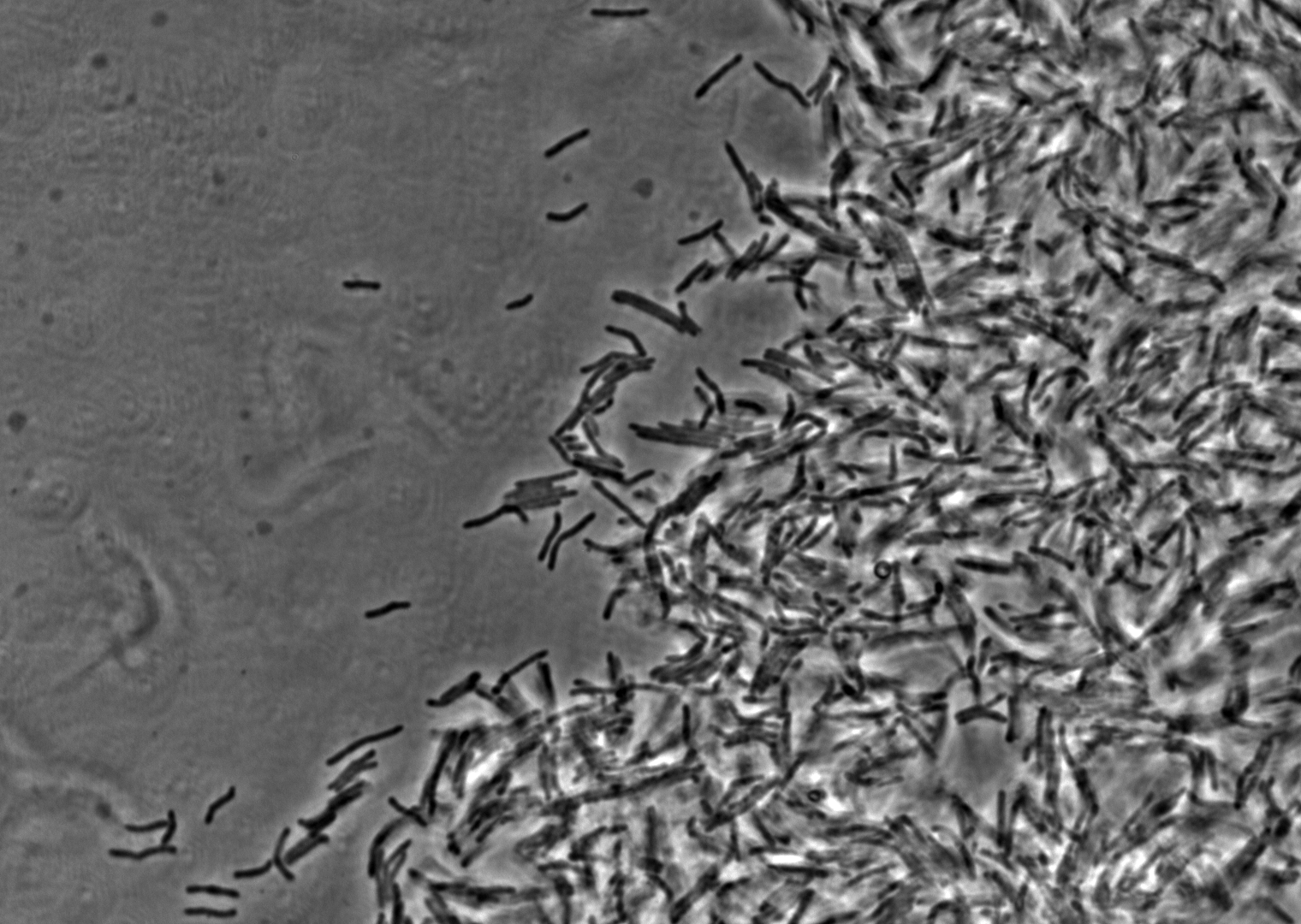

Supplement: Figure 5—figure supplement 4—source data 1. [file elife-37243-fig5-figsupp4-data1.zip › Figure 5-figure supplement 4 source data/2. 15 min/1. PC/1.tif]

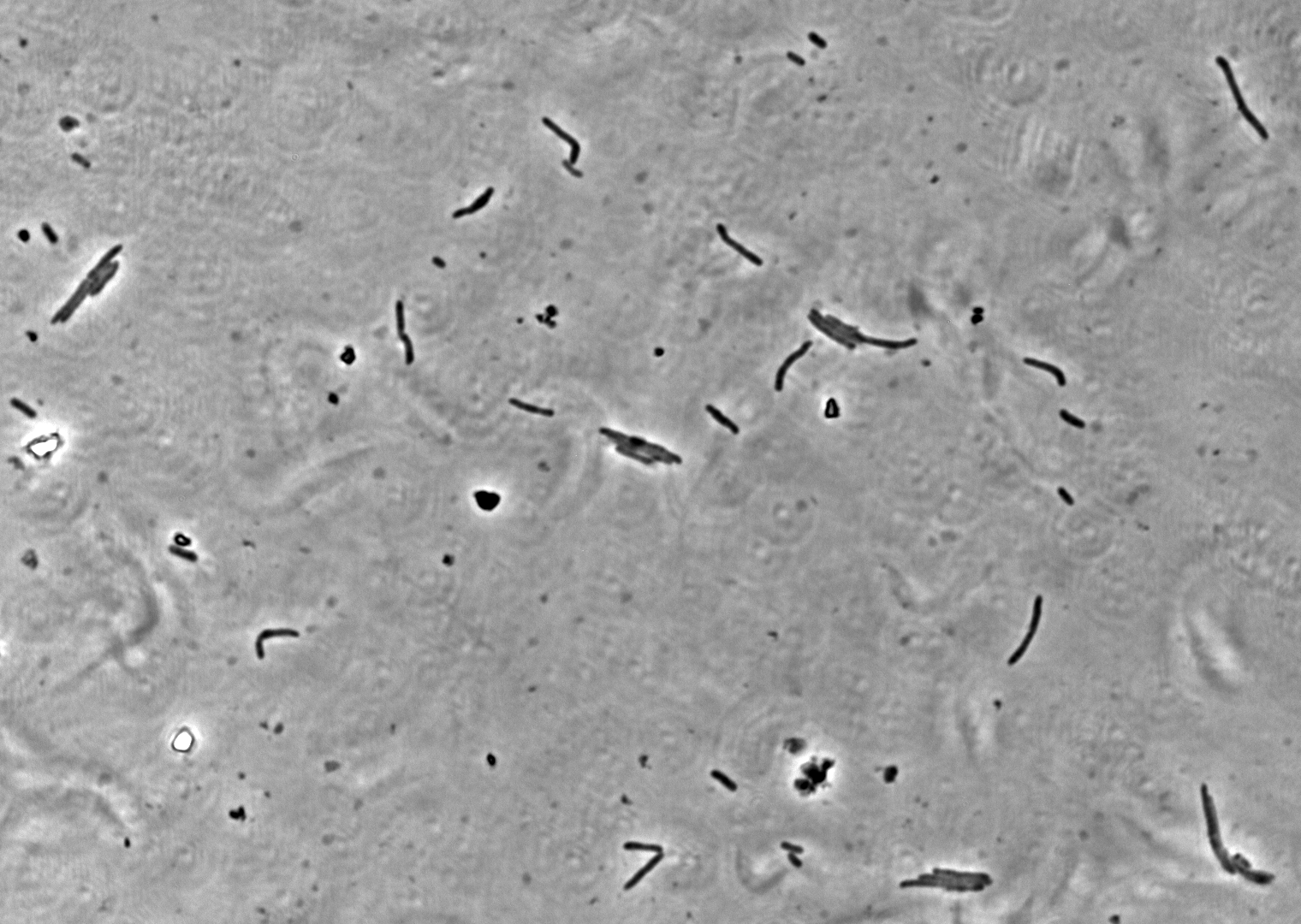

Supplement: Figure 5—figure supplement 4—source data 1. [file elife-37243-fig5-figsupp4-data1.zip › Figure 5-figure supplement 4 source data/2. 15 min/1. PC/2.tif]

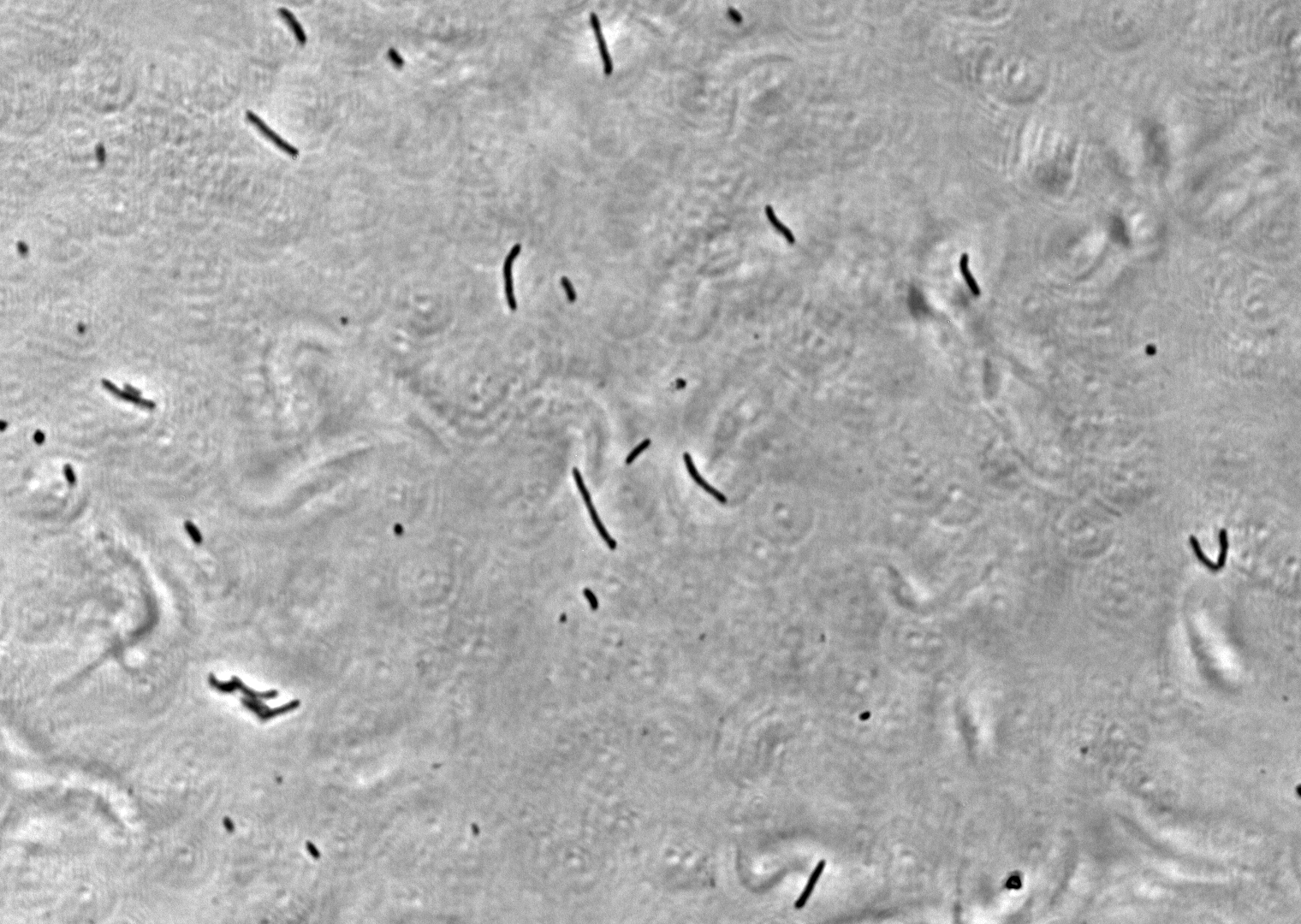

Supplement: Figure 5—figure supplement 4—source data 1. [file elife-37243-fig5-figsupp4-data1.zip › Figure 5-figure supplement 4 source data/2. 15 min/1. PC/3.tif]

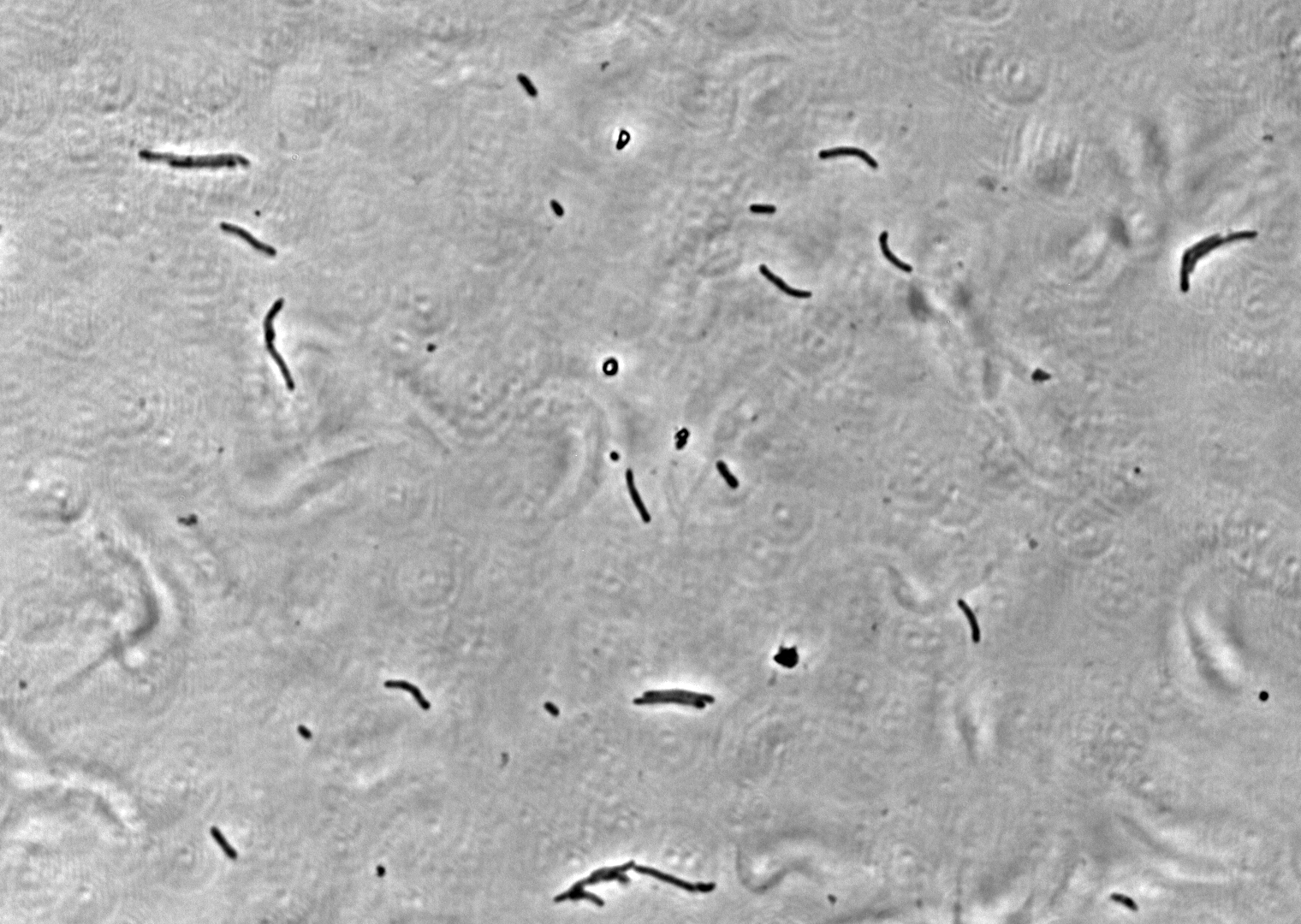

Supplement: Figure 5—figure supplement 4—source data 1. [file elife-37243-fig5-figsupp4-data1.zip › Figure 5-figure supplement 4 source data/2. 15 min/1. PC/4.tif]

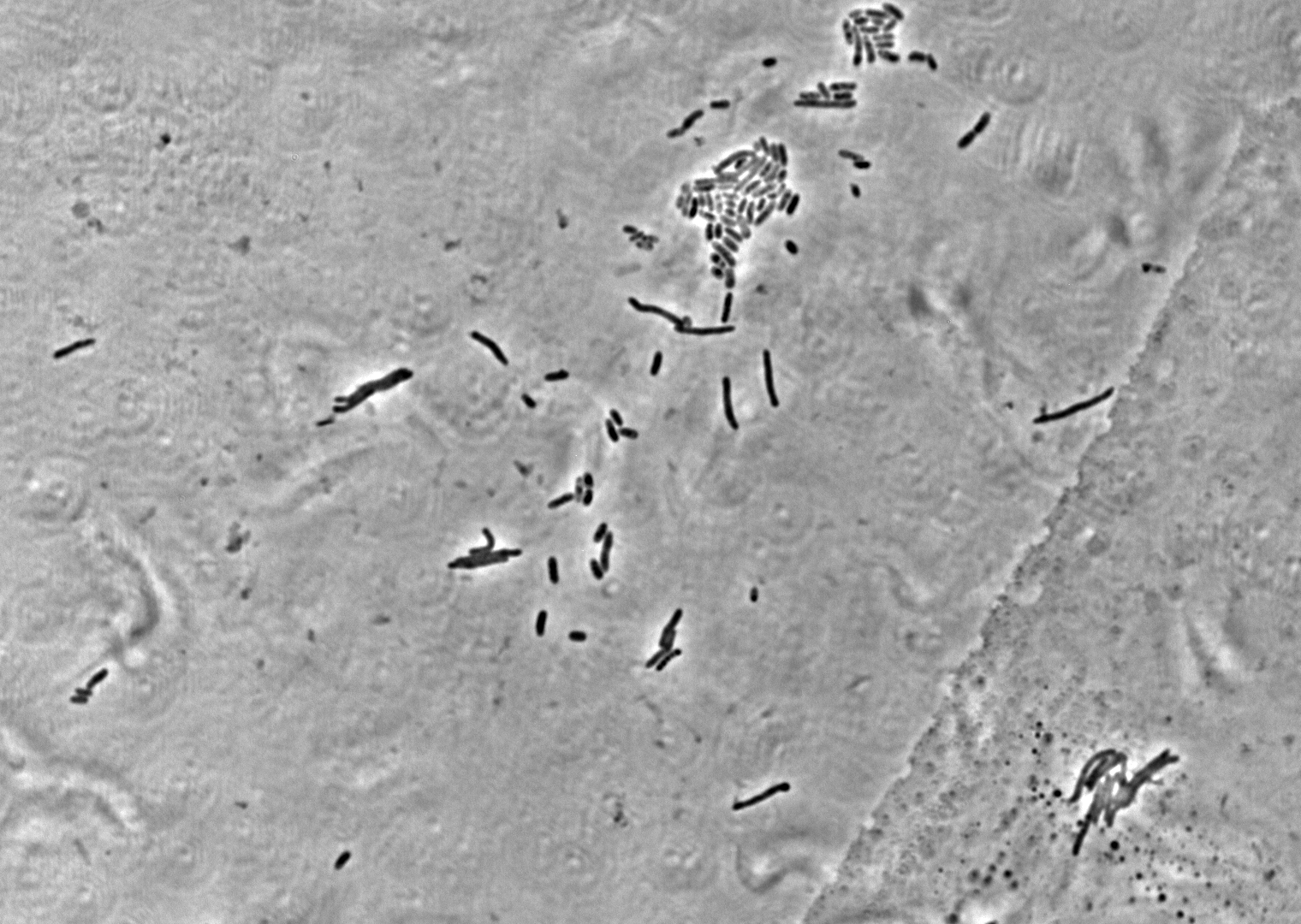

Supplement: Figure 6—source data 1. [file elife-37243-fig6-data1.zip › Figure 6 source data/1. wt 30 min/1. PC/1.tif]

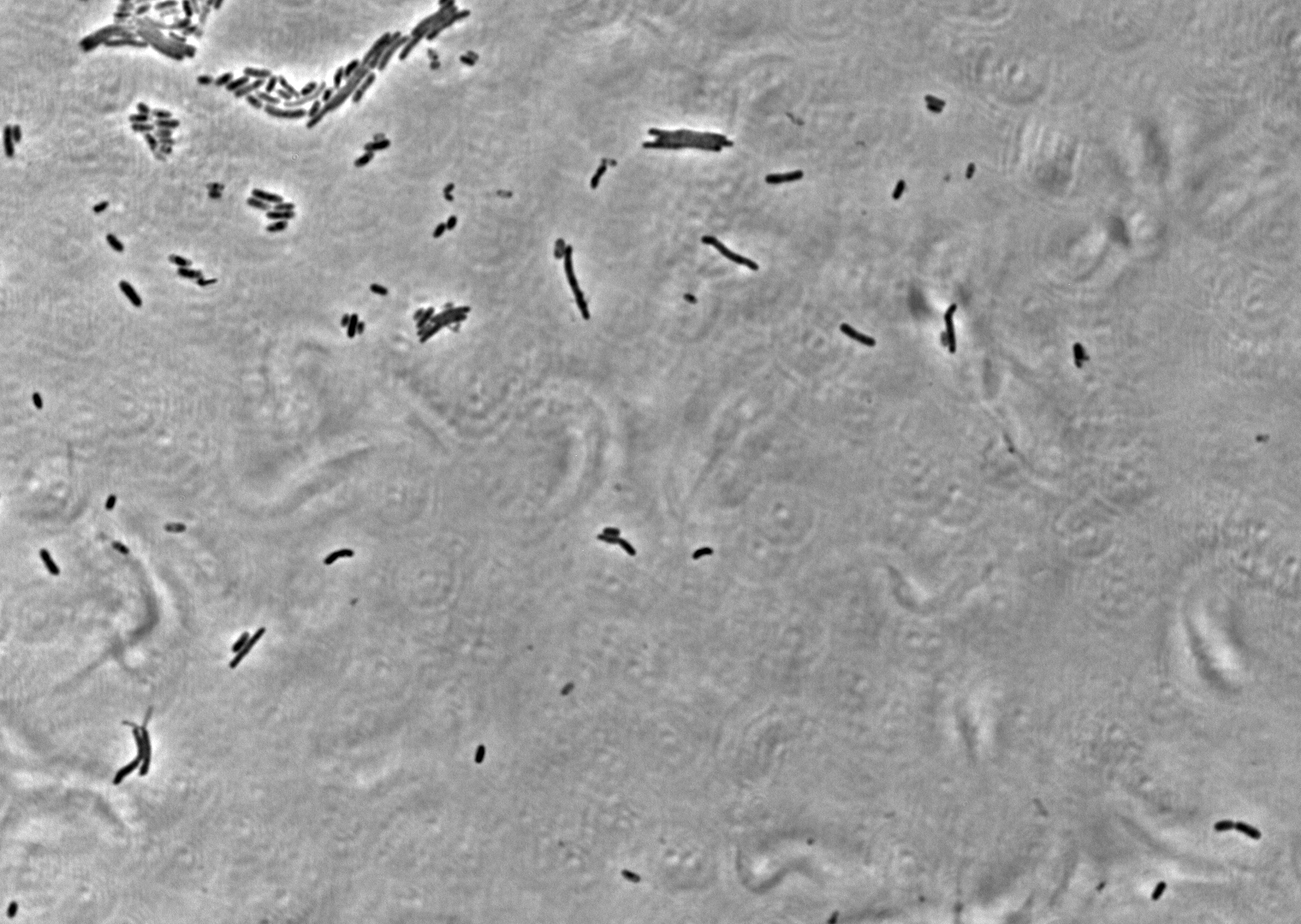

Supplement: Figure 6—source data 1. [file elife-37243-fig6-data1.zip › Figure 6 source data/1. wt 30 min/1. PC/2.tif]

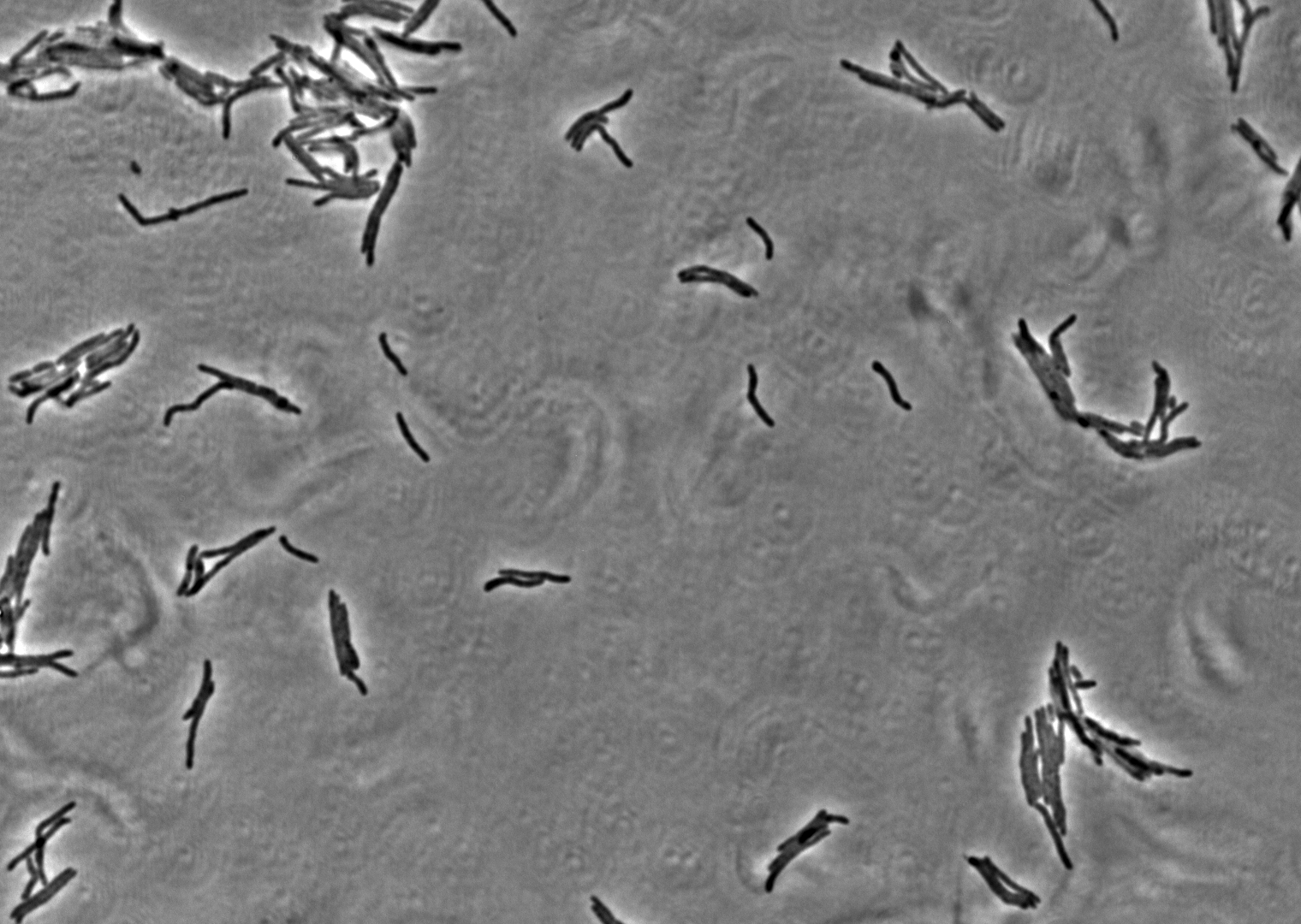

Supplement: Figure 6—source data 1. [file elife-37243-fig6-data1.zip › Figure 6 source data/1. wt 30 min/1. PC/3.tif]

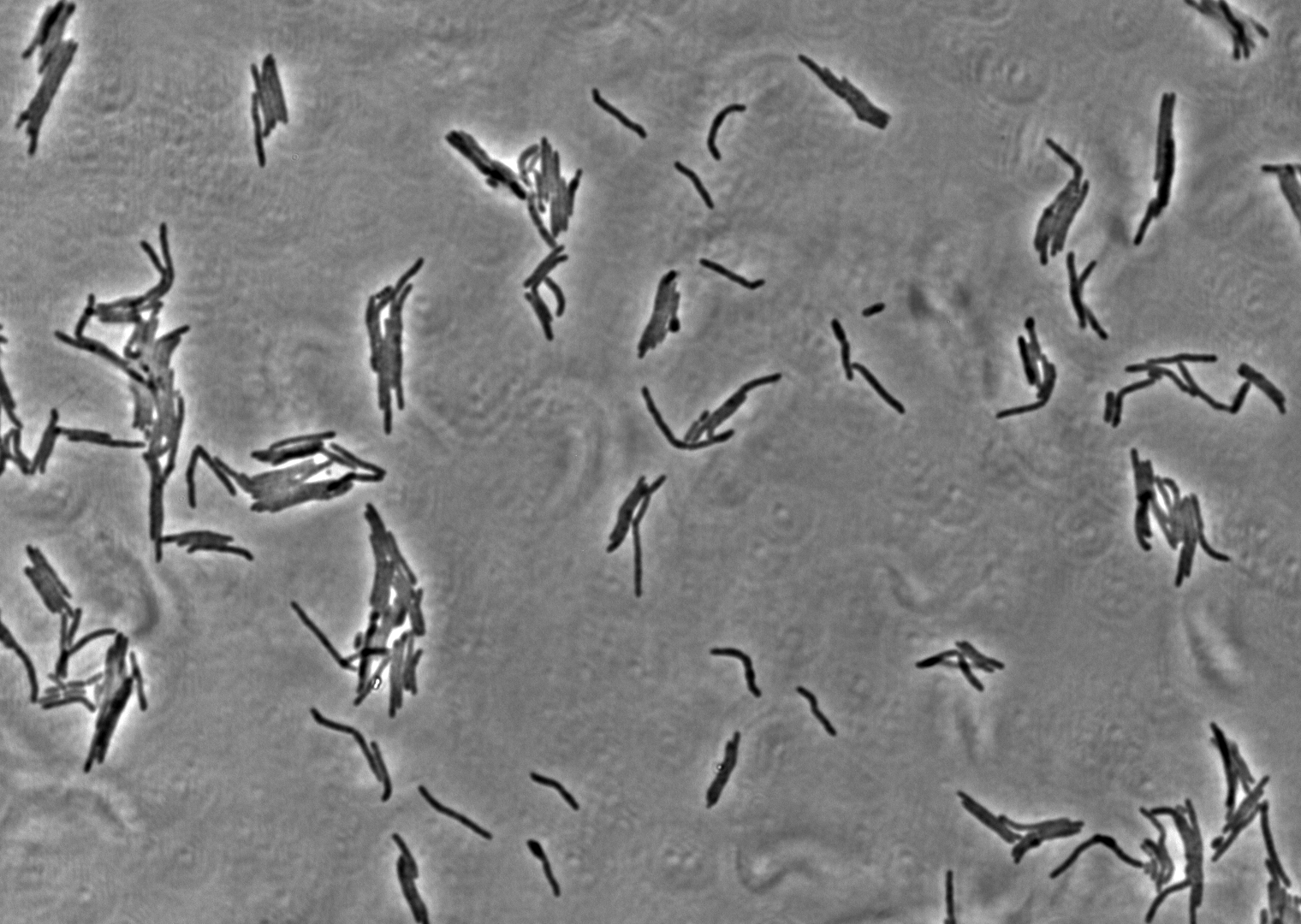

Supplement: Figure 6—source data 1. [file elife-37243-fig6-data1.zip › Figure 6 source data/1. wt 30 min/1. PC/4.tif]

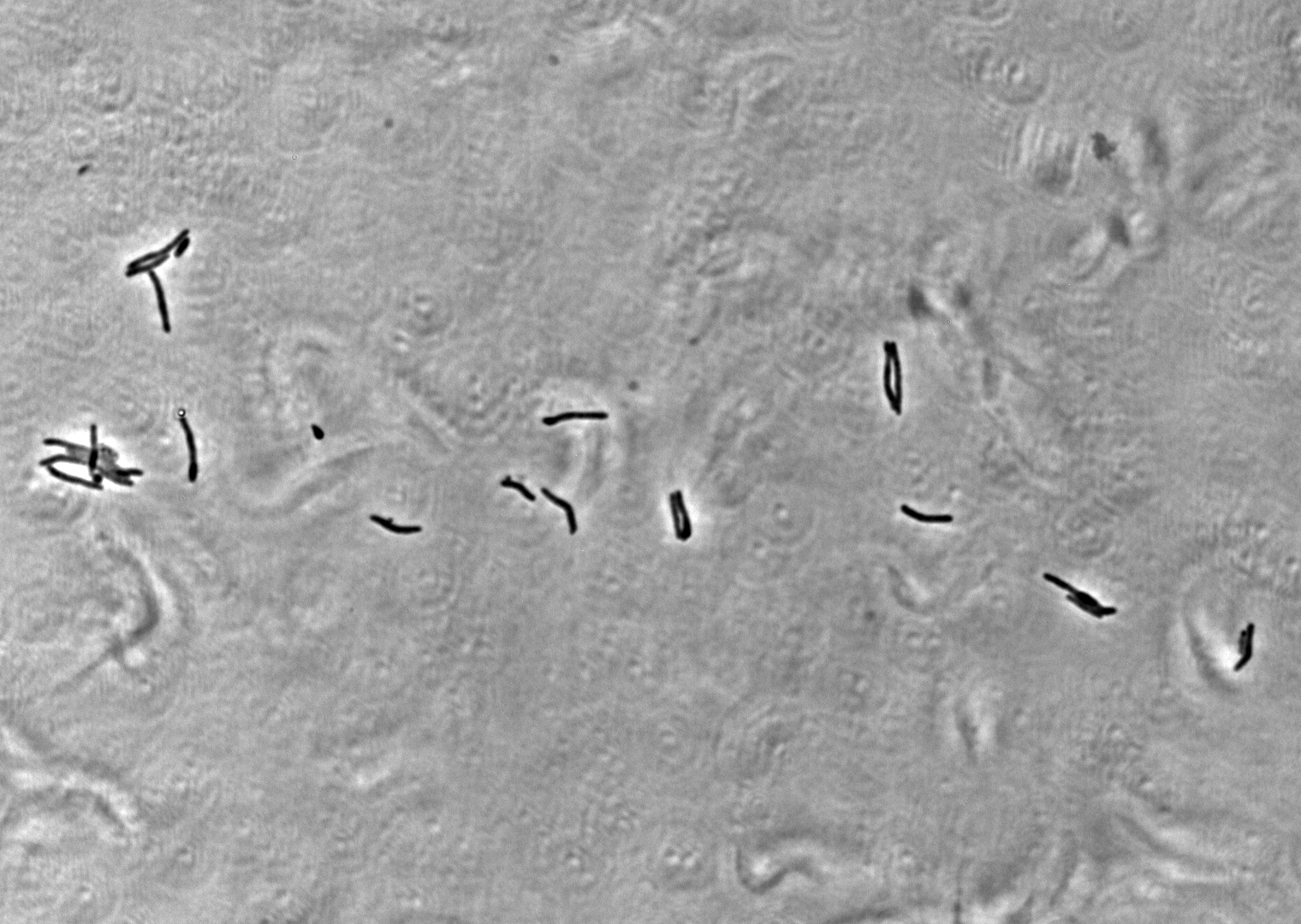

Supplement: Figure 6—source data 1. [file elife-37243-fig6-data1.zip › Figure 6 source data/1. wt 30 min/1. PC/5.tif]

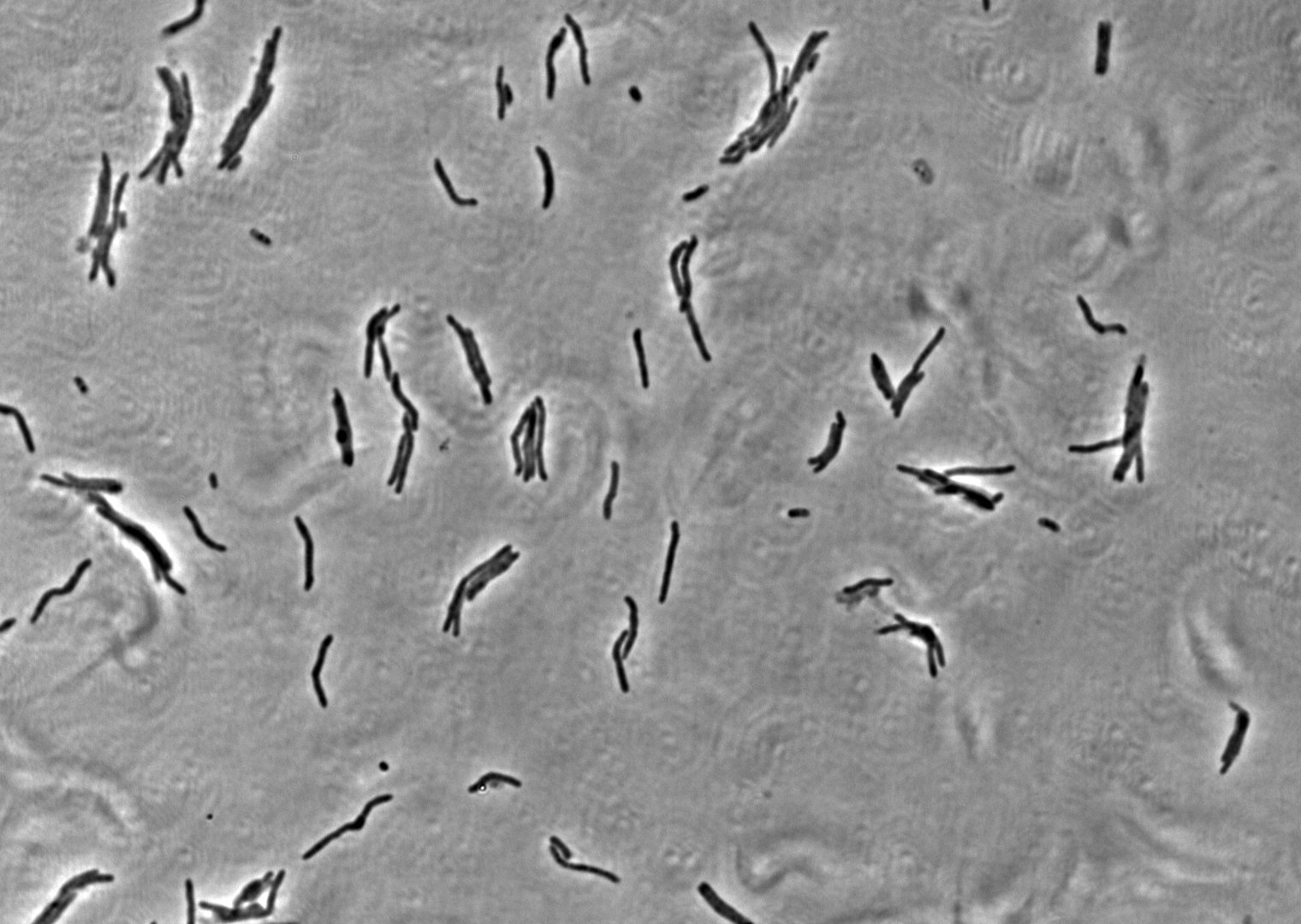

Supplement: Figure 6—source data 1. [file elife-37243-fig6-data1.zip › Figure 6 source data/2. wt + enzymes 30 min/1. PC/1.tif]

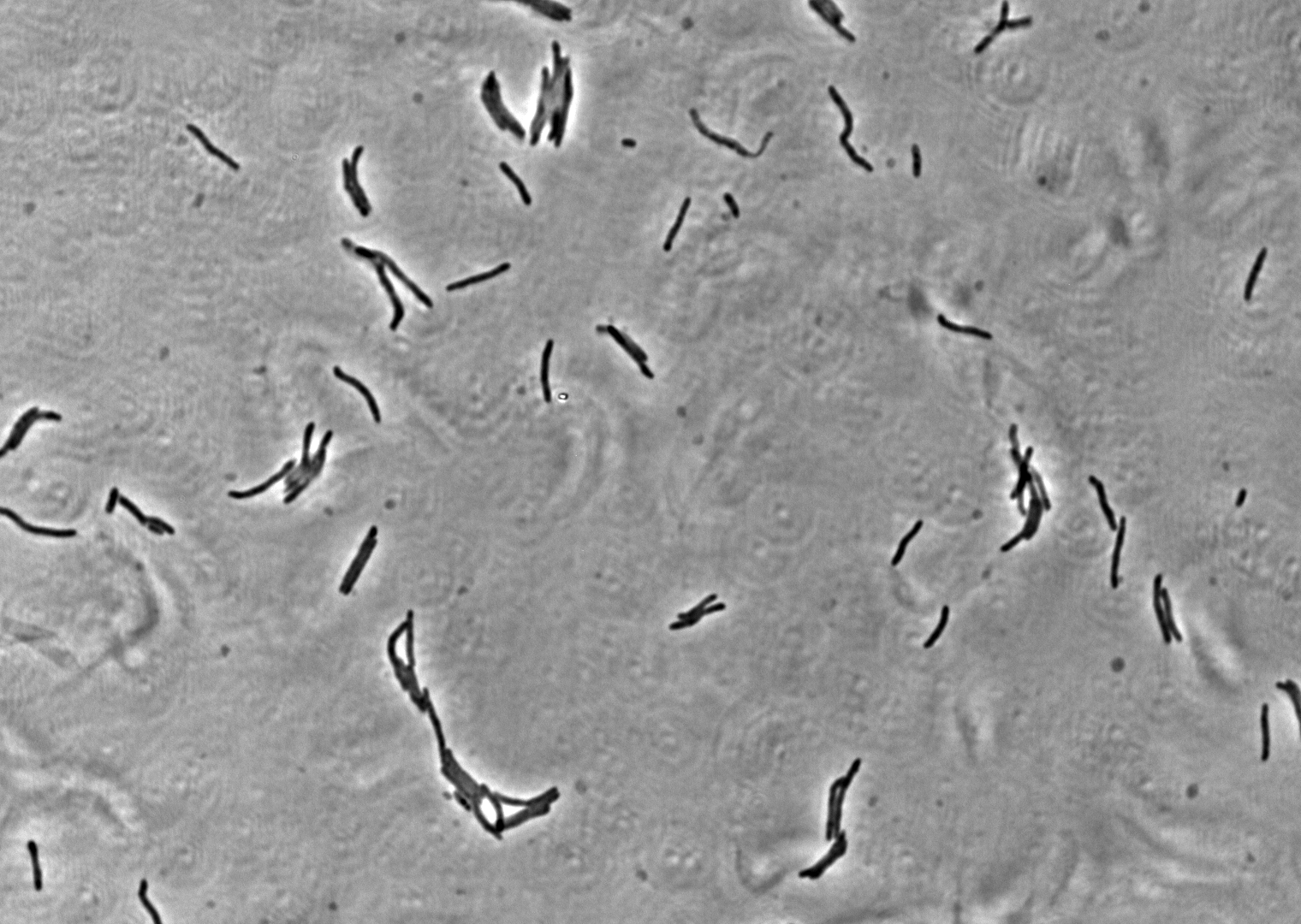

Supplement: Figure 6—source data 1. [file elife-37243-fig6-data1.zip › Figure 6 source data/2. wt + enzymes 30 min/1. PC/2.tif]

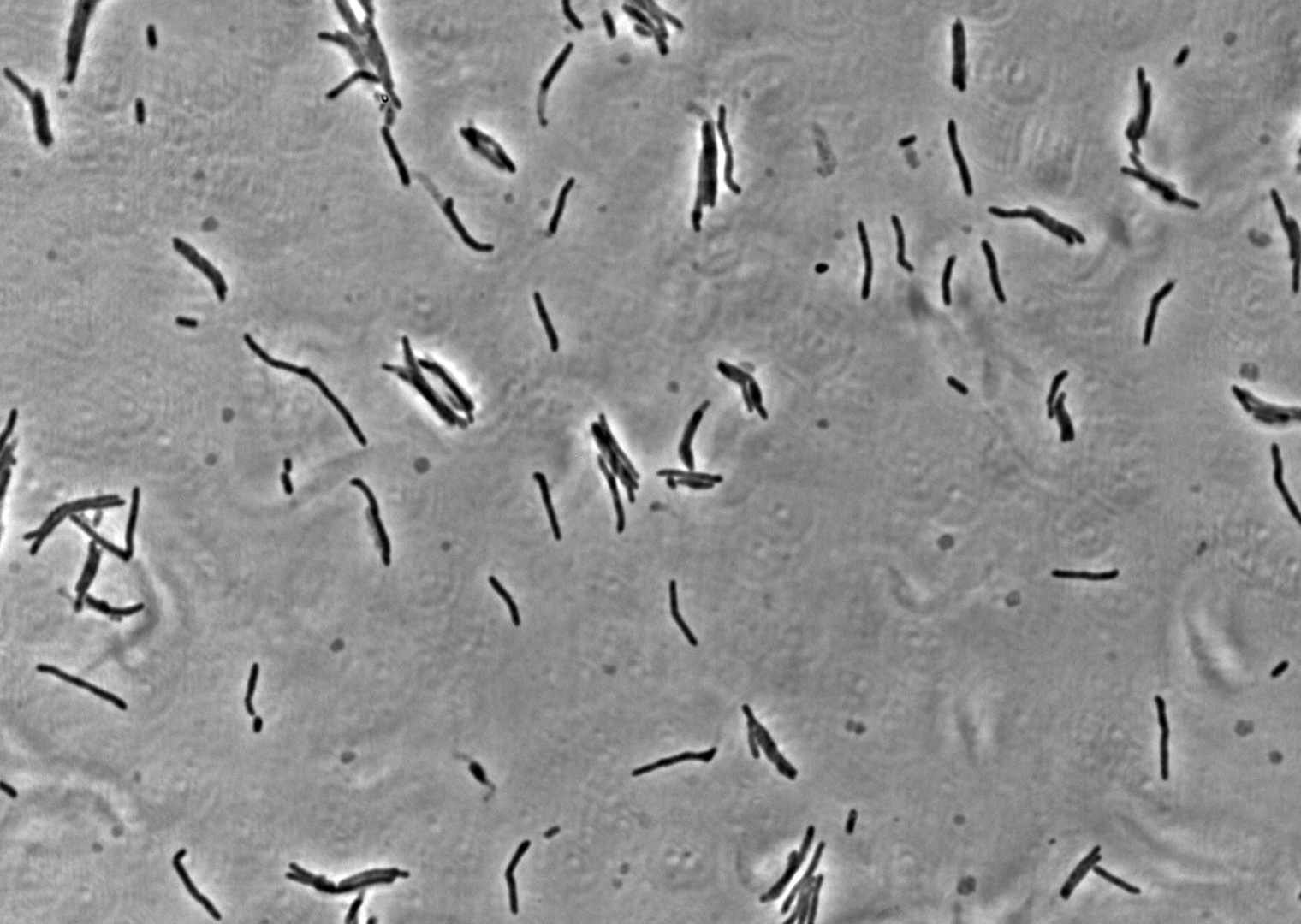

Supplement: Figure 6—source data 1. [file elife-37243-fig6-data1.zip › Figure 6 source data/2. wt + enzymes 30 min/1. PC/3.tif]

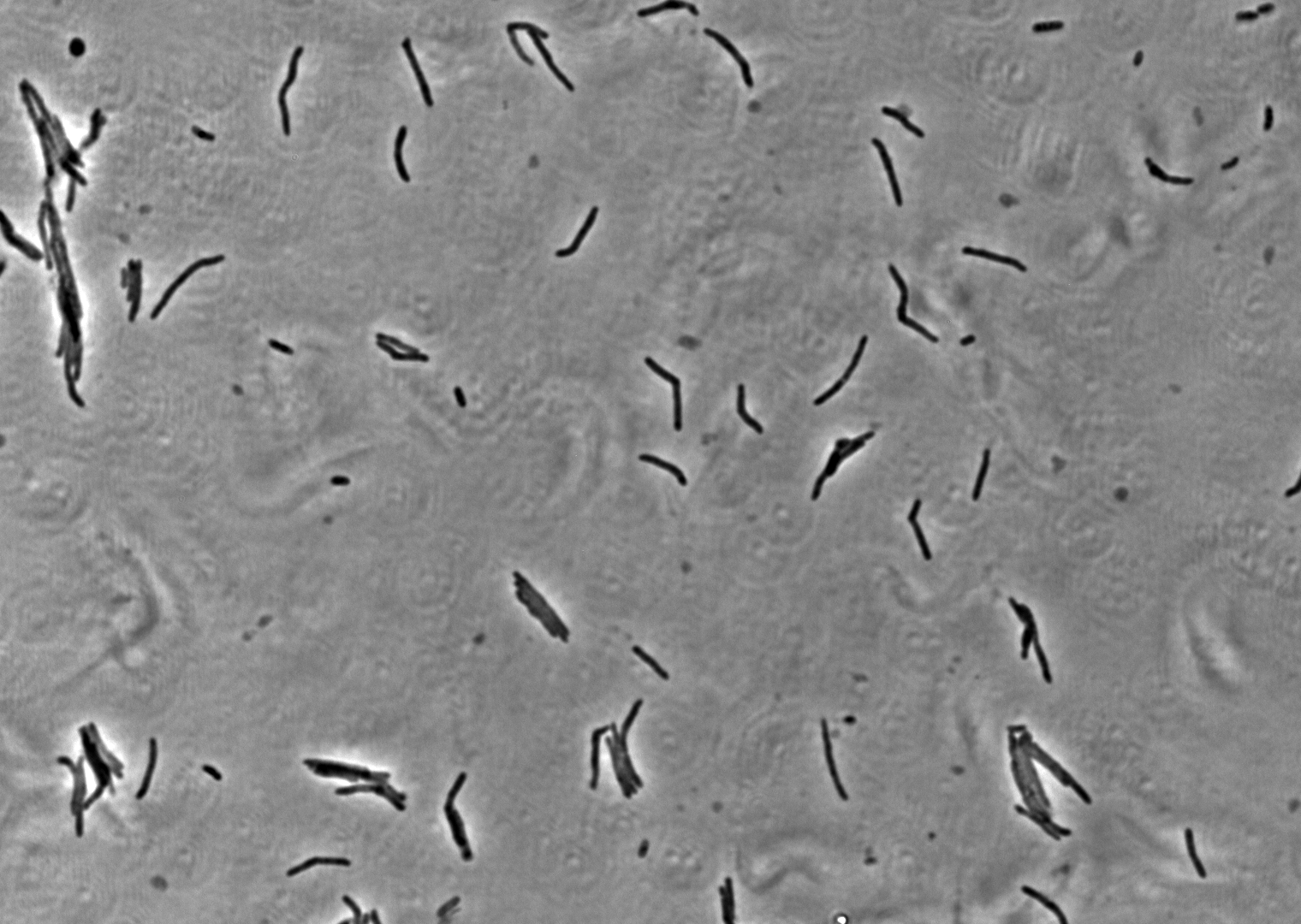

Supplement: Figure 6—source data 1. [file elife-37243-fig6-data1.zip › Figure 6 source data/2. wt + enzymes 30 min/1. PC/4.tif]
